# Microstructure and in-depth proteomic analysis of *Perna viridis* shell

**DOI:** 10.1371/journal.pone.0219699

**Published:** 2019-07-19

**Authors:** Zhi Liao, Yu-ting Jiang, Qi Sun, Mei-hua Fan, Jian-xin Wang, Hai-ying Liang

**Affiliations:** 1 Laboratory of Marine Biological Source and Molecular Engineering, College of Marine Science, Zhejiang Ocean University, Zhoushan, Zhejiang, P.R. China; 2 Fisheries College, Guangdong Ocean University, Zhanjiang, Guangdong, P.R. China; Dalian Ocean University, CHINA

## Abstract

For understanding the structural characteristics and the proteome of *Perna* shell, the microstructure, polymorph, and protein composition of the adult *Perna viridis* shell were investigated. The *P*. *viridis* shell have two distinct mineral layers, myostracum and nacre, with the same calcium carbonate polymorph of aragonite, determined by scanning electron microscope, Fourier transform infrared spectroscopy, and x-ray crystalline diffraction. Using Illumina sequencing, the mantle transcriptome of *P*. *viridis* was investigated and a total of 69,859 unigenes was generated. Using a combined proteomic/transcriptomic approach, a total of 378 shell proteins from *P*. *viridis* shell were identified, in which, 132 shell proteins identified with more than two matched unique peptides. Of the 132 shell proteins, 69 are exclusive to the nacre, 12 to the myostracum, and 51 are shared by both. The Myosin-tail domain containing proteins, Filament-like proteins, and Chitin-binding domain containing proteins represent the most abundant molecules. In addition, the shell matrix proteins (SMPs) containing biomineralization-related domains, such as Kunitz, A2M, WAP, EF-hand, PDZ, VWA, Collagen domain, and low complexity regions with abundant certain amino acids, were also identified from *P*. *viridis* shell. Collagenase and chitinase degradation can significantly change the morphology of the shell, indicating the important roles of collagen and chitin in the shell formation and the muscle-shell attachment. Our results present for the first time the proteome of *P*. *viridis* shell and increase the knowledge of SMPs in this genus.

## Introduction

The bivalve shell, consisting of calcium carbonate crystals within an organic matrix, has been investigated as a typical biomineralization model for many years [1–4]. The bivalve shell presents superior mechanical properties, such as stiffness, fracture toughness, and tensile strength, because of the complex architecture and involvement of biological macromolecules [5–7]. It is well known that there are three major polymorphs of calcium carbonate: calcite, aragonite, and vaterite. Of these, the two most thermodynamically stable structures, calcite and aragonite, are deposited extensively as biominerals [8]. In general, most adult bivalve shells are composed of calcite and/or aragonite and consist of various microstructures (or layers) with different morphologies, such as prismatic, nacreous, myostracum, foliated, cross-lamellar, granular, composite-prismatic, and homogeneous structures [9–11]. On the inner shell surface of bivalves, the muscle attachment area (usually named the muscle scar) is distinguished from the non-attachment area by a unique surface texture. The adductor muscle scar (AMS) is the most conspicuous area on the inner shell surface of bivalves and provides a location where the adductor muscle can be fastened strongly to the shell. Although the muscle–shell interface presents a strong connection between organic and inorganic materials, only a few studies, such as the analysis of ultrastructural features of the muscle–shell attachment in *gastropods* [12], *monoplacophorans* [13], *scaphopods* [14], *cephalopods* [15], and the bivalve *Pinctada radiata* [16] have been performed in this field. In these earlier studies, a collagenous intercellular matrix and/or specialized epithelia cells (adhesive epithelium) were believed to interweave between the muscle fibres and the shell. The myostracum is the key layer for supporting the attached muscle [10]. However, studies on this layer are few in number, and the structural details and the protein composition of this layer have not been investigated extensively, except for structural analyses of the myostracum in *Crassostrea gigas* [17] and *Mytilus galloprovincialis* [18] and proteomic analyses of the myostracum layer from *Mytilus* shell [19, 20].

As a potential organism for commercial cultivation, *Perna viridis* (also named jadeite mussel in China) is primarily distributed along the Southeast Asian coasts [21]. This mussel has attracted much attention not only for its aquaculture potential but also for its scientific potential for intertidal ecology, pollution monitoring, biofouling, and invasion biology, as reviewed by Rajagopal et.al [22]. Four species are distinguished within the genus *Perna*, including *Perna viridis*, *Perna perna*, *Perna canaliculus*, and *Perna picta*. The shell colour of *Perna* (such as green, blue-green, and brown) is a morphological characteristic for distinguishing among different *Perna* species, as reviewed by Rajagopal *et*.*al* [22]. However, the shell structure and proteome of *Perna* have not been explored thus far, and genomic data of the *Perna* genus are still unknown. In the present study, the structural characteristics of the shell from adult *P*. *viridis* were studied by scanning electron microscopy (SEM), X-ray diffraction (XRD), and Fourier transform infrared spectroscopy (FTIR). Two shell layers, nacre and myostracum, with different morphologies were identified from the *P*. *viridis* shell. Both the nacre layer and the myostracum layer are of aragonite type. Furthermore, the transcriptome of the *P*. *viridis* mantle tissue was sequenced, and a total of 69,859 unigenes were assembled. In addition, a parallel proteomics analysis for the two shell layers was performed, and a total of 378 proteins were identified from the *P*. *viridis* shell.

## Materials and methods

### Ethics statement

All procedures were in accordance with the guidelines of the Regulations for the Administration of Laboratory Animals (Decree No. 2 of the State Science and Technology Commission of the People's Republic of China, November 14, 1988) and were approved by the Institutional Animal Care and Use Committee of Zhejiang Ocean University.

### Sample preparation

*P*. *viridis* specimens used in this study were collected in Zhanjiang city of Guangdong Province, China (21°6′7″N, 110°24′42″E). The mussel was cut open using a razor blade, and the soft parts of the body were removed. The fresh shells were then collected, lightly scrubbed, and dried at room temperature.

**Fig 1:**
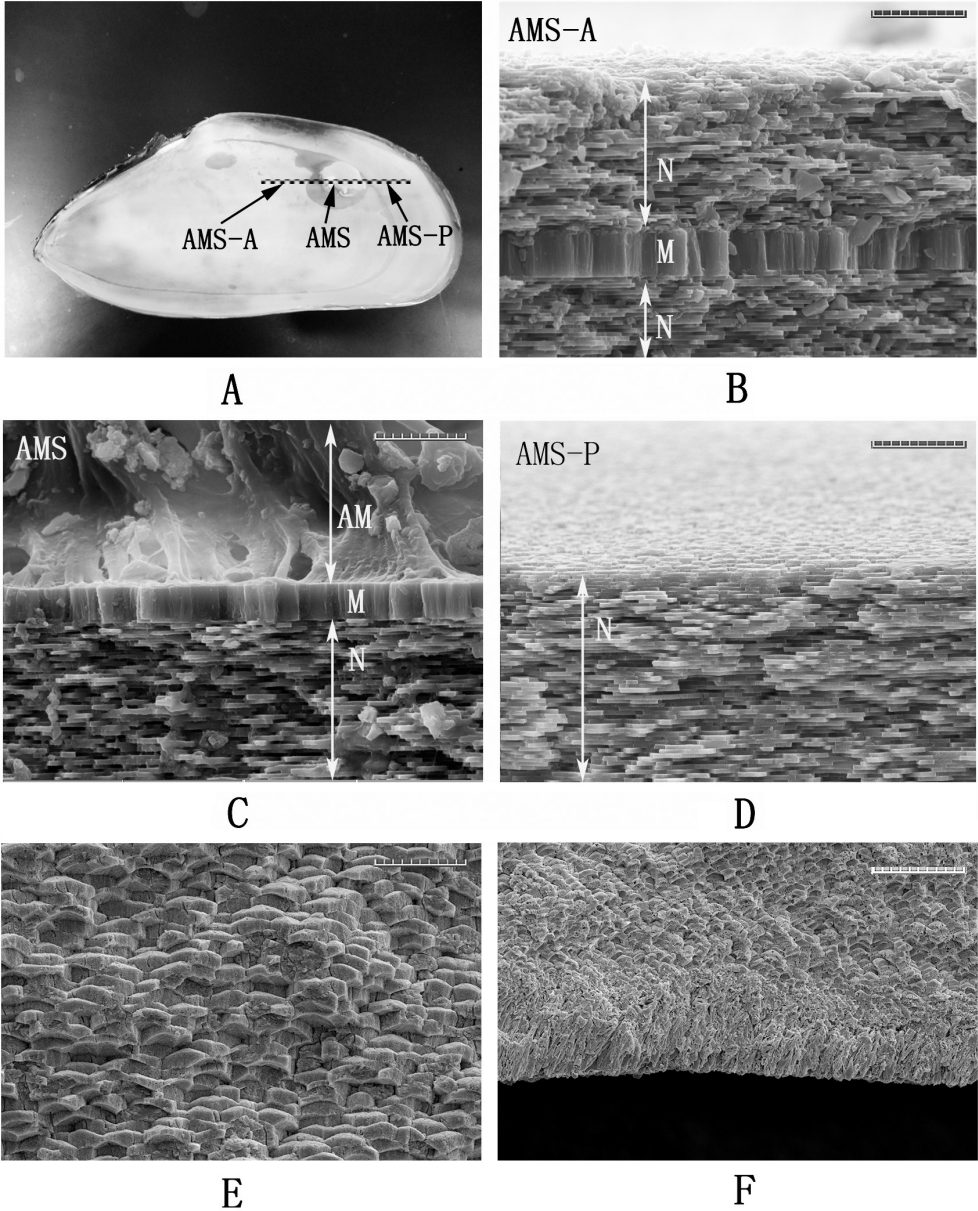
The photograph of inner surface and the SEM images of cross-section of *P*. *viridis* shell. **A**: the photograph of inner surface of an adult *P*. *viridis* shell. The black dot line represents the cutting plane; “AMS-A”, “AMS”, and “AMS-P” represent the area of anterior side from adductor muscle scar (AMS), central AMS, and the posterior side from AMS, respectively. B: the SEM image of the section of AMS-A. N represents the nacre layer, M represents the myostracum layer; **C**: the SEM image of the section of central AMS; **D**: the SEM image of the section of AMS-P. **E**: the SEM image of the section close to the outside of the shell; **F**: the SEM image of the section of the outside of the shell. The bar is 10 μm for B, C, D, and E, and 20 μm for F.

We divided the shell sample of *P*. *viridis* into two parts: the adductor muscle attachment area, corresponding to the AMS (adductor muscle scar), and the non-muscle attachment area. The latter was subdivided into AMS-A (located at the anterior side of the AMS) that corresponded to the whitish area that composed the majority of the inner shell surface, and AMS-P (located at the posterior side of the AMS) that was located on the margin outside of the pallial line (Fig 1A). Using a knife, the shell was finely separated along the cutting line, as denoted in Fig 1A. Fresh fractured shell samples were cleaned using MilliQ water. The attached adductor muscle was removed by soaking the shell sample in 5% NaOH solution, lightly scrubbing it, and drying at room temperature. The deproteinization process was carried out by soaking the samples in 20% NaOH solution at 65°C for 1 h. The samples were then rinsed in MilliQ water and freeze-dried before use.

Shell samples (polished by abrasive paper) were treated with enzymes (collagenase and chitinase) to analyse the composition and distribution of organic materials in the shell. For collagenase digestion, two collagenases, type-I (No. c0130, Sigma-Aldrich) and type-II (No. c6885, Sigma-Aldrich), were used. The shell samples were submerged in the buffer (50 mM Tricine with 10 mM calcium chloride and 400 mM sodium chloride, pH 7.5) containing 1 mg/mL collagenase (type-I or type-II) for 24 h at 25°C. For chitinase degradation, two chitinases were used including the chitinase from *Streptomyces griseus* (No. c6137, Sigma-Aldrich) and the Chitotriosidase (No. SAE0052, Sigma-Aldrich). The shell samples were submerged in 50 mM potassium phosphate buffer (pH 7.0) containing 1 mg/mL enzyme for 4 h at 37°C. The control samples were prepared by soaking with the same buffer without enzymes.

### SEM, XRD, and FTIR analysis

The prepared samples were sputter-coated with gold and examined with a VEGA-3 TCSCANER SEM at 10 kV accelerator voltage. Shell layers were identified by the sharp contact between two types of shell microstructure and by the change in mineralogy. Layers were described according to their position within the shell.

Fractured samples from the AMS and AMS-A were collected, and the inner surfaces of these samples were analysed *in situ* by XRD using a RIGAKU Ultima IV XRD system with power of 40 kV and 44 mA. The scanning speed was 2 theta/min.

Powdered samples were collected using a scalpel from the inner surface of AMS-A and AMS, corresponding to the nacre and the myostracum, respectively. The infrared spectra of powdered samples were recorded using a Fourier Transform Infrared (FTIR) spectrometer with a resolution of 4/cm on a Nicolet iS50 spectrophotometer (Thermo Scientific). The system was purged with dry N_2_ to reduce interfering water vapor IR absorption, and no water contribution was verified by measuring KBr pellets.

### RNA preparation and high-throughput sequencing of *P*. *viridis* mantle

Total RNA was extracted from the mantle tissues of *P*. *viridis* (n = 10) using TRIzol reagent (Invitrogen) according to the manufacturer’s instructions. The integrity and purity of the extracted RNA were assessed using an Agilent 2100 Bioanalyzer (Agilent RNA 6000 Nano Kit) and agarose gel electrophoresis. Further, the RNA was quantified using a NanoDrop2000 spectrophotometer (NanoDrop Technologies Inc).

The mRNA was isolated and fragmented into ~200 nt. cDNA was generated using a TruSeq TM RNA sample prep kit (Illumina) and purified with AMpure XP Beads (AGENCOURT). Then, the purified cDNA was end-repaired, ligated with an adapter, and enriched. After quantification and qualification, the cDNA library was amplified on cBot (Truseq PE Cluster Kit v3-cBot-HS, Illumina) to generate the clusters on the flowcell, and the amplified flowcell was paired-end sequenced on a HiSeq 4000 System (TruSeq SBS Kit v3-HS, Illumina).

### Unigene assembly and functional annotations

The sequence analysis and read assembly were performed by the BGI Company (www.bgitechsolutions.com) followed by the standard commercial pipeline. The reads were assembled into clusters using Trinity (trinityrnaseq-r2013-02-25) by three steps, Inchworm, Chrysalis, and Butterfly, which had been successfully used for full-length transcriptome assembly of the RNA sequencing data from the species without reference genome [23]. The contigs that resulted from the assembly of multiple sequences were referred to as transcripts. The transcripts were further divided into two classes, the unigene and the cluster (named with a prefix “CL”), containing several contigs showing more than 70% similarity. These data were archived in the NCBI Sequence Read Archive (SRA) under the accession No. SRP144773. The transcripts were functionally annotated against the NT, NR, GO, KOG, KEGG, and SwissProt databases using the BLASTX algorithm with a specific cut-off E-value < le-05.

### Proteomic analysis of *P*. *viridis* shell

The powdered shell samples from the nacre and myostracum layers of the *P*. *viridis* (n = 120) shell were collected and pooled and then suspended in cold acetic acid solution (5%, *v/v*) for 12 h at 4°C with continuous stirring. The suspension was then centrifuged at 12,000 ×g and 4°C for 15 min. The supernatant comprising the acid-soluble matrix was filtered (0.22 μm) and dialyzed (MWCO: 1 kDa) against MilliQ water before it was freeze-dried and weighed. The acid-insoluble matrix was rinsed 6 times with MilliQ water and further freeze-dried for use.

Prior to MS analysis, the protein samples of the *P*. *viridis* shells were reduced with 10 mM dithiothreitol (DTT) in 50 mM NH_4_HCO_3_ at 57°C for 1 h and alkylated using 20 mM iodoacetamide (IAA) in 50 mM NH_4_HCO_3_ for 45 min at room temperature in the dark. The excess reagent was removed by dialyzing (MWCO: 1 kDa) against water overnight. After lyophilization, the sample was treated with trypsin for 12 h at 37°C. The digests were then lyophilized and re-suspended in 0.1% formic acid and 2% acetonitrile for LC-MS/MS analysis. The LC-MS/MS analysis was performed using a Q-Exactive spectrometer (Thermo Fisher Scientific, San Jose, CA) interfaced with a LC-20AD HPLC system (Shimadzu, Japan). The trypsin-digested protein sample mixture was separated on a C18 column (75 μm × 150 mm, 3.6 μ). The HPLC gradient was 8~35% buffer B (98% ACN, 0.1% formic acid) in buffer A (2% ACN, 0.1% formic acid) at a flow rate of 300 nL/min over 37 min, followed by 35–60% buffer B over 5 min.

Isolated peptides from HPLC were submitted to an ion-trap mass spectrometer with data-dependent acquisition (DDA) model detection under an ion spray voltage of 1.6 kV. The MS data were acquired automatically, following an MS survey scan over m/z 350~1600 m/z at a resolution of 70,000 for full scan and 17,500 for MS/MS measurements. The MS/MS spectra were sequentially and dynamically acquired with a dynamic exclusion duration of 15 s for the 20 most intense peptides with intensities greater than 10,000 and positive charges from 2^+^ to 7^+^. The raw MS/MS data were converted into MGF format for bioinformatics analysis by Proteome Discoverer (Thermo Fisher Scientific Inc., MA, USA).

Protein identification was performed using Mascot database-searching software (version 2.3, Matrix Science, London, UK) against the mantle transcriptome database of *P*. *viridis* generated by Illumina sequencing. Carbamidomethyl was set as a fixed modification. Oxidation, Gln->pyro-Glu, and Deamidated were set as variable modifications. The peptide mass tolerance was set to 20 ppm, and the fragment mass tolerance was set to 0.05 Da, respectively. False discovery rate (FDR) analysis was performed, and an FDR<0.01 was considered for protein identification by using a target-decoy search strategy, a methodology for distinguishing correct from incorrect peptide identifications [24].

For all of the identified proteins, functional annotations were performed using the Blast2GO programme against the databases of Gene Ontology (GO), Kyoto Encyclopedia of Genes and Genomes (KEGG), and Cluster of Orthologous Groups of proteins (COG). The homologous sequence searching of identified proteins was performed against the Non-redundant protein sequences (NR) at NCBI. The signal peptides were predicted using the SignalP 4.1 online tool (http://www.cbs.dtu.dk/services/SignalP/), and conserved domains were predicted using the SMART online tool at http://smart.embl-heidelberg.de/. The amino acid composition was analysed using the ProtParam tool at http://web.expasy.org/protparam/.

## Results

### The microstructure of the *P*. *viridis* shell

As shown in Fig 1A, on the inner surface of adult *P*. *viridis* shells, two distinct areas with different colours and texture can be observed optically, including the whitish area accounting for majority of the inner surface (represented by AMS-A and AMS-P) and the AMS with muscle attached. When the adductor muscle was removed, the AMS of *P*. *viridis* presented a large, shiny area of light colour located towards the posterodorsal edge of each valve and merged with the scar of protractor muscle and retractor muscle (Fig 1A). The SEM images of sections perpendicular to the shell surfaces of the AMS, AMS-A, and AMS-P are shown in Fig 1B–1D, respectively. We describe the shell microstructures using the terminology (such as nacre and myostracum) defined by Carter [10] and Luciana et al. [25], and the relative position of these layers within the shell (Fig 1). In this study, the two microstructures observed in the *P*. *viridis* shell, the nacre and the myostracum layers, have different thicknesses and morphologies. At the AMS-A, the myostracum layer is buried between two nacreous laths, the exterior nacre and the interior nacre layer. At the AMS, the exterior nacre layer disappeared and the myostracum layer, with a thickness of approximately 5 μm, is attached by the adductor muscles (Fig 1C). The myostracum layer is elongated from the shell umbo to the scar and finally disappears at the AMS-P (Fig 1D). At the outside of the shell, we found that the tablets of the nacre layer become thicker and a prismatic-like layer can be seen at the outermost layer of the shell (Fig 1E and 1F).

**Fig 2:**
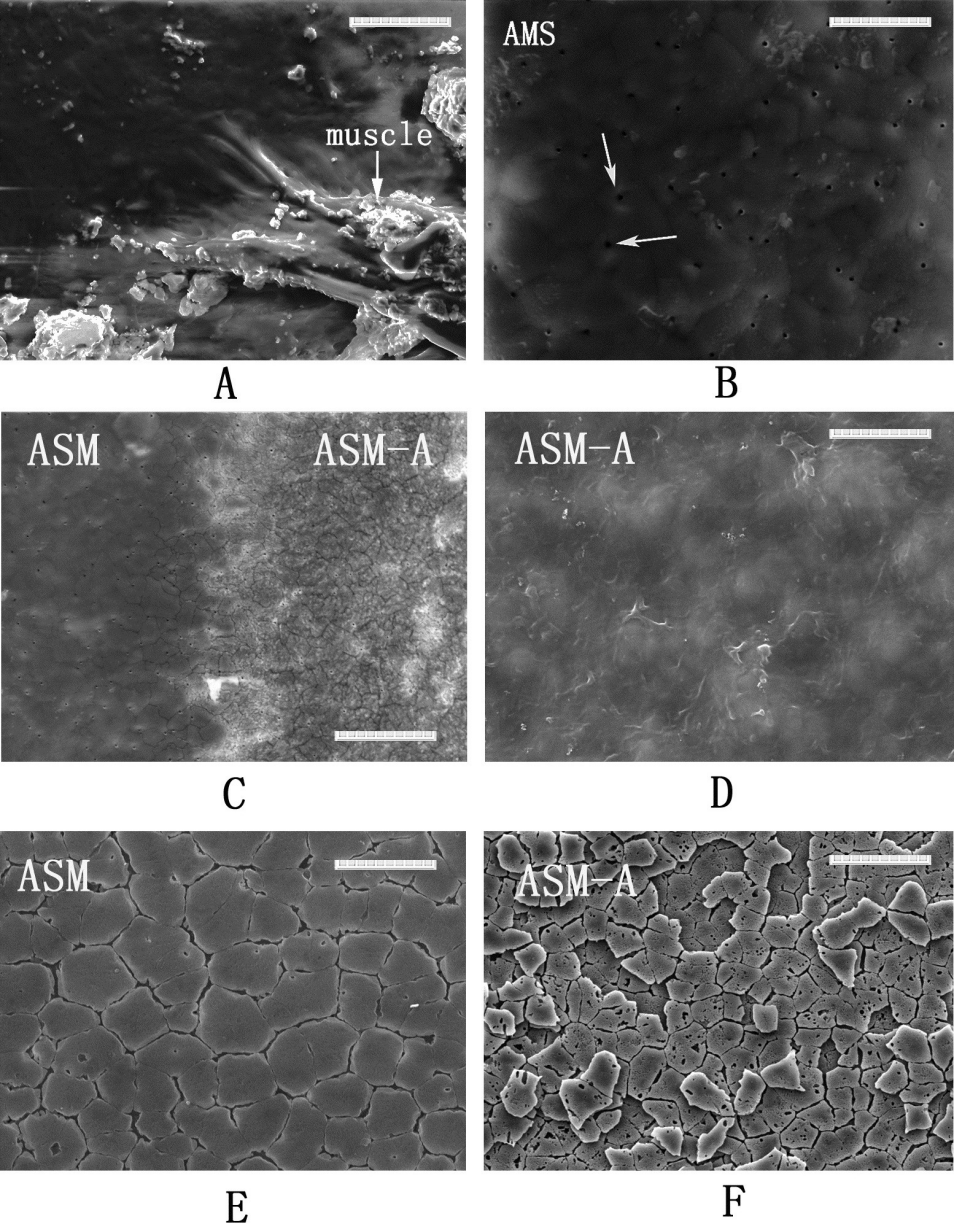
Surface images of AMS and AMS-A region. **A**: the inner surface image of natural shell of *P*. *viridis* at AMS region with the adductor muscle attached; **B**: the SEM image of surface of AMS region after the adductor muscle removed. The pit structures are denoted by arrows; **C**: the SEM image of the surface from transition zone between the AMS and AMS-A; **D**: the SEM image of the surface of AMS-A; **E**: The SEM image of the AMS surface after deproteinization; **F**: The SEM image of the AMS-A surface after deproteinization.

Fig 2 shows the SEM images of the inner shell surface. The surface of muscle-attached AMS and non-attachment areas were selected for analysis. As shown in Fig 2A, the muscle fibres are tightly attached to the AMS. A film with many nanoscale pit structures was observed on the AMS when the muscle was detached using 5% NaOH (Fig 2B). These pit structures are also present at the transition areas of the AMS edge (Fig 2C). However, for the non-attachment area, represented by AMS-A in this study, a translucent film without pit structures was observed (Fig 2D), exhibiting a different appearance from the AMS area (Fig 2B). On the surface of the AMS-A, some distinct polygonal shapes, corresponding to the underlying tablets of the nacre layer, were seen under the film (Fig 2D). However, the film in the AMS area was too thick to easily distinguish the shape of the underlying calcium carbonate crystals (Fig 2B). After deproteinization with 20% NaOH, the film covering the AMS was removed, and mosaic tiles with some holes and cracks were observed (Fig 2E). For the AMS-A, the tablet surface was visible after the film was removed (Fig 2F).

### The polymorph character of shell layers

**Fig 3:**
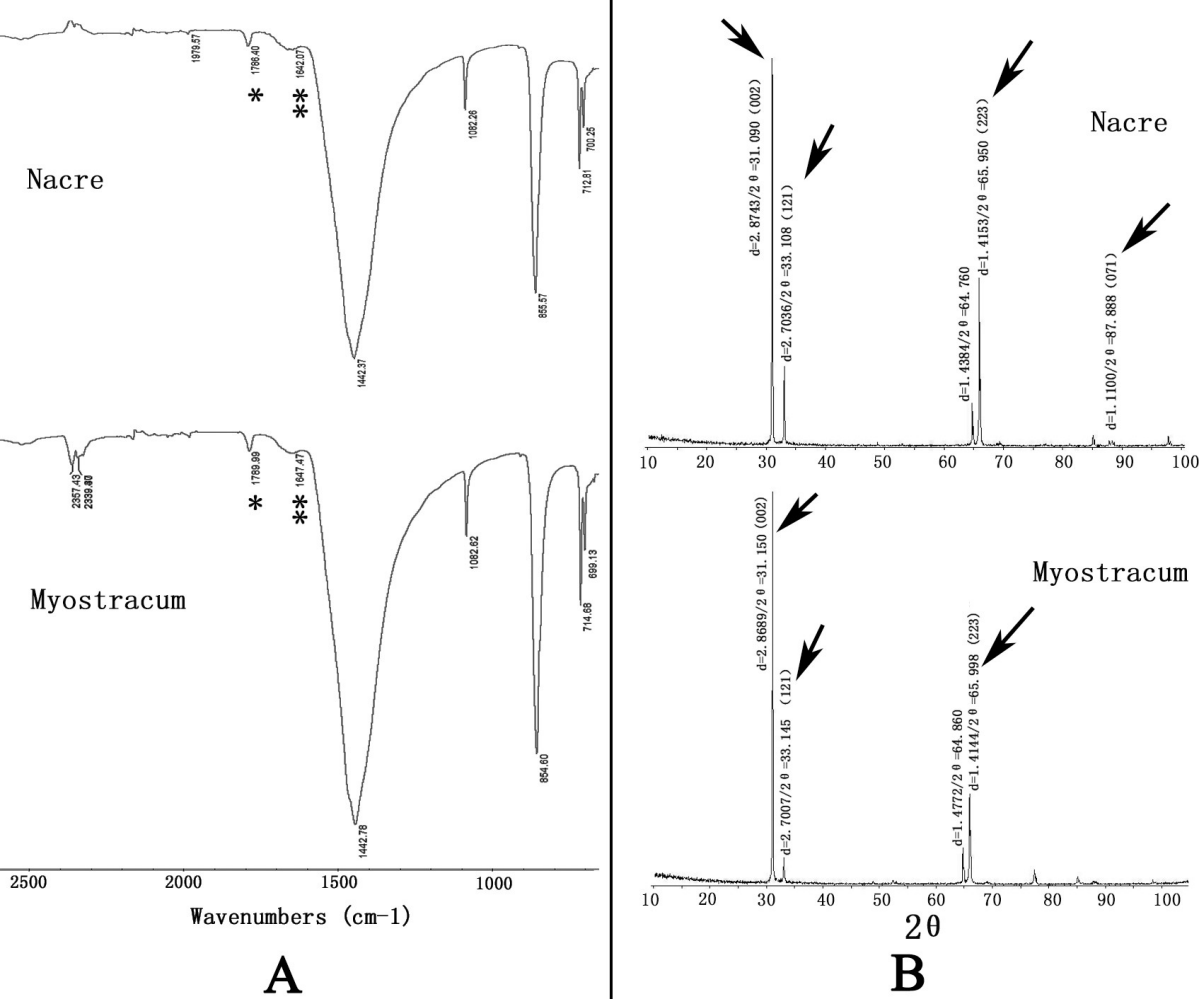
FTIR spectra and XRD profiles of the nacre layer and myostracum layer. **A:** FTIR spectra of the two layers of *P*. *viridis* shell. The single star and the double star indicate the amide I and amide II region, respectively. **B:** XRD profiles of the two layers of *P*. *viridis* shell. The arrows represent the aragonite peaks.

Fig 3 shows the FTIR and XRD spectra of the two layers from the *P*. *viridis* shell. The nacre and the myostracum layers showed similar FTIR spectra with four characteristic bands (1082–1085/cm, 854–857/cm, 714/cm, and 699/cm), indicating that the internal vibration modes of CO_3_^2−^ ions were detected (Fig 3A), which represents the characteristics of the aragonite structure. Meanwhile, an amide I feature (located in 1647–1650/cm, denoted by the double star in Fig 3A) and C = O feature (1784–1789/cm, denoted by the single star in Fig 3A) were also detected in both nacre and myostracum layers, indicating the presence of a small amount of organic matrixes in these two layers. The polymorphs of the nacre and the myostracum layer were further determined as aragonite according to the XRD profiles (Fig 3B).

### Organic matrix distribution

**Fig 4:**
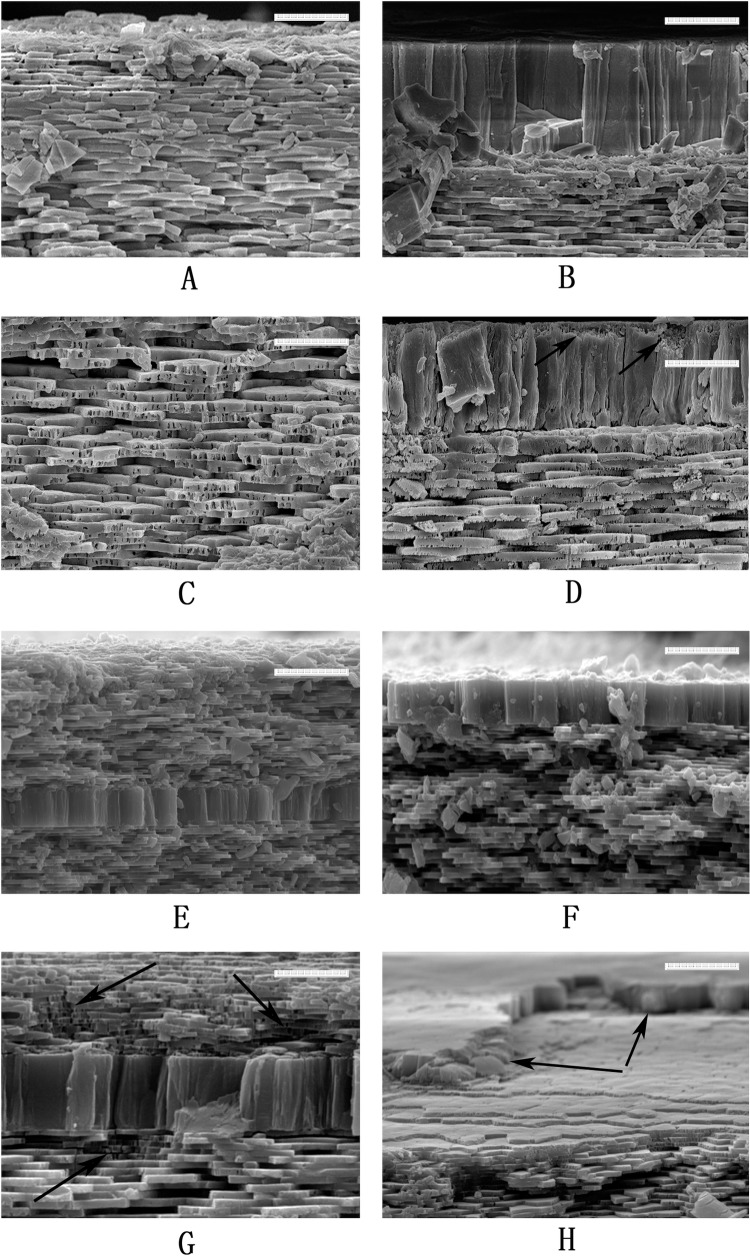
SEM images of shell cross-section at AMS-A and AMS of *P*. *viridis* after collagenases digestion. **A**: the section image of AMS-A from the control sample; **B**: the section image of AMS from the control sample; **C:** the SEM image of AMS–A section after type-I collagenase digestion; the etched cracks and holes can be seen at the tablet of the nacre layer; **D**: the SEM image of AMS section after type-I collagenase digestion; the etched area at the myostracum layer are denoted by black arrows; **E**: the section image of AMS-A from the control sample; **F**: the section image of AMS from the control sample; **G**: the SEM image of AMS–A section after type-II collagenase digestion; the etched area at the nacre layer are circled with white dash line and the etched crack of the myostracum layer are denoted by black arrows; **H**: the SEM image of AMS section after type-II collagenase digestion; the etched area at the nacre layer are denoted by black arrows. The bar is 5 μm.

To identify the distribution of the organic matrix in the *P*. *viridis* shell, shell samples were digested by collagenases and chitinases, respectively. Compared to the control samples (Fig 4A and 4B), the nacre layer of AMS-A was obviously etched by type-I collagenase digestion, resulting in many cracks and holes presented in the tablets (Fig 4C); etched cracks can also be seen in the myostracum layer of AMS-A (Fig 4D). For type-II collagenase digestion, comparing with the control samples (Fig 4E and 4F), the nacre layer of AMS-A was slightly etched (Fig 4G), and at the AMS region, the integrity of the myostracum layer was damaged due to part of the myostracum layer detaching from the surface of the AMS after collagenase digestion (Fig 4H).

**Fig 5:**
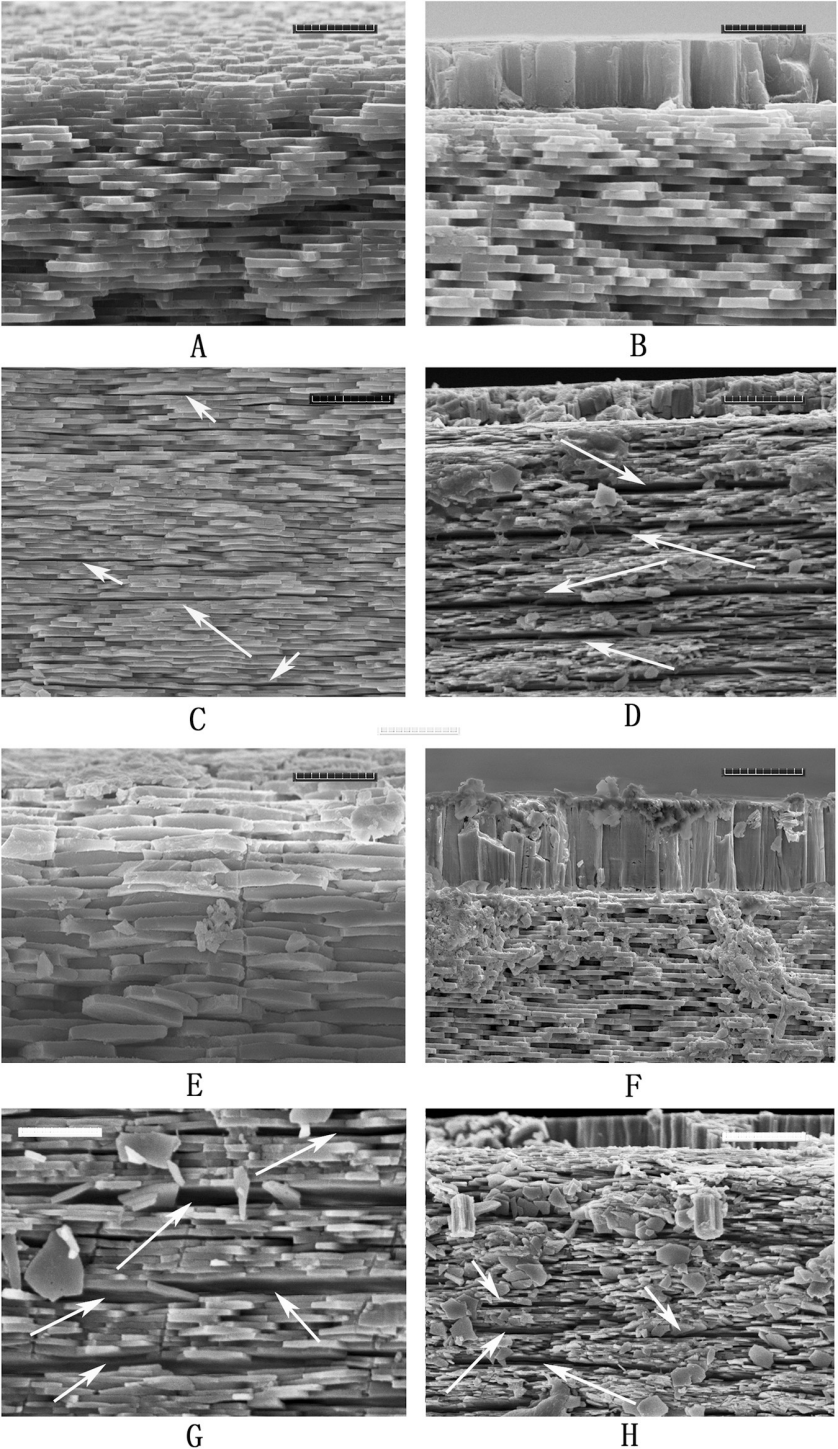
SEM images of shell cross-section at AMS-A and AMS of *P*. *viridis* after chitinases digestion. **A**: the section image of AMS-A from the control sample; **B**: the section image of AMS from the control sample; **C:** the SEM image of AMS–A section after chitinase digestion; the etched long cracks at the nacre layer are denoted by arrows; **D**: the SEM image of AMS section after chitinase digestion; the etched long cracks at the nacre layer are denoted by arrows. **E**: the section image of AMS-A from the control sample; **F**: the section image of AMS from the control sample; **G**: the SEM image of AMS–A section after chitinase digestion; the etched long cracks at the nacre layer are denoted by arrows; **H**: the SEM image of AMS section after chitinase digestion; the etched long cracks at the nacre layer are denoted by arrows. The bar is 5 μm for A, B, E, F and G, and 10 μm for C, D, and H, respectively.

To test the distribution of chitin in different layers of *P*. *viridis* shell, AMS-A and AMS were treated with chitinase and chitotriosidase respectively. Both chitinase and chitotriosidase resulted in a similar effects for the shell samples. Compared to the control samples (Fig 5A and 5B), the microstructure of AMS-A after chitinase digestion showed some long cracks at the interlamellar of the nacre layer (Fig 5C). For the AMS region, long cracks are present at the interior nacre layer and the myostracum-nacre interface (Fig 5D). For chitotriosidase digestion, similar results were presented comparing to the control samples (Fig 5E and 5F). Long cracks can be seen at the nacre layer of the AMS-A and AMS region after chitotriosidase digestion (Fig 5G and 5H).

**Fig 6:**
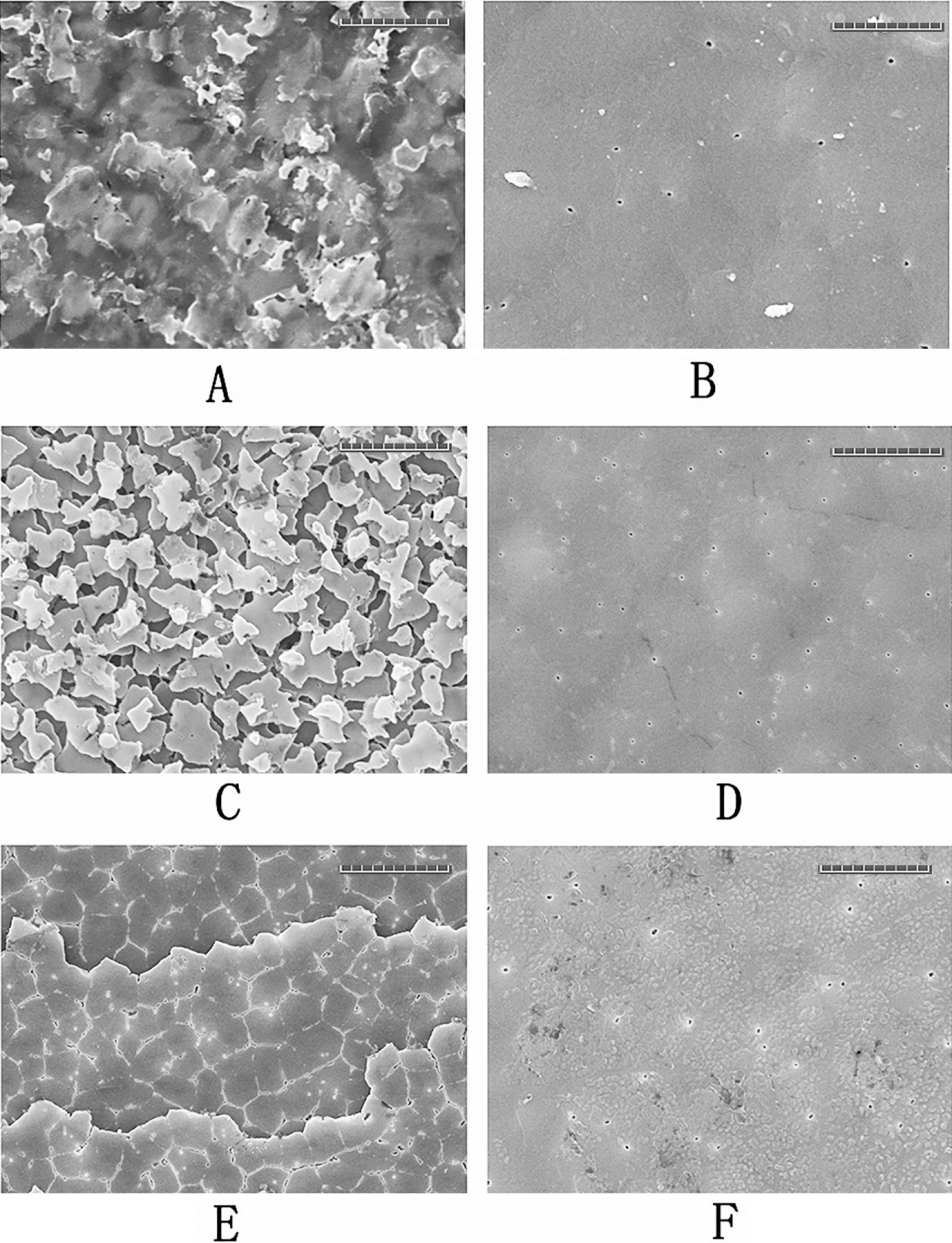
SEM images of inner surface at the AMS-A and the AMS area after enzyme digestion. **A**: the surface image of AMS-A from control sample; **B**: the surface image of AMS from control sample; **C:** the SEM image of AMS–A surface after collagenase digestion; **D**: the SEM image of AMS surface after collagenase digestion; **E:** the SEM image of AMS–A surface after chitinase digestion; **F**: the SEM image of AMS surface after chitinase digestion.

For the surface of AMS and AMS-A samples after enzyme digestion, we noticed that, compared to the control samples (Figs 6A and 5B), the film covering the AMS-A region was digested by collagenase, and the underlying tablets of the nacre layer were presented individually (Fig 6C). In addition, nacreous tablets on the surface of AMS-A showed a terrace pattern after chitinase digestion (Fig 6E). Interestingly, the film covering the AMS region showed strong resistance to the two enzymes, and no significant morphological changes were observed in this study (Figs 6D and 5F).

### Transcriptome sequencing, assembly, and annotation

The mantle is the main organ that secretes shell proteins. For *P*. *viridis*, the RNA from 10 mantle samples was sequenced to yield a total of 83.28 Mb raw reads using an Illumina Hiseq4000 system. After further quality filtration, a total of 59.52 Mb of clean reads were obtained and used for subsequent assembly and annotation. De novo assembly of the clean reads yielded a total of 69,859 unigenes with an average length of 690 nt and an N50 of 1,093 nt (S1 Fig). The transcriptomic data of *P*. *viridis* mantle were submitted to the SRA database under No. SRP144773.

**Fig 7:**
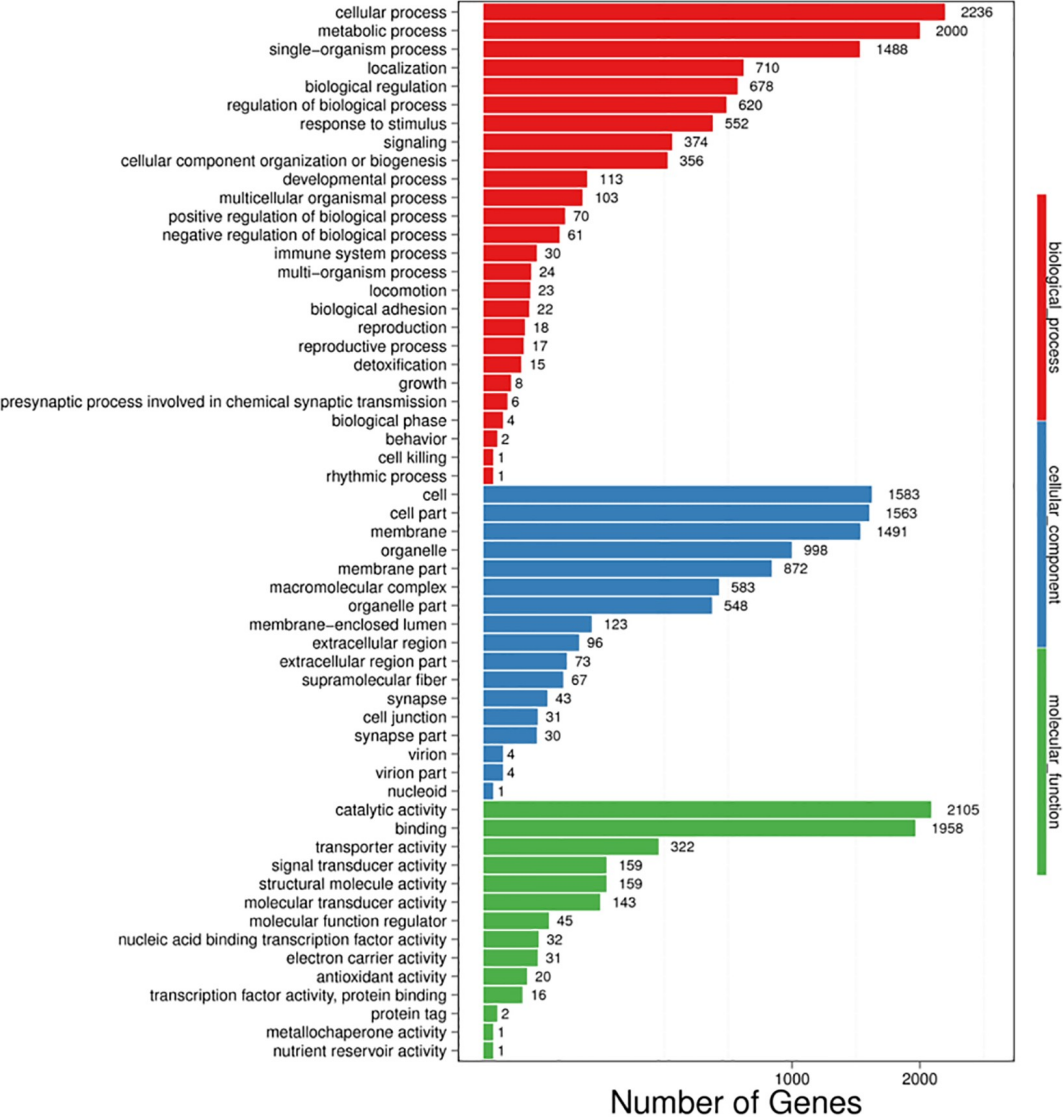
Gene Ontology annotation of the unigenes from *P*. *viridis* mantle transcriptome.

**Fig 8:**
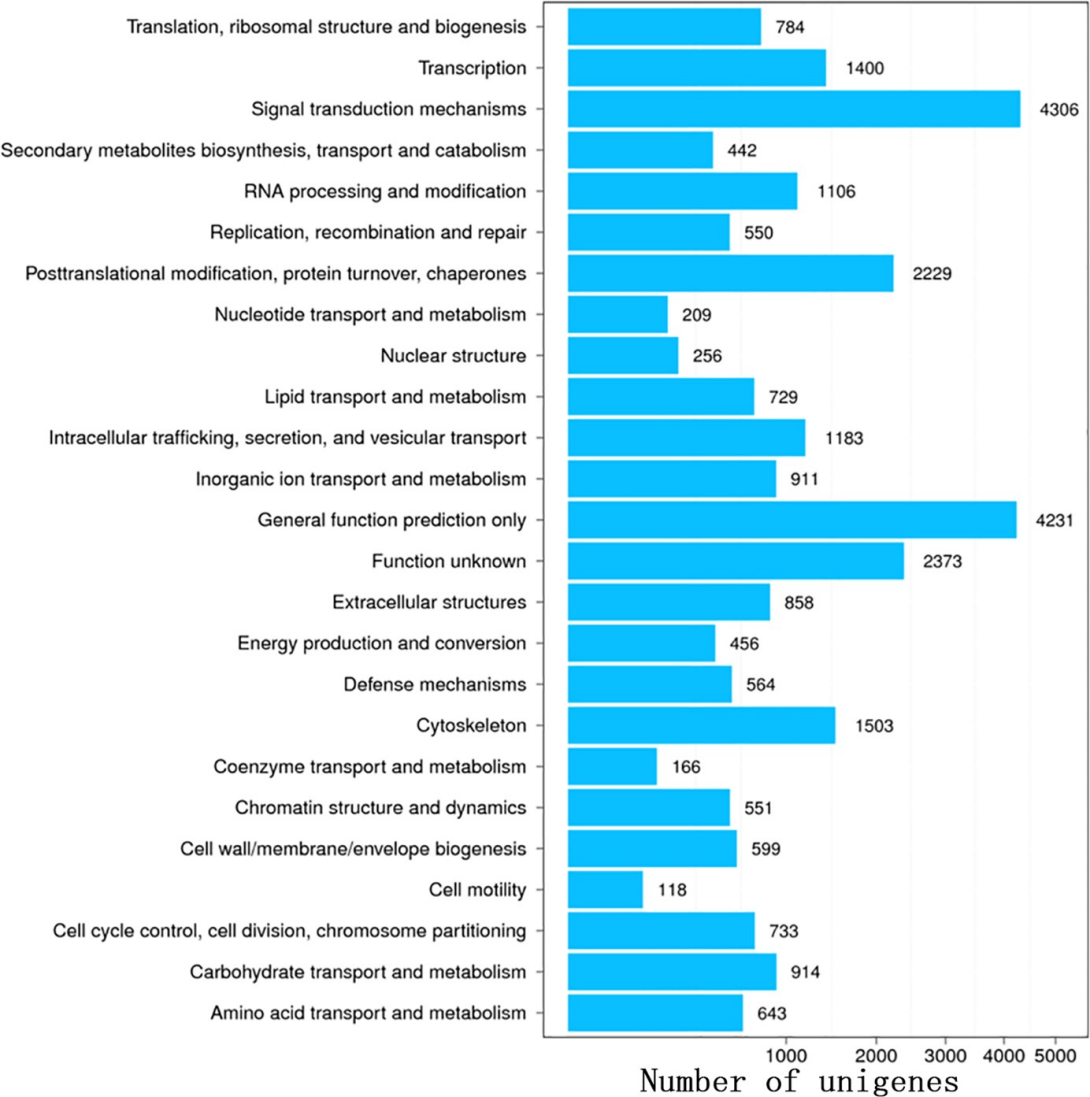
KOG annotation of the unigenes from *P*. *viridis* mantle transcriptome.

**Fig 9:**
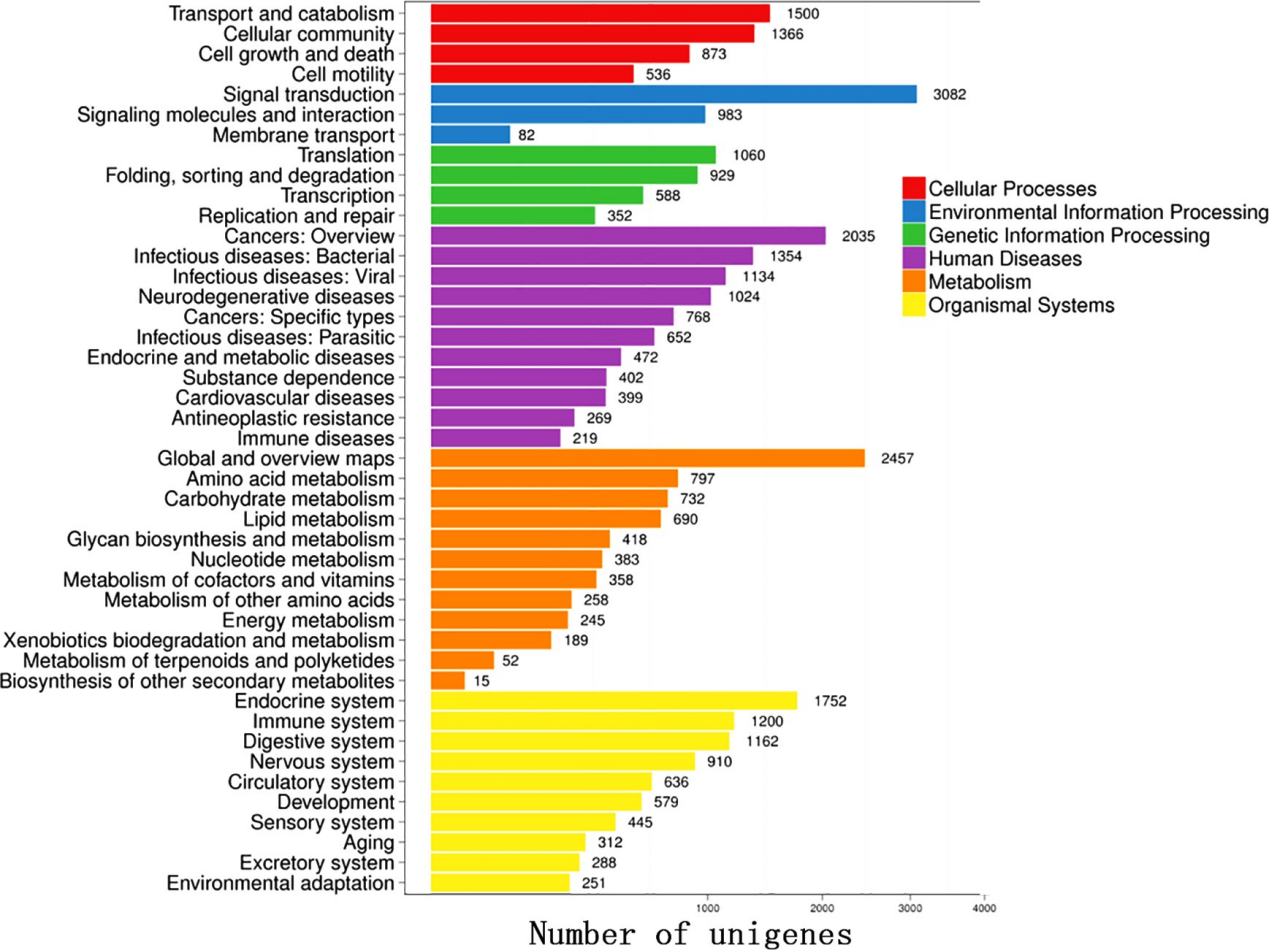
COG annotation of the unigenes from *P*. *viridis* mantle transcriptome.

The functional annotation of 69,859 unigenes revealed 26,852 (NR, 38.44%), 6,325 (NT, 9.05%), 19,395 (SwissProt, 27.76%), 17,972 (KOG, 25.73%), 19,682 (KEGG, 28.17%), 4,832 (GO, 6.92%), and 20,870 (InterPro, 29.87%) unique annotations with an E-value cut-off of 1E-05 (S2 Fig). In addition, 24,021 CDSs were detected by Transdecoder, and 6,084 SSRs were found in 5,337 unigenes. Out of the 26,852 significant matches found in the NR database, 16,003 (59.60%) unigenes were from *Crassostrea gigas*. Other matches were from *Lottia gigantea* (1,544, 5.75%), *Lingula anatina* (1,099, 4.09%), *Aplysia californica* (864, 3.22%), *Octopus bimaculoides* (730, 2.72%), and *Biomphalaria glabrata* (519, 1.93%) (S3 Fig). A total of 4,832 unigenes were assigned to GO categories (Fig 7). The majority of genes in the Biological processes annotation shared genes associated with the terms Cellular process, Metabolic process, and Single-organism process. The Cellular component annotation included genes associated with the terms Cell, Cell part, and Membrane. The Molecular function annotation included a dominance of genes associated with Catalytic activity, Binding, and Transporter activity. A total of 17,972 unigenes were mapped to the KOG database, and the majority of KOG functions shared genes with Signal transduction mechanisms (4,306), General function prediction only (4,231), Function unknown (2,373), Posttranslational modification, protein turnover, chaperones (2,229), Cytoskeleton (1,503), Transcription (1,400), and Intracellular trafficking, secretion, and vesicular transport (1,183) (Fig 8). According to the KEGG annotation, the matched 19,682 unigenes were divided into 6 categories, including Cellular Processes, Environmental Information Processing, Genetic Information Processing, Human Diseases, Metabolism, and Organismal Systems. In KEGG classification, Signal transduction (3,082) encompassed most of the KEGG classification, followed by Global and overview maps (2,457), Cancers overview (2,035), Endocrine system (1,752), Transport and catabolism (1,500), and Cellular community (1,366) (Fig 9).

### Identification of the transcripts encoding biomineralization- related proteins

To obtain an integrated view of the transcriptional events of biomineralization-related processes in the *P*. *viridis* mantle, the unique transcript library was screened for reported SMPs. Taken together, 111 transcripts were identified with significant hits (E-value<1E-05) to 14 reported biomineralization-related protein families, including SMPs from *Pinctada*, *Crassostrea*, *Mytilus*, and *Lingula* (Table 1). Of the 113 matched transcripts, the most abundant ESTs were annotated as Perlucin family (32 transcripts), followed by Carbonic Anhydrase (18 transcripts), Calponin (16 transcripts) and Tyrosinase (9 transcripts). However, some known SMPs, such as Nacrein, Prisilkin, and Prismalin, were not found in the *P*. *viridis* mantle transcriptome.

**Table 1:** The candidate mantle transcripts involved in biomineralization from *P*. *viridis* mantle.

SMPs	Unigene ID	Length(nt)	FPKM	Homology ID	E-value	Annotation	Species
***Shell Matrix Protein***	CL1751.Contig1	950	1382.52	gi|358640269|	1.00E-26	shell matrix protein	*Pinctada maxima*
	Unigene13969	768	421.83	gi|358640269|	5.40E-34	shell matrix protein	*Pinctada maxima*
	CL3334.Contig1	1034	0.77	gi|37991666|	2.50E-10	shell matrix protein	*Pinctada fucata*
	CL3334.Contig2	999	2.57	gi|37991666|	2.40E-10	shell matrix protein	*Pinctada fucata*
***Matrix protein***	CL1058.Contig2	680	4.91	gi|762093630|	7.70E-24	insoluble matrix shell protein 5	*Crassostrea gigas*
	Unigene51584	2135	3.75	gi|906541679|	2.70E-83	matrix protein-1	*Mytilus coruscus*
	Unigene58359	252	1.98	gi|906541679|	2.10E-11	matrix protein-1	*Mytilus coruscus*
	Unigene18850	315	1.08	gi|906541679|	2.00E-06	matrix protein-1	*Mytilus coruscus*
***BMSP***	Unigene13592	341	1.3	gi|347800228|	2.50E-39	BMSP	*Mytilus galloprovincialis*
	Unigene49574	1189	394.35	gi|347800228|	3.10E-20	BMSP	*Mytilus galloprovincialis*
	Unigene13592	341	1.3	gi|347800228|	2.50E-39	BMSP	*Mytilus galloprovincialis*
***Shell Protein***	CL1834.Contig5	1221	508.08	gi|824631220|	8.50E-90	shell protein-3	*Mytilus coruscus*
	CL1834.Contig4	1203	345.49	gi|824631220|	8.40E-90	shell protein-3	*Mytilus coruscus*
	Unigene48620	1448	11.22	gi|824631258|	4.20E-19	shell protein-4	*Mytilus coruscus*
	CL2107.Contig1	275	3.05	gi|824631258|	3.30E-18	shell protein-4	*Mytilus coruscus*
	Unigene14698	225	2.36	gi|824631258|	1.60E-07	shell protein-4	*Mytilus coruscus*
	CL2107.Contig2	212	0.65	gi|824631258|	8.80E-11	shell protein-4	*Mytilus coruscus*
	Unigene37596	360	0.6	gi|824631258|	2.40E-24	shell protein-4	*Mytilus coruscus*
	Unigene49392	229	0.57	gi|824631258|	6.40E-07	shell protein-4	*Mytilus coruscus*
	Unigene25946	784	25.32	gi|824631302|	7.50E-39	shell protein-5	*Mytilus coruscus*
	Unigene39912	2396	20.16	gi|824631368|	3.10E-293	shell protein-6	*Mytilus coruscus*
	Unigene39912	2396	20.16	gi|824631368|	3.10E-293	shell protein-6	*Mytilus coruscus*
***Shematrin***	Unigene25272	570	7066.74	sp|P86950|	2.20E-18	Shematrin-like protein 2	*Pinctada maxima*
	CL906.Contig2	237	529.04	sp|P86950|	1.30E-13	Shematrin-like protein 2	*Pinctada maxima*
	Unigene35681	357	483.05	sp|P86950|	3.30E-10	Shematrin-like protein 2	*Pinctada maxima*
***Perlwapin***	Unigene6222	470	69.04	gi|322966963|	3.10E-16	Perlwapin-like protein	*Mytilus galloprovincialis*
	Unigene49895	469	12.37	gi|322966963|	1.70E-14	Perlwapin-like protein	*Mytilus galloprovincialis*
	Unigene22852	473	3.12	gi|322966963|	6.50E-14	Perlwapin-like protein	*Mytilus galloprovincialis*
	CL1164.Contig2	299	0.94	gi|322966963|	7.00E-14	Perlwapin-like protein	*Mytilus galloprovincialis*
***Perlucin***	Unigene44001	798	1320.8	gi|919038087|	6.50E-22	perlucin-like protein	*Lingula anatina*
	CL3071.Contig1	682	35.05	gi|762162178|	1.00E-15	perlucin-like protein	*Crassostrea gigas*
	CL2964.Contig2	1027	16.67	gi|762146046|	5.00E-43	perlucin-like protein	*Crassostrea gigas*
	CL2929.Contig1	658	13.66	gi|762086100|	1.20E-18	perlucin-like protein	*Crassostrea gigas*
	Unigene48742	1020	9.11	gi|762146046|	7.00E-37	perlucin-like protein	*Crassostrea gigas*
	Unigene819	1803	6.91	gi|762119800|	9.20E-24	perlucin-like protein	*Crassostrea gigas*
	CL2056.Contig2	838	6.62	gi|919097779|	1.20E-26	perlucin-like protein	*Lingula anatina*
	CL2056.Contig1	784	6.12	gi|919097779|	1.20E-25	perlucin-like protein	*Lingula anatina*
	Unigene15067	465	5.48	gi|762134185|	1.50E-23	perlucin-like protein	*Crassostrea gigas*
	Unigene29242	804	4.73	gi|762092014|	1.10E-21	perlucin-like protein	*Crassostrea gigas*
	Unigene8405	469	3.69	gi|919038103|	2.50E-18	perlucin-like protein	*Lingula anatina*
	Unigene28554	486	3.53	gi|762095947|	1.50E-24	perlucin-like protein	*Crassostrea gigas*
	Unigene43921	727	3.51	gi|762138945|	1.90E-20	perlucin-like protein	*Crassostrea gigas*
	CL3208.Contig2	479	3.14	gi|762140856|	2.50E-29	perlucin-like protein isoform X1	*Crassostrea gigas*
	CL2964.Contig1	397	2.67	gi|762150628|	1.20E-37	perlucin-like protein	*Crassostrea gigas*
	CL787.Contig1	533	2.25	gi|762140856|	1.60E-56	perlucin-like protein isoform X1	*Crassostrea gigas*
	Unigene30113	466	1.75	gi|405974900|	4.50E-31	Perlucin	*Crassostrea gigas*
	Unigene26171	715	1.72	gi|322966877|	1.20E-19	Perlucin-like protein	*Mytilus galloprovincialis*
	Unigene11150	387	1.65	gi|762159611|	9.50E-11	perlucin-like protein	*Crassostrea gigas*
	Unigene55945	451	1.59	gi|762135368|	6.90E-13	perlucin-like protein	*Crassostrea gigas*
	CL2929.Contig2	706	1.37	gi|762086100|	1.30E-18	perlucin-like protein	*Crassostrea gigas*
	Unigene40000	333	1.34	gi|762134185|	4.00E-10	perlucin-like protein	*Crassostrea gigas*
	Unigene37476	242	1.05	gi|322966877|	3.50E-11	Perlucin-like protein	*Mytilus galloprovincialis*
	Unigene50431	432	0.96	gi|762138945|	1.50E-17	perlucin-like protein	*Crassostrea gigas*
	CL3208.Contig1	565	0.88	gi|762140856|	3.00E-29	perlucin-like protein isoform X1	*Crassostrea gigas*
	Unigene31631	374	0.86	gi|322966877|	8.80E-14	Perlucin-like protein	*Mytilus galloprovincialis*
	Unigene19021	338	0.66	gi|762095947|	5.50E-15	perlucin-like protein	*Crassostrea gigas*
	CL787.Contig2	241	0.53	gi|762140858|	1.20E-08	perlucin-like protein isoform X2	*Crassostrea gigas*
	Unigene26208	414	0.51	gi|762095947|	2.40E-20	perlucin-like protein	*Crassostrea gigas*
	Unigene33094	445	0.46	gi|762095947|	4.10E-18	perlucin-like protein	*Crassostrea gigas*
	Unigene4690	292	0.4	gi|762144436|	5.40E-27	perlucin-like isoform X1	*Crassostrea gigas*
	Unigene11302	341	0.32	gi|762156590|	4.4E-12	perlucin-like protein isoform X1	*Crassostrea gigas*
***MUSP***	Unigene39610	909	7.8	gi|322518389|	1.4E-49	MUSP-3	*Mytilus californianus*
***N66***	Unigene49391	716	1.85	gi|762167078|	1.30E-08	N66 matrix protein-like	*Crassostrea gigas*
***Lustrin***	Unigene27969	819	1.41	gi|403310241|	1.90E-29	Lustrin A	*Patella vulgata*
***Fibronectin***	Unigene19411	3686	10.13	gi|762106647|	3.10E-228	fibronectin type-III domain-containing protein 3A-like isoform X2	*Crassostrea gigas*
	CL1048.Contig6	3709	9.77	gi|405965005|	2.50E-132	Fibronectin type 3 and ankyrin repeat domains protein 1	*Crassostrea gigas*
	CL1048.Contig3	3372	5.66	gi|405965005|	1.70E-79	Fibronectin type 3 and ankyrin repeat domains protein 1	*Crassostrea gigas*
	Unigene8731	691	1.1	gi|762106659|	4.80E-13	fibronectin type-III domain-containing protein 3a-like isoform X7	*Crassostrea gigas*
***Carbonic Anhydrase***	Unigene14068	1431	28.19	gi|762105612|	4.20E-80	carbonic anhydrase 14-like isoform X2	*Crassostrea gigas*
	CL3477.Contig2	1086	21.6	gi|926623760|	1.30E-44	carbonic anhydrase 1-like	*Limulus polyphemus*
	Unigene20239	854	6.19	gi|762105618|	6.40E-92	carbonic anhydrase-like isoform X2	*Crassostrea gigas*
	CL1540.Contig1	874	5.02	gi|930420592|	8.60E-60	carbonic anhydrase II	*Mytilus galloprovincialis*
	Unigene24266	1141	3.09	gi|556721699|	4.80E-26	carbonic anhydrase, chloroplastic-like	*Pantholops hodgsonii*
	CL3477.Contig1	1011	2.73	gi|926623760|	2.60E-44	carbonic anhydrase 1-like	*Limulus polyphemus*
	CL2669.Contig2	1251	2.15	gi|762087946|	1.30E-64	carbonic anhydrase-like protein 2 isoform X2	*Crassostrea gigas*
	Unigene14464	324	2.09	gi|906541712|	3.60E-32	carbonic anhydrase-like protein, partial	*Mytilus coruscus*
	Unigene11124	1076	2.02	gi|762143564|	3.40E-58	carbonic anhydrase 1-like	*Crassostrea gigas*
	Unigene28187	307	1.87	gi|762087944|	3.20E-09	carbonic anhydrase-like protein 2 isoform X1	*Crassostrea gigas*
	Unigene10741	575	1.7	gi|906541712|	9.30E-39	carbonic anhydrase-like protein, partial	*Mytilus coruscus*
	CL2669.Contig1	1132	1.59	gi|762087944|	4.00E-49	carbonic anhydrase-like protein 2 isoform X1	*Crassostrea gigas*
	Unigene33816	458	1.56	gi|930420592|	2.40E-53	carbonic anhydrase II	*Mytilus galloprovincialis*
	Unigene42754	229	1.15	gi|762079780|	1.30E-07	carbonic anhydrase-related protein-like isoform X1	*Crassostrea gigas*
	Unigene35161	1125	1.12	gi|762158559|	3.00E-81	carbonic anhydrase-related protein 10-like	*Crassostrea gigas*
	CL1540.Contig2	479	0.96	gi|930420592|	1.20E-07	carbonic anhydrase II	*Mytilus galloprovincialis*
	Unigene38982	216	0.63	gi|762105616|	2.90E-17	carbonic anhydrase-like isoform X1	*Crassostrea gigas*
	Unigene28176	266	0.46	gi|906541712|	1.60E-14	carbonic anhydrase-like protein	*Mytilus coruscus*
***calponin***	Unigene20161	1988	618.84	gi|906541670|	4.20E-203	calponin-like protein-1	*Mytilus coruscus*
	Unigene20162	1146	274.46	gi|906541757|	1.30E-95	calponin-like protein-2	*Mytilus coruscus*
	CL919.Contig3	6425	6.65	gi|762128026|	4.50E-166	calponin homology domain-containing protein	*Crassostrea gigas*
	Unigene5471	2710	4.62	gi|762080984|	1.00E-71	calponin homology domain-containing protein isoform X3	*Crassostrea gigas*
	Unigene35980	548	4.14	gi|642928295|	6.90E-07	calponin homology domain-containing protein	*Tribolium castaneum*
	CL919.Contig2	6386	3.64	gi|762128026|	4.50E-166	calponin homology domain-containing protein	*Crassostrea gigas*
	Unigene35941	922	2.39	gi|762142911|	3.70E-45	calponin homology domain-containing protein isoform X1	*Crassostrea gigas*
	Unigene46074	669	2	gi|762142911|	1.40E-33	calponin homology domain-containing protein isoform X1	*Crassostrea gigas*
	CL1145.Contig2	2337	1.64	gi|762142923|	8.00E-222	calponin homology domain-containing protein isoform X3	*Crassostrea gigas*
	Unigene31595	851	1.2	gi|762120051|	1.80E-83	calponin homology domain-containing protein	*Crassostrea gigas*
	CL1145.Contig3	2080	0.98	gi|762142923|	9.40E-206	calponin homology domain-containing protein isoform X3	*Crassostrea gigas*
	Unigene41313	265	0.92	gi|871234357|	3.90E-16	calponin homology domain-containing protein	*Aplysia californica*
	CL1145.Contig1	2239	0.8	gi|762142923|	4.10E-207	calponin homology domain-containing protein isoform X3	*Crassostrea gigas*
	CL919.Contig1	5545	0.63	gi|762128026|	4.30E-80	calponin homology domain-containing protein	*Crassostrea gigas*
	Unigene12035	227	0.58	gi|762142911|	1.70E-12	calponin homology domain-containing protein isoform X1	*Crassostrea gigas*
	Unigene34566	346	0.32	gi|762126854|	1.50E-07	calponin homology domain-containing protein	*Crassostrea gigas*
***tyrosinase***	CL1107.Contig2	2016	10.61	gi|906541766|	1.40E-97	tyrosinase-like protein-1	*Mytilus coruscus*
	CL1804.Contig1	1007	3.62	gi|906541766|	1.20E-81	tyrosinase-like protein-1	*Mytilus coruscus*
	CL2142.Contig1	2824	9.16	gi|906541766|	3.80E-109	tyrosinase-like protein-1	*Mytilus coruscus*
	CL3071.Contig2	724	52.42	gi|405959193|	1.80E-15	tyrosinase-like protein tyr-3	*Crassostrea gigas*
	Unigene14152	1554	44.84	gi|405964315|	1.40E-49	tyrosinase-like protein tyr-3	*Crassostrea gigas*
	Unigene18799	661	0.58	gi|765826513|	1.30E-15	tyrosinase-like protein 2 precursor	*Crassostrea gigas*
	Unigene28500	1624	11.79	gi|405967028|	8.50E-162	tyrosinase-like protein tyr-1	*Crassostrea gigas*
	Unigene31416	1780	6.17	gi|762072681|	1.90E-61	tyrosinase-like protein 2	*Crassostrea gigas*
	Unigene35768	1619	1324.17	gi|405964315|	4.30E-49	tyrosinase-like protein tyr-3	*Crassostrea gigas*

### Proteomic profile of the *P*. *viridis* shell

The 69,859 unigenes identified from the *P*. *viridis* mantle provided transcriptomic data for shell proteomic analysis. The shell proteins were divided into four parts, the acid-soluble fraction and the acid-insoluble fraction from the myostracum layer, and the same parts from the nacreous layer. The number of the total spectra, the identified spectra, the identified peptides, and the identified proteins of the four samples from MS analysis are listed in Table 2. The unique spectrum number, unique peptide number, protein mass distribution, protein coverage, and the length of the matched peptide are shown in S4 and S5 Figs. The proteomic data of the *P*. *viridis* shell were submitted to http://www.iprox.org/ and http://proteomecentral.proteomexchange.org with the Nos. IPX0001273000 and PXD010566, respectively.

**Table 2:** The MS information of the four samples extracted from *P*. *viridis* shell. M-AIS, the acid-insoluble sample from myostracum layer. M-AS, the acid-soluble sample from myostracum layer. N-AIS, the acid-insoluble sample from nacre layer. N-AS, the acid-soluble sample from nacre layer.

Sample	Total spectra	Identified spectra	Identified peptides	Identified proteins
M-AIS	19986	925	403	147
M-AS	19216	626	271	110
N-AIS	19529	1629	755	277
N-AS	19634	1240	491	204

For the myostracum layer, 110 and 147 proteins with an FDR<0.01 were identified from the acid-soluble and -insoluble fraction, respectively (S1 and S2 Tables). For the nacre layer, 204 and 277 proteins with an FDR<0.01 were identified from the acid-soluble and -insoluble fraction, respectively (S3 and S4 Tables). Considering the overlap between the four sets, we identified a total of 378 proteins from the *P*. *viridis* shell. The nacre layer contributed 330 proteins, and the myostracum layer contributed 183 proteins. In addition, 195 proteins were found to be exclusive to the nacre layer, 48 proteins were exclusive to the myostracum layer, and 135 proteins were shared by both the nacre and myostracum layers. Of the 378 proteins identified from the *P*. *viridis* shell, 317 proteins had homologies in the NR database of NCBI, and 61 proteins had no significant similarity found. Out of the 317 significant matches found in the NR database, 98 proteins were from *Crassostrea*. Other matches were from *Mizuhopecten* (66 proteins), *Mytilus* (63 proteins), *Lingula* (8 proteins), *Haliotins* (4 proteins), and *Perna* (3 proteins) (S1–S4 Tables). In addition, 65 (~20%) identified SMPs from the *P*. *viridis* shell had predicted signal peptides with a peptide length of 16 ~ 27 amino acids. Protein homologous sequence searching was further performed against the NR databases at NCBI. The COG, GO, and KEGG databases were used to functionally classify the identified *P*. *viridis* SMPs.

**Fig 10:**
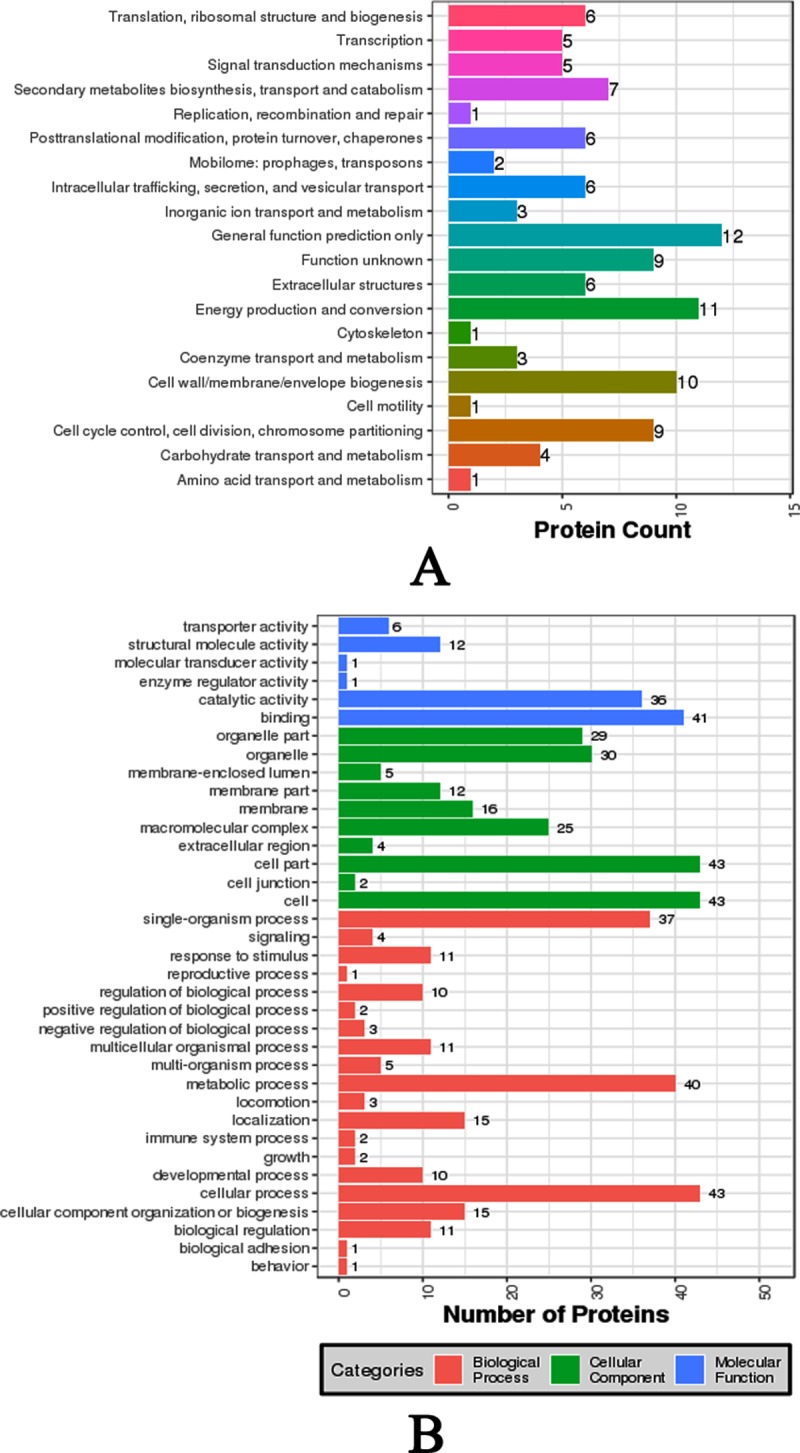
Functionally annotation of the myostracum SMPs identified from *P*. *viridis* shell. **A**: COG annotation of the SMPs from myostracum layer; **B**: GO annotation of the SMPs from myostracum layer.

For the myostracum layer, a total of 59 proteins were assigned to the COG annotation with different categories (Fig 10A). General function prediction only, Energy production and conversion, and Cell wall/membrane/envelope biogenesis were the categories shared by the majority proteins from this layer (Fig 10A). In addition, 66 proteins from the myostracum layer were assigned to GO annotation (Fig 10B). Binding, Cell part/Cell, and Cellular process were the top hits for Molecular function, Cellular component, and Biological processes, respectively (Fig 10B).

**Fig 11:**
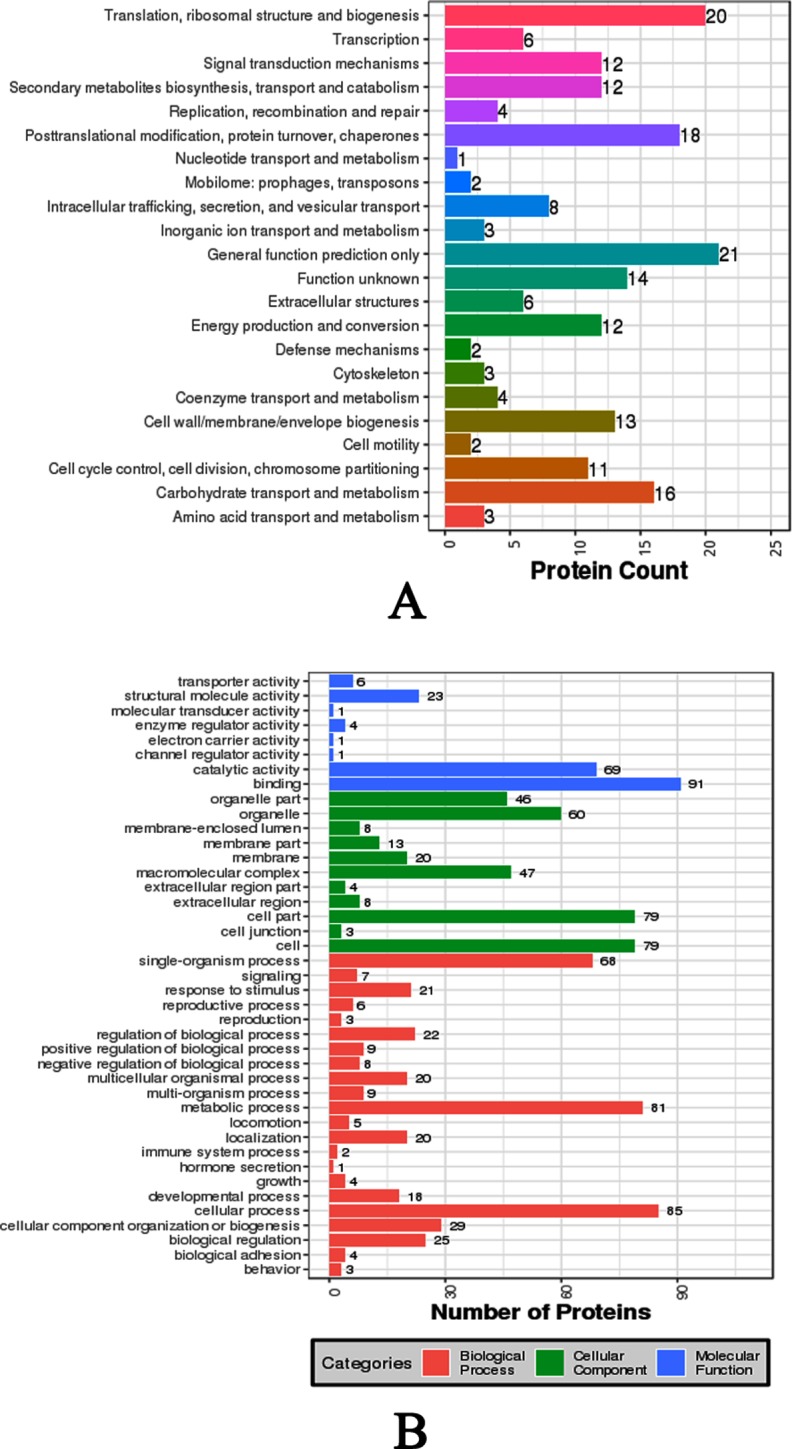
Functionally annotation of the nacre SMPs identified from *P*. *viridis* shell. **A**: COG annotation of the SMPs from the nacrelayer; **B**: GO annotation of the SMPs from the nacre layer.

For the nacre layer, a total of 196 proteins were assigned to COG annotation (Fig 11A). General function prediction only, Energy production and conversion, and Cell wall/membrane/envelope biogenesis were the categories shared by the majority proteins from this layer (Fig 11A). For GO annotation, 135 proteins were assigned and Binding, Cell part/Cell, and Cellular process were the top hits for Molecular function, Cellular component, and Biological processes, respectively (Fig 11B).

According to the KEGG annotation, 123 proteins from the myostracum layer were associated with 163 pathways. For the nacre layer, 241 proteins were associated with 217 KEGG pathways. The top 10 KEGG pathways of these samples are listed in Table 3. Metabolic and Focal adhesion were the pathways associated with the majority of all samples.

**Table 3:** The top 10 KEGG pathways of the identified proteins from the nacre and the myostracum layer of *P*. *viridis* shell.

samples	pathway name	prortein numbers (percentage)	pathway ID
myostracum	Metabolic pathways	20 (16.26%)	ko01100
	Focal adhesion	15 (12.2%)	ko04510
	PI3K-Akt signaling pathway	12 (9.76%)	ko04151
	Human papillomavirus infection	12 (9.76%)	ko05165
	ECM-receptor interaction	12 (9.76%)	ko04512
	Protein digestion and absorption	10 (8.13%)	ko04974
	Adrenergic signaling in cardiomyocytes	9 (7.32%)	ko04261
	Cardiac muscle contraction	8 (6.5%)	ko04260
	Phagosome	7 (5.69%)	ko04145
	Carbon metabolism	7 (5.69%)	ko01200
nacre	Metabolic pathways	47 (19.5%)	ko01100
	Focal adhesion	27 (11.2%)	ko04510
	Human papillomavirus infection	22 (9.13%)	ko05165
	PI3K-Akt signaling pathway	21 (8.71%)	ko04151
	Protein digestion and absorption	18 (7.47%)	ko04974
	ECM-receptor interaction	17 (7.05%)	ko04512
	Carbon metabolism	16 (6.64%)	ko01200
	Ribosome	13 (5.39%)	ko03010
	Proteoglycans in cancer	12 (4.98%)	ko05205
	Cardiac muscle contraction	11 (4.56%)	ko04260

## Discussion

In the present study, we observed that the *P*. *viridis* shell consists of two distinct layers, the nacre and myostracum. As shown in Fig 1, the nacre layer, representing the predominant microstructure with paralleled stacks of tablets, occupies the greatest area of the *P*. *viridis* shell. The myostracum layer with columnar structures is a thin layer embedded in the nacre layer (Fig 1B). In addition, both the nacre and myostracum of the *P*. *viridis* shell were determined as aragonite polymorphs according to the FTIR and XRD profiles (Fig 3). Molluscs present a layered structure that can be categorized into seven kinds of generally accepted structures, including columnar and sheet, prismatic, crossed-lamellar, foliated, homogeneous, and complex crossed-lamellar structures [10, 26]. Most bivalves contain at least three different microstructures and both aragonite and calcite in the shell [27, 28]. Thus, *P*. *viridis* has a simple shell structure compared with the shells from other bivalves, such as *Mytilus* [29], *Crassostrea* [30], *clams* [31], and *pteriomorphs* [32]. Therefore, the *P*. *viridis* shell is a good model for exploring the proteomic map layer by layer.

### Microstructure of *P*. *viridis* shell at the AMS

For bivalves, the adductor muscle is the organ that controls the closing of the valves, and the tight junction between the muscle and shell is a good model of a “bi-material interface” that can be used to develop bio-inspired strategies for connecting dissimilar advanced materials. In the case of *P*. *viridis*, the adductor muscle is fastened strongly to the shell, and it is hard to separate the valves. Galtsoff reported that in *Crassostrea virginica*, the adhesion withstands a pulling force up to 10 kg [33], indicating the high strength, strong adhesion and toughness, and long fatigue lifetime of the adductor muscle–shell interface. In the AMS region of the *P*. *viridis* shell, the myostracum layer is exposed to the inner shell surface and connected directly with the adductor muscles (Fig 1C), suggesting the important roles of the myostracum layer in muscle-shell attachment as well as in the strength of the shell.

The inner surface of the *P*. *viridis* shell is covered by a thin organic film. This film, also named the innermost shell lamella (ISL), has also been reported in other mollusc shells, including *Mercenaria mercenaria*, *Rangia cuneata*, and *Pinctada fucata* [34–36]. The ISL tightly adheres to the calcified shell layer and plays an intermediary role in shell formation [37, 38]. Additionally, the film is believed to play a key role in muscle–shell attachment as an epithelium–film–shell junction for molluscs [12, 15, 16]. In the AMS region, pit structures were observed on the ISL after the adductor muscle was removed by 5% NaOH (Fig 2B), as well on as the calcified shell surface after deproteinization by 20% NaOH (Fig 2D), suggesting the presence of organic fibres existing in both the film and the beneath calcium carbonate crystal. Similar results were also found in the shell of the scallop *Patinopecten yessoensis*, and a model suggesting that the adductor muscle was attached to the shell by the insertion of organic fibres and fibril bundles branched from the muscle into the pits on the myostracum was proposed [39]. In addition, the ultrastructure of muscle–shell attachment in gastropods revealed a similar conclusion [12]. The mineral phase of the *P*. *viridis* shell is intimately associated with biological matrixes in both the myostracum and nacre layers. Different types of matrices have been observed in bivalve shells: the interlamellar matrix sheets separate adjacent crystal layers, and the intercrystalline matrix separates adjacent CaCO_3_ crystals within the shell layer [40]. Considering the presentation of the ISL structure on the shell inner surface, the shell matrix in this study is represented not only by the interlamellar and intercrystalline matrix inside the shell but also by the matrix from the ISL covering on the shell surface.

### Shell proteome of *P*. *viridis*

In the present study, we characterized the mantle transcriptome of *P*. *viridis* using an Illumina system to identify putative biomineralization-related genes and provide a dataset for the next *P*. *viridis* shell proteomic analysis. We obtained over 83 Mb of raw sequence data and were able to assemble these reads into 69,859 transcripts, 29,921 (42.83%) of which were annotated by BLAST searches against five public databases (S2 Fig). The size of transcripts from the *P*. *viridis* mantle transcriptome was lower than that of *M*. *coruscus* [20], *Tectus pyramis* [41], and *Patinopecten yessoensis* [42] but higher than *Pecten maximus* [43], *P*. *maximus* [44], *C*. *gigas* [44], and *M*. *truncata* [45]. Similar to other molluscs, the *P*. *viridis* mantle transcriptome was heavily dominated by muscle-related genes (Myosin, Actin, etc.), reflecting the contractile nature of this organ and mitochondrial respiratory chain-related genes (NADH dehydrogenase, cytochrome c, arginine kinase, etc.), demonstrating that the mantle is a metabolically and transcriptionally active tissue. In addition, 111 biomineralization-related transcripts were identified from the *P*. *viridis* mantle transcriptome (Table 1), representing a highly conserved core set of genes involved in shell formation.

Using a combined proteomic/transcriptomic approach, we identified a total of 378 SMPs associated with the nacre and myostracum layers in the *P*. *viridis* shell. Of this protein set, 132 SMPs identified with more than two matched unique peptides are listed (Table 4) and discussed here. We also observed that approximately 20% of identified SMPs presented a predicted signal peptide, suggesting that these proteins are secreted by the mantle tissue through a classical cellular secretion pathway. As shown in Table 4, of the 132 SMPs, 69 are exclusive to the nacre, 12 to the myostracum, and 51 SMPs are shared by both layers. According to the protein scores and the number of matched peptides, the Myosin-tail domain-containing proteins, Filament-like proteins, Chitin-binding domain-containing proteins, FN3 (Fibronectin-3) domain-containing proteins, Peroxidase-like proteins, Calponin-homology (CH)/Calponin domain-containing proteins, and Filamin-like proteins represent the most abundant molecules in *P*. *viridis* shell (Table 4).

**Table 4:** SMPs identified with more than two matched peptides from the *P*. *viridis* shell by LC-MS/MS. The MS/MS spectra were used for searching against the Illumina-sequencing based *P*. *viridis* mantle transcriptome using Mascot software. N represents the nacre layer and M represents the myostracum layer.

Transcripts ID	Shell layer	Protein Q-score	Matched peptides	Homologous protein [species]	Homologous ID / E-value	Sequential features
**CL3395.Contig3**	**M**	7.7937	2	RS-rich protein-2 [Mytilus coruscus]	AKS48164.1/4e-146	—
**Unigene42298**	**M**	4.9918	2	—	—	—
**Unigene6711**	**M**	6.7523	2	—	—	—
**Unigene34438**	**M**	7.7937	2	Reticulocyte-binding protein 2-like protein a [Crassostrea gigas]	EKC19657.1/4e-39	CAP(PF00188)
**Unigene54043**	**M**	6.7850	3	keratin 8 [Epinephelus coioides]	ACH73075.1/5e-58	Filament(PF00038)
**CL2084.Contig1**	**M**	122.8763	38	filament-like protein-2 [Mytilus coruscus]	AKS48133.1/0.0	Filament(SM001391); LTD(PF00932)
**Unigene46263**	**M**	7.7937	2	—	—	Internal repeat 2
**Unigene58165**	**M**	9.9049	3	EGF-like domain containing protein [Oryctes borbonicus]	KRT86894.1/5e-29	LDLa(SM000192)
**Unigene1696**	**M**	6.2551	2	flotillin-1-like isoform X1 [Mizuhopecten yessoensis]	XP_021361554.1/0.0	PHB(SM000244); Flot(PF15975)
**Unigene15208**	**M**	7.5557	2	—	—	Signal peptide(1–17); Efh(SM000054)
**Unigene5251**	**M**	9.8017	3	tubulin alpha-1A chain [Mizuhopecten yessoensis]	XP_021370666.1/0.0	Tubulin(SM000864); Tubulin_C(SM000865)
**Unigene52317**	**M**	7.7937	2	—	—	—
**Unigene57065**	**N**	14.8267	4	H5p [Mytilus edulis]	AFR31803.1/ 1e-79	—
**Unigene39231**	**N**	6.2986	2	phosphoglycerate mutase [Hymenolepis microstoma]	CDS33978.1/8e-130	PGAM(SM000855)
**CL1023.Contig5**	**N**	8.3901	2	protease inhibitor-like protein-1 [Mytilus coruscus]	ALA16013.1 /5e-67	A2M(SM001360)
**CL1023.Contig4**	**N**	8.6714	3	protease inhibitor-like protein-1 [Mytilus coruscus]	ALA16013.1 /4e-46	A2M_comp(PF07678); SCOP d1ayoa_
**CL1231.Contig1**	**N**	10.7490	3	protease inhibitor-like protein-1 [Mytilus coruscus]	ALA16013.1 /3e-109	A2M_N (PF01835)
**Unigene6218**	**N**	6.4703	2	CD109 antigen-like isoform X1 [Crassostrea virginica]	XP_022323515.1/0.0	A2M_N_2(SM001359); A2M(SM001360); Thiol-ester_cl(PF10569); A2M_comp(PF07678); A2M_recep(SM001361)
**CL1387.Contig2**	**N**	6.3279	2	beta-actin [Meretrix meretrix]	AEK81538.1/0.0	ACTIN(SM000268)
**Unigene7385**	**N**	5.1611	2	S-adenosylhomocysteine hydrolase [Crassostrea ariakensis]	ACT35639.1 /0.0	AdoHcyase_NAD(SM000997)
**CL818.Contig4**	**N**	4.7938	2	Protein hu-li tai shao [Crassostrea gigas]	EKC20098.1 /0.0	Aldolase_II(SM001007)
**Unigene16016**	**N**	6.3969	2	amidohydrolase [Colwellia psychrerythraea]	WP_081967745.1/4e-161	Amidohydro_1(PF01979)
**CL2840.Contig1**	**N**	8.3901	2	RS-rich protein-1 [Mytilus coruscus]	AKS48138.1 /4e-86	Arg(11.2%)
**Unigene6047**	**N**	8.3901	2	arginine kinase-like protein-1 [Mytilus coruscus]	AKS48144.1/0.0	ATP-gua_PtransN(PF02807); ATP-gua_Ptrans (PF00217)
**CL3748.Contig1**	**N**	14.7293	4	radixin-like isoform X4 [Crassostrea virginica]	XP_022292008.1/0.0	B41(SM000295); FERM_C (SM001196); ERM(PF00769)
**Unigene20345**	**N**	9.3624	3	Protein flp [Crassostrea gigas]	EKC28749.1 /7e-54	Beta-lactamase(PF00144)
**Unigene1886**	**N**	6.4703	2	sodium/potassium-transporting ATPase subunit alpha-like [Crassostrea virginica]	XP_022323941.1/0.0	Cation_ATPase_N(SM000831); E1-E2_ATPase(PF00122); HAD (PF12710); Cation_ATPase_C(PF00689)
**CL2603.Contig1**	**N**	12.9166	4	transgelin-like protein-3 [Mytilus coruscus]	AKS48154.1/8e-109	CH(SM000033)
**Unigene31135**	**N**	10.4751	4	sushi-like protein [Mytilus coruscus]	AKS48157.1 /0.0	ChtBD2(SM000494)
**Unigene51584**	**N**	8.3901	2	mucin-2-like [Crassostrea virginica]	XP_022335717.1/ 6e-134	ChtBD2(SM000494); LamG(SM000282)
**Unigene12178**	**N**	10.7490	3	byssal amine oxidase-like protein 1 [Mytilus coruscus]	ANN45949.1 /0.0	Cu_amine_oxidN2(PF02727); Cu_amine_oxid(PF01179)
**Unigene44023**	**N**	6.2336	2	eukaryotic initiation factor 4A-I-like [Crassostrea virginica]	XP_022288944.1/0.0	DEXDc(SM000487); HELICc(SM000490)
**Unigene17159**	**N**	8.3901	2	myosin essential light chain [Crassostrea gigas]	CAD91423.1/3e-88	EF-hand_7(PF13499)
**CL4656.Contig1**	**N**	15.7389	4	retrograde protein of 51 kDa-like isoform X4 [Crassostrea virginica]	XP_022320110.1/0.0	Filament(SM001391); SCOP(d1ifra_)
**CL1116.Contig1**	**N**	7.7945	2	SH3 domain-binding glutamic acid-rich protein-like isoform X5 [Mizuhopecten yessoensis]	XP_021366845.1/8e-25	Glutaredoxin(PF00462)
**Unigene49324**	**N**	8.3901	2	Fructose-bisphosphate aldolase [Crassostrea gigas]	EKC30386.1/0.0	Glycolytic(PF00274)
**Unigene3946**	**N**	8.3901	2	elongation factor 1 alpha [Mytilus galloprovincialis]	BAD35019.1/0.0	GTP_EFTU (PF00009); GTP_EFTU_D2 (PF03144); GTP_EFTU_D3(PF03143)
**Unigene3720**	**N**	11.2787	3	Histone H2B type 1-M [Tupaia chinensis]	ELV13502.1 /1e-66	H4(SM000417)
**Unigene19938**	**N**	16.7803	4	mitochondrial H+ ATPase a subunit [Pinctada fucata]	ABJ51956.1 /0.0	HAS-barrel(PF09378); ATP-synt_ab (PF00006); ATP-synt_ab_C(PF00306)
**Unigene32384**	**N**	8.3901	2	HSP90 [Mytilus coruscus]	ALL27016.1 /0.0	HATPase_c(SM000387); HSP90(PF00183)
**Unigene5067**	**N**	5.5154	2	small heat shock protein 22 [Mytilus galloprovincialis]	AEP02967.1 /2e-92	HSP20(PF00011)
**CL3397.Contig2**	**N**	18.3658	5	filamin-A-like isoform X5 [Crassostrea virginica]	XP_022315171.1/0.0	IG_FLMN(SM000557)
**Unigene635**	**N**	6.6520	2	heparan sulfate proteoglycan-like protein-1 [Mytilus coruscus]	AKS48136.1 /3e-57	IGc2(SM000408)
**Unigene40680**	**N**	11.4569	4	twitchin [Mytilus galloprovincialis]	BAC00784.1/0.0	IGc2(SM000408); FN3(SM000060); Pkinase_Tyr(PF07714)
**Unigene49709**	**N**	6.4366	2	—	—	Internal repeat 1
**CL2227.Contig1**	**N**	12.5852	3	type I secretion C-terminal target domain-containing protein [Comamonas terrigena]	WP_098066268.1/6e-19	Internal repeat 1
**Unigene53667**	**N**	6.8220	2	Papilin [Echinococcus granulosus]	XP_024351037.1 /4e-09	KU(SM000131)
**CL3668.Contig2**	**N**	4.8186	2	Papilin [Mizuhopecten yessoensis]	OWF36203.1 /9e-150	Kunitz_BPTI(PF00014); KU(SM000131); IGc2(SM000408); PLAC(PF08686)
**Unigene7565**	**N**	6.2556	2	sushi-like protein [Mytilus coruscus]	AKS48157.1/5e-27	Laminin_G_3 (PF13385)
**Unigene38699**	**N**	8.3901	2	—	—	Ldh_1_N (PF00056); Ldh_1_C(PF02866)
**CL4067.Contig1**	**N**	6.9252	2	inactive pancreatic lipase-related protein 1-like [Crassostrea virginica]	XP_022340243.1 /8e-125	Lipase(PF00151)
**Unigene41989**	**N**	12.9692	4	ADP,ATP carrier protein 3, mitochondrial-like [Crassostrea virginica]	XP_022315856.1/0.0	Mito_carr(PF00153)
**Unigene2452**	**N**	8.3901	2	mCG10343, isoform CRA_c [Mus musculus]	EDL21528.1/5e-177	Mito_carr(PF00153)
**CL955.Contig5**	**N**	6.6520	2	catchin protein [Mytilus galloprovincialis]	CAB64664.1 /0.0	Myosin_tail_1 (PF01576)
**Unigene41665**	**N**	7.0836	2	Na+/K+ ATPase beta subunit [Doryteuthis opalescens]	ABO61331.1/9e-80	Na_K-ATPase(PF00287)
**Unigene34337**	**N**	17.5772	5	Phosphoenolpyruvate carboxykinase [GTP] [Crassostrea gigas]	EKC27095.1/0.0	PEPCK(PF00821)
**Unigene34402**	**N**	6.4366	2	flotillin-2a-like isoform X2 [Crassostrea virginica]	XP_022341488.1/0.0	PHB(SM000244); Flot (PF15975)
**Unigene43**	**N**	12.5852	3	pyruvate kinase PKM-like isoform X7 [Crassostrea virginica]	XP_022311730.1/0.0	PK(PF00224); PK_C (PF02887)
**CL105.Contig2**	**N**	6.9252	2	transcriptional activator protein Pur-beta-like isoform X2 [Mizuhopecten yessoensis]	XP_021353033.1/5e-148	PUR(SM000712)
**Unigene24855**	**N**	5.3625	2	RNA-binding protein [Pinctada fucata]	ABP04054.1 /0.0	RRM(SM000360)
**CL3802.Contig3**	**N**	9.0108	3	twitchin-like protein-1 [Mytilus coruscus]	AKS48140.1 /0.0	S_TKc(SM000220); IGc2(SM000408); IG(SM000409)
**Unigene13632**	**N**	7.0836	2	Neuroserpin [Orchesella cincta]	ODM93513.1 /5e-22	Serpin(PF00079)
**Unigene42847**	**N**	10.7490	3	integral membrane protein 2B-like [Stylophora pistillata]	XP_022807324.1 /0.014	Signal peptide(1–18)
**Unigene33532**	**N**	8.3901	2	heme-binding protein 2-like [Crassostrea virginica]	XP_022345420.1/5e-28	Signal peptide(1–18)/SOUL(PF04832)
**Unigene240**	**N**	6.2989	2	—	—	Signal peptide(1–19)/scop d1gkub1
**Unigene6555**	**N**	21.9945	6	byssal metalloproteinase inhibitor-like protein 1 [Mytilus coruscus]	ANN45954.1 /4e-09	Signal peptide(1–20)/NTR(SM000206)
**Unigene17542**	**N**	6.6520	2	pernin precursor [Perna canaliculus]	AAK20952.1/0.0	Signal peptide(1–20)/Sod_Cu(PF00080)
**Unigene23862**	**N**	6.1542	2	metalloproteinase inhibitor 3-like [Crassostrea virginica]	XP_022302578.1/6e-10	Signal peptide(1–22)/C345C(SM000643)
**Unigene2638**	**N**	7.8147	3	shell matrix protein-like [Mizuhopecten yessoensis]	XP_021364733.1 /7e-123	Signal peptide(1–23)/SCOP d1c4ra_
**Unigene34954**	**N**	5.2978	2	—	—	Thr(15.1%); Lys(12.4%); Pro(10.9%); Gln(10.7%)
**Unigene49503**	**N**	11.5256	4	protein-glutamine gamma-glutamyltransferase K-like isoform X1 [Mizuhopecten yessoensis]	XP_021343535.1/0.0	Transglut_N(PF00868); TGc(SM000460); Transglut_C(PF00927)
**CL4276.Contig2**	**N**	19.2462	5	Tropomyosin [Saccostrea glomerata]	AVD53650.1/1e-139	Tropomyosin(PF00261)
**CL2714.Contig1**	**N**	14.6887	4	troponin T, skeletal muscle-like isoform X1 [Mizuhopecten yessoensis]	PVD33201.1/3e-90	Troponin(PF00992)
**CL299.Contig1**	**N**	27.9989	8	tubulin alpha-1A [Enchytraeus cf. crypticus SL-2017]	AOR07106.1 /0.0	Tubulin (SM000864); Tubulin_C (SM000865)
**CL4310.Contig5**	**N**	10.5980	3	tubulin beta-4B chain isoform X1 [Heterocephalus glaber]	XP_004848801.1 /0.0	Tubulin (SM000864); Tubulin_C(SM000865)
**Unigene26**	**N**	10.5492	3	putative alpha-tubulin [Oikopleura dioica]	AAP80594.1/0.0	Tubulin(SM000864); Tubulin_C(SM000865)
**CL1757.Contig1**	**N**	5.7771	2	tubulin beta chain isoform X1 [Parasteatoda tepidariorum]	XP_015920593.1/0.0	Tubulin(SM000864); Tubulin_C(SM000865)
**Unigene31729**	**N**	6.3126	2	sushi-like protein [Mytilus coruscus]	AKS48157.1/2e-37	VWA(PF00092)
**Unigene34815**	**N**	28.4017	10	Collagen alpha-3(VI) chain [Crassostrea gigas]	EKC21865.1/0.0	VWA(SM000327); EGF(SM000181)
**Unigene31163**	**N**	14.8605	4	gamma-crystallin N-like [Acanthaster planci]	XP_022092298.1/3e-74	XTALbg(SM000247); ETX_MTX2(PF03318)
**CL579.Contig12**	**N**	7.0836	2	titin-like [Mizuhopecten yessoensis]	XP_021347443.1/0.0	ZU5(PF00791); DEATH(SM000005)
**CL2840.Contig3**	**N+M**	21.3773	6	RS-rich protein-1 [Mytilus coruscus]	AKS48138.1/0.0	—
**Unigene37654**	**N+M**	6.2986	2	phage head morphogenesis protein [Clostridium amazonitimonense]	WP_032122034.1/3.8	—
**CL678.Contig2**	**N+M**	29.9862	9	actin [Crassostrea virginica]	XP_022325998.1/0.0	ACTIN(SM000268)
**CL563.Contig3**	**N+M**	25.0163	7	actin [Crassostrea virginica]	XP_022325998.1/0.0	ACTIN(SM000268)
**CL2840.Contig2**	**N+M**	9.4067	3	RS-rich protein-1 [Mytilus coruscus]	AKS48138.1/0.0	Arg(11.2%)
**CL2840.Contig7**	**N+M**	21.5286	6	RS-rich protein-2 [Mytilus coruscus]	AKS48164.1/4e-146	Arg(12.8%); Ser(11.3%); Val(10.1%)
**CL93.Contig1**	**N+M**	19.4072	5	ATP synthase subunit beta, mitochondrial [Mizuhopecten yessoensis]	XP_021356377.1/0.0	ATP-synt_ab_N (PF02874); AAA(SM000382)
**Unigene20161**	**N+M**	41.3441	12	calponin-like protein-1 [Mytilus coruscus]	AKS48134.1 /0.0	CH(SM000033); Calponin(PF00402)
**Unigene51075**	**N+M**	38.1946	10	Alpha-actinin [Crassostrea gigas]	EKC43084.1 /0.0	CH(SM000033); SPEC(SM000150); EFh(SM000054); efhand_Ca_insen (SM001184)
**Unigene2367**	**N+M**	95.1889	25	matrix protein-1 [Mytilus coruscus]	AKS48137.1 /0.0	ChtBD2(SM000494); Laminin_G_3 (PF13385)
**CL1804.Contig1**	**N+M**	26.6198	9	collagen alpha-1(II) chain isoform X2 [Otolemur garnettii]	XP_003793620.1/2e-35	Collagen(PF01391)
**Unigene44900**	**N+M**	6.6520	2	Collagen alpha-1(IV) chain [Crassostrea gigas]	EKC43052.1 /4e-172	Collagen(PF01391); C4(SM000111)
**Unigene34861**	**N+M**	15.4737	4	sarco/endoplasmic reticulum calcium ATPase isoform A [Pinctada fucata]	ABS19815.1/0.0	E1-E2_ATPase(PF00122); HAD (PF12710); Cation_ATPase_C(PF00689)
**CL1011.Contig5**	**N+M**	83.8526	26	filament-like protein-2 [Mytilus coruscus]	AKS48133.1/0.0	Filament(SM001391); LTD(PF00932)
**CL1886.Contig2**	**N+M**	89.0478	27	filament-like protein-2 [Mytilus coruscus]	AKS48133.1 /0.0	Filament(SM001391); LTD(PF00932)
**Unigene30013**	**N+M**	12.5852	3	glycine-rich cell wall structural protein-like [Crassostrea virginica]	XP_022339053.1 /4.9	Gly(30.9%); Ala(13.8%)
**Unigene35808**	**N+M**	8.7624	3	chitinase-3 [Hyriopsis cumingii]	AFO53261.1/0.0	Glyco_18(SM000636); ChtBD2(SM000494);
**CL758.Contig1**	**N+M**	10.0437	3	glyceraldehyde-3-phosphate dehydrogenase [Littorina littorea]	AJA37895.1/0.0	Gp_dh_N(SM000846); Gp_dh_C(PF02800)
**Unigene37757**	**N+M**	7.5557	2	heat shock protein beta-1-like isoform X1 [Folsomia candida]	XP_021944507.1/1e-12	HSP20(PF00011)
**Unigene40545**	**N+M**	40.4858	10	filamin-like protein-1 [Mytilus coruscus]	AKS48135.1 /0.0	IG_FLMN(SM000557)
**Unigene8686**	**N+M**	16.1597	5	—	—	Internal repeat 1
**Unigene25503**	**N+M**	12.5852	3	—	—	Internal repeat 1
**Unigene32111**	**N+M**	8.3901	3	apolipoprotein A1/A4/E [Desulfobacca acetoxidans]	WP_013707457.1/0.007	Internal repeat 1
**Unigene40702**	**N+M**	11.0548	4	nacre protease inhibitor-like protein 1 [Mytilus galloprovincialis]	AKQ70858.1 /2e-47	KU(SM000131)
**Unigene18932**	**N+M**	10.0785	3	heat shock protein 71 [Perna viridis]	ABJ98722.1/0.0	MreB_Mbl(PF06723)
**Unigene9058**	**N+M**	40.2960	10	myosin heavy chain, striated muscle-like isoform X6 [Crassostrea virginica]	XP_022317649.1/0.0	Myosin_N (PF02736); MYSc(SM000242)
**Unigene10181**	**N+M**	247.6202	64	paramyosin-like isoform X3 [Crassostrea virginica]	XP_022322570.1/0.0	Myosin_tail_1(PF01576)
**CL1023.Contig1**	**N+M**	70.0233	20	paramyosin-like isoform X3 [Crassostrea virginica]	XP_022322570.1/0.0	Myosin_tail_1(PF01576)
**CL955.Contig7**	**N+M**	50.2367	14	catchin protein [Mytilus galloprovincialis]	CAB64664.1 /0.0	Myosin_tail_1(PF01576)
**Unigene6274**	**N+M**	27.0882	9	myosin heavy chain [Mytilus galloprovincialis]	CAB64662.1 /0.0	MYSc(SM000242); IQ(SM000015); Internal repeat; Myosin_tail_1 (PF01576)
**Unigene3051**	**N+M**	23.0788	6	mucin-3A-like [Mizuhopecten yessoensis]	XP_021361490.1 /0.002	Pro(10.6%); Thr(10.3%)
**Unigene483**	**N+M**	13.7472	4	—	—	SCOP d1gkub1
**CL185.Contig3**	**N+M**	32.2540	8	DNA N6-methyl adenine demethylase-like isoform X4 [Crassostrea virginica]	XP_022319021.1 /1e-44	SCP(SM000198); ShKT(SM000254);
**Unigene52025**	**N+M**	8.3901	2	cyclin-dependent kinase 1 isoform X2 [Trichechus manatus latirostris]	XP_023595093.1/1.1	Ser(10.9%); Asn(10.1%)
**Unigene51161**	**N+M**	10.6491	3	myostatin [Mytilus chilensis]	AGU13048.1/0.0	Signal peptide((1–23); TGFb_propeptide(PF00688); TGFB(SM000204)
**Unigene48535**	**N+M**	31.6407	8	—	—	Signal peptide(1–16)/Gly(16.3%); Asn(12.7%)
**CL474.Contig2**	**N+M**	15.5873	4	valine-rich protein-like isoform X4 [Crassostrea virginica]	XP_022307204.1/3e-09	Signal peptide(1–17)
**Unigene37646**	**N+M**	19.9339	5	P,N-U7 [Pinctada fucata]	AKV63173.1/0.71	Signal peptide(1–17)/Arg(10.5%)
**Unigene39912**	**N+M**	62.9260	15	shell protein-6 [Mytilus coruscus]	AKI87977.1 /0.0	Signal peptide(1–17)/FN3(SM000060)
**Unigene39610**	**N+M**	19.0895	5	shell mytilin-3 [Mytilus coruscus]	AKI87980.1/7e-40	Signal peptide(1–17)/Gly(15.3%); Phe(14.1%); Ser(10.7%)
**CL4671.Contig1**	**N+M**	23.0788	6	valine-rich protein-like isoform X4 [Crassostrea virginica]	XP_022307204.1/3e-09	Signal peptide(1–17)/Gly(18.3%); Val(16.7%); Pro(11.3%)
**Unigene58102**	**N+M**	20.9753	5	KS-rich protein [Mytilus coruscus]	AKS48160.1/5e-14	Signal peptide(1–18)/Arg(10.7%)
**Unigene51088**	**N+M**	40.3825	10	Matrilin-2 [Crassostrea gigas]	EKC40227.1 /1.2	Signal peptide(1–19)/SCOP(d1dqca_)
**CL3951.Contig2**	**N+M**	15.7389	4	—	—	Signal peptide(1–20)/Arg(10.8%);
**Unigene12260**	**N+M**	12.5852	3	—	—	Signal peptide(1–21)/Gly(23.6%); Met(15.4%)
**Unigene15131**	**N+M**	7.0836	2	2-oxoglutarate dehydrogenase [Halioglobus sp. HI00S01]	KZX60278.1/0.011	Signal peptide(1–22)/scop d1stfi_
**Unigene7251**	**N+M**	8.3901	2	collagen pro alpha-chain [Haliotis discus]	BAA75668.1/0.0	Signal peptide(1–22)/VWC(SM000214); Collagen (PF01391); Internal repeats; COLFI(SM000038)
**Unigene6649**	**N+M**	57.1628	14	byssal peroxidase-like protein 1 [Mytilus coruscus]	ANN45955.1 /0.0	Signal peptide(1–23)/An_peroxidase (PF03098)
**Unigene2736**	**N+M**	8.3901	2	—	—	Signal peptide(1–24)
**Unigene43460**	**N+M**	29.3655	7	fibril-forming collagen alpha chain-like [Crassostrea virginica]	XP_022341069.1 /0.0	Signal peptide(1–24)/VWC(SM000214); Collagen (PF01391); Internal repeats; COLFI(SM000038)
**Unigene57834**	**N+M**	25.1704	6	Collagen alpha-5(VI) chain [Mizuhopecten yessoensis]	OWF49639.1 /1e-152	Signal peptide(1–27)/VWA(SM000327);

### Most abundant SMPs in the *P*. *viridis* shell

Myosin-tail domain-containing proteins have been identified from many bivalve shells, such as *Mytilus* [19, 20] and clam [46] and were previously considered cellular contaminants [47, 48]. However, the myosin-tail domain-containing protein is also an actin-binding protein involved in mechanochemical cross-bridges [49], and two myosin-tail domains can form a coil-coil structure and then assemble into the macromolecular thick filament [50]. In the present study, both actin and Filament-like proteins were identified confidently from the *P*. *viridis* shell. These results indicate the possibility that myosin-tail domain-containing proteins, together with actin and filament-like proteins, may be associated with biomineralization, as suggested by Jackson *et al*. [51]. In addition, filament-like proteins have also been identified from the shells of *Mytilus* [19, 20], snails [52], and *Magellania venosa* [51], suggesting a conserved distribution of this protein in mollusc shells. Chitin-binding domain-containing proteins, also representing a conserved protein group from the shell matrix, have been identified from *Crassostrea* [53, 54], *Mytilus* [19, 20], *Pinctada* [55], and *scallops* [55]. The main role of the Chitin-binding domain in the shell is to interact with the chitin, which is a key component of the mollusc shell [56]. FN3 domain-containing proteins have been reported as calcite-specific (prism layer) shell proteins and were absent in the aragonite layer, such as in *M*. *truncata*, which has a shell consisting of aragonite entirely [57] and the nacre layer of *Unionoida* [58]. However, FN3 domain-containing proteins were identified from the aragonite shell layer of *Mytilus* (nacre and myostracum layer) [19, 20] and *Lottia* (cross-lamellar layer) [59]. These results indicate that FN3 domain-containing proteins may play different roles in different mollusc shell layers rather than being calcite-specific shell proteins. Peroxidase-like proteins have been identified from the shells of *Mytilus* [19, 20], *Lottia* [59, 60], *Crassostrea* [54], and *Pinctada* [55]. As a redox enzyme, the putative function of Peroxidase-like proteins in shell biomineralization remains enigmatic. CH/calponin domain-containing proteins have been identified from the shells of *Mytilus* [19, 20] and *Crassostrea* [54]. The potential actin-binding function of the calponin domain [61, 62] confers these proteins with possible roles in calponin-actin interaction, which may be involved in shell biomineralization or myostracum muscle attachment. Filamin-like proteins have been identified from *Mytilus* [19, 20] and *Crassostrea* [54], although the real function of filamin in biomineralization is still unknown. Recently, a filamin-like protein of *P*. *fucata* (pf-filamin-A) was reported as a calcium-sensing protein related to the synthesis and transportation of calcium-containing nanoparticles on the surface of the nacreous layer [63], indicating an important role for filamin in biomineralization. The presence of these abundant matrix proteins in both the nacre and myostracum layers of the *P*. *viridis* shell suggested an important function in shell formation and demonstrate the conservation of these proteins in various mollusc shells, especially bivalve shells.

### SMPs with biomineralization-related domains in the *P*. *viridis* shell

In addition to SMPs being highly abundant, many *P*. *viridis* SMPs were identified with the presence of reported biomineralization-related domains, such as Kunitz, A2M, WAP, EF-hand, PDZ, VWA, Collagen domain, and low-complexity regions with abundant certain amino acids, such as R-rich, G-rich, S-rich, and T-rich [64–66]. These proteins can be broadly categorized into six groups: enzymes, structural proteins, immune-related proteins, low-complexity-region-containing proteins, other proteins, and uncharacterized proteins.

Various enzymes have been identified from many mollusc shells, and some of them, such as tyrosinase, chitinase, SOD, and arginine kinase, are believed to play roles in biomineralization. For example, tyrosinase has possible functions in periostracum tanning [67], biomineral hydrogel maturation, hardening [68], and shell damage repair [69]. Chitinase is secreted outside of the cell to reconstruct the chitin network, suggesting a function in shell chitin metabolism during shell formation and growth. Chitinase had been identified from other bivalve shells and showed high conservation across the Metazoa [55]. In bivalves, chitin can join to form a sheet or layered structure sandwiched between two layers of protein to form a protein/polysaccharide network, enabling complex large-scale composite shells to be fabricated [68, 70, 71]. Additionally, chitin is also responsible for the mechanical strength and functionality of the resulting material [72]. In the case of the *P*. *viridis* shell, the distribution of the chitinase-sensitive matrix (or chitin-like material) at the nacre layer from AMS-A showed a sheet-like pattern (Fig 5G). However, this laminar structure was thinner than that in the nacre layer from the AMS region (Fig 5H). Considering that the AMS is the most important stress distribution site on the shell, the chitin-like material, mainly presented at the interface of the myostracum and the nacre layer at the AMS, may act as superglue to join these layers together and make them whole to support the stress from the adductor muscle.

Structural proteins, such as actins, tubulins, paramyosin/myosin, and collagens, have been identified from the shell of many mollusc species [19, 20, 54, 55]. The presence of these structural proteins in shells is much debated, and the presence of actins, tubulins and paramyosins/myosins identified from the shell matrix has been previously described as contaminants [73]. However, we cannot exclude the possible roles of these structural proteins in shell formation because of the wide distribution and high abundance of these cytoskeletal proteins in many mollusc shells, even those that have been washed thoroughly with hypochlorite solution [51] or sodium hydroxide [55]. In addition, actin has been suggested to be an organic remnant from the migration of secreted biomineral components from outer mantle epithelial cells to the shell [51, 74]. Furthermore, many shell matrix proteins have been identified with actin-binding domains, such as PDZ, THY, and calponin, from *Mytilus* [19, 20], indicating that a possible protein interactional network mediated by actin may exist in the mollusc shell. Collagens have been identified from many mollusc shells, including *Mytilus* [19, 20], *Hyriopsis cumingii* [75], and *Pinctada fucata* [76]. A collagenous matrix has been suggested to be involved in the deposition of calcium phosphate in hard tissues, such as bone, dentin, and cementum [77, 78]. In these tissues, minerals exist in the basic organic frameworks, which are formed by collagen fibrils [79, 80]. These results indicate the important roles of collagen in biomineralization. In addition, the myostracum layer in the AMS area is susceptible to collagenase digestion, indicating the presence of a collagen-like matrix in the interface between the myostracum and the nacre layer (Fig 5). The specific distribution of the collagenase-sensitive matrix in the AMS area suggests that collagen might play an important role in muscle–shell attachment (myostracum-nacre interface, for example). The current results are in accordance with the observation of Galtsoff [33] in the study of muscle–shell attachment of *C*. *virginica*, where collagenase significantly reduced the adherence of the muscle to the shell.

### Immune-related proteins in the *P*. *viridis* shell

The existence of immune-related proteins within the shell organic matrix is not new in molluscs because immune-related proteins have been broadly identified from the shell of *Bivalvia* [19, 20, 54, 55], *Gastropoda* [51, 59], and Brachiopoda [51, 81]. Recently, a significant number of SMPs containing immune-related domains were identified from the shell of four highly divergent bivalves, including the Pacific oyster (*C*. *gigas*), the blue mussel (*M*. *edulis*), the clam (*M*. *truncate*), and the king scallop (*P*. *maximus*), indicating the important roles of these SMPs in defence against pathogens [82]. Of this protein group, the protease-inhibitor-like proteins (PILPs) are important not only for assisting the immune system against microbial invasion [83] but also as a part of the shell matrix framework [84]. In this study, abundant PILPs containing conserved proteinase inhibitor domains, such as WAP, KU, A2M, Serpin, and C345C, were identified in both the nacre and myostracum layers (Table 3). It has been suggested that PILPs are involved in biomineralization processes as shell matrix protection systems against proteolysis during shell formation and thus protect shell structures from proteases secreted by fouling organisms and predators [85, 86]. Another immune-related protein, mucin (or mucin-like protein), was also reported as a biomineralization-related protein [87, 88] and identified first from the fan mussel as a matrix protein associated with the nacre layer [89, 90]. In this study, two proteins (Unigene51584 and Unigene3051, as shown in Table 3) with homologies to the mucin family were identified from the *P*. *viridis* shell, confirming the existence of mucin-like proteins in the mollusc shell. However, the real function of mucin-like proteins in biomineralization is still unknown, although it was suggested as one of the constituents of a gel-like matrix that can be pushed aside when the nacre tablet grows laterally [68]. Unlike Mucoperlin (the mucin-like protein from the *Pinna nobilis* shell), one of the mucin-like proteins (Unigene51584) from the *P*. *viridis* shell contains a chitin-binding domain (ChtBD2) in its sequence (Table 3), indicating a possible function of this protein in the interaction with chitin.

### SMPs with LCRCPs in the *P*. *viridis* shell

In our study, Gly-, Arg-, Ser-, and Thr-rich shell proteins were also identified from *P*. *viridis*. Most of these proteins lacked significant homologs and known domains, but low-complexity regions existed in the sequence. Low-complexity region-containing proteins (LCRCPs), characterized by a high abundance of certain amino acids (such as Glu, Asp, Gly, Ala, Met, Arg, Thr, and Ser) in their sequence, have been reported previously from many mollusc shells [19, 20, 54, 55, 91]. Some of these proteins, identified in previous works, are well-known shell matrix proteins, such as MSI60 and carbonic anhydrase (CA), in which MSI60 is a Gly- and Ala-rich protein [92, 93], and CA is a ubiquitous enzyme with high abundance of Gly, Asn, and Asp in mollusc shells [94]. LCRCPs have been proposed to confer proteins with the enhanced flexibility for the transport of proteins across the cell membrane and the intrinsic plasticity that allows a single protein to recognize several biological targets without sacrificing its specificity [95], which may aid in the transportation of shell proteins via mantle epithelial cell membranes and the interaction among the shell proteins. In addition, the low-complexity region of LCRCPs has been proposed to adopt a specific structure when bound to the calcium carbonate crystal surface [96]. In parallel, it has been proposed that the numerous Gly and Ala residues of some shell proteins, such as silk-like fibroins, are implicated in the tensile mechanical properties of these proteins [97].

### Comparison of the SMPs from *P*. *viridis* with other bivalves

Irrespective of shell morphology and microstructure, we found that many SMPs identified from *P*. *viridis* shell are shared by other bivalves. Table 5 summarizes the protein features of identified SMPs from *P*. *viridis* and from other mollusc models, including Unionoids [58], Pinctada [55, 76], Ostreidae [54], Myoida [57], and Mytilus [19, 20]. As shown in this table, some SMPs, such as S-rich, G-rich, peroxidase, VWA-domain-containing, Glyco-hydro-18, and Lam-G, are shared by various bivalves shell. Thus, we suggest that these SMPs are evolutionarily conservative and represent part of the “basic tool kit” for the construction of the CaCO3 molluscan exo-skeleton, as previously described by Marie, et al [58]. Taken together, these results indicate that a conserved molecular machinery exists for shell biomineralization in bivalves. However, this assumption needs further validation via functional analyses.

**Table 5:** Comparison of SMPs with domain features in the shells of various Bivalves. The “√” indicates identification of this protein in this species.

organisms		*Elliptio complanata*	*Villosa leinosa*	*Pinctada spp*.	*Mytilus spp*.	*Perna viridis*	*Pinctada spp*.	*Mytilus spp*.	*Perna viridis*	*Crassostrea gigas*	*Mya truncata*
shell microstructures		nacre	nacre	nacre	nacre	nacre	prisms	prisms	prisms	prisms	whole shell
RLCD containing	S-rich	√	√		√	√	√	√	√	√	
	G-rich	√	√	√	√	√	√	√		√	
	V-rich	√			√	√	√	√			
	T-rich	√	√	√			√				
	M-rich	√	√	√	√						
	P-rich	√									
	Q-rich	√	√	√	√		√	√		√	
extracellular matrix	VWA	√	√	√	√	√		√		√	√
	Filament-like				√	√		√	√		
	H5P					√					
	EGF				√	√	√	√	√	√	
	CCP\SUSHI	√	√		√	√	√				√
	MSP-like	√	√							√	
enzyme	SH3					√					
	Tyrosinase			√			√	√		√	
	Peroxidase					√	√		√	√	
Ca-binding	Calponin				√	√			√		
	D-rich	√	√	√	√		√				
	EF-hand		√			√	√		√	√	
polysaccharide interacting	Glyco-hydro-18	√	√		√	√		√	√	√	√
	Actin					√		√	√		
	mucin-2-like					√			√		
	Chit_bind	√	√			√	√		√	√	
	Lam-G	√	√	√	√	√	√	√	√		√
	CBD2	√	√	√	√		√	√		√	
immunity	WAP	√	√							√	
	Peptidase C1				√			√		√	
	macroglobulin	√	√	√	√			√		√	
	C1q				√			√		√	
	TIMP				√		√				
other orphans		17	21	11	20	80	17	16	38	35	15
references		[58]	[58]	[55,76]	[19,20]	this study	[55,76]	[19,20]	this study	[54]	[57]

## Conclusions

In this paper, the microstructure and mineral polymorphs of the *P*. *viridis* shell were studied, and two shell layers, the nacre and myostracum with the same aragonite polymorph, were characterized by SEM, FTIR, and XRD. We also tested the microstructural changes of the shell samples after chitinase and collagenase digestion. In the AMS region, collagenase digestion can reduce the adhesion between the myostracum layer and the nacre layer, suggesting the important roles of collagens in myostracum-nacre interface. Chitinase digestion resulted in long cracks at the interlamellar of the nacre layer and the myostracum/nacre interface, indicating the important role of chitin in shell formation. Using Illumina sequencing, the mantle transcriptome of *P*. *viridis* was investigated, and a total of 69,859 unigenes were generated, of which approximately 43% were assigned putative functions. The most highly expressed genes were those with dominant biological functions of contraction and energy production. Using a combined proteomic/transcriptomic approach, we identified a total of 378 SMPs from *P*. *viridis* shell, of which 132 SMPs were identified as having more than two matched unique peptides. Of the 132 SMPs, 69 were exclusive to the nacre, 12 to the myostracum, and 51 were shared by both. The Myosin-tail domain-containing proteins, Filament-like proteins, and Chitin-binding domain-containing proteins represented the most abundant molecules. In addition, SMPs containing biomineralization-related domains, such as Kunitz, A2M, WAP, EF-hand, PDZ, VWA, Collagen domain, and LCRCPs with abundant certain amino acids, were also identified from the *P*. *viridis* shell. These proteins can be broadly categorized into six groups, and the SMP-containing domains, such as immunomodulatory, might imply their role in different biological functions, not only in biomineralization. Our results present for the first time the proteome of the *P*. *viridis* shell and increase the knowledge of SMPs in this genus.

## Supporting information

S1 Fig Length of the unigenes from the *P*. *viridis* mantle transcriptome. (TIF)

S2 Fig Venn diagram of the annotation of *P*. *viridis* mantle transcriptome in NR, KEGG, InterPro, SwissProt, and KOG database. (TIF)

S3 Fig Species distribution of the annotated unigenes from *P*. *viridis* mantle transcriptome. (TIF)

S4 Fig The MS information of the identified proteins from myostracum layer of *P*. *viridis* shell. For the acid-insoluble sample, the unique peptide number, the length of matched peptide, the protein coverage, the protein mass distribution, and the unique spectrum number are shown in A ~ E, respectively. For the acid-soluble sample, the results are shown in F ~ J, respectively.
(TIF)

S5 Fig The MS information of the identified proteins from nacre layer of *P*. *viridis* shell. For the acid-insoluble sample, the unique peptide number, the length of matched peptide, the protein coverage, the protein mass distribution, and the unique spectrum number are shown in A ~ E, respectively. For the acid-soluble sample, the results are shown in F ~ J, respectively.
(TIF)

S1 Table 110 proteins identified with FDR<0.01 from the acid-soluble samples of myostracum layer of *P*. *viridis* shell. (DOCX)

S2 Table 147 proteins identified with FDR<0.01 from the acid-insoluble samples of myostracum layer of *P*. *viridis* shell. (DOCX)

S3 Table 204 proteins identified with FDR<0.01 from the acid-soluble samples of nacre layer of *P*. *viridis* shell. (DOCX)

S4 Table 277 proteins identified with FDR<0.01 from the acid-insoluble samples of nacre layer of *P*. *viridis* shell. (DOCX)
