# Supplementary figures and images for: Microstructure and in-depth proteomic analysis of Perna viridis shell
Source: PLoS One. 2019 Jul 19;14(7):e0219699. doi: 10.1371/journal.pone.0219699 (PMC6641155; doi:10.1371/journal.pone.0219699)

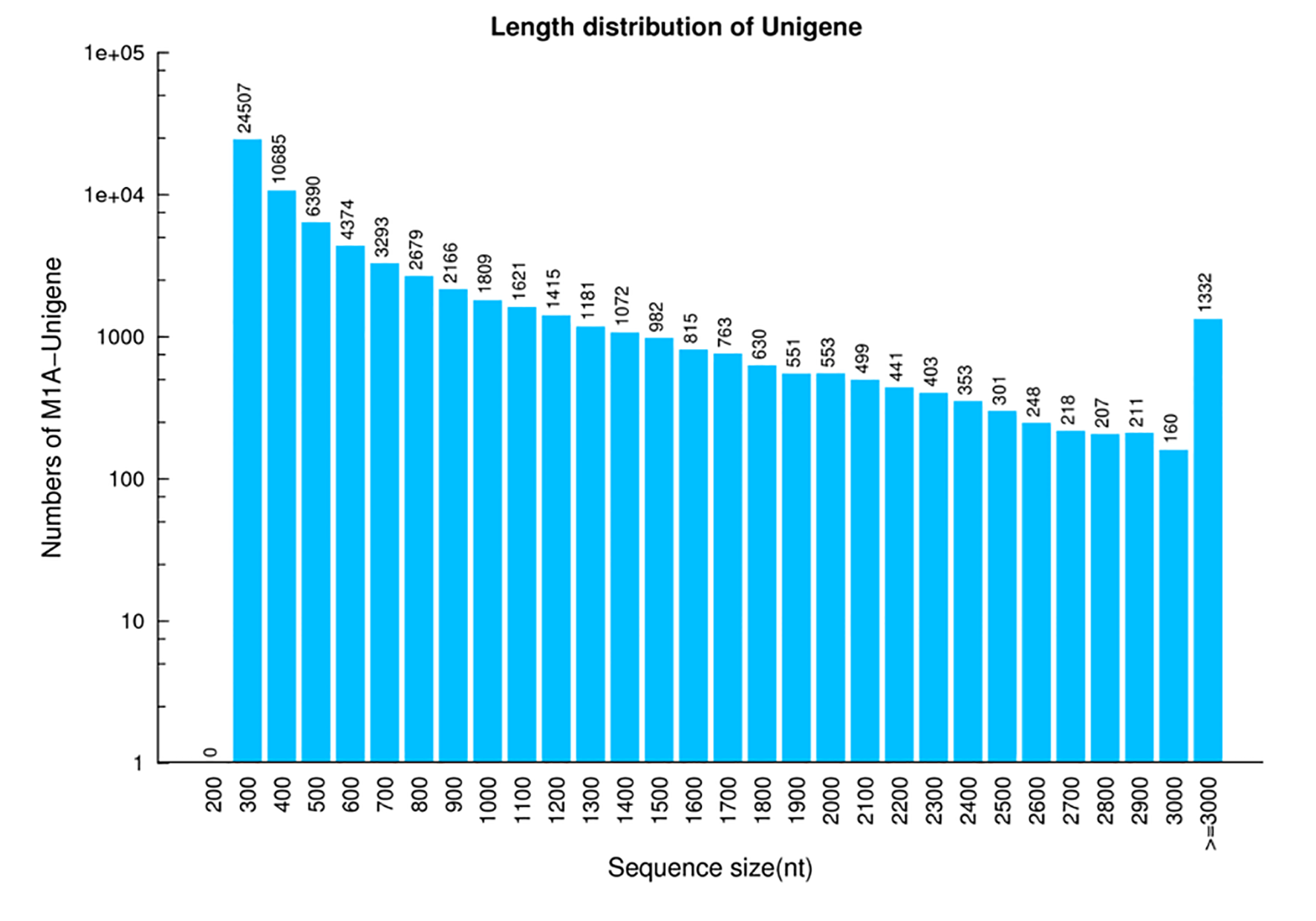

Supplement: S1 Fig — (TIF) [file pone.0219699.s001.tif]

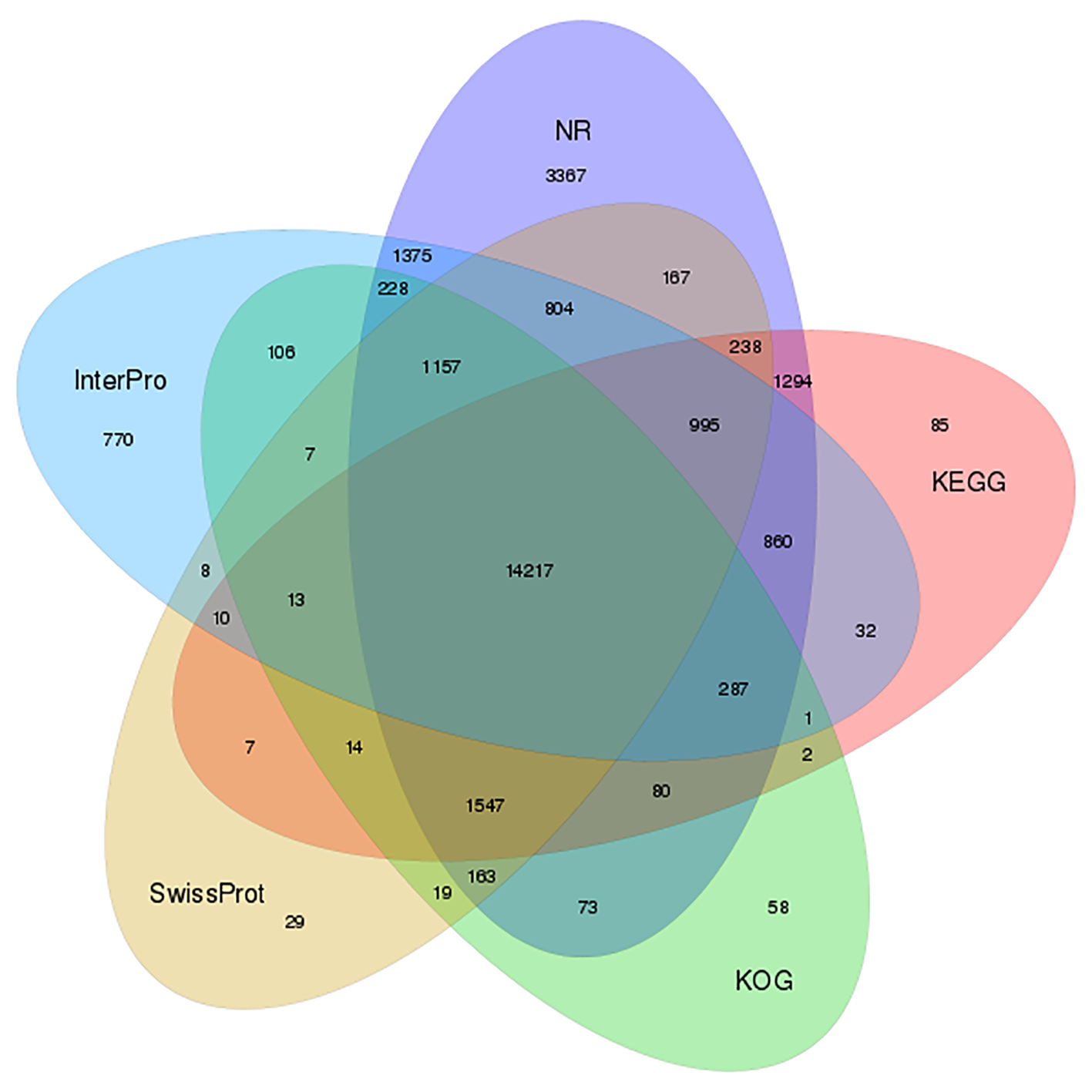

Supplement: S2 Fig — (TIF) [file pone.0219699.s002.tif]

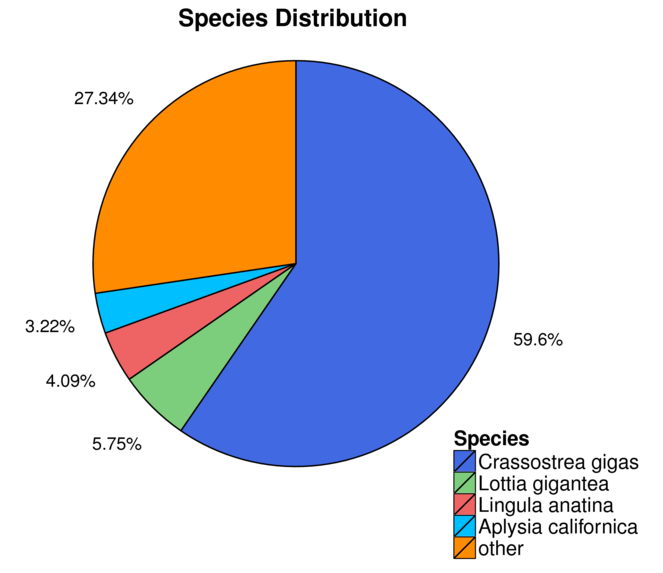

Supplement: S3 Fig — (TIF) [file pone.0219699.s003.tif]

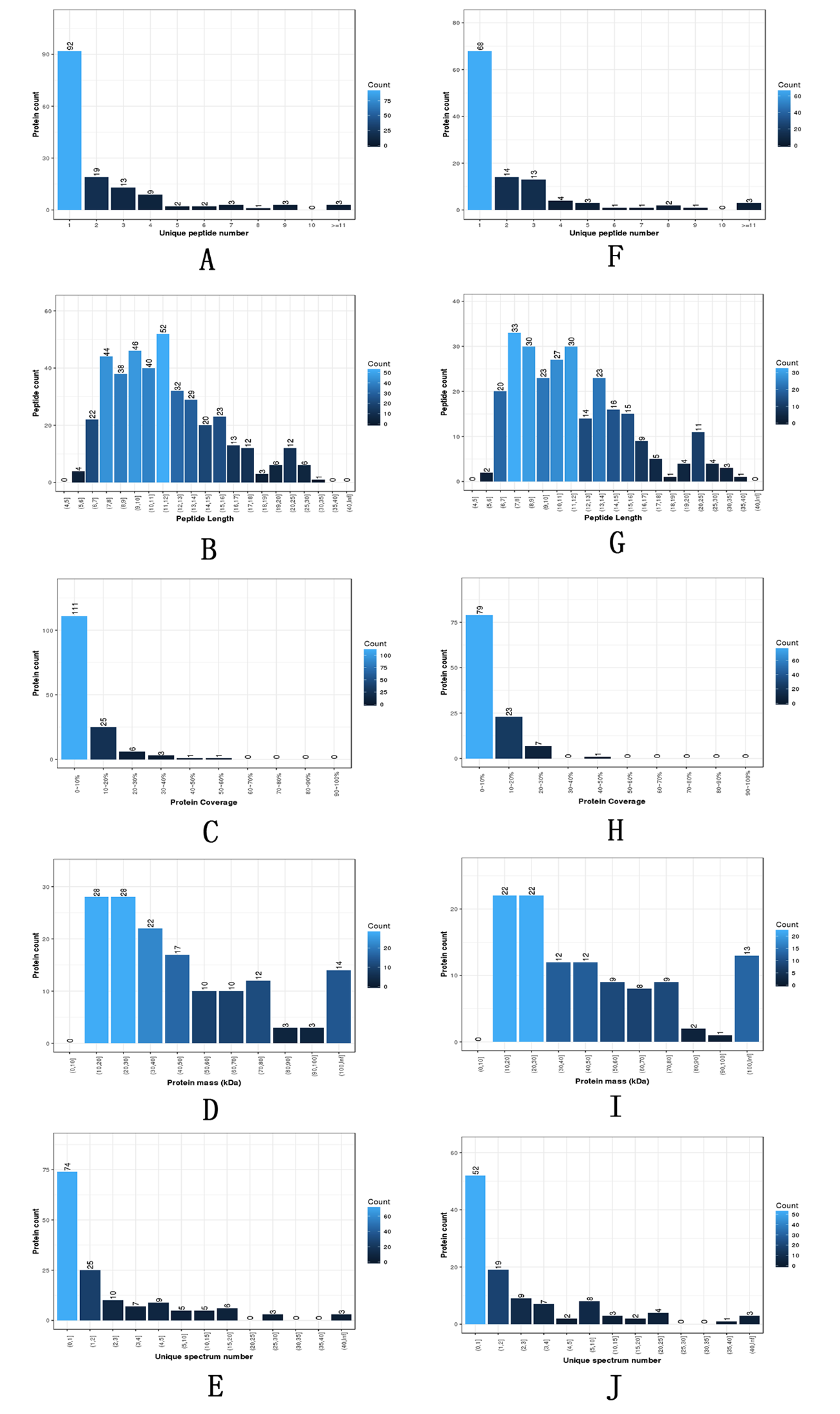

Supplement: S4 Fig — For the acid-insoluble sample, the unique peptide number, the length of matched peptide, the protein coverage, the protein mass distribution, and the unique spectrum number are shown in A ~ E, respectively. For the acid-soluble sample, the results are shown in F ~ J, respectively. (TIF) [file pone.0219699.s004.tif]

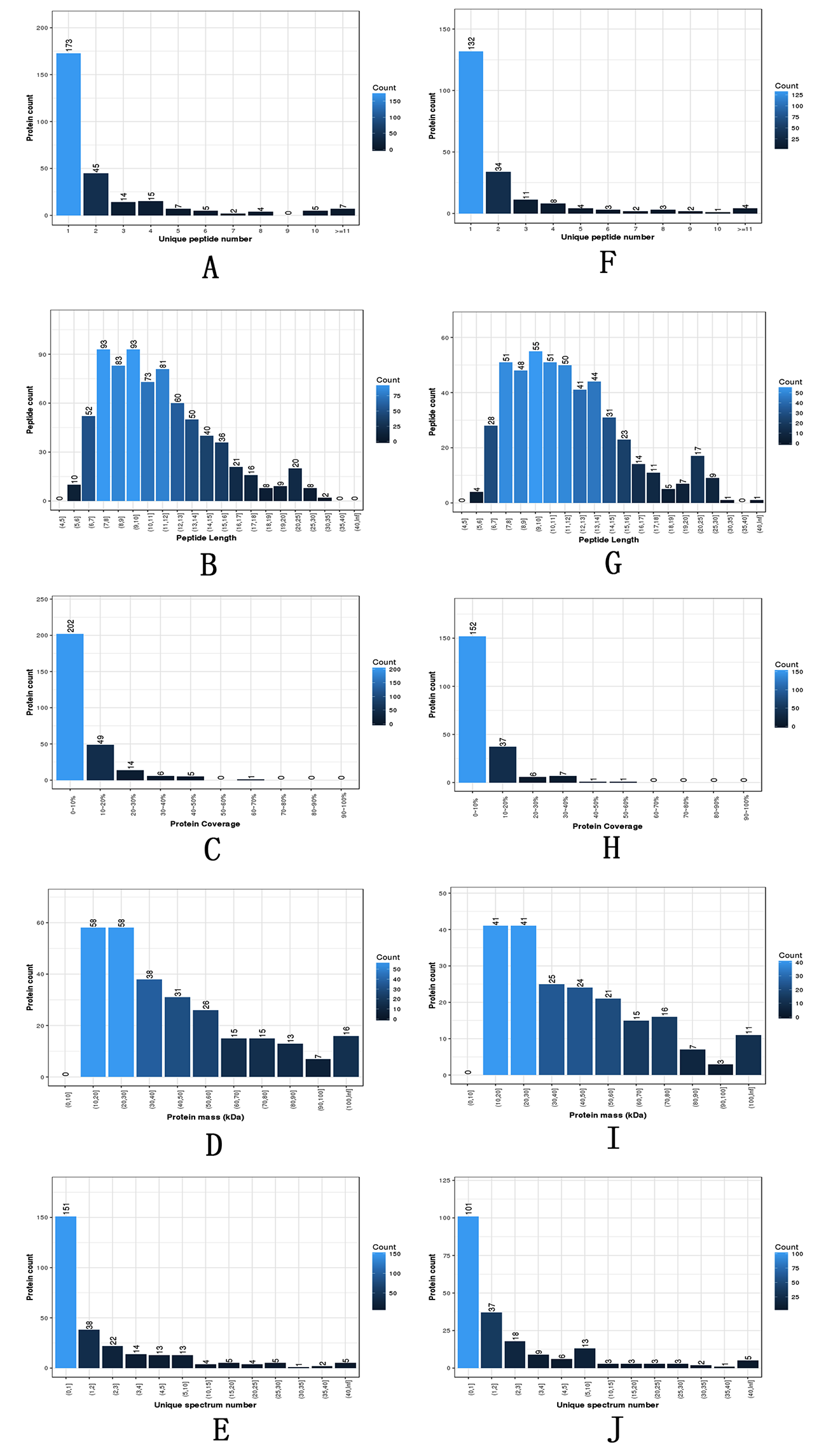

Supplement: S5 Fig — For the acid-insoluble sample, the unique peptide number, the length of matched peptide, the protein coverage, the protein mass distribution, and the unique spectrum number are shown in A ~ E, respectively. For the acid-soluble sample, the results are shown in F ~ J, respectively. (TIF) [file pone.0219699.s005.tif]
