# Supplementary material for: Microstructure and in-depth proteomic analysis of Perna viridis shell
Source: PLoS One. 2019 Jul 19;14(7):e0219699. doi: 10.1371/journal.pone.0219699 (PMC6641155; doi:10.1371/journal.pone.0219699)
Supplement: S1 Table — (DOCX) [file pone.0219699.s006.docx]

**S1 Table**

| **matched unigene** | **score** | **matched peptides** | **number of matched peptides** | **protein sequence** | **Homologous protein [species]** | **Homologous ID / E-value** | **Domain and signal peptide(SP)** |
| --- | --- | --- | --- | --- | --- | --- | --- |
| **CL1886.Contig2** | 83.8526 | ASVGGGSGGTVYTR;ASVGGGSGGTVYTRTVEYGMGR;DIQNEYDNKVDQIR;DTTEENREFWK;ELAALAYR;EVVYTFR;EYESMQSEHTMETVK;FITLENTSSQR;FLEAQNR;GAAQLSEMITEYETK;GSAQEAGINDLVFR;IDLNNETLNHLDAENR;IDLNNETLNHLDAENRR;LADLEAR;LEIQNSSLQEEMNGLR;LLESEESR;LNQQLSDYESEINMLR;NAQLEAQYNSLLR;NVIEQSMNTQSR;QTLEEEMEFLK;RTISSLETER;SSGLGNLSPGSYEK;SSIGPSMR;SSTVVNR;SSTVVNRSSIGPSMR;TISSLETER | 26 | MSKSSERITEKRTVITSSSSNYDDGDDSIYYKSGIQPRSSTVVNRSSIGPSMRASVGGGSGGTVYTRTVEYGMGRSSGLGNLSPGSYEKVSNTGVMTVKSSREKEKKDMQDLNERFANYIEKVRFLEAQNRKLAGELEHLKTKWGKETSAIKSMYEQELAEARKLIDDLTRDKNKLEIQNSSLQEEMNGLRRQMDDLKKYHALDQEQINKLNQQLSDYESEINMLRRTISSLETERARDKDRINKLQGEVDRLRIDLNNETLNHLDAENRRQTLEEEMEFLKKVHEQELKELAALAYRDTTEENREFWKSELSQAIRDIQNEYDNKVDQIRGDMESYYNLKVQEFRTGATKQNMEVTHVKEENKKLVKSISDLKGRLADLEARNAQLEAQYNSLLREYESMQSEHTMETVKLKEEITNLRAEMEAILVELQSLMDAKLSLELEIAAYRKLLESEESRVGMRNVIEQSMNTQSRGAAQLSEMITEYETKGDSHSSMKMMRGEVSAKTTYQKTSTGPVSIAEVNPEGKFITLENTSSQRREVNLDGWKIRRELDGQREVVYTFRNFTLKPHKSVKIFARGSAQEAGINDLVFRDEETWGVGSQVSTCLVNEKGEEKATHRQRTAYN | filament-like protein-2 [Mytilus coruscus] | AKS48133.1/0.0 | Filament(SM001391); LTD(PF00932) |
| **Unigene10181** | 70.0233 | CDELGAENAQLR;DLDSDVSTSTR;DLENELEADQR;ELEDALDSER;ELEGALDNANR;ELELQLEETQR;ENGQLQAALR;IAIQQELEDAR;IQELEDNCEQLR;IRDLENELEADQR;IRELEDALDSER;LEEAIGSSTTFSEVSR;MEADIAAMQSDLDDALNAQR;NAENELGEVSAR;NQLSISER;QNLQVQLAALQSDYDNLNAR;RMEADIAAMQSDLDDALNAQR;SLLEHAER;TLVEVETR;YEEESEAASNLR | 20 | MSLLRDLDSDVSTSTRIVRHTYNVYRGSSPGTQNRLEGRIRELEDALDSERELRLRYEKQSAELTFQLDQLSDRLEEAIGSSTTFSEVSRKREAEVSKVRKDLELASAQFEATEANMRRRHQEALNDLTDQLEHMGKAKARAEKEKNQLIIEIDSLQGINDGLQKAKMSADSKIDALEGSNGRLKINVDDLTRQLNDANSAKARLTQENFDLQHQVQELDGANAGLAKAKAQLQILCDDLKRNLDDESRQRQNLQVQLAALQSDYDNLNARYEEESEAASNLRAQLSKVNADYAALKTKYDKELIAKQEELEEIKRRLSVRIQELEDNCEQLRTRCNSLEKTKNKLTAEIREITIELENTQIIVQDLTKRNRQLENDNAALQKRCDELGAENAQLRNDKANLEQEVYRLKVANAELAEKNGNLERENGQLQAALREAQNELKSANRQINELTALKAQLEAERDNLAAALRDTEEALRDAEAKLAAAQAALNQLRAEMEQRLREKDEEIDSIRKSSARAIDELQRTLVEVETRYKTEITRIKKKYETDIRELEGALDNANRANAEYLKQIKSLQNRNRELELQLEETQRQLDDARNQLSISERKRIAIQQELEDARSLLEHAERARKNAENELGEVSARLTEVQLQVTALTNDKRRMEADIAAMQSDLDDALNAQRAAEERADRLQAEVNRLADELRQEQENYKNAESLRKQLEIEIREITVRLEEAEAFAQREGKRQIAKLQARIRDLENELEADQRRLREAAASARKFERQWKEVVQQADDDRRQVAELTSLTDQLTMKCKTYKRMIEEAEDVANITMNKYRKAQSMIDEAEQRADMAEKNLTAVRRSRSMSVSREVTRVVRV | paramyosin-like isoform X3 [Crassostrea virginica] | XP_022322570.1/0.0 | Myosin_tail_1(PF01576) |
| **Unigene2367** | 50.0326 | AIGSASGLLASK;ASLLLQSAR;DISNGGLAFDHSHIYLR;GGSTSASGSVSAGGSVGSSR;GLGASSGFSSAGAAASASGLGSASLGSTR;GLGSVSVTTGGLGQDLR;GLNGIGGSVTSSLTSK;HGSISGSGISSLDSR;LLTALTK;LSFDNDER;MYLWGFQSK;SGSSSGSSGSSGSSGSGGSSGSSSFGSSTSGSDSR;SRPEPIISNCGPNGDSSVEIVVYR;TGGNLEIR | 14 | GAGAGAGSGVGLGFGVGAGAGAGSGTGLGVGAGAGAGAGSGIGQGIGIGAAGAGSGIGSGAGAGIGVGSGIGFGAGAGAGAGAGSGIGIGVGAGAGAGAGAGLGIGSGIGAGAGAGAGAGSGVEIGMGAGIGAGAGSGFGQGIGATSGLGSSSSGSWGSAGSGESSCSCASQGPKSETIQASAKLAVVGDRNVLIAKMPRAKVVDSDGLGDFLLEFPNLTIQPTLTEKIASPAELFGMNMNKGTHSTDDSDILVVALLPKGSKLGSKMFQPKSSGSGSSSGSSSSSGTGSSSGSSSSSASTNDNPSVSVDTSQDDGKLKIKIKAENVKDSTASIVKNQLDKIKSDEISVDTSKDDGKLKIKIKAKVDDLSKSDINALSSESSYSGSSSGSGSSGGSGSAGGSGSAGGLGSAGGSGSASGSGSAGGSSAAGGWGSNGGLGFSGDLGSAGSSTSAGGSGAASGSTGGSGSVGISGSISVSGSTGGSGINVGLGSAGALGTAGLSGSVGGSKLTVGLGNARGGSTSASGSVSAGGSVGSSRGSGSARAIGSASGLLASKIKASLLLQSARGLGASSGFSSAGAAASASGLGSASLGSTRGLGSVSVTTGGLGQDLRSGSSSGSSGSSGSSGSGGSSGSSSFGSSTSGSDSRLKIEKSKDDGKIKYKIKTVKGKGDSKTKSEYKLVVDKSDGSSSKVKVKFGKSDQDRSSSKIKHGSISGSGISSLDSRGKSYRREEDGDKWKLKSSGSRGLNGIGGSVTSSLTSKKQQIAEAVKVLKALKASQKASNALTAGVSLKAKLPDTRYKLPEQGGTLNILSQKLTKDIKFKQSRDNDERKKLSYKLSFDNDERKSRNRLLTALTKTSTNTVVPKTTGGAKVPNLEGLCLIYLGAFVDAIGYAPLPGQCHKLVQCFYLGGKLKAVARDCPAGMFWDQNTLSCRPPGDVLCFEDKCLIPGTLHYRRNGGCNCFYKCRDGISEPSCCPKGFRYDDDKGCVPAFGSLACDDECETPTTLTTQVTVSSCPSLPDPNNKYGYLAPEHGGLRIRACPAGTIYSASQCQCKSNMNGSGAMRGSLRKQYRQCSAEFNINFDDGFKDISNGGLAFDHSHIYLRKGKAVFGGNSRMYLWGFQSKYLGKTFAIKTRVKVNKGAGRSRPEPIISNCGPNGDSSVEIVVYRGKLIFKAKTSDNPDTVYFNEKYDDDKWMDITYYYDGNYFGGSCNGRSFKQRTGGNLEIRDNPMTIGLCSGKNPGFHGEIDELEIYTACIPKGF | matrix protein-1 [Mytilus coruscus] | AKS48137.1/0.0 | ChtBD2（SM000494）;Pfam Laminin_G_3(PF13385) |
| **CL1011.Contig5** | 26.6198 | GAPGPAGPR;GPIGPEGPSGQGLPGPQGPPGR;GPMGPEGPQGPPGLAGPR;GQPGPQGSPGLR;GQVGIPGPR;GSPGLGGTSTR;MGAVGAPGR;TPHTGPPPGYGWSQQR;YPPYGNSYGPSAYQQR | 9 | GPPGKDGRPGMRGLTGKPGNAGIQGPIGPPGPQGPPGKDGRPGMRGLTGKPGNAGIQGPIGPPGPQGPPGKDGKVGLQGAPGVAGKPGTNGVPGMRGLTGRPGKQAIPIKAAAIPGPPGPIGPQGLIGPAGPKGANGRPGMRGLTGRPGAHGNDGKAGPPGKDGLPGKPGPPGAAGRPGMRGLTGRPGAGKDGPQGPPGKDGAPGRPGMRGLTGRPGAGKEGPPGKDGAPGKPGQRGIRGWRGWRGFKGAPGPAGPRGPMGPEGPQGPPGLAGPRGSPGLGGTSTRQTVVRGPPGPPGPQGTRGPIGPEGPSGQGLPGPQGPPGRGQPGPQGSPGLRGATGSPGLQGKPGIPGKPGAPGMQGLPGPIGNPGPPGAGVKGMGKEQVAVQGPPGPPGPPGAAGRPGKDGHPGVVGPMGPRGHPGEPGMMGPHGPPGKDGKPGPKGDSGAEGPPGQVGPMGPAGPQGKAMQATHHDIVQGLPGPPGPPGERGQVGIPGPRGYRGHPGAKGDYGARGPPGRMGAVGAPGRMGQPGPPGRQGLDGAVGPIGKPGPKGECSCGGPPMGAMRPPMGAGMGPRMGPGPYRPHMPYMAPKTPHTGPPPGYGWSQQRYPPYGNSYGPSAYQQRSNSLIGGTGKPSGSWRKKPVDTEAEMK | collagen alpha-1(II) chain isoform X2 [Otolemur garnettii] | XP_003793620.1/2e-35 | Collagen(PF01391) |
| **Unigene51088** | 28.9330 | EGSSDAQEIMQDR;QSLNNSPMFNSR;QTSLQLSSAR;SPLLSQDLATIR;SSFSQSSLPMMQSR;SSLSLLDR;TVVNPTDNFAALMER;YIEIVHGNVHER | 8 | MYQEGIILIFFIFISHSNGQGCPHSATPVPGDPTRYIEIVHGNVHERNCAPGTEYNTFTCDCSVFSGASIQALMDGSAGSHPDLLNMPLETLQKEFLQLTSQRAPQSTRTDSRRTSKRRKSKTAEFQYNPKKKESQESILFGLLADSALLNLLPTNPPSKAVKHVEKPQFKSEFIDYSLLAPTKAPKIKTFSALNLPKVTPKPKLKSPLLSQDLATIRAFLKERREQRAREKALLKTKQSPITSSASTSSIGLSGTKNAFSFDSGLKILGSSTTKQSTASNLGLNDLRTSAAKSTSSLDIGFNDYHKAALASNTGGLNTNNVKSSVGIAVPTPDAGLNEFRSSLSLLDRNYPTPTPHSRDTFANVKTVVNPTDNFAALMERRKLEQALITKSEPSSSISGISNTASKSSNGLGGLDATKKAILKNLVARLNALEEKNKGTATSSQQQNSMMSQTSSNSDQMQGQSTSLSQRQSLNNSPMFNSRQTSLQLSSARSSFPQSRSSFSQSSLPMMQSRQTPFSMMFRGVAAKEGSSDAQEIMQDRITEMGGAAALAMGAPLSMINSEISSQLSSTMLENGPLPFLM | uncharacterized protein LOC111135614 [Crassostrea virginica] | XP_022341547.1/0.53 | Signal peptide(1-19);SCOP(d1dqca;chitin-binding) |
| **CL185.Contig3** | 30.2228 | AESPLPDDVLIER;APPAFQQPPPR;HPPPPHQPNQPPPPR;QPAPQQHR;QPQYQQPQQPPVAR;QQHPQPLPPQQPPR;QQQPQYPQQQPAYR;QQYQSSQQPYQGQR | 8 | PEVNLWNGSEMKLINVWTKVWTVFGLWNIAPTLGQGPMAMGGIGGASPSRGGKMTQKFINTILKLHNDYRRTEGASNMKKLRWSRALQRDAQLWANKCRYTHAYGKWGENLFKAESPLPDDVLIERAVNEWYYEKMSWKFTPDCNEACHYTQVVWAESEEIGCAYKRCTTLMLMEEFVMNGWMLVCYYNTQGNIIGKMPYSVGKACSACKPGYKCDRGLCDKQRTVHKYLPQHNQQKPKPVFRAPPAFQQPPPRHAPPPRHAPPPRHGPPSRHPPPPHQPNQPPPPRQQHPQPLPPQQPPRFRVSAPQPPPRPNSSLRPQYPPQHGQPAPYRPQQLANQPARPPSGSPPTNRPKPEPKPQPKPQEPQMQYISGKVLSGWIPAQTSKATPAPTNATFQQPQVQYQHQVPLKQQPYQHSNQAQPQRQQYQSSQQPYQGQRQQQPQYPQQQPAYRQPAPQQHRQPQYQQPQQPPVARQQPNQPQHQQHARPQSTRHSPYQQPYQQPHPQYNAQPAPAQQPQYPPAQPHNPYPDQPSHQPAPYVDPRPIYVPPAPAAPSSPTTTIPPPPPPTCLDGDKHCKHWGVHCKTNPYVHTNCRLTCNTCDIPTPPVHAKPIQTTQKSKAEPTIIPSRPPSPSPGRDHFHQPKPQHRPQPKPRRPPPQPQYNHYATTTAAYYPTTTGYNNQAPTTQTPYNAPGQGTSAAASSVASVGPHVGFCRDFDNRCKEWAKYCGMDEYVDDMCRLTCMRCKK | DNA N6-methyl adenine demethylase-like isoform X4 [Crassostrea virginica] | XP_022319021.1/1e-44 | SCP(SM000198);ShKT(SM000254) |
| **CL563.Contig3** | 25.0163 | AGFAGDDAPR;DSYVGDEAQSK;GYSFTTTAER;HQGVMVGMGQK;QEYDESGPSIVHR;SYELPDGQVITIGNER;TTGIVLDSGDGVTHTVPIYEGYALPHAILR | 7 | MCDDEVAALVVDNGSGMCKAGFAGDDAPRAVFPSIVGRPRHQGVMVGMGQKDSYVGDEAQSKRGILTLKYPIEHGIVTNWDDMEKIWHHTFYNELRVAPEEHPVLLTEAPLNPKANREKMTQIMFETFNAPAMYVAIQAVLSLYASGRTTGIVLDSGDGVTHTVPIYEGYALPHAILRLDLAGRDLTDYLMKILTERGYSFTTTAEREIVRDIKEKLCYVALDFEQEMATAASSSSLEKSYELPDGQVITIGNERFRCPESLFQPSFLGMESAGIHETTYNSIMKCDVDIRKDLYANTVLSGGTTMFPGIADRMQKEITALAPSTMKIKIIAPPERKYSVWIGGSILASLSTFQQMWISKQEYDESGPSIVHRKCF | actin,adductor muscle [Crassostrea virginica] | XP_022325998.1/0.0 | ACTIN(SM000268) |
| **CL2840.Contig2** | 21.3773 | ALVDTETFASPR;ASSYLDDIYYPEPIVR;SLPPPVISLER;TPTPVTTSR;VAVLASPLR;VEVVTPR | 6 | MTVRRSRFQSVPPGYFSSTKGHSSLKRWYPTTTRASSYLDDIYYPEPIVRSRGFYDTTREENEIRRDVNHELLYTSNLVDDTYDIANKSRNRDQMLLREATRALVDTETFASPRSAVTSRRVRQTSVVRTPTPVTTSRAVSCPPVSRGSSQVVVVLTSPAFKGGSRVFSAKSAAMAVRERSLPPPVISLERSKSVHPQLLPSDILEKRINARRIINTVAGPYMLPLYHPYQSIYQPYISMYQPLYARRKYLATLKDVPSRGRVAVLASPLRRKKYRKAALVGNRVEVVTPRKRKPRSTYAANKMRELKRDEREMEVEAVPVQSTTSLKASTVSPNYKGGKLHWDEDGKVTQPTNLMSWQYRIESRVPPGDHLFPVKTIGHVRDKLLHVKEQMDRHRQLMDRYLPDEDSKTDVKTKIMNMYVDMEQHNPAS | RS-rich protein-1 [Mytilus coruscus] | AKS48138.1/0.0 | — |
| **Unigene3051** | 17.1980 | AIQNTQNGMWAAGR;CQMIIGIPALPDLR;FTPLHTLLTR;QMPTWPR;QTGNFFVVDTVPGR | 5 | SIIPPDTKYQDIVVDNLSYSGRPGSGSVLIEPVPVSSSFGTLPDGVVIADSGSPAAGALPDGVIIEPVPEGSNLGGIQIPTGGNVPVLIKDDNLLIKPSPEKTVWTSEPILINDSNLIATTPSKEEYGQNLSGSGKGSTSAIKTTTSTSSVTIQSQEPSAVVTVNNGTKSNGDNEGTTWLVTPTLPPTTKAPVINPNSGTPKPLSCPDFDSRYENGKFIVTLDEGKCEMVVSERAIQNTQNGMWAAGRFTPLHTLLTRKYKPEINFRSTTLAPTGYPCLHKTRQTGNFFVVDTVPGRCQMIIGIPALPDLRNLLTQRQQKVPTWNKPRQMPTWPRRRPSKEREDNESSD | uncharacterized protein LOC111131742 [Crassostrea virginica] | XP_022335121.1/5e-07 | — |
| **Unigene48535** | 16.3160 | GLNSGGVNSGTR;MGGVNSGTR;QINLSNLNAEQLEVIMDQR;TEFFIPR;YDTVNSMMCR | 5 | SLICILLLVKFALIAGQRQLPRTEFFIPRTQQNAKRVQQKFTQLERLKQLNNMSGMKFGNSGASGNGWNNNVGANGGRGLNSGGVNSGTRMGGVNSGTRMGGNRRQINLSNLNAEQLEVIMDQRRMQMEMGMGMGMGMSGGGMGAGNGGYTFINVEALNNAAEMGIMPSMFMGNNGARGQQGMLGNNNNNGKGGMNVGRGNGSRRNGMNLPLCQPLYECAENFIPPQCRTWRYDTVNSMMCRSCPINTCQGGAKEMFNTMYEHHPKRLLFEALTGK | — | — | Signal peptide(1-16) |
| **Unigene39912** | 16.5580 | GGPVIALETLADAPSEPR;INLVEPDYPYIVVVR;LADMEQATGELVR;LEWTSDFLDDDTTHVFR;SLPIEGATQR | 5 | MLSIGLILVSLVSVSHGQWRQDMFSQAENKLRNIVQNGQILLDFIYQERQKHGGGNMTSGSMMSQNLAYSSFINDVEVRLADMEQATGELVRIMRTCPDAPLAPPSPTNVIVESTTTDNVSSIVVKWDPPFNPPENMQYKVYFVPVDINGMQTAGEVVFRICDSTQTIASITDLSPHSRYRIRVGAVAGSIAESTSVPLNVKTPDLIPSRVQNVMVKSSTPNTITLMWNPPSTMGDLVSYEIYYEENPINKMHVTVSPPENTFTIKDLSEGTTYKFEVSAKSDNGEGIRSLPIEGATQRFIPRAPQSFTGVALNKTAVKVTWTPPPPQPGDGIIRGYLINYTDVRYTDVSEHRVGADVFETVITKLTPAQVYYFRAFAFTKKSVGRGGPVIALETLADAPSEPRQLQIMIIREEPPKIGLTWLPPLHTYGNLLNYTLIWGVQNGANRTEYISPTRLEWTSDFLDDDTTHVFRLAAVNKVGLGDAAITTYRTPKKVPIIPPNVKVKRVTFENNGTTILNVTWDNPVVAVDGFRILYRKFQLVYSGRWELVEVFESNKKFVRINLVEPDYPYIVVVRGIPKGQIFNQYNMGGGQHMSRPNSHIAQSFGGASPPI | shell protein-6 [Mytilus coruscus] | AKI87977.1/0.0 | Signal peptide(1-17);FN3(SM000060) |
| **CL955.Contig7** | 12.1509 | DLEESTLQHEAQISSLR;ELEDLGER;ELEGELDSEQR;ESYNLAER;IEELEEELEAER;LQGELEDLGIDVER;NSLQSIIDELR;SSVSISR;SYSAELFR;YQQQVSEVQR | 4 | MSDLKIQVSTSVTSSKTEKPAVVKEEKAPEERIKTPDIISSPPAPEEAKVTTVKEETRRASASTLTPKMSDTSRITKTTRTSSIRTADYESTVGQLTKDYRGTSPAVLEGIASQPVLYSKAFDKIGQQKLSARSRKILRDTTDLALVAPGLKNLLEARIEELEEELEAERAARTKVDKQRAELARELEDLGERLDEAGGATSAQVELNKKREQELLKLRRDLEESTLQHEAQISSLRKKQQDATNELADQVDQLQKAKAKVEKERQQFRSECDDLQAQLQHYSKNKGVSEKMAKQLENQIAELQQKCDEANRNVNDLNSQKAKMQAENSNVVAQLEDTEHQIGSLSKERNSLQSIIDELRQNVEEETRARMKLQSDIRNLNADLDAAKEQIEEEQEGKADLQRQLSKANNEAQQWRSKYENEGANKAEELEEAKRKLQAKLQEAEQNAEAANAKVSSLEKAKNRLQGELEDLGIDVERANANANALEKKQRAFDKTIQEWQAKVSDLQSELENAQKEARSYSAELFRCKAQYEESQDSVDALRRENKNLADEIHELTEQLSEGGRNVHEVEKARRRLEMEKEELQAALEEAESALEQEEAKVMRGQLEISNVRSEIERRLAEKEEEFENTRRNHQRALDSMQASLEAEAKGKAEAMRIKKKLEQDINELEIALDASNRAKAELEKNIKRYQQQVSEVQRQVEEEQRQKEEVRESYNLAERRCNMISGEVEELRTALEQAERARKGAENELFEANDRVNELSAEVQSISSQKRKLDGDIQAMQSDLDEMNNEVRNADDRARRAQEDSARLADEIRNEQEHSQQIEKFRKSLEGQVKDLQVRLEEAESQALKGGKKMIAKLEQRVRELEGELDSEQRRHAETQKNMRKADRRLKEIAFQADEDRKNQESLNSMIDTLNAKLKTYKRQVEEAEEIAAINLAKYRKVQQELEDAEERADSAEGSLQKLRAKNRSSVSISRSSVTHTPSRTLLSTERSDI | catchin protein [Mytilus galloprovincialis] | CAB64664.1/0.0 | Myosin_tail_1(PF01576) |
| **Unigene51075** | 15.1114 | LASDLLEWIR;NINEVENQILTR;QGLEEAER;VGWEQLLTAIAR | 4 | MEDYPSDGYMDEEEEWDREGLLDPAWEKQQKKTFTAWCNSHLRKAGTQIEDIEEDFRNGLKLMLLLEVISGEQLPRPDRGKMRFHKIANVNKALDYIASKGVRLVSIGAEEIVDGNCKMTLGMIWTIILRFAIQDITVEELTAKEGLLLWCQRKTAPYKNVNVQNFHLSWKDGLAFCALIHRHRPELIDYYKLSRENPLENLNTAFNVAEQHLDIPRMLDPEDMVNSAKPDERSVMAYVSSYYHAFSGAQQAETAANRICKVLKVNQENERLMEEYERLASDLLEWIRKTTPWLENRTTDNTLPGTQRKLEEFRDYRRKHKPPKLEDKARLENSFNTLQTRLRLSNRPAYLPTEGKMVSDIANAWKGLELAEKGFEEWLLSELQRLERLDHLAQKFRHRCEIHEEWAEGKEDMLQSQDYLKCRLNELKAMKKKHEAFESDLAAHQDRVEQIAAIAQELNVLHYHDVQSVNTRCQLICDQWDRLGTLTAQRRQGLEEAERILEKIDQLYLDFAKRAAPFNNWLDGAKEDLLDMFIVHSIEEIQDLIEAHEQFKGTLGEADKEYNSIMGLANEVQRLAQQYGLTLKENPYTTVSPQEDIANKWGEVKQLVPKRDRTLHDEKIKQENNERLRRQFAQKSNVVGPWIENQLDGVASIGVTARTSLEEQLNKLRQFEKATESYRVHMDELERYNEEVQESMIFENRYTHYTMETLRVGWEQLLTAIARNINEVENQILTRDSKGISEDQMNEFRVSFNHFDKNRTRRLEPKEFKACLVSLGYNIRDDRQGDADFQRIMSIVDPNNSGYVTFEAFLDFMTRETADTDTAEQVMQSFKILAGDKPFITAQILRQELPPDQAEYCIQRMAPYSGRDAVPGALDYMSFSTALYGESDL | Alpha-actinin, sarcomeric [Crassostrea gigas] | EKC43084.1/0.0 | CH(SM000033);SPEC(SM000150);EFh(SM000054);efhand_Ca_insen(SM001184) |
| **Unigene37646** | 15.1114 | LTWACESPCR;LVCYDQCR;MDGEENEYEGPTSMSNR;RMDGEENEYEGPTSMSNR | 4 | VSFKFLSFFLLLMTVTGQRRRPISKTDDRVSFWYQCKQECIRLTWACESPCRNYAETRMAYKMCALECKLDRLVCYDQCREFIAQGLVWLASPSNNNKRNRRRMDGEENEYEGPTSMSNRKDFMAFLNYILES | P,N-U7 [Pinctada fucata] | AKV63173.1/0.71 | Signal peptide(1-17) |
| **Unigene43460** | 12.6413 | FLQAQVGPSGPR;GPQGDIGPSGER;GTMGEPGPTGPQGER;SVYGGAMTSWFR | 4 | MKFGTVRWKNCIAPLFLFIVLVKSQEEDKANCLYEGTTYYHGDQWKPENCKWCVCDNGVADCKELLDCDGFGQITTGQESNTAIQGHQAIENEAEGSVGSPGRDGSQGLPGPIGDPGVNGKHGIPGPPGPPGVPPMSADQAYNRYFQQTYGQSFKAGGPAMGPRFLQAQVGPSGPRGSPGLPGQPGPQGADGVRGESGDTGPPGNPGLRGAPGAPGPPGLEGDSGRNGETGPRGLSGPKGPTGPAGMPGMPGMKGHRGLQGVQGPSGEQGRPGDKGSSGAPGAPGPNGPEGPRGSQGDRGSDGSAGPAGLPGVDGLAGAAGEPGPVGRTGPPGSPGLPGQKGEAGASGPKGSQGLQGSRGDPGISGPPGAEGMAGSDGLPGSNGEKGASGDPGPAGSPGFQGPRGPSGLNGSPGNAGAKGAPGQPGSPGFKGERGPKGIRGSGGDRGPPGAPGNEGKRGQRGTMGEPGPTGPQGERGSTGMRGYPGPIGDPGAAGEEGGIGPRGRRGEPGPNGVPGRMGPPGARGPRGGNGGPGIDGMAGRPGPPGVTGNDGRPGEMGAPGIPGPAGIQGVQGNPGTRGPPGKDGNPGAQGPRGPQGDIGPSGERGNTGPRGAVGEPGGRGPEGNGGAPGFVGAPGPPGGQGEPGKPGEPGPPGKAGKAGRPGSRGERGIPGVTGEPGAPGLSGVQGPEGGAGRDGERGAPGEPGGLGEPGPEGPAGRQGMRGPRGERGAKGEMGEAGLPGEDGREGRKGPSGPQGPPGEPGPPGEPNEKGSVGDLGLPGERGARGTPGDRGPQGTAGIQGEPGQPGMAGAPGPKGQRGQTGQKGEQGAAGIAGAQGAPGPTGRDGMNGRKGVRGDRGSQGLPGQPGTPGGVGPVGNAGPHGDDGPPGPPGEDGIKGSRGETGHVGRPGESGAPGLPGEPGLKGARGEDGESGGVGQVGPAGPPGEMGVPGDSGIRGERGNAGPPGRPGQPGDPGRAGLNGAPGNAGPPGPPGLAGPSGEVGHPGPPGPDGAPGLQGAQGEKGPDGDIGMPGAMGLMGFSGPPGPPGPAGPAGERGERGEPGPNGAAGQQGARGPPGPQGPQGPSGEKGSLGENGDKGDPGLMGMPGLSGPEGPVGDLGPTGPQGPPGQRGPDGRRGDPGSDGMVGPSGPPGPPGPRGPQGEDGRRGSMGEAGNPGPPGAPGRSVYGGAMTSWFRGSSGNKGWQGDEPVPAEEIDTDVFKALEEVTLQIEKIKNPTGEQDSPGRTCEDLRAHNPDIKDGYYWINPNLGPIYDVIKVRCDFRKKRVFTCVQPEVKTIENMNIAQKNDHTWISEVLGSKFDYDPSLFVKPQIKFLQYLHQKANQEIVYKCKNSVAIDDDKSIQLAGFDNSLLSSKGKRSIRYKIKKDNCKNKNGSWEKTVLEVNTKRTKALPIMDIGVYDIGGADQDFKIELGEVCFFN | fibril-forming collagen alpha chain-like [Crassostrea virginica] | XP_022341069.1/0.0 | Signal peptide(1-24);VWC(SM000214);Collagen(PF01391);Internal repeat 2;COLFI(SM000038) |
| **CL758.Contig1** | 10.0437 | LTGMAFR;VGINGFGR;VPVPDVSVVDLTVR | 3 | MKVGINGFGRIGRLVMRAAIDKGVSVVAVNDPFIDLDYMVYMFKYDSTHGCFNGTVEAKDGKLIINGNAVAVFGERDPANIPWGANGAEYVVESTGVFTTKDKASAHFKGGAKKVVISAPSADSPMFVMGVNEEKYTKDLTVVSNASCTTNCLAPLAKIINDKFGIIEGLMTTVHAITATQKTVDGPSMKDWRGGRGAAQNIIPSSTGAAKAVGKVIPELNGKLTGMAFRVPVPDVSVVDLTVRLQNGASYDNIKKAIKEASEGPMKGIMGYTEDDVVSQDFRGDNRSSIFDAKAGIALSETFVKLVSWYDNEYGYSCRVIDLLKHMSKVDSA | glyceraldehyde-3-phosphate dehydrogenase [Littorina littorea] | AJA37895.1/0.0 | Gp_dh_N(SM000846);Gp_dh_C(PF02800) |
| **Unigene58165** | 9.9049 | CISWYQICDGK;DGSDEDVNLCR;EHTCGPNYVQCDDGLQCIHER | 3 | HVLIVRRALDQKLIIMHRFLQTSLYILVALGFVKAAGVCDSGYEKCGNSDQCIFYGLFCDGEVNCENGADENDAFCREHTCGPNYVQCDDGLQCIHERGYCNGKAMCKDKSDELPQNCQKFQCPDIAVKCASGDECTPWYELCDGESHEIEGCKDGSDEDVNLCRASTCPSGTQKCDNGLQCYHDLYACDGKNDCSDGTDEKPDMCRAFQCKMNRVKCADGLRCISWYQICDGKSNCIDQSDESTAICDGNQSLRRLKSLTVLPSA | EGF-like domain containing protein [Oryctes borbonicus] | KRT86894.1/5e-29 | LDLa(SM000192) |
| **CL3951.Contig2** | 7.4705 | GNDLEAAR;GTFTDGTR;SGPFLVFR | 3 | MKDIVYIAVLFFTLFHVANSLCSHPCSRVNRGTFTDGTRNFVFGCTNTSVLQVYEGNRFIEDRECYARSGPFLVFRVGSRYQCFKDTVVDPNTNVVMIYFAPTQTFSTNPSICDVCAGEYTFALFVPRGNDLEAARRLPRPPLGCNRPPNCPILPDPYYIPCTGCEPKEDDGLCCSSCQDINNVYNRYGRGNQDRRNRDRRGRSNRFIGSLTQSFNNGRYSRPTKRSAKTC | hypothetical protein [Gracilimonas sp. 8A47] | WP_109647365.1/3.3 | Signal peptide(1-20) |
| **Unigene8686** | 11.3335 | RLGLDDFGDDNGDDN;SDGEYGSNGAGYR;WDSDQDFR | 3 | SGTLYGSCHSKTLDMRKLAVVSILLFLGLPSNIICRNDVKEKDGNRGNRWDDMSDSWGFSEDSSDGGWGSNSVKWDSDSEGWDSDNFGSDRRSPRGRGGNGNNRLDSDSGWYTSEYFSDESDNRVGIRGDNGNKLRNIVGLESEGIDWDSDNSDSSNGAVKKAGNGNSGFGSNGIDRNSETSDSSDGVSKNAGNGNESNGRGRKGNINIGGHSGGRFDSDDSDWGSDRWDSDQDFRRGRSNGNKENNGGGDSNGGGRRWGDSDRRDSDSWDSDDNNIWDGSDGWERERSDGEYGSNGAGYRGSDDSDWFESDNRFDSAESFERLIKRLRRPNDGDSDGNSPPGVKFLGNNGVKRADFWDDSDDLWNDYAIGRRRHRPRRRRISAKRLGLDDFGDDNGDDN | — | — | Internal repeat 1 |
| **Unigene5251** | 9.8017 | AVFVDLEPTVVDEVR;EIVDLVLDR;NLDIERPTYTNLNR | 3 | MRECISVHVGQAGVQMGNACWELYCLEHGIQPDGQMPSDKTIGGGDDSFNTFFSETGAGKHVPRAVFVDLEPTVVDEVRTGTYRQLFHPEQLITGKEDAANNYARGHYTIGKEIVDLVLDRIRKLADQCTGLQGFLIFHSFGGGTGSGFTSLLMERLSVDYGKKSKLEFAIYPAPQVSTAVVEPYNSILTTHTTLEHSDCAFMVDNEAIYDICRRNLDIERPTYTNLNRLIAQIVSSITASLRFDGALNVDLTEFQTNLVPYPRIHFPLATYAPVISAEKAYHEQLSVAEITNATFEPANQLVKCDPRHGKYMACCMLYRGDVVPKDVNAAIATIKTKRTIQFVDWCPTGFKVGINYQPPTVVPGGDLAKVQRAVCMLSNTTAIAEAWARLDHKFDLMYAKRAFVHWYVGEGMEEGEFSEAREDLAALEKDYEEVGVDSVEGEGEEEGDEY | tubulin alpha-1A chain [Mizuhopecten yessoensis] | XP_021370666.1/0.0 | Tubulin(SM000864);Tubulin_C(SM000865) |
| **Unigene30013** | 10.2922 | GDIASGIGGGAIGGR;GTTGFAGAVSGR;TGAVSNFPVFGPGINR | 3 | GRTGAVSNFPVFGPGINRGFGSSFDGNFGAGFGIGPIGGGFPSFGGPASLASLNAALSGSINAATDGFPGFVGGPLGTLSGSLSGGLNAATGGFPGSIGGPGPLGTLTGALSGGLSAATTGLNAGLNAGLSAALRGDIASGIGGGAIGGRGTTGFAGAVSGRFAGTVGAGAGGAIGKGKVY | glycine-rich cell wall structural protein-like [Crassostrea virginica] | XP_022339053.1/4.9 | — |
| **Unigene20161** | 10.0437 | GMTGFGAIR;GMTSFGSQR;NLPMVLATISHVGTEAQR | 3 | MADRVKPMGMDRALISKMGAKYDPAVESEVRGWINQLIGEDIGEGPSNLEKGLRDGVILCNLMKKIIDGTPSESLPPACNKLNLTPSPSELPFKQMENIEKFLKAAHAYGVPNTSLFQTVELYEARNLPMVLATISHVGTEAQRHNYNGPTIGSKPTEKHRVQFTYEQLKNSHGTIGLQSGTNKFASQKGMRIGAIRHISDIRADDLDKEGTTLLTLQAGTNRFASQKGMTGFGAVRHIADIRADDADKAGDNIITLQAGTNKFASQRGMTGFGAIRHVSDIKADEFDPNTQSHIGLQAGSNQFASQKGMTGFGAVRHICDIRADDLDREAQAEIPLQYGTNKGASQRGMTSFGSQRHIADIKVSDLAEDMKRQDLDMTPKEYQQYRRELEEAAKQQGQEVEEPQYE | calponin-like protein-1 [Mytilus coruscus] | AKS48134.1/0.0 | CH(SM000033);Calponin(PF00402) |
| **Unigene12260** | 10.2922 | MTEHGLAR;QNGLGGGNPLICSR;RQNGLGGGNPLICSR | 3 | MDPYLFLCVLAIAFSVHNVYAQMGMGHQNQGNMRRKFPGRQAGMGGGMGGGMGTGNNNQQGMNLGGSPADMGMPGNGMGGQGQGQGHGNGGQGQGNGMNNMGGGGGSGGLMGGMLGGGMPMDAMMGGMQANMMAMGAMNSEIPPHMIMSGTFNPKNFAQYRSCEKTPSNLNTICDPSSPNPCPQGAMISKSTPFAMGMGMAMGGMGGMGMGGMNRRMNRRQNGLGGGNPLICSRMTEHGLARCCAKNMMTARMLDKWFK | — | — | Signal peptide(1-21) |
| **Unigene58102** | 9.8017 | EPENPPMENALEPAR;EPLTVDPYGDALTSGRPEVAHPEFISSR;VRPSTAEFIEQGPLFEHDGTQR | 3 | MKETVLCIVFLQLALVFGAPKSTQTNKVVPKDRVLQGAVEQLFIPDGNSLFEIDIVQGYNPEVIQHVRRKRQAEEKSLERKSSSKKRRRKKKKRRRSEKKPGAVTTLREITPTEKIMLPPVEREPENPPMENALEPARKSRRTFRPPPPTDPAPTLPALVLSEMENNNDDNSGLLKVKSLKSVFINTDNMRTNKLMADRIKADAIHAKDVVVTSKKKKSGSRSGGRKRRRRVRPSTAEFIEQGPLFEHDGTQRVNSILEQPQYIPETILTKNPYGLGDTIYQPEKPRRRFRVNERHLYREPLTVDPYGDALTSGRPEVAHPEFISSRVRSKVPLLSEFIEGTSGNSKPSTTFVNRRRPFAKQRPIEEFHIFDNPINTERQNPVAKRVDKTSKRFTKNVDPWFL | KS-rich protein [Mytilus coruscus] | AKS48160.1/5e-14 | Signal peptide(1-18) |
| **Unigene483** | 11.3335 | AAAAAAASASASAGSGIGVASR;AALVQLVIK;LYAYDYYK | 3 | GFGGGFGGGAGAGAGAGAGAGAGAGAGAGASAAAAAAAAASARRAALVQLVIKARAAAQARAAAAAAASASASAGSGIGVASRFGGGFGGGAGAGAGAGAGAGAGAGAGGAGGASAAAAAAAAAAAAARNANLRGWQSANANSLAAAIAAASAGGGGGAGAGAGAGAGAGAGGGAGGGAGGGAGGGSGGSGGSGGSGGSGGSGGSGGSGGSGSAVRLYAYDYYKNSDDKKGPGYERS | — | — | SCOP(d1gkub1) |
| **Unigene40545** | 10.2922 | EVGVHLVNVYR;LANGHLGISFTPR;VVAPSGVEEEAIVQEIDDGQYAVR | 3 | ATYYPKDEGKAKVDVKYAGQNVPGSPFPVEVFPGVDASKVLVSGPGVGKNVYASMPATFTIDTRNAGNAPLDVVVQRPDGSFIKPLVQDNGDGTYTVQYVPDDLGTYVLRVKFAGKEVPNSPFKVTSHPTGDASKCVITEGLENKTVQVNKETVICVDASQAGDGKVTCRIRSPQGSDIDIDIVENADGTFSLLFTPQIEGAYTISIKFGGQTVPGGEYDIQTTGYTAVNEDLISADTVDSVKGAAPGSGLFQPVDFCIPVGPIFNFVSAYIVMPSGKKAYPKIEDNKDGTVTIRYQPTETGLHELHVNYNNEEIEGSPFKFHVDAVNSGHVTAYGPGLSHGIVNEPAYFTIVTKDAGAGGLSLSIEGPSKTEIKCNDNGDGTCTVSYIPTAPGEYNITVKFAGQHISGSPFTSKITSPPGEIKRKSQFGRSSEFELKVVEEDINNLMATIRTPSGVEEPCLLKRLANGHLGISFTPREVGVHLVNVYRNGHHIQNSPFQITVGESELGNASKVKVYGPGLEQGNANELNEFTVDTKDAGYGGLSLSIEGPSKADIECQDNEDGACRVTYKPTEPGNYIVNVKFADEHVPGSPFNVKVAGEPSPKLTERITRHREAADVMHIGSQCELSLKIPASFFMKMMTHEQIEAILVQQQEGTSPFDMTASVTNPSGVTELCDIVSLDDNHYSIKFVPKEMGVHTVSVKHKDMHIPGSPFEFTVGPIAGGGSHKVHAAGPGLERGEINQPCDFNIYTREAGAGGLSIAVEGPSKAELDFDDRKDGSCGVTYKVAEPGEYLVSIKFNDEHIPDSPFRVNVSPSIGDAKKLSVSALQSKGLQIGKPAAFVVNFNGAQKGKLKARVVAPSGVEEEAIVQEIDDGQYAVRFIPRENGGHNVHVFFNDCEIPESPFRIMVGKVDCDPGMVHASGDGLRTGQSGQPAKFFVNTVNAGPGALGVTVEGPSKVKLECTEKEEGYEFTYYPTAPGDYLITIRYAGVHIAGSPFKARIEGQAGPSDVIQHGMSQVVVETVTKTSVMSKFQAIPQFKSDASRVTCEGNGLKKAFRGKQATFNVDTSNAGNNMMFVGMMGPKGPCEELCVHHKGGYQYKINYVVKERGDYMLIVKWGEEQIPGSPFCVHVE | filamin-like protein-1 [Mytilus coruscus] | AKS48135.1/0.0 | IG_FLMN(SM000557) |
| **Unigene25503** | 11.3335 | GSPFMMIDNR;TNPWNENSLYR;YSTQYQQTPNQQHTTMSGFR | 3 | GESDNNEENEESSTQEKQKMQYYGNNDENRLLDDSGIESDDSAQSDGKNDKFDYSDRESFEKEYNSLSSEEDHSEMANNDLNKDYDSDEDSYQHNEIKLNVKTSSSSLNWGYNQPTPTPSSVRTNPWNENSLYRSNVDYDDKDVNVEGDSVNAFKKIVPGFTEKTPSYNYMEIQNNKLQVNTFGSDTNDYSESENEKENEGDDVGDGDENKKWNKFSEEKEDKQQNEHWSAGAVPDIYAGTVIVTSRKRGSRIDNTGKEKENENEIENEDKISSKNSKLLGVKKGKKVSSWGGQEGNSAEKAENEVVEKSPDAENEKPEKGSLDKQKEDENSNNLIGLTTIYQEEGDGKSANEENELKQSVKENSKKSLKEENEMKTLKEENEIKSHKEENEMKSPEEEIKIKTPKEENSFGEMKVDLAGHIGDSNIVKPEEDIGESKSNSVFMNNQQFRNVDDPSEPVKSASNSMGVKPFIGDSPKFMGNMKQAQMFYPTAFPAMNSFTTRGSPFMMIDNRYSTQYQQTPNQQHTTMSGFRKRVQNRISKSMGHNIDDPVMTQPLTLKVKIKSTRKSKKGLSPNLSKLVLKMFMNSKSGAPPCDGNLKSVCRPVKMWSKYQQVADWCTSLCPYGQCPSAVCKCQCTGSPMGSVGQKKCRATNTYKQNSGELDQWCQKTCSKGDCPALLCVCS | Pinctada martensii chitin binding protein mRNA, complete cds | KJ930034.2/0.039 | Internal repeat 1 |
| **Unigene54043** | 6.7850 | FASFIDK;FLEQQNK;YEDEINKR | 3 | RTQEKEQIKTLNNRFASFIDKVRFLEQQNKMLETKWNLLQGQTTTRSNIDAMFEAYIANLRRQLDSLGNDKMKLEADLHNMQGLVEDFKNKYEDEINKR | keratin 8 [Epinephelus coioides] | ACH73075.1/5e-58 | Filament(PF00038) |
| **Unigene18932** | 7.5557 | IINEPTAAAIAYGLDK;TTPSYVAFTDTER | 2 | LFGYKIAAASRGIHLKEKKRAYKNKKEQSPQNRNMAKAPAVGIDLGTTYSCVGVFQHGKVEIIANDQGNRTTPSYVAFTDTERLIGDAAKNQVAMNPVNTVFDAKRLIGRKFDDASVQSDMKHWPFTVINDSSKPKIRVEYKGEQKTFFPEEISSMVLVKMKETAESYLGKTITNSVVTVPAYFNDSQRQATKDAGTISGMNVLRIINEPTAAAIAYGLDKKATGERNVLIFDLGGGTFDVSILTIEDGIFEVKSTSGDTHLGGEDFDNRMVNHFIQEFKRKHKKDISENKRAVRRLRTACERAKRTLSSSTQASVEIDSLFEGIDFYTSITRARFEELNADLFRGTLEPVEKSLRDAKMDKASIHDIVLVGGSTRIPKIQKLLQDFFNGKDLNKSINPDEAVAYGAAVQAAILSGDKSEEVQDLLLLDVAPLSLGIETAGGVMTSLIKRNTTIPTKQTQTFTTYSDNQPGVLIQVYEGERAMTKDNNLLGKFELTGIPPAPRGVPQIEVTFDIDANGILNVSAVDKSTGKENKITITNDKGRLSKEEIERMVNDAEKYKDEDEKQKDRIGAKNSLESYAFNMKSTVEDEKLKDKISEDDKKVIMDKCDEIIKWLDANTLAEKEEFEDKQKELEKTCNPIITKLYQAAGGAPGGAGGMPGGMPNFGGAGGPTGGAGSGGSGGPTIEEVD | heat shock protein 71 [Perna viridis] | ABJ98722.1/0.0 | MreB_Mbl(PF06723) |
| **Unigene6274** | 7.5557 | ELTDQLSEGGR;IEELEEELEAER;LAQQLEEAR;SYSAELFR | 2 | LQQFFNHHMFVLEQEEYKKEGIQWEFINFGMDLQACIDLIEKPMGILSILEEECMFPKASDKSFKEKLFTTHMGKSPNFNKPGKASKGKKSDFELTHYAGIVPYGTEGWLEKNKDPINETVVDLLSKSKEHLVQTLFAPPAPVEGGGSKKKKSSAFQTISAVHRESLNKLMKNLYSTHPHFVRCIIPNELKQPGLIDAFLVLNQLQCNGVLEGIRICRKGFPSRIVYSEFKQRYSILAPNAIPQGFVDGKVVTDKVLTALQLDPAEYRLGNTKVFFKAGVVGNLENMRDERLSAIISMMQAHIRAYLIRKSYKKLCDQRIGLSVIQRNIRKWLVLKNWQWWKLYSKVKPLLNIARQEEEMQKKLEQLKKLEEDLAKCEKIKKELEVQNVTLLEQKNDLFLQLQTEQDNVIDLEQRVEQLVKQKADFESQIKELEERLLDEEDAASELENIKKKMEGENDELKKDIEDLESSLAKAEQEKTTKDNQIKTLQDEMAQQDEMIAKLNKDKKGMDEAHKKTLEDLQKEEDKVNHLNKVKQKLEQTLDELEDGLEREKKVRSDVEKAKRKVEQDLKATQETVEDLERVKRDLEEANRKKDAEINSLNSRLEDESSLVAQLQRKIKELNARIEELEEELEAERAARTKVEKQRAEISRELDDLSDRLDEAGGATQAQLDLNKKREQELVKMRRDMEETILQHEAQVSTLRKKQADAANEMADQIDQLQKVRNKLEKEKKDMKREMDDMQATFQHQLKNRGASDKVVKQFESQIADLNAELEKSQRNLSDMVNNKTKFEREAAELSQQLEEAEHNVGSFSKEKSRLAQQLEEARSALEDETRVRQKLQSEIRNLTGDLDAAREQVEEEQEGRSDLQRQLNKANTEAQTWRSKYETEGAARAEELEDSKRKLQAKLAEAEQNADAANAKVSQLEKAKNRLQGELEDLAIETERATANANAMEKKQRGFDKTVAEWKSKVNDLQLELEAAQKEARSYSAELFRVKAQVEESQDSVEALRRENKNLAEEIRELTDQLSEGGRSVHEVEKAKRRLEMEKEELQAALEEAESTLEQEEAKVVRAQLEISTIRNDIDRRLHEKDEEFENTRRNHQRALDSMNASLEAEAKGKAEAMRIKKKLEQDINELEVALDASNRAKAELE | myosin heavy chain [Mytilus galloprovincialis] | CAB64662.1/0.0 | MYSc(SM000242);IQ(SM000015);Internal repeat 1;Myosin_tail_1(PF01576) |
| **CL4671.Contig1** | 7.5557 | GPGGPVAVPQR;GPVLLNIDTVDAASGQR | 2 | MLPVVLLISLFAAGTLGTGYVPPPKLPKKVVSLPIPKPVLKGKPLRQDTAFDHHQIVPIKKTEVRRVVEKVPLVHEVPIFITKNKPFYQPQVVPKPIVVHEVVGLPIVRDKHVVHPVYIDRPYIVEKKQFVEQPFPVEKPIPIDFIKRITKVYDRKVEVPHIVNIPLVKTVDRPRAVPYTREVVQHYDVHVNVPRPRPVKVLRHKTHVEKVPFETPNLIVRKNPVHHAVNEYIPKVPQGFNGRVFAGTQEDAAVTAEASRFGPGSGGRTISVSRGPGGPVAVPQRGPVLLNIDTVDAASGQRIPDGAVVARGARSGPASVAGVSAPRQGGAPVLLGQDLSGPGVGSLGPAIPGGPGPVGVEAVAVGGPGPVGGPGPLVGGGIGGAAVVVGGGGGGIGGGAGVVAVGGGGGGVDLGAGPVVVDGGRGGGAGPIVVSGGGGGGGVDGGFEIVDVGANGEGIINIGPGGLGQDIEIVAVGPDGKDIGSFVVGAGDIAGGTGKKGKK | valine-rich protein-like isoform X4 [Crassostrea virginica] | XP_022307204.1/3e-09 | Signal peptide(1-17) |
| **Unigene52025** | 6.5143 | FAESTSALFR;TINSFMEPFQR | 2 | DLQSYLDDLPMGTGLTGRNMHNNNFNGQDLQSYLDDLPPGQRMNGGGLRQFPDHVYENKFKFNRMGGHQSQFGTQNHKVVGDTMKATTRTINSFMEPFQRIRSMNNMNSNQFLGLSKATDLGSNINRITNHRESPEPTSFHHQIRPHATQMSKQRFAESTSALFREPKSANSRFPKHSTLNNVISQTVITEPSLSPMGLGAFLTKQNPIQKLLSPSKPSTTSTLQDNSATKTSVDNQSKLPIQKSLSAVSNTFRMGNNNQVKEFSPKTAKFQNKHI | cyclin-dependent kinase 1 isoform X2 [Trichechus manatus latirostris] | XP_023595093.1/1.1 | — |
| **Unigene35808** | 5.8645 | INQELEGSR;SFTLADR | 2 | MDPHLCTHIIYSFAKLNGNRLAPFEWNDESTEWMKGMYEKFNSIKQQNPRIKTLLAIGGWNMGSEPFTHMVKTTQSRQEFVKSAVDFLRQRNFDGLDLDWEYPANRGSPPKDKHRFTMLVQQLREAFDRDALTTGRSRLLITAAVAAGKKNIDSGYDVPALGRLLDFISIMTYDLHGSWESNTGHNSPLFARSGETGEQRYLNLDWAANYWNRMGVPKSKLNIGLGLYGRSFTLADRNVNNVGAVASGKGKAGKFTREGGFLSYYEVCEMMKSGGKKYYINEQKVPYLVKDDQWVGYDDVDSLSIKVQYVKQQRFAGIMVWALDLDDFKGSCGQGRYPLLKRINQELEGSRYQPDYSIMNAPSIPDILNQPLAPVAPPPRRRKQKPVRKPPVQTPILPALPPAHHTPLLPPVPRVDQHRTSSKDFTCRKGYDGYFASPDSCSKYYMCTDGTAFKFNCAPGLKFNKEHNFCDWPEKVKCTESTKKSSKKNKNRVQQALLPPNPPIRQPEPPRYEPPPPPQPPPTSANGWNFNANAHAATLPPPVPSSPTNSQQSSAWDWVSMIDNPMPFFMSLFGSNDLFADMCANKANGIYPQRDNCRGFIECSEGVSFKGACGPGLAFNPSQQTCDYTHNVPGCK | chitinase-3 [Hyriopsis cumingii] | AFO53261.1/0.0 | Glyco_18(SM000636);ChtBD2(SM000494); |
| **Unigene57834** | 7.5557 | FAVIITDGSSR;VGMMTFGTNPR | 2 | QSSLVVDCFTMGYFALLVLVAVSVVECRNYHSTHVRCEKPVDLAFVIDISSSIWYKHFRREISFIHDIVNLLDVGDRPTQSRVAAVSFSNRLKPEFGLGQYSTKEGVLNAINNIAYEGGDATRTYLGLEYVHNTIFAPGNGERSNVANVVVVLTDGVTNPGSYDNFTRTEAKQKTQIHAQNIRDIVRAQIYAIGIGNEVDKNEIKGIANKPSEQFTLFVDTFTELDTDAVKKAVLTKVCDSLPQREQECSTSKADIFFVVDESSSLMWDANFRKELKFVGSVIDQFELGKDLVRVGMMTFGTNPRMLFYLNDFKTKTEIESLLKITPWHGGNTYLDKAIESLMTYGLNPGFGSRSDVPQIAVIITDGKSTHPTETEKQIAIMKRMNYVVFAIGVGPNKDPIELHKIASHPSNVFEVDNLDGLVAIRQQLLSQLCPGDQPKPPPVNNCQNSMADLIFVADSSTSIGLTAYNEFKTFAKSVVEKFTVGPKNIQIGLITFSNDAHYEFSLNEYRTKEEVTKAIERVPYSTGNTNTHKALEILIKQGFSYINGGRGTSVPRFAVIITDGSSRQPEMTKQLARKAKDQGIILFSIGVGPYITQTELDGMASSPTSMYSFKVDNYAALTRIEQSLVKRTCEEATRQRQFS | Collagen alpha-5(VI) chain [Mizuhopecten yessoensis] | OWF49639.1/1e-152 | Signal peptide(1-27);VWA(SM000327) |
| **Unigene37757** | 7.5557 | EGGIPSYTTEYR;YGTDITVPNR | 2 | MEYERKVYRKETTREGGIPSYTTEYRIGTDRPRYGTDITVPNRYTTTTYKTTGPIVYSSYSSRPATEYTTYTEPSVEDTRVRKEWDETFKRVAPRADDWSLSDVISKRMVLVKDDEWDPYETSFPEERKRFGKPSPITSDVSGRKTFLVEYQIGDFRPEEVEIKTIGNTLKIHAKNSDSGSMKREYSREISIPQEVNPDLISAKLNRTGRLSIEAPIFNTSHKTKIDRRIPVLRN | heat shock protein beta-1-like isoform X1 [Folsomia candida] | XP_021944507.1/1e-12 | HSP20(PF00011) |
| **Unigene51161** | 5.8645 | DHDLSHISSEQLR;GLVVEAVDEQGR | 2 | MEQISFFCFRCLTLSVLFKLYFCNPARVDLRDHDLSHISSEQLRRVVQQNENQDITTDDDNLLEKLYNQTQYHPTNDESRCISCTIREDQKKHRIESIKNRISHALRIDVLGKPNMTNTKLPKIPQFQKLKERYEIREALDMQNDQYRGEREDYEDEFGQSHRTFTFAQNPPEELGIQQPNAIYFDMPDQTDRSLQKATLWVYVTPSDQQHVTEIYLYTLVKRSKSSDTLIKQFLYRKKRTSRGWQQFNLLNEVEKWTEDPSYNRGLVVEAVDEQGRNVVVMPSSDDNGYQPVLETRTSPHHQHSRNKRSIYLDCSEQRATEACCRYPLTVDFVEFGWDFIIAPLTYSAYYCAGECRNQHMDSGAHSYLQQQVGNVPTEHGPCCSPTRMGHLSMLYFDHSMQIQFTTLPRMKVERCGCA | myostatin [Mytilus chilensis] | AGU13048.1/0.0 | Signal peptide((1-23);TGFb_propeptide(PF00688);TGFB(SM000204) |
| **Unigene15208** | 7.5557 | DADEDGDGLLSR;TDINGENDVK | 2 | MRLTLGILCVVISSVVAPPPLDVELRAEKEYLEQLKQQQEDILDTLGDNLIPDSEEEKAIEDTDAEDPIVLKKDEAEFVPISFPSKFDKYDENGDDFIDEGELITVIGVSENIALALKDADEDGDGLLSRDEFENGPWDLDATDLEDVEAMDDLMDENEDLIDKEFIDYDSESFNDILEGDEGMEDEDNEIVDDIEDMKEDFEEDRHERTDINGENDVKEKVEEVVNDLQENIKDVRENVKDINQNVFEKIGDSKK | uncharacterized protein LOC110443432 [Mizuhopecten yessoensis] | XP_021343328.1/1e-09 | Signal peptide(1-17);Efh(SM000054) |
| **CL474.Contig2** | 6.1270 | LTIVTDCIR;YGRPNPGVCPVPR | 2 | VGEDKICCPAGKCHVKDCVYPPRYGRPNPGVCPVPRALTPLQCSRLTIVTDCIRDTDCRDGKKCCETGCDVLKCLTPMINRPECPSVKCTQYTDLCKSDKDCKYGEVCCYGENSDAYCKKCVKRDTTNGHSRRKHSSAPVPMCLTCCGPPPCCGPCISCGAGQNSSHMFCNL | Tetratricopeptide repeat protein 8 [Trichinella sp. T6] | KRX73298.1/2e-06 | WAP(SM000217) |
| **Unigene15131** | 4.8372 | LLVAENMIVAK;LPDAVLNVVR | 2 | NRELCAMFKTILILCIIGSSLGQPGGWQDYHGKLPDAVLNVVRKRLLVAENMIVAKMETRNVKSQVVAGVNYKFDVKVTGVSGRIVDCHFLVYVNLSGIAKVKSHKC | oxoglutarate dehydrogenase [Halioglobus sp. HI00S01] | KZX60278.1/0.019 | Signal peptide(1-22);SCOP(d1stfi_) |
| **CL678.Contig2** | 6.0239 | AAAAAGAAAAAGAGAGAGGSSGLSAALR;VVIQLLTR | 2 | GGLGGGLGGGLGGGLGGGLGGGLGGGADAELELFEDLLGTYGLDIFEGEEGLAALSLLGGLGAGAGAGAGAGAGLGLGGAGAAAAAAAAAAAAAGAGAGAGAGAGAGAGAGAGAGAGAGAGFGGAGGSAAAAAAAAAAAASARSRAAAAAGAAAAAGAGAGAGGSSGLSAALRSRLLARIAARRAAASAAAAASAAAAGGAGGAGGAGAGAGAGAGAGAGGGAGSGAGAGAGAGAGAGAGAGAGAGAGAGAGSGGARGILGWLLRRRAMARAAAAAAAGAGSGGSGGSGGNGGGSDGDCGDSDSDSGSDSNGDNDTDSSDSEGSDGSDSDSGSDPDGDGDSDSSGSSNSSDDNGDSGDYDSGDDGDGDDGGDFKAVAKVVIQLLTRVLSSGVLTAGASAGASASAGAGAGAGAGLGGGFGAGGGAGAGAGAGAGAGFGSGIGLGFGGGFGGGFGGGAGAGAGAGAGAGAGAGAGAGAGAGAGAGAGAGAGAGA | — | — | SCOP(d1gkub1) |
| **Unigene40702** | 5.0857 | LPSYPGYNPR;NPFFTINMYYFNAQTGR | 2 | WKLFCESNTELRMIRFLIILLVPVTAAFLFSSLNFNNVNQNLDLRKLLTYRQLPHRCRLPSYPGYNPRNPFFTINMYYFNAQTGRCETFGYSGRGGNRNRYRSPVECLSRCACHMPVDPGTCHNSTTGITRYYYNKVFKMCASFQFNGCEGNDNNFADFMSCQLACGRSGGGGEIEL | nacre protease inhibitor-like protein 1 [Mytilus galloprovincialis] | AKQ70858.1/2e-47 | KU(SM000131) |
| **Unigene32111** | 6.5143 | IEDAQVPSR;IEDTEVQSR | 2 | SLLGKLKEMKKRIEDAQVPSRDTKEMERKTEDLEKSVFGKLKEMKERFEEVQVPSHDVKEMERKIEEIEQYLFDNLNDLKKRIEDTEVQSRGDVKEMKRRTDDLEISLLGKLKEMKKRIEDAQVPSRDTKEMERKTEDLEKSVFGKLKEMKERFEEVQVPSHDVKEMERKIEEIEQYLFDNLNDLKKRIEDTEVQSRGDVKEMKRRTDDLE | putative kinesin [Trypanosoma vivax Y486] | CCC47259.1/ 1e-05 | Internal repeat 1 |
| **Unigene56883** | 2.3492 | AGLQFPVGR | 1 | GKDSGKTKTKAISRSQRAGLQFPVGRIHRHLKSRTTSHGRVGATAAVYSAAILEYLTAEVLELAGNASKDLKVKRITPRHLQLAIRGDEELDSLIKATIAGGGVIPHIHKSLIGKKGQQKTV | unnamed protein product [Oncorhynchus mykiss] | CDQ62413.1/9e-81 | H2A(SM000414) |
| **Unigene29076** | 3.7778 | AKSMTQVPPGAVLISPNDVVVMMEK | 1 | MADRGSYYDGDVIGKTRPFFRAKSMTQVPPGAVLISPNDVVVMMEKKIKDYPNKMSIWPLYGHMYINGGAGLITAATFTTQFRRMFKIKGNLALGSTYGFSCLGGCLLPIMLHNFLVHENILKGYIGCRLCLTTRSASIQALSGCLYPMLWSGAVCILKAREFHTYIIPNATQGSLIVQMIKRTSSIGLTASVLMMANFMLGMFLCEKELESYETHFGRNFDPERNEELEFYTDMNTGNDLNR | conserved hypothetical protein [Culex quinquefasciatus] | XP_001850901.1 /1e-13 | DUF1370(PF07114) |
| **CL955.Contig1** | 3.7778 | ELEGELDSEQR;ESYNLAER;SSVSISR;VGLSVIQR;YQQQVSEVQR | 1 | TASVLHMGEMKFKQRGEQAEPDGTAEAEKVSFLLGVNSNDFVKCLVKPKIKVGTEVVAQGRNKAQVMNSISAMAKSLYDRLFAWLVKRVNHSLDTKAKRNYYIGVLDIAGFEIFDFNTFEQLCINYTNERLQQFFNHHMFVLEQEEYKKEGIQWEFINFGMDLQACIDLIEKPMGILSILEEQCMFPKADDKSFKEMLFTNHMGKSPNFTKPGKAAKGKNGDFELHHYAGSVPYNIAGWLDKNKDPINETVVELLQGSKEHLVVTLFAPPEGAEATGGTKKKKKSSAFQTISAVHRESLNKLMKNLYSTHPHFVRCIIPNEMKQPGVIDAELVLNQLQCNGVLEGIRICRKGYPSRIIYAEFKQRYSILAPNAVPQGFVDGKVVTEKVLLALQLDPAEYKLGNTKVFFKAGVLGNLENMRDERLGAIVSMFQAHIRGYLIRKAYKKLQDQRVGLSVIQRNIRKWLLLRNWQWWKLFAKVKPLLNYAREEEEMQKKMEMMKKMEEDLAKTEKIKKELEIKNVELLEQKNDLFLQLQTQEDTVIDLEERVQQLVNQKCEFEAQMKEMEERLLDEEDAAAELENVKKKMEGENSELKRDIEDLETTLAKAEQDKTTKDNQIKTLQGEMAQQDEQIGKLNKEKKNMEELQKKTLEDLQKEEDKVNHLNKVKQKLEQTLDEMEDSLEREKKIRGDVDKAKRKVEQDLKATQELVEDLESNKRELEEANRKKDSEMSMLNSRIEDCEGVNAQQNRKIKDLMATIEELEEELEAERAARAKAEKQRAELARELDEISSQLEEQGGATQAQVDLNKKREQELVRLRREMEEMTLQNESQVSQIRKKAQDQANELADQIDGLNKLKSKLEKEKKDLKRELDDVQSQVQYSMKNKGVSDKVAKQMEVQISEMNSRVEESQRTIVDINSLKTKLQSEVADLNRQLEDAEHNIGSLTKDKTSLNHQLEESKRSLEDETRTRQKLQNEIRNLNADVDSIREAFEEEQESKSDLQRQLSRAKNEAQQWRSRFETEGTAKADELEEAKRKLAARLAEAEQNAEAANAKASGLEKAKNRLQGELDDLLVEIERSNVSSSTLEKKQRQFDRTIQEWTTKVKELQTEVDTAQAEARGYSAELFRSKAQYEECNSTIESLRRENKNLADEIRELTDQLSDGGRNAHEVEKAKRRLEMEKEELQAALEEAESALEQEEAKVMRGQLEISNVRSEIERRLAEKEEEFENTRRNHQRALDSMQASLEAEAKGKAEAMRIKKKLEQDINELEIALDASNRAKAELEKNIKRYQQQVSEVQRQVEEEQRQKEEVRESYNLAERRCNMISGEVEELRTALEQAERARKGAENELFEANDRVNELSAEVQSISSQKRKLDGDIQAMQSDLDEMNNEVRNADDRARRAQEDSARLADEIRNEQEHSQQIEKFRKSLEGQVKDLQVRLEEAESQALKGGKKMIAKLEQRVRELEGELDSEQRRHAETQKNMRKADRRLKEIAFQADEDRKNQESLNSMIDTLNAKLKTYKRQVEEAEEIAAINLAKYRKVQQELEDAEERADSAEGSLQKLRAKNRSSVSISRSSVTHTPATSPSVLNSSNLLSPRSMSRGPDSSFLSPRSASRGPGLYRRSVTPSYEDDDY | pedal retractor muscle myosin heavy chain [Mytilus galloprovincialis] | CAB64663.1 /0.0 | MYSc(SM000242);IQ(SM000015);Myosin_tail_1(PF01576) |
| **CL2370.Contig1** | 2.1572 | SLNGDGQRMNVNAEMNLGVQDNATQALHVMNVK | 1 | MLYINCRFQSVNVAKRNTIQFLKSLNGDGQRMNVNAEMNLGVQDNATQALHVMNVKRLVFQSVLNHSGGARDENQDEGTVVMRPTVQDTLKYQFTKSIPTECTTTVLQTRDLRDQMLKICHNNSKN | DNA polymerase III subunit beta [Mycoplasma elephantis] | WP_027333982.1/4.7 | — |
| **CL1827.Contig4** | 2.4880 | VTVPVLWDK | 1 | SIKCKHMFQFLTLIASYIYIYIFAWNCLASCSWKYKMSKAFDSVIGIKGEFKRAQSAFRSFITADGSSGFPAEANRYHLYVSLACPWAHRTLIVRKLKGLEDVISTTVVDWNMGDRGWRFTDKVERCSLDTLNGKDYLREVYQIIDPNYQGRVTVPVLWDKKQSTIVNNESSEIIRMLNKEFNAFCKTKEQAVLDFYPEKLRSKIDDINSWIYNDINNGVYKSGFARSQEAYDAAVTALFSALERVEEILSKNRYLTGDRLTEADIRLFVTLIRFDKVYHGHFKCNKKRIIDYPNIWGHTKEIYQLDGIKDTVDFHHITWHYMFSHDSINPYRIISIGPDLGLDEPHGRGNM | uncharacterized protein LOC110049612 [Orbicella faveolata] | XP_020611075.1/1e-158 | GST_N_2(PF13409) ;GST_C_2 (PF13410) |
| **Unigene13309** | 3.7778 | GILGNSGGSGGGLLSR | 1 | QLLRAQPQGSTENLWEDELNNTMRILSIFVFLAALACADALLSDLLRLQVLKGILGNSGGSGGGLLSRLGQSSNGAQSASVVSSSSAGSYMENYYKLQYCRETPFRFIRKCTTSSQCSPYLECFENVCCATNPLSLRIVD | uncharacterized protein LOC110459472 [Mizuhopecten yessoensis] | XP_021367428.1/ 8e-04 | — |
| **Unigene19938** | 3.7778 | EAYPGDVFYLHSR | 1 | LQSAILVGHRREQELVKMLSARFAATLVRQLPRAAPKVCRHALGAGYVASRNISTSTPLCAGAEVSSILEERILGQTSQTNLEETGRVLSIGDGIARVYGLKNIQAEEMVEFSSGLKGMALNLERDNVGVVVFGNDKLIKEGDIVKRTGAIVDVPVGKEMLGRVVDALGIPIDGKGPLGTSTRARVGVKAPGIIPRISVKEPMQTGIKAVDSLVPIGRGQRELIIGDRQTGKTAIAIDTIINQKRFNDGTDEKAKLYCIYVAIGQKRSTVAQIVKRLTDADAMKYTVIVSATASDAAPLQYLAPYSGCAMGEYFRDNGMHAVIIYDDLSKQAVAYRQMSLLLRRPPGREAYPGDVFYLHSRLLERAAKMNDDNGGGSLTALPVIETQAGDVSAYIPTNVISITDGQIFLETELFFKGIRPAINVGLSVSRVGSAAQTKAMKQVAGSMKLELAQYREVAAFAQFGSDLDQATQNLLNRGVRLTELLKQGQYIPMPIEEQVAIIYAGVRGHLDKLDPTKITDFEEAFLQHIRGSQKDLLATIAKDGMITEDSDAKLKQVVKNFLAGFEG | mitochondrial H+ ATPase a subunit [Pinctada fucata] | ABJ51956.1/0.0 | HAS-barrel(PF09378);ATP-synt_ab(PF00006);ATP-synt_ab_C(PF00306) |
| **Unigene7385** | 3.7778 | IPAINVNDSVTK | 1 | MSTKPPFKVADINLADWGRKCIEIAENEMPGLMQMRKMYGETKPLKGARVAGCLHMTTQTAVLIETLTALGAQVQWSSCNIFSTQDFAAAAIAKTGVPVYAWKGETDEEYIWCIEQTLVFPDGQPLNMILDDGGDLTNLVHERFPQYLPGIVGLSEETTTGVHNLHKMMKDGKLKIPAINVNDSVTKSKFDNLYGCRESLVDGIKRATDVMLAGKVAMVAGYGDVGKGCAHALRAFGARVMVVEIDPIIALQAAMEGFEVTTVEECLPKCRLFVTATGCSSIIHDKMFEQMLEDSIVCNIGHFDCELDVKWLNENCAKKEQIKPQVDRYTLKNGRHVILLAEGRLVNLGCAHGHPSFVMSNSFTNQVLAQIELWTKKEEYKNKISVTVLPKKLDEAVAAAHLDHLGVKLTKLTEEQSSYLGIPRDGPFKPEIYRY | S-adenosylhomocysteine hydrolase [Crassostrea ariakensis] | ACT35639.1/0.0 | AdoHcyase_NAD(SM000997) |
| **CL3395.Contig3** | 2.7365 | GVPDEEIESR | 1 | MSLGSIPMADSGICHCSAEWGMAEIIKGESKVAAVTTMSDDDTAVLAGVLAGLATFLFLALPILCCLCPLPFACCGGGGGGAGKKGAAAVGAQRRKNIHEFSSGRSTDVESIGSYRSWDKNWDKLDRFDHDNLYEVDMKLPRAWLESLRGVPDEEIESRLREMSRDGGWEGNDIKQSQEMELRREMYEESGSHGIGGMGTMGTDGSRYATAQRMEHSGGEGELVAEYEITRHVTMDTTRMALPETEYIYERDIRGENNIADRDNFKRIYYTYQRIKGDDSKQTSF | hypothetical protein AM593_02533 [Mytilus galloprovincialis] | OPL21563.1/2e-80 | — |
| **Unigene6555** | 3.7778 | NSYVQQCDCEIIIGR | 1 | DNKVYIVAVLILWTCCPADAVCTCKYLDLQTKLCKSDFALVGRIVSRKPTVDGPRDHLEYTATAISIIKGRISTTFVFQAPKTSSDCGVEFPVRSIQFLMGKRSGGKFVVTSCEVNGQNRPFTTAQWTYLFSRSKRNSYVQQCDCEIIIGRDTPAVGDFCRLVGDGKNQTCYMRNALCRKVGSQGSRQCSWINGEACG | byssal metalloproteinase inhibitor-like protein 1 [Mytilus coruscus] | ANN45954.1/5e-08 | Signal peptide(1-20);NTR(SM000206) |
| **Unigene2791** | 2.7365 | GVNSLIPWR | 1 | KALLIIAFVLPVCEGFILQKNNQNVNRGVNSLIPWRRIGTADVNPSACPDYQQLCFHNKSYCKLVCQSCQKHECVNVKNTDETTCPCECFQNCEPSKSMRRRPLIGRMSVADELGDDHFAYYVGDEDS | otogelin [Corvus cornix cornix] | XP_019141110.2/6.7 | — |
| **Unigene23744** | 3.7778 | TIDTMSLTADHVLADR | 1 | IQISKSDSNQNGLLNARKILSKTIDTMSLTADHVLADRIRSDKIKTTNLRVTDKRKSKVSYYHYDVETPDVTRTNLPVTNKSPTPYSGGDHHSFNRKSKKSGHFHNNDFVHRKVSRGYSKTSTDSRRKILNIQRPRQSSTDKYFESTKVRIKPKNMQNSFEVEYLAPYAVTKPNMAMFIESPTTIRRLSTPDTSNRHYQEYLGYGELNFDPVLPTVDHLQ | — | — | — |
| **CL93.Contig1** | 3.7778 | FTQAGSEVSALLGR | 1 | VIAQSTVAMMHAARRACVGLLKATKHSLTSPAVPSTATKALPSYFNTRHYAAEQTQPNTAKGRVVSVIGAVVDVQFDEELPPILNALSVENRTPKLILEVAQHLGENTVRTIAMDGTEGLVRGTSCIDTGYPIRIPVGPATLGRIINVVGDPIDERGPVKTDKFLSIHAEAPDFVEMSVTQEVLETGIKVVDLLAPYAKGGKIGLFGGAGVGKTVLIMELINNVAKAHGGYSVFAGVGERTREGNDLYHEMITSKVISLTDDTSKVSLVYGQMNEPPGARARVALTGLTVAEYFRDQEGQDVLLFIDNIFRFTQAGSEVSALLGRIPSAVGYQPTLATDMGTMQERITTTKKGSITSVQAIYVPADDLTDPAPATTFAHLDATTVLSRGISELGIYPAVDPLDSNSRILDPFVVGEEHYDVARNVQKILQNYKSLQDIIAILGMDELSEEDKLTVARARKIQRFLSQPFQVAEVFTGSEGKYVPLKESIAGFQRILSGELDHLPEVAFYMVGPIEEAVAKAERLAEDQS | ATP synthase subunit beta, mitochondrial [Mizuhopecten yessoensis] | XP_021356377.1/0.0 | ATP-synt_ab_N(PF02874);AAA(SM000382) |
| **Unigene39610** | 3.7778 | GGNGGGFVTSR | 1 | MMKALAVLFFIIQVSHGSFGGPYGSSYYNPFQMDKFMYDFFTTFNNIMSMKAPAPRPKPQTFPGAQLFPPTFPDFSGKNSGFKTVLINDMKPGTRKTFKVNNGQGIAFRSRDGNAGGMSFSSGTGSGNGFAFGGTFNRGGNGGGFVTSRSGPKGTKVSYSKGIPKFAKNLFSSFSFF | shell mytilin-3 [Mytilus coruscus] | AKI87980.1/7e-40 | Signal peptide(1-17) |
| **Unigene6047** | 3.7778 | FLEAAYGYR | 1 | MYRNYITQLDQKKGGQQQGKMAEETQAQLWDKLNKGESKSLLKKHLTPELYEQLKDKKTSLGGTLGDCIRSGANNLDSGVGLYACDPEAYTTFKPLFDAVIKDYHKVDGVNHPKPDFGDVSKLEDLDQYGGDMIVSTRVRVGRSHDGYSFPPCLTKESRKEMMDKTAEACDKLTGDLKGKMYRLESMSKEENQQLIDDHFLFKNDDRFLEAAYGYRDWPNNRGIFHNPSKTFLVWANEEDHLRFISMQKGGNLKEVYGRLVEAIKQLEAKLTFAKKDGYGYLTFCPTNLGTTCRASVHIKIPKLSKLPEFKQICEKHNLQPRGIHGEHTESVGGVFDISNKRRLGLTEFEAIMEMQNGVKEIIKMEKSL | arginine kinase-like protein-1 [Mytilus coruscus] | AKS48144.1/0.0 | ATP-gua_PtransN(PF02807);ATP-gua_Ptrans(PF00217) |
| **Unigene37975** | 2.2460 | AECIQSQYGNYTVKEANMNLNGK | 1 | EHLAASNFSVASTDLSASVKSNGKSTKGATRAILCSLFICLLAIIALAVALAIVVTKKIDEKNEVPPAPTVSTGSGTGSQTGTGSGSGSQGSGGTSQLPGICTTPDCVQAAAKMISSMDMSVNPCDDFFEYACGGWRRSHVIPEDKAVLGTFYELRDDVDIKIKAVIEEPITQNEWEAITKAKNYYKSCINLTEIEDRNLTDVITTIGQLGGWPVTSTSWDESSFSLETVLLETRRYTNSPPIFDSYAYTDSKNPDARILYVDQQTFGMPNRDYFLKGRNDPVLLAYEKFATDTAIKFGADPEVAKNDMRDMVDFEIKLANISLPPAERRDEQKMYNLVKIQDLIDNYSGINWMGYFEGLLKINGLDITVAATEPVINRNPEYTRQLPNILASTSKRTLANYIVWRYVKRLPDVLPERIRDLQTAYHKALIGTAQVEPRWKTCSKAANSFFGLAVGNLFIHNYFGADAKVDVETMIKKLRESMKELISSNDWMDDATKSVAREKADYITPRIGYPNEVDLEAKVNKKYERITINSGDLYQNTLSLWKSGATENIEDLRKPVDKNKWETAPATVNAFYDPQKNQIMFPAGILQPPFYNKNYPDYLNYGGIGYVIGHEITHGFDDSGRMYDKDGKLNQWWTPTAIDKFRGKAECIQSQYGNYTVKEANMNLNGKLTLGENIADNGGIRESYVAYRKLVKQKHKGVEEKKLPGLQMNPDQLFFLNAAQVWCGIIRPKEAVRRVLIDSHSDARSRVVGPLQNFDLFSKTFNCQPGTYMNPSNKCVVWG | membrane metallo-endopeptidase-like 1 isoform X1 [Mizuhopecten yessoensis] | XP_021361070.1/0.0 | Peptidase_M13_N(PF05649);Peptidase_M13(PF01431) |
| **Unigene38699** | 3.7778 | IQNAGTEVVEAK | 1 | MFSRLAKPSCIVHVARRSFSLTSQAQQGPKVTVCGASGGIGQPLSLLLKNSPKVASLSLYDIAHTPGVAADLSHIETRAKVSGHLGPESLEACLTGSDVVLIPAGVPRKPGMTRDDLFNTNAGIVRDLVEACGNFCPKAMICIITNPVNSTVPIAAEVLKKKGVYDPRRLFGVTTLDIVRANTFIAEAKGLDVSKVNVPVIGGHSGVTIVPIISQATPSVSFPSEERKKISVRIQNAGTEVVEAKAGAGSATLSMAFAAARFTSSLLEALDGGEGQVECAYVQSEETDAPFFSTPILLGKNGVEKNLGRGKLIDYEMQLLEEAMPELKANIQKGVDFVSK | malate dehydrogenase, mitochondrial-like [Mizuhopecten yessoensis] | XP_021347898.1/2e-175 | Ldh_1_N(PF00056);Ldh_1_C(PF02866) |
| **Unigene51259** | 2.0866 | YSAQFLR | 1 | MRIIYVCLAALMVHVNAQDSPGVQIVKKLTGKGFENAVEDVMKCSKFDAEKQWIELEGCVGNVRDGICSGKYSGDAETDCGDYKEGVKWVGMVEVLNHMYCEGFGGCRKEIAEHFCSYYEGNFRDCVNFYIPEKPQRSPGVQMVIKLAGEGFDKATEDVMECAKFDPATEWQQLESCVEGVRADICSGKYSGSSDTKCDDPEAVKWVGMVEVINEIYCEEHGCRKEIAQEICKYYDGDYEGCVNFYTPGGSVAKRKALPKLPPISKKSSQRQASSSLKDVLEKYLSAEDVNKRNRLPDLPPMSKGPSRRYSAQFLRNLLRKYKK | asparaginase [Geosporobacter ferrireducens] | WP_069975581.1 /1.1 | Signal peptide(1-17);Internal repeat 1 |
| **Unigene42863** | 3.7778 | ASANAAAFLQWIPAVGWR | 1 | MRQLVLVALLGFVTNTYAGCSFKASANAAAFLQWIPAVGWRSFSCAPGLFFNPGLCCCAPFGVGASAAASASAAAGAGGSAAAAAAAAAAAAAAAGLGVGAGAGALAGAGALAGAGALAGAGALA | — | — | Signal peptide(1-18);SCOP(d1gkub1) |
| **Unigene34096** | 3.7778 | TDVLLDINR | 1 | TTNWIDGLHQQSGNFNLYLAGNEFSCVCDYKSFIDWLSRTDVLLDINRNYSCTFPNGTRIRIPEVIQNYHRIFSHCNAIAWLRTGVICIVSSFFVIGLTAIIYQFRWRFTYFMYRQLKSRYIKEDPFVFDFVYDVFVAYANDCSEWLVESLIPTLEQEWNLNVCIKDRDFPIGADRGDTVVQS | — | — | LRRCT(SM000082）；SCOP （d1fyva_） |
| **Unigene53044** | 3.7778 | GYQDVIFGGFDQR | 1 | DKSSVGRNISTKVPDGMPVKPYLTDVASIRQDITDDYKYKDESDRERLAVINANRSALKTRPVYNRGYQDVIFGGFDQRDFIMIGKEFKVTVTFRNVGDHVRTIKGRLICESVSYIGIKQKTIKEHSFEIPLDPRSEDVASMKVGLTEYLPHISEQMGMKVSAILQVVETGHIQAFQDDFRLRKPDIQIEILDNHIRVGESFKCRLSFTNPLSAALSKCCLTIEGPGLDNEEQFDLSNISGHQEWAAHLQLTPRKPGRRQITASLDTHQIQNIVGVAEVKVLP | Protein-glutamine gamma-glutamyltransferase K [Mizuhopecten yessoensis] | OWF48045.1/3e-98 | Transglut_C(PF00927) |
| **Unigene2736** | 3.7778 | TQGSYTEIQECFAR | 1 | RKVMEQKLFLIGVVLMCSLLSVNSLCNFPCSVQTGGDYDGELGSFRWSCDNSSRLIRTQGSYTEIQECFARNGPFTVLRRNGNQYQCVKEAAVTGKVTWVYETEFLTMWNPPTVCSICTPVLMRPMMYVDPSVICIPGTKKKGQYTLKSLKKMKPPPIGCNRPKNCPLSSTLDVPCTGCEPFDDGSCCPGCKRKLQQSYAYAYAYADSMFGPQFMGHYNTML | hypothetical protein [Halomicrobium katesii] | WP_080505770.1/0.14 | Signal peptide(1-24) |
| **Unigene52625** | 2.3492 | SSLLQEAR | 1 | DGNNNVPHGIKSANVTKSSLLQEARKFIRLRSPEKEMPHPPILECLCNRKLMGILCRSCGDMFRGRVRKICPFHPTSRYLMDYEVCPSCKATQLQEFEEKVTDMDCEKERNV | CG13380 [Drosophila busckii] | ALC44588.1/6e-09 | — |
| **CL3814.Contig2** | 3.7778 | AEDPVPEEDYEYTSVR | 1 | MPPPIAGHTPRKVTLNKFGGGTTSFGQSFQSNKKSSTTWQPVPAPAGGSMMNRVQDSLDSALSPTSPPQGYYQQQYQQPQQQQYRPPPQQQQYRPPPQQYQSPSQQQYQPPPQQQYQPPPQQYQPPQNQYQPQPQQEPYTPTYQTVGDLQPDYVRAEDPVPEEDYEYTSVRDRKKQFIETRQDAPLIKRGKKKFVPPVAAAYQSFGTDYSSPQPKQVEQPRFPPAPKPVPPPVNRAPEPVDQQDGPKPWAGSLRSESGGPKLWELEDKEYIMPSQLEARQQQQQQQRQSRGQTRQQRQPPAVSPKPISKGTNQIKVAVAPPQQSPARQISVRTSSTVSKQQPQQSQQQGDRDWNQSYVYKMVKEETKRETQMYPGQAPITTQTYSSKTYQSGQPAQEDTYGISDF | PDZ domain-containing protein-1 [Mytilus coruscus] | AKS48171.1/4e-91 | — |
| **Unigene20852** | 3.7778 | YGLEDYIEIK | 1 | SADIEKAVTGVMGSKFRNTGQACICANRIFVQDSIYDKFVSSLANRMTKELHIADGFDDKATQGPLINQRAVDKIESLVNDAKEQGGKVVVGGQRRKGNFFDPTLISDVTTKMRCYNEEQFGPLAAVIKYHTDEEVISMANNTSSGLAGYVFTENINQMWRIAEKLEYGIVGVNEGLPAMPEAIFGGWKESGLGREGGKYGLEDYIEIKYVCLGGLSD | succinate-semialdehyde dehydrogenase, mitochondrial-like isoform X2 [Crassostrea virginica] | XP_022301488.1/2e-93 | Aldedh(PF00171) |
| **CL2714.Contig1** | 3.7778 | QQYDMIELAER | 1 | MSDTEETTQPENEAKLAMEEAAARKAEKIAMEIAEFEEQRREEKAKEEEELAMLREKREQRKIERAEEEKRLAQLRIEEEARRKQEEKERQQKKAEDEQRRKEERERKRKEQEERLKLVKKPNFVITKRADGGDDERRKKAEQKAEEMQKSKEQLEQEKRAILAQRIQELNIDGLKSDGLIQKAKDLHEKLHNLMGEQYDLEQKFKRQQYDMIELAERARQMNKGKNRSTMGVKVDESFDRLADKFINAPPKIQLCSKYERHTDNRSYNDRMNLFEEFSKPKPPPEIIRKGAQTSGAEDGEEEEEE | troponin T [Mizuhopecten yessoensis] | BAA22851.1/ 1e-42 | Troponin(PF00992) |
| **CL4259.Contig2** | 2.1572 | FAAYFQQGDMESNGK | 1 | SRRILVFVLRSRKMANIERPLLTAEASYQSLQKFYDENGSKINMPDMFSKDPGRFDKFSTTVKTKDGDILLDYSKNLVNEEVLKMLFQLARDRKVEEMRQAMFTGERINFTEDRAVLHVALRNRSNSPIMVNGKDVMPDVNRVLQHMREFTQSVISGEWKGYTGKAITDIVNIGIGGSDLGPLMATEALQPYQKGPNAHFVSNIDGTHMAKTLKKLNPETTLFIIASKTFTTQETITNAESAKAWFLEAAKDPSAVGKHFVALSTNATKVKDFGIDEKNMFEFWDWVGGRYSLWSAIGMSIALFIGMDNFEELLAGGHFMDQHFLNTPLEQNIPVILALLGVWYSNFYKAETQTLLPYDQYMHRFAAYFQQGDMESNGKYITRSGQRVDYTTGPVIWGEPGTNGQHAFYQLIHQGTRLIPCDFLMPVETQNPIQGGLHHQILLANFLAQTEALMCGKSKDDARAELTKAGMSGDALEHILPHKVFEGNRPSNSIMFQKLTPFMLGVLIAMYEHKIFVQGTIWDINSYDQWGVELGKQLAKVIQPELKNNDPVSSHDSSTNGLINFIKSHRS | glucose-6-phosphate isomerase-like [Crassostrea virginica] | XP_022344543.1/0.0 | PGI(PF00342) |
| **Unigene9290** | 2.3492 | LTVEINK | 1 | LDIIPQAIKDKLENVFRKYVGENDDLKIKYERLKVNSEQQYFDIEKQLVTSNRKLVAETTENTELKTSLTQIEQKYKDISEKYAEFRETQEGSLASQLHLTKVNDQLEAEKRDLTVLLDKRNKEIDRLNEEWKEITEKLSKATSAKCEAQAKLSDLQSSQVTTEFQHKRLQQENEQMKGQIDWLNTDLGEKTKELMNCRKEQNSKVINIQTQLEEKVDEVKQLTDTVESLKKTCSDKENKVEKLIQRSKDAADANIQSEEQFRKELEAKSKLIELYKGSAEDAEDKVTELNRAVEELQKLLQVSADEHTIMEKEKNEEIGKLTVEINKKEDDIRKLNQELINANDLIEAARKKNATDQQVETMFPSAAATSKYLKSGMTLTQIYNEYVNTSDELRLEKEENQRLQQSLDLVLQEIEEKAPILRKQREDYEKCLNNIDQLTKQLDGALLENQTLRSEADESIRKYNYMQRENHKYQQQMLDLSHQVQFLVKEIEEVKTGRAIRTDINLSSQEVSSSSDAISSRLVTFRDIEELQMQNQRLLSVARALGEEKEKEERIATESKTKELKEELQMAQNELKELKIARDRQQEMVQAIINQRDMYKVLAQENAGAIPSSLSTTPMPSRSASSIMRSPVSSRSSGLEATIEETKTALKQMTENFTTYKKEKNENERILNDQMEKAREELNGFRVQNAKLASQLDFQAERYKVLQGNNDGYKKEIASLREKIQKYSTSVAKHEQTINTLRQDSMSAKEDLARSQAQNQNMRMEKELLKSSEGRLLQEIESLRRERHSQTMLMANLEAIKNNLERSEFETKTRYSNKIEALEMEVNNLKRKLNDENDERKALTTLWENNTRTLRAQLEQRIKDHEDNKLKFIALDKEKQELRQKVLDLEMQLLAAEQAALNRGSTSTVVPGQTQSESSALDREKIKDLQSSLDQSTLEIRQLKEQLEQVKSQREQFKTIADGLQKT | nucleoprotein TPR-like isoform X1 [Mizuhopecten yessoensis] | XP_021362246.1/0.0 | — |
| **Unigene5656** | 2.4880 | ATFIGEGK | 1 | LIAHGDNGKATFIGEGKTIRKYPLIIASRCNIPFLLPVEGTGEIIKGEVYEVDNAMLRRLDALEGHPVWYKREEIPIHCNKTVLKCWCYFLDHYRVDLLHRKYHDNYDSRSNEYITPAERKRMDTENFWLEVKRPEFYISPTEFERMYPYAKDKY | putative gamma-glutamylcyclotransferase CG2811 [Mizuhopecten yessoensis] | XP_021342717.1/ 1e-29 | GGACT (PF06094) |
| **CL3748.Contig1** | 3.7778 | LAAQYAEAQEEIQR | 1 | MPKAVNVRVTTMDAELEFAIQPSTTGKQLFDQVVKTIGLREIWFFGLQYVDSKGYTTWLKLNKKVLSQDVKRETPLQFKFRAKFFPEDVTEELIQDITQRMFFLQVKDCILSDEIYCPPETSVLLASYACQAKFGDYNKETHPNGFLANERLLPQRVMEQHKMTREQWEERITNWWSEHHGSLREDAMMEYLKIAQDLEMYGVNYFDIKNKKGTELLLGVDALGLNVYEKEDKLSPKIGFPWSEIRNISFNDKKFVIKPIDKKAPDFVFYAPRLRINKRILALCMGNHELYMRRRKPDTIEVQQMKAQAKEDKMSKQQEKQRLESERIAREEAEKKQKEMEEKLRRFEEESERRAKEMAEQERRLRDMTEEMEAYKRQQEELEEQRRAAEELRRQYEESAHLAQEEKDRLAAQYAEAQEEIQRSMAVLEEKESEMNSMQQNLEQAQKEREEKEQALIEAMNTIHVRETEHEENTVEEVNHEYSQVETSEDVHMTFENEYEENTVEEMNHEYSADLQDYEQVESLPRPEEERLTEAEKNQRMKEQLKSLQEELQNTKIDEKATTTDMLHAENVKQGRDKYKTLKQIRQGNTKKRIDEFESM | Radixin [Crassostrea gigas] | EKC27987.1/0.0 | B41(SM000295);FERM_C(SM001196);ERM(PF00769) |
| **CL3196.Contig1** | 3.7778 | QAVSTSVTQFLSDQISR | 1 | MDFVKYLMVPAFLGSIFHNTVNGELTSSLKQAVSTSVTQFLSDQISRVKISPISIAGIILTVTGTFTANAKKVYVYIEIVKATNQVGYCRAIIGQMTIGFKPSQPSGSTEGPVNLSSSLIGELKKKLAEEICKNMKNTLNSNPLFPPP | transcriptional regulator [Phaeobacter sp. 11ANDIMAR09] | WP_054459405.1/1.8 | — |
| **Unigene17193** | 3.7778 | GLVVPVIR | 1 | LKSMALLLVQRCLPRITRRLSPTNTKFVLEEASKNIRVKSHICCSSQCRNYTDVQYIRFTKKYCCNSQLIIKRNFHVTNRYFDDVLTAATPPFADSISEGDVRFEKAVGDFVKEDEIVCEIETDKTSVPVQAPKSGIIQSFLVDDGATVQAGTPLFTLKLSDSPGESAPESVAASEKPPPPAVTSKAPETPVATPASGPIPTTPPPPQPIPKAPISTKPLDSIKPIPATDAPVMGARTEKRVKMTRIRQKTSQRLKAAQNECAMLTTFNEIDMSNVIEMRNQYKEAFQKKYGLKLGFMSAFVKAAAYALTDQPAVNAVIDESEILYRDYIDISVAVSTPKGLVVPVIRNVGSMNYADIERAIAELGEKARTGSLAIEDMDGGTFTISNGGVFGSLFGTPIINPPQSAILGMHAINDKPVAIKGKVEIRPIMVVALTYDHRLIDGREAVTFLKKIKSAVEDPRVLLLDL | 2-oxoglutarate dehydrogenase complex component E2 [Mizuhopecten yessoensis] | OWF50391.1/0.0 | Biotin_lipoyl_2(PF13533) ;2-oxoacid_dh(PF00198) |
| **Unigene17159** | 2.3492 | EGQGYISGAEMR | 1 | MSKLSKGEIEDAREVFDLFDFWDGRDGDVDAAVVGDVCRCLGINPTNAVIKKNGGTDKMGEKGYKFEDFLSIYETVNQQTEQGTYADYMEAFKTFDREGQGYISGAEMRQVLSSLGEKLTDEQVDEIIRLTDLQEDLEGNVKYEDFIKKVMAGPYPD | myosin essential light chain [Crassostrea gigas] | CAD91423.1/4e-79 | EF-hand_7(PF13499) |
| **Unigene28664** | 3.7778 | YTSLLQR | 1 | EARKREFMQGRYTSLLQRQRDGRGSLHLVVALINAGADIDGSQYQPITVAAEADLADTVYMLINYGAWIPINWKFLSPDVLFGENMAQILDRIKSHVEVQITLLFQCKRKIRKLLAHTGQIESSINKLPIP | ankyrin repeat domain-containing protein, partial [Alkalispirochaeta alkalica] | WP_083914805.1/0.73 | SCOP(d1sw6a_) |
| **Unigene26867** | 3.7778 | IVVYLNK | 1 | FFPRTFFAIWGTSKYQIRQLLPRNRLTILTIWRNYCKVANSTSKPRCNVGTIGHVDHGKTTLTAAITKVLAKHGQSKLVTFDQIDKAPDEKKRGITINTAHVGYETSKRHYAHTDCPGHIDYIKNMITGTSQMDGAILVVAASEGSMPQTREHLLLAKQIGVDKIVVYLNKMDLVDDELGDLVELEMRELLEEYGYDSTKTPVIRGSA | elongation factor Tu, mitochondrial [Culex quinquefasciatus] | XP_001843948.1/1e-84 | MMR_HSR1( PF01926) |
| **Unigene44594** | 2.3492 | DPQIFVGR | 1 | MTEWVKTSANNIPNGSIRGGYDKNGHTLFIARALTDDGFYSAGKASLHYEDGAHIPYRGQEIIVYEYEILVLPSQADGFYDWKPTASANVPSNAVPSDINRDPQIFVGRFVHEGCLIPGKVDKKKMKCYIAHNGKEYPNDHYEVLVKVK | DM9-domain containing protein 2 [Crassostrea gigas] | AVN66933.1/3e-31 | DM9(SM000696) |
| **Unigene6711** | 3.7778 | FEFSYFGTR | 1 | TDGFLAHNGRNNGMTYNIINYKLLTNKRLCIRFEFSYFGTRTPHHSCHQALLTTTATSKITKPSTVLTTTLRPRTTSSASDTSSHSTTKTSTTADTTTEKLITPTKSTTQRLTTEHQTWPTVSATTATTSVRSYTATSAIFTTTMQTTQNTQRTTAQQRRTTTTLPPTTTTLPTRPPMPDPSR | — | — | — |
| **Unigene27989** | 3.7778 | SGFGAAGFGVGGGR | 1 | MGSGKGMGNGGRGMHMEHGMGSGGDGMGMGNGGVGGSLNVWDHLSSNTGSSGSGSGRNPANNGRSGFGAAGFGVGGGRTLSNMGGSMHKNGGMGSGLASHGTNVWNRVNGQSNNKPMSNSGSVLSGPGGARNNGVANMNNGGTPLVDQGTKSKPVEHPPTPPASMSSVGGGFGRASDISFHTMGSMLQPNQGGSVSQPQSKNQGQTQPKQPGNATPQVSNQNPANSGSSGGGVGVNPQTNQPATNSQNSGPTGNNIPGINNGGSQGGNVGGSHGGNMGGSQGGNMGGSPQIETTGGATSAPNHNFGQGNSFFGHGTNNQGNLGTNNVVGTTAYPGFTSMATWMFLK | — | — | — |
| **Unigene27147** | 2.3492 | KGSSNNLK | 1 | CDTSLVILTQADLWNEEEYKKRWTKENNYDIAERKGNIFMAIQKYIHGPAVSIGNSGTFLPSKETGPPVPTSVRSSLIQFKCRPLSTVNEGNINSKEKGDSCIEQCPVKKSVLDLENVCPQRTQAVNMQQQLPKSDCTPKIKDTNKETGDSELKLQPNSGNKLLSNKNQTFDESEHNKIKYTTRKDEQNILNKTIPNSSICLKEIQEAKNSDIEIKQDMVYHLPKPSEQAHSISCEVTKVVLDTNQNEKFVNIHQFAQVPLQNMQGIQQNLTMPIQSAVTSVKNPVQFITMPGVQWQYPAHEHFQNHTRKVQISEKEYDIHNLKRNENWKFKRTNAHASKEVEISTVPTCSGKEVISAIENQSLSSDKMSHNQWVSGETIDKSSNEVTPLTLSHLFDIKVSNIPSIGSNARKFVPVGQNSEATTKKGSSNNLKHDMKIPSDNYCTLLPQTSSGSEKLIEQYLQAGSNTVKNGCQEKQQSIMKVNNAVIAIAPPSTVQEFSNCKPGNSLKDHQASLKKSRKQNDSMLVTDVVFDENRKAWIMSMKPIIQNNDPKCLVQPKDKEYHLKEQSSYRKHISENIGKAKHDNSGSIREKCSKIISSECYEPDEDNVTREKTVKKDLDQPSCNFTITDVKSINDLSNNQYCKTHLEIKVEPLDTSLDGNNYTKSGSVLQKDYQYHKQDVHPNHDGTELVINNDFKDASLLNSTFQVNKVEAFPSFQKKFLQRKGKIAQILKKIQDDSKRLENFRCLKQNKQKYPCKKTEVEISTLEDTCKPGQQNSKKYFFDGCDNYLDFSTASVDKKSDGMDILTNDNGSRMQTTPLKLSIRKKDGNQYEIVKSNVSRQIIYAPSFEEDPNDVNTHTSHSKEISTIDKQRKRKLDCCTMYDGKSGGLLPCLLPLKKRCVIHQHETSNGSLNSTVTEGEHLFDYVLSEKESDLESDCSLTDEPSLLKCVNTRFYNFNDIDSD | — | — | — |
| **Unigene52317** | 3.7778 | LTPIDYEFHVWR | 1 | VYTDVDRNMKVVRGNILIKNNFEDGLDEFNDNINVFDNGFNMSKSNPLYNSDEDIYKRYEEEKRRQQERDIRDMENMAFETVDRFSKSKNVTRKADGERRKPRSKQNLTNLLLTRLSTMDSKDIRQTLDVKHHHEDVYGDPHLEQADGTSSRRITNSNYDTVTENVDLEMKDGKAYITITVTAERLTPIDYEFHVWRKNQAIVTRVVEIDFYADEQRRMLYDRVMDRVDDEGYSTTNKYSHYTRRKPQEVVLSSQDTLELFNELMDAAGGEGDREDITVKTKRNKTLLPENPFLY | hypothetical protein AM593_03515 [Mytilus galloprovincialis] | OPL33566.1/5e-170 | — |
| **Unigene26538** | 2.2460 | IPEPEPAGR | 1 | IIALLRPAEDHKCRPCVNWIHWFFGTVAWCLAIPNMFIGMDFGKAHVPWWATWILCIYILYHIIVEVTLEVHQCCTHKKNKERRKKYEFQKRENPKARIPEPEPAGRVFKRNMLIVHFIITCIVNFIMLIAIAAS | ferric-chelate reductase 1-like isoform X1 [Mizuhopecten yessoensis] | XP_021369047.1/ 4e-79 | B561(SM000665) |
| **Unigene14496** | 3.7778 | SNTLQNLHVQQPAR | 1 | GACCGAPPSPEMGMRSNTLQNLHVQQPARQQYETQASILTNFNEMANVYVDKNAQKTPAKNESDVLSPDYRPYLPPSTYSPFGPYGAPSQPNNGNYDQNAQIKK | — | — | — |
| **CL46.Contig2** | 3.7778 | GFLTTQLPLPDTMVDFWTMITDHNSSTVVLLLNNIK | 1 | MVKPKTVKESASFYDNCDKSTLIKVSDLQDYIKQQQKIDGFQKQYKNIPYGVQFSTTKAEMTENTPKNRYKLTLPYDHTRVVLESSCSDYINANFVDGFESDRRYIASQGPTKNTINDHWKMIWQYNIRKIVMLTNLMEIGKIKCEMYWPIGGEKMKCGDIVLTLLLEKERAAYITREIGVENIKTQEKREVIQYHFTAWPDHGTPDPLYLALFHKHIICDQHNKNNGPILVHCSAGIGRTGTYIGLDALYEEGAITQYVNVVQYVKKMRYSRMNMIQTQEQYECLHFALLERFTLQDTSLRKADFANVWKDIKADKCPMNHLGLSEEFKMLQEQLEEHQPLQYSDAKSDDNIVKNRSQNVLASDKTRLFLISYEKGRTNYINAVQVPSYTKFKGFLTTQLPLPDTMVDFWTMITDHNSSTVVLLLNNIKEADLVYSNSEDNLSSGPFIIKPTRSGSVKHNDIDVKTVLLSQKDDKARPIEIFQCVYKDRPDPSSLCTIVSLISTRIDMSCDPVTIVCSDGAKNIGLFCTFINAVSSMTIDGNADLFQLARLLKLRRHEFFNDLDEYRLCYEAVNLYLESSDVYANC | receptor-type tyrosine-protein phosphatase alpha-like [Crassostrea virginica] | XP_022306856.1/1e-127 | PTPc(SM000194) |
| **Unigene3834** | 2.1572 | GVLMVGPPGTGK | 1 | MSSATLLGEIDENTKLGRENALLGNYDTSLVYYQGVLQQIQKLISTISEADRKRKWMQARDLISQEHDQIKEISETLSSFKSNNPKPYPDNDFGSPFGDYARHEEPTRDPDVWPPPTPVEYRPSPNIRGGRPAPKKVEPVRRPGGPSKAAPSRQPDRGRPGAPGYGRDNRGRDNKDNKKKNDDGEKKFDPTGYDKGLVEGLERDIIQKNPNVNWDDIADLTEAKKLLQEAVVLPLVIPDFFKGIRRPWRGVLMVGPPGTGKTMLAKAVATECGTTFFNVSSSTLTSKYRGESEQLVRLLFEMARFYAPSTIFIDEIDSICSKRGSDSEHEASRRVKSELLIQMDGMYEGVGGSADDSEEQKIVMVLAATNFPWDLDEALRRRLEKRIYIPLPTAVGREELLKINLKGLEIAKDVKLSQLAETLEGYSGADITNVCRDAAMMSFRRRISGLTPEQVRNIPKEELEIPPNMEDFEMAIKKVNKSVSAQDLEKYQNWMKEFGSV | Katanin p60 ATPase-containing subunit A-like 1 [Mizuhopecten yessoensis] | OWF43912.1/0.0 | PDB(2RPA\|A);AAA(SM000382);Vps4_C(PF09336) |
| **Unigene53649** | 2.2460 | IILLAEGR | 1 | SEDMNGGGRNDSAGILAMKPNPGLKFEKKLSETERSTRDMKLVAKSGKKKKDVKFVLTPTKKRLLSRSISASSSTDSFDSTSYTGSSSDEDDVNPREKVQKNSKGSGDFCVRNIDHAAFGRREIEIAEQEMPGVIALRKRAEADKPLSGAKIIGCTHITAQTAVLIETLAALGASVRWAACNIYSTQNEVAAALAEAGYPIFAWKGETEEDFWWCIDKCINCEGWQPNMILDDGGDATHLMLKRYPAMFNMIKGIVEESVTGVHRLYQLSKGGKLTVPAMNVNDSVTKTKFDNLYSCRESVLDALKRTTDVMFGGKQILICGYGEVGKGCAAALKGLGCSVMVTEIDPICALQACMDGFRVVRLEEVIRSIDILITCTGNKNVVTRSHMDRLKTGCIICNMGHSNTEIDVGSLRTPDLTWEQVRSQVHHIIWPDGKRIILLAEGRLVNLSCSSVPSFVVSITAATQALALIELYNAPPGRYKQDVYLLPKKMDEYVASLHLPNFDAHLTELSDEQAKYLGLNKTGPFKPNYYRY | S-adenosylhomocysteine hydrolase-like protein 1 isoform X2 [Mizuhopecten yessoensis] | XP_021362456.1/0.0 | AdoHcyase_NAD (SM000997) |
| **Unigene20162** | 2.7365 | GMTAMGAVR | 1 | ISHVGTEAQRLNYNGPTIGAKPTEKRAVKFSYEQLKQSCGLIGLQSGTNKFASQRGMTAMGAVRHISDIRADKFSKEAEGEINLQSGTNKFASQRGMTAMGAVRHICDIRADQYDPESNKEINLQSGTNKFDSQAGMRGFGAIRHISDVKVNELDREGTSVLRLDMGYVGGDSQKGMTSFGAQRHITNVKVNDLAEEFALQHGKPAPTPQPQAVEEVAQEEEEE | calponin-like protein-2 [Mytilus coruscus] | AKS48163.1/2e-125 | Calponin(PF00402) |
| **CL1815.Contig1** | 3.7778 | DIVQFVPFR | 1 | LTSFKLQRTPSFLEYLYGGMQINFTVGIDFTASNGDPNSSNSLHYINPYQPNEYQQAIQAVGNVCQDYDTDKMFPALGFGARIPPNNEVSMEFALNFNATNPYCAGVQGILEAYTNCIRQIRLYGPTNVAPIIYHVARFADAAQKEEATKGAHSYFTLLLLTDGVITDMNDTRQAIVNASGLPMSLIIIGVGDADFADMEFLDGDGGVLKAPNGQPAQRDIVQFVPFRDFKRVSAAQLAKHVLAEVPQQVVKYYTMRQIMPNPPRQAQQ | copine-3-like [Crassostrea virginica] | XP_022342060.1/9e-143 | VWA(SM000327) |
| **CL3011.Contig1** | 2.2460 | YCPGER | 1 | METQILVVSTIIVGTFALVPIPLEPQQPCFTHFGECLRYCPGERIPDVEGIESSCITNTAGYQFCKFYHCPPAKCRGRKKDLYGKCYYCPGKCIDGGRIHNNGQAFLSFDMMNSCFCGQDNSKHCTKRPVDQYAYCNHARPGTDGDAGTVVVVGKPQPKIEIEPEVVAVVHAPPPPPQPNVVAVVQPPQPPPQQNVVAVVQQPPPPPPKPRVTIVHQPTHRPPHANVVAFVPQPTPPRPAPRVAHVPAPTAPPPPPQFAHVPAPTAPPTPPQHAYVPAPTPPPQPPNHVHVPAPTQPPYVPPGVAYVQSPTQPPPPQYLPHYQQHNPPGMVPHPNFQAKSPKSNLNQRSNSHPYPQMGYNYHHNPHPSWNNQHQQYPGPPNNQQYNTPNPFFLWKFMMANSKSKQKEIEL | — | — | Signal peptide(1-17) |
| **Unigene5482** | 3.7778 | HGDDLR | 1 | MTEFSDRFNYVYSCDLDVNVQIKIGTLEGERQRPSYQELLKDPMLKFSGVYQEGHADLYVTCRIYADGRPLSLAVSTSYKAFSTRWNWNEWLTLPLKFSDLPRNAVLAMTIWDIYGTNKAKPVGGTTISLFGKRGTFRKGMMDLKVWPDEEANPQINSTTPGKTKDSNDQMSRLAKLSKKHRDGHMVKVDWLDRLTFREIELINEKQKRDSNFMYLMIEFPYIHYNDVQYTVVYFEKGGDEPFQYRVQADIVNVPDPEILMENLVESKHHKLARSLHSGPTDRDMKPDAKTRDQLNAIVAYPPTKMLSSEEQDLVWKFRFYLSSQKKALTKFLKCVNWKMPQEAKQAIELMSRWSPIDADDALELLSPAYTHPTVRKYAVSRLRQADDEDLLLYLFQLVQALRYEDFEKIKHDTDQLTTRRDSISDLSDRDRTHTVLTRAGSHDSIIDALGGASPKQEESPELKLSKEIDLASFLIERACNNSILSNYFYWYVSVECENDDSTAKEQRVNEMYLCIMKRFSQALVKGGQECRLKRATLARQQMFIEKLVNLVKAVTRESGSRKKKIERLRALLQDPETNKINFASFEDLPLPLDPSVKINGISVDKATLMKSALMPCRLTFKTNTGAEYVTMFKHGDDLRQDQLILQIITLMDKLLQTENLDLKLTPYKVLATSSKHGFVQFVEESTPLAEVLASGNTIHNYLKKHAPMDGAPYGISPEVLDNYIKSCAGYCVVTYLLGVGDRHYDNLLLTKTGKLFHVDFGYILGRDPKVLPPPMRLGREMVDAMGGTNSEHFHDFKKHCYTSFLALRRSANLILNLFALVVDASIPDIALEPDKTVKKVQDKFVLHLNDEEAVHYMQNLIEISVTAVMAALMENIHKMAQYWRK | phosphatidylinositol 3-kinase catalytic subunit type 3-like [Mizuhopecten yessoensis] | XP_021360544.1/0.0 | PI3K_C2(SM000142);PI3Ka(SM000145);PI3Kc(SM000146) |
| **CL4091.Contig2** | 2.2460 | WTVYTKSPSFNDATAPQYVVTISCDDSNSTPR | 1 | MSAYFLFSCMLVFFTWRQCQSAAPIFSGLPTERSVGELETTSRLIYILTVSDPDGDAFQCSVSATVPTSAFFECKKHTVLNKWTVYTKSPSFNDATAPQYVVTISCDDSNSTPRTAELTVKVVNNDLLAFTNMPVSTTSFDAMTTGAGTTLYTVAATSTLGSGLLTYTMTPMTDFSIDSSTGRVTNIDHLNRQISSPADLWVTVSDGTISVTDLLRINMNNLNNVPYFTNLVTPQTITIPEDTASGTLLFSLTSQDNDVGAALSYSCTVNPTTDAIKFSFSTNTLELRLASGQTFDFETRNFYNLTFTVDDTMASTTSILYINIQNANEPCYFDQSLYHVTTPEGSAGSGSVNPSFVVKDYDGTSTYSYSFMNFNNSNRFSIDASTGVITFAVNYDIDNSAMPALVYLTVICTDTTGETGTSQVEITITDVNDNAPSFATASSILTVNQYTAAGALIGSVLPTDADQGVNADVTCSGTSGVAAALSYYQVSSDCGVYLLQQPFGTIAYGTLYTLTVTATDNGNPQLSSTYTVDVLFRELTTTTATTTTTTNPYDLWDDPGAVAGISMAIILGTILSAVFLYFCIRCCYTGMCCGPDPCDFCNWCTRDACNCCQPRYRQNTRTITPQEYRKPRERADEFDYYDKNFDSDYESLKNEETRRPRVHNNSSRLSGLRSYPDFPQSQQSALSPSDGELVERPARILYSGRPLPIGYY | protocadherin-like wing polarity protein stan isoform X1 [Crassostrea virginica] | XP_022292040.1/0.0 | Signal peptide(1-21);CA(SM000112); |
| **Unigene3465** | 3.7778 | GLDTLGNMGFHDYR | 1 | IDRISGVHHDGIREITDKINWEKLYEKLLNSDNKSFEEVLKDLSPDDLLVYEDTINPDDIEKVRTFLLSKLEKNKKYESQSKRGLDTLGSMGFHDFRKRGLDTLGGMGFHSFKKRLNGQNGYTDFGKRGLDTLGGMGFHNYKKKGLDTLGGMGFHNFKKSVSDENQAHIIKKRSLDTLGGMSYHDYKKRGLDTLGNMGFHDFKKRGLDSLGGMGFHDFKKRGLDTHGGMGFHNYKRGLDTLGGMGFHDYKRGLDTLGSMGFHDFKRGLDTLGGMGFHNYKRGLDTLGNMGFHDFKKRGIDTLGGMGFHDAKRGLDTLGGMGFLDYKRGLDTLGNMGFHDYRKRGIDTLGGMGFFDYKKRGLDTLGGMGFHDYKRGLDTLGSMGFHDFKRGLDTLGGMGFHDSKKRGFDTLGSMAFPIYKRQLDINMADEYPSDEEIADILDDLNDQLSLADDNTILNSQSNTGRLKRNVNMAEEPPKQKSRR | feeding circuit activating peptides-like [Crassostrea virginica] | XP_022335398.1/2e-59 | Internal repeat 1 |
| **Unigene7001** | 2.3492 | VVAVGGKPK | 1 | MADESEIQADMDAGMKRGFDEETDDQGQKRRRGDGPRVELRFLLASKNAGAIIGKGGSNIKRLRADYKASVTVPDSTTPERVLTVGAEIGTALDCLLDIIPKLEDYKNFKDDDFDCELRLLVHQSQAGCVIGRAGFKIKELREKTGAQIKVYSQCAPESTERVVAVGGKPKVVVDCIDTIHELLKSAPPKGPNQPYDPMYWDEFMVSEYGGYTAAEGGRGKGGPRGAPPGRGMGMGGMRGR | heterogeneous nuclear ribonucleoprotein K-like isoform X3 [Mizuhopecten yessoensis] | XP_021358948.1/3e-114 | KH(SM000322) |
| **Unigene36836** | 2.0866 | GSQNTIETVR | 1 | MLLEIQRTVILFVLLLSYICAQEVAKVGGKIKVESLDPFAKPGVDPFASIRSAGGSGAQKQGATILTKKETVRTVTTNNKPVVDGSDVRSSQAGTSKNTQTTNEKPYVETTTIERKTRVIGSGSGTLRKSSNTNAQNIASKTETTKTQRTVNVVNRGTGNTGTQSRSGSVQTTGSRSQSSTTRTVQTTNTGSRTGSGERNTDLPRQTGGNRGSGQNIISVSSQTDRQLGQIGSRGSQNTIETVRRNDNVGPFGSSGGSNTGIYTNDGTMKIDTNRGNALPEMGGEMGATDKPILYFRKLYHDQSICLNCIGSGSDCYTEHPDDCRKYIRCTTSSLGGRMEALEMDCAFGTFWSNEANTCDTASEVFCNKDPCMDTSLKTYHSGLNCRSYWRCVNGRSNAECCDNGQEFVDGHCIPSDRCTIDDGECPFKSQFLDTIPEKKPTCNLEPVPEKGEKAFRMNNHWALMYCADGTKFDMKTCGCIVSIFNKVRCVPTVDIGFDEDRVIDRTRGYIKSENVTVTGGKGSAYALFQGESEINVPYFKNNENIKSHLVVSVRFFKNDDKGPGSQVLASNCKTSNKRTLTPSFAILLNKKEKQIVFFASTKKNDKYGATQHITLPFTPGKWTTATLKFLDETLQGTVETLEADGTPKIQKEEKPLKGTLNTGIMPLMIGTCNMNDGFFGYIHKAKAYMCKPDQP | Follistatin-related protein 4 [Crassostrea gigas] | EKC18454.1/7e-42 | Signal peptide(1-21);ChtBD2(SM000494) |
| **CL3895.Contig2** | 2.3492 | GTFANIR | 1 | VTEAKVKMATGVNPYQQLVESVKVGEKSYQFFNVAALKDPRYEKLPFSIRVLLESAVRNCDGFHVNQKDVENILDWEKNQNSNVEIPFKPARVILQDFTGVPAVVDFAAMRDAVKRLGGDPEKINPVCPADLVIDHSIQVDVSRSTLRFSPNPGGGSCLKCEGPCSEKICPFHGAKTSSADALEQNQELEFERNRERFVFLKWGAQALKNMLIVPPGSGIVHQVNLEYLARVVFNDNGTLYPDSLVGTDSHTTMINGLGVVGWGVGGIEAEAVMLGQSISMVLPQVVGYKLMGEVDQMVTSTDVVLTVTKHLRQIGVVGKFVEFFGPGVANLSIADRSTISNMCPEYGATVGYFPIDNNSLDYLLQTGRTPEQTKLIEQYLRSVTMFRNYSNSEEDPVFSEVYELDLSSVKACCSGPKRPQDKVLVDDMKTDFTNCLNNKVGFKGFAIPSDKQLTTIPFVYDNKEYTLSHGSVVIAAITSCTNTSNPSVMLGAGLLAKNAVESGLTVQPYIKTSLSPGSGVVTYYLRDSGVTPYLEKLGFDIVGYGCMTCIGNSGPLPEPVTESIEKGELVACGVLSGNRNFEGRIHPLTRANYLASPLLVIAYALAGTVLIDFEKEPLGTNGEGKPVFLRDIWPTRKQIQELEKAVVVPTMFNDVYSRIQSGTTRWNSLEAPEGMLYPWDDKSTYIKSPPFFETMTKDLSGVQGVKDANVLLNLPDSVTTDHISPAGSIARNSPAARYLGSRGLTAREFNSYGSRRGNDDVMARGTFANIRLVNKFMKKPGPKTVHIPSGEEMDIYDAAQRYKQENKSVIILAGKEYGSGSSRDWAAKGPWILGIKAVIAESYERIHRSNLVGMGIIPLQYLEGQTATSLGLTGQETYTIELPSDLSPGHLTDVKLNDGRTFQVRVRFDTEVELVYFRHGGILNYMIRRML | cytoplasmic aconitate hydratase-like isoform X1 [Crassostrea virginica] | XP_022311953.1/0.0 | Aconitase(PF00330);Aconitase_C(PF00694) |
| **Unigene2555** | 3.7778 | FPGQLNADLR;LHFFMPGFAPLTSR | 1 | SESCECLQGFQLCHSLGGGTGSGMGTLIISKIREEYPDRIMNTFSVVPSPKVSDTVVEPYNSTLSVHQLVENTDETFCIDNEALYDICFNTLKLKNPTYGDLNHLVSLTMSGVTTCLRFPGQLNADLRKLAVNMVPFPRLHFFMPGFAPLTSRACKDYRAVSVQELTQQMFDAKNMMAACDPRHGRYLTVATIFRGHISMKEVDEQMLNVQNKNSSYFVEWIP | beta-tubulin [Papilio machaon] | BAN92310.1/6e-141 | Tubulin(SM000864);Tubulin_C(SM000865) |
| **CL1757.Contig1** | 2.0866 | FPGQLNADLR;YLTVACMFR | 1 | MREIVHIQAGQCGNQIGSKFWEVISDEHGIDPRGLYHGDSDSQLERISVYFSEATGNKYVPRAVLLDLEPGTMDSVRSGPFGQLFRPDNYIFGQSGAGNNWAKGHYTEGAELVDSVMDIIRRETEGCECLQGFQMAHSLGGGTGSGMGTLLNSKIREEYPDRIMTTFSVMPSPKVSDTVVEPYNATLSVHQLVENTDETFCIDNEALYDICFRTLKLTTPTYGDLNHLVSATMSGVTTCLRFPGQLNADLRKLAVNMVPFPRLHFFMPGFVPLTSRSSQQYRALTVPELTQQMFDAKNMMTACDPRHGRYLTVACMFRGKMSMKEVDEQMLNVQNKNSSYFVEWIPNNVKTAVCDVAPRGLKMSATFIGNSTAIQEIFKRISEQFTAMFRRKAFLHWYTGEGMDEMEFTEAESNMNDLVSEYQQYQDATVEDDMDFEEEEGEADEF | tubulin beta chain isoform X1 [Parasteatoda tepidariorum] | XP_015920593.1/0.0 | Tubulin(SM000864);Tubulin_C(SM000865) |
| **CL2758.Contig1** | 3.7778 | SAVVAAER | 1 | MATNILCFGFLISTTILKLVNGAAPISAKGGGSNSFVFSAKNKFSFSNPSPPTSSQGQGFSSSSSSSSSSSNAGSGGGFSPNNNQNQWKQTLPINYEPNKQGNNQQPNQQNGNQLSSQQQQQQQQQQQQSGQQHQQQTQMQHQNMQMHPNNQFGQPQTKPMNQNQKFNSPGQMGSGQQWQPMGFGQQQQQQPQNPQHQQQNTFSNPQQPQNGFSNPQQNSFSNPQQHQLSNMNTGQTMFQQPPNQQQKQQFGQPNQIQNSHMTPFGQQQQQQQQQQQRNQFNKFGQPQSGSNMNVLSQGGQPNNGMPPYQGMPLTANNQQGFQGQRPQQQQQSGHMTQYFPPHQSQQTNQMHGPGQNQGHQGQMPMNGNMPMQGSPQNMQMGSHQQPMNGQLPAHMQGGPQSMPHPSGSSAPAHQQMGRPHPPIGGAINPPMGGAINPPHGGPQMGGPGHSPHGGPYNPNQGGPGYPPQGHGPRPGHPNHNVAVIDHPRKTTTTPKPQTSTIDGEGMVCMTTADCEIGCCFNATGQLLDTTTYGAGGPKEGRASGKCFIRKPGLGDVCDDLCACTMGHDCYRRYVPVYPKPGQKTAPVIDPEAAPKPQRTCVRSAVVAAERIAFWSCYFDVSCSGPLP | — | — | Signal peptide(1-22) |
| **CL1023.Contig1** | 3.7778 | STESADAVER | 1 | KNTKTFVVEDVVLPKFEVNVILPSIQLTTDSHFTATITAQYTFGKPVEGDVLLSIYSGSKRRGITKRFKINGKATIRVLMSEIAPRFRYFTVEAEVTEAVTGDKQGNAQRTHLYETQEKLLFSPTMPYTFKPGLDYNIILRATQENDKALTGYLGQVNVTVFYRVPKQKEEKTGALQCTEGMCPPSDETEEKVLWSKNIQIPESGLMKEVASFPINALSGQVQADYRMASDRKYLSKAQSPSSNYIQVTIVNDQKAKAGTNLPLMIKATEPVQYVNYKIFAKQYMMQQGTFDMQNTSSKQVQIAITTDMAPKVKLLVYYTRPSDGEIVAAAVKFPIEGIFDYEVTLRFNKERALPGDNVTLSVTADPNSLVSVLAVDKSVLLLRTGNDVTVKDVINELNNYDGNLYPNFGAWDYWFSRPISSIDASSVFKDMGVYVLTDSLLYKHSEVVRRSQSFNVPMNAEILAMDAPAMSFTSGGMDDLAKPSRTRKNFPETWLWTNTMTGASGISMINAKAPDTITEWVTSAFAVNPTSGLGVSSDIANLTIFQKFFMRFELPYSTIRGEIVIVQITLFNYLATAQNVQVKLNGGEGFSFVDANGDPLNTGSNGMTKTTLVKNDSVSSVYFPIKPTTVGKVTLSATARSTESADAVERELIVEAEGIKQSYNIPLLLEAQSPNQGTVIRTPVTFPPNRVPDSTFVKVQVIGDFLGLALANVENLLGSTSYGSGEQNMITFVPNVYISSYLKTTNRLTTEIKKKTERLMQGGYQRQLSFARIDGSFSAFGSNDPRGSTWLTAYVIKSFAQAAEFTYIDKKVITKAIKWLLTQQAHSGNFEENGIFIRKELQGGSTSSARSLTAFVLIALYEAKSNDQVEAEIKSNVDSAISKATQFVAEGAPASITNVYELAISFYALSLVKHASKNLLLVELEKKESIDGEEKFWRIPKTEADIIQPWKEWSPQNEEFRALDIETTAYVLQGYNLNDDTKNGTRILRWFGRRRRSRGGFKSAQDTAIALEGLSELAKKLYVPSTSLTINVKADNLAGRTFNIRDENSLVLQNEDITTLVDHIEVTTSGTGISLMDIDVYFNVMSELRVPAFNMTTALPKDSTKGFRLRICFSYLKDDESGMALLEISLPSGMEADLTSLDTSRTWGKFKKAEKAFRQINLYFESIQSRNMCVELDVNRVSLVARHQAVPIRLSEISEPSNEVIRLYKSKALSTATIIEVCGADNCQELRK | protease inhibitor-like protein-1 [Mytilus coruscus] | ALA16013.1/0.0 | A2M_N_2(SM001359);A2M(SM001360);Thiol-ester_cl(PF10569);A2M_comp(PF07678);A2M_recep(SM001361) |
| **CL2603.Contig1** | 3.7778 | EFEFQSGDLWEVR | 1 | MSDKTRANKSGLGYAVEKKMEDNYDREEAAGTPTHVVNWVNGILGSEHDPIPGTDWKSICNHLRDGVALCKMVNILLKKDGKSPITFQKKVMSPFVAMTNIENFNKGCLDYGLDREFEFQSGDLWEVRKGPFLNVINCIHSLGFVANKKNVVPGYTGEIRKYLDNE | transgelin-like protein-3 [Mytilus coruscus] | AKS48154.1/8e-109 | CH(SM000033) |
| **Unigene12955** | 2.0866 | DMVMITGILMVLLMSGLVNSNDCPK | 1 | EKILSVSATRDMVMITGILMVLLMSGLVNSNDCPKYNLSSSDQLLIDMIQHYLYSSRRTEVKAHSNTGFGTVYTRWGRKTCPRSAELVYTGQVGGMQHNVQGGGVNKLCLPNDPENGQAYSYNNDALYGSEYQLYDSAKPSGLSTSLYNKEVPCAVCRRPQVSSIIMIPARKTCYRGWNIEYNGYLMTEHKAHYRTDYACIDMNAEPLDDSNGNEDGVLFYPVRTKCGSLRCPPYKNETEVYCVVCSK | hypothetical protein AM593_01958 [Mytilus galloprovincialis] | OPL20968.1/1e-87 | — |
| **Unigene17542** | 2.0866 | SIAIHQR | 1 | MKLLMLSLVIFAALALQVRADGQCTPNTSSKNHDDPHDDNHKDDQHGDDHHDDDHHDDDETMHYAQCEMEPNPHMASNLHHHVHGSIELSQKGHGAVYLEVHLVGFNTSEDHADHHHGLHLHMLGDMSAGCDSIGDLYNAHPEKHANPGDLGDLVDDDRGVVNEVHHYDWLDIDGTAPNTEALIGHSMTILQGSHKDPDTPASRIACCVIGHGKARPKTAAALHHELEEDKTEHYAHCDVRSNTHQPKALHHHVHGTIDMKQVGYGDLEVTYHLEGFNVSDDYKDHLHDVQIYTNGDLTSGCDNLGAKYDPHEDYHSDLGDLGDIHDDDYGVVNESHRYSWINIFGDDSVLGRSIAIHQRDHLHTSAKIACCVIGRGQSHPEIVHKAKCVVRPNTESTGLHHHVTGNITFEQTPGGATHMTADLTGFNVSEDLSHHRHGVQLHEWGDMSNGCHSLGRMYHGHDDPHDPKRPGDLGDVIDDSNGDVHATRALDHINVEDLNARSLVIMQGGHEVESERVACCVIGRA | pernin precursor [Perna canaliculus] | AAK20952.1/0.0 | Signal peptide(1-20);Sod_Cu(PF00080) |
| **CL4656.Contig1** | 3.7778 | LNDQIGGYEGELANLR | 1 | MSQEKVEVRTRNTKTAQTMGPRSTIITRHSTSGTLPMAGTRSSTFRASYGGGGFGGAASFATGTVSGMSQKNVANVLDTRAKEKNEMNVLNERFASYIEKVRFVEAQNKALLAEIERLKKQKNFDVSEIKELYEQEIADSRKIIDDLSDEKAKFDSTLVSLQDQLEDEKRDRINAEKTVDDLRNKIDRLNDQIGGYEGELANLRLRIDSLEDENARLKKDKKTLQDDIARIRADLDEETCKRIQAEMKLQTAEEDFKFNQNIYEAEIAELRAMLDKDKSIEMKDIWKGEIQKAISELQAQYAAELDRMQGDMQKNFEMQLNEMKAGVNRDNMEALSAREESKKVKGKLSELQPLINQLQAENAMLKSRLDALQIQYDDECREHEEDRLKLESQIQKLSSELESILRELQILQDAKLSLELEISCYRKLLESEEQSLKRVVEESSGARSSGAQLLSDMIVTKGGSEASQKSSMSTSSRKVNLVKNSRGDLRFERCDPSGTKVTIKNNGTKSISMRGWRLIKNINGVDKCKFNFADDYTIGAQREVTICGKVMADELEYGELLGDFNTWGTHGKFILFDDKNVEKASMDVQVL | retrograde protein of 51 kDa-like isoform X4 [Crassostrea virginica] | XP_022320110.1/0.0 | Filament(SM001391); SCOP(d1ifra_) |
| **Unigene17750** | 3.7778 | FNTIAECMNACR | 1 | MMWLPLGFITLAIFHGVNGQAANAKQKAAKTVGKMDKKCFDNPRTGMCMGMFQAKWFFNEATGKCVMSQGCFYQGFISMQECRKECQCRQPLNEGSGSGPVGANCELEVQKYAMVGEVCTPFMFTGCGGNGNRFNTIAECMNACREREPLDMMGEMMPGMMGWAGMSGMGGGMMGNMNFRGA | proline-rich protein HaeIII subfamily 1-like [Crassostrea virginica] | XP_022333931.1/ 2e-29 | Signal peptide(1-19);KU (SM000131) |
| **Unigene55006** | 2.7365 | IEFLEDTK | 1 | MNSLGTATLFLVVIWISVSEQGILPSRKNQVRSRRNYPERKPENIEKVLASGKENSGGGPASSGGQGNKHNVVIDQPPSNNGGLSNGNFGGHGAVVDDPTPNLGGPGNHDVSVADDPTPISGGGSPDKGNNNPESPKPEGGSPNGQGNNYDVSGPGDTAPNGLGNIHDAPVPDGNNNIAQPKDVGPQNGPGNNQDSPAASGGADPNGHGNNQDSPATSGGADPNGHGNNQDSPAATGGAETNGQGNNHDATIPASSGGGVQVDDHNTHIVGNAGGPVDANDHPLPRRTSRRRKKGRKGDKNENKEKRIEFLEDTKEMRDGAKATEDNEEDSEPQPVKPRKLRPKPTPAEKVINILTHNQPLPSSDPSTNQPSQQSESAASNPSPASGLVDNYPSPVSSHVNNNPSPSKPKEKARLFLFGDWSDCDSSDCWSSESSDSEKGSSSEKSTKVDSNIIESKLKFLRSRPKALIKMAAKDIDDISDKSSDCNSSDESDCEYWYSSEESVANDNGRRSSSKKNWNKKGSSESFPRGRKYGSRESYEKNSDGRDYSSDSSGWYDYESYEDDWSSEGRRNKHGDNRKTKTNGDSNNNDNNNDNNHENGNNNENNHDNGNAKNNHGDNGKGKRVERKRKLNFQDWDSYEYSDYSDSYESSDYKWHSKDSAEKTEKRNKEKKSKKGWYYDSSEYYSDSYDYESDDSGYYSDSSEWYENDDIFHKK | — | — | Signal peptide(1-19);Internal repeat 1 |
| **CL4205.Contig2** | 2.1572 | ILFQEFR | 1 | EAMTIMGICPEDQAALLRVISAVLMFGNMQFKQERSSDQATMPDDTVAQKSCHLLGLSVTNMTQAFLRPRIKVGRDFVTKAQTKEQVEFAVEAIAKACYERMFRWLVTRINRSLDRTKRQGASFIGILDIAGFEIFKMNSFEQLCINYTNEKLQQLFNHTMFILEQEEYQREGIEWKFIDFGLDLQPTIDLLEKPMGVLALLDEECWFPKATDKSFIEKVKSQHSTHPKFKKPDFRADADFSLIHYAGMVDYCASNWLTKNMDPLNENVVSLLQNSSDPFVAAIWKDAEIVGMGATAGGDTMFGSRTRKGMFRTVGQLYKEQLAKLMATLRNTNPNFVRCIIPNHEKKAGKIDSPLVLEQLRCNGVLEGIRICRQGFPNRILFQEFRQRYEILCPNTIPKGFMDGKKAVEKMIKALELDPNLYRIGQSKIFFRAGVLAHLEEERDLKLTDVIIQFQSLCRGLLARRNYQKRLQQLSAIRVIQRNCAAYLKLRNWAWWRLFTKVKPLLPVTGQEEKLTVKEEELRKVSEAFDKSKNEVQELERKYAQIIEEKSILAEQLQAETEMCAEAEESRARMAAKKQELEDILHDLELRIEEEEDKCNQLIDERKKFQQTVNDLEEQLEEEEQSRQKLQLEKVSADSKIKKLEEDLAIQDDSNQKLTKEKRFLDERVSELQTQLVEEEEKSKQMTKLKNKYEQIIKDLEEKLRKEQQARQELEKIKRRLETELNDLREQLNEKRQQLEDLQYQLSKREEEVQHALQNAEEESVGKAATMKQVREIQNQIQELQEDLDAERESRNKAEKQKRDISEELEALKTEFEESLDATAAVQELRNKREDELRDLKKSLDDAQKKYEGNLYELRSKYNQQAEGLNEELENVKKSKSSLEKMKQTLEHENSDLANDLKAVQMAKQESERKRRQLEQNVQELNVKLTEVERMKGDSSDRATKLQNELDQINSQLEQSDAKLLQTNAKNSSLEAQLTEINEQLQEETKGKLSVQSRLRQAEDDNRALKDQLEEEEDQKMALQKQISDLQFKVTEYKKKAEDETKVQEALDEYKKKTDREMEAMNAKLDEMKAVNDRLEKSKKKLQQEVEDANVELESQRSSFTQMERKQRKFDQMLAEEKAVSEKLALDRDQAEKDSRDKETRILNMARELDDLREQYDKQRSLTVSQQRELDDLMSSKDDVGKNVHDLEKAKRTLDAMLEEQKQKIEELEDDLQTTEDAKLRLEVNMQAMKAQHDRDLLAKEETVEEQKKSLLRQLREMEAELEDERKQKASAVNSRKKLENDIKDLQQQAELAVRVKEDAVKQLKRCQAQMKDNMRELEEARQSRDEMQAAMKDMERKLKNYEADLLKLQEDLAASERQRRNAEAERDELQDEMGSNAAGRSAFLDEKRKLEARIAALEDELEDEQTNSESLLDKARRSQLQLDQLTADLNSEKSVTQKLENQRTTLERQNKELKEKLNELENTLRTRTKATIASLEGKISSLEEQLDIESKERSGMQKVNRRLERKLKEMSVQAEEERRHADQNKEQADKMMNRVKALKRQVDEAEEEITRLNAQKRKIQRELDEQMEQNESAAREISQLKKYRPSASRAGARSTVLSSRIADLDDDDDQDETKENAEA | myosin heavy chain, non-muscle-like isoform X3 [Mizuhopecten yessoensis] | XP_021350591.1/0.0 | MYSc(SM000242);IQ(SM000015);Myosin_tail_1(PF01576) |
| **CL4276.Contig1** | 3.7778 | EDSYEETIR | 1 | MNDYNQSEKDLTMNDYNQSEKDLTMNDYNQSEKDLMDHMQLSQDSVSSYQLSQESVHSNVSQDLTSELLEGEVNETDKPKVRKKKSGPKKVKKKKSENTGDEAEEDKPKKKKKKVKITKRPEPEGGNAQPSLINPMTEFDRVIYSNEPLFSDLDMSDNENTNDMPERPTRAPRPGGVMIPCLQLGMHSNTMIKFAIIGSEIQNLLRVSLIRHEQEIQSLTRKISLLEEDIMKAEERFTTASGKLEEASKAADESERGRRALENKTFLDDGRIEQLESALKQTELIANEAERKYDEAARKLIVAETELERTEEKYDHMRRQVKTLEEELHLATNNLRGLEISEEKASQREDSYEETIRDLTNRLKDAEYRAETSERT | tropomyosin isoform X12 [Crassostrea virginica] | XP_022332784.1/4e-70 | Internal repeat 1;Tropomyosin_1(PF12718) |
| **Unigene20294** | 3.7778 | MGHAGAIIAGGK | 1 | PVLTMSTATKVLGKVSAIGRVGIRTCYTNSRPNLGINKKTKVICQGFTGKQGTFHSQQAIEYGTKMVGGVSPGKGGQKHLGLPVFNSVKEAREQTGADASAIYVPPPFAAAAIIEAIDAEVPLIVCITEGIPQQDMVKVKHKLIRQSKSRLVGPNCPGIIKPGECKIGIMPGHIHKRGKIGIVSRSGTLTYEAVHQTTQAGLGQSLCVGIGGDPFNGTNFIDCLEVFLQDPQTHGIVLIGEIGGQAEEKASEYLRNNNCGSDAKPVISFIAGVTAPPGRRMGHAGAIIAGGKGGADEKIEALREAGVDVTMSPAQLGTTMAKAMSAAGKL | succinate--CoA ligase [ADP/GDP-forming] subunit alpha, mitochondrial-like isoform X2 [Mizuhopecten yessoensis] | XP_021357525.1/ 1e-168 | CoA_binding(SM000881);Ligase_CoA(PF00549) |
| **CL3650.Contig2** | 3.7778 | YGGITCPNTCEDYAR | 1 | MKCKLLALAVPIVFLFGCIIAEISVKCLQCICNVESGCNQKENCNSNGGYEACGAFHIYEAYWIDCGKPGSSFKNCTLDYECSKDCVMKYMKRYGGITCPNTCEDYARMHNGGPYGCQNNALSSIKKNFAVIPRVAFLI | lysozyme [Mytilus galloprovincialis] | OPL33781.1/2e-35 | Signal peptide(1-21);Destabilase(PF05497) |
