# Supplementary material for: Microstructure and in-depth proteomic analysis of Perna viridis shell
Source: PLoS One. 2019 Jul 19;14(7):e0219699. doi: 10.1371/journal.pone.0219699 (PMC6641155; doi:10.1371/journal.pone.0219699)
Supplement: S2 Table — (DOCX) [file pone.0219699.s007.docx]

**S2 Table**

| **matched unigene** | **score** | **matched peptides** | **number of matched peptides** | **protein sequence** | **Homologous protein [species]** | **Homologous ID / E-value** | **Domain and signal peptide(SP)** |
| --- | --- | --- | --- | --- | --- | --- | --- |
| **CL1011.Contig5** | 21.6459 | GAPGPAGPR;GPIGPEGPSGQGLPGPQGPPGR;GPMGPEGPQGPPGLAGPR;GQPGPQGSPGLR;GSPGLGGTSTR;MGAVGAPGR;YPPYGNSYGPSAYQQR | 7 | GPPGKDGRPGMRGLTGKPGNAGIQGPIGPPGPQGPPGKDGRPGMRGLTGKPGNAGIQGPIGPPGPQGPPGKDGKVGLQGAPGVAGKPGTNGVPGMRGLTGRPGKQAIPIKAAAIPGPPGPIGPQGLIGPAGPKGANGRPGMRGLTGRPGAHGNDGKAGPPGKDGLPGKPGPPGAAGRPGMRGLTGRPGAGKDGPQGPPGKDGAPGRPGMRGLTGRPGAGKEGPPGKDGAPGKPGQRGIRGWRGWRGFKGAPGPAGPRGPMGPEGPQGPPGLAGPRGSPGLGGTSTRQTVVRGPPGPPGPQGTRGPIGPEGPSGQGLPGPQGPPGRGQPGPQGSPGLRGATGSPGLQGKPGIPGKPGAPGMQGLPGPIGNPGPPGAGVKGMGKEQVAVQGPPGPPGPPGAAGRPGKDGHPGVVGPMGPRGHPGEPGMMGPHGPPGKDGKPGPKGDSGAEGPPGQVGPMGPAGPQGKAMQATHHDIVQGLPGPPGPPGERGQVGIPGPRGYRGHPGAKGDYGARGPPGRMGAVGAPGRMGQPGPPGRQGLDGAVGPIGKPGPKGECSCGGPPMGAMRPPMGAGMGPRMGPGPYRPHMPYMAPKTPHTGPPPGYGWSQQRYPPYGNSYGPSAYQQRSNSLIGGTGKPSGSWRKKPVDTEAEMK | collagen alpha-1(II) chain isoform X2 [Otolemur garnettii] | XP_003793620.1/2e-35 | Collagen(PF01391) |
| **Unigene10181** | 131.0288 | ANLEQEVYR;AQSMIDEAEQR;CDELGAENAQLR;DLDSDVSTSTR;DLENELEADQR;DLENELEADQRR;DNLAAALR;DTEEALRDAEAK;EKDEEIDSIR;ELEDALDSER;ELEGALDNANR;ELELQLEETQR;ENGQLQAALR;EVVQQADDDR;EVVQQADDDRR;GSSPGTQNRLEGR;HQEALNDLTDQLEHMGK;IAIQQELEDAR;IDALEGSNGR;INVDDLTR;IQELEDNCEQLR;IRDLENELEADQR;IRELEDALDSER;LAAAQAALNQLR;LEEAEAFAQR;LEEAIGSSTTFSEVSR;LTAEIR;LTQENFDLQHQVQELDGANAGLAK;MEADIAAMQSDLDDALNAQR;MIEEAEDVANITMNK;NAENELGEVSAR;NRELELQLEETQR;QLEIEIR;QLENDNAALQK;QNLQVQLAALQSDYDNLNAR;QVAELTSLTDQLTMK;RMEADIAAMQSDLDDALNAQR;SLLEHAER;YEEESEAASNLR | 39 | MSLLRDLDSDVSTSTRIVRHTYNVYRGSSPGTQNRLEGRIRELEDALDSERELRLRYEKQSAELTFQLDQLSDRLEEAIGSSTTFSEVSRKREAEVSKVRKDLELASAQFEATEANMRRRHQEALNDLTDQLEHMGKAKARAEKEKNQLIIEIDSLQGINDGLQKAKMSADSKIDALEGSNGRLKINVDDLTRQLNDANSAKARLTQENFDLQHQVQELDGANAGLAKAKAQLQILCDDLKRNLDDESRQRQNLQVQLAALQSDYDNLNARYEEESEAASNLRAQLSKVNADYAALKTKYDKELIAKQEELEEIKRRLSVRIQELEDNCEQLRTRCNSLEKTKNKLTAEIREITIELENTQIIVQDLTKRNRQLENDNAALQKRCDELGAENAQLRNDKANLEQEVYRLKVANAELAEKNGNLERENGQLQAALREAQNELKSANRQINELTALKAQLEAERDNLAAALRDTEEALRDAEAKLAAAQAALNQLRAEMEQRLREKDEEIDSIRKSSARAIDELQRTLVEVETRYKTEITRIKKKYETDIRELEGALDNANRANAEYLKQIKSLQNRNRELELQLEETQRQLDDARNQLSISERKRIAIQQELEDARSLLEHAERARKNAENELGEVSARLTEVQLQVTALTNDKRRMEADIAAMQSDLDDALNAQRAAEERADRLQAEVNRLADELRQEQENYKNAESLRKQLEIEIREITVRLEEAEAFAQREGKRQIAKLQARIRDLENELEADQRRLREAAASARKFERQWKEVVQQADDDRRQVAELTSLTDQLTMKCKTYKRMIEEAEDVANITMNKYRKAQSMIDEAEQRADMAEKNLTAVRRSRSMSVSREVTRVVRV | paramyosin-like isoform X3 [Crassostrea virginica] | XP_022322570.1/0.0 | Myosin_tail_1(PF01576) |
| **CL1886.Contig2** | 122.8763 | AEMEAILVELQSLMDAK;ASVGGGSGGTVYTR;ASVGGGSGGTVYTRTVEYGMGR;DIQNEYDNKVDQIR;DMQDLNER;DTTEENREFWK;ELAALAYR;ELDGQREVVYTFR;EVNLDGWK;EVVYTFR;EYESMQSEHTMETVK;FITLENTSSQR;FLEAQNR;GAAQLSEMITEYETK;GSAQEAGINDLVFR;IDLNNETLNHLDAENR;IDLNNETLNHLDAENRR;KLIDDLTR;KLLESEESR;LADLEAR;LEIQNSSLQEEMNGLR;LLESEESR;LNQQLSDYESEINMLR;NAQLEAQYNSLLR;NVIEQSMNTQSR;QTLEEEMEFLK;REVNLDGWK;RTISSLETER;SMYEQELAEAR;SSGLGNLSPGSYEK;SSIGPSMR;SSIGPSMRASVGGGSGGTVYTR;SSTVVNR;SSTVVNRSSIGPSMR;TISSLETER;TVEYGMGR;TVITSSSSNYDDGDDSIYYK;VGMRNVIEQSMNTQSR | 38 | MSKSSERITEKRTVITSSSSNYDDGDDSIYYKSGIQPRSSTVVNRSSIGPSMRASVGGGSGGTVYTRTVEYGMGRSSGLGNLSPGSYEKVSNTGVMTVKSSREKEKKDMQDLNERFANYIEKVRFLEAQNRKLAGELEHLKTKWGKETSAIKSMYEQELAEARKLIDDLTRDKNKLEIQNSSLQEEMNGLRRQMDDLKKYHALDQEQINKLNQQLSDYESEINMLRRTISSLETERARDKDRINKLQGEVDRLRIDLNNETLNHLDAENRRQTLEEEMEFLKKVHEQELKELAALAYRDTTEENREFWKSELSQAIRDIQNEYDNKVDQIRGDMESYYNLKVQEFRTGATKQNMEVTHVKEENKKLVKSISDLKGRLADLEARNAQLEAQYNSLLREYESMQSEHTMETVKLKEEITNLRAEMEAILVELQSLMDAKLSLELEIAAYRKLLESEESRVGMRNVIEQSMNTQSRGAAQLSEMITEYETKGDSHSSMKMMRGEVSAKTTYQKTSTGPVSIAEVNPEGKFITLENTSSQRREVNLDGWKIRRELDGQREVVYTFRNFTLKPHKSVKIFARGSAQEAGINDLVFRDEETWGVGSQVSTCLVNEKGEEKATHRQRTAYN | filament-like protein-2 [Mytilus coruscus] | AKS48133.1/0.0 | Filament(SM001391); LTD(PF00932) |
| **Unigene2367** | 71.4714 | ACPAGTIYSASQCQCK;AIGSASGLLASK;ASLLLQSAR;ASNALTAGVSLK;CLIPGTLHYR;DISNGGLAFDHSHIYLR;GGSTSASGSVSAGGSVGSSR;GLGASSGFSSAGAAASASGLGSASLGSTR;GLGSVSVTTGGLGQDLR;GLNGIGGSVTSSLTSK;HGSISGSGISSLDSR;LLTALTK;LSFDNDER;MYLWGFQSK;SGSSSGSSGSSGSSGSGGSSGSSSFGSSTSGSDSR;SNMNGSGAMR;SRPEPIISNCGPNGDSSVEIVVYR;TGGNLEIR;TSDNPDTVYFNEK;YGYLAPEHGGLR;YKLPEQGGTLNILSQK | 21 | GAGAGAGSGVGLGFGVGAGAGAGSGTGLGVGAGAGAGAGSGIGQGIGIGAAGAGSGIGSGAGAGIGVGSGIGFGAGAGAGAGAGSGIGIGVGAGAGAGAGAGLGIGSGIGAGAGAGAGAGSGVEIGMGAGIGAGAGSGFGQGIGATSGLGSSSSGSWGSAGSGESSCSCASQGPKSETIQASAKLAVVGDRNVLIAKMPRAKVVDSDGLGDFLLEFPNLTIQPTLTEKIASPAELFGMNMNKGTHSTDDSDILVVALLPKGSKLGSKMFQPKSSGSGSSSGSSSSSGTGSSSGSSSSSASTNDNPSVSVDTSQDDGKLKIKIKAENVKDSTASIVKNQLDKIKSDEISVDTSKDDGKLKIKIKAKVDDLSKSDINALSSESSYSGSSSGSGSSGGSGSAGGSGSAGGLGSAGGSGSASGSGSAGGSSAAGGWGSNGGLGFSGDLGSAGSSTSAGGSGAASGSTGGSGSVGISGSISVSGSTGGSGINVGLGSAGALGTAGLSGSVGGSKLTVGLGNARGGSTSASGSVSAGGSVGSSRGSGSARAIGSASGLLASKIKASLLLQSARGLGASSGFSSAGAAASASGLGSASLGSTRGLGSVSVTTGGLGQDLRSGSSSGSSGSSGSSGSGGSSGSSSFGSSTSGSDSRLKIEKSKDDGKIKYKIKTVKGKGDSKTKSEYKLVVDKSDGSSSKVKVKFGKSDQDRSSSKIKHGSISGSGISSLDSRGKSYRREEDGDKWKLKSSGSRGLNGIGGSVTSSLTSKKQQIAEAVKVLKALKASQKASNALTAGVSLKAKLPDTRYKLPEQGGTLNILSQKLTKDIKFKQSRDNDERKKLSYKLSFDNDERKSRNRLLTALTKTSTNTVVPKTTGGAKVPNLEGLCLIYLGAFVDAIGYAPLPGQCHKLVQCFYLGGKLKAVARDCPAGMFWDQNTLSCRPPGDVLCFEDKCLIPGTLHYRRNGGCNCFYKCRDGISEPSCCPKGFRYDDDKGCVPAFGSLACDDECETPTTLTTQVTVSSCPSLPDPNNKYGYLAPEHGGLRIRACPAGTIYSASQCQCKSNMNGSGAMRGSLRKQYRQCSAEFNINFDDGFKDISNGGLAFDHSHIYLRKGKAVFGGNSRMYLWGFQSKYLGKTFAIKTRVKVNKGAGRSRPEPIISNCGPNGDSSVEIVVYRGKLIFKAKTSDNPDTVYFNEKYDDDKWMDITYYYDGNYFGGSCNGRSFKQRTGGNLEIRDNPMTIGLCSGKNPGFHGEIDELEIYTACIPKGF | matrix protein-1 [Mytilus coruscus] | AKS48137.1/0.0 | ChtBD2（SM000494; Pfam Laminin_G_3(PF13385) |
| **CL185.Contig3** | 30.3353 | AESPLPDDVLIER;APPAFQQPPPR;FINTILK;HPPPPHQPNQPPPPR;QPAPQQHR;QPQYQQPQQPPVAR;QQHPQPLPPQQPPR;QQQPQYPQQQPAYR;QQYQSSQQPYQGQR | 9 | PEVNLWNGSEMKLINVWTKVWTVFGLWNIAPTLGQGPMAMGGIGGASPSRGGKMTQKFINTILKLHNDYRRTEGASNMKKLRWSRALQRDAQLWANKCRYTHAYGKWGENLFKAESPLPDDVLIERAVNEWYYEKMSWKFTPDCNEACHYTQVVWAESEEIGCAYKRCTTLMLMEEFVMNGWMLVCYYNTQGNIIGKMPYSVGKACSACKPGYKCDRGLCDKQRTVHKYLPQHNQQKPKPVFRAPPAFQQPPPRHAPPPRHAPPPRHGPPSRHPPPPHQPNQPPPPRQQHPQPLPPQQPPRFRVSAPQPPPRPNSSLRPQYPPQHGQPAPYRPQQLANQPARPPSGSPPTNRPKPEPKPQPKPQEPQMQYISGKVLSGWIPAQTSKATPAPTNATFQQPQVQYQHQVPLKQQPYQHSNQAQPQRQQYQSSQQPYQGQRQQQPQYPQQQPAYRQPAPQQHRQPQYQQPQQPPVARQQPNQPQHQQHARPQSTRHSPYQQPYQQPHPQYNAQPAPAQQPQYPPAQPHNPYPDQPSHQPAPYVDPRPIYVPPAPAAPSSPTTTIPPPPPPTCLDGDKHCKHWGVHCKTNPYVHTNCRLTCNTCDIPTPPVHAKPIQTTQKSKAEPTIIPSRPPSPSPGRDHFHQPKPQHRPQPKPRRPPPQPQYNHYATTTAAYYPTTTGYNNQAPTTQTPYNAPGQGTSAAASSVASVGPHVGFCRDFDNRCKEWAKYCGMDEYVDDMCRLTCMRCKK | DNA N6-methyl adenine demethylase-like isoform X4 [Crassostrea virginica] | XP_022319021.1/1e-44 | SCP(SM000198);ShKT(SM000254) |
| **CL563.Contig3** | 29.9862 | AGFAGDDAPR;DLTDYLMK;EITALAPSTMK;GYSFTTTAER;IWHHTFYNELR;QEYDESGPSIVHR;SYELPDGQVITIGNER;TTGIVLDSGDGVTHTVPIYEGYALPHAILR;VAPEEHPVLLTEAPLNPK | 9 | MCDDEVAALVVDNGSGMCKAGFAGDDAPRAVFPSIVGRPRHQGVMVGMGQKDSYVGDEAQSKRGILTLKYPIEHGIVTNWDDMEKIWHHTFYNELRVAPEEHPVLLTEAPLNPKANREKMTQIMFETFNAPAMYVAIQAVLSLYASGRTTGIVLDSGDGVTHTVPIYEGYALPHAILRLDLAGRDLTDYLMKILTERGYSFTTTAEREIVRDIKEKLCYVALDFEQEMATAASSSSLEKSYELPDGQVITIGNERFRCPESLFQPSFLGMESAGIHETTYNSIMKCDVDIRKDLYANTVLSGGTTMFPGIADRMQKEITALAPSTMKIKIIAPPERKYSVWIGGSILASLSTFQQMWISKQEYDESGPSIVHRKCF | actin,adductor muscle [Crassostrea virginica] | XP_022325998.1/0.0 | ACTIN(SM000268) |
| **Unigene39912** | 31.7230 | GGPVIALETLADAPSEPR;ICDSTQTIASITDLSPHSR;INLVEPDYPYIVVVR;LADMEQATGELVR;LEWTSDFLDDDTTHVFR;QLQIMIIR;SLPIEGATQR;VGADVFETVITK;VGLGDAAITTYR | 9 | MLSIGLILVSLVSVSHGQWRQDMFSQAENKLRNIVQNGQILLDFIYQERQKHGGGNMTSGSMMSQNLAYSSFINDVEVRLADMEQATGELVRIMRTCPDAPLAPPSPTNVIVESTTTDNVSSIVVKWDPPFNPPENMQYKVYFVPVDINGMQTAGEVVFRICDSTQTIASITDLSPHSRYRIRVGAVAGSIAESTSVPLNVKTPDLIPSRVQNVMVKSSTPNTITLMWNPPSTMGDLVSYEIYYEENPINKMHVTVSPPENTFTIKDLSEGTTYKFEVSAKSDNGEGIRSLPIEGATQRFIPRAPQSFTGVALNKTAVKVTWTPPPPQPGDGIIRGYLINYTDVRYTDVSEHRVGADVFETVITKLTPAQVYYFRAFAFTKKSVGRGGPVIALETLADAPSEPRQLQIMIIREEPPKIGLTWLPPLHTYGNLLNYTLIWGVQNGANRTEYISPTRLEWTSDFLDDDTTHVFRLAAVNKVGLGDAAITTYRTPKKVPIIPPNVKVKRVTFENNGTTILNVTWDNPVVAVDGFRILYRKFQLVYSGRWELVEVFESNKKFVRINLVEPDYPYIVVVRGIPKGQIFNQYNMGGGQHMSRPNSHIAQSFGGASPPI | shell protein-6 [Mytilus coruscus] | AKI87977.1/0.0 | Signal peptide(1-17);FN3(SM000060) |
| **Unigene6649** | 24.8981 | ATDLLENNFFR;FFYENNFVPTGFTTAQLR;FIPPTYGDPSNIGTTPR;INSVIGVPLPSPR;IPSMPVNAFVSDR;NAFGAAAYR;NPALFGADGIGR;SGGALPDAGEFSR | 8 | KDMIKNCLSITIVLSLFPVLTYTQGDIGAAIADAVTEVGTRRPGGPALASAATGAGPPADVSGFGGVPGAGLAGVAAPGPADAATDPISIHALFSTRREAPGTVAAFGTAGDITTTATANFAARSGGALPDAGEFSRQIAPYCPVRRALCDPGAPYRTADGSCNNLNNPLWGASITNQARFIPPTYGDPSNIGTTPRINSVIGVPLPSPRIISNAVHRGGVSPPRSTIFNVALTHFGQFIDHDIISTPILRDGNEEIRCCTGAGVPVLRPECFSFFTPPGDFQTTCMHFVRSDFGPEPGCPPGPRNQINQRTSFLDLSVTYGNTRDGQNDLREFNTGRLLEGPGRILPEGPSGSECVGPSPCFKAGDNRPAEIPMLTVMHIVFLREHNDIVDGLRSMNPSWDDERLYQVAKKILTGIYQHIIYTEFLPIIIGQQGMDIFGLRSTPVGHSNSYNPTANPATRNAFGAAAYRFGHSLVGSFVAAYNIDLTPRATDLLENNFFRTDAIRNPALFGADGIGRWMTSQLKSRADRFLTPSVRDRLFQTAPGNGFDLSALNIQRGRDHGIPSYNRWRQFCGLPPALHFGTGPLGLINHDPSAAAALGSVYSHPNDIDLYAGALSERPAPGALVGPIFRCIIGLQFRLYKIGDRFFYENNFVPTGFTTAQLREIKSQTMSALYCRTIRIPSMPVNAFVSDRSGAPRIPCQFLRPLDLRPWRK | byssal peroxidase-like protein 1 [Mytilus coruscus] | ANN45955.1/0.0 | Signal peptide(1-23);An_peroxidase(PF03098) |
| **CL955.Contig7** | 23.0841 | CNMISGEVEELR;DLEESTLQHEAQISSLR;DTTDLALVAPGLK;EIAFQADEDR;ELEGELDSEQR;ELEGELDSEQRR;ESYNLAER;GQLEISNVR;IEELEEELEAER;LAEKEEEFENTR;LQGELEDLGIDVER;NLNADLDAAK;NSLQSIIDELR;QNVEEETR;RDLEESTLQHEAQISSLR;SSVSISR;SYSAELFR;VRELEGELDSEQR;YQQQVSEVQR | 7 | MSDLKIQVSTSVTSSKTEKPAVVKEEKAPEERIKTPDIISSPPAPEEAKVTTVKEETRRASASTLTPKMSDTSRITKTTRTSSIRTADYESTVGQLTKDYRGTSPAVLEGIASQPVLYSKAFDKIGQQKLSARSRKILRDTTDLALVAPGLKNLLEARIEELEEELEAERAARTKVDKQRAELARELEDLGERLDEAGGATSAQVELNKKREQELLKLRRDLEESTLQHEAQISSLRKKQQDATNELADQVDQLQKAKAKVEKERQQFRSECDDLQAQLQHYSKNKGVSEKMAKQLENQIAELQQKCDEANRNVNDLNSQKAKMQAENSNVVAQLEDTEHQIGSLSKERNSLQSIIDELRQNVEEETRARMKLQSDIRNLNADLDAAKEQIEEEQEGKADLQRQLSKANNEAQQWRSKYENEGANKAEELEEAKRKLQAKLQEAEQNAEAANAKVSSLEKAKNRLQGELEDLGIDVERANANANALEKKQRAFDKTIQEWQAKVSDLQSELENAQKEARSYSAELFRCKAQYEESQDSVDALRRENKNLADEIHELTEQLSEGGRNVHEVEKARRRLEMEKEELQAALEEAESALEQEEAKVMRGQLEISNVRSEIERRLAEKEEEFENTRRNHQRALDSMQASLEAEAKGKAEAMRIKKKLEQDINELEIALDASNRAKAELEKNIKRYQQQVSEVQRQVEEEQRQKEEVRESYNLAERRCNMISGEVEELRTALEQAERARKGAENELFEANDRVNELSAEVQSISSQKRKLDGDIQAMQSDLDEMNNEVRNADDRARRAQEDSARLADEIRNEQEHSQQIEKFRKSLEGQVKDLQVRLEEAESQALKGGKKMIAKLEQRVRELEGELDSEQRRHAETQKNMRKADRRLKEIAFQADEDRKNQESLNSMIDTLNAKLKTYKRQVEEAEEIAAINLAKYRKVQQELEDAEERADSAEGSLQKLRAKNRSSVSISRSSVTHTPSRTLLSTERSDI | catchin protein [Mytilus galloprovincialis] | CAB64664.1/0.0 | Myosin_tail_1(PF01576) |
| **Unigene43460** | 24.2891 | FLQAQVGPSGPR;GEDGESGGVGQVGPAGPPGEMGVPGDSGIR;GEPGPNGAAGQQGAR;GPQGDIGPSGER;GTMGEPGPTGPQGER;NGETGPR;SVYGGAMTSWFR | 7 | MKFGTVRWKNCIAPLFLFIVLVKSQEEDKANCLYEGTTYYHGDQWKPENCKWCVCDNGVADCKELLDCDGFGQITTGQESNTAIQGHQAIENEAEGSVGSPGRDGSQGLPGPIGDPGVNGKHGIPGPPGPPGVPPMSADQAYNRYFQQTYGQSFKAGGPAMGPRFLQAQVGPSGPRGSPGLPGQPGPQGADGVRGESGDTGPPGNPGLRGAPGAPGPPGLEGDSGRNGETGPRGLSGPKGPTGPAGMPGMPGMKGHRGLQGVQGPSGEQGRPGDKGSSGAPGAPGPNGPEGPRGSQGDRGSDGSAGPAGLPGVDGLAGAAGEPGPVGRTGPPGSPGLPGQKGEAGASGPKGSQGLQGSRGDPGISGPPGAEGMAGSDGLPGSNGEKGASGDPGPAGSPGFQGPRGPSGLNGSPGNAGAKGAPGQPGSPGFKGERGPKGIRGSGGDRGPPGAPGNEGKRGQRGTMGEPGPTGPQGERGSTGMRGYPGPIGDPGAAGEEGGIGPRGRRGEPGPNGVPGRMGPPGARGPRGGNGGPGIDGMAGRPGPPGVTGNDGRPGEMGAPGIPGPAGIQGVQGNPGTRGPPGKDGNPGAQGPRGPQGDIGPSGERGNTGPRGAVGEPGGRGPEGNGGAPGFVGAPGPPGGQGEPGKPGEPGPPGKAGKAGRPGSRGERGIPGVTGEPGAPGLSGVQGPEGGAGRDGERGAPGEPGGLGEPGPEGPAGRQGMRGPRGERGAKGEMGEAGLPGEDGREGRKGPSGPQGPPGEPGPPGEPNEKGSVGDLGLPGERGARGTPGDRGPQGTAGIQGEPGQPGMAGAPGPKGQRGQTGQKGEQGAAGIAGAQGAPGPTGRDGMNGRKGVRGDRGSQGLPGQPGTPGGVGPVGNAGPHGDDGPPGPPGEDGIKGSRGETGHVGRPGESGAPGLPGEPGLKGARGEDGESGGVGQVGPAGPPGEMGVPGDSGIRGERGNAGPPGRPGQPGDPGRAGLNGAPGNAGPPGPPGLAGPSGEVGHPGPPGPDGAPGLQGAQGEKGPDGDIGMPGAMGLMGFSGPPGPPGPAGPAGERGERGEPGPNGAAGQQGARGPPGPQGPQGPSGEKGSLGENGDKGDPGLMGMPGLSGPEGPVGDLGPTGPQGPPGQRGPDGRRGDPGSDGMVGPSGPPGPPGPRGPQGEDGRRGSMGEAGNPGPPGAPGRSVYGGAMTSWFRGSSGNKGWQGDEPVPAEEIDTDVFKALEEVTLQIEKIKNPTGEQDSPGRTCEDLRAHNPDIKDGYYWINPNLGPIYDVIKVRCDFRKKRVFTCVQPEVKTIENMNIAQKNDHTWISEVLGSKFDYDPSLFVKPQIKFLQYLHQKANQEIVYKCKNSVAIDDDKSIQLAGFDNSLLSSKGKRSIRYKIKKDNCKNKNGSWEKTVLEVNTKRTKALPIMDIGVYDIGGADQDFKIELGEVCFFN | fibril-forming collagen alpha chain-like [Crassostrea virginica] | XP_022341069.1/0.0 | Signal peptide(1-24);VWC(SM000214);Collagen(PF01391);Internal repeat 2;COLFI(SM000038) |
| **Unigene51088** | 22.3396 | EGSSDAQEIMQDR;QSLNNSPMFNSR;QTSLQLSSAR;SPLLSQDLATIR;SSFSQSSLPMMQSR;YIEIVHGNVHER | 6 | MYQEGIILIFFIFISHSNGQGCPHSATPVPGDPTRYIEIVHGNVHERNCAPGTEYNTFTCDCSVFSGASIQALMDGSAGSHPDLLNMPLETLQKEFLQLTSQRAPQSTRTDSRRTSKRRKSKTAEFQYNPKKKESQESILFGLLADSALLNLLPTNPPSKAVKHVEKPQFKSEFIDYSLLAPTKAPKIKTFSALNLPKVTPKPKLKSPLLSQDLATIRAFLKERREQRAREKALLKTKQSPITSSASTSSIGLSGTKNAFSFDSGLKILGSSTTKQSTASNLGLNDLRTSAAKSTSSLDIGFNDYHKAALASNTGGLNTNNVKSSVGIAVPTPDAGLNEFRSSLSLLDRNYPTPTPHSRDTFANVKTVVNPTDNFAALMERRKLEQALITKSEPSSSISGISNTASKSSNGLGGLDATKKAILKNLVARLNALEEKNKGTATSSQQQNSMMSQTSSNSDQMQGQSTSLSQRQSLNNSPMFNSRQTSLQLSSARSSFPQSRSSFSQSSLPMMQSRQTPFSMMFRGVAAKEGSSDAQEIMQDRITEMGGAAALAMGAPLSMINSEISSQLSSTMLENGPLPFLM | uncharacterized protein LOC111135614 [Crassostrea virginica] | XP_022341547.1/0.53 | Signal peptide(1-19);SCOP(d1dqca;chitin-binding) |
| **Unigene9058** | 20.6772 | EEQAEADGTAEAEK;ENQSILITGESGAGK;IAGADIETYLLEK;IEDMANLTYLNEASVLHNLR;LQQFFNHHMFILEQEEYK;NLYSTHPHFVR;VTFQQAAER | 6 | MTSGISPDDPDYQYLCVDRKALMKEQTVTFDGKKNCWVPDEKLGFVAAEIQSSKGDEITVKTVEKMEMRTVKKDDIQQMNPPKFEKIEDMANLTYLNEASVLHNLRSRYGSGFIYTYSGLFCVVINPYRWLPIYTDSIIQKFKGKRRSEMPPHLFSISDNAYQFMLQDRENQSILITGESGAGKTENTKKVIMYFAKVAASLGKKDKEEETAAKDKKGNLEDQIIQANPVLEAFGNAKTVRNNNSSRFGKFIRIHFGPTGKIAGADIETYLLEKSRVTFQQAAERDYHIFYMLLSNAFPKYHEMMLLTPDPGLFSFINQGALTVDGIDDVEEMKIADSSFDILGFTEEEKTSLYKCTAAIMHMGETKFKQRPREEQAEADGTAEAEKAAFLLGVNAGDLLKSLLKPKIKVGTEVVTQGRTKEQVVYSVSAMAKSLYDRMFKWLVMRVNQTLDTKNKRNYFIGVLDIAGFEIFNFNTFEQLCINYTNERLQQFFNHHMFILEQEEYKKEGIQWEFIDFGMDLQACIDLIEKPLGILSILEEECMFPKADDKSFKDKLFANHLGKSPNFGKPGAASKGKGQSDFELHHYAGIVPYSTVGWLDKNKDPINETVVELLSHSKEHLVQTLFAPAKEEPGTTHKKKKSSAFQTISAVHRESLNKLMKNLYSTHPHFVRCIIPNEMKQPGLIDAGLVLNQLQCNGVLEGIR | myosin heavy chain, striated muscle-like isoform X6 [Crassostrea virginica] | XP_022317649.1/0.0 | Myosin_N (PF02736);MYSc(SM000242) |
| **Unigene37646** | 15.0703 | LTWACESPCR;LVCYDQCR;MDGEENEYEGPTSMSNR;RMDGEENEYEGPTSMSNR;VSFWYQCK | 5 | VSFKFLSFFLLLMTVTGQRRRPISKTDDRVSFWYQCKQECIRLTWACESPCRNYAETRMAYKMCALECKLDRLVCYDQCREFIAQGLVWLASPSNNNKRNRRRMDGEENEYEGPTSMSNRKDFMAFLNYILES | P,N-U7 [Pinctada fucata] | AKV63173.1/0.71 | Signal peptide(1-17) |
| **Unigene51075** | 18.4428 | LASDLLEWIR;LENSFNTLQTR;NINEVENQILTR;QGLEEAER;VGWEQLLTAIAR | 5 | MEDYPSDGYMDEEEEWDREGLLDPAWEKQQKKTFTAWCNSHLRKAGTQIEDIEEDFRNGLKLMLLLEVISGEQLPRPDRGKMRFHKIANVNKALDYIASKGVRLVSIGAEEIVDGNCKMTLGMIWTIILRFAIQDITVEELTAKEGLLLWCQRKTAPYKNVNVQNFHLSWKDGLAFCALIHRHRPELIDYYKLSRENPLENLNTAFNVAEQHLDIPRMLDPEDMVNSAKPDERSVMAYVSSYYHAFSGAQQAETAANRICKVLKVNQENERLMEEYERLASDLLEWIRKTTPWLENRTTDNTLPGTQRKLEEFRDYRRKHKPPKLEDKARLENSFNTLQTRLRLSNRPAYLPTEGKMVSDIANAWKGLELAEKGFEEWLLSELQRLERLDHLAQKFRHRCEIHEEWAEGKEDMLQSQDYLKCRLNELKAMKKKHEAFESDLAAHQDRVEQIAAIAQELNVLHYHDVQSVNTRCQLICDQWDRLGTLTAQRRQGLEEAERILEKIDQLYLDFAKRAAPFNNWLDGAKEDLLDMFIVHSIEEIQDLIEAHEQFKGTLGEADKEYNSIMGLANEVQRLAQQYGLTLKENPYTTVSPQEDIANKWGEVKQLVPKRDRTLHDEKIKQENNERLRRQFAQKSNVVGPWIENQLDGVASIGVTARTSLEEQLNKLRQFEKATESYRVHMDELERYNEEVQESMIFENRYTHYTMETLRVGWEQLLTAIARNINEVENQILTRDSKGISEDQMNEFRVSFNHFDKNRTRRLEPKEFKACLVSLGYNIRDDRQGDADFQRIMSIVDPNNSGYVTFEAFLDFMTRETADTDTAEQVMQSFKILAGDKPFITAQILRQELPPDQAEYCIQRMAPYSGRDAVPGALDYMSFSTALYGESDL | Alpha-actinin, sarcomeric [Crassostrea gigas] | EKC43084.1/0.0 | CH(SM000033);SPEC(SM000150);EFh(SM000054);efhand_Ca_insen(SM001184) |
| **CL1804.Contig1** | 13.5045 | DYPLMDIPVHQPFR;GIPGPESSLLNPELLR;NIDGYSNVFTQYIYR;QPGSATGLIQDR | 4 | DALALLHQDDAIPSAHDGPNFLSWHRYFLVLFEEALRTKNPKMALPYWDSTLDFRMVDPSKSIYWTEIFVGNPTGVVYTGPFADWITPANNTLLRRGIPGPESSLLNPELLREVFKQKYHYQILFPTNVSLHSNFETHHDGVHRWIGGQDGHMGGLSTSPQEPAFWNFHCHIDYLWEQFREQQRRRGIDSSRDYPLMDIPVHQPFRKMDNLWPPLRNIDGYSNVFTQYIYRYEAAPTCRNKCGGAKTEYLYCNRRINQCVSRSRSTFERLTGIGRSRQPGSATGLIQDRVASLQPERNVSLSSLADRPNTEPKFRKAIVDPRTT | tyrosinase-like protein-1 [Mytilus coruscus] | AKS48166.1/3e-98 | Tyrosinase(PF00264) |
| **CL3395.Contig3** | 11.2211 | GVPDEEIESR;HVTMDTTR;MALPETEYIYER;MEHSGGEGELVAEYEITR | 4 | MSLGSIPMADSGICHCSAEWGMAEIIKGESKVAAVTTMSDDDTAVLAGVLAGLATFLFLALPILCCLCPLPFACCGGGGGGAGKKGAAAVGAQRRKNIHEFSSGRSTDVESIGSYRSWDKNWDKLDRFDHDNLYEVDMKLPRAWLESLRGVPDEEIESRLREMSRDGGWEGNDIKQSQEMELRREMYEESGSHGIGGMGTMGTDGSRYATAQRMEHSGGEGELVAEYEITRHVTMDTTRMALPETEYIYERDIRGENNIADRDNFKRIYYTYQRIKGDDSKQTSF | hypothetical protein AM593_02533 [Mytilus galloprovincialis] | OPL21563.1/2e-80 | — |
| **CL4671.Contig1** | 15.5873 | GPGGPVAVPQR;GPVLLNIDTVDAASGQR;SGPASVAGVSAPR;VFAGTQEDAAVTAEASR | 4 | MLPVVLLISLFAAGTLGTGYVPPPKLPKKVVSLPIPKPVLKGKPLRQDTAFDHHQIVPIKKTEVRRVVEKVPLVHEVPIFITKNKPFYQPQVVPKPIVVHEVVGLPIVRDKHVVHPVYIDRPYIVEKKQFVEQPFPVEKPIPIDFIKRITKVYDRKVEVPHIVNIPLVKTVDRPRAVPYTREVVQHYDVHVNVPRPRPVKVLRHKTHVEKVPFETPNLIVRKNPVHHAVNEYIPKVPQGFNGRVFAGTQEDAAVTAEASRFGPGSGGRTISVSRGPGGPVAVPQRGPVLLNIDTVDAASGQRIPDGAVVARGARSGPASVAGVSAPRQGGAPVLLGQDLSGPGVGSLGPAIPGGPGPVGVEAVAVGGPGPVGGPGPLVGGGIGGAAVVVGGGGGGIGGGAGVVAVGGGGGGVDLGAGPVVVDGGRGGGAGPIVVSGGGGGGGVDGGFEIVDVGANGEGIINIGPGGLGQDIEIVAVGPDGKDIGSFVVGAGDIAGGTGKKGKK | valine-rich protein-like isoform X4 [Crassostrea virginica] | XP_022307204.1/3e-09 | Signal peptide(1-17) |
| **Unigene20161** | 12.3863 | GMTGFGAIR;GMTSFGSQR;NLPMVLATISHVGTEAQR;YDPAVESEVR | 4 | MADRVKPMGMDRALISKMGAKYDPAVESEVRGWINQLIGEDIGEGPSNLEKGLRDGVILCNLMKKIIDGTPSESLPPACNKLNLTPSPSELPFKQMENIEKFLKAAHAYGVPNTSLFQTVELYEARNLPMVLATISHVGTEAQRHNYNGPTIGSKPTEKHRVQFTYEQLKNSHGTIGLQSGTNKFASQKGMRIGAIRHISDIRADDLDKEGTTLLTLQAGTNRFASQKGMTGFGAVRHIADIRADDADKAGDNIITLQAGTNKFASQRGMTGFGAIRHVSDIKADEFDPNTQSHIGLQAGSNQFASQKGMTGFGAVRHICDIRADDLDREAQAEIPLQYGTNKGASQRGMTSFGSQRHIADIKVSDLAEDMKRQDLDMTPKEYQQYRRELEEAAKQQGQEVEEPQYE | calponin-like protein-1 [Mytilus coruscus] | AKS48134.1/0.0 | CH(SM000033);Calponin(PF00402) |
| **Unigene3051** | 13.8642 | AIQNTQNGMWAAGR;CQMIIGIPALPDLR;FTPLHTLLTR;QTGNFFVVDTVPGR | 4 | SIIPPDTKYQDIVVDNLSYSGRPGSGSVLIEPVPVSSSFGTLPDGVVIADSGSPAAGALPDGVIIEPVPEGSNLGGIQIPTGGNVPVLIKDDNLLIKPSPEKTVWTSEPILINDSNLIATTPSKEEYGQNLSGSGKGSTSAIKTTTSTSSVTIQSQEPSAVVTVNNGTKSNGDNEGTTWLVTPTLPPTTKAPVINPNSGTPKPLSCPDFDSRYENGKFIVTLDEGKCEMVVSERAIQNTQNGMWAAGRFTPLHTLLTRKYKPEINFRSTTLAPTGYPCLHKTRQTGNFFVVDTVPGRCQMIIGIPALPDLRNLLTQRQQKVPTWNKPRQMPTWPRRRPSKEREDNESSD | uncharacterized protein LOC111131742 [Crassostrea virginica] | XP_022335121.1/5e-07 |  |
| **Unigene34861** | 13.0074 | CLALATIDNPPR;EFDDLSIEEQR;IGVFGENESTEGLSFTGR;ISTIHSTTLR | 4 | MAGKNAESAIEALKEYEPEIAKVVRKNHRGIQRIRASNLVPGDIVEVSVGDKVPADIRISTIHSTTLRIDQSILTGESVSVIKHTDPIPDPRAVNQDKKNVLFSGTNIAAGKCRGIVIGTGLNTEIGKIRDEMMDTETEKTPLQQKLDEFGQQLSKVITIICIAVWAINIGHFNDPAHGGSWVKGAIYYFKIAVALAVAAIPEGLPAVITTCLALGTRRMAKKNAIVRSLPSVETLGCTSVICSDKTGTLTTNQMSVCRMFTFAKIEGNDVKTDQFEITGSTYAPEGDIYKDGKKVLSGEFPGLEELATVCVMCNDSSVDYNDTKNIYEKVGEATETALTVLVEKMNYYNTDKTGLNKREKGTACNHVISQMWKKEFTLEFSRDRKSMSVYCSPNKPTRTAQGCKMFAKGAPEGILDRCTHVRVGANKVPMSPAIKNEIMKHVASYGTGRDTLRCLALATIDNPPRREDMDLEDSRKFIEYETNMTFVGVVGMLDPPRTEVMDSIKRCRDAGIRVIVITGDNKATAEAICRRIGVFGENESTEGLSFTGREFDDLSIEEQRRACMKARLFARVEPTHKSKIVEYLQGEGEVSAMTGDGVNDAPALKKAEIGIAMGSGTAVAKTASEMVLADDNFASIVAAVEEGRAIYNNMKQFIRYLISSNIGEVVCIFLTAALGIPEALIPVQLLWVNLVTDGLPATALGFNPPDLDIMKKQPRSTKDSLISGWLFFRYMAIGIYVGCATVGAAAWWFMVYDHGPKLNYYQLTHHSQCLAQDQRFQGINCNIFDSPEPMTMALSVLVVVEMLNALNSLSENQSLLAMPPWSNPWLLGAIALSMSLHFLILYTEVMSTIFQITPLNFAEWSAVLKISIPVIILDETLKFVARKFTDDYPNFF | sarco/endoplasmic reticulum calcium ATPase isoform A [Pinctada fucata] | ABS19815.1/0.0 | E1-E2_ATPase(PF00122);HAD(PF12710);Cation_ATPase_C(PF00689) |
| **Unigene39610** | 15.5873 | DGNAGGMSFSSGTGSGNGFAFGGTFNR;GGNGGGFVTSR;SRDGNAGGMSFSSGTGSGNGFAFGGTFNR;VNNGQGIAFR | 4 | MMKALAVLFFIIQVSHGSFGGPYGSSYYNPFQMDKFMYDFFTTFNNIMSMKAPAPRPKPQTFPGAQLFPPTFPDFSGKNSGFKTVLINDMKPGTRKTFKVNNGQGIAFRSRDGNAGGMSFSSGTGSGNGFAFGGTFNRGGNGGGFVTSRSGPKGTKVSYSKGIPKFAKNLFSSFSFF | shell mytilin-3 [Mytilus coruscus] | AKI87980.1/7e-40 | Signal peptide(1-17) |
| **Unigene483** | 11.8421 | AAAAAAASASASAGSGIGVASR;AALVQLVIK;LYAYDYYK;RAALVQLVIK | 4 | GFGGGFGGGAGAGAGAGAGAGAGAGAGAGASAAAAAAAAASARRAALVQLVIKARAAAQARAAAAAAASASASAGSGIGVASRFGGGFGGGAGAGAGAGAGAGAGAGAGGAGGASAAAAAAAAAAAAARNANLRGWQSANANSLAAAIAAASAGGGGGAGAGAGAGAGAGAGGGAGGGAGGGAGGGSGGSGGSGGSGGSGGSGGSGGSGGSGSAVRLYAYDYYKNSDDKKGPGYERS | — | — | SCOP(d1gkub1) |
| **Unigene48535** | 11.5174 | GLNSGGVNSGTR;MGGVNSGTR;TEFFIPR;YDTVNSMMCR | 4 | SLICILLLVKFALIAGQRQLPRTEFFIPRTQQNAKRVQQKFTQLERLKQLNNMSGMKFGNSGASGNGWNNNVGANGGRGLNSGGVNSGTRMGGVNSGTRMGGNRRQINLSNLNAEQLEVIMDQRRMQMEMGMGMGMGMSGGGMGAGNGGYTFINVEALNNAAEMGIMPSMFMGNNGARGQQGMLGNNNNNGKGGMNVGRGNGSRRNGMNLPLCQPLYECAENFIPPQCRTWRYDTVNSMMCRSCPINTCQGGAKEMFNTMYEHHPKRLLFEALTGK | — | — | Signal peptide(1-16) |
| **CL1023.Contig1** | 9.9300 | HQAVPIR;LSEISEPSNEVIR;STESADAVER | 3 | KNTKTFVVEDVVLPKFEVNVILPSIQLTTDSHFTATITAQYTFGKPVEGDVLLSIYSGSKRRGITKRFKINGKATIRVLMSEIAPRFRYFTVEAEVTEAVTGDKQGNAQRTHLYETQEKLLFSPTMPYTFKPGLDYNIILRATQENDKALTGYLGQVNVTVFYRVPKQKEEKTGALQCTEGMCPPSDETEEKVLWSKNIQIPESGLMKEVASFPINALSGQVQADYRMASDRKYLSKAQSPSSNYIQVTIVNDQKAKAGTNLPLMIKATEPVQYVNYKIFAKQYMMQQGTFDMQNTSSKQVQIAITTDMAPKVKLLVYYTRPSDGEIVAAAVKFPIEGIFDYEVTLRFNKERALPGDNVTLSVTADPNSLVSVLAVDKSVLLLRTGNDVTVKDVINELNNYDGNLYPNFGAWDYWFSRPISSIDASSVFKDMGVYVLTDSLLYKHSEVVRRSQSFNVPMNAEILAMDAPAMSFTSGGMDDLAKPSRTRKNFPETWLWTNTMTGASGISMINAKAPDTITEWVTSAFAVNPTSGLGVSSDIANLTIFQKFFMRFELPYSTIRGEIVIVQITLFNYLATAQNVQVKLNGGEGFSFVDANGDPLNTGSNGMTKTTLVKNDSVSSVYFPIKPTTVGKVTLSATARSTESADAVERELIVEAEGIKQSYNIPLLLEAQSPNQGTVIRTPVTFPPNRVPDSTFVKVQVIGDFLGLALANVENLLGSTSYGSGEQNMITFVPNVYISSYLKTTNRLTTEIKKKTERLMQGGYQRQLSFARIDGSFSAFGSNDPRGSTWLTAYVIKSFAQAAEFTYIDKKVITKAIKWLLTQQAHSGNFEENGIFIRKELQGGSTSSARSLTAFVLIALYEAKSNDQVEAEIKSNVDSAISKATQFVAEGAPASITNVYELAISFYALSLVKHASKNLLLVELEKKESIDGEEKFWRIPKTEADIIQPWKEWSPQNEEFRALDIETTAYVLQGYNLNDDTKNGTRILRWFGRRRRSRGGFKSAQDTAIALEGLSELAKKLYVPSTSLTINVKADNLAGRTFNIRDENSLVLQNEDITTLVDHIEVTTSGTGISLMDIDVYFNVMSELRVPAFNMTTALPKDSTKGFRLRICFSYLKDDESGMALLEISLPSGMEADLTSLDTSRTWGKFKKAEKAFRQINLYFESIQSRNMCVELDVNRVSLVARHQAVPIRLSEISEPSNEVIRLYKSKALSTATIIEVCGADNCQELRK | protease inhibitor-like protein-1 [Mytilus coruscus] | ALA16013.1/0.0 | A2M_N_2(SM001359);A2M(SM001360);Thiol-ester_cl(PF10569);A2M_comp(PF07678);A2M_recep(SM001361) |
| **CL678.Contig2** | 8.7988 | AAAAAGAAAAAGAGAGAGGSSGLSAALR;GILGWLLR;VVIQLLTR | 3 | GGLGGGLGGGLGGGLGGGLGGGLGGGADAELELFEDLLGTYGLDIFEGEEGLAALSLLGGLGAGAGAGAGAGAGLGLGGAGAAAAAAAAAAAAAGAGAGAGAGAGAGAGAGAGAGAGAGAGFGGAGGSAAAAAAAAAAAASARSRAAAAAGAAAAAGAGAGAGGSSGLSAALRSRLLARIAARRAAASAAAAASAAAAGGAGGAGGAGAGAGAGAGAGAGGGAGSGAGAGAGAGAGAGAGAGAGAGAGAGAGSGGARGILGWLLRRRAMARAAAAAAAGAGSGGSGGSGGNGGGSDGDCGDSDSDSGSDSNGDNDTDSSDSEGSDGSDSDSGSDPDGDGDSDSSGSSNSSDDNGDSGDYDSGDDGDGDDGGDFKAVAKVVIQLLTRVLSSGVLTAGASAGASASAGAGAGAGAGLGGGFGAGGGAGAGAGAGAGAGFGSGIGLGFGGGFGGGFGGGAGAGAGAGAGAGAGAGAGAGAGAGAGAGAGAGAGAGA | — | — | SCOP(d1gkub1) |
| **CL758.Contig1** | 7.4251 | LTGMAFR;VGINGFGR;VPVPDVSVVDLTVR | 3 | MKVGINGFGRIGRLVMRAAIDKGVSVVAVNDPFIDLDYMVYMFKYDSTHGCFNGTVEAKDGKLIINGNAVAVFGERDPANIPWGANGAEYVVESTGVFTTKDKASAHFKGGAKKVVISAPSADSPMFVMGVNEEKYTKDLTVVSNASCTTNCLAPLAKIINDKFGIIEGLMTTVHAITATQKTVDGPSMKDWRGGRGAAQNIIPSSTGAAKAVGKVIPELNGKLTGMAFRVPVPDVSVVDLTVRLQNGASYDNIKKAIKEASEGPMKGIMGYTEDDVVSQDFRGDNRSSIFDAKAGIALSETFVKLVSWYDNEYGYSCRVIDLLKHMSKVDSA | glyceraldehyde-3-phosphate dehydrogenase [Littorina littorea] | AJA37895.1/0.0 | Gp_dh_N(SM000846);Gp_dh_C(PF02800) |
| **Unigene12260** | 10.0281 | MTEHGLAR;QNGLGGGNPLICSR;RQNGLGGGNPLICSR | 3 | MDPYLFLCVLAIAFSVHNVYAQMGMGHQNQGNMRRKFPGRQAGMGGGMGGGMGTGNNNQQGMNLGGSPADMGMPGNGMGGQGQGQGHGNGGQGQGNGMNNMGGGGGSGGLMGGMLGGGMPMDAMMGGMQANMMAMGAMNSEIPPHMIMSGTFNPKNFAQYRSCEKTPSNLNTICDPSSPNPCPQGAMISKSTPFAMGMGMAMGGMGGMGMGGMNRRMNRRQNGLGGGNPLICSRMTEHGLARCCAKNMMTARMLDKWFK | — | — | Signal peptide(1-21) |
| **Unigene18932** | 10.0785 | FEELNADLFR;IINEPTAAAIAYGLDK;TTPSYVAFTDTER | 3 | LFGYKIAAASRGIHLKEKKRAYKNKKEQSPQNRNMAKAPAVGIDLGTTYSCVGVFQHGKVEIIANDQGNRTTPSYVAFTDTERLIGDAAKNQVAMNPVNTVFDAKRLIGRKFDDASVQSDMKHWPFTVINDSSKPKIRVEYKGEQKTFFPEEISSMVLVKMKETAESYLGKTITNSVVTVPAYFNDSQRQATKDAGTISGMNVLRIINEPTAAAIAYGLDKKATGERNVLIFDLGGGTFDVSILTIEDGIFEVKSTSGDTHLGGEDFDNRMVNHFIQEFKRKHKKDISENKRAVRRLRTACERAKRTLSSSTQASVEIDSLFEGIDFYTSITRARFEELNADLFRGTLEPVEKSLRDAKMDKASIHDIVLVGGSTRIPKIQKLLQDFFNGKDLNKSINPDEAVAYGAAVQAAILSGDKSEEVQDLLLLDVAPLSLGIETAGGVMTSLIKRNTTIPTKQTQTFTTYSDNQPGVLIQVYEGERAMTKDNNLLGKFELTGIPPAPRGVPQIEVTFDIDANGILNVSAVDKSTGKENKITITNDKGRLSKEEIERMVNDAEKYKDEDEKQKDRIGAKNSLESYAFNMKSTVEDEKLKDKISEDDKKVIMDKCDEIIKWLDANTLAEKEEFEDKQKELEKTCNPIITKLYQAAGGAPGGAGGMPGGMPNFGGAGGPTGGAGSGGSGGPTIEEVD | heat shock protein 71 [Perna viridis] | ABJ98722.1/0.0 | MreB_Mbl(PF06723) |
| **Unigene30013** | 8.7624 | GDIASGIGGGAIGGR;GTTGFAGAVSGR;TGAVSNFPVFGPGINR | 3 | GRTGAVSNFPVFGPGINRGFGSSFDGNFGAGFGIGPIGGGFPSFGGPASLASLNAALSGSINAATDGFPGFVGGPLGTLSGSLSGGLNAATGGFPGSIGGPGPLGTLTGALSGGLSAATTGLNAGLNAGLSAALRGDIASGIGGGAIGGRGTTGFAGAVSGRFAGTVGAGAGGAIGKGKVY | glycine-rich cell wall structural protein-like [Crassostrea virginica] | XP_022339053.1/4.9 |  |
| **Unigene35808** | 8.7624 | FTMLVQQLR;INQELEGSR;SFTLADR | 3 | MDPHLCTHIIYSFAKLNGNRLAPFEWNDESTEWMKGMYEKFNSIKQQNPRIKTLLAIGGWNMGSEPFTHMVKTTQSRQEFVKSAVDFLRQRNFDGLDLDWEYPANRGSPPKDKHRFTMLVQQLREAFDRDALTTGRSRLLITAAVAAGKKNIDSGYDVPALGRLLDFISIMTYDLHGSWESNTGHNSPLFARSGETGEQRYLNLDWAANYWNRMGVPKSKLNIGLGLYGRSFTLADRNVNNVGAVASGKGKAGKFTREGGFLSYYEVCEMMKSGGKKYYINEQKVPYLVKDDQWVGYDDVDSLSIKVQYVKQQRFAGIMVWALDLDDFKGSCGQGRYPLLKRINQELEGSRYQPDYSIMNAPSIPDILNQPLAPVAPPPRRRKQKPVRKPPVQTPILPALPPAHHTPLLPPVPRVDQHRTSSKDFTCRKGYDGYFASPDSCSKYYMCTDGTAFKFNCAPGLKFNKEHNFCDWPEKVKCTESTKKSSKKNKNRVQQALLPPNPPIRQPEPPRYEPPPPPQPPPTSANGWNFNANAHAATLPPPVPSSPTNSQQSSAWDWVSMIDNPMPFFMSLFGSNDLFADMCANKANGIYPQRDNCRGFIECSEGVSFKGACGPGLAFNPSQQTCDYTHNVPGCK | chitinase-3 [Hyriopsis cumingii] | AFO53261.1/0.0 | Glyco_18(SM000636);ChtBD2(SM000494); |
| **Unigene40545** | 10.6491 | EVGVHLVNVYR;LANGHLGISFTPR;VVAPSGVEEEAIVQEIDDGQYAVR | 3 | ATYYPKDEGKAKVDVKYAGQNVPGSPFPVEVFPGVDASKVLVSGPGVGKNVYASMPATFTIDTRNAGNAPLDVVVQRPDGSFIKPLVQDNGDGTYTVQYVPDDLGTYVLRVKFAGKEVPNSPFKVTSHPTGDASKCVITEGLENKTVQVNKETVICVDASQAGDGKVTCRIRSPQGSDIDIDIVENADGTFSLLFTPQIEGAYTISIKFGGQTVPGGEYDIQTTGYTAVNEDLISADTVDSVKGAAPGSGLFQPVDFCIPVGPIFNFVSAYIVMPSGKKAYPKIEDNKDGTVTIRYQPTETGLHELHVNYNNEEIEGSPFKFHVDAVNSGHVTAYGPGLSHGIVNEPAYFTIVTKDAGAGGLSLSIEGPSKTEIKCNDNGDGTCTVSYIPTAPGEYNITVKFAGQHISGSPFTSKITSPPGEIKRKSQFGRSSEFELKVVEEDINNLMATIRTPSGVEEPCLLKRLANGHLGISFTPREVGVHLVNVYRNGHHIQNSPFQITVGESELGNASKVKVYGPGLEQGNANELNEFTVDTKDAGYGGLSLSIEGPSKADIECQDNEDGACRVTYKPTEPGNYIVNVKFADEHVPGSPFNVKVAGEPSPKLTERITRHREAADVMHIGSQCELSLKIPASFFMKMMTHEQIEAILVQQQEGTSPFDMTASVTNPSGVTELCDIVSLDDNHYSIKFVPKEMGVHTVSVKHKDMHIPGSPFEFTVGPIAGGGSHKVHAAGPGLERGEINQPCDFNIYTREAGAGGLSIAVEGPSKAELDFDDRKDGSCGVTYKVAEPGEYLVSIKFNDEHIPDSPFRVNVSPSIGDAKKLSVSALQSKGLQIGKPAAFVVNFNGAQKGKLKARVVAPSGVEEEAIVQEIDDGQYAVRFIPRENGGHNVHVFFNDCEIPESPFRIMVGKVDCDPGMVHASGDGLRTGQSGQPAKFFVNTVNAGPGALGVTVEGPSKVKLECTEKEEGYEFTYYPTAPGDYLITIRYAGVHIAGSPFKARIEGQAGPSDVIQHGMSQVVVETVTKTSVMSKFQAIPQFKSDASRVTCEGNGLKKAFRGKQATFNVDTSNAGNNMMFVGMMGPKGPCEELCVHHKGGYQYKINYVVKERGDYMLIVKWGEEQIPGSPFCVHVE | filamin-like protein-1 [Mytilus coruscus] | AKS48135.1/0.0 | IG_FLMN(SM000557) |
| **Unigene40702** | 8.6644 | LPSYPGYNPR;NPFFTINMYYFNAQTGR;SPVECLSR | 3 | WKLFCESNTELRMIRFLIILLVPVTAAFLFSSLNFNNVNQNLDLRKLLTYRQLPHRCRLPSYPGYNPRNPFFTINMYYFNAQTGRCETFGYSGRGGNRNRYRSPVECLSRCACHMPVDPGTCHNSTTGITRYYYNKVFKMCASFQFNGCEGNDNNFADFMSCQLACGRSGGGGEIEL | nacre protease inhibitor-like protein 1 [Mytilus galloprovincialis] | AKQ70858.1/2e-47 | KU(SM000131) |
| **Unigene51161** | 10.6491 | DHDLSHISSEQLR;EALDMQNDQYR;GLVVEAVDEQGR | 3 | MEQISFFCFRCLTLSVLFKLYFCNPARVDLRDHDLSHISSEQLRRVVQQNENQDITTDDDNLLEKLYNQTQYHPTNDESRCISCTIREDQKKHRIESIKNRISHALRIDVLGKPNMTNTKLPKIPQFQKLKERYEIREALDMQNDQYRGEREDYEDEFGQSHRTFTFAQNPPEELGIQQPNAIYFDMPDQTDRSLQKATLWVYVTPSDQQHVTEIYLYTLVKRSKSSDTLIKQFLYRKKRTSRGWQQFNLLNEVEKWTEDPSYNRGLVVEAVDEQGRNVVVMPSSDDNGYQPVLETRTSPHHQHSRNKRSIYLDCSEQRATEACCRYPLTVDFVEFGWDFIIAPLTYSAYYCAGECRNQHMDSGAHSYLQQQVGNVPTEHGPCCSPTRMGHLSMLYFDHSMQIQFTTLPRMKVERCGCA | myostatin [Mytilus chilensis] | AGU13048.1/0.0 | Signal peptide((1-23);TGFb_propeptide(PF00688);TGFB(SM000204) |
| **Unigene57834** | 10.6491 | FAVIITDGSSR;TYLGLEYVHNTIFAPGNGER;VGMMTFGTNPR | 3 | QSSLVVDCFTMGYFALLVLVAVSVVECRNYHSTHVRCEKPVDLAFVIDISSSIWYKHFRREISFIHDIVNLLDVGDRPTQSRVAAVSFSNRLKPEFGLGQYSTKEGVLNAINNIAYEGGDATRTYLGLEYVHNTIFAPGNGERSNVANVVVVLTDGVTNPGSYDNFTRTEAKQKTQIHAQNIRDIVRAQIYAIGIGNEVDKNEIKGIANKPSEQFTLFVDTFTELDTDAVKKAVLTKVCDSLPQREQECSTSKADIFFVVDESSSLMWDANFRKELKFVGSVIDQFELGKDLVRVGMMTFGTNPRMLFYLNDFKTKTEIESLLKITPWHGGNTYLDKAIESLMTYGLNPGFGSRSDVPQIAVIITDGKSTHPTETEKQIAIMKRMNYVVFAIGVGPNKDPIELHKIASHPSNVFEVDNLDGLVAIRQQLLSQLCPGDQPKPPPVNNCQNSMADLIFVADSSTSIGLTAYNEFKTFAKSVVEKFTVGPKNIQIGLITFSNDAHYEFSLNEYRTKEEVTKAIERVPYSTGNTNTHKALEILIKQGFSYINGGRGTSVPRFAVIITDGSSRQPEMTKQLARKAKDQGIILFSIGVGPYITQTELDGMASSPTSMYSFKVDNYAALTRIEQSLVKRTCEEATRQRQFS | Collagen alpha-5(VI) chain [Mizuhopecten yessoensis] | OWF49639.1/1e-152 | Signal peptide(1-27);VWA(SM000327) |
| **Unigene58102** | 11.6905 | EPENPPMENALEPAR;SVFINTDNMR;VRPSTAEFIEQGPLFEHDGTQR | 3 | MKETVLCIVFLQLALVFGAPKSTQTNKVVPKDRVLQGAVEQLFIPDGNSLFEIDIVQGYNPEVIQHVRRKRQAEEKSLERKSSSKKRRRKKKKRRRSEKKPGAVTTLREITPTEKIMLPPVEREPENPPMENALEPARKSRRTFRPPPPTDPAPTLPALVLSEMENNNDDNSGLLKVKSLKSVFINTDNMRTNKLMADRIKADAIHAKDVVVTSKKKKSGSRSGGRKRRRRVRPSTAEFIEQGPLFEHDGTQRVNSILEQPQYIPETILTKNPYGLGDTIYQPEKPRRRFRVNERHLYREPLTVDPYGDALTSGRPEVAHPEFISSRVRSKVPLLSEFIEGTSGNSKPSTTFVNRRRPFAKQRPIEEFHIFDNPINTERQNPVAKRVDKTSKRFTKNVDPWFL | KS-rich protein [Mytilus coruscus] | AKS48160.1/5e-14 | Signal peptide(1-18) |
| **Unigene8686** | 8.7624 | ERSDGEYGSNGAGYR;SDGEYGSNGAGYR;WDSDQDFR | 3 | SGTLYGSCHSKTLDMRKLAVVSILLFLGLPSNIICRNDVKEKDGNRGNRWDDMSDSWGFSEDSSDGGWGSNSVKWDSDSEGWDSDNFGSDRRSPRGRGGNGNNRLDSDSGWYTSEYFSDESDNRVGIRGDNGNKLRNIVGLESEGIDWDSDNSDSSNGAVKKAGNGNSGFGSNGIDRNSETSDSSDGVSKNAGNGNESNGRGRKGNINIGGHSGGRFDSDDSDWGSDRWDSDQDFRRGRSNGNKENNGGGDSNGGGRRWGDSDRRDSDSWDSDDNNIWDGSDGWERERSDGEYGSNGAGYRGSDDSDWFESDNRFDSAESFERLIKRLRRPNDGDSDGNSPPGVKFLGNNGVKRADFWDDSDDLWNDYAIGRRRHRPRRRRISAKRLGLDDFGDDNGDDN | — | — | Internal repeat 1 |
| **CL2084.Contig1** | 5.9434 | ELSELQDQLTGSTSSR;SWSSMSFK | 2 | MDTKCVFLLLCAVTMVIGSSIRKKRSPANPLTKSEVSYYLKELSELQDQLTGSTSSRKRDRSSKRRKNKHEEQWRAGKSWWEQKHGKNKKSGRSRSWSSMSFKCDQPLSYGNTDCTEAVVRYYYERESKMCREFHYTGCGGNRNNFRSLSECKQKCKR | protease inhibitor-like protein-C [Mytilus coruscus] | AKS48156.1/4e-40 | Signal peptide(1-18);KU(SM000131) |
| **CL2840.Contig3** | 6.7523 | ALVDTETFASPR;ASSYLDDIYYPEPIVR;ASTVSLVR;EATRALVDTETFASPR;GFYDTTR;GSSQAALVGNR;GSSQAALVGNRVEVVTPR;TPTPVTTSR;VEVVTPR | 2 | MTVRRSRFQSVPPGYFSSTKGHSSLKRWYPTTTRASSYLDDIYYPEPIVRSRGFYDTTREENEIRRDVNHELLYTSNLVDDTYDIANKSRNRDQMLLREATRALVDTETFASPRSAVTSRRVRQTSVVRTPTPVTTSRAVSCPPVSRGSSQAALVGNRVEVVTPRKRKPRSTYAANKMRELKRDEREMEVEAVPVQSTTSLKASTVSLVRY | RS-rich protein-2 [Mytilus coruscus] | AKS48164.1/4e-65 | — |
| **CL2840.Contig7** | 7.7937 | ALVDTETFASPR;ASSYLDDIYYPEPIVR;ASTVSLVR;EATRALVDTETFASPR;GFYDTTR;SLPPPVISLER;TPTPVTTSR;VAVLASPLR;VEVVTPR | 2 | MTVRRSRFQSVPPGYFSSTKGHSSLKRWYPTTTRASSYLDDIYYPEPIVRSRGFYDTTREENEIRRDVNHELLYTSNLVDDTYDIANKSRNRDQMLLREATRALVDTETFASPRSAVTSRRVRQTSVVRTPTPVTTSRAVSCPPVSRGSSQVVVVLTSPAFKGGSRVFSAKSAAMAVRERSLPPPVISLERSKSVHPQLLPSDILEKRINARRIINTVAGPYMLPLYHPYQSIYQPYISMYQPLYARRKYLATLKDVPSRGRVAVLASPLRRKKYRKAALVGNRVEVVTPRKRKPRSTYAANKMRELKRDEREMEVEAVPVQSTTSLKASTVSLVRY | RS-rich protein-2 [Mytilus coruscus] | AKS48164.1/4e-146 | — |
| **CL93.Contig1** | 6.1312 | FTQAGSEVSALLGR;ILDPFVVGEEHYDVAR | 2 | VIAQSTVAMMHAARRACVGLLKATKHSLTSPAVPSTATKALPSYFNTRHYAAEQTQPNTAKGRVVSVIGAVVDVQFDEELPPILNALSVENRTPKLILEVAQHLGENTVRTIAMDGTEGLVRGTSCIDTGYPIRIPVGPATLGRIINVVGDPIDERGPVKTDKFLSIHAEAPDFVEMSVTQEVLETGIKVVDLLAPYAKGGKIGLFGGAGVGKTVLIMELINNVAKAHGGYSVFAGVGERTREGNDLYHEMITSKVISLTDDTSKVSLVYGQMNEPPGARARVALTGLTVAEYFRDQEGQDVLLFIDNIFRFTQAGSEVSALLGRIPSAVGYQPTLATDMGTMQERITTTKKGSITSVQAIYVPADDLTDPAPATTFAHLDATTVLSRGISELGIYPAVDPLDSNSRILDPFVVGEEHYDVARNVQKILQNYKSLQDIIAILGMDELSEEDKLTVARARKIQRFLSQPFQVAEVFTGSEGKYVPLKESIAGFQRILSGELDHLPEVAFYMVGPIEEAVAKAERLAEDQS | ATP synthase subunit beta, mitochondrial [Mizuhopecten yessoensis] | XP_021356377.1/0.0 | ATP-synt_ab_N(PF02874);AAA(SM000382) |
| **Unigene1696** | 6.2551 | IGEAEAQR;SQQIQLQEQEITR | 2 | EINRNSKLGKGLGRNNQIQFAVMGFETCGPNEVMVVSGCCHARSIFVPGGRVFVWPVIQKLQRLSLNTMTLHIESPNVYTQFGVAISVTGVAQVKIQGSNQDMLASACELFLGKNDTEIRKVAQETLEGHQRAIMGNMTVEEIYKDRKKFSKAVFEVASSDLVNMGIFVVSYTITDIRDSEGYLKSLGLARTAQVKRDARIGEAEAQRDAGIKEALAEEKRMTARYSNDVDIAQSQRDFELKKAQYDQEVFTKKATSELAYDLQAAKTKQAIKEEQMQIKVIERSQQIQLQEQEITRRERELDAQIRKPAEAEKFRLEKIAEANRKRIILEAEAEAESIKLRGDAEAYSIEAKAKAEAEQMAKKADAWKDYQDAAMIDMILETLPKIAAEISAPLSSTKKITMVSSGKGSVGAAKVTGEVLDIMEKIPLVVENLTGINISKNIKAATRRP | flotillin-1-like isoform X1 [Mizuhopecten yessoensis] | XP_021361554.1/0.0 | PHB(SM000244);Flot(PF15975) |
| **Unigene25503** | 6.7523 | GSPFMMIDNR;TNPWNENSLYR | 2 | GESDNNEENEESSTQEKQKMQYYGNNDENRLLDDSGIESDDSAQSDGKNDKFDYSDRESFEKEYNSLSSEEDHSEMANNDLNKDYDSDEDSYQHNEIKLNVKTSSSSLNWGYNQPTPTPSSVRTNPWNENSLYRSNVDYDDKDVNVEGDSVNAFKKIVPGFTEKTPSYNYMEIQNNKLQVNTFGSDTNDYSESENEKENEGDDVGDGDENKKWNKFSEEKEDKQQNEHWSAGAVPDIYAGTVIVTSRKRGSRIDNTGKEKENENEIENEDKISSKNSKLLGVKKGKKVSSWGGQEGNSAEKAENEVVEKSPDAENEKPEKGSLDKQKEDENSNNLIGLTTIYQEEGDGKSANEENELKQSVKENSKKSLKEENEMKTLKEENEIKSHKEENEMKSPEEEIKIKTPKEENSFGEMKVDLAGHIGDSNIVKPEEDIGESKSNSVFMNNQQFRNVDDPSEPVKSASNSMGVKPFIGDSPKFMGNMKQAQMFYPTAFPAMNSFTTRGSPFMMIDNRYSTQYQQTPNQQHTTMSGFRKRVQNRISKSMGHNIDDPVMTQPLTLKVKIKSTRKSKKGLSPNLSKLVLKMFMNSKSGAPPCDGNLKSVCRPVKMWSKYQQVADWCTSLCPYGQCPSAVCKCQCTGSPMGSVGQKKCRATNTYKQNSGELDQWCQKTCSKGDCPALLCVCS | Pinctada martensii chitin binding protein mRNA, complete cds | KJ930034.2/0.039 | Internal repeat 1 |
| **Unigene2736** | 6.7523 | NGPFTVLR;TQGSYTEIQECFAR | 2 | RKVMEQKLFLIGVVLMCSLLSVNSLCNFPCSVQTGGDYDGELGSFRWSCDNSSRLIRTQGSYTEIQECFARNGPFTVLRRNGNQYQCVKEAAVTGKVTWVYETEFLTMWNPPTVCSICTPVLMRPMMYVDPSVICIPGTKKKGQYTLKSLKKMKPPPIGCNRPKNCPLSSTLDVPCTGCEPFDDGSCCPGCKRKLQQSYAYAYAYADSMFGPQFMGHYNTML | hypothetical protein [Halomicrobium katesii] | WP_080505770.1/0.14 | Signal peptide(1-24) |
| **Unigene34438** | 7.7937 | QSTAGSITPEEQNYLTQAHNLLR;YSTYEDWLR | 2 | IYKTYFTKALRPREVMMDALKAWETEKQNLFSWDAKCTDSETCNYKQLVSAKVDSVGCALIKCPRMIESKKTTDYDMYYFLCFYSPRVPLDQQPFTYGRQCSQCPKGYSCKRKLCTRPKWTKILHRDNKPNSIRTQPLPKPTKNIVIREDSRGYRNFRNVSNDQSNVIRVISPAELTGSKPSEKVFNRLTSLSRTKRIKRQARRRYSTYEDWLRRERARRERQRNEEIYRREVERRRREQERQDAYRRRNSRLSPIRQRPNRQSTAGSITPEEQNYLTQAHNLLRGNTMQDLKWSRYIQRWADYVIRCNIEYPGPITMYTNFGNVQNGGNIYNIVYDWGNEGYNTNRILKNGCRTPNDRERCNHNTIVRNDFIREFACAARDCSNNRRQLTCIYR | Reticulocyte-binding protein 2-like protein a [Crassostrea gigas] | EKC19657.1/4e-39 | CAP(PF00188) |
| **Unigene37654** | 4.4081 | NIGHFHPAGSVR;TELLESIIDGSSVR | 2 | LWKRAFVYNVDPQIKSSKTELLESIIDGSSVRIVIKENENNIMSVRADNLGLNTNKSEVAAQSIRNIGHFHPAGSVREFHTVPYWEFTTVVTTGDLIKTRWSTGTYEF | uncharacterized protein LOC106163392 isoform X1 [Lingula anatina] | XP_013396411.1/6e-07 | — |
| **Unigene37757** | 6.7523 | EGGIPSYTTEYR;YGTDITVPNR | 2 | MEYERKVYRKETTREGGIPSYTTEYRIGTDRPRYGTDITVPNRYTTTTYKTTGPIVYSSYSSRPATEYTTYTEPSVEDTRVRKEWDETFKRVAPRADDWSLSDVISKRMVLVKDDEWDPYETSFPEERKRFGKPSPITSDVSGRKTFLVEYQIGDFRPEEVEIKTIGNTLKIHAKNSDSGSMKREYSREISIPQEVNPDLISAKLNRTGRLSIEAPIFNTSHKTKIDRRIPVLRN | heat shock protein beta-1-like isoform X1 [Folsomia candida] | XP_021944507.1/1e-12 | HSP20(PF00011) |
| **Unigene42298** | 4.9918 | EETLAYEESTR;STVEVNGGTGR | 2 | YSGTYSRENMQVQPYGYSDSSSMKRTTAYEYEDGGSPYGSSSMKTVKQSYEMSSAPQINPYFVQQPLILPPFPVPQYNLLQLEPVPQKVTTGVQVVTEQREQKEEPRIVTSTDETDSKHFKERPVYKSVALVKGPYESPKWHTAKKHKPYEPPDEVDSGRMYKVVKSSASDDVREETLAYEESTRTTTTMKAENAGFSGGQFFDENRNVSSIEMGNSRPLNLTGHGVVNLKHTQEKRPFRSTVEVNGGTGRKPYDQLDGYQSKLSTYLNHQQNYQGTP | hypothetical protein AM593_03515 [Mytilus galloprovincialis] | OPL33566.1/3e-97 | — |
| **Unigene44900** | 5.4866 | AVGQDLGTSGSCLR;GFSGLEGIR | 2 | GVPGLNGRPGQVGPQGDTGLPGFPGLAGSRGEDGLDGLPGINGEPGQPGDSGYPGPAGPPGDDSPYIQGARGDPGIDGIDGRPGIKGHRGDDGLPGLDGLPGMKGNPGFEGFGLKGEPGDNGITGLPGSAGPKGYPGRPGIFGFNGAKGEPGLPGVNGEPGPDGQPGLNGLPGDAGLDGFPGSKGESGDYGYNGQPGLPGTPGENGFRGSKGEPGLNGLPGMEGDVGEQGKTMNPNGYTSGPKGVPGDFGEKGLNGIDGNRGFSGLEGIRGEPGLPGLPGEIGIPGPKGVRGDAGRDGYPGLPGLDGLPGLAGLPGENGIAGRSGIPSGVYFARHSQTTLVPECPTGTSLMWEGYSLSFIMGNGRAVGQDLGTSGSCLRRFSTMPFMFCNINNVCNVANRNDYSYWLSTLEPMTPMMNPISGPPLQDYISRCSVCEATGEVIAVHSQTVRLPDCPVGFKSLWIGYSFLMNTGAGGRGSGQNMQSPGSCLEDFRAAPFIECHSRGTCNYYATSLSFWMATIESFNQFRRPIGETLKAGNLRTRVGRCQVCMRY | Collagen alpha-1(IV) chain [Crassostrea gigas] | EKC43052.1/4e-172 | Collagen(PF01391);C4(SM000111) |
| **Unigene46263** | 7.7937 | STGSGVSLTESR;TTITTVDSGSSR | 2 | NCPASRSERICNTQPDQCRECEVKGRRYRGNSRFSYDEDCYRYNCDCNCNGSWNCPASRTQNICGSGQDQQIGESRSTVTTSNSGSITRTTTTGSGCRECEVKGRRYSGNSRFSYDDGCYRFNCDCNCDGSWNCPASRTQDICRSGQGQNTGGSQTIVTTLESGGSTRTTSTGTGCRHCEVKGRRYRGNSRFSYDEGCSRFNCDCRCDGSWNCPASRTQNICESGQRRQTGGSRTTITTVDSGSSRTTGTGCNECEVKGRRYRGNSRFSYDEGCYRFNCDCNCDGSYNCPSSRTQNLCGSGQGVRTGTRTTLTTSTKTTGSGESAIGNGVDSRRTGSSQISGSRRSTGSGVSLTESRRTGGGLSRAEKTFTETRTSTSSRSTSSKTVVGGSQTKTELRSRSGSTTGSAVDSYGCHYCIVNKIQHRGNSNFQFEDNCFRFSCECRCDGSWLCPADRTINICPDRCQNCNVNGKSYPSNTIFEYDEGCNRFNCDCACDGSFNCPAERTKDICSGRQDKCKECNVNGRRYQPNTQFSYDERCYRYICDCNCDGSYRCPAERTKNICGSLRADTCQECRVNGKNYEPKKTFSYIDGCALRKCTCDCSGRHRCTATTDICKSSSQVCRDCVVKEKRYLPNEPFKYTEGCDRFNCDCNCDGSWTCPAERTENLCAIGQCQKCSVRGEVKEGNSGFVVTENCRQYKCWCHCDGNWSCSKDFTRSCNLG | uncharacterized protein LOC106876685 isoform X6 [Octopus bimaculoides] | XP_014780810.1/2e-109 | Internal repeat 2 |
| **Unigene52025** | 6.7523 | FAESTSALFR;TINSFMEPFQR | 2 | DLQSYLDDLPMGTGLTGRNMHNNNFNGQDLQSYLDDLPPGQRMNGGGLRQFPDHVYENKFKFNRMGGHQSQFGTQNHKVVGDTMKATTRTINSFMEPFQRIRSMNNMNSNQFLGLSKATDLGSNINRITNHRESPEPTSFHHQIRPHATQMSKQRFAESTSALFREPKSANSRFPKHSTLNNVISQTVITEPSLSPMGLGAFLTKQNPIQKLLSPSKPSTTSTLQDNSATKTSVDNQSKLPIQKSLSAVSNTFRMGNNNQVKEFSPKTAKFQNKHI | cyclin-dependent kinase 1 isoform X2 [Trichechus manatus latirostris] | XP_023595093.1/1.1 | — |
| **Unigene52317** | 7.7937 | DMENMAFETVDR;LTPIDYEFHVWR | 2 | VYTDVDRNMKVVRGNILIKNNFEDGLDEFNDNINVFDNGFNMSKSNPLYNSDEDIYKRYEEEKRRQQERDIRDMENMAFETVDRFSKSKNVTRKADGERRKPRSKQNLTNLLLTRLSTMDSKDIRQTLDVKHHHEDVYGDPHLEQADGTSSRRITNSNYDTVTENVDLEMKDGKAYITITVTAERLTPIDYEFHVWRKNQAIVTRVVEIDFYADEQRRMLYDRVMDRVDDEGYSTTNKYSHYTRRKPQEVVLSSQDTLELFNELMDAAGGEGDREDITVKTKRNKTLLPENPFLY | hypothetical protein AM593_03515 [Mytilus galloprovincialis] | OPL33566.1/5e-170 |  |
| **Unigene5251** | 6.5280 | AVFVDLEPTVVDEVR;NLDIERPTYTNLNR | 2 | MRECISVHVGQAGVQMGNACWELYCLEHGIQPDGQMPSDKTIGGGDDSFNTFFSETGAGKHVPRAVFVDLEPTVVDEVRTGTYRQLFHPEQLITGKEDAANNYARGHYTIGKEIVDLVLDRIRKLADQCTGLQGFLIFHSFGGGTGSGFTSLLMERLSVDYGKKSKLEFAIYPAPQVSTAVVEPYNSILTTHTTLEHSDCAFMVDNEAIYDICRRNLDIERPTYTNLNRLIAQIVSSITASLRFDGALNVDLTEFQTNLVPYPRIHFPLATYAPVISAEKAYHEQLSVAEITNATFEPANQLVKCDPRHGKYMACCMLYRGDVVPKDVNAAIATIKTKRTIQFVDWCPTGFKVGINYQPPTVVPGGDLAKVQRAVCMLSNTTAIAEAWARLDHKFDLMYAKRAFVHWYVGEGMEEGEFSEAREDLAALEKDYEEVGVDSVEGEGEEEGDEY | tubulin alpha-1A chain [Mizuhopecten yessoensis] | XP_021370666.1/0.0 | Tubulin(SM000864);Tubulin_C(SM000865) |
| **Unigene53044** | 6.7523 | GYQDVIFGGFDQR;LAVINANR | 2 | DKSSVGRNISTKVPDGMPVKPYLTDVASIRQDITDDYKYKDESDRERLAVINANRSALKTRPVYNRGYQDVIFGGFDQRDFIMIGKEFKVTVTFRNVGDHVRTIKGRLICESVSYIGIKQKTIKEHSFEIPLDPRSEDVASMKVGLTEYLPHISEQMGMKVSAILQVVETGHIQAFQDDFRLRKPDIQIEILDNHIRVGESFKCRLSFTNPLSAALSKCCLTIEGPGLDNEEQFDLSNISGHQEWAAHLQLTPRKPGRRQITASLDTHQIQNIVGVAEVKVLP | Protein-glutamine gamma-glutamyltransferase K [Mizuhopecten yessoensis] | OWF48045.1/3e-98 | Transglut_C(PF00927) |
| **Unigene6711** | 6.7523 | FEFSYFGTR;NNGMTYNIINYK | 2 | TDGFLAHNGRNNGMTYNIINYKLLTNKRLCIRFEFSYFGTRTPHHSCHQALLTTTATSKITKPSTVLTTTLRPRTTSSASDTSSHSTTKTSTTADTTTEKLITPTKSTTQRLTTEHQTWPTVSATTATTSVRSYTATSAIFTTTMQTTQNTQRTTAQQRRTTTTLPPTTTTLPTRPPMPDPSR | — | — | — |
| **Unigene7251** | 6.7523 | GPQGPAGEPGPAGPPGPFGER;QGPSGDTGPR | 2 | MMLSRVRWGLFGLLVVIWNVNAQKACKYQGNDIGVEEEFVPDENVPCRTCKFDQDCRLHCDFKSCDPLECLEGQTEVLHPGQCCPVCEELILVDKSLKNPKVDGKLVNDEKAGVWGGAVGPGSANGYVVGAPGPRGFTGPPGPPGPVGYQGPRGEPGEPGQPGPSGERGFPGPSGPPGSPGEEGLPGEQGPTGPIGSLGNAGQPGMPGMPGPKGHRGFPGQTGKTGDEGRPGEKGPAGPSGAPGSPGPMGPRGPPGERGRDGSPGPQGIRGQDGKRGDSGPPGPIGSSGSPGFPGSSGPKGDSGQPGQRGEQGLQGPPGVSGLPGPPGESGNPGRPGQDGDPGIKGDLGQSGAPGAAGFPGPQGPPGQPGEPGTPGPAGESGLAGQDGRQGDPGERGYPGAPGEPGLTGLAGAEGKRGAPGIPGPPGPSGISGERGPPGPAGAPGQVGPAGAKGRDGERGADGERGVAGEPGTPGIPGPPGPVGQRGPIGNTGNEGKPGVQGPAGNSGADGRPGEQGQQGPPGAPGLAGPQGQPGESGPSGRDGESGPPGAQGPRGERGPAGEPGPIGGPGLPGPQGERGSPGPQGETGVQGLAGAAGAPGEPGRTGESGSAGPQGEPGVQGERGEQGFPGDLGPEGKRGPSGERGPQGPAGEPGPAGPPGPFGERGAPGPQGLVGLQGDRGPNGPPGTRGNRGPAGERGKDGAVGIPGEVGPAGLPGPAGPVSLIMPEKGDAGPPGTPGENGLKGDTGPQGYPGNPGPMGPQGAAGPPGVPGEVGSEGRQGPSGDTGPRGYPGEPGPGGETGKNGVDGAQGPPGEIGQQGPAGAPGSPGHPGPPGPQGPQGNTGFVGAPGKAGQRGDRGETGPPGPAGKDGEQGPPGLNGQVGERGNPGQQGPQGPPGPSGPQGERGSPGYPGGQGESGPAGEPGVQGSVGQPGDDGADGIPGPPGPPGPSGQPGFTGPNGEPGAPGLNGLPGAIGQSGDKGSRGPSGPPGLQGPPGPPGQSGPTGNAGPQGERGERGVAGEAGIPGAPGQQGATGATGPAGSPGETGRTGAKGDKGWPGMPGGQGLPGPQGPGGEKGPSGPPGPPGQPGSNGARGNPGRDGEPGPPGMPGRAGSRGPQGDDGLTGPSGPPGPPGPPGSPGYAPVWPGGNWQQQQNKGPDPLYYGDEPDKTPVINDDLSRIQEALHRTKRPSGKKHNPGVTCKDLLLQNPDFEDGWYYIDPNGGSFYDAVEVYCRMNIDGETCIPAGRKLYAEDQWTKVTKSQWFAKEILGGAEFDYKIDDIQLKMLQMHSTKARQRVTYKCMNSDPTGAVLMSNEFESLETLANKQEPYSAVKVEAFGNCDRTSNANRWGEMIFDVKSERSESLPLLDIRLKDVGQSNQEFALSLGEVCFNT | collagen pro alpha-chain [Haliotis discus] | BAA75668.1/0.0 | Signal peptide(1-22);Collagen(PF01391);Internal repeat 3;Internal repeat 5;Internal repeat 6;COLFI(SM000038) |
| **CL105.Contig2** | 3.8968 | SQIAIPAQGMIEFR | 1 | RQNRKGCTMSDRESGDEQQEHQGQGSGEQELATKTLQIQSKRFYLDVKQNRRGRFIKIAEVGAGGKKSRLLLAMSTAAEFRDYLTDFSEHYASLDNLPEDGKLKSETMIKDNRRYYLDLKENQRGRFLRVAQTRPRGGPRSQIAIPAQGMIEFRDALTDLLDEFGTDDHGESDEGQGELPESKYLRVENKVFYFDVGSNRRGVYLRISEVRSNYRTAVTIPERSWGRFRDMLSEFVEGSSQSGTAAEAPKETK | transcriptional activator protein Pur-beta-like isoform X2 [Mizuhopecten yessoensis] | XP_021353033.1/4e-129 | PUR(SM000712) |
| **CL1444.Contig2** | 2.3583 | ETFELTR | 1 | SEKKGKRGITFAENVVGGYTSSSTNSDSDEDASDSGSTASYDEGSYDGREGSIVYQCKDDEAIAKGIPGAKMFDQNIRETFELTREMRQSCETLAKYLEDSTEVQTKELNASLDRIKEEWFKISSAKLSEPYQVEDFMSSFNEISRRLLEYIVNIQDSNGNTALHYAVSHCNFEIVSLLLDTGVIDLSKQNKAGYTSTMLATLAYTQTDRQREVVQRLFSMADTNAKAAKAPSDVTPILLSDSGSGSFRQQSQSLAPGFRSSSGSIYIPPRSAPKRSTYHKFFRI | KN motif and ankyrin repeat domain-containing protein 1 [Crassostrea gigas] | EKC18202.1/ 5e-108 | ANK(SM000248); |
| **CL1815.Contig1** | 2.3583 | DIVQFVPFR | 1 | LTSFKLQRTPSFLEYLYGGMQINFTVGIDFTASNGDPNSSNSLHYINPYQPNEYQQAIQAVGNVCQDYDTDKMFPALGFGARIPPNNEVSMEFALNFNATNPYCAGVQGILEAYTNCIRQIRLYGPTNVAPIIYHVARFADAAQKEEATKGAHSYFTLLLLTDGVITDMNDTRQAIVNASGLPMSLIIIGVGDADFADMEFLDGDGGVLKAPNGQPAQRDIVQFVPFRDFKRVSAAQLAKHVLAEVPQQVVKYYTMRQIMPNPPRQAQQ | copine-3-like [Crassostrea virginica] | XP_022342060.1/9e-143 | VWA(SM000327) |
| **CL1881.Contig1** | 2.1737 | LLLPGELAK | 1 | MPPKATGSKGAKKAVTKAKAARAGGDKKRKRRRRESYGIYIYKVLRQVHPDTGVSSKAMSIMNSFVNDIFERIAAEASRLAHYNKRSTITSREIQTAVRLLLPGELAKHAVSEGTKAVTKYTSSK | histone H2B [Mytilus chilensis] | AAP94663.1/6e-54 | H2B(SM000427) |
| **CL1924.Contig1** | 3.8968 | AIIDVLAFR | 1 | KILLTYEWTFRNFNLAFTFSDEKKKMSYPYGGGNPGYGPPGGGYPQQGGYPQGPPQPGYPQQQPGYPGQVAMPTVESASAAAYAPGPGGYPGGPGQGLGFDGVGGPPQGSGYGPPQPAPYGAPPAPAPAMPSSYGPPQPQYQSSTPAYGSAPGYNPAPQGAPPPAQPGYGAPPFQPGHNSMPNQSYGASAPPPAGGSYGAPPQPQGGYGAPTSQPGGYGQPQQQQQGYGQPTSQNYGQPPSSQQYGSQQKSQPAANAYVQRTEGTLRPASNFNSENDANILRKAMKGFGTDEKAIIDVLAFRSGEQRQQIRTMFKTMFGKDLIKELKSELGGKFEDVVIALMMPWDEYDAYELKRAMKGVGTDEDAMIEILCSRSNKQIQEINATYKRLYHKKLEDDIISDTSGHFKRLMVSMANGGRMESQDVDSNKAQQDAQRLLQAGEKRLGTDESTFNAILASQSYIQLRAVFDAYQKIAGRDIEQSIKSEMSGNLEIGMLAIARIVKNRPAYFAQKLYHSMKGLGTDDKTLIRVIVTRAEVDMVQIKQEFQKLYGKSLDQFIREDTSGDYRKVLVALVSQGGY | annexin A7-like isoform X1 [Crassostrea virginica] | XP_022317069.1/0.0 | ANX(SM000335) |
| **CL249.Contig2** | 2.1364 | EPFPNVNR | 1 | RNAHATSFLVGAMAAGTLYTYPDNFRAFKAQIAAQFSGTDIKLASGFKFGETNQSKDFLSKFPLGKVPAFVSSKGDCIFESNAIAYYLGNPQLRGTSDKDASLVLQWINFGDNEILPSACTWVYPCLGIVQYNKQETEKAREQIKKALSVLNNYLLTRTYLVGERITQADISLACNMLSLYKYVLDPKFREPFPNVNRWFTTVVNQPQFKKVVGDFKFCEKMAEFDSKKYQELHGKGDGGKKDKKKEEKKQDKPKQQPQAKKEKPKKEEEEEDDGFPKEKEQKDPFGQLPRGNFNMDEFKREYSNKDTIKEALPYFWKNFEKDNYSIWFCNYKYNDELTRIFMTCNLVGGFYQRIDKMRKNAFGSMCVFGEDNNNSISGIWFWKGQDLAFKLSPDWQIDYESYDWKKLDPTSAETKKMVQEYFAWEGDFGGKKFNQGKIFK | elongation factor 1-gamma-like [Crassostrea virginica] | XP_022332141.1/0.0 | GST_N( PF02798);GST_C_2(PF13410);EF1G(SM001183) |
| **CL2603.Contig1** | 2.8554 | EFEFQSGDLWEVR | 1 | MSDKTRANKSGLGYAVEKKMEDNYDREEAAGTPTHVVNWVNGILGSEHDPIPGTDWKSICNHLRDGVALCKMVNILLKKDGKSPITFQKKVMSPFVAMTNIENFNKGCLDYGLDREFEFQSGDLWEVRKGPFLNVINCIHSLGFVANKKNVVPGYTGEIRKYLDNE | transgelin-like protein-3 [Mytilus coruscus] | AKS48154.1/8e-109 | CH(SM000033) |
| **CL2714.Contig1** | 3.8968 | QQYDMIELAER | 1 | MSDTEETTQPENEAKLAMEEAAARKAEKIAMEIAEFEEQRREEKAKEEEELAMLREKREQRKIERAEEEKRLAQLRIEEEARRKQEEKERQQKKAEDEQRRKEERERKRKEQEERLKLVKKPNFVITKRADGGDDERRKKAEQKAEEMQKSKEQLEQEKRAILAQRIQELNIDGLKSDGLIQKAKDLHEKLHNLMGEQYDLEQKFKRQQYDMIELAERARQMNKGKNRSTMGVKVDESFDRLADKFINAPPKIQLCSKYERHTDNRSYNDRMNLFEEFSKPKPPPEIIRKGAQTSGAEDGEEEEEE | troponin T [Mizuhopecten yessoensis] | BAA22851.1/ 1e-42 | Troponin(PF00992) |
| **CL2758.Contig1** | 2.8554 | SAVVAAER | 1 | MATNILCFGFLISTTILKLVNGAAPISAKGGGSNSFVFSAKNKFSFSNPSPPTSSQGQGFSSSSSSSSSSSNAGSGGGFSPNNNQNQWKQTLPINYEPNKQGNNQQPNQQNGNQLSSQQQQQQQQQQQQSGQQHQQQTQMQHQNMQMHPNNQFGQPQTKPMNQNQKFNSPGQMGSGQQWQPMGFGQQQQQQPQNPQHQQQNTFSNPQQPQNGFSNPQQNSFSNPQQHQLSNMNTGQTMFQQPPNQQQKQQFGQPNQIQNSHMTPFGQQQQQQQQQQQRNQFNKFGQPQSGSNMNVLSQGGQPNNGMPPYQGMPLTANNQQGFQGQRPQQQQQSGHMTQYFPPHQSQQTNQMHGPGQNQGHQGQMPMNGNMPMQGSPQNMQMGSHQQPMNGQLPAHMQGGPQSMPHPSGSSAPAHQQMGRPHPPIGGAINPPMGGAINPPHGGPQMGGPGHSPHGGPYNPNQGGPGYPPQGHGPRPGHPNHNVAVIDHPRKTTTTPKPQTSTIDGEGMVCMTTADCEIGCCFNATGQLLDTTTYGAGGPKEGRASGKCFIRKPGLGDVCDDLCACTMGHDCYRRYVPVYPKPGQKTAPVIDPEAAPKPQRTCVRSAVVAAERIAFWSCYFDVSCSGPLP | — | — | Signal peptide(1-22) |
| **CL3460.Contig1** | 2.2344 | TLPTLIR | 1 | MLQTAMEVDTGWDSISILMADEAGKWMDSIINLKNSVIGNNKYKSLAVQQGIIPRLLQWIIDDGVPIELRTEAAIVLGSLAKGTEQDLVMLVSAGSVSVLLIGITNNNVKFVEACLRCLKTIYLMNDPPIQLIFENKTVIPHMINIISKSICTQECITTIFSRCCTMKEHQEKLCMNGALATLAPLLTSSIYKVQMPTLKSIAVLCYQNEDVAKATATATYNGESIPSLLLKLLARDKTSEMQMAAARCLTYLCRGGALQPSSNVIMYKTLPTLIRMCKKDRTLEENVEGAETLAYLIEEDPDLQAMASISDHIIKTLAEYLRYTDVQQINSRVTQKKEINWSNELRQAAFRAFASLGANDEDIRKKIIETENLMDHIISGMNSDDVKVKGAAVRCLHSLSRSVQQLRTTFQDHVIWKPLMNMIQNGPEELLMITSSTLCNLLLEFSPGREVSNPRKAILDAGAISILVTLTARQEPELRLNGIWGLMNMAFQSELKVKSQIIEAVGTEQLFKLLSDPDPNILIRTLGLIRNLLSGKNHIDSIMSVYGTQIMQAVVFILEGDHASDIKEQTLCILTNVADGDDAKGYIIENEDVLKKLMTYMMHTNVKLQISATTCISNLVWNEDEGAYKRQGKLREMGVQKILQQLLSSNDAILFDRVKDALKQFG | armadillo repeat-containing protein 8-like [Mizuhopecten yessoensis] | XP_021358110.1/0.0 | ARM(SM000185) |
| **CL3748.Contig1** | 3.8968 | LAAQYAEAQEEIQR | 1 | MPKAVNVRVTTMDAELEFAIQPSTTGKQLFDQVVKTIGLREIWFFGLQYVDSKGYTTWLKLNKKVLSQDVKRETPLQFKFRAKFFPEDVTEELIQDITQRMFFLQVKDCILSDEIYCPPETSVLLASYACQAKFGDYNKETHPNGFLANERLLPQRVMEQHKMTREQWEERITNWWSEHHGSLREDAMMEYLKIAQDLEMYGVNYFDIKNKKGTELLLGVDALGLNVYEKEDKLSPKIGFPWSEIRNISFNDKKFVIKPIDKKAPDFVFYAPRLRINKRILALCMGNHELYMRRRKPDTIEVQQMKAQAKEDKMSKQQEKQRLESERIAREEAEKKQKEMEEKLRRFEEESERRAKEMAEQERRLRDMTEEMEAYKRQQEELEEQRRAAEELRRQYEESAHLAQEEKDRLAAQYAEAQEEIQRSMAVLEEKESEMNSMQQNLEQAQKEREEKEQALIEAMNTIHVRETEHEENTVEEVNHEYSQVETSEDVHMTFENEYEENTVEEMNHEYSADLQDYEQVESLPRPEEERLTEAEKNQRMKEQLKSLQEELQNTKIDEKATTTDMLHAENVKQGRDKYKTLKQIRQGNTKKRIDEFESM | Radixin [Crassostrea gigas] | EKC27987.1/0.0 | B41(SM000295);FERM_C(SM001196);ERM(PF00769) |
| **CL3763.Contig5** | 3.8968 | LEQIYEEER | 1 | GYKSEPDTSSRIKNRTKSLSDFKADFKSSSTKPRTLDTSEISAEVQDFLNYLDEWNPPNARSTVEKYRCQPRSIVDYEPGFSSIAFQEAKVGTAHNPPIEKPGQFSRYTEGGKALPSDPKEDAADPADLYKQVQKGGDIPIRGLQKPGPEKPAIDYYEDAFKFQTSDNVRKSPTERVQRRREDEEEYRKKRLEQIYEEERRRKKEQEIADIEARKHSDFFTNEIIMAEPAQKSPIPADRFDEPISGYNTVPADRRRGFKIHGKAKALYSFNAQNPRELSFRKGDVIFLLRQIDKNWFEGERHGRSGIFPVNYVEVITSIEEAHSAAREAEGQARTKYNFNAQTGVELSLRKGDVVTLLRSVDENWFEGRVGNKQGIFPKSYVEVIFDPSTPLVTPAPSVITTPMTGRGTPEMLSPSGFDAPTPPPQPSPGAFSPRTASFLHQQQRQFAPPQQQQQQQNYNSHPQSAFSPQRANMNY | Vinexin [Mizuhopecten yessoensis] | OWF46493.1/1e-138 | SH3(SM000326) |
| **CL3802.Contig3** | 2.2344 | IANFGALR | 1 | EDKYEMCLILEFLAGGELFDRIAAEDYKMTECEVINYMRQVCDGLKHMHENSIVHLDVKPENVMCTTKNSNEVKMIDFGLATKLNPDEVVKVTTATAEFAAPEIVDREPVGFYTDMWAVGVLAYVLLSGLSPFAGEDDLETLANVQRCDWEFADDAFSNISPEAKDFIRQLLIRQPQRRMTVHECLDHPWLKGDLSSRTTRIPSSRYDNIRKKLKAKYADWPAPNPAIGRIANFGALRKNRPKEFSIFDAWFDRKEAAPRFIRKPRTVITAEGQTAKFDCKIIGASPPIVTWSFDNSVLSQSVKYMQKYRGNEYELKISRIKMSDKGVYTVIAENSFGKKEEHGTLKVEANPDLPKIPSSRDTTPLRRSRRPSMSPAPEVKPIEEAPRISFGLRPRLIQAGTEFKLLTCVQSTPTPKVTWSKDGKDISKDPHYMCSYSGGVATIEVQGARMADTGVYTIHAVNELGEHETSSKVVVEDRAHDFDKADIFKSHKQSRSARRTKSGFNFEDSSSSYTETSTSSSSTKTSRSSRRFESSTEESYSSSSSRRSNRRKEIEEPSYEAPEFTTQLSPLILDEGDRLKLTCTVKGRPDPEVEWFYNGQLMQSDDAIKITAIGGVHTLVINSCILDDDGSYVCKAKNPGGQASTRTTVQVNEKKSLSSSKPDFIEHPAGVSLEDGDQATIAAKISGNPEVLWYRGKELIKDSADFQYKQDGNVFKLIIAEVFPDDTGVYKCIASNTAGSVTSSFYIKVEEPDIAPSGPVFVSHPKSQSLEEGTAFVASCTLDKADSVQWSKDGKDVESSERFKFSQDGNTFTFEIPAALATDSGEYTVTAKNSTGSSQWTFTLSVA | twitchin-like protein-1 [Mytilus coruscus] | AKS48140.1/0.0 | S_TKc(SM000220);IGc2(SM000408);IG(SM000409) |
| **CL3814.Contig2** | 3.8968 | AEDPVPEEDYEYTSVR | 1 | MPPPIAGHTPRKVTLNKFGGGTTSFGQSFQSNKKSSTTWQPVPAPAGGSMMNRVQDSLDSALSPTSPPQGYYQQQYQQPQQQQYRPPPQQQQYRPPPQQYQSPSQQQYQPPPQQQYQPPPQQYQPPQNQYQPQPQQEPYTPTYQTVGDLQPDYVRAEDPVPEEDYEYTSVRDRKKQFIETRQDAPLIKRGKKKFVPPVAAAYQSFGTDYSSPQPKQVEQPRFPPAPKPVPPPVNRAPEPVDQQDGPKPWAGSLRSESGGPKLWELEDKEYIMPSQLEARQQQQQQQRQSRGQTRQQRQPPAVSPKPISKGTNQIKVAVAPPQQSPARQISVRTSSTVSKQQPQQSQQQGDRDWNQSYVYKMVKEETKRETQMYPGQAPITTQTYSSKTYQSGQPAQEDTYGISDF | PDZ domain-containing protein-1 [Mytilus coruscus] | AKS48171.1/4e-91 | — |
| **CL3895.Contig2** | 2.1364 | GTFANIR | 1 | VTEAKVKMATGVNPYQQLVESVKVGEKSYQFFNVAALKDPRYEKLPFSIRVLLESAVRNCDGFHVNQKDVENILDWEKNQNSNVEIPFKPARVILQDFTGVPAVVDFAAMRDAVKRLGGDPEKINPVCPADLVIDHSIQVDVSRSTLRFSPNPGGGSCLKCEGPCSEKICPFHGAKTSSADALEQNQELEFERNRERFVFLKWGAQALKNMLIVPPGSGIVHQVNLEYLARVVFNDNGTLYPDSLVGTDSHTTMINGLGVVGWGVGGIEAEAVMLGQSISMVLPQVVGYKLMGEVDQMVTSTDVVLTVTKHLRQIGVVGKFVEFFGPGVANLSIADRSTISNMCPEYGATVGYFPIDNNSLDYLLQTGRTPEQTKLIEQYLRSVTMFRNYSNSEEDPVFSEVYELDLSSVKACCSGPKRPQDKVLVDDMKTDFTNCLNNKVGFKGFAIPSDKQLTTIPFVYDNKEYTLSHGSVVIAAITSCTNTSNPSVMLGAGLLAKNAVESGLTVQPYIKTSLSPGSGVVTYYLRDSGVTPYLEKLGFDIVGYGCMTCIGNSGPLPEPVTESIEKGELVACGVLSGNRNFEGRIHPLTRANYLASPLLVIAYALAGTVLIDFEKEPLGTNGEGKPVFLRDIWPTRKQIQELEKAVVVPTMFNDVYSRIQSGTTRWNSLEAPEGMLYPWDDKSTYIKSPPFFETMTKDLSGVQGVKDANVLLNLPDSVTTDHISPAGSIARNSPAARYLGSRGLTAREFNSYGSRRGNDDVMARGTFANIRLVNKFMKKPGPKTVHIPSGEEMDIYDAAQRYKQENKSVIILAGKEYGSGSSRDWAAKGPWILGIKAVIAESYERIHRSNLVGMGIIPLQYLEGQTATSLGLTGQETYTIELPSDLSPGHLTDVKLNDGRTFQVRVRFDTEVELVYFRHGGILNYMIRRML | cytoplasmic aconitate hydratase-like isoform X1 [Crassostrea virginica] | XP_022311953.1/0.0 | Aconitase(PF00330);Aconitase_C(PF00694) |
| **CL3951.Contig2** | 2.8554 | SGPFLVFR | 1 | MKDIVYIAVLFFTLFHVANSLCSHPCSRVNRGTFTDGTRNFVFGCTNTSVLQVYEGNRFIEDRECYARSGPFLVFRVGSRYQCFKDTVVDPNTNVVMIYFAPTQTFSTNPSICDVCAGEYTFALFVPRGNDLEAARRLPRPPLGCNRPPNCPILPDPYYIPCTGCEPKEDDGLCCSSCQDINNVYNRYGRGNQDRRNRDRRGRSNRFIGSLTQSFNNGRYSRPTKRSAKTC | hypothetical protein [Gracilimonas sp. 8A47] | WP_109647365.1/3.3 | Signal peptide(1-20) |
| **CL41.Contig2** | 2.2344 | STLEER | 1 | MSTLEERREARRRRRQQEEESGSTETPSEDSSSRRSRRRRGTEDDIDTTTTEEVSSYRSRRRRGQEEEETDSAPAPVSEPVSNDTEEEDRRRQQEAEEEAERQAAEAARRRAQEEEEAREREEQERRRREEEERRQEEERRRREEEERLERERLEAEEREREEAARIMEEQQKKKKGKRKGLGGLSPEKKKMLKKLIMQKAAEDLRNEAKAKAEEKERYINERVGSLSTEGKSEGDLVKLCKQLHQQLSKLEEEVYDWEVKIRKQDQEIIQLTLKVNDSKGKFAKPVLKKVNKTESKLNKIASKKDKSDFRDNLKSSGQSKYTLDEEGGESAE | trichohyalin-like isoform X6 [Crassostrea virginica] | XP_022318801.1/2e-55 | Troponin( PF00992) |
| **CL4276.Contig1** | 2.3583 | EDSYEETIR | 1 | MNDYNQSEKDLTMNDYNQSEKDLTMNDYNQSEKDLMDHMQLSQDSVSSYQLSQESVHSNVSQDLTSELLEGEVNETDKPKVRKKKSGPKKVKKKKSENTGDEAEEDKPKKKKKKVKITKRPEPEGGNAQPSLINPMTEFDRVIYSNEPLFSDLDMSDNENTNDMPERPTRAPRPGGVMIPCLQLGMHSNTMIKFAIIGSEIQNLLRVSLIRHEQEIQSLTRKISLLEEDIMKAEERFTTASGKLEEASKAADESERGRRALENKTFLDDGRIEQLESALKQTELIANEAERKYDEAARKLIVAETELERTEEKYDHMRRQVKTLEEELHLATNNLRGLEISEEKASQREDSYEETIRDLTNRLKDAEYRAETSERT | tropomyosin isoform X12 [Crassostrea virginica] | XP_022332784.1/4e-70 | Internal repeat 1;Tropomyosin_1(PF12718) |
| **CL4310.Contig5** | 3.8968 | FPGQLNADLR;ISEQFTAMFR;LHFFMPGFAPLTSR | 1 | MREIVHIQAGQCGNQIGAKFWEVISDEHGIDPTGTYHGDSDLQLERINVYYNEATGGKYVPRAILVDLEPGTMDSVRSGPFGQIFRPDNFVFGQSGAGNNWAKGHYTEGAELVDSVLDVVRKEAESCDCLQGFQLTHSLGGGTGSGMGTLLISKIREEYPDRIMNTFSVVPSPKVSDTVVEPYNATLSVHQLVENTDETYCIDNEALYDICFRTLKLTTPTYGDLNHLVSATMSGVTTCLRFPGQLNADLRKLAVNMVPFPRLHFFMPGFAPLTSRGSQQYRALTVPELTQQMFDAKNMMAACDPRHGRYLTVAAMFRGRMSMKEVDEQMLNVQNKNSSYFVEWIPNNVKTAVCDIPPRGLKMSATFVGNSTAIQELFKRISEQFTAMFRRKAFLHWYTGEGMDEMEFTEAESNMNDLVSEYQQYQDATAEEEGEFEEEGEEEDA | tubulin beta-4B chain isoform X1 [Heterocephalus glaber] | XP_004848801.1/0.0 | Tubulin(SM000864);Tubulin_C(SM000865) |
| **CL4656.Contig1** | 3.8968 | LNDQIGGYEGELANLR | 1 | MSQEKVEVRTRNTKTAQTMGPRSTIITRHSTSGTLPMAGTRSSTFRASYGGGGFGGAASFATGTVSGMSQKNVANVLDTRAKEKNEMNVLNERFASYIEKVRFVEAQNKALLAEIERLKKQKNFDVSEIKELYEQEIADSRKIIDDLSDEKAKFDSTLVSLQDQLEDEKRDRINAEKTVDDLRNKIDRLNDQIGGYEGELANLRLRIDSLEDENARLKKDKKTLQDDIARIRADLDEETCKRIQAEMKLQTAEEDFKFNQNIYEAEIAELRAMLDKDKSIEMKDIWKGEIQKAISELQAQYAAELDRMQGDMQKNFEMQLNEMKAGVNRDNMEALSAREESKKVKGKLSELQPLINQLQAENAMLKSRLDALQIQYDDECREHEEDRLKLESQIQKLSSELESILRELQILQDAKLSLELEISCYRKLLESEEQSLKRVVEESSGARSSGAQLLSDMIVTKGGSEASQKSSMSTSSRKVNLVKNSRGDLRFERCDPSGTKVTIKNNGTKSISMRGWRLIKNINGVDKCKFNFADDYTIGAQREVTICGKVMADELEYGELLGDFNTWGTHGKFILFDDKNVEKASMDVQVL | retrograde protein of 51 kDa-like isoform X4 [Crassostrea virginica] | XP_022320110.1/0.0 | Filament(SM001391); SCOP(d1ifra_) |
| **CL79.Contig3** | 2.8554 | VGSISDYDPMNDR | 1 | MNPPCAKCSKTVYPTEKLNCLDKIWHKGCFKCQVCNMTLNMKNYKGYDKLPYCNAHYPTTKHTAVADTPENRRIADNTKIQSNIKYHEDFERQKGKKLTVVDDPETQRVRQNTANISQVVYSGHKDQLKDMEYNRPAEQVNDVRVRPNPGSIHSYDPMRDQQQNQGTPYSQRNSGAQVYDSNTGREDRNFNARIQPFYQHPAYGMDNPSSQRRVGSISDYDPMNDRWGSVAGQFNAQNQQQRNQPPPPQQYAPPPQEADRFAGKGDKNDDDLSYGYDTTGKGMVCRAAYDYVAADDDEVSFNEGDFIIFCQPIDAGWMEGTVEATGRRGMLPSNYVETVKK | LIM and SH3 domain protein F42H10.3 isoform X6 [Lingula anatina] | XP_023930739.1/3e-101 | LIM (SM000132);NEBU(SM000227);SH3(SM000326) |
| **CL919.Contig2** | 2.1364 | QNLDLR | 1 | MDAFENVKEFSPPRQDEDELGKDDEDEEETDDEEDDDEDGDESGDEEEGDDDELGEDEEWVTDDDDAEDSQDVTLVTSDDDGEGGESCPICLNKFRDQDIGTPESCDHCFCLECIQEWANNVNTCPVDRQVFHLILAKHAYGDRVFKKIPVEDRKIEDEEDEDPTYCEICGRCDREDRLLLCDGCDQGYHCECLTPQLQDIPVEEWFCPDCAQTETEAVTVDDDEEIAEIISENAESLPSLRPQRRLIARTRASERVREQIIELRLRRADREARVTRRRTILLSDDDEDNSQPQAEISTQPSTSGALRKTPTKRKTPKAKRKTRKRKKTTKGAKKTPGKGKGKKKRRRKRKKVTKTSKRKAQIKKLVGKMVSREVRREVVPPASVKSRIAKNLGLSKPPTGTTIPLQKVPTDKGVDTARSNIGITQLSVMGDKNELIGFCDEPGDLIPSASKPSKKSKYSTLALFSHRPVGKPVIKIDQSPAASTGAAGFDLLGSIMDNQEMLGKDSSDVTIHRDGSLSKKAECKPVRKPSGQLLTPQKSQAELYDDLPPLEVSDLNSDLPSPEKETQTAGQNQISDESMKEIKYLDRQVDTTVSPDKSLDLSAVRNDFVETSKDTSFQIKKSNENSIIDNAGESDSNDRSDKNSVNCDIQNSESDLVTEEGSSKKKSLSTNKESGDQNSNSEKSADKSTDSLLESNNKTAKKADKNISNSSDKIISKSNSTTKKIEMASQLDDISDESNGFHDYLEEKELEEKLQQAQEKLRKLENKGKKDKKKELLKEKKKEVLKEKRPEKTNTGKEKTNEIESRKEKSDKVKKKLKESDKSEKKVVKKTKEAGNEKSKEKKCRGSKDEDDIMITTPPPQEIEVIEIPDDDDESEYVQNLDKKIIKSYSGDGSRKTKSRQNLDLRSVVKKEKTDKDEKENRKRRESERDHESDRNSKKKKRYRSRSKSRDRRHRSRSYERSHRSTSRSRDRYKHRRSRSRSRDRSRRSRSRERWSRDRSRDWSRDRSRGRDRSRDRERSRERNRSRDRDRSRSKDRSRKRRHRDKSKDRSTSREKNRMRSRDTSRERRRDSSPQVSLNISSLDSGRLDTGRLIDLFDDKWKKREDLKKSKKKKKDKGHESPIKSVQSPLKRPLEEDNDAEGNITKKSKTPVKEKTPEPVDSVQPIPKIIHVFPEKEKKEIDMFADDNSDMDTDIPEPAQDTVILINTVDNISNSQRPNLVPLPIVPGDDKKDDKVPEMISQVESNEVIPVINSANTDNITVASPEYDPAFPTDDMEESPLQDEKSPPGTPVEYTNPPPQTNLPLIAGEPPRLITVTQPLDEIFPDRVSPLNQLPPQLGKGLLGEGGPLILQRGEAPIIQPQDVQVGIRFLQGIRPGMSPQRAMLMNPVLMNRPPQMQPRFTRIPPGHLPQFGLQVTTQEGVALPVQGQFPPGMVAPGRLPPPQPGLINGSFEGIQPDTSQPPPPFQLLAGAGNHRLPGDPRLQLPPHVLAHGPPAAGAPLHLQPGPGMEQHVSIPSPLVSVSGPPMEGPHLTLQGPPQISLSGPLPRLALPNHPLSVPSSQIRIPSPDAPLGNPLTKLPARMPRLQIPASAALVSVEPAGILERPYTPPTPTNDDTPFMNQAVEASALAKLSRSQSPQTMPNISLLGGKPLMSINLKASKTLQNVNPLLSGKSGHLDEYPSPDENEGDDIIKDSGKTSGNVPQSQSTQLTQLTKLLNAQAQLAQLVSKGKTAKPSSKSGHHKSNDHKFKVPLPPKGKTSNGKVKVGESVDVIDMDVASPLDESSIEIPDSPDDFDKAVFGSKEKENKKRHDKHDRKSHKHKDRKAKASSSNVKLGGSNKKPVMSVNDENLKDVIRELEMDEVPSSAVELTNKEKFLKKLHLQERVVDEVKHALKPFYSQRRINKDQYKEIMRKAVPKVCHSKSGDINPQKIATLVEAYVSKYTKKQKKGEES | protein SCAF11-like [Mizuhopecten yessoensis] | XP_021339730.1/6e-108 | RING(SM000184) |
| **CL955.Contig1** | 3.8968 | CNMISGEVEELR;EIAFQADEDR;ELEGELDSEQR;ELEGELDSEQRR;ESYNLAER;GQLEISNVR;LAEKEEEFENTR;NLYSTHPHFVR;SSVSISR;VGLSVIQR;VRELEGELDSEQR;YQQQVSEVQR | 1 | TASVLHMGEMKFKQRGEQAEPDGTAEAEKVSFLLGVNSNDFVKCLVKPKIKVGTEVVAQGRNKAQVMNSISAMAKSLYDRLFAWLVKRVNHSLDTKAKRNYYIGVLDIAGFEIFDFNTFEQLCINYTNERLQQFFNHHMFVLEQEEYKKEGIQWEFINFGMDLQACIDLIEKPMGILSILEEQCMFPKADDKSFKEMLFTNHMGKSPNFTKPGKAAKGKNGDFELHHYAGSVPYNIAGWLDKNKDPINETVVELLQGSKEHLVVTLFAPPEGAEATGGTKKKKKSSAFQTISAVHRESLNKLMKNLYSTHPHFVRCIIPNEMKQPGVIDAELVLNQLQCNGVLEGIRICRKGYPSRIIYAEFKQRYSILAPNAVPQGFVDGKVVTEKVLLALQLDPAEYKLGNTKVFFKAGVLGNLENMRDERLGAIVSMFQAHIRGYLIRKAYKKLQDQRVGLSVIQRNIRKWLLLRNWQWWKLFAKVKPLLNYAREEEEMQKKMEMMKKMEEDLAKTEKIKKELEIKNVELLEQKNDLFLQLQTQEDTVIDLEERVQQLVNQKCEFEAQMKEMEERLLDEEDAAAELENVKKKMEGENSELKRDIEDLETTLAKAEQDKTTKDNQIKTLQGEMAQQDEQIGKLNKEKKNMEELQKKTLEDLQKEEDKVNHLNKVKQKLEQTLDEMEDSLEREKKIRGDVDKAKRKVEQDLKATQELVEDLESNKRELEEANRKKDSEMSMLNSRIEDCEGVNAQQNRKIKDLMATIEELEEELEAERAARAKAEKQRAELARELDEISSQLEEQGGATQAQVDLNKKREQELVRLRREMEEMTLQNESQVSQIRKKAQDQANELADQIDGLNKLKSKLEKEKKDLKRELDDVQSQVQYSMKNKGVSDKVAKQMEVQISEMNSRVEESQRTIVDINSLKTKLQSEVADLNRQLEDAEHNIGSLTKDKTSLNHQLEESKRSLEDETRTRQKLQNEIRNLNADVDSIREAFEEEQESKSDLQRQLSRAKNEAQQWRSRFETEGTAKADELEEAKRKLAARLAEAEQNAEAANAKASGLEKAKNRLQGELDDLLVEIERSNVSSSTLEKKQRQFDRTIQEWTTKVKELQTEVDTAQAEARGYSAELFRSKAQYEECNSTIESLRRENKNLADEIRELTDQLSDGGRNAHEVEKAKRRLEMEKEELQAALEEAESALEQEEAKVMRGQLEISNVRSEIERRLAEKEEEFENTRRNHQRALDSMQASLEAEAKGKAEAMRIKKKLEQDINELEIALDASNRAKAELEKNIKRYQQQVSEVQRQVEEEQRQKEEVRESYNLAERRCNMISGEVEELRTALEQAERARKGAENELFEANDRVNELSAEVQSISSQKRKLDGDIQAMQSDLDEMNNEVRNADDRARRAQEDSARLADEIRNEQEHSQQIEKFRKSLEGQVKDLQVRLEEAESQALKGGKKMIAKLEQRVRELEGELDSEQRRHAETQKNMRKADRRLKEIAFQADEDRKNQESLNSMIDTLNAKLKTYKRQVEEAEEIAAINLAKYRKVQQELEDAEERADSAEGSLQKLRAKNRSSVSISRSSVTHTPATSPSVLNSSNLLSPRSMSRGPDSSFLSPRSASRGPGLYRRSVTPSYEDDDY | pedal retractor muscle myosin heavy chain [Mytilus galloprovincialis] | CAB64663.1 /0.0 | MYSc(SM000242);IQ(SM000015);Myosin_tail_1(PF01576) |
| **Unigene11395** | 2.1364 | QTSSQTASKSR | 1 | NRKYLVHMNDENDFLLSHINEQGVDLKLIASAPLPLSDAQLSKSLEKTVDGPNRITVSPDSYATILRGFPDSSEVEVFSIPRTPLAESTPSGKSDPMLGLLGKQTSSQTASKSRGAILHLPKSGQIVRALPSWKVPDYVYEKDQKPKDVSGFLEVTDVVNRTLRYIP | — | — | — |
| **Unigene13309** | 3.8968 | GILGNSGGSGGGLLSR | 1 | QLLRAQPQGSTENLWEDELNNTMRILSIFVFLAALACADALLSDLLRLQVLKGILGNSGGSGGGLLSRLGQSSNGAQSASVVSSSSAGSYMENYYKLQYCRETPFRFIRKCTTSSQCSPYLECFENVCCATNPLSLRIVD | uncharacterized protein LOC110459472 [Mizuhopecten yessoensis] | XP_021367428.1/ 8e-04 | — |
| **Unigene14034** | 3.8968 | DTDIAGVTQDSR | 1 | ASHQQNQQQQQQIQHTEHREYKYNTQNFNYNQQPNQQFMTGSQQHINMTEGMPAQNIPIQHQQGRYQQQQNQHQQQLQYHTGSQQNINNMLDGMPVQNMPIQHNHGGSQFNTMSSGHGSVSSPQGFNTLGSNQSYRTEQHQKYHTMSSSGSHDGFGTMGSQNFGTMSSGHGYNTMGSQQNGSLHVDTTNRSMHSGPSSAGSPHSPDTLNALRQQLHVAHNMSSSSGGALSPGPHSMTGQSSPSVYFGMSRRGSLTSLADTADAVHATPKFVKNTSKYWYMPNITREEAISMLKDKAPGTFVVRDSNSFPGAFGLALKVATIPPNVQTKSSGDPAADLVRHFLIEPTPKGVRLRGCSNEPVFGSLASLVYQHSITPLALPCKLVLPEVDPSIESSMDVTHTSEQPSSAAALLAQGAACNVLYINSIDTESLTGPQAVARALKLTFDTAPSPTTTVVHFKVSNQGITLTDNQRKLFFRRHYPVSAVTYCGMDPESRKWKRDTDIAGVTQDSRVFGFVARKHSGASDNACHLFAELDPEQPASAIVNFVTKIMIGQGSKK | Tensin-1 [Mizuhopecten yessoensis] | OWF49222.1/3e-158 | Internal repeat 1;SH2(SM000252);PTB(SM000462) |
| **Unigene14496** | 3.8968 | SNTLQNLHVQQPAR | 1 | GACCGAPPSPEMGMRSNTLQNLHVQQPARQQYETQASILTNFNEMANVYVDKNAQKTPAKNESDVLSPDYRPYLPPSTYSPFGPYGAPSQPNNGNYDQNAQIKK | — | — | — |
| **Unigene15208** | 3.8968 | DADEDGDGLLSR | 1 | MRLTLGILCVVISSVVAPPPLDVELRAEKEYLEQLKQQQEDILDTLGDNLIPDSEEEKAIEDTDAEDPIVLKKDEAEFVPISFPSKFDKYDENGDDFIDEGELITVIGVSENIALALKDADEDGDGLLSRDEFENGPWDLDATDLEDVEAMDDLMDENEDLIDKEFIDYDSESFNDILEGDEGMEDEDNEIVDDIEDMKEDFEEDRHERTDINGENDVKEKVEEVVNDLQENIKDVRENVKDINQNVFEKIGDSKK | uncharacterized protein LOC110443432 [Mizuhopecten yessoensis] | XP_021343328.1/1e-09 | Signal peptide(1-17);Efh(SM000054) |
| **Unigene15218** | 2.8554 | QAVDVSPLR | 1 | MTETWDEPATTVAELPEIKLFGKWSSDDVQVSDISLTDYIAVKEKYAKYLPHSAGRYQMKRFRKAQCPIVERLVCSLMMHGRNNGKKLLAMRIVKHAFDIIHLLTGENPLQVLVNAIINSGPREDSTRIGRAGTVRRQAVDVSPLRRVNQAIWLLCTGARDTSFRNIKTIAECLADELINAAKGSSNSHAIKKKDELERVAKSNR | 40S ribosomal protein S5 [Mizuhopecten yessoensis] | XP_021375269.1/9e-143 | Ribosomal_S7(PF00177) |
| **Unigene17159** | 2.6312 | EGQGYISGAEMR | 1 | MSKLSKGEIEDAREVFDLFDFWDGRDGDVDAAVVGDVCRCLGINPTNAVIKKNGGTDKMGEKGYKFEDFLSIYETVNQQTEQGTYADYMEAFKTFDREGQGYISGAEMRQVLSSLGEKLTDEQVDEIIRLTDLQEDLEGNVKYEDFIKKVMAGPYPD | myosin essential light chain [Crassostrea gigas] | CAD91423.1/3e-88 | EF-hand_7(PF13499) |
| **Unigene17193** | 2.8554 | GLVVPVIR | 1 | LKSMALLLVQRCLPRITRRLSPTNTKFVLEEASKNIRVKSHICCSSQCRNYTDVQYIRFTKKYCCNSQLIIKRNFHVTNRYFDDVLTAATPPFADSISEGDVRFEKAVGDFVKEDEIVCEIETDKTSVPVQAPKSGIIQSFLVDDGATVQAGTPLFTLKLSDSPGESAPESVAASEKPPPPAVTSKAPETPVATPASGPIPTTPPPPQPIPKAPISTKPLDSIKPIPATDAPVMGARTEKRVKMTRIRQKTSQRLKAAQNECAMLTTFNEIDMSNVIEMRNQYKEAFQKKYGLKLGFMSAFVKAAAYALTDQPAVNAVIDESEILYRDYIDISVAVSTPKGLVVPVIRNVGSMNYADIERAIAELGEKARTGSLAIEDMDGGTFTISNGGVFGSLFGTPIINPPQSAILGMHAINDKPVAIKGKVEIRPIMVVALTYDHRLIDGREAVTFLKKIKSAVEDPRVLLLDL | 2-oxoglutarate dehydrogenase complex component E2 [Mizuhopecten yessoensis] | OWF50391.1/0.0 | Biotin_lipoyl_2(PF13533) ;2-oxoacid_dh(PF00198) |
| **Unigene17750** | 3.8968 | FNTIAECMNACR | 1 | KNFGMMWLPLGFITLAIFHGVNGQAANAKQKAAKTVGKMDKKCFDNPRTGMCMGMFQAKWFFNEATGKCVMSQGCFYQGFISMQECRKECQCRQPLNEGSGSGPVGANCELEVQKYAMVGEVCTPFMFTGCGGNGNRFNTIAECMNACREREPLDMMGEMMPGMMGWAGMSGMGGGMMGNMNFRGA | proline-rich protein HaeIII subfamily 1-like [Crassostrea virginica] | XP_022333931.1/ 2e-29 | Signal peptide(1-23);KU (SM000131) |
| **Unigene1886** | 2.1364 | QNPGGFVER | 1 | FPLYWTSDTMASTKVSKSQDKRADSYRYAVTPGGDDGKKKKSKKQKKKENLDELKQELEMDEHKVPIDELYERLGSNPNTGLSVEEARRILERDGPNALTPPPTTPEWVKFCKQMFTGFSLLLWIGAILCFIAYSIQASQDENPPGDNLYLGIVLTAVVVVTGCFSYYQEAKSSKIMDSFKNMVPQYATVVRGGSIFEVKAEEIALGDIVNIKFGDRVPADVRVITAHGFKVDNSSLTGESEPQTRTADFTNENPLETRNLAFFSTNAVEGTCKGIVVKTGDATVMGRIANLASGLDVGTTPIAKEIAHFIHIITGVAVFLGVSFFIIAFILGYFWLDAVIFLIGIIVANVPEGLLATVTVCLTLTAKRMASKNCLVKNLEAVETLGSTSTICSDKTGTLTQNRMTVAHMWFDGRIYEADTSDDQTNATYGKNDESWMALSRISMLCNRAEFKAEQDNVPVLKRECTGDASESALLKCVELSIGKVTQFRQKNKKICEIPFNSTNKYQVSIHETDNPNDPRYLLVMKGAPERILDRCSTVLFQGKEIPLDDNFREHFNSAYMELGGLGERVLGFCDYFLPSDQYPIGYAFDSDEQNFPLTGLRFVGLMSMIDPPRAAVPDAVGKCRSAGIKVIMVTGDHPITAKAIAKGVGIISEGSKTVEDIAAERGCPIEEVEPSEANAAVVHGSDLRDMTPAQIDEILKNHPEIVFARTSPQQKLIIVEGCQRQGQIVAVTGDGVNDSPALKKADIGVAMGIAGSDVSKQAADMILLDDNFASIVTGVEEGRLIFDNLKKSIAYTLTSNIPEISPFLMFILLDIPLPLGTITILCIDLGTDMVPAISLAYEGPESDIMKREPRDPVKDKLVNERLISMAYGQIGMIQASAGFFVYFVIMVENGFWVSRLLGLREEWDSMAVNDLQDSYGQEWTYGQRKILEYTCHTAFFVSIVVVQWADLLICKTRRLSIVQQGMKNHHMTFGLFFETALAAFLTYCPGLDKGLRMQNLRATWWFPAMPFSLAIFVYDESRKFILRQNPGGFVERETYY | eukaryotic initiation factor 4A-I-like [Crassostrea virginica] | XP_022288944.1/0.0 | Ribosomal_S9(PF00380) |
| **Unigene19938** | 3.8968 | EAYPGDVFYLHSR | 1 | LQSAILVGHRREQELVKMLSARFAATLVRQLPRAAPKVCRHALGAGYVASRNISTSTPLCAGAEVSSILEERILGQTSQTNLEETGRVLSIGDGIARVYGLKNIQAEEMVEFSSGLKGMALNLERDNVGVVVFGNDKLIKEGDIVKRTGAIVDVPVGKEMLGRVVDALGIPIDGKGPLGTSTRARVGVKAPGIIPRISVKEPMQTGIKAVDSLVPIGRGQRELIIGDRQTGKTAIAIDTIINQKRFNDGTDEKAKLYCIYVAIGQKRSTVAQIVKRLTDADAMKYTVIVSATASDAAPLQYLAPYSGCAMGEYFRDNGMHAVIIYDDLSKQAVAYRQMSLLLRRPPGREAYPGDVFYLHSRLLERAAKMNDDNGGGSLTALPVIETQAGDVSAYIPTNVISITDGQIFLETELFFKGIRPAINVGLSVSRVGSAAQTKAMKQVAGSMKLELAQYREVAAFAQFGSDLDQATQNLLNRGVRLTELLKQGQYIPMPIEEQVAIIYAGVRGHLDKLDPTKITDFEEAFLQHIRGSQKDLLATIAKDGMITEDSDAKLKQVVKNFLAGFEG | mitochondrial H+ ATPase a subunit [Pinctada fucata] | ABJ51956.1/0.0 | HAS-barrel(PF09378);ATP-synt_ab(PF00006);ATP-synt_ab_C(PF00306) |
| **Unigene20162** | 3.8968 | GMTAMGAVR | 1 | ISHVGTEAQRLNYNGPTIGAKPTEKRAVKFSYEQLKQSCGLIGLQSGTNKFASQRGMTAMGAVRHISDIRADKFSKEAEGEINLQSGTNKFASQRGMTAMGAVRHICDIRADQYDPESNKEINLQSGTNKFDSQAGMRGFGAIRHISDVKVNELDREGTSVLRLDMGYVGGDSQKGMTSFGAQRHITNVKVNDLAEEFALQHGKPAPTPQPQAVEEVAQEEEEE | calponin-like protein-2 [Mytilus coruscus] | AKS48163.1/2e-125 | Calponin(PF00402) |
| **Unigene20241** | 2.2344 | VTGVITQGR | 1 | SGSGLKIAVFICLFGLIYCNVCVTNGPLGMITGAIQDWQITASSTYPKAWDKKCSEKYARVYLPNKYGWCSKYKSSSEWLKIDLGVAARVTGVITQGRGDGKEWVTSFKVSYSMDDYNEQYVTDQYENHKVFEGNTDAFSIKHTYLDR | Lactadherin [Crassostrea gigas] | EKC24096.1/7e-80 | Signal peptide(1-19);FA58C(SM000231) |
| **Unigene20294** | 2.8554 | MGHAGAIIAGGK | 1 | PVLTMSTATKVLGKVSAIGRVGIRTCYTNSRPNLGINKKTKVICQGFTGKQGTFHSQQAIEYGTKMVGGVSPGKGGQKHLGLPVFNSVKEAREQTGADASAIYVPPPFAAAAIIEAIDAEVPLIVCITEGIPQQDMVKVKHKLIRQSKSRLVGPNCPGIIKPGECKIGIMPGHIHKRGKIGIVSRSGTLTYEAVHQTTQAGLGQSLCVGIGGDPFNGTNFIDCLEVFLQDPQTHGIVLIGEIGGQAEEKASEYLRNNNCGSDAKPVISFIAGVTAPPGRRMGHAGAIIAGGKGGADEKIEALREAGVDVTMSPAQLGTTMAKAMSAAGKL | succinate--CoA ligase [ADP/GDP-forming] subunit alpha, mitochondrial-like isoform X1 [Mizuhopecten yessoensis] | XP_021357524.1/0.0 | CoA_binding(SM000881);Ligase_CoA(PF00549) |
| **Unigene20448** | 2.8554 | APGQAWGFR | 1 | MATVQVQRAPGQAWGFRLAGGRDFNVPLQIKKVEQGSPVAGVLSPGDNIIGIGHSDARNMTHMQANQMIRGAGNMLQLTIVKGHGDVNSRISSIKPKGPVKFSPWKAQST | — | — | PDZ(SM000228) |
| **Unigene20852** | 2.8554 | YGLEDYIEIK | 1 | SADIEKAVTGVMGSKFRNTGQACICANRIFVQDSIYDKFVSSLANRMTKELHIADGFDDKATQGPLINQRAVDKIESLVNDAKEQGGKVVVGGQRRKGNFFDPTLISDVTTKMRCYNEEQFGPLAAVIKYHTDEEVISMANNTSSGLAGYVFTENINQMWRIAEKLEYGIVGVNEGLPAMPEAIFGGWKESGLGREGGKYGLEDYIEIKYVCLGGLSD | succinate-semialdehyde dehydrogenase, mitochondrial-like isoform X2 [Crassostrea virginica] | XP_022301488.1/2e-93 | Aldedh(PF00171) |
| **Unigene22896** | 2.2848 | QKEALVEQEK | 1 | RHTRFRQKCFKNITTDHRMSAVEPSRSMLPSITPRTAPSTAPIGIGRNKAFPPVPKRYEETKPVVIQGHAHRMIIGIGNELQRRSLEVNELDKLRAIREAEQAVWAEAEQIKAEAVQRAKEEARLDQEKMLKKVSKAQEKALKEEALRVEMAMQKLAIEQVKQERLEAEKRQKEALVEQEKLFTKTLSEAVATARKEEQTTAAKKAADVDKANKEELASALKKSEEQKKKALADLESSKNKEMAAKISKTEETERKKANDTLQATNKKYEAEIAKLKVEIKEKQAEISKHLKNIQAVEVAKSNVEVKLLELHSEYQNFINRVQPFEPGMADYLIKPVYIDEIGKVPDH | — | — | — |
| **Unigene23744** | 3.8968 | TIDTMSLTADHVLADR | 1 | IQISKSDSNQNGLLNARKILSKTIDTMSLTADHVLADRIRSDKIKTTNLRVTDKRKSKVSYYHYDVETPDVTRTNLPVTNKSPTPYSGGDHHSFNRKSKKSGHFHNNDFVHRKVSRGYSKTSTDSRRKILNIQRPRQSSTDKYFESTKVRIKPKNMQNSFEVEYLAPYAVTKPNMAMFIESPTTIRRLSTPDTSNRHYQEYLGYGELNFDPVLPTVDHLQ | — | — |  |
| **Unigene23964** | 3.8968 | YDFILSANQPIR | 1 | RLEGYHDHPCIRQCEEDEEPKECTYDFTVEYYYTLTQACYDCPYNITDCLRPHCVSADGISRGLITVNRMLPGPSIHVCKGDTVVVNVKNKLEGGEGVSLHWHGVLQEGTPHMDGVSMLTQCPIHPYTTFQYRFKANDPGTHFWHAHAGLQRTDGIFGSFVIRQPRAHDPHSILFDDDLPEHTIMLNDWMEHIGAVSFAAGHHAMKDHFPTSILINGKGTVKEFNEMMDHSAHKDSALPEDMIMDTLTTTRTMEMNHVDQMFEHMMHRRSSENQEMPPSHNMDNMPHSMTDLDARHTPHSIFNVKQGKRYRFRVISNGIANCPIQFSIDHHSLTMVTTDGSPFNPITVESFTVFAGERYDFILSANQPIRNYWIRARGLAECGPQYKSVSQTAFLTYEGSAIVLPQEPPDYHSGNRPGLMLNPLNIGPSRGFITVD | laccase-2-like [Crassostrea virginica] | XP_022312912.1/3e-127 | Cu-oxidase_3( PF07732);Cu-oxidase(PF00394/) |
| **Unigene240** | 3.8968 | AAAEAAAAAVAAEQAIADAEK | 1 | MRTTVFLACFVVCVAVASAHFWHPVIKIPGQKWCTCRPKCQKNEFTAVDGSCGLKILFQTSYDTCCKVLPNFVTNRYPSGIRSIVVRRLGGNGGAGGNGGNGGAGGNGGNGGAGGNGGNGSNGGNGGNGGEGNGAAEKAAETAAAEAAAAAAEMEAAQAKAKAARAAAEAAAAAVAAEQAIADAEKAAADAAAAKAEQAAAEAEEAASKAQAIADAAEKAAAAAQVADEEAAAAAEEAEKAAGEAKEAKEDADKAAAEAGPEVALVGDDAGKDDGGDEGGRGPTNIKIVLAGRRGGFKPYKPFPKYA | — | — | Signal peptide(1-19);SCOP(d1dqca;chitin-binding) |
| **Unigene24249** | 2.1364 | IIFDDFR | 1 | FALRKMKTSQVPWGLGWLYRLSPEKQDTMLKMMVLTIAAILSFSTRLFSVLRFESVIHEFDPYFNYRTTRFLAEEGFYNFHNWFDDRAWYPLGRIIGGTIYPGLMVTSAVLYHVMWFFHITIDIRNVCVFLAPLFSSLTTLVTYQFTKELKDAGAGLVAAAMISIVPGYISRSVAGSYDNEGIAIFCMLLTYTLWIKSVKTGSVFWSALCSLAYFYMVSSWGGYVFLINLIPLHVLALMATGRFSHRIYVAYSTVYCLGTILSMQISFVGFQPVQSSEHMAALGVFGLCQIHAFIDYVRSRLNAEQFNVLFRSMVLLVGILGTAAAAVATATGKISPWTGRFYSLLDPSYAKNNIPIIASVSEHQPTSWSSFYFDLQMLVFMFPAGLYFCFSRLTDANIFIIMYGVTSIYFAGVMVRLMLVLAPVMCILSGIGVSATLTTYMKNLDVKKDKKSKRADSTYPMKNEVASAVVFMVTAFLITYTFHCTWVTSEAYSSPSIVLSARSGDGGRIIFDDFREAYYWLRQNTPEDAKVMSWWDYGYQITAMANRTILVDNNTWNNTHISRVGQAMASPEEKSYEIMRELDVNYVLVIFGGLTGYSSDDINKFLWMVRIGGSTDRGSHIKEADYYTPQGEFRIDKDGSPTLLNCLMYKMCYYRFGQVYTEGGKPPGYDRVRNAEIGNKDFELDVLEEAYTSEHWLVRIYKVKDLDNRGL | dolichyl-diphosphooligosaccharide--protein glycosyltransferase subunit STT3A [Mizuhopecten yessoensis] | XP_021367729.1/0.0 | STT3(PF02516) |
| **Unigene2452** | 2.2344 | IQTQPGWSSTLR | 1 | LLISPFRRNTPRRRFKMEAISKAVKNNIFSSPFQQAKCEGLKVNETRTIAAASANAEEISCEFGSNKYYALCGFGGILSCGITHTAVVPLDLVKCRIQVDPGKYGGIVNGFKISVKEGGAKELTKGWAPTLIGYSMQGLCKFGFYEVFKIVYGNMLGEENTFLWRTSLYLAASASAEFFADIALCPMEACKVRIQTQPGWSSTLREGFPRILKEEGVSGFYKGLVPLWARQIPYTMMKFACFERTLEALYKYVVPKPRSECNKTEQLMVTFSAGYIAGVFCAIVSHPADTIVSKLNQDKGSNFVDIAKRLGLLGMWKGLFPRIIMIGTLTALQWFIYDSVKVYFRLPRPPPPEMPESLKRKMGIA | Phosphate carrier protein, mitochondrial [Crassostrea gigas] | EKC26686.1/0.0 | Mito_carr(PF00153) |
| **Unigene24901** | 3.8968 | EPEVSNVQLR | 1 | SSMHLVLAFVLLALIVQQSLQCTCDWRWRQVDGENVPNTLCYEDGEYIVFKLDKKERIPGRSGYDEIDGRMHFDVTVSKYITKAAGEQVLPEGRKVTLDAPIGSSLCGYIDFKPGVTHVLSIHGGLKDKINFNACSFYSREPEVSNVQLRLMSGKLTCPKDTDPMLGHF | serine hydrolase [Lentzea waywayandensis] | WP_093592321.1 /0.026 | Signal peptide(1-21) |
| **Unigene2555** | 2.2344 | FPGQLNADLR;LHFFMPGFAPLTSR;YLTVATIFR | 1 | SESCECLQGFQLCHSLGGGTGSGMGTLIISKIREEYPDRIMNTFSVVPSPKVSDTVVEPYNSTLSVHQLVENTDETFCIDNEALYDICFNTLKLKNPTYGDLNHLVSLTMSGVTTCLRFPGQLNADLRKLAVNMVPFPRLHFFMPGFAPLTSRACKDYRAVSVQELTQQMFDAKNMMAACDPRHGRYLTVATIFRGHISMKEVDEQMLNVQNKNSSYFVEWIP | beta-tubulin [Papilio machaon] | BAN92310.1/6e-141 | Tubulin(SM000864);Tubulin_C(SM000865) |
| **Unigene26867** | 2.6312 | IVVYLNK | 1 | FFPRTFFAIWGTSKYQIRQLLPRNRLTILTIWRNYCKVANSTSKPRCNVGTIGHVDHGKTTLTAAITKVLAKHGQSKLVTFDQIDKAPDEKKRGITINTAHVGYETSKRHYAHTDCPGHIDYIKNMITGTSQMDGAILVVAASEGSMPQTREHLLLAKQIGVDKIVVYLNKMDLVDDELGDLVELEMRELLEEYGYDSTKTPVIRGSA | elongation factor Tu, mitochondrial [Culex quinquefasciatus] | XP_001843948.1/1e-84 | MMR_HSR1( PF01926) |
| **Unigene26873** | 2.3583 | EGANINKSLTTLGK | 1 | NMNETSSRSHAVFTIIFTQKRHDISTDLTGEKVSKISLVDLAGSERADSTGATGTRLKEGANINKSLTTLGKVISALAECANKKKKKENFIPYRDSVLTWLLRENLGGNSKTAMIAALSPADINYDETLSTLRYADRAKQIMCKAVVNEDPNARLIRELKEEVAKLREILQHE | kinesin-like protein unc-104 isoform X4 [Mizuhopecten yessoensis] | XP_021372883.1/8e-96 | KISc(SM000129) |
| **Unigene27944** | 3.8968 | TELEEIAAENPGR | 1 | HIFVTVAANRSMGRALTVGLPVAIGLGVVAVTAILAKIFLFGNKKKKSPVTLEDPEKKFPLKLIDKEEVSPDTRRFRFALPSPEHILGLPIGQHIYLTARIDGQLVIRPYTPVSSDDNKGFMDLVVKVYFKNVHPKFPDGGKMSQYLENMDIGDFIDVRGPSGLCVYDGQGVFKIKPDKKSEPETVMAKKLGMIAGGTGITPMLQLIRACFKDRNDTTQIFLLFANQTENDILLRTELEEIAAENPGRFKLWFTLDRPEDDWKYSKGFISADMIKDHLPPPGDDTLILMCGPPAMINFACLPNLDKLGYTPKMRFAY | NADH-cytochrome b5 reductase 3-like isoform X1 [Crassostrea virginica] | XP_022332541.1/1e-178 | FAD_binding_6 (PF00970);NAD_binding_1( PF00175) |
| **Unigene27989** | 2.8554 | SGFGAAGFGVGGGR | 1 | MGSGKGMGNGGRGMHMEHGMGSGGDGMGMGNGGVGGSLNVWDHLSSNTGSSGSGSGRNPANNGRSGFGAAGFGVGGGRTLSNMGGSMHKNGGMGSGLASHGTNVWNRVNGQSNNKPMSNSGSVLSGPGGARNNGVANMNNGGTPLVDQGTKSKPVEHPPTPPASMSSVGGGFGRASDISFHTMGSMLQPNQGGSVSQPQSKNQGQTQPKQPGNATPQVSNQNPANSGSSGGGVGVNPQTNQPATNSQNSGPTGNNIPGINNGGSQGGNVGGSHGGNMGGSQGGNMGGSPQIETTGGATSAPNHNFGQGNSFFGHGTNNQGNLGTNNVVGTTAYPGFTSMATWMFLK | — | — | — |
| **Unigene2937** | 2.2344 | QLLDAGGHAHNR | 1 | SSSAFTAYNQSSTATTGGGAGMGGGSGMSGGGAGGGYGMSSGEFSSTTTRTIMGGGGSGGATGFGAGYSSGGQSSSYSANKMYSAMDAGESTTADYDLASGSGTMMGGSGGASNTMNKYSTMSSSYQVQNVRTVLVNNQGTQQLVTYHSASGAVTPGAAGDEVRVDCCLMNEDGRTVVTGSTLGPPQVWNMQTGELLRIMKGDTVGSTNLHLACNDRLLVGAVHADLEINEYSTRKGVTVKKLQIWDFVTGKPLDMGVDETCSALCVMSDPDKVVFGRSDKFGNATSIVVWDLLGNQPIKEMRYDAPVGNNDYINFLALSQNDRYCVAGFTNSFDNYAEFVVFDMTLTSYNINDANVLRLDANPECTAILPQEEACTGLRNGDLVVWKLRTGQADRQLLDAGGHAHNREVKAVTLSQDNRYLVSASADNTMKLWDMTIEKPVQTLRGHTDEVWCSAISVDNEIIVSGSKDGTIRLWRTKNGSEICAFNTGVDVFNVTMSQDKGTIVALGDKFGARKLIMLQVVRTKVRRQTSS | WD repeat-containing protein 5B [Mizuhopecten yessoensis] | OWF43129.1/0.0 | Internal repeat 1;WD40(SM000320) |
| **Unigene31394** | 2.6312 | ALNELFPDWK | 1 | GCKTVSRIVSVCKHVRLYSASPKITTHYSVHPRENDERWKDVDMTRYEDEADVLIVGGGPAGLSAACRLKQLANEHGKELRVCLVEKAPEIGAHTLSGACIEPKALNELFPDWKEKGAPLNTLVTEDKFSLLTEKSAIPIPVFSGMPMANHGNYIVRLGNVVRWLGQQAEELGVEIYPGYAASEVLFHEDGSVKGIATNDVGIHKDGSPKETFERGMELHAKVTIFGEGCHGCLAKQLYKKFDLRKDCEPQSYGIGLKELWEIDPEKHRPGRVEHTIGWPFDSHTYGGTFLYHLDEGPLVTLGMVVGLDYQNPYISPFREFQRFKHHPSIESTLSGGKRIGYGARALNEGGFQSLPKLTFPGGCLAGCSPGFMNVPKIKGTHNAMKSGMIAAESVFETLHNETLVSNSPTAGLNPEMYEEQIRKSWIWDELYSVRNVRPSFNTRFGLYGGLAYTGLFYVLLRGKEPWTLSHGGADHTKLKPAAECTPIEYPKADGKISFDLLESVALTGTNHDHDQPAHLTLLNDDTPANLNLSVYNGPEQRFCPAGVYEYIETEDGSGKRLQINAQNCIHCKTCDIKDPSQNINWVCPQGGEGPAYNGM | electron transfer flavoprotein-ubiquinone oxidoreductase, mitochondrial-like [Mizuhopecten yessoensis] | XP_021375935.1/0.0 | NAD_binding_8(PF13450) ;ETF_QO(PF05187) |
| **Unigene32880** | 2.8554 | IVDSPVVLTGR | 1 | SKVSGGPIIASSVDGVLRGSDPLDFGSSGAVLRKGSSLLDDRSTSFIGRGESFGFESSIGGPIVRKVVSGNIDGTTVIKKVVGGRTGLGESRVVLDDRNRIGDSPVVFTGRNRIVDSPVVLTGRRGLGDSTVVLDDRTGLGSQIVLKSVGHGGSRRGSVGVIKTIDGAIRTTDVLDAGVRSGISGRTIGVSESGILKGALVGSTGSSCRQACYRDADCFGSKVCVTVGCSRVCRRSESNGYSR | hypothetical protein AM593_05232 [Mytilus galloprovincialis] | OPL32726.1/ 5e-04 | — |
| **Unigene34096** | 2.6312 | TDVLLDINR | 1 | TTNWIDGLHQQSGNFNLYLAGNEFSCVCDYKSFIDWLSRTDVLLDINRNYSCTFPNGTRIRIPEVIQNYHRIFSHCNAIAWLRTGVICIVSSFFVIGLTAIIYQFRWRFTYFMYRQLKSRYIKEDPFVFDFVYDVFVAYANDCSEWLVESLIPTLEQEWNLNVCIKDRDFPIGADRGDTVVQS | — | — | LRRCT(SM000082）；SCOP （d1fyva_） |
| **Unigene34337** | 2.6312 | VDEHGEFLWPGFGDNIR | 1 | ETRSKGRRSVLLSTLHLQFFLQVNPDQKMSVFGEEDTQFYEVHNIVVQHIGPVPIAKGDFHMLPKKVQKFIAKWVDICAPRALYICDGSQHEADEITHKLLERGVLTTLPKYENCYLCRTDPADVARVEAKTWISTEDRYETVPHVREGVKGCLGQWIAPKDLEKEMGERYPGCMKGRTMFVIPFSMGPVGGPISKIGIQLTDSNYVLLCMRIMTRVSPVVLDKLGDGDFVRCVHSVGCPRPVQRKVVNHWPCNPEKIMIAHRPKEREILSYGSGYGGNSLLGKKCFALRIASVIARDEGWLAEHMLIMGLTNEKTGEEKFICAAFPSACGKTNLAMLKPTIPGYKVRVVGDDIAWLKFDKDGVLRAINPEAGFFGVAPGTNMKTNPNAMLTFQKNSIFTNVAETADGGFYWEGMEDEYDKNMLITTWLNQKWHIGAPGKAAHPNSRFTCPASQCPIIHPKWEAPEGVPISALIFGGRRPTGVPLVFETFSWEHGVSVGACVKSEATAAAEFKGKVIMHDPMAMRPFMGYNFGNYLQHWLDLNAPPHKMPKIFHVNWFRVDEHGEFLWPGFGDNIRVLDWVLRRCAGEQNIAEETAIGYVPKKSSFNLSGIEDQVKWEELFSLPKHYWLDDMRESRRFLEDQVGSDVPKTIWKEIEDQEKRIEKML | Phosphoenolpyruvate carboxykinase [GTP] [Crassostrea gigas] | EKC27095.1/0.0 | PEPCK(PF00821) |
| **Unigene34402** | 2.6312 | VILQTLEGHLR | 1 | AKGLHLFTKAEKDTSMGNIQTVGPNQAMVISGGCCGGTNRKLIVGSWGWAWCLVTDVQVISLEVMTLNPVCESVETSEGVPVTVTGVAQVKIMRDQSFLEKACEQFLGKSVREVERVILQTLEGHLRAILGTLSVEAIYQDRDQFAQLVREVASPDVCKMGIEIMSFTIKDIFDNVEYLASLGRAQTAAVKRDADIGVAEANRDAGIREAECDKTKMDTKFSADAKIADSSRNYQMQKADFDQEVNARKAEAELAYQLQAAKEKQKIRAEEIEIEVVERRKMIDVEEKEILRKEKELIATVKRPAEANAFKMELIAEGQRTQTVEQARAEAEKIRLVGAAEAEAIEAVGKAEAERMRLKASAYKQYGEAAMLSLVLETLPKVAAEVSAPLSKTDEIVLVGDDRTTSEVSRLVSQLPPAVQALTGVDLSKVLSKVPGAST | flotillin-2a-like isoform X2 [Crassostrea virginica] | XP_022341488.1/0.0 | PHB(SM000244);Flot(PF15975) |
| **Unigene3465** | 3.8968 | GLDTLGNMGFHDYR | 1 | IDRISGVHHDGIREITDKINWEKLYEKLLNSDNKSFEEVLKDLSPDDLLVYEDTINPDDIEKVRTFLLSKLEKNKKYESQSKRGLDTLGSMGFHDFRKRGLDTLGGMGFHSFKKRLNGQNGYTDFGKRGLDTLGGMGFHNYKKKGLDTLGGMGFHNFKKSVSDENQAHIIKKRSLDTLGGMSYHDYKKRGLDTLGNMGFHDFKKRGLDSLGGMGFHDFKKRGLDTHGGMGFHNYKRGLDTLGGMGFHDYKRGLDTLGSMGFHDFKRGLDTLGGMGFHNYKRGLDTLGNMGFHDFKKRGIDTLGGMGFHDAKRGLDTLGGMGFLDYKRGLDTLGNMGFHDYRKRGIDTLGGMGFFDYKKRGLDTLGGMGFHDYKRGLDTLGSMGFHDFKRGLDTLGGMGFHDSKKRGFDTLGSMAFPIYKRQLDINMADEYPSDEEIADILDDLNDQLSLADDNTILNSQSNTGRLKRNVNMAEEPPKQKSRR | feeding circuit activating peptides-like [Crassostrea virginica] | XP_022335398.1/2e-59 | Internal repeat 1 |
| **Unigene34815** | 2.8554 | YVYGSTNTQDALR | 1 | GVVSFSDSARQEFYLNTYNNADDINKAIRGIRYVYGSTNTQDALRMVREYQFTQSNGDRPGVPNVVVMVTDGESNIEHTRTLPEAMMLKDTGATLITIAVGFTSDSAELRGLTSEPVQSNLIKVDDYDSLDVLKDKLVTPLCTDANLCSPNPCKNNAECVDGLRSYRCICKEGNYGANCEKECTGEADVVFMLDSSSNIGEQTFDRMKRYSETLVREMNVEECGVRVGFMKYSSRPMVQFNMNRYHDTNTITRAVDSIHYTRGQANMADALKEVRTRMFNSADDRRDVRNVIFLMSDGSADIKKDETMMEAEMTISSGINIIPIGIQLRRREELDNIALVQGVNVEEIKDEKDVMAMSDQVLKPVKQVSDFCSGNPCQHGGSCHSDALGYRCDCVPGFTGDNCSKRCKASGDIVFAVDTSRYVTRKDLRQVKKFLKSLVKRMSFRNRMMRAGIVQFGKTADVKLTFRESVKKRNVINAVSSIRRSDGNPDPAEAFRKARISILEAGDRVDVPNYVILITHSMRQESDVIHEANKLKQKGTKVFGVGIGLSASDKEFMMAAVSNPENTYMYNTESVSGLADISDQIMAYLCNDQDYCAAAPCQNGGYCVNKQDGYYCECKDGYAGQNCEKACDAKADVAFLLDSSGSIGQQNFRLVKDFVHRVVQEMAIGKGHTRVGVASYSTNARMGFHLDDYLTKESVQDAISSIGYEYGNTNTAAGIKMVRRSIFNPARGDRSDAQNYLVIITDGVSNVNAENTIPEAKRAKEDGAHVYTIGVGSFDPTELKAMASEPVDKNSFMIDDFKALSSLTTDLIKATCRDPSACADNPCLNGGVCSIGVASFVCACPNGYSGERCEKACVDRKDIAFVLDSSSSVGKGNFDYMLDFVRALVEEIGSTSNEHKFALITYSTEVHLIFSFGRYRNNGEVGKAIATTRYTAGSTNTAGGLRTACEVFNGGEYGGRRAAEDVVILLTDGQSNVNSHDTIPAAEALKQKGIKVITVGINIQDTAEIKAIASSDNDVFLAESFKSLQDIKQDISDNSCKAKGS | uncharacterized protein LOC110464458 isoform X10 [Mizuhopecten yessoensis] | XP_021375363.1/0.0 | VWA(SM000327);EGF(SM000181) |
| **Unigene34826** | 2.2848 | LAGLLTR | 1 | EKMAAPMQRSVNVLQLLAYACAREQAVLNRCILSSHVIRNRSHSSKPPDEITGIRQDNKIVNPQSNSKPVNKAQKISRAMRAYLEKAKKYESVLEEQTVLYELGRRHLANIMGQDPTNFSVDDMTKAVQYLLPSGLHEKKARPVIKTPIDLLPMQKVARFGSDGRPFSPMFYTGKPQFYETLYDITLRLQKLKEKEDELARAGKFNDIYANKMRFIDSDWVKFDKFKKIIKEEALSEDNYAEFQKNMQRLIDHPLNHLEESYIMMFRDKFTSSGTKRDVYEIHELEDGTKYVIAQGGRKTSKAEVKVTVPGTGKFTCNGESILYFPKIADREQVLFPLELTGMLGEVDIEAMVENGGHTGQAGAVRLGISRGLVNFVDDEIVERMRLAGLLTRDPRMRERKKPFKKGARKSFTWKKR | sodium/potassium-transporting ATPase subunit alpha-like [Mizuhopecten yessoensis] | XP_021367153.1/0.0 | BBOX(SM000336) |
| **Unigene35728** | 3.8968 | IHHHSSDVMR | 1 | LYRAFERSGMPSEKDLTYLLPSAVRKMSDYNSQRWLKRIHHHSSDVMRRNSYDCKAKFVDIMSRWPLFGSTFFVIKNPPTTPAIKGECLLAVNKNGIQFLKLQSHESILQYSFSEVLSTRQYRSENNQHYLDMKLGNLMVQKIVRIETDQGSDISNLIGQYMQVIARNRKRPLTDRSTLDRTSLQRYH | Myosin-XV [Crassostrea gigas] | EKC41639.1/6e-81 | B41(SM000295) |
| **Unigene35748** | 2.8554 | VNNVGLWQSCDDR | 1 | MFIVSVYFKVAFALICVGLVFELIGFASPYWYSTRVNNVGLWQSCDDRIYHGDQCLSYRATTAVKAARAFSAIALILFVVLVILLLLYWCQCPRSDLILASIIICFITAACVFIGIICWVTRFSSNLSWAFYLCVIAGILAVVAGVLLIPERRIIIIGRTTTTGGTAVRTVTTTRTVTVR | lens fiber membrane intrinsic protein-like [Mizuhopecten yessoensis] | XP_021368751.1/9e-33 | — |
| **Unigene3720** | 2.0466 | DAVTYTEHAK | 1 | YNQYNMSGRGKGGKGLGKGGAKRHRKVLRDNIQGITKPAIRRLARRGGVKRISGLIYEETRGVLKVFLENVIRDAVTYTEHAKRKTVTAMDVVYALKRQGRTLYGFGG | core histone H2A/H2B/H3/H4 [Ancylostoma ceylanicum] | EPB74136.1/4e-61 | H4(SM000417) |
| **Unigene3849** | 2.8554 | TFLGSVSDYVVQHAR | 1 | LHSLSKFVSHIILFFVKVHIKMAEGSSRIVMIAVDASKHSDEAFDWYLNNMARDSDDVRVVHCAEYNIDLGFGLNYDEKQIQQITQQVKEQQEKVDQLKSSFVDKLRGKGPGDESFRIKGEAFVITGKKPGEAIIAEAEKQSANLIIMGTRGLGKIRRTFLGSVSDYVVQHARCPVVVVRH | universal stress protein PHOS32-like [Crassostrea virginica] | XP_022298339.1/2e-42 | Usp(PF00582) |
| **Unigene38699** | 3.8968 | IQNAGTEVVEAK | 1 | MFSRLAKPSCIVHVARRSFSLTSQAQQGPKVTVCGASGGIGQPLSLLLKNSPKVASLSLYDIAHTPGVAADLSHIETRAKVSGHLGPESLEACLTGSDVVLIPAGVPRKPGMTRDDLFNTNAGIVRDLVEACGNFCPKAMICIITNPVNSTVPIAAEVLKKKGVYDPRRLFGVTTLDIVRANTFIAEAKGLDVSKVNVPVIGGHSGVTIVPIISQATPSVSFPSEERKKISVRIQNAGTEVVEAKAGAGSATLSMAFAAARFTSSLLEALDGGEGQVECAYVQSEETDAPFFSTPILLGKNGVEKNLGRGKLIDYEMQLLEEAMPELKANIQKGVDFVSK | malate dehydrogenase, mitochondrial-like [Mizuhopecten yessoensis] | XP_021347898.1/2e-175 | Ldh_1_N(PF00056);Ldh_1_C(PF02866) |
| **Unigene40538** | 2.8554 | QEQPVGLFGLGLGGLGGGR | 1 | MKTFVVVALVLCVMVAHVRSQGHSLDLFGMGMGNNMGNPGGGIGGSVSASTGAGAANSGSDAMFMKMMKLILLKLNKLEKQQRPPKQEQPVGLFGLGLGGLGGGRRGNSLHHHLLLEHLSK | — | — | Signal peptide(1-20) |
| **Unigene41989** | 3.8968 | QFNGMVDVYR | 1 | MSKQLSFVENFALSGAAAVISKTAAAPIERIKLLVQNQDEMLKTGRLSEPYKGVIDCTMRTYKTEGVLPFWRGNLANCIRYFPTQALNFAFKDKVKAMFKSSKSDSYGLKFGKNIASGGAAGAMSLCFVYSLDYCRTRLANDAKSGKKGGERQFNGMVDVYRKTIASDGIAGLYRGFVISCVGIVVYRGFYFGLFDTLRPILLGESANVLLSFALGYVVTISAGLLSYPIDTIRRRMMMTSGEAVKYKGSIDCTLQIVKNEGFMSLMKGAGANILRGVAGAGVLAGFDKFQEYYIKWRVGANK | ADP,ATP carrier protein 3, mitochondrial-like [Crassostrea virginica] | XP_022315856.1/0.0 | Mito_carr(PF00153) |
| **Unigene42863** | 3.8968 | ASANAAAFLQWIPAVGWR | 1 | MRQLVLVALLGFVTNTYAGCSFKASANAAAFLQWIPAVGWRSFSCAPGLFFNPGLCCCAPFGVGASAAASASAAAGAGGSAAAAAAAAAAAAAAAGLGVGAGAGALAGAGALAGAGALAGAGALA | — | — | Signal peptide(1-18);SCOP(d1gkub1) |
| **Unigene44001** | 2.6312 | IEDAQVPSR | 1 | GKLNEMKRRTDDLEISLLGKLKEMKKRIEDAQVPSRDTKEMERKTEDFKRSLLDQLKEMRKRIEDAQFPSHEDVQEMGSKIDDLENTLLGKLNEMKKRIEDPRVRHRGCAKRWKHLLGNCYFFSKDRRTWSDSQTVCEGMSSNLVKIESQEENEAIVSTIKEIGIKDRDYWIGLNDIAKENSFVWVSDNTPVGFNKWHRKEPNNLLWRQDCGQLYNKYDWEWDDVECDSKKLFICKIRPE | perlucin-like [Lingula anatina] | XP_013402508.1/3e-25 | Internal repeat 1;CLECT(SM000034) |
| **Unigene44023** | 2.6312 | VLITTDLLAR | 1 | RYFSENMSYNPEDGDRSRNTNEGPEQKSNEEYSAEAGGVIQSNWDEVVDNFDDMRLKEELLRGIYAYGFEKPSAIQQRAIIPCIKGRDVIAQAQSGTGKTATFSIAILQQLDISQKDCQALVLAPTRELAMQIQKVVIALGDYMGAQCHACIGGTNVREDMHKLSTGVHIVVGTPGRVHDMINRRALNPRAIKLFVLDEADEMLSRGFKDQIYDVFRYMPEDIQVILLSATMPNEVLDVTQKFMRDPIRILVKKEELTLEGIRQFYIQVEREEWKLDTLCDLYETLTITQAVIFCNTRRKVDWLTDKMLSRDFTVSAMHGDMDQKERDVIMREFRTGSSRVLITTDLLARGIDVQQVSLVINYDLPANRENYIHRIGRGGRFGRKGVAINFVTQEDIRTLKDIEQFYNTQIEEMPMDVADLI | — | — | Cation_ATPase_N(SM000831);E1-E2_ATPase(PF00122);HAD(PF12710);Cation_ATPase_C(PF00689) |
| **Unigene44068** | 2.8554 | MNVLADALR | 1 | MVRMNVLADALRSIAAAERRGKRQVLIRPSSKVIIKFLTVMMKHGYIGEFEIVDDHRNGKIVVNLTGRLNKCGVISPRFDIALRDMEKWTTNLLPSRQFGFLVLTTSGGIMDHEEARRKHLGGKILGFFF | 40S ribosomal protein S15Aa [Mizuhopecten yessoensis] | XP_021374413.1/1e-85 | Ribosomal_S8(PF00410) |
| **Unigene44069** | 3.8968 | ESLPLIVFLR | 1 | MGYRGRRKHLKRLTAPKSWMLDKLGGVFAPRPSSGPHKLRESLPLIVFLRNRLKYALTHDEVKKIVMQRLIKVDGKVRTDMGFPAGFMDVVTIDKTSENFRLLYDVKGRFAVHRIKPEEAKYKLCRVKKLGVALKGVPYVITHDGRTIRYPDPLVKVNDSIMVDIATGKIKDFIKFDSGNLCMITGGHNLGRVGVIQHRERHPGSFDIVHIKDSLGHTFATRLSYVFVIGKGNKPYVSLPRGKGVKLSIAEERDRRIAARS | 40S ribosomal protein S4-like [Crassostrea virginica] | XP_022290789.1/4e-12 | RS4NT(PF08071);S4(SM000363);KOW(PF00467);40S_S4_C(PF16121) |
| **Unigene44556** | 2.2344 | ISQPSYIPTEQDVLR | 1 | MGCAASSDDKAASERSKQIDKTLRMDGEKAAREVKLLLLGAGESGKSTIVKQMKIIHEKGYSQEECLTYKPVVYSNTIQSMMAIIRAMGQLKIDFGHPDRADDARQLFALMGNADEGELSPELAAIMKRLWKDSGVQGCFSRSREYQLNDSAEYYLNSLDRISQPSYIPTEQDVLRTRVKTTGIVETHFTFKDLHFKMFDVGGQRSERKKWIHCFEGVTAIIFIVAMSEYDLTLVEDQEMNRMMESMKLFDSICNNKWFTDTSIILFLNKKDLFEEKIKKSPLTICFPEFAGENNYEQAAAYIQLQFENLNKRKDTKEIYTHFTCATDTNNVQFVFDAVTDVIIKNNLKDCGLF | guanine nucleotide-binding protein G(i) subunit alpha isoform X2 [Crassostrea virginica] | XP_022300181.1/0.0 | G_alpha(SM000275) |
| **Unigene44594** | 2.2848 | DPQIFVGR | 1 | MTEWVKTSANNIPNGSIRGGYDKNGHTLFIARALTDDGFYSAGKASLHYEDGAHIPYRGQEIIVYEYEILVLPSQADGFYDWKPTASANVPSNAVPSDINRDPQIFVGRFVHEGCLIPGKVDKKKMKCYIAHNGKEYPNDHYEVLVKVK | DM9-domain containing protein 2 [Crassostrea gigas] | AVN66933.1/3e-31 | DM9(SM000696) |
| **Unigene46265** | 2.6312 | ILNNLSSLDER | 1 | MEEVSCRPCQRLKQSTLVPSAYWCIQCSEMLCVACFRYHKVLKITKDHSFLTIDEYKPLANILSMIPNTCSSHEQTLKFLCVDHDVPCCQLCKTEDHPECTFSIAGYNDNLEKQIIQKESDKIKTLIDTLDKANDKILNNLSSLDERREAFHSIIKQKTETLLTTIEDLKIKSNYFEEEYQKRKEEIGKQQKSFIVKRKNLEVQQETLNAMQKLKSAPVSYSLALKQLCLFIEKEENFLQTLVKSLCTVKCTFDIRIQMKNSAFEVMDKISFTTLSIDLDERTEENSSSTVENENVMISQSVPSNNLMTPAEQPEAAEIKASGGKHVHQRELLLKRTYYFRVEKQNTNVFIRCLNILPMGHIIFGEQSKPRLMVYEKKGMRKCELLLKSYPEAIAVDNINNLIYVSSGQFVLKIDAINYNSLVIINQFPMNDICYGVGLSFGAIKVNCINDGLKTVMPNGKIVSVDRELNGKFKCKEKGKELVLIRNGSSIIEFNCSKKLQSMSVIGECGFDTDNEGNIIISSPDSNRIYAYDTDKTAWQVQLDIQPYKPMGVALDTQTKELVVIVDGGKSIWTYSKQF | 28S ribosomal protein S9, mitochondrial-like [Limulus polyphemus] | XP_013781006.1/3e-97 | BBOX(SM000336) |
| **Unigene49324** | 3.8968 | FAPINVENTEENR | 1 | MPVFPQYLSKEQEDELRQIANAIVAPGKGILAADESTGSIGKRFAPINVENTEENRRRYRELLFTCDKSLAENISGVIMFHETFYQKAKDGTPFPKLLQSVGIIPGIKVDKGVVPLAGTDNECTTQGLDGLSERCAQYKKDGAQFAKWRCVLKIQQYTPSYQAMLENANVLARYASICQQNGLVPIVEPEVLPDGEHDLATAQKVTEEVLAFTYKALADHHVFLEGTLLKPNMVTAGMSCAKRNSPAENALATVTALSRAVPPAVPGVTFLSGGQSEDDATVNLNAINTCPGKKPWALTFSFGRALQASVLKAWQGKDENVKGAQDELMKRAKANGLAAVGKFSGGLAGTAGSDSLFVAQHAY | Fructose-bisphosphate aldolase [Crassostrea gigas] | EKC30386.1/0.0 | Glycolytic(PF00274) |
| **Unigene49344** | 2.0466 | LLEVPRK | 1 | MPTSRSDVLSPLTTPAIQKRSGLSNTASSVGHFVSPSTSRHKGMDSPVTMYMEEHDGATEKRDRRRSRALELKQTVGSPASPGERRGSFSSSSSHGLTANQLADHYSNCIKLSAENKINSKNAFGLHLIDYMSDLIKKKELENFQVASTTLDASAKIYAGRVDAIHAETYKVLSGLGSSKDKNKQNEEEEEDGTEPMDEDGTPDNEDKIKKKKKKKNTTIETNLKNITTAKVDLAFEVDPMFQLMSEAFDEGGTMGLLHNTLRCYNDNQELVLDSSTVVGGNCCEPPQYKPIDISDLKDMFKGVDIKAKEICPSFSSFTFMDWDENAEDPSNVSQDNEDHAFDINAEPEPIPEGDNDNDDMPDMAGDFPENEYAESDTEDNQGSGETIGDGKAAEMINTMESIKHGTTGTLLSVLASEPSDYSYFNSTLLRAWAGPSHWKIKPLSKDSRIRSLAEPGASKKKLRKEPFEVNYNQQIDFDSFFKPSKSTMLTKATMIKHSKKKKSLPKDLHYDADKLFRLFGKSKIMIKRQKNTNDNVDVDAEIDNYNFNNSNDMENYCPRGDEACDDDDDDENGDFGFNFTADVGSQETNDSQPSVLVNDTALDGTLLGDKLLEVPRKVAKIDIGYAKTAKKLDVKKLKRTIWGILTESEEDKDKPGASGGQTDILNKTMDKTIYFTELLSELPKRVSGQTQENLSVPIAFVCLLHLANEKNLRIEDSEMKDLIISQDVLH | — | — | Cnd2(PF05786) |
| **Unigene5067** | 2.1364 | YDWDFFDR | 1 | MSGRLVPVHRYDWDFFDRQMSLFPSFKDDFDKDFFSDFKSTKIEDEIARMKREMFQLTSPEQSLKVDQPFVEDFTGNKKMALRFDCSKFKPEEIEVKTVDRSLTVHAKHEEKSPGRSVYREFTKSYTLPRDIDPLSLKSSLTNDGFLQVEAPAPKTCIARKEIFIPIEKMLK | small heat shock protein 22 [Mytilus galloprovincialis] | AEP02967.1/8e-80 | HSP20(PF00011) |
| **Unigene51624** | 3.8968 | SSQSSLAASLFDLTTTIAK | 1 | MNKLFLSLLVFSVLLAFAHSKPAKKRSNALKSSQSSLAASLFDLTTTIAKIGSSRSKKRSSKKSRRGKKKSSSRKSKSKKSKKSKKSKKSSKKSKKRRS | — | — | Signal peptide(1-20) |
| **Unigene54892** | 3.8968 | LMDFFTQPESHFQNSR | 1 | QQQIISPNYLTDTQLLQNAFPNIGETNGLLQQSTAFQEQQPLFGASPSLKADPLQHLVSSPGIGNFQQDSRLMDFFTQPESHFQNSRHIKTILPRSQQAPQMLQTVPVGEKPLTPLNTRCPPDSPRVKCWHDPCKVTTCPLFREAICVKYQCGECQAKFTIDGVDITHYCHV | — | — | — |
| **Unigene55006** | 2.2848 | IEFLEDTK | 1 | MNSLGTATLFLVVIWISVSEQGILPSRKNQVRSRRNYPERKPENIEKVLASGKENSGGGPASSGGQGNKHNVVIDQPPSNNGGLSNGNFGGHGAVVDDPTPNLGGPGNHDVSVADDPTPISGGGSPDKGNNNPESPKPEGGSPNGQGNNYDVSGPGDTAPNGLGNIHDAPVPDGNNNIAQPKDVGPQNGPGNNQDSPAASGGADPNGHGNNQDSPATSGGADPNGHGNNQDSPAATGGAETNGQGNNHDATIPASSGGGVQVDDHNTHIVGNAGGPVDANDHPLPRRTSRRRKKGRKGDKNENKEKRIEFLEDTKEMRDGAKATEDNEEDSEPQPVKPRKLRPKPTPAEKVINILTHNQPLPSSDPSTNQPSQQSESAASNPSPASGLVDNYPSPVSSHVNNNPSPSKPKEKARLFLFGDWSDCDSSDCWSSESSDSEKGSSSEKSTKVDSNIIESKLKFLRSRPKALIKMAAKDIDDISDKSSDCNSSDESDCEYWYSSEESVANDNGRRSSSKKNWNKKGSSESFPRGRKYGSRESYEKNSDGRDYSSDSSGWYDYESYEDDWSSEGRRNKHGDNRKTKTNGDSNNNDNNNDNNHENGNNNENNHDNGNAKNNHGDNGKGKRVERKRKLNFQDWDSYEYSDYSDSYESSDYKWHSKDSAEKTEKRNKEKKSKKGWYYDSSEYYSDSYDYESDDSGYYSDSSEWYENDDIFHKK | — | — | Signal peptide(1-19);Internal repeat 1 |
| **Unigene55260** | 3.8968 | WDSDYIDGVSPLK | 1 | AKPWFFGQFEDGILETCLWCVRKGFNDNITKAMADPVRVSRIISKIVNSFDDNGILTGRWDSDYIDGVSPLKWSGSVRILRQYARTGKAVKYGQCFVFS | — | — | — |
| **Unigene55909** | 2.8554 | LSHYLTSHGR | 1 | MSNSHLAFLLFCIVPHNTVISSRPNQDRTPATAHNSRLSHYLTSHGRIRPYQWLNDDNNLDIPGDEHLQGDGTHYHGENMLGDGVQQHVMDAEDPEIQRHICCSLGRAQAVGSQNCNVRHYIRIARESLRVQESSHDTEQFSSTNSGSGKAYRCILRHRHSFTKCCHTARISNHDHHARRLHRHHSS | biotin/lipoyl-binding protein [Paenibacillus gorillae] | WP_042163681.1/1.3 | Signal peptide(1-21) |
| **Unigene56883** | 2.3583 | AGLQFPVGR | 1 | GKDSGKTKTKAISRSQRAGLQFPVGRIHRHLKSRTTSHGRVGATAAVYSAAILEYLTAEVLELAGNASKDLKVKRITPRHLQLAIRGDEELDSLIKATIAGGGVIPHIHKSLIGKKGQQKTV | unnamed protein product [Oncorhynchus mykiss] | CDQ62413.1/9e-81 | H2A(SM000414) |
| **Unigene6047** | 2.8554 | FLEAAYGYR | 1 | MYRNYITQLDQKKGGQQQGKMAEETQAQLWDKLNKGESKSLLKKHLTPELYEQLKDKKTSLGGTLGDCIRSGANNLDSGVGLYACDPEAYTTFKPLFDAVIKDYHKVDGVNHPKPDFGDVSKLEDLDQYGGDMIVSTRVRVGRSHDGYSFPPCLTKESRKEMMDKTAEACDKLTGDLKGKMYRLESMSKEENQQLIDDHFLFKNDDRFLEAAYGYRDWPNNRGIFHNPSKTFLVWANEEDHLRFISMQKGGNLKEVYGRLVEAIKQLEAKLTFAKKDGYGYLTFCPTNLGTTCRASVHIKIPKLSKLPEFKQICEKHNLQPRGIHGEHTESVGGVFDISNKRRLGLTEFEAIMEMQNGVKEIIKMEKSL | arginine kinase-like protein-1 [Mytilus coruscus] | AKS48144.1/0.0 | ATP-gua_PtransN(PF02807);ATP-gua_Ptrans(PF00217) |
| **Unigene6218** | 3.8968 | VAEQQPSPVR | 1 | EVKTTEKTGKVYYQIISKGSIVQTGEIIMGDATVMKAPIMITTEMAPKARFIVNYVRADGEIVTDGVTFSVDGTFKNNVAIRFSKDEALPGSRMLVDLEADAGSQVNVLAVDKSVLLLKSGNDISEDKITEELQSYDNAGGGGFYPVFARMWYWPSTGRDASDVFDNNGMVVLTDAMLYNYEDPFRPMWRRGGAIAFAGAPMPMMAVQNSPPEMAEMARPDTLKAVTKIRKIFPETWLWVNASVMDTGKTTLNVKVPDTITSWVATAFAVNQKSGLGLTPQPANLNVFMPFFVQLVLPYSVVRGELVVLQVNIHNYLPRNEWVLVILEQNMGMHNVITYSNGMRKLAHAKVGRWIKLQGGSIGSAFFPIKPQEVGQLKISVSAKTGSASDAVEKHLLVEPSGTPQEYNVPVLIDLKSVGTFQKKVNITFPKQTSPGSRRVAASVIGDLMGPSINGLDKLIKMPYGCGEQNMLNFAPNIFVRRYLSITNNLKSDMDAKSKEYMVKGYQRELTYQHKDGSFSAFGESDKSGTTWLTSFVVKSFAQAKQYIFIDDELVLKALEWLVKQQNETIGTFDEPGKVLHKAMQGGSAAGERSLTAFVLIAMKEADVIQGASDVTSRSVQKATNFLEGEVDKLIDTYEMAIVSYALKLVNSIKGDIILERLNALATVEDGLKYWEHDVQESKEQKFVSWNPPHSQSNAIDIELTSYILMNYALNKNINNGLPILRWLTTQRNPDGGFSSTQDTIIALQALAEFAGEIYSSDFNMRLTLQSSKGEPFMDSYTITPENALVLKTFDIPVGVEELTVIANGKGVALAEVAVYFHTDSDIKTSSFDINTTLSEETTNGFKLEVCGRYLRKGATGMSLMEIGIPSGMTPDYETLDFKKAPEYKRKEELFRKLVLYFDSFDKEPQCVSLYIIRTDRVAEQQPSPVRIYDYYEPSNQKTNFYTSSKLSDSSLCDVCGKECFCTN | CD109 antigen-like isoform X1 [Crassostrea virginica] | XP_022323515.1/0.0 | A2M_N_2(SM001359);A2M(SM001360);Thiol-ester_cl(PF10569);A2M_comp(PF07678);A2M_recep(SM001361) |
| **Unigene6274** | 3.8968 | ELTDQLSEGGR;IEELEEELEAER;NLYSTHPHFVR;SYSAELFR | 1 | LQQFFNHHMFVLEQEEYKKEGIQWEFINFGMDLQACIDLIEKPMGILSILEEECMFPKASDKSFKEKLFTTHMGKSPNFNKPGKASKGKKSDFELTHYAGIVPYGTEGWLEKNKDPINETVVDLLSKSKEHLVQTLFAPPAPVEGGGSKKKKSSAFQTISAVHRESLNKLMKNLYSTHPHFVRCIIPNELKQPGLIDAFLVLNQLQCNGVLEGIRICRKGFPSRIVYSEFKQRYSILAPNAIPQGFVDGKVVTDKVLTALQLDPAEYRLGNTKVFFKAGVVGNLENMRDERLSAIISMMQAHIRAYLIRKSYKKLCDQRIGLSVIQRNIRKWLVLKNWQWWKLYSKVKPLLNIARQEEEMQKKLEQLKKLEEDLAKCEKIKKELEVQNVTLLEQKNDLFLQLQTEQDNVIDLEQRVEQLVKQKADFESQIKELEERLLDEEDAASELENIKKKMEGENDELKKDIEDLESSLAKAEQEKTTKDNQIKTLQDEMAQQDEMIAKLNKDKKGMDEAHKKTLEDLQKEEDKVNHLNKVKQKLEQTLDELEDGLEREKKVRSDVEKAKRKVEQDLKATQETVEDLERVKRDLEEANRKKDAEINSLNSRLEDESSLVAQLQRKIKELNARIEELEEELEAERAARTKVEKQRAEISRELDDLSDRLDEAGGATQAQLDLNKKREQELVKMRRDMEETILQHEAQVSTLRKKQADAANEMADQIDQLQKVRNKLEKEKKDMKREMDDMQATFQHQLKNRGASDKVVKQFESQIADLNAELEKSQRNLSDMVNNKTKFEREAAELSQQLEEAEHNVGSFSKEKSRLAQQLEEARSALEDETRVRQKLQSEIRNLTGDLDAAREQVEEEQEGRSDLQRQLNKANTEAQTWRSKYETEGAARAEELEDSKRKLQAKLAEAEQNADAANAKVSQLEKAKNRLQGELEDLAIETERATANANAMEKKQRGFDKTVAEWKSKVNDLQLELEAAQKEARSYSAELFRVKAQVEESQDSVEALRRENKNLAEEIRELTDQLSEGGRSVHEVEKAKRRLEMEKEELQAALEEAESTLEQEEAKVVRAQLEISTIRNDIDRRLHEKDEEFENTRRNHQRALDSMNASLEAEAKGKAEAMRIKKKLEQDINELEVALDASNRAKAELE | myosin heavy chain [Mytilus galloprovincialis] | CAB64662.1/0.0 | MYSc(SM000242);IQ(SM000015);Internal repeat 1;Myosin_tail_1(PF01576) |
| **Unigene635** | 2.1364 | VNLFVR | 1 | ICTASSAAGIDRDYVDLKVQGVLPDGNGPIKIDTQTVNIGERVEMECVVTGEPRPTVSWSRVGEPIPDTATVNDVFLVIPQVRIEDAGTYVCTAQNLGGIVQQRVNLFVRARPIISGSQA | heparan sulfate proteoglycan-like protein-1 [Mytilus coruscus] | AKS48136.1/3e-57 | IGc2(SM000408) |
| **Unigene7385** | 2.8554 | IPAINVNDSVTK | 1 | MSTKPPFKVADINLADWGRKCIEIAENEMPGLMQMRKMYGETKPLKGARVAGCLHMTTQTAVLIETLTALGAQVQWSSCNIFSTQDFAAAAIAKTGVPVYAWKGETDEEYIWCIEQTLVFPDGQPLNMILDDGGDLTNLVHERFPQYLPGIVGLSEETTTGVHNLHKMMKDGKLKIPAINVNDSVTKSKFDNLYGCRESLVDGIKRATDVMLAGKVAMVAGYGDVGKGCAHALRAFGARVMVVEIDPIIALQAAMEGFEVTTVEECLPKCRLFVTATGCSSIIHDKMFEQMLEDSIVCNIGHFDCELDVKWLNENCAKKEQIKPQVDRYTLKNGRHVILLAEGRLVNLGCAHGHPSFVMSNSFTNQVLAQIELWTKKEEYKNKISVTVLPKKLDEAVAAAHLDHLGVKLTKLTEEQSSYLGIPRDGPFKPEIYRY | S-adenosylhomocysteine hydrolase [Crassostrea ariakensis] | ACT35639.1/0.0 | AdoHcyase_NAD(SM000997) |
| **Unigene7730** | 2.1737 | NCDDPNNDEDDDCKR | 1 | AGAGAGAGAGAGAGAGAGAGAGAGAGAGAGSSAAAQAISLLLSSGNDDVRRAAAFALAAASSGGGGAGAGAGAGAGAGGGAGGGAGGGAGGGAGGAGGAGGAGGSGGSGGSGGSGGSGGSGGWGWSNNKNCDDPNNDEDDDCKRLRGY | — | — | SCOP(d1b3ua_) |
| **Unigene8830** | 2.1364 | TTDGYLLR | 1 | MAVGKNKRLTKGGKKGGKKKIIDPFTKKDWYDVKAPCMFVVRQIGKTLVTRTQGTKIASDGLKGRVFEISLADLQNDEVSFRKFKLMAEEVQGRNVLTNFHGMDLTRDKLCSMVKKWQTLIEANVDVRTTDGYLLRMFCIGFTKKKNMQVKKTCYAQHTQVKAIRKKMVDIITKEVSSNDMKEIVNKLIPDSIGKDIEKACQGIYPLHDVYIRKVKVLKKPKFDLGRLMELHGEGGASKTVVTESGETVERPEGYEPPVLESV | ribosomal protein S3a [Mytilus trossulus] | ALX27208.1/5e-178 | Ribosomal_S3Ae(PF01015) |
| **Unigene9746** | 3.8968 | AYGPGLSEGVCNQPAR | 1 | PTKTSKIRAYGPGLSEGVCNQPARFTVETNGEVGALGFSIEGPSEAKIDCQDNGDGSADVTYYPTSPGEYAVHILCNDEDIPESPYMAQIAPATNAFDASKVIAEGPGLQKTGVTTNKYAEFTVDTRKAGKAPLKITCEDDQHKPVNVEIVDKKNGTFACKYMPKKQCKHTVTITYGGVQIPKSPFKVNVGEVSNPGNVKVYGPGVEKGVKTFKTTYFIVDCKSAGPGDIAIALVDAQGKDVPVNTIDQKDGTFKIEYTPNSPGTYIVSVYFANQEIPKSPIKVNVESSIDLSKVKVVGLDTPIKIGEKRDISVITKGCGKADGPVKVTMITPSKKKVNIPVKESLETWKG | filamin-like protein-3 [Mytilus coruscus] | AKS48150.1/0.0 | IG_FLMN(SM000557) |
