# Supplementary material for: Microstructure and in-depth proteomic analysis of Perna viridis shell
Source: PLoS One. 2019 Jul 19;14(7):e0219699. doi: 10.1371/journal.pone.0219699 (PMC6641155; doi:10.1371/journal.pone.0219699)
Supplement: S3 Table — (DOCX) [file pone.0219699.s008.docx]

**S3 Table**

| **matched unigene** | **score** | **matched peptides** | **number of matched peptides** | **protein sequence** | **Homologous protein [species]** | **Homologous ID / E-value** | **Domain and signal peptide(SP)** |
| --- | --- | --- | --- | --- | --- | --- | --- |
| **Unigene10181** | 143.9521 | ANLEQEVYR;AQLEAERDNLAAALR;CDELGAENAQLR;DLDSDVSTSTR;DLENELEADQR;DLENELEADQRR;DTEEALR;DTEEALRDAEAK;EKDEEIDSIR;ELEDALDSER;ELEGALDNANR;ELELQLEETQR;ENGQLQAALR;EVVQQADDDRR;GSSPGTQNR;GSSPGTQNRLEGR;HQEALNDLTDQLEHMGK;IAIQQELEDAR;IAIQQELEDARSLLEHAER;IDALEGSNGR;INVDDLTR;IQELEDNCEQLR;IRDLENELEADQR;IRELEDALDSER;IVRHTYNVYR;KAQSMIDEAEQR;KNAENELGEVSAR;LAAAQAALNQLR;LEEAEAFAQR;LEEAIGSSTTFSEVSR;LQAEVNR;LTQENFDLQHQVQELDGANAGLAK;MEADIAAMQSDLDDALNAQR;MIEEAEDVANITMNK;NAENELGEVSAR;NQLSISER;NRELELQLEETQR;QINELTALK;QLDDARNQLSISER;QLENDNAALQK;QNLQVQLAALQSDYDNLNAR;QVAELTSLTDQLTMK;RMEADIAAMQSDLDDALNAQR;SLLEHAER;TLVEVETR;YEEESEAASNLR | 46 | MSLLRDLDSDVSTSTRIVRHTYNVYRGSSPGTQNRLEGRIRELEDALDSERELRLRYEKQSAELTFQLDQLSDRLEEAIGSSTTFSEVSRKREAEVSKVRKDLELASAQFEATEANMRRRHQEALNDLTDQLEHMGKAKARAEKEKNQLIIEIDSLQGINDGLQKAKMSADSKIDALEGSNGRLKINVDDLTRQLNDANSAKARLTQENFDLQHQVQELDGANAGLAKAKAQLQILCDDLKRNLDDESRQRQNLQVQLAALQSDYDNLNARYEEESEAASNLRAQLSKVNADYAALKTKYDKELIAKQEELEEIKRRLSVRIQELEDNCEQLRTRCNSLEKTKNKLTAEIREITIELENTQIIVQDLTKRNRQLENDNAALQKRCDELGAENAQLRNDKANLEQEVYRLKVANAELAEKNGNLERENGQLQAALREAQNELKSANRQINELTALKAQLEAERDNLAAALRDTEEALRDAEAKLAAAQAALNQLRAEMEQRLREKDEEIDSIRKSSARAIDELQRTLVEVETRYKTEITRIKKKYETDIRELEGALDNANRANAEYLKQIKSLQNRNRELELQLEETQRQLDDARNQLSISERKRIAIQQELEDARSLLEHAERARKNAENELGEVSARLTEVQLQVTALTNDKRRMEADIAAMQSDLDDALNAQRAAEERADRLQAEVNRLADELRQEQENYKNAESLRKQLEIEIREITVRLEEAEAFAQREGKRQIAKLQARIRDLENELEADQRRLREAAASARKFERQWKEVVQQADDDRRQVAELTSLTDQLTMKCKTYKRMIEEAEDVANITMNKYRKAQSMIDEAEQRADMAEKNLTAVRRSRSMSVSREVTRVVRV | paramyosin-like isoform X3 [Crassostrea virginica] | XP_022322570.1/0.0 | Myosin_tail_1(PF01576) |
| **Unigene2367** | 52.6687 | AIGSASGLLASK;ASLLLQSAR;CLIPGTLHYR;DISNGGLAFDHSHIYLR;GGSTSASGSVSAGGSVGSSR;GLGASSGFSSAGAAASASGLGSASLGSTR;GLGSVSVTTGGLGQDLR;GLNGIGGSVTSSLTSK;HGSISGSGISSLDSR;LLTALTK;LSFDNDER;MYLWGFQSK;SGSSSGSSGSSGSSGSGGSSGSSSFGSSTSGSDSR;SRPEPIISNCGPNGDSSVEIVVYR;TGGNLEIR;YGYLAPEHGGLR | 16 | GAGAGAGSGVGLGFGVGAGAGAGSGTGLGVGAGAGAGAGSGIGQGIGIGAAGAGSGIGSGAGAGIGVGSGIGFGAGAGAGAGAGSGIGIGVGAGAGAGAGAGLGIGSGIGAGAGAGAGAGSGVEIGMGAGIGAGAGSGFGQGIGATSGLGSSSSGSWGSAGSGESSCSCASQGPKSETIQASAKLAVVGDRNVLIAKMPRAKVVDSDGLGDFLLEFPNLTIQPTLTEKIASPAELFGMNMNKGTHSTDDSDILVVALLPKGSKLGSKMFQPKSSGSGSSSGSSSSSGTGSSSGSSSSSASTNDNPSVSVDTSQDDGKLKIKIKAENVKDSTASIVKNQLDKIKSDEISVDTSKDDGKLKIKIKAKVDDLSKSDINALSSESSYSGSSSGSGSSGGSGSAGGSGSAGGLGSAGGSGSASGSGSAGGSSAAGGWGSNGGLGFSGDLGSAGSSTSAGGSGAASGSTGGSGSVGISGSISVSGSTGGSGINVGLGSAGALGTAGLSGSVGGSKLTVGLGNARGGSTSASGSVSAGGSVGSSRGSGSARAIGSASGLLASKIKASLLLQSARGLGASSGFSSAGAAASASGLGSASLGSTRGLGSVSVTTGGLGQDLRSGSSSGSSGSSGSSGSGGSSGSSSFGSSTSGSDSRLKIEKSKDDGKIKYKIKTVKGKGDSKTKSEYKLVVDKSDGSSSKVKVKFGKSDQDRSSSKIKHGSISGSGISSLDSRGKSYRREEDGDKWKLKSSGSRGLNGIGGSVTSSLTSKKQQIAEAVKVLKALKASQKASNALTAGVSLKAKLPDTRYKLPEQGGTLNILSQKLTKDIKFKQSRDNDERKKLSYKLSFDNDERKSRNRLLTALTKTSTNTVVPKTTGGAKVPNLEGLCLIYLGAFVDAIGYAPLPGQCHKLVQCFYLGGKLKAVARDCPAGMFWDQNTLSCRPPGDVLCFEDKCLIPGTLHYRRNGGCNCFYKCRDGISEPSCCPKGFRYDDDKGCVPAFGSLACDDECETPTTLTTQVTVSSCPSLPDPNNKYGYLAPEHGGLRIRACPAGTIYSASQCQCKSNMNGSGAMRGSLRKQYRQCSAEFNINFDDGFKDISNGGLAFDHSHIYLRKGKAVFGGNSRMYLWGFQSKYLGKTFAIKTRVKVNKGAGRSRPEPIISNCGPNGDSSVEIVVYRGKLIFKAKTSDNPDTVYFNEKYDDDKWMDITYYYDGNYFGGSCNGRSFKQRTGGNLEIRDNPMTIGLCSGKNPGFHGEIDELEIYTACIPKGF | matrix protein-1 [Mytilus coruscus] | AKS48137.1 /0.0 | ChtBD2(SM000494);Laminin_G_3 (PF13385) |
| **CL1886.Contig2** | 47.2944 | ASVGGGSGGTVYTR;DIQNEYDNKVDQIR;EVNLDGWK;EYESMQSEHTMETVK;FLEAQNR;GSAQEAGINDLVFR;IDLNNETLNHLDAENR;IDLNNETLNHLDAENRR;KLIDDLTR;LADLEAR;LNQQLSDYESEINMLR;NAQLEAQYNSLLR;NVIEQSMNTQSR;QTLEEEMEFLK;SSTVVNR | 15 | MSKSSERITEKRTVITSSSSNYDDGDDSIYYKSGIQPRSSTVVNRSSIGPSMRASVGGGSGGTVYTRTVEYGMGRSSGLGNLSPGSYEKVSNTGVMTVKSSREKEKKDMQDLNERFANYIEKVRFLEAQNRKLAGELEHLKTKWGKETSAIKSMYEQELAEARKLIDDLTRDKNKLEIQNSSLQEEMNGLRRQMDDLKKYHALDQEQINKLNQQLSDYESEINMLRRTISSLETERARDKDRINKLQGEVDRLRIDLNNETLNHLDAENRRQTLEEEMEFLKKVHEQELKELAALAYRDTTEENREFWKSELSQAIRDIQNEYDNKVDQIRGDMESYYNLKVQEFRTGATKQNMEVTHVKEENKKLVKSISDLKGRLADLEARNAQLEAQYNSLLREYESMQSEHTMETVKLKEEITNLRAEMEAILVELQSLMDAKLSLELEIAAYRKLLESEESRVGMRNVIEQSMNTQSRGAAQLSEMITEYETKGDSHSSMKMMRGEVSAKTTYQKTSTGPVSIAEVNPEGKFITLENTSSQRREVNLDGWKIRRELDGQREVVYTFRNFTLKPHKSVKIFARGSAQEAGINDLVFRDEETWGVGSQVSTCLVNEKGEEKATHRQRTAYN | filament-like protein-2 [Mytilus coruscus] | AKS48133.1 /0.0 | Filament(SM001391);LTD(PF00932) |
| **Unigene39912** | 37.7745 | GGPVIALETLADAPSEPR;ICDSTQTIASITDLSPHSR;INLVEPDYPYIVVVR;LADMEQATGELVR;LEWTSDFLDDDTTHVFR;QLQIMIIR;SLPIEGATQR;TEYISPTR;VGADVFETVITK;VGLGDAAITTYR;WELVEVFESNK | 11 | MLSIGLILVSLVSVSHGQWRQDMFSQAENKLRNIVQNGQILLDFIYQERQKHGGGNMTSGSMMSQNLAYSSFINDVEVRLADMEQATGELVRIMRTCPDAPLAPPSPTNVIVESTTTDNVSSIVVKWDPPFNPPENMQYKVYFVPVDINGMQTAGEVVFRICDSTQTIASITDLSPHSRYRIRVGAVAGSIAESTSVPLNVKTPDLIPSRVQNVMVKSSTPNTITLMWNPPSTMGDLVSYEIYYEENPINKMHVTVSPPENTFTIKDLSEGTTYKFEVSAKSDNGEGIRSLPIEGATQRFIPRAPQSFTGVALNKTAVKVTWTPPPPQPGDGIIRGYLINYTDVRYTDVSEHRVGADVFETVITKLTPAQVYYFRAFAFTKKSVGRGGPVIALETLADAPSEPRQLQIMIIREEPPKIGLTWLPPLHTYGNLLNYTLIWGVQNGANRTEYISPTRLEWTSDFLDDDTTHVFRLAAVNKVGLGDAAITTYRTPKKVPIIPPNVKVKRVTFENNGTTILNVTWDNPVVAVDGFRILYRKFQLVYSGRWELVEVFESNKKFVRINLVEPDYPYIVVVRGIPKGQIFNQYNMGGGQHMSRPNSHIAQSFGGASPPI | shell protein-6 [Mytilus coruscus] | AKI87977.1 /0.0 | Signal peptide(1-17)/FN3(SM000060) |
| **Unigene34815** | 28.4017 | AVDSIHYTR;EMNVEECGVR;ISILEAGDR;NVINAVSSIR;SDGNPDPAEAFR;VDVPNYVILITHSMR;YHDTNTITR;YSETLVR;YTAGSTNTAGGLR;YVYGSTNTQDALR | 10 | GVVSFSDSARQEFYLNTYNNADDINKAIRGIRYVYGSTNTQDALRMVREYQFTQSNGDRPGVPNVVVMVTDGESNIEHTRTLPEAMMLKDTGATLITIAVGFTSDSAELRGLTSEPVQSNLIKVDDYDSLDVLKDKLVTPLCTDANLCSPNPCKNNAECVDGLRSYRCICKEGNYGANCEKECTGEADVVFMLDSSSNIGEQTFDRMKRYSETLVREMNVEECGVRVGFMKYSSRPMVQFNMNRYHDTNTITRAVDSIHYTRGQANMADALKEVRTRMFNSADDRRDVRNVIFLMSDGSADIKKDETMMEAEMTISSGINIIPIGIQLRRREELDNIALVQGVNVEEIKDEKDVMAMSDQVLKPVKQVSDFCSGNPCQHGGSCHSDALGYRCDCVPGFTGDNCSKRCKASGDIVFAVDTSRYVTRKDLRQVKKFLKSLVKRMSFRNRMMRAGIVQFGKTADVKLTFRESVKKRNVINAVSSIRRSDGNPDPAEAFRKARISILEAGDRVDVPNYVILITHSMRQESDVIHEANKLKQKGTKVFGVGIGLSASDKEFMMAAVSNPENTYMYNTESVSGLADISDQIMAYLCNDQDYCAAAPCQNGGYCVNKQDGYYCECKDGYAGQNCEKACDAKADVAFLLDSSGSIGQQNFRLVKDFVHRVVQEMAIGKGHTRVGVASYSTNARMGFHLDDYLTKESVQDAISSIGYEYGNTNTAAGIKMVRRSIFNPARGDRSDAQNYLVIITDGVSNVNAENTIPEAKRAKEDGAHVYTIGVGSFDPTELKAMASEPVDKNSFMIDDFKALSSLTTDLIKATCRDPSACADNPCLNGGVCSIGVASFVCACPNGYSGERCEKACVDRKDIAFVLDSSSSVGKGNFDYMLDFVRALVEEIGSTSNEHKFALITYSTEVHLIFSFGRYRNNGEVGKAIATTRYTAGSTNTAGGLRTACEVFNGGEYGGRRAAEDVVILLTDGQSNVNSHDTIPAAEALKQKGIKVITVGINIQDTAEIKAIASSDNDVFLAESFKSLQDIKQDISDNSCKAKGS | Collagen alpha-3(VI) chain [Crassostrea gigas] | EKC21865.1/0.0 | VWA(SM000327);EGF(SM000181) |
| **Unigene6274** | 27.0882 | ATQETVEDLER;DMEETILQHEAQVSTLR;ELTDQLSEGGR;EQVEEEQEGRSDLQR;IEELEEELEAER;IGLSVIQR;LEDESSLVAQLQR;LQGELEDLAIETER;LSAIISMMQAHIR;NLTGDLDAAR;SYSAELFR | 9 | LQQFFNHHMFVLEQEEYKKEGIQWEFINFGMDLQACIDLIEKPMGILSILEEECMFPKASDKSFKEKLFTTHMGKSPNFNKPGKASKGKKSDFELTHYAGIVPYGTEGWLEKNKDPINETVVDLLSKSKEHLVQTLFAPPAPVEGGGSKKKKSSAFQTISAVHRESLNKLMKNLYSTHPHFVRCIIPNELKQPGLIDAFLVLNQLQCNGVLEGIRICRKGFPSRIVYSEFKQRYSILAPNAIPQGFVDGKVVTDKVLTALQLDPAEYRLGNTKVFFKAGVVGNLENMRDERLSAIISMMQAHIRAYLIRKSYKKLCDQRIGLSVIQRNIRKWLVLKNWQWWKLYSKVKPLLNIARQEEEMQKKLEQLKKLEEDLAKCEKIKKELEVQNVTLLEQKNDLFLQLQTEQDNVIDLEQRVEQLVKQKADFESQIKELEERLLDEEDAASELENIKKKMEGENDELKKDIEDLESSLAKAEQEKTTKDNQIKTLQDEMAQQDEMIAKLNKDKKGMDEAHKKTLEDLQKEEDKVNHLNKVKQKLEQTLDELEDGLEREKKVRSDVEKAKRKVEQDLKATQETVEDLERVKRDLEEANRKKDAEINSLNSRLEDESSLVAQLQRKIKELNARIEELEEELEAERAARTKVEKQRAEISRELDDLSDRLDEAGGATQAQLDLNKKREQELVKMRRDMEETILQHEAQVSTLRKKQADAANEMADQIDQLQKVRNKLEKEKKDMKREMDDMQATFQHQLKNRGASDKVVKQFESQIADLNAELEKSQRNLSDMVNNKTKFEREAAELSQQLEEAEHNVGSFSKEKSRLAQQLEEARSALEDETRVRQKLQSEIRNLTGDLDAAREQVEEEQEGRSDLQRQLNKANTEAQTWRSKYETEGAARAEELEDSKRKLQAKLAEAEQNADAANAKVSQLEKAKNRLQGELEDLAIETERATANANAMEKKQRGFDKTVAEWKSKVNDLQLELEAAQKEARSYSAELFRVKAQVEESQDSVEALRRENKNLAEEIRELTDQLSEGGRSVHEVEKAKRRLEMEKEELQAALEEAESTLEQEEAKVVRAQLEISTIRNDIDRRLHEKDEEFENTRRNHQRALDSMNASLEAEAKGKAEAMRIKKKLEQDINELEVALDASNRAKAELE | myosin heavy chain [Mytilus galloprovincialis] | CAB64662.1 /0.0 | MYSc(SM000242);IQ(SM000015);Internal repeat;Myosin_tail_1 (PF01576) |
| **Unigene51075** | 28.8189 | LASDLLEWIR;LENSFNTLQTR;LMEEYER;NINEVENQILTR;QGLEEAER;VEQIAAIAQELNVLHYHDVQSVNTR;VGWEQLLTAIAR;YNEEVQESMIFENR;YTHYTMETLR | 9 | MEDYPSDGYMDEEEEWDREGLLDPAWEKQQKKTFTAWCNSHLRKAGTQIEDIEEDFRNGLKLMLLLEVISGEQLPRPDRGKMRFHKIANVNKALDYIASKGVRLVSIGAEEIVDGNCKMTLGMIWTIILRFAIQDITVEELTAKEGLLLWCQRKTAPYKNVNVQNFHLSWKDGLAFCALIHRHRPELIDYYKLSRENPLENLNTAFNVAEQHLDIPRMLDPEDMVNSAKPDERSVMAYVSSYYHAFSGAQQAETAANRICKVLKVNQENERLMEEYERLASDLLEWIRKTTPWLENRTTDNTLPGTQRKLEEFRDYRRKHKPPKLEDKARLENSFNTLQTRLRLSNRPAYLPTEGKMVSDIANAWKGLELAEKGFEEWLLSELQRLERLDHLAQKFRHRCEIHEEWAEGKEDMLQSQDYLKCRLNELKAMKKKHEAFESDLAAHQDRVEQIAAIAQELNVLHYHDVQSVNTRCQLICDQWDRLGTLTAQRRQGLEEAERILEKIDQLYLDFAKRAAPFNNWLDGAKEDLLDMFIVHSIEEIQDLIEAHEQFKGTLGEADKEYNSIMGLANEVQRLAQQYGLTLKENPYTTVSPQEDIANKWGEVKQLVPKRDRTLHDEKIKQENNERLRRQFAQKSNVVGPWIENQLDGVASIGVTARTSLEEQLNKLRQFEKATESYRVHMDELERYNEEVQESMIFENRYTHYTMETLRVGWEQLLTAIARNINEVENQILTRDSKGISEDQMNEFRVSFNHFDKNRTRRLEPKEFKACLVSLGYNIRDDRQGDADFQRIMSIVDPNNSGYVTFEAFLDFMTRETADTDTAEQVMQSFKILAGDKPFITAQILRQELPPDQAEYCIQRMAPYSGRDAVPGALDYMSFSTALYGESDL | Alpha-actinin, sarcomeric [Crassostrea gigas] | EKC43084.1 /0.0 | CH(SM000033);SPEC(SM000150);EFh(SM000054);efhand_Ca_insen (SM001184) |
| **CL955.Contig7** | 26.0114 | CNMISGEVEELR;DLEESTLQHEAQISSLR;DTTDLALVAPGLK;ELEDLGER;ELEGELDSEQR;ELEGELDSEQRR;ESYNLAER;GQLEISNVR;IEELEEELEAER;LDEAGGATSAQVELNK;LEEAESQALK;LQGELEDLGIDVER;NSLQSIIDELR;QNVEEETR;RDLEESTLQHEAQISSLR;SSVSISR;SYSAELFR;VRELEGELDSEQR;YQQQVSEVQR | 8 | MSDLKIQVSTSVTSSKTEKPAVVKEEKAPEERIKTPDIISSPPAPEEAKVTTVKEETRRASASTLTPKMSDTSRITKTTRTSSIRTADYESTVGQLTKDYRGTSPAVLEGIASQPVLYSKAFDKIGQQKLSARSRKILRDTTDLALVAPGLKNLLEARIEELEEELEAERAARTKVDKQRAELARELEDLGERLDEAGGATSAQVELNKKREQELLKLRRDLEESTLQHEAQISSLRKKQQDATNELADQVDQLQKAKAKVEKERQQFRSECDDLQAQLQHYSKNKGVSEKMAKQLENQIAELQQKCDEANRNVNDLNSQKAKMQAENSNVVAQLEDTEHQIGSLSKERNSLQSIIDELRQNVEEETRARMKLQSDIRNLNADLDAAKEQIEEEQEGKADLQRQLSKANNEAQQWRSKYENEGANKAEELEEAKRKLQAKLQEAEQNAEAANAKVSSLEKAKNRLQGELEDLGIDVERANANANALEKKQRAFDKTIQEWQAKVSDLQSELENAQKEARSYSAELFRCKAQYEESQDSVDALRRENKNLADEIHELTEQLSEGGRNVHEVEKARRRLEMEKEELQAALEEAESALEQEEAKVMRGQLEISNVRSEIERRLAEKEEEFENTRRNHQRALDSMQASLEAEAKGKAEAMRIKKKLEQDINELEIALDASNRAKAELEKNIKRYQQQVSEVQRQVEEEQRQKEEVRESYNLAERRCNMISGEVEELRTALEQAERARKGAENELFEANDRVNELSAEVQSISSQKRKLDGDIQAMQSDLDEMNNEVRNADDRARRAQEDSARLADEIRNEQEHSQQIEKFRKSLEGQVKDLQVRLEEAESQALKGGKKMIAKLEQRVRELEGELDSEQRRHAETQKNMRKADRRLKEIAFQADEDRKNQESLNSMIDTLNAKLKTYKRQVEEAEEIAAINLAKYRKVQQELEDAEERADSAEGSLQKLRAKNRSSVSISRSSVTHTPSRTLLSTERSDI | catchin protein [Mytilus galloprovincialis] | CAB64664.1 /0.0 | Myosin_tail_1(PF01576) |
| **CL185.Contig3** | 28.9939 | AESPLPDDVLIER;APPAFQQPPPR;HPPPPHQPNQPPPPR;QPAPQQHR;QPQYQQPQQPPVAR;QQHPQPLPPQQPPR;QQQPQYPQQQPAYR;QQYQSSQQPYQGQR | 8 | PEVNLWNGSEMKLINVWTKVWTVFGLWNIAPTLGQGPMAMGGIGGASPSRGGKMTQKFINTILKLHNDYRRTEGASNMKKLRWSRALQRDAQLWANKCRYTHAYGKWGENLFKAESPLPDDVLIERAVNEWYYEKMSWKFTPDCNEACHYTQVVWAESEEIGCAYKRCTTLMLMEEFVMNGWMLVCYYNTQGNIIGKMPYSVGKACSACKPGYKCDRGLCDKQRTVHKYLPQHNQQKPKPVFRAPPAFQQPPPRHAPPPRHAPPPRHGPPSRHPPPPHQPNQPPPPRQQHPQPLPPQQPPRFRVSAPQPPPRPNSSLRPQYPPQHGQPAPYRPQQLANQPARPPSGSPPTNRPKPEPKPQPKPQEPQMQYISGKVLSGWIPAQTSKATPAPTNATFQQPQVQYQHQVPLKQQPYQHSNQAQPQRQQYQSSQQPYQGQRQQQPQYPQQQPAYRQPAPQQHRQPQYQQPQQPPVARQQPNQPQHQQHARPQSTRHSPYQQPYQQPHPQYNAQPAPAQQPQYPPAQPHNPYPDQPSHQPAPYVDPRPIYVPPAPAAPSSPTTTIPPPPPPTCLDGDKHCKHWGVHCKTNPYVHTNCRLTCNTCDIPTPPVHAKPIQTTQKSKAEPTIIPSRPPSPSPGRDHFHQPKPQHRPQPKPRRPPPQPQYNHYATTTAAYYPTTTGYNNQAPTTQTPYNAPGQGTSAAASSVASVGPHVGFCRDFDNRCKEWAKYCGMDEYVDDMCRLTCMRCKK | DNA N6-methyl adenine demethylase-like isoform X4 [Crassostrea virginica] | XP_022319021.1 /1e-44 | SCP(SM000198);ShKT(SM000254); |
| **CL1011.Contig5** | 25.1754 | GAPGPAGPR;GPIGPEGPSGQGLPGPQGPPGR;GPMGPEGPQGPPGLAGPR;GQPGPQGSPGLR;GQVGIPGPR;GSPGLGGTSTR;MGAVGAPGR;YPPYGNSYGPSAYQQR | 8 | GPPGKDGRPGMRGLTGKPGNAGIQGPIGPPGPQGPPGKDGRPGMRGLTGKPGNAGIQGPIGPPGPQGPPGKDGKVGLQGAPGVAGKPGTNGVPGMRGLTGRPGKQAIPIKAAAIPGPPGPIGPQGLIGPAGPKGANGRPGMRGLTGRPGAHGNDGKAGPPGKDGLPGKPGPPGAAGRPGMRGLTGRPGAGKDGPQGPPGKDGAPGRPGMRGLTGRPGAGKEGPPGKDGAPGKPGQRGIRGWRGWRGFKGAPGPAGPRGPMGPEGPQGPPGLAGPRGSPGLGGTSTRQTVVRGPPGPPGPQGTRGPIGPEGPSGQGLPGPQGPPGRGQPGPQGSPGLRGATGSPGLQGKPGIPGKPGAPGMQGLPGPIGNPGPPGAGVKGMGKEQVAVQGPPGPPGPPGAAGRPGKDGHPGVVGPMGPRGHPGEPGMMGPHGPPGKDGKPGPKGDSGAEGPPGQVGPMGPAGPQGKAMQATHHDIVQGLPGPPGPPGERGQVGIPGPRGYRGHPGAKGDYGARGPPGRMGAVGAPGRMGQPGPPGRQGLDGAVGPIGKPGPKGECSCGGPPMGAMRPPMGAGMGPRMGPGPYRPHMPYMAPKTPHTGPPPGYGWSQQRYPPYGNSYGPSAYQQRSNSLIGGTGKPSGSWRKKPVDTEAEMK | collagen alpha-1(II) chain isoform X2 [Otolemur garnettii] | XP_003793620.1 /2e-35 | Pfam(PF01391) |
| **Unigene51088** | 24.7016 | QSLNNSPMFNSR;QTSLQLSSAR;SPLLSQDLATIR;SSFPQSR;SSFSQSSLPMMQSR;SSLSLLDR;YIEIVHGNVHER | 7 | MYQEGIILIFFIFISHSNGQGCPHSATPVPGDPTRYIEIVHGNVHERNCAPGTEYNTFTCDCSVFSGASIQALMDGSAGSHPDLLNMPLETLQKEFLQLTSQRAPQSTRTDSRRTSKRRKSKTAEFQYNPKKKESQESILFGLLADSALLNLLPTNPPSKAVKHVEKPQFKSEFIDYSLLAPTKAPKIKTFSALNLPKVTPKPKLKSPLLSQDLATIRAFLKERREQRAREKALLKTKQSPITSSASTSSIGLSGTKNAFSFDSGLKILGSSTTKQSTASNLGLNDLRTSAAKSTSSLDIGFNDYHKAALASNTGGLNTNNVKSSVGIAVPTPDAGLNEFRSSLSLLDRNYPTPTPHSRDTFANVKTVVNPTDNFAALMERRKLEQALITKSEPSSSISGISNTASKSSNGLGGLDATKKAILKNLVARLNALEEKNKGTATSSQQQNSMMSQTSSNSDQMQGQSTSLSQRQSLNNSPMFNSRQTSLQLSSARSSFPQSRSSFSQSSLPMMQSRQTPFSMMFRGVAAKEGSSDAQEIMQDRITEMGGAAALAMGAPLSMINSEISSQLSSTMLENGPLPFLM | Matrilin-2 [Crassostrea gigas] | EKC40227.1 /1.2 | Signal peptide(1-19)/SCOP d1dqca_ |
| **Unigene48535** | 20.1318 | FTQLER;GLNSGGVNSGTR;MGGVNSGTR;QINLSNLNAEQLEVIMDQR;RNGMNLPLCQPLYECAENFIPPQCR;TEFFIPR;YDTVNSMMCR | 7 | SLICILLLVKFALIAGQRQLPRTEFFIPRTQQNAKRVQQKFTQLERLKQLNNMSGMKFGNSGASGNGWNNNVGANGGRGLNSGGVNSGTRMGGVNSGTRMGGNRRQINLSNLNAEQLEVIMDQRRMQMEMGMGMGMGMSGGGMGAGNGGYTFINVEALNNAAEMGIMPSMFMGNNGARGQQGMLGNNNNNGKGGMNVGRGNGSRRNGMNLPLCQPLYECAENFIPPQCRTWRYDTVNSMMCRSCPINTCQGGAKEMFNTMYEHHPKRLLFEALTGK | — | — | Signal peptide(1-16)/Gly(16.3%);Asn(12.7%) |
| **Unigene40545** | 18.7605 | EVGVHLVNVYR;FNDEHIPDSPFR;GEINQPCDFNIYTR;LANGHLGISFTPR;VNVSPSIGDAK;VVAPSGVEEEAIVQEIDDGQYAVR | 6 | ATYYPKDEGKAKVDVKYAGQNVPGSPFPVEVFPGVDASKVLVSGPGVGKNVYASMPATFTIDTRNAGNAPLDVVVQRPDGSFIKPLVQDNGDGTYTVQYVPDDLGTYVLRVKFAGKEVPNSPFKVTSHPTGDASKCVITEGLENKTVQVNKETVICVDASQAGDGKVTCRIRSPQGSDIDIDIVENADGTFSLLFTPQIEGAYTISIKFGGQTVPGGEYDIQTTGYTAVNEDLISADTVDSVKGAAPGSGLFQPVDFCIPVGPIFNFVSAYIVMPSGKKAYPKIEDNKDGTVTIRYQPTETGLHELHVNYNNEEIEGSPFKFHVDAVNSGHVTAYGPGLSHGIVNEPAYFTIVTKDAGAGGLSLSIEGPSKTEIKCNDNGDGTCTVSYIPTAPGEYNITVKFAGQHISGSPFTSKITSPPGEIKRKSQFGRSSEFELKVVEEDINNLMATIRTPSGVEEPCLLKRLANGHLGISFTPREVGVHLVNVYRNGHHIQNSPFQITVGESELGNASKVKVYGPGLEQGNANELNEFTVDTKDAGYGGLSLSIEGPSKADIECQDNEDGACRVTYKPTEPGNYIVNVKFADEHVPGSPFNVKVAGEPSPKLTERITRHREAADVMHIGSQCELSLKIPASFFMKMMTHEQIEAILVQQQEGTSPFDMTASVTNPSGVTELCDIVSLDDNHYSIKFVPKEMGVHTVSVKHKDMHIPGSPFEFTVGPIAGGGSHKVHAAGPGLERGEINQPCDFNIYTREAGAGGLSIAVEGPSKAELDFDDRKDGSCGVTYKVAEPGEYLVSIKFNDEHIPDSPFRVNVSPSIGDAKKLSVSALQSKGLQIGKPAAFVVNFNGAQKGKLKARVVAPSGVEEEAIVQEIDDGQYAVRFIPRENGGHNVHVFFNDCEIPESPFRIMVGKVDCDPGMVHASGDGLRTGQSGQPAKFFVNTVNAGPGALGVTVEGPSKVKLECTEKEEGYEFTYYPTAPGDYLITIRYAGVHIAGSPFKARIEGQAGPSDVIQHGMSQVVVETVTKTSVMSKFQAIPQFKSDASRVTCEGNGLKKAFRGKQATFNVDTSNAGNNMMFVGMMGPKGPCEELCVHHKGGYQYKINYVVKERGDYMLIVKWGEEQIPGSPFCVHVE | filamin-like protein-1 [Mytilus coruscus] | AKS48135.1 /0.0 | IG_FLMN(SM000557) |
| **Unigene3051** | 19.0439 | AIQNTQNGMWAAGR;CQMIIGIPALPDLR;FTPLHTLLTR;QMPTWPR;QTGNFFVVDTVPGR;STTLAPTGYPCLHK | 6 | SIIPPDTKYQDIVVDNLSYSGRPGSGSVLIEPVPVSSSFGTLPDGVVIADSGSPAAGALPDGVIIEPVPEGSNLGGIQIPTGGNVPVLIKDDNLLIKPSPEKTVWTSEPILINDSNLIATTPSKEEYGQNLSGSGKGSTSAIKTTTSTSSVTIQSQEPSAVVTVNNGTKSNGDNEGTTWLVTPTLPPTTKAPVINPNSGTPKPLSCPDFDSRYENGKFIVTLDEGKCEMVVSERAIQNTQNGMWAAGRFTPLHTLLTRKYKPEINFRSTTLAPTGYPCLHKTRQTGNFFVVDTVPGRCQMIIGIPALPDLRNLLTQRQQKVPTWNKPRQMPTWPRRRPSKEREDNESSD | mucin-3A-like [Mizuhopecten yessoensis] | XP_021361490.1 /0.002 | Pro(10.6%);Thr(10.3%) |
| **Unigene20161** | 18.8134 | AAHAYGVPNTSLFQTVELYEAR;GMTGFGAIR;GMTGFGAVR;GMTSFGSQR;NLPMVLATISHVGTEAQR;YDPAVESEVR | 6 | MADRVKPMGMDRALISKMGAKYDPAVESEVRGWINQLIGEDIGEGPSNLEKGLRDGVILCNLMKKIIDGTPSESLPPACNKLNLTPSPSELPFKQMENIEKFLKAAHAYGVPNTSLFQTVELYEARNLPMVLATISHVGTEAQRHNYNGPTIGSKPTEKHRVQFTYEQLKNSHGTIGLQSGTNKFASQKGMRIGAIRHISDIRADDLDKEGTTLLTLQAGTNRFASQKGMTGFGAVRHIADIRADDADKAGDNIITLQAGTNKFASQRGMTGFGAIRHVSDIKADEFDPNTQSHIGLQAGSNQFASQKGMTGFGAVRHICDIRADDLDREAQAEIPLQYGTNKGASQRGMTSFGSQRHIADIKVSDLAEDMKRQDLDMTPKEYQQYRRELEEAAKQQGQEVEEPQYE | calponin-like protein-1 [Mytilus coruscus] | AKS48134.1 /0.0 | CH(SM000033);Calponin(PF00402) |
| **Unigene8686** | 16.1597 | ERSDGEYGSNGAGYR;LGLDDFGDDNGDDN;RLGLDDFGDDNGDDN;SDGEYGSNGAGYR;WDSDQDFR | 5 | SGTLYGSCHSKTLDMRKLAVVSILLFLGLPSNIICRNDVKEKDGNRGNRWDDMSDSWGFSEDSSDGGWGSNSVKWDSDSEGWDSDNFGSDRRSPRGRGGNGNNRLDSDSGWYTSEYFSDESDNRVGIRGDNGNKLRNIVGLESEGIDWDSDNSDSSNGAVKKAGNGNSGFGSNGIDRNSETSDSSDGVSKNAGNGNESNGRGRKGNINIGGHSGGRFDSDDSDWGSDRWDSDQDFRRGRSNGNKENNGGGDSNGGGRRWGDSDRRDSDSWDSDDNNIWDGSDGWERERSDGEYGSNGAGYRGSDDSDWFESDNRFDSAESFERLIKRLRRPNDGDSDGNSPPGVKFLGNNGVKRADFWDDSDDLWNDYAIGRRRHRPRRRRISAKRLGLDDFGDDNGDDN | — | — | Internal repeat |
| **Unigene57834** | 18.3436 | EISFIHDIVNLLDVGDRPTQSR;FAVIITDGSSR;TYLGLEYVHNTIFAPGNGER;VAAVSFSNR;VGMMTFGTNPR | 5 | MGYFALLVLVAVSVVECRNYHSTHVRCEKPVDLAFVIDISSSIWYKHFRREISFIHDIVNLLDVGDRPTQSRVAAVSFSNRLKPEFGLGQYSTKEGVLNAINNIAYEGGDATRTYLGLEYVHNTIFAPGNGERSNVANVVVVLTDGVTNPGSYDNFTRTEAKQKTQIHAQNIRDIVRAQIYAIGIGNEVDKNEIKGIANKPSEQFTLFVDTFTELDTDAVKKAVLTKVCDSLPQREQECSTSKADIFFVVDESSSLMWDANFRKELKFVGSVIDQFELGKDLVRVGMMTFGTNPRMLFYLNDFKTKTEIESLLKITPWHGGNTYLDKAIESLMTYGLNPGFGSRSDVPQIAVIITDGKSTHPTETEKQIAIMKRMNYVVFAIGVGPNKDPIELHKIASHPSNVFEVDNLDGLVAIRQQLLSQLCPGDQPKPPPVNNCQNSMADLIFVADSSTSIGLTAYNEFKTFAKSVVEKFTVGPKNIQIGLITFSNDAHYEFSLNEYRTKEEVTKAIERVPYSTGNTNTHKALEILIKQGFSYINGGRGTSVPRFAVIITDGSSRQPEMTKQLARKAKDQGIILFSIGVGPYITQTELDGMASSPTSMYSFKVDNYAALTRIEQSLVKRTCEEATRQRQFS | Collagen alpha-5(VI) chain [Mizuhopecten yessoensis] | OWF49639.1 /1e-152 | Signal peptide(1-17)/VWA(SM000327); |
| **Unigene37646** | 15.8036 | LTWACESPCR;LVCYDQCR;MDGEENEYEGPTSMSNR;RMDGEENEYEGPTSMSNR;VSFWYQCK | 5 | VSFKFLSFFLLLMTVTGQRRRPISKTDDRVSFWYQCKQECIRLTWACESPCRNYAETRMAYKMCALECKLDRLVCYDQCREFIAQGLVWLASPSNNNKRNRRRMDGEENEYEGPTSMSNRKDFMAFLNYILES | P,N-U7 [Pinctada fucata] | AKV63173.1/0.71 | Signal peptide(1-17)/Arg(10.5%) |
| **CL4276.Contig2** | 14.3016 | EDSYEETIR;KLAITEVDLER;LAITEVDLER;QIQEHEQEIQSLTR;TLQVQNDQASQR | 5 | MDAIKKKMVAMKMEKENALDRAEQLEQKLRETEEAKAKIEDDYNSLVKKNIQTENDYDNCNTQLQDVQAKYEKAEKQIQEHEQEIQSLTRKISLLEEDIMKAEERFTTASGKLEEASKAADESERNRKVLENLNSGNDERIDQLEKQLTEAKWIAEEADKKYEEAARKLAITEVDLERAEARLEAAEAKVIDLEEQLTVVGANIKTLQVQNDQASQREDSYEETIRDLTNRLKDAENRATEAERTVSKLQKEVDRLEDELLTEKEKYKAISDELDATFAELAGY | Tropomyosin [Saccostrea glomerata] | AVD53650.1/1e-139 | Tropomyosin(PF00261) |
| **Unigene58102** | 14.5475 | EPENPPMENALEPAR;EPLTVDPYGDALTSGRPEVAHPEFISSR;SVFINTDNMR;VRPSTAEFIEQGPLFEHDGTQR | 4 | MKETVLCIVFLQLALVFGAPKSTQTNKVVPKDRVLQGAVEQLFIPDGNSLFEIDIVQGYNPEVIQHVRRKRQAEEKSLERKSSSKKRRRKKKKRRRSEKKPGAVTTLREITPTEKIMLPPVEREPENPPMENALEPARKSRRTFRPPPPTDPAPTLPALVLSEMENNNDDNSGLLKVKSLKSVFINTDNMRTNKLMADRIKADAIHAKDVVVTSKKKKSGSRSGGRKRRRRVRPSTAEFIEQGPLFEHDGTQRVNSILEQPQYIPETILTKNPYGLGDTIYQPEKPRRRFRVNERHLYREPLTVDPYGDALTSGRPEVAHPEFISSRVRSKVPLLSEFIEGTSGNSKPSTTFVNRRRPFAKQRPIEEFHIFDNPINTERQNPVAKRVDKTSKRFTKNVDPWFL | KS-rich protein [Mytilus coruscus] | AKS48160.1/5e-14 | Signal peptide(1-18)/Arg(10.7%) |
| **Unigene483** | 13.3039 | AAAAAAASASASAGSGIGVASR;AALVQLVIK;FGGGFGGGAGAGAGAGAGAGAGAGAGGAGGASAAAAAAAAAAAAAR;LYAYDYYK | 4 | GFGGGFGGGAGAGAGAGAGAGAGAGAGAGASAAAAAAAAASARRAALVQLVIKARAAAQARAAAAAAASASASAGSGIGVASRFGGGFGGGAGAGAGAGAGAGAGAGAGGAGGASAAAAAAAAAAAAARNANLRGWQSANANSLAAAIAAASAGGGGGAGAGAGAGAGAGAGGGAGGGAGGGAGGGSGGSGGSGGSGGSGGSGGSGGSGGSGSAVRLYAYDYYKNSDDKKGPGYERS | — | — | SCOP(d1gkub1) |
| **Unigene43460** | 12.1614 | FLQAQVGPSGPR;GPQGDIGPSGER;GTMGEPGPTGPQGER;SVYGGAMTSWFR | 4 | MKFGTVRWKNCIAPLFLFIVLVKSQEEDKANCLYEGTTYYHGDQWKPENCKWCVCDNGVADCKELLDCDGFGQITTGQESNTAIQGHQAIENEAEGSVGSPGRDGSQGLPGPIGDPGVNGKHGIPGPPGPPGVPPMSADQAYNRYFQQTYGQSFKAGGPAMGPRFLQAQVGPSGPRGSPGLPGQPGPQGADGVRGESGDTGPPGNPGLRGAPGAPGPPGLEGDSGRNGETGPRGLSGPKGPTGPAGMPGMPGMKGHRGLQGVQGPSGEQGRPGDKGSSGAPGAPGPNGPEGPRGSQGDRGSDGSAGPAGLPGVDGLAGAAGEPGPVGRTGPPGSPGLPGQKGEAGASGPKGSQGLQGSRGDPGISGPPGAEGMAGSDGLPGSNGEKGASGDPGPAGSPGFQGPRGPSGLNGSPGNAGAKGAPGQPGSPGFKGERGPKGIRGSGGDRGPPGAPGNEGKRGQRGTMGEPGPTGPQGERGSTGMRGYPGPIGDPGAAGEEGGIGPRGRRGEPGPNGVPGRMGPPGARGPRGGNGGPGIDGMAGRPGPPGVTGNDGRPGEMGAPGIPGPAGIQGVQGNPGTRGPPGKDGNPGAQGPRGPQGDIGPSGERGNTGPRGAVGEPGGRGPEGNGGAPGFVGAPGPPGGQGEPGKPGEPGPPGKAGKAGRPGSRGERGIPGVTGEPGAPGLSGVQGPEGGAGRDGERGAPGEPGGLGEPGPEGPAGRQGMRGPRGERGAKGEMGEAGLPGEDGREGRKGPSGPQGPPGEPGPPGEPNEKGSVGDLGLPGERGARGTPGDRGPQGTAGIQGEPGQPGMAGAPGPKGQRGQTGQKGEQGAAGIAGAQGAPGPTGRDGMNGRKGVRGDRGSQGLPGQPGTPGGVGPVGNAGPHGDDGPPGPPGEDGIKGSRGETGHVGRPGESGAPGLPGEPGLKGARGEDGESGGVGQVGPAGPPGEMGVPGDSGIRGERGNAGPPGRPGQPGDPGRAGLNGAPGNAGPPGPPGLAGPSGEVGHPGPPGPDGAPGLQGAQGEKGPDGDIGMPGAMGLMGFSGPPGPPGPAGPAGERGERGEPGPNGAAGQQGARGPPGPQGPQGPSGEKGSLGENGDKGDPGLMGMPGLSGPEGPVGDLGPTGPQGPPGQRGPDGRRGDPGSDGMVGPSGPPGPPGPRGPQGEDGRRGSMGEAGNPGPPGAPGRSVYGGAMTSWFRGSSGNKGWQGDEPVPAEEIDTDVFKALEEVTLQIEKIKNPTGEQDSPGRTCEDLRAHNPDIKDGYYWINPNLGPIYDVIKVRCDFRKKRVFTCVQPEVKTIENMNIAQKNDHTWISEVLGSKFDYDPSLFVKPQIKFLQYLHQKANQEIVYKCKNSVAIDDDKSIQLAGFDNSLLSSKGKRSIRYKIKKDNCKNKNGSWEKTVLEVNTKRTKALPIMDIGVYDIGGADQDFKIELGEVCFFN | fibril-forming collagen alpha chain-like [Crassostrea virginica] | XP_022341069.1 /0.0 | Signal peptide(1-24)/VWC(SM000214);Collagen (PF01391);Internal repeat 2;COLFI(SM000038) |
| **Unigene40702** | 11.0548 | CETFGYSGR;LPSYPGYNPR;NPFFTINMYYFNAQTGR;SPVECLSR | 4 | WKLFCESNTELRMIRFLIILLVPVTAAFLFSSLNFNNVNQNLDLRKLLTYRQLPHRCRLPSYPGYNPRNPFFTINMYYFNAQTGRCETFGYSGRGGNRNRYRSPVECLSRCACHMPVDPGTCHNSTTGITRYYYNKVFKMCASFQFNGCEGNDNNFADFMSCQLACGRSGGGGEIEL | nacre protease inhibitor-like protein 1 [Mytilus galloprovincialis] | AKQ70858.1 /2e-47 | KU(SM000131) |
| **Unigene40680** | 11.4569 | AMEDEFGR;FPTCENIR;GYNSEVTER;MCFFNITSLTPEHEYQFR | 4 | ISSPPKINVPKRFQDVAIFEKGENVVLKIPFTGSPKPTITWLHDGEEVKGRGYNSEVTERHAILTIKDAGKDQDGPYRITAENSLGSDSAVIKIQINDRPDPPRFPTCENIRDDSVLLTWKPPLNDGGSFITQYVIEKCEPPNKNWIRVATSRMCFFNITSLTPEHEYQFRVIAENFYGSSDPCEPTPVIKTDIPDAIKKKRAMEDEFGRKVRGTGPKVDNYDKYYHDLWKKYVPQPVSVKTGNVSDYYDILEELGSGAFGVVHRCVEKSTGRVFVAKFINTPYPLDKYAVKNEINVMNQCHHPKLLQLKDAFEDKY | twitchin [Mytilus galloprovincialis] | BAC00784.1/0.0 | IGc2(SM000408);FN3(SM000060);Pkinase_Tyr(PF07714) |
| **Unigene31135** | 10.4751 | DPCFDFTVR;FYFEEIPGLGMMTR;GSQYNPGLCTCDR;VNDELTPSR | 4 | GPTADRVNDELTPSRQWPTREATDMKSPSSSLLQAENGKKEPSAGWQTQDTIDNNQDSSRQWQTDRTIDSNNGPQRQNPTRVSVDNRLYRHREEDRDTNKLLKISVEANLLNGKDIVGTKIEKFVGGDPWWTQNTNPNYEVQDPTYQQPSFNEQKWYKQSDPIKAPWVNIPVQKSTPAAWKTYAPSTPSYQVYTKPQTETQTTTSAWASEYRNPLISPVTLFSINGPVDNRRTQPVYPRRPIPRFLTTTSTTTTTTTTTTPRAPVVSEPQPFAVGPAQGKDRIEEEHLCYGSQYVDGVGYNADPKSCNEFIQCFYEGGQIKVERARCPFRMFWDQRLLLCRPSGSMSCWRDPCFDFTVRSYDHKGNCRAHWSCRNGLSQAKCCPDGFAFNEEKQECVPDPGCHDWCEDSGELTFTSPFASCRMTPFETNERFYFEEIPGLGMMTRSCPRGSQYNPGLCTCDRSEEVRKNSCRPELILDFNDGFSDKSGNNLAVTPENVHIENGAARFDGSGKIRLWRFSGVSYGSQFAVTMKYKENPHVQSAEPMHLLSNCFNPEVMRPSIDIAISPNKGAMFLATTDKGGEQMSVMKYNSTSWKTLSFVYDGRHLIGNIDGKKTTVPLTGTIEERPSALILGACDYFGGYIGMMDDVKLYLCVPHGIT | sushi-like protein [Mytilus coruscus] | AKS48157.1 /0.0 | ChtBD2(SM000494) |
| **CL3951.Contig2** | 10.8650 | FIGSLTQSFNNGR;GNDLEAAR;GTFTDGTR;SGPFLVFR | 4 | MKDIVYIAVLFFTLFHVANSLCSHPCSRVNRGTFTDGTRNFVFGCTNTSVLQVYEGNRFIEDRECYARSGPFLVFRVGSRYQCFKDTVVDPNTNVVMIYFAPTQTFSTNPSICDVCAGEYTFALFVPRGNDLEAARRLPRPPLGCNRPPNCPILPDPYYIPCTGCEPKEDDGLCCSSCQDINNVYNRYGRGNQDRRNRDRRGRSNRFIGSLTQSFNNGRYSRPTKRSAKTC | — | — | Signal peptide(1-20)/Arg(10.8%); |
| **CL1023.Contig1** | 12.2166 | FELPYSTIR;LSEISEPSNEVIR;QSYNIPLLLEAQSPNQGTVIR;STESADAVER;TPVTFPPNR | 4 | KNTKTFVVEDVVLPKFEVNVILPSIQLTTDSHFTATITAQYTFGKPVEGDVLLSIYSGSKRRGITKRFKINGKATIRVLMSEIAPRFRYFTVEAEVTEAVTGDKQGNAQRTHLYETQEKLLFSPTMPYTFKPGLDYNIILRATQENDKALTGYLGQVNVTVFYRVPKQKEEKTGALQCTEGMCPPSDETEEKVLWSKNIQIPESGLMKEVASFPINALSGQVQADYRMASDRKYLSKAQSPSSNYIQVTIVNDQKAKAGTNLPLMIKATEPVQYVNYKIFAKQYMMQQGTFDMQNTSSKQVQIAITTDMAPKVKLLVYYTRPSDGEIVAAAVKFPIEGIFDYEVTLRFNKERALPGDNVTLSVTADPNSLVSVLAVDKSVLLLRTGNDVTVKDVINELNNYDGNLYPNFGAWDYWFSRPISSIDASSVFKDMGVYVLTDSLLYKHSEVVRRSQSFNVPMNAEILAMDAPAMSFTSGGMDDLAKPSRTRKNFPETWLWTNTMTGASGISMINAKAPDTITEWVTSAFAVNPTSGLGVSSDIANLTIFQKFFMRFELPYSTIRGEIVIVQITLFNYLATAQNVQVKLNGGEGFSFVDANGDPLNTGSNGMTKTTLVKNDSVSSVYFPIKPTTVGKVTLSATARSTESADAVERELIVEAEGIKQSYNIPLLLEAQSPNQGTVIRTPVTFPPNRVPDSTFVKVQVIGDFLGLALANVENLLGSTSYGSGEQNMITFVPNVYISSYLKTTNRLTTEIKKKTERLMQGGYQRQLSFARIDGSFSAFGSNDPRGSTWLTAYVIKSFAQAAEFTYIDKKVITKAIKWLLTQQAHSGNFEENGIFIRKELQGGSTSSARSLTAFVLIALYEAKSNDQVEAEIKSNVDSAISKATQFVAEGAPASITNVYELAISFYALSLVKHASKNLLLVELEKKESIDGEEKFWRIPKTEADIIQPWKEWSPQNEEFRALDIETTAYVLQGYNLNDDTKNGTRILRWFGRRRRSRGGFKSAQDTAIALEGLSELAKKLYVPSTSLTINVKADNLAGRTFNIRDENSLVLQNEDITTLVDHIEVTTSGTGISLMDIDVYFNVMSELRVPAFNMTTALPKDSTKGFRLRICFSYLKDDESGMALLEISLPSGMEADLTSLDTSRTWGKFKKAEKAFRQINLYFESIQSRNMCVELDVNRVSLVARHQAVPIRLSEISEPSNEVIRLYKSKALSTATIIEVCGADNCQELRK | protease inhibitor-like protein-1 [Mytilus coruscus] | ALA16013.1 /0.0 | A2M_N_2(SM001359);A2M(SM001360);Thiol-ester_cl(PF10569);A2M_comp (PF07678);A2M_recep(SM001361) |
| **Unigene51161** | 10.2252 | DHDLSHISSEQLR;EALDMQNDQYR;GLVVEAVDEQGR | 3 | MEQISFFCFRCLTLSVLFKLYFCNPARVDLRDHDLSHISSEQLRRVVQQNENQDITTDDDNLLEKLYNQTQYHPTNDESRCISCTIREDQKKHRIESIKNRISHALRIDVLGKPNMTNTKLPKIPQFQKLKERYEIREALDMQNDQYRGEREDYEDEFGQSHRTFTFAQNPPEELGIQQPNAIYFDMPDQTDRSLQKATLWVYVTPSDQQHVTEIYLYTLVKRSKSSDTLIKQFLYRKKRTSRGWQQFNLLNEVEKWTEDPSYNRGLVVEAVDEQGRNVVVMPSSDDNGYQPVLETRTSPHHQHSRNKRSIYLDCSEQRATEACCRYPLTVDFVEFGWDFIIAPLTYSAYYCAGECRNQHMDSGAHSYLQQQVGNVPTEHGPCCSPTRMGHLSMLYFDHSMQIQFTTLPRMKVERCGCA | myostatin [Mytilus chilensis] | AGU13048.1/0.0 | Signal peptide(1-23)/TGFb_propeptide（PF00688）；TGFB（SM000204） |
| **Unigene39610** | 9.6089 | DGNAGGMSFSSGTGSGNGFAFGGTFNR;GGNGGGFVTSR;VNNGQGIAFR | 3 | MMKALAVLFFIIQVSHGSFGGPYGSSYYNPFQMDKFMYDFFTTFNNIMSMKAPAPRPKPQTFPGAQLFPPTFPDFSGKNSGFKTVLINDMKPGTRKTFKVNNGQGIAFRSRDGNAGGMSFSSGTGSGNGFAFGGTFNRGGNGGGFVTSRSGPKGTKVSYSKGIPKFAKNLFSSFSFF | shell mytilin-3 [Mytilus coruscus] | AKI87980.1/7e-40 | Signal peptide(1-17)/Gly(15.3%);Phe(14.1%);Ser(10.7%) |
| **Unigene35808** | 7.5486 | FTMLVQQLR;INQELEGSR;SFTLADR | 3 | MDPHLCTHIIYSFAKLNGNRLAPFEWNDESTEWMKGMYEKFNSIKQQNPRIKTLLAIGGWNMGSEPFTHMVKTTQSRQEFVKSAVDFLRQRNFDGLDLDWEYPANRGSPPKDKHRFTMLVQQLREAFDRDALTTGRSRLLITAAVAAGKKNIDSGYDVPALGRLLDFISIMTYDLHGSWESNTGHNSPLFARSGETGEQRYLNLDWAANYWNRMGVPKSKLNIGLGLYGRSFTLADRNVNNVGAVASGKGKAGKFTREGGFLSYYEVCEMMKSGGKKYYINEQKVPYLVKDDQWVGYDDVDSLSIKVQYVKQQRFAGIMVWALDLDDFKGSCGQGRYPLLKRINQELEGSRYQPDYSIMNAPSIPDILNQPLAPVAPPPRRRKQKPVRKPPVQTPILPALPPAHHTPLLPPVPRVDQHRTSSKDFTCRKGYDGYFASPDSCSKYYMCTDGTAFKFNCAPGLKFNKEHNFCDWPEKVKCTESTKKSSKKNKNRVQQALLPPNPPIRQPEPPRYEPPPPPQPPPTSANGWNFNANAHAATLPPPVPSSPTNSQQSSAWDWVSMIDNPMPFFMSLFGSNDLFADMCANKANGIYPQRDNCRGFIECSEGVSFKGACGPGLAFNPSQQTCDYTHNVPGCK | chitinase-3 [Hyriopsis cumingii] | AFO53261.1 /0.0 | Glyco_18(SM000636);ChtBD2(SM000494) |
| **Unigene30013** | 8.4664 | GDIASGIGGGAIGGR;GTTGFAGAVSGR;TGAVSNFPVFGPGINR | 3 | GRTGAVSNFPVFGPGINRGFGSSFDGNFGAGFGIGPIGGGFPSFGGPASLASLNAALSGSINAATDGFPGFVGGPLGTLSGSLSGGLNAATGGFPGSIGGPGPLGTLTGALSGGLSAATTGLNAGLNAGLSAALRGDIASGIGGGAIGGRGTTGFAGAVSGRFAGTVGAGAGGAIGKGKVY | glycine-rich cell wall structural protein-like [Crassostrea virginica] | XP_022339053.1 /4.9 | Gly(30.9%);Ala(13.8%) |
| **Unigene2638** | 7.8147 | ADTLSNGPAELR;AGGFEIGMCNR;NYFTQYIHGYGTVR | 3 | MYRIVFPVLLIIVSCLHLSLSQRLRSCVLRAVEGDKRKYKVYLTASRASGEFKCADGTQFYQDKCLCGWPPKNETDNTRPITRPVDLRPDTSRPVVQPVDPRPITRPIDLRPVNPVQPIFPDRPNPNIRPVIPRPPEPCLTEADPDRNYFTQYIHGYGTVRRRCPIGTLYNEVECGCVDLAPVTSIYCKPEIFLDFDSKPINDNGGTHIPIGNNGHVDSVAKAGRFNGIGRLTIWMYSNIDFGEKLTISFRFYDFPGGPEEQVLVSNCMDNELGAVEIALAPRNKEVVFRADTLSNGPAELRLPYKDKSWKNVTFVYDGTALRGKVDEEEQAVAMKGHLDTRAGGFEIGMCNRRGYVGYIDDLKIHRCVDVDYVIGNGNGPPKPYDARRSNLDIAGP | shell matrix protein-like [Mizuhopecten yessoensis] | XP_021364733.1 /7e-123 | Signal peptide(1-23)/SCOP d1c4ra_ |
| **Unigene26** | 10.5492 | AVFVDLEPTVVDEVR;LIGQIVSSITASLR;NLDIERPTYTNLNR | 3 | MRECISIHVGQAGVQIGNACWELYCLEHGIQPDGQMPSDKTIGGGDDSFNTFFSETGAGKHVPRAVFVDLEPTVVDEVRTGTYRQLFHPEQLITGKEDAANNYARGHYTIGKEIVDLVLDRIRKLADQCTGLQGFLIFHSFGGGTGSGFASLLMERLSVDYGKKSKLEFAIYPAPQVSTAVVEPYNSILTTHTTLEHSDCAFMVDNEAIYDICRRNLDIERPTYTNLNRLIGQIVSSITASLRFDGALNVDLTEFQTNLVPYPRIHFPLVTYAPVISAEKAYHEQLSVAEITNACFEPANQMVKCDPRHGKYMACCMLYRGDVVPKDVNAAIATIKTKRTIQFVDWCPTGFKVGINYQPPTVVPGGDLAKVQRAVCMLSNTTAIAEAWARLDHKFDLMYAKRAFVHWYVGEGMEEGEFSEAREDLAALEKDYEEVGVDSVEGEEEEGEQEY | putative alpha-tubulin [Oikopleura dioica] | AAP80594.1/0.0 | Tubulin（SM000864）；Tubulin_C（SM000865） |
| **Unigene25503** | 10.6503 | GSPFMMIDNR;TNPWNENSLYR;YSTQYQQTPNQQHTTMSGFR | 3 | GESDNNEENEESSTQEKQKMQYYGNNDENRLLDDSGIESDDSAQSDGKNDKFDYSDRESFEKEYNSLSSEEDHSEMANNDLNKDYDSDEDSYQHNEIKLNVKTSSSSLNWGYNQPTPTPSSVRTNPWNENSLYRSNVDYDDKDVNVEGDSVNAFKKIVPGFTEKTPSYNYMEIQNNKLQVNTFGSDTNDYSESENEKENEGDDVGDGDENKKWNKFSEEKEDKQQNEHWSAGAVPDIYAGTVIVTSRKRGSRIDNTGKEKENENEIENEDKISSKNSKLLGVKKGKKVSSWGGQEGNSAEKAENEVVEKSPDAENEKPEKGSLDKQKEDENSNNLIGLTTIYQEEGDGKSANEENELKQSVKENSKKSLKEENEMKTLKEENEIKSHKEENEMKSPEEEIKIKTPKEENSFGEMKVDLAGHIGDSNIVKPEEDIGESKSNSVFMNNQQFRNVDDPSEPVKSASNSMGVKPFIGDSPKFMGNMKQAQMFYPTAFPAMNSFTTRGSPFMMIDNRYSTQYQQTPNQQHTTMSGFRKRVQNRISKSMGHNIDDPVMTQPLTLKVKIKSTRKSKKGLSPNLSKLVLKMFMNSKSGAPPCDGNLKSVCRPVKMWSKYQQVADWCTSLCPYGQCPSAVCKCQCTGSPMGSVGQKKCRATNTYKQNSGELDQWCQKTCSKGDCPALLCVCS | — | — | Internal repeat |
| **Unigene12260** | 9.5078 | MTEHGLAR;QNGLGGGNPLICSR;RQNGLGGGNPLICSR | 3 | MDPYLFLCVLAIAFSVHNVYAQMGMGHQNQGNMRRKFPGRQAGMGGGMGGGMGTGNNNQQGMNLGGSPADMGMPGNGMGGQGQGQGHGNGGQGQGNGMNNMGGGGGSGGLMGGMLGGGMPMDAMMGGMQANMMAMGAMNSEIPPHMIMSGTFNPKNFAQYRSCEKTPSNLNTICDPSSPNPCPQGAMISKSTPFAMGMGMAMGGMGGMGMGGMNRRMNRRQNGLGGGNPLICSRMTEHGLARCCAKNMMTARMLDKWFK | — | — | Signal peptide(1-21)/Gly(23.6%);Met(15.4%) |
| **CL93.Contig1** | 10.5492 | IINVVGDPIDER;ILDPFVVGEEHYDVAR;VALTGLTVAEYFR | 3 | VIAQSTVAMMHAARRACVGLLKATKHSLTSPAVPSTATKALPSYFNTRHYAAEQTQPNTAKGRVVSVIGAVVDVQFDEELPPILNALSVENRTPKLILEVAQHLGENTVRTIAMDGTEGLVRGTSCIDTGYPIRIPVGPATLGRIINVVGDPIDERGPVKTDKFLSIHAEAPDFVEMSVTQEVLETGIKVVDLLAPYAKGGKIGLFGGAGVGKTVLIMELINNVAKAHGGYSVFAGVGERTREGNDLYHEMITSKVISLTDDTSKVSLVYGQMNEPPGARARVALTGLTVAEYFRDQEGQDVLLFIDNIFRFTQAGSEVSALLGRIPSAVGYQPTLATDMGTMQERITTTKKGSITSVQAIYVPADDLTDPAPATTFAHLDATTVLSRGISELGIYPAVDPLDSNSRILDPFVVGEEHYDVARNVQKILQNYKSLQDIIAILGMDELSEEDKLTVARARKIQRFLSQPFQVAEVFTGSEGKYVPLKESIAGFQRILSGELDHLPEVAFYMVGPIEEAVAKAERLAEDQS | ATP synthase subunit beta, mitochondrial [Mizuhopecten yessoensis] | XP_021356377.1/0.0 | ATP-synt_ab_N (PF02874);AAA(SM000382) |
| **CL3397.Contig2** | 9.1838 | FADEHVR;SEMDYEETGNR;TPSGGHGDCFVQEMDDGIYAIR | 3 | MPSVEGLKPDIKDCDDGSIIVQYKPSKSGTHEVQMAYEGSATEGSPFSCVVDEIGGGYVTAFGAGLVGGMSGQNQTFTITAKKGTLSDIDINIDGPTKTEYKRSDHGDRCDVTFMPMTPGAYNINIKYKGKTIKGSPFVSKVSGEGRKRSQISLGNASEYALNVMEPDIVDLVGSVRGPKGGFEPCILKKSKDGHLCVSSFSPKVAGDYKVQVYRDEKNIKGSPFNVSVSDKDIANAGKVKVTGATEKAVANESNVLNIDASEAGYGGITMVVEGPHRSEMDYEETGNRTFKCTYSPHEPGIYILNVRFADEHVRGSPFLLNVGGQPSGRIRETVEIDMEQAEPVKKGTKCEFLLKIPGTNPFDMEASVTDPAGTTELCEVMDEDDFHYRINLTPHKDGLHTLSIKHKALHISGSPFQYSVGQLSSGGYHKVQVGGPGVEKGEVGKENHFNVYTREAGVGKLSIGIEGPSPAKIVQERRPNGFLGVSYKVEKPGMYGIHVKFNDEHIPNSPFMVNIAPDSGIARTVTVHALKDRGLAVDKATTFTVSYNGAKGRLNAHLRTPSGGHGDCFVQEMDDGIYAIRYIPKENGVHYIDIKLDDHHIPDSPFAVMVGSAAADPAMVHAYGEGLESGKSGMKNKFVVRTAGAGSGFLALTIDGPSKAALSCKEVDEGYEFSYTPFCPGKYLIAIKYGNINIAGSPYVADITGSGRKPSPVKEQSTMVVETVEKQPGAKSLARFKGDAGRVNVRGPGLKKAVPGRLQSFTVDVKDAGHAMLMVGMVAPSGIAEPELAVKKNTKTEFTVSFKVQELGDHTLSVKYGDEDIPGSPFVLHAA | filamin-A-like isoform X5 [Crassostrea virginica] | XP_022315171.1/0.0 | IG_FLMN(SM000557) |
| **CL2840.Contig2** | 9.4067 | ALVDTETFASPR;ASSYLDDIYYPEPIVR;EATRALVDTETFASPR;ERSLPPPVISLER;GFYDTTR;GFYDTTREENEIR;SLPPPVISLER;TPTPVTTSR;VAVLASPLR;VEVVTPR | 3 | MTVRRSRFQSVPPGYFSSTKGHSSLKRWYPTTTRASSYLDDIYYPEPIVRSRGFYDTTREENEIRRDVNHELLYTSNLVDDTYDIANKSRNRDQMLLREATRALVDTETFASPRSAVTSRRVRQTSVVRTPTPVTTSRAVSCPPVSRGSSQVVVVLTSPAFKGGSRVFSAKSAAMAVRERSLPPPVISLERSKSVHPQLLPSDILEKRINARRIINTVAGPYMLPLYHPYQSIYQPYISMYQPLYARRKYLATLKDVPSRGRVAVLASPLRRKKYRKAALVGNRVEVVTPRKRKPRSTYAANKMRELKRDEREMEVEAVPVQSTTSLKASTVSPNYKGGKLHWDEDGKVTQPTNLMSWQYRIESRVPPGDHLFPVKTIGHVRDKLLHVKEQMDRHRQLMDRYLPDEDSKTDVKTKIMNMYVDMEQHNPAS | RS-rich protein-1 [Mytilus coruscus] | AKS48138.1/0.0 | Arg(11.2%) |
| **Unigene7385** | 5.1611 | HVILLAEGR;IPAINVNDSVTK | 2 | MSTKPPFKVADINLADWGRKCIEIAENEMPGLMQMRKMYGETKPLKGARVAGCLHMTTQTAVLIETLTALGAQVQWSSCNIFSTQDFAAAAIAKTGVPVYAWKGETDEEYIWCIEQTLVFPDGQPLNMILDDGGDLTNLVHERFPQYLPGIVGLSEETTTGVHNLHKMMKDGKLKIPAINVNDSVTKSKFDNLYGCRESLVDGIKRATDVMLAGKVAMVAGYGDVGKGCAHALRAFGARVMVVEIDPIIALQAAMEGFEVTTVEECLPKCRLFVTATGCSSIIHDKMFEQMLEDSIVCNIGHFDCELDVKWLNENCAKKEQIKPQVDRYTLKNGRHVILLAEGRLVNLGCAHGHPSFVMSNSFTNQVLAQIELWTKKEEYKNKISVTVLPKKLDEAVAAAHLDHLGVKLTKLTEEQSSYLGIPRDGPFKPEIYRY | S-adenosylhomocysteine hydrolase [Crassostrea ariakensis] | ACT35639.1 /0.0 | AdoHcyase_NAD(SM000997) |
| **Unigene6555** | 7.7945 | ISTTFVFQAPK;NSYVQQCDCEIIIGR | 2 | DNKVYIVAVLILWTCCPADAVCTCKYLDLQTKLCKSDFALVGRIVSRKPTVDGPRDHLEYTATAISIIKGRISTTFVFQAPKTSSDCGVEFPVRSIQFLMGKRSGGKFVVTSCEVNGQNRPFTTAQWTYLFSRSKRNSYVQQCDCEIIIGRDTPAVGDFCRLVGDGKNQTCYMRNALCRKVGSQGSRQCSWINGEACG | byssal metalloproteinase inhibitor-like protein 1 [Mytilus coruscus] | ANN45954.1 /4e-09 | Signal peptide(1-20)/NTR(SM000206) |
| **Unigene635** | 6.6520 | IEDAGTYVCTAQNLGGIVQQR;VGEPIPDTATVNDVFLVIPQVR | 2 | ICTASSAAGIDRDYVDLKVQGVLPDGNGPIKIDTQTVNIGERVEMECVVTGEPRPTVSWSRVGEPIPDTATVNDVFLVIPQVRIEDAGTYVCTAQNLGGIVQQRVNLFVRARPIISGSQA | heparan sulfate proteoglycan-like protein-1 [Mytilus coruscus] | AKS48136.1 /3e-57 | IGc2(SM000408) |
| **Unigene6047** | 5.3625 | FLEAAYGYR;TFLVWANEEDHLR | 2 | MYRNYITQLDQKKGGQQQGKMAEETQAQLWDKLNKGESKSLLKKHLTPELYEQLKDKKTSLGGTLGDCIRSGANNLDSGVGLYACDPEAYTTFKPLFDAVIKDYHKVDGVNHPKPDFGDVSKLEDLDQYGGDMIVSTRVRVGRSHDGYSFPPCLTKESRKEMMDKTAEACDKLTGDLKGKMYRLESMSKEENQQLIDDHFLFKNDDRFLEAAYGYRDWPNNRGIFHNPSKTFLVWANEEDHLRFISMQKGGNLKEVYGRLVEAIKQLEAKLTFAKKDGYGYLTFCPTNLGTTCRASVHIKIPKLSKLPEFKQICEKHNLQPRGIHGEHTESVGGVFDISNKRRLGLTEFEAIMEMQNGVKEIIKMEKSL | arginine kinase-like protein-1 [Mytilus coruscus] | AKS48144.1/0.0 | ATP-gua_PtransN(PF02807);ATP-gua_Ptrans (PF00217) |
| **Unigene53667** | 5.2544 | CELFYFR;YFFNQQTGR | 2 | DFVTAVPTMKILVLLVVCLTVASVSGFWKWGRRHHRPRRPDCTSPKQKGWGLLKFRRYFFNQQTGRCELFYFRGLKGNRNRYKTLQKCEEACGVATTRTLPITTTAATTTSTLPPITTLNEGGNGGGGGNGGNGGSGGNGGNGGSGGDGGGG | Papilin [Echinococcus granulosus] | XP_024351037.1 /4e-09 | KU(SM000131) |
| **Unigene52025** | 6.5050 | FAESTSALFR;TINSFMEPFQR | 2 | DLQSYLDDLPMGTGLTGRNMHNNNFNGQDLQSYLDDLPPGQRMNGGGLRQFPDHVYENKFKFNRMGGHQSQFGTQNHKVVGDTMKATTRTINSFMEPFQRIRSMNNMNSNQFLGLSKATDLGSNINRITNHRESPEPTSFHHQIRPHATQMSKQRFAESTSALFREPKSANSRFPKHSTLNNVISQTVITEPSLSPMGLGAFLTKQNPIQKLLSPSKPSTTSTLQDNSATKTSVDNQSKLPIQKSLSAVSNTFRMGNNNQVKEFSPKTAKFQNKHI | cyclin-dependent kinase 1 isoform X2 [Trichechus manatus latirostris] | XP_023595093.1/1.1 | Ser(10.9%);Asn(10.1%) |
| **Unigene49324** | 7.7945 | FAPINVENTEENR;NSPAENALATVTALSR | 2 | MPVFPQYLSKEQEDELRQIANAIVAPGKGILAADESTGSIGKRFAPINVENTEENRRRYRELLFTCDKSLAENISGVIMFHETFYQKAKDGTPFPKLLQSVGIIPGIKVDKGVVPLAGTDNECTTQGLDGLSERCAQYKKDGAQFAKWRCVLKIQQYTPSYQAMLENANVLARYASICQQNGLVPIVEPEVLPDGEHDLATAQKVTEEVLAFTYKALADHHVFLEGTLLKPNMVTAGMSCAKRNSPAENALATVTALSRAVPPAVPGVTFLSGGQSEDDATVNLNAINTCPGKKPWALTFSFGRALQASVLKAWQGKDENVKGAQDELMKRAKANGLAAVGKFSGGLAGTAGSDSLFVAQHAY | Fructose-bisphosphate aldolase [Crassostrea gigas] | EKC30386.1/0.0 | Glycolytic(PF00274) |
| **Unigene44900** | 6.6520 | AVGQDLGTSGSCLR;SGIPSGVYFAR | 2 | GVPGLNGRPGQVGPQGDTGLPGFPGLAGSRGEDGLDGLPGINGEPGQPGDSGYPGPAGPPGDDSPYIQGARGDPGIDGIDGRPGIKGHRGDDGLPGLDGLPGMKGNPGFEGFGLKGEPGDNGITGLPGSAGPKGYPGRPGIFGFNGAKGEPGLPGVNGEPGPDGQPGLNGLPGDAGLDGFPGSKGESGDYGYNGQPGLPGTPGENGFRGSKGEPGLNGLPGMEGDVGEQGKTMNPNGYTSGPKGVPGDFGEKGLNGIDGNRGFSGLEGIRGEPGLPGLPGEIGIPGPKGVRGDAGRDGYPGLPGLDGLPGLAGLPGENGIAGRSGIPSGVYFARHSQTTLVPECPTGTSLMWEGYSLSFIMGNGRAVGQDLGTSGSCLRRFSTMPFMFCNINNVCNVANRNDYSYWLSTLEPMTPMMNPISGPPLQDYISRCSVCEATGEVIAVHSQTVRLPDCPVGFKSLWIGYSFLMNTGAGGRGSGQNMQSPGSCLEDFRAAPFIECHSRGTCNYYATSLSFWMATIESFNQFRRPIGETLKAGNLRTRVGRCQVCMRY | Collagen alpha-1(IV) chain [Crassostrea gigas] | EKC43052.1 /4e-172 | Collagen（PF01391）;C4（SM000111） |
| **Unigene42847** | 5.3555 | FTENIETNLAR;YGVDSGR | 2 | MLVISVCVLLCVFSPAFPYDLKFDYQISEGRARFTENIETNLARNTVKYYTPAHNDVLESYKMLDFNKKMQITCLPALKQCRLRDIDVEELTGDAGTVAESFVHSWNKGENSIDSANSRTINEVYYIDNEEVKNTIGLGEDLREFYEKYGKNDKGEGFPLYKEKKLPENAVLLNITRSGVKRTKRAFNPLNNDCNGQAPVTRYGVDSGRSCNYLKICKQAAVVNGQRVFADCGNVHITSPLVYVCVCCPGVTEINLNSNKCACTKMNGP | integral membrane protein 2B-like [Stylophora pistillata] | XP_022807324.1 /0.014 | Signal peptide(1-18) |
| **Unigene38699** | 6.6520 | IQNAGTEVVEAK;LFGVTTLDIVR | 2 | MFSRLAKPSCIVHVARRSFSLTSQAQQGPKVTVCGASGGIGQPLSLLLKNSPKVASLSLYDIAHTPGVAADLSHIETRAKVSGHLGPESLEACLTGSDVVLIPAGVPRKPGMTRDDLFNTNAGIVRDLVEACGNFCPKAMICIITNPVNSTVPIAAEVLKKKGVYDPRRLFGVTTLDIVRANTFIAEAKGLDVSKVNVPVIGGHSGVTIVPIISQATPSVSFPSEERKKISVRIQNAGTEVVEAKAGAGSATLSMAFAAARFTSSLLEALDGGEGQVECAYVQSEETDAPFFSTPILLGKNGVEKNLGRGKLIDYEMQLLEEAMPELKANIQKGVDFVSK | — | — | Ldh_1_N (PF00056);Ldh_1_C(PF02866) |
| **Unigene37757** | 5.0117 | EGGIPSYTTEYR;YGTDITVPNR | 2 | MEYERKVYRKETTREGGIPSYTTEYRIGTDRPRYGTDITVPNRYTTTTYKTTGPIVYSSYSSRPATEYTTYTEPSVEDTRVRKEWDETFKRVAPRADDWSLSDVISKRMVLVKDDEWDPYETSFPEERKRFGKPSPITSDVSGRKTFLVEYQIGDFRPEEVEIKTIGNTLKIHAKNSDSGSMKREYSREISIPQEVNPDLISAKLNRTGRLSIEAPIFNTSHKTKIDRRIPVLRN | heat shock protein 30C-like [Crassostrea virginica] | XP_022323008.1/5e-16 | HSP20 (PF00011) |
| **Unigene3720** | 5.5095 | ISGLIYEETR;VFLENVIR | 2 | YNQYNMSGRGKGGKGLGKGGAKRHRKVLRDNIQGITKPAIRRLARRGGVKRISGLIYEETRGVLKVFLENVIRDAVTYTEHAKRKTVTAMDVVYALKRQGRTLYGFGG | Histone H2B type 1-M [Tupaia chinensis] | ELV13502.1 /1e-66 | H4(SM000417) |
| **Unigene34337** | 4.9215 | VDEHGEFLWPGFGDNIR;VLDWVLR | 2 | ETRSKGRRSVLLSTLHLQFFLQVNPDQKMSVFGEEDTQFYEVHNIVVQHIGPVPIAKGDFHMLPKKVQKFIAKWVDICAPRALYICDGSQHEADEITHKLLERGVLTTLPKYENCYLCRTDPADVARVEAKTWISTEDRYETVPHVREGVKGCLGQWIAPKDLEKEMGERYPGCMKGRTMFVIPFSMGPVGGPISKIGIQLTDSNYVLLCMRIMTRVSPVVLDKLGDGDFVRCVHSVGCPRPVQRKVVNHWPCNPEKIMIAHRPKEREILSYGSGYGGNSLLGKKCFALRIASVIARDEGWLAEHMLIMGLTNEKTGEEKFICAAFPSACGKTNLAMLKPTIPGYKVRVVGDDIAWLKFDKDGVLRAINPEAGFFGVAPGTNMKTNPNAMLTFQKNSIFTNVAETADGGFYWEGMEDEYDKNMLITTWLNQKWHIGAPGKAAHPNSRFTCPASQCPIIHPKWEAPEGVPISALIFGGRRPTGVPLVFETFSWEHGVSVGACVKSEATAAAEFKGKVIMHDPMAMRPFMGYNFGNYLQHWLDLNAPPHKMPKIFHVNWFRVDEHGEFLWPGFGDNIRVLDWVLRRCAGEQNIAEETAIGYVPKKSSFNLSGIEDQVKWEELFSLPKHYWLDDMRESRRFLEDQVGSDVPKTIWKEIEDQEKRIEKML | Phosphoenolpyruvate carboxykinase [GTP] [Crassostrea gigas] | EKC27095.1/0.0 | PEPCK(PF00821) |
| **Unigene32111** | 5.5095 | IEDAQVPSR;IEDTEVQSR | 2 | SLLGKLKEMKKRIEDAQVPSRDTKEMERKTEDLEKSVFGKLKEMKERFEEVQVPSHDVKEMERKIEEIEQYLFDNLNDLKKRIEDTEVQSRGDVKEMKRRTDDLEISLLGKLKEMKKRIEDAQVPSRDTKEMERKTEDLEKSVFGKLKEMKERFEEVQVPSHDVKEMERKIEEIEQYLFDNLNDLKKRIEDTEVQSRGDVKEMKRRTDDLE | apolipoprotein A1/A4/E [Desulfobacca acetoxidans] | WP_013707457.1/0.007 | Internal repeat |
| **Unigene2736** | 6.6520 | NGPFTVLR;TQGSYTEIQECFAR | 2 | MEQKLFLIGVVLMCSLLSVNSLCNFPCSVQTGGDYDGELGSFRWSCDNSSRLIRTQGSYTEIQECFARNGPFTVLRRNGNQYQCVKEAAVTGKVTWVYETEFLTMWNPPTVCSICTPVLMRPMMYVDPSVICIPGTKKKGQYTLKSLKKMKPPPIGCNRPKNCPLSSTLDVPCTGCEPFDDGSCCPGCKRKLQQSYAYAYAYADSMFGPQFMGHYNTML | — | — | Signal peptide(1-21) |
| **Unigene24855** | 5.3625 | GYSFITFVDR;TGYSLDVTTGQR | 2 | MADVNGLDHKDQNMAESDVKPEEYVKLVEYGINARVAEELCNVYKTEKLTHAELDERALDALKEFNADDAIAVLKQFLDSPLEHVTNKSAYLCGMMKTYRQNKKQGSTATVAKGPDEAKLKEILDRTGYSLDVTTGQRKYGGPPPGMDESSQPGAGHEVFCGKIPKDVFEDEIIPLFEKCGQIWDLRLMMDPMTGFNRGYSFITFVDREGANEAVKQLDNYEIKSGKRLKVNISVANQRLFVGNIPKSKTKEEIMEEFSKKTEGLQDVIIYRSADKENQKNRGFAFLEYDSHKSASTAKRKLSSGRSKVWNCDVIVDWADPIEEPDSGTMSKVKVLYIRNLSSDVTEDVLKEKFSEYGKIERVKKIKDYGFVHYEERDDATKALEAMNGQKLGKLEIEVSLAKPPTENKKKEQRKREQERRQMMGYDDFGYGPPMPPGRMMPPGRGMRRGMPPPPRFDYYDDFFGYEDYNDYYGGGYAPQMPRVRGGRGAPPFASANARTRRGPTPWWT | RNA-binding protein [Pinctada fucata] | ABP04054.1 /0.0 | RRM（SM000360） |
| **Unigene23862** | 6.1542 | IILCDLWK;SVGEVNTGEIYYTVR | 2 | IEVKMLLIVALSLLALVVGVDSCTCAQSYLHQDYCKSEFLIRAKVLSMTRSVGEVNTGEIYYTVRVYNIYKNSSYFIGKSTVDIWTPGNDGICKVPLLIDKTYYLGGTVDTKRSRLRIILCDLWKLTADLKVCQKKLIAQGQFDCTCKDPSCFQDC | metalloproteinase inhibitor 3-like [Crassostrea virginica] | XP_022302578.1/6e-10 | Signal peptide(1-22)/C345C（SM000643） |
| **Unigene19938** | 5.9950 | EAYPGDVFYLHSR;VLSIGDGIAR | 2 | LQSAILVGHRREQELVKMLSARFAATLVRQLPRAAPKVCRHALGAGYVASRNISTSTPLCAGAEVSSILEERILGQTSQTNLEETGRVLSIGDGIARVYGLKNIQAEEMVEFSSGLKGMALNLERDNVGVVVFGNDKLIKEGDIVKRTGAIVDVPVGKEMLGRVVDALGIPIDGKGPLGTSTRARVGVKAPGIIPRISVKEPMQTGIKAVDSLVPIGRGQRELIIGDRQTGKTAIAIDTIINQKRFNDGTDEKAKLYCIYVAIGQKRSTVAQIVKRLTDADAMKYTVIVSATASDAAPLQYLAPYSGCAMGEYFRDNGMHAVIIYDDLSKQAVAYRQMSLLLRRPPGREAYPGDVFYLHSRLLERAAKMNDDNGGGSLTALPVIETQAGDVSAYIPTNVISITDGQIFLETELFFKGIRPAINVGLSVSRVGSAAQTKAMKQVAGSMKLELAQYREVAAFAQFGSDLDQATQNLLNRGVRLTELLKQGQYIPMPIEEQVAIIYAGVRGHLDKLDPTKITDFEEAFLQHIRGSQKDLLATIAKDGMITEDSDAKLKQVVKNFLAGFEG | mitochondrial H+ ATPase a subunit [Pinctada fucata] | ABJ51956.1 /0.0 | HAS-barrel(PF09378);ATP-synt_ab (PF00006);ATP-synt_ab_C(PF00306) |
| **Unigene17542** | 6.6520 | SLVIMQGGHEVESER;YSWINIFGDDSVLGR | 2 | MKLLMLSLVIFAALALQVRADGQCTPNTSSKNHDDPHDDNHKDDQHGDDHHDDDHHDDDETMHYAQCEMEPNPHMASNLHHHVHGSIELSQKGHGAVYLEVHLVGFNTSEDHADHHHGLHLHMLGDMSAGCDSIGDLYNAHPEKHANPGDLGDLVDDDRGVVNEVHHYDWLDIDGTAPNTEALIGHSMTILQGSHKDPDTPASRIACCVIGHGKARPKTAAALHHELEEDKTEHYAHCDVRSNTHQPKALHHHVHGTIDMKQVGYGDLEVTYHLEGFNVSDDYKDHLHDVQIYTNGDLTSGCDNLGAKYDPHEDYHSDLGDLGDIHDDDYGVVNESHRYSWINIFGDDSVLGRSIAIHQRDHLHTSAKIACCVIGRGQSHPEIVHKAKCVVRPNTESTGLHHHVTGNITFEQTPGGATHMTADLTGFNVSEDLSHHRHGVQLHEWGDMSNGCHSLGRMYHGHDDPHDPKRPGDLGDVIDDSNGDVHATRALDHINVEDLNARSLVIMQGGHEVESERVACCVIGRA | pernin precursor [Perna canaliculus] | AAK20952.1/0.0 | Signal peptide(1-20)/Sod_Cu(PF00080) |
| **Unigene17159** | 6.6520 | DGDVDAAVVGDVCR;EGQGYISGAEMR | 2 | MSKLSKGEIEDAREVFDLFDFWDGRDGDVDAAVVGDVCRCLGINPTNAVIKKNGGTDKMGEKGYKFEDFLSIYETVNQQTEQGTYADYMEAFKTFDREGQGYISGAEMRQVLSSLGEKLTDEQVDEIIRLTDLQEDLEGNVKYEDFIKKVMAGPYPD | myosin essential light chain [Crassostrea gigas] | CAD91423.1/3e-88 | EF-hand_7(PF13499) |
| **Unigene16016** | 6.3969 | LNPLEAIQEAATR;LTHLHIVSEHDIPR | 2 | ETTSERYTIYVNGIIYTVNPKDSDWDKYPEEAMVINQITGKIVFIGTTQKALKYDLENSSVYNLDGSTVLPGFHDVHMHPLEAMTSLGGGCKIPGDTDPEHLLGQIAECNPNDGGNSWVLGHGHSIESLLKHIHNRGRPPREIIDEVIPDNPVVLMEETSHSVWVNSLALDLAHISKDSDDPVGGIIMRDSETLMPNGILLENAGNMMMDVAMASFTNLNQSNYESLKASLIELKKNGITSICDARTYWKRSHHLAWKKACINEELTVRVNLGLWAYPNMEDSEQIRSLKELYTNDFSRRCFLKINEIKLYSDGLLETTTAAMLEPYLKNLRLPGLQENIGMNYFSQSRVEKYLKALQSFGADKGFNFHIHAIGDRGIKEILNAISNTQTDGTRHRLTHLHIVSEHDIPRFSSLGVVADFQVTNTHIPAVEEAVGLKRAENYIPVRSIEKTGAKTTLSSDWDVSRLNPLEAIQEAATRDRQAVSVKSAIEMKTVNSAYAMRQDDIVGYLGNNMDADFVILSRDILSIDTNNIGKTEVLLTAIQGKVIYQKFNDYPSKE | amidohydrolase [Colwellia psychrerythraea] | WP_081967745.1/4e-161 | Amidohydro_1（PF01979） |
| **CL955.Contig5** | 6.6520 | DSYNLVER;GQLEISNVR;ITELEAELDNEQR | 2 | QAALEEAESALEQEEAKVMRGQLEISNVRSEIERRLAEKEEEFENTRRNHQRALDSMQASLEAEAKGKAEAMRIKKKLEQDINELEVALDASNRAKAELEKNIKRYQQQVTELQVQVEDEQRQREEARDSYNLVERRCNMLQGETDELRTALEQAERARKSAENDLYESNDRVNELSAELSSMSSQKRKLESDINAMQTDLDDMSNEIKAADDRARKSTADAERLANELRSEQEHSMQIEKHRKSIESTVKDLQIRLDEAESQALKGGKKIIAKLEQRITELEAELDNEQRRHAETQKNMRKADRRLKEIAFQADEDRKNQDRLQEMIDALNNKIKTYKRQVEEAEEIAAINLAKYRKVQQEFEDAEERAESADSALQKLRMKNRSSVSMARTTISSTTTTS | catchin protein [Mytilus galloprovincialis] | CAB64664.1 /0.0 | Myosin_tail_1 (PF01576) |
| **CL818.Contig4** | 4.7938 | QLDNVGYR;TGYLYR | 2 | MSAPQVNGPSSPTGKYIDNIDPDDPEYQRQMRRPAEIKEDVKQMQDRSRVSLVLNSEAFRKELEEIIKEQFSEGNCPTNLLALQQITELLNPNNKTQSSGGVWRGVPSVIPINDIKGTETAKYAKHEKQLRCKVASLYRIIDMNGWTCGIYNHISARINQEQEHFLLNPFGVLYSEVTASSLVKIDMQGEVIDPGSTTLGINKAGFTLHSAIHQARPDIKCIIHLHTPEAIAVSTMKCGFLPLSQEALIVGEVSYHDYNGILVDEAERDQLQRSLGPNNKVMFLRNHGVVACGSSIEEAYHYAVNVMSACTTQTKAVPAGIDNLILVSEEIKKLTFQVGSQGGGGVDTGGRKWKTGELEFEAVMRQLDNVGYRTGYLYRNPHIKQEVKKEKNNSDVEIPPTSSSFTYVFDGDIEHSKYISPLKLAKDRQKQNYKAGWLVNSPNTYTRQEIEETGTPNPKKYSKWIAEADGSPNRKCTPIRLENPNQFAPQGENPKEYREKQKAIRKDYYEERISAGPQSKILEGITWEEAKQLQDGNLSMVGDNVVIYGAASKGIIQRDQQHNVQVYKTQYAANPFDSINEEEIEKYKIEVEQRGKGDAEPTEDLSPGPDGKLISTEERMQIIQQQQVETTPEHKPEVEKPKPEEKAPAVRRTSSNREPPRSPELIQELKEKNFERSKSERFGRDRVLNGDEKPSSPAKSDTLKSTDSASGGDTLDDRSSKEGSPVKELPSPTKDKKKKKKFRMPSFSKTKKNKDSKESTL | Protein hu-li tai shao [Crassostrea gigas] | EKC20098.1 /0.0 | Aldolase_II（SM001007） |
| **CL758.Contig1** | 6.3279 | LTGMAFR;VPVPDVSVVDLTVR | 2 | MKVGINGFGRIGRLVMRAAIDKGVSVVAVNDPFIDLDYMVYMFKYDSTHGCFNGTVEAKDGKLIINGNAVAVFGERDPANIPWGANGAEYVVESTGVFTTKDKASAHFKGGAKKVVISAPSADSPMFVMGVNEEKYTKDLTVVSNASCTTNCLAPLAKIINDKFGIIEGLMTTVHAITATQKTVDGPSMKDWRGGRGAAQNIIPSSTGAAKAVGKVIPELNGKLTGMAFRVPVPDVSVVDLTVRLQNGASYDNIKKAIKEASEGPMKGIMGYTEDDVVSQDFRGDNRSSIFDAKAGIALSETFVKLVSWYDNEYGYSCRVIDLLKHMSKVDSA | glyceraldehyde-3-phosphate dehydrogenase [Littorina littorea] | AJA37895.1/0.0 | Gp_dh_N（SM000846）；Gp_dh_C(PF02800) |
| **CL678.Contig2** | 6.6520 | AAAAAGAAAAAGAGAGAGGSSGLSAALR;VVIQLLTR | 2 | GGLGGGLGGGLGGGLGGGLGGGLGGGADAELELFEDLLGTYGLDIFEGEEGLAALSLLGGLGAGAGAGAGAGAGLGLGGAGAAAAAAAAAAAAAGAGAGAGAGAGAGAGAGAGAGAGAGAGFGGAGGSAAAAAAAAAAAASARSRAAAAAGAAAAAGAGAGAGGSSGLSAALRSRLLARIAARRAAASAAAAASAAAAGGAGGAGGAGAGAGAGAGAGAGGGAGSGAGAGAGAGAGAGAGAGAGAGAGAGAGSGGARGILGWLLRRRAMARAAAAAAAGAGSGGSGGSGGNGGGSDGDCGDSDSDSGSDSNGDNDTDSSDSEGSDGSDSDSGSDPDGDGDSDSSGSSNSSDDNGDSGDYDSGDDGDGDDGGDFKAVAKVVIQLLTRVLSSGVLTAGASAGASASAGAGAGAGAGLGGGFGAGGGAGAGAGAGAGAGFGSGIGLGFGGGFGGGFGGGAGAGAGAGAGAGAGAGAGAGAGAGAGAGAGAGAGAGA | — | — | SCOP(d1gkub1) |
| **CL579.Contig12** | 6.6520 | ILTDDLPR;VAVSPIVTVEPR | 2 | EDEVTGKRITRILTDDLPRYFALVTKIKEEKRQVGDEGGVISSTVVPQVQAVFPEGALTKKIRVGLQAQKINPDLVAKMFGNRVAVSPIVTVEPRRRKFHKDIILTIPVPKAAQKGMINQYGGDAPTLRVLYSIAEGTDAAVWDDYTNNTKLEFSEDCVSFTTRVSARFWLMDCQNIADAPRMATELYHEAITVPYMSRFVVFAKRNAEEEGKLRMFCMTDDKIDKTLEKQEHFVEVARSRDVEVLDGRPQFVEMAGNLIPITKSGDQLYINFKAFRENRLPCTVRIRDMDQDPAARVAFMKEPKVARGEVPQTPICNLNVSLPDMSTSSSMEMDGEAVRELRNRSSLIKEHDIVVQDRVQQASIRLTDVADTVKGDWVMLAQQLDVSSSEINDINNQYKTVNDQGLAMLQLWVDKDTQPNKGQVLEKALVKIGREDVVKKCIYNVENVEDEMEAAAARVAMDQSGFDTFTEEVGISKEDSMKRNMSLDVQFDEQDMVKESESIAESPSSDGKHAPQALEEETEPRDDEVVVQVDERRPLAKQEVKGEKQADDYMDLIEQLDRYCDEKEQKESEGQDTDKPLVFEDEITHTKHERMTPEEIISIVESIGQQSAPPPRQDDDDEGVHTPPPSPAERDQYEDEEEECEETTEIVETYTERIDDNTVRTIRKTTTINSHGTTIRTEILKEEQHRDASLDQLAARLSQTDEPAGGANETGV | titin-like [Mizuhopecten yessoensis] | XP_021347443.1/0.0 | ZU5(PF00791);DEATH(SM000005) |
| **CL474.Contig2** | 5.5095 | LTIVTDCIR;YGRPNPGVCPVPR | 2 | VGEDKICCPAGKCHVKDCVYPPRYGRPNPGVCPVPRALTPLQCSRLTIVTDCIRDTDCRDGKKCCETGCDVLKCLTPMINRPECPSVKCTQYTDLCKSDKDCKYGEVCCYGENSDAYCKKCVKRDTTNGHSRRKHSSAPVPMCLTCCGPPPCCGPCISCGAGQNSSHMFCNL | fibrillin-2-like [Stylophora pistillata] | XP_022806910.1 /0.003 | WAP（SM000217） |
| **CL4671.Contig1** | 6.7531 | GPVLLNIDTVDAASGQR;SGPASVAGVSAPR | 2 | MLPVVLLISLFAAGTLGTGYVPPPKLPKKVVSLPIPKPVLKGKPLRQDTAFDHHQIVPIKKTEVRRVVEKVPLVHEVPIFITKNKPFYQPQVVPKPIVVHEVVGLPIVRDKHVVHPVYIDRPYIVEKKQFVEQPFPVEKPIPIDFIKRITKVYDRKVEVPHIVNIPLVKTVDRPRAVPYTREVVQHYDVHVNVPRPRPVKVLRHKTHVEKVPFETPNLIVRKNPVHHAVNEYIPKVPQGFNGRVFAGTQEDAAVTAEASRFGPGSGGRTISVSRGPGGPVAVPQRGPVLLNIDTVDAASGQRIPDGAVVARGARSGPASVAGVSAPRQGGAPVLLGQDLSGPGVGSLGPAIPGGPGPVGVEAVAVGGPGPVGGPGPLVGGGIGGAAVVVGGGGGGIGGGAGVVAVGGGGGGVDLGAGPVVVDGGRGGGAGPIVVSGGGGGGGVDGGFEIVDVGANGEGIINIGPGGLGQDIEIVAVGPDGKDIGSFVVGAGDIAGGTGKKGKK | valine-rich protein-like isoform X4 [Crassostrea virginica] | XP_022307204.1/3e-09 | Signal peptide(1-17)/Gly(18.3%);Val(16.7%);Pro(11.3%) |
| **CL3748.Contig1** | 6.6520 | ITNWWSEHHGSLR;LAAQYAEAQEEIQR | 2 | MPKAVNVRVTTMDAELEFAIQPSTTGKQLFDQVVKTIGLREIWFFGLQYVDSKGYTTWLKLNKKVLSQDVKRETPLQFKFRAKFFPEDVTEELIQDITQRMFFLQVKDCILSDEIYCPPETSVLLASYACQAKFGDYNKETHPNGFLANERLLPQRVMEQHKMTREQWEERITNWWSEHHGSLREDAMMEYLKIAQDLEMYGVNYFDIKNKKGTELLLGVDALGLNVYEKEDKLSPKIGFPWSEIRNISFNDKKFVIKPIDKKAPDFVFYAPRLRINKRILALCMGNHELYMRRRKPDTIEVQQMKAQAKEDKMSKQQEKQRLESERIAREEAEKKQKEMEEKLRRFEEESERRAKEMAEQERRLRDMTEEMEAYKRQQEELEEQRRAAEELRRQYEESAHLAQEEKDRLAAQYAEAQEEIQRSMAVLEEKESEMNSMQQNLEQAQKEREEKEQALIEAMNTIHVRETEHEENTVEEVNHEYSQVETSEDVHMTFENEYEENTVEEMNHEYSADLQDYEQVESLPRPEEERLTEAEKNQRMKEQLKSLQEELQNTKIDEKATTTDMLHAENVKQGRDKYKTLKQIRQGNTKKRIDEFESM | radixin-like isoform X4 [Crassostrea virginica] | XP_022292008.1/0.0 | B41(SM000295);FERM_C (SM001196);ERM(PF00769) |
| **CL3668.Contig2** | 4.8186 | EGENLYLACDSR;IDNGQNQPAVR | 2 | GRCTKFGYGGCDGNANNFATREACGNKCGDAYTPDGTTSISPSIPKEQCQQPKDFGNGQGREVMYYYNPAVGDCQPFWYSGAGGNNNRFRNSTRCRTVCRRDYTRVPVVTSRPPVTTTPPTIPSPDPGTDVIITGNECDENSERGPCTNYTVKWFYNRSQGRCTRFWYGGCEGNGNKFDSEAACKARCVEKVYTGSETADLVDGNATVIIEQPDRSLLIECRGPQGTVAWYKNARILTSDQRFTVFENGSLFIAQLNKDDSGVYACRIDNGQNQPAVRRFRLQIEVPITIFPTPSTIVVRPGENAFLHCQAYGSPQPTVTWTKDGQSIASGGRYYMYQNGTLIISNTQQTDISDYTCTARNGRSSPAQRLVRLSLQESVSASIKPIDGRLREGENLYLACDSRGFPPPTVRWEKMGVELITTGRMTVNGPNLRITDLTLDDTGSYTCVVNNAEDSAKDSKSIQVIPKDVDIPECVDSASVTMCRLIVRAGLCGYTDYSKQCCYSCDQSKLRG | Papilin [Mizuhopecten yessoensis] | OWF36203.1 /9e-150 | Kunitz_BPTI(PF00014);KU(SM000131); IGc2(SM000408);PLAC(PF08686) |
| **CL2840.Contig3** | 6.6520 | ALVDTETFASPR;ASSYLDDIYYPEPIVR;EATRALVDTETFASPR;GFYDTTR;GFYDTTREENEIR;GSSQAALVGNR;GSSQAALVGNRVEVVTPR;TPTPVTTSR;VEVVTPR | 2 | MTVRRSRFQSVPPGYFSSTKGHSSLKRWYPTTTRASSYLDDIYYPEPIVRSRGFYDTTREENEIRRDVNHELLYTSNLVDDTYDIANKSRNRDQMLLREATRALVDTETFASPRSAVTSRRVRQTSVVRTPTPVTTSRAVSCPPVSRGSSQAALVGNRVEVVTPRKRKPRSTYAANKMRELKRDEREMEVEAVPVQSTTSLKASTVSLVRY | RS-rich protein-2 [Mytilus coruscus] | AKS48164.1/4e-65 | Arg(13.7%)；Ser(11.8%)；Thr(10.9%)；Val(10.4%) |
| **CL1757.Contig1** | 5.1185 | FPGQLNADLR;ISEQFTAMFR;LHFFMPGFVPLTSR;YLTVACMFR | 2 | MREIVHIQAGQCGNQIGSKFWEVISDEHGIDPRGLYHGDSDSQLERISVYFSEATGNKYVPRAVLLDLEPGTMDSVRSGPFGQLFRPDNYIFGQSGAGNNWAKGHYTEGAELVDSVMDIIRRETEGCECLQGFQMAHSLGGGTGSGMGTLLNSKIREEYPDRIMTTFSVMPSPKVSDTVVEPYNATLSVHQLVENTDETFCIDNEALYDICFRTLKLTTPTYGDLNHLVSATMSGVTTCLRFPGQLNADLRKLAVNMVPFPRLHFFMPGFVPLTSRSSQQYRALTVPELTQQMFDAKNMMTACDPRHGRYLTVACMFRGKMSMKEVDEQMLNVQNKNSSYFVEWIPNNVKTAVCDVAPRGLKMSATFIGNSTAIQEIFKRISEQFTAMFRRKAFLHWYTGEGMDEMEFTEAESNMNDLVSEYQQYQDATVEDDMDFEEEEGEADEF | tubulin beta chain isoform X1 [Parasteatoda tepidariorum] | XP_015920593.1/0.0 | Tubulin(SM000864);Tubulin_C(SM000865) |
| **CL1387.Contig2** | 6.3279 | AGFAGDDAPR;AVFPSIVGRPR;DLTDYLMK;GYSFTTTAER;GYSFTTTAEREIVR;HQGVMVGMGQK;IIAPPER;IWHHTFYNELR;QEYDESGPSIVHR;SDEDVAALVIDNGSGMCKAGFAGDDAPR;SYELPDGQVITIGNER;TTGIVLDSGDGVSHTVPIYEGYALPHAIMR;VAPEEHPVLLTEAPLNPK | 2 | MSDEDVAALVIDNGSGMCKAGFAGDDAPRAVFPSIVGRPRHQGVMVGMGQKDSYVGDEAQSKRGILTLKYPIEHGIVTNWDDMEKIWHHTFYNELRVAPEEHPVLLTEAPLNPKANREKMTQIMFETFNSPAMYVAIQAVLSLYASGRTTGIVLDSGDGVSHTVPIYEGYALPHAIMRLDLAGRDLTDYLMKILTERGYSFTTTAEREIVRDIKEKLCYVALDFEQEMQTASSSSSLEKSYELPDGQVITIGNERFRCPEAMFQPSFLGMESAGIHETTYNSIMKCDVDIRKDLYANTVLSGGSTMYPGIADRMQKEITALAPQTMKIKIIAPPERKYSVWIGGSILASLSTFQQMWISKQEYDESGPSIVHRKCF | beta-actin [Meretrix meretrix] | AEK81538.1/0.0 | ACTIN（SM000268） |
| **CL1116.Contig1** | 7.7945 | EAEAAGEEESASER;EEAMAAEAESSSTR | 2 | MVIKVYISSVAGSSKMRKEQQHIKDILQSLKIDFEEIDISDPQNEEPKKFMRANSKTKKEGAVPLPPQIFVDDEYCGDCEDFDMSIEEQTMYEFLKMASPVKKSEATVTVTGEKSELIEDIAKQDEEKEKEDKENEKNKTETTDENKDETEKKDEDDKKEEKPVEEEKESKEEAKTEEKDSEEKKDEVDESATFTISSDVTIDEPDNKAEDTVDKNTEEEATFELKTEETVEEDVKKSDDTKEETKTDDTEKIEEKTEESKTEEKAGEKQKEEEKVQDSSIEESRIEETAKPDEKEAEPEKQEEKVEPVKEEVTVEEPAKEEESKEEEKKEEPPKEEKVEESPKVEEAVPEPVDMDEEDNSLEARRRRRRLEREKKEREEAMAAEAESSSTRRRRGEEAEEEPSGSESLEERRRRRREERAKREAEAAGEEESASERRRRRRREVMDN | SH3 domain-binding glutamic acid-rich protein-like isoform X5 [Mizuhopecten yessoensis] | XP_021366845.1/8e-25 | Glutaredoxin（PF00462） |
| **Unigene9746** | 3.8972 | AYGPGLSEGVCNQPAR | 1 | PTKTSKIRAYGPGLSEGVCNQPARFTVETNGEVGALGFSIEGPSEAKIDCQDNGDGSADVTYYPTSPGEYAVHILCNDEDIPESPYMAQIAPATNAFDASKVIAEGPGLQKTGVTTNKYAEFTVDTRKAGKAPLKITCEDDQHKPVNVEIVDKKNGTFACKYMPKKQCKHTVTITYGGVQIPKSPFKVNVGEVSNPGNVKVYGPGVEKGVKTFKTTYFIVDCKSAGPGDIAIALVDAQGKDVPVNTIDQKDGTFKIEYTPNSPGTYIVSVYFANQEIPKSPIKVNVESSIDLSKVKVVGLDTPIKIGEKRDISVITKGCGKADGPVKVTMITPSKKKVNIPVKESLETWKG | filamin-like protein-3 [Mytilus coruscus] | AKS48150.1 /0.0 | IG_FLMN(SM000557) |
| **Unigene9334** | 3.8972 | ELLNYGAYVDQIQDR | 1 | DKMADNMKTSIVCFLIVLAISGIHSTTPVVLWHGMGDSCCNPLSMGSIKKLIEKKVERVYVNSLEIGSNIEEDTLNGFFLNVNTQISMVCDMLAKDSKLKGGYNAIGFSQGGQFLRAVAQRCPNPPMLNLISVGGQHQGVYGFPRCPGNNETICNMVRELLNYGAYVDQIQDRLVQAEYWHDPLNEDEYKKKSVFLAEINNENVKNATYKTNLLKLKNFVMVKFLDDGMVQPRDSEWFGFYNAGQAKTVYNLTQSKLYTEDWLGLQQLDKSGRLHFLSSPGDHLQFTEQWFIDNIINKFLK | palmitoyl-protein thioesterase 1-like [Mizuhopecten yessoensis] | XP_021340150.1/5e-156 | Palm_thioest /PF02089 |
| **Unigene9058** | 2.7547 | VTFQQAAER | 1 | MTSGISPDDPDYQYLCVDRKALMKEQTVTFDGKKNCWVPDEKLGFVAAEIQSSKGDEITVKTVEKMEMRTVKKDDIQQMNPPKFEKIEDMANLTYLNEASVLHNLRSRYGSGFIYTYSGLFCVVINPYRWLPIYTDSIIQKFKGKRRSEMPPHLFSISDNAYQFMLQDRENQSILITGESGAGKTENTKKVIMYFAKVAASLGKKDKEEETAAKDKKGNLEDQIIQANPVLEAFGNAKTVRNNNSSRFGKFIRIHFGPTGKIAGADIETYLLEKSRVTFQQAAERDYHIFYMLLSNAFPKYHEMMLLTPDPGLFSFINQGALTVDGIDDVEEMKIADSSFDILGFTEEEKTSLYKCTAAIMHMGETKFKQRPREEQAEADGTAEAEKAAFLLGVNAGDLLKSLLKPKIKVGTEVVTQGRTKEQVVYSVSAMAKSLYDRMFKWLVMRVNQTLDTKNKRNYFIGVLDIAGFEIFNFNTFEQLCINYTNERLQQFFNHHMFILEQEEYKKEGIQWEFIDFGMDLQACIDLIEKPLGILSILEEECMFPKADDKSFKDKLFANHLGKSPNFGKPGAASKGKGQSDFELHHYAGIVPYSTVGWLDKNKDPINETVVELLSHSKEHLVQTLFAPAKEEPGTTHKKKKSSAFQTISAVHRESLNKLMKNLYSTHPHFVRCIIPNEMKQPGLIDAGLVLNQLQCNGVLEGIR | myosin heavy chain, striated muscle-like isoform X6 [Crassostrea virginica] | XP_022317649.1/0.0 | Myosin_N (PF02736);MYSc(SM000242) |
| **Unigene9037** | 2.2570 | YNQILR | 1 | PFSQRADYHHNIRLKGWKNFSNLCNMPIKAVKARQIFDSRGNPTVEVDLTTDKGLFRAAVPSGASTGIYEALELRDKDPKAYHGKGVSKAVGHVNNDIAPKLVSSGLDEADQTAVDQFLLSLDGTENKDKLGANAILGVSMAVCRAGAAKKGVPLYRHIADLAGNKDLILPVPAFNVINGGSHAGNKLAMQEFMILPTGATSFSEAMKMGSETYHHLKAVIKKKYGQDACNVGDEGGFAPNILDNREGLDLLVSAIEMAGYTGKIKIGMDVAASEFCKDGKYDLDFKNKNSNPNDYLSPDALGDLYRGFVDNYPIVSIEDPFDQDDWSAYTKLTGSVPIQIVGDDLLVTNPKRVQKGIDVKACNCLLLKVNQIGSITEAIQAWKMSKLENWGVMVSHRSGETEDTFIADLVVGLCTGQIKTGAPCRSERLAKYNQILRIEEELGANAKYAGDKFRFPY | RecName: Full=Enolase; AltName: Full=2-phospho-D-glycerate hydro-lyase; AltName: Full=2-phosphoglycerate dehydratase | O02654.1/0.0 | Enolase_N（SM001193)；Enolase_C(SM001192) |
| **Unigene89** | 2.2570 | FGAATGHELQR | 1 | MSFSHIFGTAVFFINVIYVFAQRPSQIGQNPFAATQGFGMRQGMQNMFNGQRRFGGGNGGFNIPPPAGFQQVNRQFQNNMPFQLQNALPRNPTADGNNVNGVLSVLNGGGAGFTGRFGAATGHELQRQLTLTGEPGAVFTGTNNPQARPGFTQFNQRQNQLPSAQLNRIQQPNMFLNRPGQNPNMPFQRPPMMQFGQQFGRQGMIQPSVVGNVPPTQSRGPRNQPQAVMPMNVQGIDMSSILPGQRLPNEPSLVNRGMPSNQQQGQTTRFGRNTMMQPQQPQTFGVPSQTIRAPQATIRQQPSQTFGPQQIGQPQTFIPPQPVAPQPSRPTQIASQGQSSRTSIAFPDQTLTQSLGVGRPASQVNVPDGAPPMLPGEVEIAPPISPAEAAQMQGSSANPPQAVSEIITISDINGNVLYRGVDLSDTEIEKITQQLVSNMTDAQAAEAAARGRELEAQGKLDSAGAPQSTGQTSSASLSSQPSTQQASGGFSSWGQQSPGIRFPGQNVVGESRTVVDFLNPSNTNEG | — | — | Signal peptide(1-21)/Glu(15.9%)；Ala(11.7%)；Arg(10.0%) |
| **Unigene8424** | 3.8972 | YMYTANSWYDISGR | 1 | MVLVLLLCLFPTTIASAVNNDPCGVSGNWTNQFGSLMVVSCQTGPVVPSEGSVTGRYMYTANSWYDISGRYTMINGDCILGFSIAIGNNYGNSNITSWTGIHYASEDNIHTQWFLSK | Chain A, Streptavidin Mutant With Osteopontin Hexapeptide Insertion Including Rgd | 1MOY_A/5e-05 | Signal peptide(1-17)/Avidin（PF01382） |
| **Unigene7565** | 2.8558 | TVLRPCPGGTAFDIR | 1 | KDSSPATTTSFAALAPKPNPTVPVFLQQKKKVAANLKNARRNVLNLRDQSFQKEISKPIPNKTSVQNTNKITRKDRTKQTSKPSRKLPDSALSRKGRPINTGSLSLTKDLKQEVAMLLEILMLEHDRMIKGKKKKKQKKTKKKLKTDTKIPSTTPTTLWKSVKRENKIKLPSKDKLLSPQRIRNSLKPRNGEEQNSWNFRRPASFRPGSEIKRQYLSQKDKQTTDEESKSDNDNCQLKPYDKDSSFYIEKTENRTVLRPCPGGTAFDIRSCACTIMTVTRSISDCEPEILLDFNNNMKDKSGKNIHVGADHIEIHNGSALFLENGDITIWRFTGSHIGRQLMIQARFKATKDPETRQYVISNCNYGTDISYGIEIDRVDEVVIFTLDTEPRDQKEIKIPFELDKWTSVSLIYDGLRFSCEVNGKKKSMPLAGNVETRSSPLRIGGCGVKQNRFSGYIDEVSMSFCIPKKRKGLLSG | sushi-like protein [Mytilus coruscus] | AKS48157.1/5e-27 | Laminin_G_3 (PF13385) |
| **Unigene7251** | 2.8558 | QGPSGDTGPR | 1 | MMLSRVRWGLFGLLVVIWNVNAQKACKYQGNDIGVEEEFVPDENVPCRTCKFDQDCRLHCDFKSCDPLECLEGQTEVLHPGQCCPVCEELILVDKSLKNPKVDGKLVNDEKAGVWGGAVGPGSANGYVVGAPGPRGFTGPPGPPGPVGYQGPRGEPGEPGQPGPSGERGFPGPSGPPGSPGEEGLPGEQGPTGPIGSLGNAGQPGMPGMPGPKGHRGFPGQTGKTGDEGRPGEKGPAGPSGAPGSPGPMGPRGPPGERGRDGSPGPQGIRGQDGKRGDSGPPGPIGSSGSPGFPGSSGPKGDSGQPGQRGEQGLQGPPGVSGLPGPPGESGNPGRPGQDGDPGIKGDLGQSGAPGAAGFPGPQGPPGQPGEPGTPGPAGESGLAGQDGRQGDPGERGYPGAPGEPGLTGLAGAEGKRGAPGIPGPPGPSGISGERGPPGPAGAPGQVGPAGAKGRDGERGADGERGVAGEPGTPGIPGPPGPVGQRGPIGNTGNEGKPGVQGPAGNSGADGRPGEQGQQGPPGAPGLAGPQGQPGESGPSGRDGESGPPGAQGPRGERGPAGEPGPIGGPGLPGPQGERGSPGPQGETGVQGLAGAAGAPGEPGRTGESGSAGPQGEPGVQGERGEQGFPGDLGPEGKRGPSGERGPQGPAGEPGPAGPPGPFGERGAPGPQGLVGLQGDRGPNGPPGTRGNRGPAGERGKDGAVGIPGEVGPAGLPGPAGPVSLIMPEKGDAGPPGTPGENGLKGDTGPQGYPGNPGPMGPQGAAGPPGVPGEVGSEGRQGPSGDTGPRGYPGEPGPGGETGKNGVDGAQGPPGEIGQQGPAGAPGSPGHPGPPGPQGPQGNTGFVGAPGKAGQRGDRGETGPPGPAGKDGEQGPPGLNGQVGERGNPGQQGPQGPPGPSGPQGERGSPGYPGGQGESGPAGEPGVQGSVGQPGDDGADGIPGPPGPPGPSGQPGFTGPNGEPGAPGLNGLPGAIGQSGDKGSRGPSGPPGLQGPPGPPGQSGPTGNAGPQGERGERGVAGEAGIPGAPGQQGATGATGPAGSPGETGRTGAKGDKGWPGMPGGQGLPGPQGPGGEKGPSGPPGPPGQPGSNGARGNPGRDGEPGPPGMPGRAGSRGPQGDDGLTGPSGPPGPPGPPGSPGYAPVWPGGNWQQQQNKGPDPLYYGDEPDKTPVINDDLSRIQEALHRTKRPSGKKHNPGVTCKDLLLQNPDFEDGWYYIDPNGGSFYDAVEVYCRMNIDGETCIPAGRKLYAEDQWTKVTKSQWFAKEILGGAEFDYKIDDIQLKMLQMHSTKARQRVTYKCMNSDPTGAVLMSNEFESLETLANKQEPYSAVKVEAFGNCDRTSNANRWGEMIFDVKSERSESLPLLDIRLKDVGQSNQEFALSLGEVCFNT | collagen pro alpha-chain [Haliotis discus] | BAA75668.1/0.0 | Signal peptide(1-22)/VWC(SM000214);Collagen (PF01391);Internal repeat ;COLFI(SM000038) |
| **Unigene6711** | 2.7547 | FEFSYFGTR | 1 | TDGFLAHNGRNNGMTYNIINYKLLTNKRLCIRFEFSYFGTRTPHHSCHQALLTTTATSKITKPSTVLTTTLRPRTTSSASDTSSHSTTKTSTTADTTTEKLITPTKSTTQRLTTEHQTWPTVSATTATTSVRSYTATSAIFTTTMQTTQNTQRTTAQQRRTTTTLPPTTTTLPTRPPMPDPSR | — | — | Thr(30.6%) |
| **Unigene6649** | 3.8972 | ATDLLENNFFR | 1 | MIKNCLSITIVLSLFPVLTYTQGDIGAAIADAVTEVGTRRPGGPALASAATGAGPPADVSGFGGVPGAGLAGVAAPGPADAATDPISIHALFSTRREAPGTVAAFGTAGDITTTATANFAARSGGALPDAGEFSRQIAPYCPVRRALCDPGAPYRTADGSCNNLNNPLWGASITNQARFIPPTYGDPSNIGTTPRINSVIGVPLPSPRIISNAVHRGGVSPPRSTIFNVALTHFGQFIDHDIISTPILRDGNEEIRCCTGAGVPVLRPECFSFFTPPGDFQTTCMHFVRSDFGPEPGCPPGPRNQINQRTSFLDLSVTYGNTRDGQNDLREFNTGRLLEGPGRILPEGPSGSECVGPSPCFKAGDNRPAEIPMLTVMHIVFLREHNDIVDGLRSMNPSWDDERLYQVAKKILTGIYQHIIYTEFLPIIIGQQGMDIFGLRSTPVGHSNSYNPTANPATRNAFGAAAYRFGHSLVGSFVAAYNIDLTPRATDLLENNFFRTDAIRNPALFGADGIGRWMTSQLKSRADRFLTPSVRDRLFQTAPGNGFDLSALNIQRGRDHGIPSYNRWRQFCGLPPALHFGTGPLGLINHDPSAAAALGSVYSHPNDIDLYAGALSERPAPGALVGPIFRCIIGLQFRLYKIGDRFFYENNFVPTGFTTAQLREIKSQTMSALYCRTIRIPSMPVNAFVSDRSGAPRIPCQFLRPLDLRPWRK | byssal peroxidase-like protein 1 [Mytilus coruscus] | ANN45955.1 /0.0 | Signal peptide(1-21)/An_peroxidase( PF03098) |
| **Unigene6643** | 2.2109 | VGGFQEVYLMEDFMLVESLR | 1 | FIKKVLGKSKTKELVIFSRYPCPGTTKTRLIPDLGTENAAYAQLKMTEHILDNIGMYYDTDNDVTTLMYYNGGTEKQMQHWLDKRKLDIAWQQQAKGGLGEKLANAFKDSFRRGSKYTVVIGSDIPGISSSIISEAYRKLQSGTNMVLGRAVDGGYYLIGLAERANIEFDKLFSDINWGTDTVFAEQSDKAKSLGISLDILSSVLRDVDIIEDIPVIEEELKITKQQLTNQKLSIVIPVYNEAKHIISTLENIITSCKNRDVLKEIIVSDGGSTDDTASIIKQFGKECDVPIKVINSHPGRGFQQKLGAEYASGDILLFVHADTKLPNHYDDAILKCLHTPGNIAGFFRFSLDSLECPERSKEVSFLFRLKMSWIRWSVLKRNLKFELPYGDQALFMEVGTYKKVGGFQEVYLMEDFMLVESLRKIGHIGIADCGPAITSSRRWERYGVFRISGMNSLIVLAYKFGVHPNKLAVWYYGDRIKRIKNRQKQD | Putative dolichol-phosphate mannosyltransferase [Crassostrea gigas] | EKC32092.1/3e-152 | DUF2064(PF09837);Glycos_transf_2(Glycos_transf_2) |
| **Unigene6218** | 2.4307 | FIVNYVR | 1 | EVKTTEKTGKVYYQIISKGSIVQTGEIIMGDATVMKAPIMITTEMAPKARFIVNYVRADGEIVTDGVTFSVDGTFKNNVAIRFSKDEALPGSRMLVDLEADAGSQVNVLAVDKSVLLLKSGNDISEDKITEELQSYDNAGGGGFYPVFARMWYWPSTGRDASDVFDNNGMVVLTDAMLYNYEDPFRPMWRRGGAIAFAGAPMPMMAVQNSPPEMAEMARPDTLKAVTKIRKIFPETWLWVNASVMDTGKTTLNVKVPDTITSWVATAFAVNQKSGLGLTPQPANLNVFMPFFVQLVLPYSVVRGELVVLQVNIHNYLPRNEWVLVILEQNMGMHNVITYSNGMRKLAHAKVGRWIKLQGGSIGSAFFPIKPQEVGQLKISVSAKTGSASDAVEKHLLVEPSGTPQEYNVPVLIDLKSVGTFQKKVNITFPKQTSPGSRRVAASVIGDLMGPSINGLDKLIKMPYGCGEQNMLNFAPNIFVRRYLSITNNLKSDMDAKSKEYMVKGYQRELTYQHKDGSFSAFGESDKSGTTWLTSFVVKSFAQAKQYIFIDDELVLKALEWLVKQQNETIGTFDEPGKVLHKAMQGGSAAGERSLTAFVLIAMKEADVIQGASDVTSRSVQKATNFLEGEVDKLIDTYEMAIVSYALKLVNSIKGDIILERLNALATVEDGLKYWEHDVQESKEQKFVSWNPPHSQSNAIDIELTSYILMNYALNKNINNGLPILRWLTTQRNPDGGFSSTQDTIIALQALAEFAGEIYSSDFNMRLTLQSSKGEPFMDSYTITPENALVLKTFDIPVGVEELTVIANGKGVALAEVAVYFHTDSDIKTSSFDINTTLSEETTNGFKLEVCGRYLRKGATGMSLMEIGIPSGMTPDYETLDFKKAPEYKRKEELFRKLVLYFDSFDKEPQCVSLYIIRTDRVAEQQPSPVRIYDYYEPSNQKTNFYTSSKLSDSSLCDVCGKECFCTN | CD109 antigen-like isoform X1 [Crassostrea virginica] | XP_022323515.1/0.0 | A2M_N_2(SM001359);A2M(SM001360);Thiol-ester_cl(PF10569); A2M_comp(PF07678);A2M_recep(SM001361) |
| **Unigene58165** | 3.8972 | EHTCGPNYVQCDDGLQCIHER | 1 | HVLIVRRALDQKLIIMHRFLQTSLYILVALGFVKAAGVCDSGYEKCGNSDQCIFYGLFCDGEVNCENGADENDAFCREHTCGPNYVQCDDGLQCIHERGYCNGKAMCKDKSDELPQNCQKFQCPDIAVKCASGDECTPWYELCDGESHEIEGCKDGSDEDVNLCRASTCPSGTQKCDNGLQCYHDLYACDGKNDCSDGTDEKPDMCRAFQCKMNRVKCADGLRCISWYQICDGKSNCIDQSDESTAICDGNQSLRRLKSLTVLPSA | EGF-like domain containing protein [Oryctes borbonicus] | KRT86894.1/1e-26 | LDLa（LDLa） |
| **Unigene5812** | 2.7547 | YLAEFATGQER | 1 | MAAEREENVYLAKLAEQAERYDEMVEAMTKVASLDIDLTVEERNLLSVAFKNVIGARRASWRIMSSLEQKEESKGESEKKEQMKQYKQKIEDELKNICGDVLKILDDHLIPQAGSGESKVFYYKMKGDYHRYLAEFATGQERKDAAENSLVAYKAASDTAQSDLAPTHPIRLGLALNFSVFYYEILNSPDRACRLAKAAFDEAISELDTLSEESYKDSTLIMQLLRDNLTLWTSDMQADDADQKQEQVQDLEGEDGS | AGAP001151-PA [Anopheles gambiae str. PEST] | XP_322009.2/7e-150 | 14_3_3 (SM000101) |
| **Unigene56734** | 2.4307 | QAVPTQR | 1 | SSEKNNAILELIRGNLTTKTNQETTVVLEENPIIILREEYKFVFPSVDTNAKSERYGNHKGKRVKRQAPGVIKSKDIHAKNIETDKMFARRVKADVVRANKVNAIVISIGKAFSMHRATNKGKPDTLVLNRDGPDPTDSAVTIVLERDGEPVSINGHPSDIEEKRKLITVKNSVVSKKNEKKSRKNKQPKQGKRKIVLPQIRNQVQFGKLGILNSKPRARLIIRQAVPTQRTRNTVFKPVIKPKPATKPKFVIKPKPVILRKPVNTKILATVESPMATRSLVNEVRKKQLLMEHWRKKIIDYQKRRKVKPLIVTRNKIVRPLRPPSSQVRLRGSLAKLVKPFVAIPVRLRPAIQPSFAKPLPKPIGMSLPLPQFRRQNMLIKPRRNTPIAKIKPLSKSDDLSDIESNDWDDDDKIDGRTKLPMKTFERVRPSFDDSDDDVWYDSEDYFYDSSYDDEAFSNEKSGQNLQRKNIHRKNGNHKNNFDEYSYNTEETSEYEYYSESDTDASQRHKDIKKVKVKTEQLKDDDKHSHRYNEKHSKGDGADLSEEYYSSEYEYYSDESDIETKRPTKSLDSIQSMTRREKIEHDTGDLKEKKIPNSITDKRDVQKQKINNFALSKRNDSKKSSKIIRVQKPKSSVVKMKTLISKSFDNFYTESDESDSVVSLENKDTLRKNFFTDSAEQWFNNKGDWDFTKDFDSFESSDKSLNEDFIPQNKLLFRLIK | — | — | Lys(13.3%) |
| **Unigene55011** | 2.8558 | FESDALLVPR | 1 | MGPLSNQAVFVFILAVTKALAANVDIPASKFVPTVAFQCGKPAMHKTAKGWVADKNTDCKKEKLDIFKYCKSIYKDLSITNIVEDHEVTINWPDKTGKTQLVKVTPYRCVVGRFESDALLVPRHCEFKHEHDTVCEGYQYWADIADKKCKGMNMKRNDFAMLLDCGIDKFNGVEYVCCPEESDPKEKPLDIPVELTPVKTKPQEILLGGDDNKEDAYIAYLRGDDSFLKKYENEHDKFLAAQSARDQRHHERITKMMKEWQEARDNVNKLKESNPKGAEKLNKEVMQRFQRLYSGYEQADRAEKKQLVALHMQHRQAELNKRKRNNLEKYMAELQRQPPVVHKIKKYLEAYIRVEEKDRMHTLNHFTHVRRNDPAEAQHMQKDTTEHLKVIDERIKQSLDMLHHYKKIEAEILPAINEFLSHYKTLSESALHVIMRPFTHDELKKSKTDDGFDNSETWDNPKKEMKMESKSSVVIDNQPAEEVREDSEDMEDEHKDFVAHHVNDKQSIQHQQFVSNSHHVVNSSTGSVFGIAIGSVAVFVIIIVAIIMLRRNKAHRHPVTHGFVEVDPAASPEERHVANMQMNGYENPTYKYFELNTNGSKK | amyloid beta A4 protein-like isoform X2 [Crassostrea virginica] | XP_022323552.1/0.0 | Signal peptide(1-21)/A4_EXTRA（SM000006）；APP_E2（PF12925）；APP_amyloid（PF10515） |
| **Unigene55006** | 2.7547 | IEFLEDTK | 1 | MNSLGTATLFLVVIWISVSEQGILPSRKNQVRSRRNYPERKPENIEKVLASGKENSGGGPASSGGQGNKHNVVIDQPPSNNGGLSNGNFGGHGAVVDDPTPNLGGPGNHDVSVADDPTPISGGGSPDKGNNNPESPKPEGGSPNGQGNNYDVSGPGDTAPNGLGNIHDAPVPDGNNNIAQPKDVGPQNGPGNNQDSPAASGGADPNGHGNNQDSPATSGGADPNGHGNNQDSPAATGGAETNGQGNNHDATIPASSGGGVQVDDHNTHIVGNAGGPVDANDHPLPRRTSRRRKKGRKGDKNENKEKRIEFLEDTKEMRDGAKATEDNEEDSEPQPVKPRKLRPKPTPAEKVINILTHNQPLPSSDPSTNQPSQQSESAASNPSPASGLVDNYPSPVSSHVNNNPSPSKPKEKARLFLFGDWSDCDSSDCWSSESSDSEKGSSSEKSTKVDSNIIESKLKFLRSRPKALIKMAAKDIDDISDKSSDCNSSDESDCEYWYSSEESVANDNGRRSSSKKNWNKKGSSESFPRGRKYGSRESYEKNSDGRDYSSDSSGWYDYESYEDDWSSEGRRNKHGDNRKTKTNGDSNNNDNNNDNNHENGNNNENNHDNGNAKNNHGDNGKGKRVERKRKLNFQDWDSYEYSDYSDSYESSDYKWHSKDSAEKTEKRNKEKKSKKGWYYDSSEYYSDSYDYESDDSGYYSDSSEWYENDDIFHKK | — | — | Signal peptide(1-19)/Internal repeat |
| **Unigene54892** | 3.8972 | LMDFFTQPESHFQNSR | 1 | QQQIISPNYLTDTQLLQNAFPNIGETNGLLQQSTAFQEQQPLFGASPSLKADPLQHLVSSPGIGNFQQDSRLMDFFTQPESHFQNSRHIKTILPRSQQAPQMLQTVPVGEKPLTPLNTRCPPDSPRVKCWHDPCKVTTCPLFREAICVKYQCGECQAKFTIDGVDITHYCHV | Deleted in malignant brain tumors 1 protein [Crassostrea gigas] | EKC18275.1/1e-05 | Gln(12.2%) |
| **Unigene54043** | 2.0978 | FASFIDK | 1 | RTQEKEQIKTLNNRFASFIDKVRFLEQQNKMLETKWNLLQGQTTTRSNIDAMFEAYIANLRRQLDSLGNDKMKLEADLHNMQGLVEDFKNKYEDEINKR | keratin 8 [Epinephelus coioides] | ACH73075.1/7e-66 | Filament (PF00038) |
| **Unigene53649** | 2.7547 | IILLAEGR | 1 | SEDMNGGGRNDSAGILAMKPNPGLKFEKKLSETERSTRDMKLVAKSGKKKKDVKFVLTPTKKRLLSRSISASSSTDSFDSTSYTGSSSDEDDVNPREKVQKNSKGSGDFCVRNIDHAAFGRREIEIAEQEMPGVIALRKRAEADKPLSGAKIIGCTHITAQTAVLIETLAALGASVRWAACNIYSTQNEVAAALAEAGYPIFAWKGETEEDFWWCIDKCINCEGWQPNMILDDGGDATHLMLKRYPAMFNMIKGIVEESVTGVHRLYQLSKGGKLTVPAMNVNDSVTKTKFDNLYSCRESVLDALKRTTDVMFGGKQILICGYGEVGKGCAAALKGLGCSVMVTEIDPICALQACMDGFRVVRLEEVIRSIDILITCTGNKNVVTRSHMDRLKTGCIICNMGHSNTEIDVGSLRTPDLTWEQVRSQVHHIIWPDGKRIILLAEGRLVNLSCSSVPSFVVSITAATQALALIELYNAPPGRYKQDVYLLPKKMDEYVASLHLPNFDAHLTELSDEQAKYLGLNKTGPFKPNYYRY | S-adenosylhomocysteine hydrolase-like protein 1 isoform X2 [Mizuhopecten yessoensis] | 0XP_021362456.1 /0.0 | AdoHcyase_NAD（SM000997） |
| **Unigene5318** | 3.8972 | DTTGTTSVNIDVPR | 1 | MEYITITLLIFSGRPAPECTIQVTDPDFQRITNMLPAPPGGNIVGGLGYQGYIVRDTTGTTSVNIDVPRGSNQALERELFEVCRFNPILKKYIAAFGRRGFNQEEGQGQLIKTTFTYDRKMDFITIVLLIFSGLPDPECTIPASYPNIRRITNMLPAPPGGNMVGGLGYKGYSVTITTIRADPVVSFNINVPRGSNRALERRLFKVCHFNRKLKKYIPAFGRRRKIG | — | — | Internal repeat |
| **Unigene51584** | 2.7547 | ACPSGTVFNAR | 1 | TTHLLENVLTGKPINDKQIVSTIQQPKQVADTAGNDVTTGDLKAEFQDLGITPSKKETGNNLLSREADHIGNLDQDPIQDVTETIQQTETSGLALESLCEHSSFIDSIGYAEYPGRCDKLVQCYNYRGRDIAVLRDCPYGYFWHQEQVLCRPPAEVPCYDDPCLDIDIVQYNRSGGCRSYYACEYGISIPQCCDKGFRFNGRRCIPDATCYDPCSTPFDIEQQMRYQPCKFLPDEYNPYGYITMEHNGLRIRACPSGTVFNARDCGCRSIGKGSPKYRQSRRRMRQCKPQFAMDFDQGFKEDSGSNMAFDVNNVVITNRAAQFDGKGKITLWGFMNRELGTRFAIRLRFKPDPTSTDIGSLISNCGMTGSPTVSIDLQKHMLVSSAKSKSFTEPTKIKDHFDPYSWNDITYVYDGRNFMVLTNGYKQRKPLGGLLETRSNAIVIGGCPRPGKGYKGLIDSVEIYSGCIPRDILKKRNRR | mucin-2-like [Crassostrea virginica] | XP_022335717.1/ 6e-134 | ChtBD2(SM000494); LamG(SM000282) |
| **Unigene5067** | 2.7547 | YDWDFFDR | 1 | MSGRLVPVHRYDWDFFDRQMSLFPSFKDDFDKDFFSDFKSTKIEDEIARMKREMFQLTSPEQSLKVDQPFVEDFTGNKKMALRFDCSKFKPEEIEVKTVDRSLTVHAKHEEKSPGRSVYREFTKSYTLPRDIDPLSLKSSLTNDGFLQVEAPAPKTCIARKEIFIPIEKMLK | small heat shock protein 22 [Mytilus galloprovincialis] | AEP02967.1 /2e-92 | HSP20(PF00011) |
| **Unigene49895** | 2.3052 | CLAVNYIPIIHLGSCR | 1 | MEYKTIRTFVTLFLTLGVIQAQECPDTSGIASACVIDPELHCFGDHQCRHGYKCCSYGCNRRCLAVNYIPIIHLGSCRNAGPLVNPKTCIIDSVCKSNEKCCSFRCAVVRPFIV | RecName: Full=Perlwapin-like protein; Flags: Precursor | P86855.1/7e-13 | Signal peptide(1-21)/ WAP（SM000217）；WAP（PF00095） |
| **Unigene49599** | 2.7547 | SPIAEAVFLR | 1 | MSSKKHSVLFICLGNICRSPIAEAVFLRLLKEKNQLQDWMVDSAAMGDWHVGKGPNERTVTTLHKFGIKDYVHKARMIQPADFEKFDYIFGMDHDNMSDLDSIKPKNCHAKLHMLGEFDPQKELIIVDPYYGHDMKDFDKVYEQCCRCCEGFFNSVTGS | acid phosphatase isoenzyme As [human, erythrocytes, Peptide, 157 aa] | AAB22516.1 /8e-53 | LMWPc（SM000226） |
| **Unigene49314** | 3.8972 | ADGDWSECDTALNIR | 1 | MKFQLALVLVALVVVTSAGRKHIDDAGCKFVKGEWSECDSSNQMKRTLTLREQNDGCPATKEQTRPCKDKKSKGCKYRADGDWSECDTALNIRTRKVVLHKGDSHCALEKTNEISCDKYQEIVRRDEERKKARERATAMKAEKKVLKHMQKKLKQLKKIGCQYVEEKSQCNNETQKVTLTFTFESGSDPSCVMEPVEVSCELHEKYEKIKQAKMEKKERKKKEKMERKKNKKLKDKKNKKERRKQRKSEG | pleiotrophin [Parasteatoda tepidariorum] | XP_015920374.1/6e-09 | Signal peptide(1-18)/PTN_MK_C（PF01091） |
| **Unigene48689** | 2.6077 | NLPDIPIHLTGR | 1 | MTGKKQNNTSPAINNSQPQHTASYKNFPISIGDHGKKFLPKPAVNIHALRNLPDIPIHLTGRAKTTLQELLQFAKPLDIVNPIKTRSKTLKNFRHKTVLAKKLSNIFRKQNRRRLHR | Transcriptional regulator, LuxR family [Granulibacter bethesdensis CGDNIH4] | AHJ65725.1 /8.6 | Lys(12.8%);Leu(10.3%) |
| **Unigene48220** | 2.7547 | AGNDVDLTIR | 1 | MASEISVVKLRRYDTGQPWGFKMQGGSEVGIPLQVAEVSPKSIAGKAGLVNGDLICYINKSYVEDVTHQHARNEMIRAGNDVDLTIRRPGGVVPQSAPPPQVEQESEDDKFRDVQPKTYQVLEKEVPHSAATGGRPASIFDKRKQARSAYTKTDKSGYMKAYGQPGN | PDZ and LIM domain protein Zasp-like [Crassostrea virginica] | XP_022337598.1/6e-67 | PDZ(SM000228) |
| **Unigene46594** | 2.7547 | GPAVQNVQGNR | 1 | VAQLDIWALTEQLKTLIKNEVRKSVSLAIQDSVQTIVENKLAIVEESLQSRINSSLSDAVSELNRRPMFLAFLRADLTSFQGDDVLKFDDVRINKGGLYDATTGLFTAPKTGDYQVSFLLMGRQEYDVTFQLQKNNDLYYHGYAAKSNYGSQGTTLLMKLKPGDQVYIRHRGPAVQNVQGNRHSFFSARLL | putative C1q domain containing protein MgC1q19 [Mytilus galloprovincialis] | CBX41668.1/9e-41 | C1Q(SM000110) |
| **Unigene46536** | 2.0391 | YASICQQNGIVPIVEPEILPDGEHDLK | 1 | DSCIGGVIFFHETLYQNTDDGTPFAKLIKDRGIVVGIKVDKGVVPLAGTNGETTTQGLDGLSERCAQYKKDGADFAKWRCVLKISDTTPSELAIFENANVLARYASICQQNGIVPIVEPEILPDGEHDLKRCQYVSEKVLAAVYKALSDHHVYLEGTLLKPNMVTAGHSCPTKYSSEEIAMATVTALRRTVPPAVTGVTFLSGGQSEEEASMNLNAINNCPLPKPWALTFSYGRALQASALNAWRGELSNEKAATEEFIKRAEANGLAALGKYESSGPAGAAGKSLYVANHAY | fructose-bisphosphate aldolase C [Cynoglossus semilaevis] | XP_008330936.1/0.0 | Glycolytic(PF00274) |
| **Unigene46263** | 2.7547 | TTITTVDSGSSR | 1 | NCPASRSERICNTQPDQCRECEVKGRRYRGNSRFSYDEDCYRYNCDCNCNGSWNCPASRTQNICGSGQDQQIGESRSTVTTSNSGSITRTTTTGSGCRECEVKGRRYSGNSRFSYDDGCYRFNCDCNCDGSWNCPASRTQDICRSGQGQNTGGSQTIVTTLESGGSTRTTSTGTGCRHCEVKGRRYRGNSRFSYDEGCSRFNCDCRCDGSWNCPASRTQNICESGQRRQTGGSRTTITTVDSGSSRTTGTGCNECEVKGRRYRGNSRFSYDEGCYRFNCDCNCDGSYNCPSSRTQNLCGSGQGVRTGTRTTLTTSTKTTGSGESAIGNGVDSRRTGSSQISGSRRSTGSGVSLTESRRTGGGLSRAEKTFTETRTSTSSRSTSSKTVVGGSQTKTELRSRSGSTTGSAVDSYGCHYCIVNKIQHRGNSNFQFEDNCFRFSCECRCDGSWLCPADRTINICPDRCQNCNVNGKSYPSNTIFEYDEGCNRFNCDCACDGSFNCPAERTKDICSGRQDKCKECNVNGRRYQPNTQFSYDERCYRYICDCNCDGSYRCPAERTKNICGSLRADTCQECRVNGKNYEPKKTFSYIDGCALRKCTCDCSGRHRCTATTDICKSSSQVCRDCVVKEKRYLPNEPFKYTEGCDRFNCDCNCDGSWTCPAERTENLCAIGQCQKCSVRGEVKEGNSGFVVTENCRQYKCWCHCDGNWSCSKDFTRSCNLG | Kielin/chordin-like protein [Crassostrea gigas] | EKC25979.1/0.003 | Internal repeat |
| **Unigene4478** | 3.8972 | QCYFGSSCFGPAFQR | 1 | NNQETVKYGIQHRERKHISLEMQFNKDIFLLLVLCLHVRYCETVIIGEWTQWTSWTSCSVICGQGTQDRIRQCYFGSSCFGPAFQRRTCISKPCSRLPQWSAWSAWTECPTSCEHRDRIKLRSRTCSEPGRCRGDGLQTRLCPYCPIRSTRPSIPQWSAWSEWTDCPASCEHEDRIKFRSRTCSQQGRCRGNGIQIRPCPRCSIPPNRPINSQWSIWSQWSPCSESCGYGSKERTRQCSGRYCPGESNERRTCFERICPTDCTDTRRDCPGLVSLYGCTYGLWNYNNCRKSCNRC | coadhesin-like isoform X2 [Orbicella faveolata] | XP_020632436.1/1e-30 | TSP1（SM000209） |
| **Unigene44594** | 2.2570 | DPQIFVGR | 1 | MTEWVKTSANNIPNGSIRGGYDKNGHTLFIARALTDDGFYSAGKASLHYEDGAHIPYRGQEIIVYEYEILVLPSQADGFYDWKPTASANVPSNAVPSDINRDPQIFVGRFVHEGCLIPGKVDKKKMKCYIAHNGKEYPNDHYEVLVKVK | Chain A, Crystal structure of a DM9 domain containing protein from Crassostrea gigas | 5MH0_A /4e-32 | DM9/SM000696 |
| **Unigene44069** | 2.7547 | ESLPLIVFLR | 1 | MGYRGRRKHLKRLTAPKSWMLDKLGGVFAPRPSSGPHKLRESLPLIVFLRNRLKYALTHDEVKKIVMQRLIKVDGKVRTDMGFPAGFMDVVTIDKTSENFRLLYDVKGRFAVHRIKPEEAKYKLCRVKKLGVALKGVPYVITHDGRTIRYPDPLVKVNDSIMVDIATGKIKDFIKFDSGNLCMITGGHNLGRVGVIQHRERHPGSFDIVHIKDSLGHTFATRLSYVFVIGKGNKPYVSLPRGKGVKLSIAEERDRRIAARS | 40S ribosomal protein S4-like [Crassostrea virginica] | XP_022290789.1 /4e-172 | RS4NT(PF08071);S4(SM000363);KOW(PF00467);40S_S4_C (PF16121) |
| **Unigene44042** | 3.8972 | EECGWSGISQLTCEAR | 1 | QNLFVRQGVSNNQSVGPRNIFEDKMKLFAVLLVASVSYGMTLGNDETCEVQPPYRKECGWVGISQETCEARGCCFNSSTSGVKWCFQKAKLFSDNPECAVQPSNREECGWSGISQLTCEARGCCFDSSMSGTKWCFQKEKEFSNDTNCDVAPIFREECGWVGISLQTCEASGCCFDSSISSAKWCFQTAKPECWSLPNERRECGWPGISKRTCEARGCCFNSNTENTKWCFHKKQ | putative gastrointestinal growth factor xP4 isoform X1 [Stylophora pistillata] | XP_022785025.1/3e-45 | PD(SM000018) |
| **Unigene44023** | 2.7547 | VLITTDLLAR | 1 | RYFSENMSYNPEDGDRSRNTNEGPEQKSNEEYSAEAGGVIQSNWDEVVDNFDDMRLKEELLRGIYAYGFEKPSAIQQRAIIPCIKGRDVIAQAQSGTGKTATFSIAILQQLDISQKDCQALVLAPTRELAMQIQKVVIALGDYMGAQCHACIGGTNVREDMHKLSTGVHIVVGTPGRVHDMINRRALNPRAIKLFVLDEADEMLSRGFKDQIYDVFRYMPEDIQVILLSATMPNEVLDVTQKFMRDPIRILVKKEELTLEGIRQFYIQVEREEWKLDTLCDLYETLTITQAVIFCNTRRKVDWLTDKMLSRDFTVSAMHGDMDQKERDVIMREFRTGSSRVLITTDLLARGIDVQQVSLVINYDLPANRENYIHRIGRGGRFGRKGVAINFVTQEDIRTLKDIEQFYNTQIEEMPMDVADLI | eukaryotic initiation factor 4A-I-like [Crassostrea virginica] | XP_022288944.1/0.0 | DEXDc(SM000487);HELICc(SM000490) |
| **Unigene43648** | 2.4997 | SVQLTASNFGEALGVGR | 1 | MEITVQQRSKEWFNLRKSVQLTASNFGEALGVGRGKPYDFLQYYIDDEEEEETSPAMQNGIDMEVVISEMYQLLTGNQIRESGFWVPVQGDELENLVGVSPDAIVEGKKGQQLGLCEFKAPVFSLYGQNTPHGIPRHYMAQIQGQMGVSKLPWCDFLAVCVRTKSLILKRVHFCETYWKTAAKLLKEFCLAVKASRLAGSIPDNNAFVNVIKQRETFFSKFITGESSIRVENLLKVNVKNNTYCGPSDVWMTFDCLMGTDLDLSPETLSFIQDKIDQIDKEIQLKQTVTV | Restriction endonuclease, type II-like superfamily protein [Arabidopsis thaliana] | NP_001077786.1/3e-12 | YqaJ (PF09588) |
| **Unigene43496** | 2.7547 | IECGYNQETR | 1 | MVLYDLKTFVLMTLCIGASQSADWDVFTFTQEWPTTVCVEGKQEHHRCVIPTGVSTWSIHGLWPTKYGTEGPTDCRNVPFNPNFIKNITGELSKFWANMYADTSDTSFWEHEWSKHGTCAMDLPATASEYLYFRKALDVMKTLDATMLLNKKNIVPSDTRIYNVSQFEEAIKDQIGVTPRIECGYNQETRKHLVYEVEICLSKEFKPVSCDHSKRYVRHHHHYSSNGSSDRTNCPHDGFLYPPIPPSDDVIG | ribonuclease Oy-like [Crassostrea virginica] | XP_022333543.1/2e-83 | Signal peptide(1-21)/Ribonuclease_T2 （PF004450) |
| **Unigene42892** | 2.8558 | AYPGTETNPTVNVR | 1 | MCLVMKLFLGKMLFVLWIYRGGAVKVIEDDVFNTIFNSYEKRAYPGTETNPTVNVRHGLSIIRIDDYNADQNKLVLNVWLHNYWTDPRLNWTDKPDHVNVPITDIWTPDITLYNGKMEDFMPQQKANIVRDGSIMFITPTILETRSCESKGDNSIICSFKFGSWVYNGLQLNLTKNHAFVDLNDFEESDDFLLENATQTRNEMYYTCCFEPFVDITYTVHLTKKRGQLGSWFGI | neuronal alpha-2 acetylcholine receptor subunit [Mytilus galloprovincialis] | OPL21079.1/1e-38 | Signal peptide(1-23)/Neur_chan_LBD（PF02931） |
| **Unigene42863** | 3.8972 | ASANAAAFLQWIPAVGWR | 1 | MRQLVLVALLGFVTNTYAGCSFKASANAAAFLQWIPAVGWRSFSCAPGLFFNPGLCCCAPFGVGASAAASASAAAGAGGSAAAAAAAAAAAAAAAGLGVGAGAGALAGAGALAGAGALAGAGALA | — | — | Signal peptide(1-18)/ SCOP d1gkub1 |
| **Unigene42007** | 2.0391 | AELNNFLTR | 1 | LHQQQTSFSFKIAVNMANQISKKRKFVADGVFRAELNNFLTRELAEDGYSGVEVRVTPTRTEIIILATRTQNVLGEKGRRIRELTSVVQKRFNFPEGTVELYAEKVAQRGLCAIAQCESLRYKLIGGLAVRRACYGVLRFIMESGAKGCEVVVSGKLRGQRAKSMKFVDGLMIHSGDPINEYVDTAVRHVLLRQGVLGIKVKIMLPWDPSGKIGPKRPLPDHVSIVEPKDEVVPARPFSEQKGSKPAEAGQTPVA | 40S ribosomal protein S3 [Crassostrea virginica] | XP_022326183.1/4e-163 | KH(SM000322) |
| **Unigene4173** | 2.2109 | FEFDDIHR | 1 | MMIVILFLLLQAFLGSTAPSDDSCGISGRWRNDLGASMIFTCEEGNVVPTEGYLTGRFEFDDIHRYDLSGRYTMVGGDVVLGFSVAIGNDDGDSSVASLTGIHYTTEDSIHSKWLLSRSTEYNDMWKNTHFGSSVFIRVK | tamavidin1 [Pleurotus cornucopiae] | BAH20870.1/8e-10 | Signal peptide(1-17)/Avidin(PF01382) |
| **Unigene40538** | 3.8972 | QEQPVGLFGLGLGGLGGGR | 1 | MKTFVVVALVLCVMVAHVRSQGHSLDLFGMGMGNNMGNPGGGIGGSVSASTGAGAANSGSDAMFMKMMKLILLKLNKLEKQQRPPKQEQPVGLFGLGLGGLGGGRRGNSLHHHLLLEHLSK | — | — | Signal peptide(1-20)/Gly(18.2%);Leu(14.9%) |
| **Unigene3946** | 2.8558 | YYVTIIDAPGHR | 1 | MGKEKIHINIVVIGHVDSGKSTSTGHLIYKCGGIDKRTIEKFEKEAAEMGKGSFKYAWVLDKLKAERERGITIDIALWKFETNKYYVTIIDAPGHRDFIKNMITGTSQADCAVLIVAAGTGEFEAGISTNGQTREHALLAFTLGVKQMIVGVNKMDSTEPPYSEARFSEIQKEVTTYLKKIGYNPKAVAFVPISGWHGDNMIEASEKMSWYKGWAVERKEGNASGKTLFDALDAILPPKRPTDKPLRLPLQDVYKIGGIGTVPVGRVETGIIKPGMVVTFAPSNLTTEVKSVEMHHESMPEALPGDNVGFNVKNVSVKEIKRGNVCGDSKNDPPKGAKSFIAQVIILNHPGEIKNGYAPVLDCHTAHIACKFVEIKEKIDRRSGKSLEEFPKFIKSGDAGIVNMTPSKPMCVETFSTYAPLGRFAVRDMRQTVAVGVIKEVTKAEASGGKVTKAAQKAGKK | elongation factor 1 alpha [Mytilus galloprovincialis] | BAD35019.1/0.0 | GTP_EFTU (PF00009);GTP_EFTU_D2 (PF03144);GTP_EFTU_D3(PF03143) |
| **Unigene39231** | 2.7547 | HYGALQGLNK | 1 | MAKYTIVFVRHGESEWNQKNLFCGWHDADLSDTGLAEAKNAGKMLKEKGYTFDIAFTSLLKRAIKTLFFVQDELDLHWIPVVRHWRLNERHYGALQGLNKSETAAKYGEAQVKIWRRSYDTPPPALEKSDERWSAKEAKYGNIDESIVPPCECLKDTVARALPFWHDAIIPAMKAGKRVIVSAHGNSLRAIVKYLDNIPDADISELNIPTGIPLVYELDADMKPVKHYYLADEAQVKAAMEKVANQGKAK | phosphoglycerate mutase [Hymenolepis microstoma] | CDS33978.1/8e-130 | PGAM(SM000855) |
| **Unigene3878** | 2.7547 | FVPGWVFAIR | 1 | MELTVSVECLLLIVCLPMIHGMGLSYNYFDQMAQTYCQGRFVPGWVFAIRRDCGGTAPTCNDMCATAKLDILKTIKFQRKNVACFDAFRVKKDHVQLAANPTDTEPDAGKVNMVTYGYGTGGCRWRPNHCGPNYCCCKAY | Atrial natriuretic peptide receptor A [Crassostrea gigas] | EKC19009.1/1e-06 | Signal peptide(1-21) |
| **Unigene38586** | 2.3637 | SSSEVPWGFR | 1 | METIWLQRSSSEVPWGFRLQGGREFAQPLSIQKVTPGSVAGNVLVPGDILLKIGNNDVTNVSHNQGQDLIRYSGDLLQLTIKRCPRVAASAPVSPTPISQYNYQHSESPSSFRSLSPISDNFPMYQQGYPYQPRMYQEDRESPRQFASLPR | PDZ and LIM domain protein Zasp [Mizuhopecten yessoensis] | OWF52989.1 /1e-46 | PDZ(SM000228) |
| **Unigene3849** | 2.8558 | TFLGSVSDYVVQHAR | 1 | LHSLSKFVSHIILFFVKVHIKMAEGSSRIVMIAVDASKHSDEAFDWYLNNMARDSDDVRVVHCAEYNIDLGFGLNYDEKQIQQITQQVKEQQEKVDQLKSSFVDKLRGKGPGDESFRIKGEAFVITGKKPGEAIIAEAEKQSANLIIMGTRGLGKIRRTFLGSVSDYVVQHARCPVVVVRH | universal stress protein PHOS32-like [Crassostrea virginica] | XP_022298339.1 /2e-42 | Usp（PF00582） |
| **Unigene3834** | 2.6077 | GVLMVGPPGTGK | 1 | MSSATLLGEIDENTKLGRENALLGNYDTSLVYYQGVLQQIQKLISTISEADRKRKWMQARDLISQEHDQIKEISETLSSFKSNNPKPYPDNDFGSPFGDYARHEEPTRDPDVWPPPTPVEYRPSPNIRGGRPAPKKVEPVRRPGGPSKAAPSRQPDRGRPGAPGYGRDNRGRDNKDNKKKNDDGEKKFDPTGYDKGLVEGLERDIIQKNPNVNWDDIADLTEAKKLLQEAVVLPLVIPDFFKGIRRPWRGVLMVGPPGTGKTMLAKAVATECGTTFFNVSSSTLTSKYRGESEQLVRLLFEMARFYAPSTIFIDEIDSICSKRGSDSEHEASRRVKSELLIQMDGMYEGVGGSADDSEEQKIVMVLAATNFPWDLDEALRRRLEKRIYIPLPTAVGREELLKINLKGLEIAKDVKLSQLAETLEGYSGADITNVCRDAAMMSFRRRISGLTPEQVRNIPKEELEIPPNMEDFEMAIKKVNKSVSAQDLEKYQNWMKEFGSV | Katanin p60 ATPase-containing subunit A-like 1 [Mizuhopecten yessoensis] | OWF43912.1/0.0 | PDB 2RPA\|A；AAA（SM000382）；Vps4_C（PF09336） |
| **Unigene37375** | 2.7547 | NWLCLVYHESR | 1 | GVPNKDLRNWLCLVYHESRFHYDAINHQNADGSKDHGIFQLNSRYFCDRPQGTSESTCWKVNTYGCADSCASFRNRDINNDASCAVRIQRCGGFKKWVAWRKHCSNVSSPQYSYRGC | C-type lysozyme 3 [Mytilus galloprovincialis] | AJQ21538.1/8e-54 | LYZ1（SM000263） |
| **Unigene36836** | 2.7547 | GSQNTIETVR | 1 | MLLEIQRTVILFVLLLSYICAQEVAKVGGKIKVESLDPFAKPGVDPFASIRSAGGSGAQKQGATILTKKETVRTVTTNNKPVVDGSDVRSSQAGTSKNTQTTNEKPYVETTTIERKTRVIGSGSGTLRKSSNTNAQNIASKTETTKTQRTVNVVNRGTGNTGTQSRSGSVQTTGSRSQSSTTRTVQTTNTGSRTGSGERNTDLPRQTGGNRGSGQNIISVSSQTDRQLGQIGSRGSQNTIETVRRNDNVGPFGSSGGSNTGIYTNDGTMKIDTNRGNALPEMGGEMGATDKPILYFRKLYHDQSICLNCIGSGSDCYTEHPDDCRKYIRCTTSSLGGRMEALEMDCAFGTFWSNEANTCDTASEVFCNKDPCMDTSLKTYHSGLNCRSYWRCVNGRSNAECCDNGQEFVDGHCIPSDRCTIDDGECPFKSQFLDTIPEKKPTCNLEPVPEKGEKAFRMNNHWALMYCADGTKFDMKTCGCIVSIFNKVRCVPTVDIGFDEDRVIDRTRGYIKSENVTVTGGKGSAYALFQGESEINVPYFKNNENIKSHLVVSVRFFKNDDKGPGSQVLASNCKTSNKRTLTPSFAILLNKKEKQIVFFASTKKNDKYGATQHITLPFTPGKWTTATLKFLDETLQGTVETLEADGTPKIQKEEKPLKGTLNTGIMPLMIGTCNMNDGFFGYIHKAKAYMCKPDQP | Follistatin-related protein 4 [Crassostrea gigas] | EKC18454.1 /9e-43 | Signal peptide(1-21)/ChtBD2 （SM000494） |
| **Unigene34861** | 2.6077 | IGVFGENESTEGLSFTGR | 1 | MAGKNAESAIEALKEYEPEIAKVVRKNHRGIQRIRASNLVPGDIVEVSVGDKVPADIRISTIHSTTLRIDQSILTGESVSVIKHTDPIPDPRAVNQDKKNVLFSGTNIAAGKCRGIVIGTGLNTEIGKIRDEMMDTETEKTPLQQKLDEFGQQLSKVITIICIAVWAINIGHFNDPAHGGSWVKGAIYYFKIAVALAVAAIPEGLPAVITTCLALGTRRMAKKNAIVRSLPSVETLGCTSVICSDKTGTLTTNQMSVCRMFTFAKIEGNDVKTDQFEITGSTYAPEGDIYKDGKKVLSGEFPGLEELATVCVMCNDSSVDYNDTKNIYEKVGEATETALTVLVEKMNYYNTDKTGLNKREKGTACNHVISQMWKKEFTLEFSRDRKSMSVYCSPNKPTRTAQGCKMFAKGAPEGILDRCTHVRVGANKVPMSPAIKNEIMKHVASYGTGRDTLRCLALATIDNPPRREDMDLEDSRKFIEYETNMTFVGVVGMLDPPRTEVMDSIKRCRDAGIRVIVITGDNKATAEAICRRIGVFGENESTEGLSFTGREFDDLSIEEQRRACMKARLFARVEPTHKSKIVEYLQGEGEVSAMTGDGVNDAPALKKAEIGIAMGSGTAVAKTASEMVLADDNFASIVAAVEEGRAIYNNMKQFIRYLISSNIGEVVCIFLTAALGIPEALIPVQLLWVNLVTDGLPATALGFNPPDLDIMKKQPRSTKDSLISGWLFFRYMAIGIYVGCATVGAAAWWFMVYDHGPKLNYYQLTHHSQCLAQDQRFQGINCNIFDSPEPMTMALSVLVVVEMLNALNSLSENQSLLAMPPWSNPWLLGAIALSMSLHFLILYTEVMSTIFQITPLNFAEWSAVLKISIPVIILDETLKFVARKFTDDYPNFF | sarco/endoplasmic reticulum calcium ATPase isoform A [Pinctada fucata] | ABS19815.1/0.0 | E1-E2_ATPase(PF00122);HAD (PF12710);Cation_ATPase_C(PF00689) |
| **Unigene34096** | 2.7547 | TDVLLDINR | 1 | TTNWIDGLHQQSGNFNLYLAGNEFSCVCDYKSFIDWLSRTDVLLDINRNYSCTFPNGTRIRIPEVIQNYHRIFSHCNAIAWLRTGVICIVSSFFVIGLTAIIYQFRWRFTYFMYRQLKSRYIKEDPFVFDFVYDVFVAYANDCSEWLVESLIPTLEQEWNLNVCIKDRDFPIGADRGDTVVQS | toll-like receptor d [Mytilus galloprovincialis] | AFU48616.1 /1e-64 | LRRCT(SM000082);SCOP d1fyva_ |
| **Unigene34064** | 2.7547 | AVLVDLEPGTMDSVR;FPGQLNADLR;LHFFMPGFAPLTSR | 1 | RKKIKMREIVHLQAGQCGNQIGAKFWEVISDEHGIDPTGTYHGDSDLQLDRINVYYNEASGGKYVPRAVLVDLEPGTMDSVRSGPFGQIFRPDNFVFGQSGAGNNWAKGHYTEGAELVDSVLDVVRKEAESCDCLQGFQLTHSLGGGTGSGMGTLLISKIREEYPDRIMNTFSVVPSPKVSDTVVEPYNATLSVHQLVENTDETYCIDNEALYDICFRTLKLTTPSYGDLNHLVSATMSGVTTCLRFPGQLNADLRKLAVNMVPFPRLHFFMPGFAPLTSRGSQQYRSLTVPELTQQMFDA | tubulin, beta, 2 [Danio rerio] | NP_001032487.2/0.0 | Tubulin(SM000864);Tubulin_C (PF03953) |
| **Unigene32384** | 2.7547 | EVVQNSAFVER | 1 | MPEPETTMDEGEVETFAFQAEIAQLMSLIINTFYSNKEIFLRELISNSSDALDKIRYESLTDPSKLDSGKDLEIRIIPDKNNNTLTIIDTGIGMTKADLVNNLGTIAKSGTKAFMEALQAGADISMIGQFGVGFYSAYLIADKVIVQTKHNDDEEYIWESSAGGSFTVKPATGEPIGRGTKIVLCIKEDQAEYLEEKRIKEVVKKHSQFIGYPIKLLVEKERDKEVSDDEEEEKKEEEDEEKKDDKPKVEDLEEDDDEDKNKDKKKKKKIKEKYTEDEELNKTKPIWTRNPDDITQEEYGEFYKSLTNDWEDHLAVKHFSVEGQLEFRALLFVPRRAPFDMFENKKKKNNIKLYVRRVFIMDNCEELIPEYLNFVKGVVDSEDLPLNISREMLQQSKILKVIRKNLVKKCLELFDDISEDKDNYKKFYEHFAKNLKLGIHEDSTNRKKIAGYLRYHTSSSGEEMASLKEYVSRMKDNQKYIYYITGESREVVQNSAFVERLRKRGLEVIYMVDPIDEYAVQQLKEYDGKNLVSVTKEGLELPEDEEEKKKFEEEKAKYEGLCKVMKDILDKKVEKVVVSNRLVTSPCCIVTSQYGWSANMERIMKAQALRDTSTMGYMAAKKHLEINPDHSIVKSLKEKADADKNDKAVKDLVLLLFETSLLASGFSLEEPGLHANRIHRMIKLGLGIDEEEAPVEEATTEEMPPLEGDEDDASRMEEVD | HSP90 [Mytilus coruscus] | ALL27016.1 /0.0 | HATPase_c(SM000387);HSP90(PF00183) |
| **Unigene31729** | 2.7547 | SVGIHLFCVGVGR | 1 | SGRQKTAQIGVIVTDGRSKYPDASRGESEALRSVGIHLFCVGVGRLVDEKQLRSMAFKPNEYFYIEPIDLAVDKLVQSVRKLTSASTAKIPASADHVGMPASEISPKFNALPTKTTIATTITTTKSTTMKTTTTTRK | sushi-like protein [Mytilus coruscus] | AKS48157.1/2e-37 | VWA(PF00092) |
| **Unigene31394** | 2.7547 | ALNELFPDWK | 1 | GCKTVSRIVSVCKHVRLYSASPKITTHYSVHPRENDERWKDVDMTRYEDEADVLIVGGGPAGLSAACRLKQLANEHGKELRVCLVEKAPEIGAHTLSGACIEPKALNELFPDWKEKGAPLNTLVTEDKFSLLTEKSAIPIPVFSGMPMANHGNYIVRLGNVVRWLGQQAEELGVEIYPGYAASEVLFHEDGSVKGIATNDVGIHKDGSPKETFERGMELHAKVTIFGEGCHGCLAKQLYKKFDLRKDCEPQSYGIGLKELWEIDPEKHRPGRVEHTIGWPFDSHTYGGTFLYHLDEGPLVTLGMVVGLDYQNPYISPFREFQRFKHHPSIESTLSGGKRIGYGARALNEGGFQSLPKLTFPGGCLAGCSPGFMNVPKIKGTHNAMKSGMIAAESVFETLHNETLVSNSPTAGLNPEMYEEQIRKSWIWDELYSVRNVRPSFNTRFGLYGGLAYTGLFYVLLRGKEPWTLSHGGADHTKLKPAAECTPIEYPKADGKISFDLLESVALTGTNHDHDQPAHLTLLNDDTPANLNLSVYNGPEQRFCPAGVYEYIETEDGSGKRLQINAQNCIHCKTCDIKDPSQNINWVCPQGGEGPAYNGM | electron transfer flavoprotein-ubiquinone oxidoreductase, mitochondrial-like [Mizuhopecten yessoensis] | XP_021375935.1/0.0 | NAD_binding_8 (PF13450)；ETF_QO （PF05187） |
| **Unigene31163** | 3.8972 | TQLILSSVLTEGR | 1 | LWFPVLLYINPCISSIIYPRSTIDMDPKITLYKDKDFKGRTVTFTESIPNFVNVGFNDTLSSVDVQGGVWIFFSDINYQGNIYVVKEGDRVNVSGYNDKASSVKLINYDFSDPPSCTLYADSNYWGRSLNLTADALNLKWYDFNDRVSSIRVQSGAWVAYTDRDFGGKQSFYLQGGHSLSASEGSFPNDSISSMRAIQTKPSGPITILKFDFDLSRAIITNKPSVVFQWTQINDSSVEQNLSITTEKSITKDNTYEFHWKQGTSITASMEMSVGIPVLSEGKLTMSATASYEVGSKTGTRTSKTEKWVVKFPSRIPARTQLILSSVLTEGRYDVPYIAVLKQGNKTWTEQGVFRGVNYYGFVTDFKEEPL | gamma-crystallin N-like [Acanthaster planci] | XP_022092298.1/3e-74 | XTALbg(SM000247); ETX_MTX2(PF03318) |
| **Unigene29213** | 2.1667 | LPEDVTCR | 1 | MQLILLVCIIYLFREVDSHGRLIDPPSRSSMWRYGFNNPANYNDNQLYCGGVQVQYEVSGGKCGVCGDPWNGSRENEEGGKYSNGIIVRSYDQGEVISVSVQLTASHKGYFEFKICKKDNPRKRTTQECLDKYPLQLEDGSGTRYNIPNKSGYQLITVKLRLPEDVTCRDCTFQWKYNAGNSWGADSSSGRNCLGCGNQEQFYGCADIAIGYDDIDEGQSLLDGQNYKKKNFVNTPPPPPVFDEEMNNINNQDYDENWATVEQSPNTNFWEALVGQAKTTENTETLLEEFKRFKKTFANRYVRPCVCHSCVGNTCVCECLPLSHGTSYIIDGVSSWLVYFLGFLSCLVPLVLL | cell wall integrity and stress response component 4-like [Lingula anatina] | XP_013418191.1/6e-73 | Signal peptide(1-18)/Chitin_bind_3(PF03067) |
| **Unigene28664** | 2.3052 | YTSLLQR | 1 | EARKREFMQGRYTSLLQRQRDGRGSLHLVVALINAGADIDGSQYQPITVAAEADLADTVYMLINYGAWIPINWKFLSPDVLFGENMAQILDRIKSHVEVQITLLFQCKRKIRKLLAHTGQIESSINKLPIP | kinase D-interacting substrate of 220 kDa isoform X2 [Orussus abietinus] | XP_012282603.1/2.4 | SCOP d1sw6a_ |
| **Unigene27989** | 2.8558 | SGFGAAGFGVGGGR | 1 | MGSGKGMGNGGRGMHMEHGMGSGGDGMGMGNGGVGGSLNVWDHLSSNTGSSGSGSGRNPANNGRSGFGAAGFGVGGGRTLSNMGGSMHKNGGMGSGLASHGTNVWNRVNGQSNNKPMSNSGSVLSGPGGARNNGVANMNNGGTPLVDQGTKSKPVEHPPTPPASMSSVGGGFGRASDISFHTMGSMLQPNQGGSVSQPQSKNQGQTQPKQPGNATPQVSNQNPANSGSSGGGVGVNPQTNQPATNSQNSGPTGNNIPGINNGGSQGGNVGGSHGGNMGGSQGGNMGGSPQIETTGGATSAPNHNFGQGNSFFGHGTNNQGNLGTNNVVGTTAYPGFTSMATWMFLK | — | — | Signal peptide(1-36)/Gly(25.1%);Asn(13.0%);Ser(12.1%) |
| **Unigene27944** | 3.8972 | TELEEIAAENPGR | 1 | HIFVTVAANRSMGRALTVGLPVAIGLGVVAVTAILAKIFLFGNKKKKSPVTLEDPEKKFPLKLIDKEEVSPDTRRFRFALPSPEHILGLPIGQHIYLTARIDGQLVIRPYTPVSSDDNKGFMDLVVKVYFKNVHPKFPDGGKMSQYLENMDIGDFIDVRGPSGLCVYDGQGVFKIKPDKKSEPETVMAKKLGMIAGGTGITPMLQLIRACFKDRNDTTQIFLLFANQTENDILLRTELEEIAAENPGRFKLWFTLDRPEDDWKYSKGFISADMIKDHLPPPGDDTLILMCGPPAMINFACLPNLDKLGYTPKMRFAY | NADH-cytochrome b5 reductase 3-like isoform X1 [Crassostrea virginica] | XP_022332541.1 /1e-178 | FAD_binding_6(PF00970); NAD_binding_(PF00175) |
| **Unigene2791** | 2.4307 | GVNSLIPWR | 1 | KALLIIAFVLPVCEGFILQKNNQNVNRGVNSLIPWRRIGTADVNPSACPDYQQLCFHNKSYCKLVCQSCQKHECVNVKNTDETTCPCECFQNCEPSKSMRRRPLIGRMSVADELGDDHFAYYVGDEDS | — | — | — |
| **Unigene27815** | 3.8972 | APLIADLDISHLAGQIR | 1 | MLNLSCGGVVWLIVCLILECKTGNFVSSADAPLLTNLRAPLIADLDISHLAGQIRNLIKEEVQNAVSGLNISSISGLKDNPAFFVTLQQHPFTLHGIDDVVKFDGIKVNIGGGYNRQTGYFVCPKAGVYHFSAMILGNHGHVVHYQLNKNNSPYVIGYSKMGTADSSTVSVVAKLEVGDRIFIKHRYRNEVEQIFGLDHSSFSGFFLHE | putative C1q domain containing protein MgC1q23 [Mytilus galloprovincialis] | CBX41672.1 /5e-43 | Signal peptide(1-22)/ C1Q（SM000110） |
| **Unigene27658** | 2.7547 | VPIPTIFVGSVQR | 1 | MEYAMMRSMGAVDENTIIITTVHDCQVMDFPDNLIEDHDVVVDYIVTPSEIIKCNRTRSKPTGIIWSKITPDKLRRVPILRKLMKMEEANGKDVTLKDGAAPPEIKTEDEGEESDRQNRRRPFNRRFRRKPFNRRNRRERNSENETEGRGEGKDESEAKSGDEENQRQRRPQRRRRFRRRRNTSENKENEEPRSGDDRRNRRSFGESENDEGKSGDEDKSRRRRRPPPRRFRRNNRRSEGEEGGEDQGQGQSPRRRRGPPRVPIPTIFVGSVQRSVRISELKSKIREKNVNPLRVIWNGAKCYALLQFPKMNEMEEAFDTLNGFEMNGRKLKIEISNRVKQFMDEQHGQGEGHAQVAQDDQPEN | methenyltetrahydrofolate synthase domain-containing protein-like [Mizuhopecten yessoensis] | XP_021347230.1/6e-66 | RRM(SM000360) |
| **Unigene27369** | 2.8558 | SGNEIDFWVQR | 1 | MSEDQPVLVQLRRKDPSSPWGFRMNGGRDQGTVLYIQKLARNGIADRSGMRPGDAILKINNVPATYLDHEQAKMEIIRSGNEIDFWVQRNVIDVGQHQPKPQQKSRAEVDEEPSEYKGYTNPNVQSRSFKILQESLNYSEAPQDEEDGGGQMKLMDMSEFMFRSGLLN | LOW QUALITY PROTEIN: PDZ and LIM domain protein 7-like [Crassostrea virginica] | XP_022313065.1/4e-54 | PDZ （SM000228） |
| **Unigene27247** | 2.7547 | FEELNADLFR;STAGDTHLGGEDFDNR | 1 | FYPEEVSSMVLTKMKEIAEAYLGKTVNNAVITVPAYFNDSQRQATKDAGTISGLNVLRIINEPTAAAIAYGLDKKVGSERNVLIFDLGGGTFDVSILTIEDGIFEVKSTAGDTHLGGEDFDNRMVNHFIAEFKRKYKKDISDNKRAVRRLRTACERAKRTLSSSTQASIEIDSLYEGVDFYTSITRARFEELNADLFRGTLDPVEKSLRDAKMDKGQIHDIVLVGGSTRIPKIQ | heat shock cognate 70 kDa [Thunnus maccoyii] | AGH24757.1/2e-167 | HSP70（PF00012） |
| **Unigene27228** | 2.4997 | VLGLVLLR | 1 | SKQPEREEKRVLGLVLLRGENLVSMTVEGPPPKDTGIARVPLAGVAGGPGVGRAAGRGVPAGAPMPQAPAGLAGPVRGVGGPSQQVMTPQGRGTVAAAAAGASIAGAPTQYPPGRGAPPPMGRGAPPPGMM | small nuclear ribonucleoprotein-associated proteins B and B' [Maylandia zebra] | XP_004558997.1 /2e-76 | Gly(19.8%)；Pro(16.8%)；Ala(15.3%) |
| **Unigene27074** | 3.8972 | NMFDDLDENSDGYLTR | 1 | TPGNSEEDSDEGLLRNMFDDLDENSDGYLTRKEVEDFWKNKGNNEDHTGYFDRADVNKDGKITFEEFKEKIGKEPKPALSHRRREQDEYFARSVDDDKSVETPDKSEEDPNEQSEEGLLRNMFDDLDENSDGYLTRKEVEDFWKNKGNNEDHTGYFDRADVNKDGKITFEEFKEKIGKGPKPT | calmodulin [Phycomyces blakesleeanus NRRL 1555(-)] | XP_018293892.1/6e-09 | EFh（SM000054） |
| **Unigene26867** | 2.7547 | IVVYLNK | 1 | FFPRTFFAIWGTSKYQIRQLLPRNRLTILTIWRNYCKVANSTSKPRCNVGTIGHVDHGKTTLTAAITKVLAKHGQSKLVTFDQIDKAPDEKKRGITINTAHVGYETSKRHYAHTDCPGHIDYIKNMITGTSQMDGAILVVAASEGSMPQTREHLLLAKQIGVDKIVVYLNKMDLVDDELGDLVELEMRELLEEYGYDSTKTPVIRGSA | elongation factor Tu, mitochondrial [Culex quinquefasciatus] | XP_001843948.1 /1e-84 | MMR_HSR1(PF01926) |
| **Unigene2675** | 2.3052 | GSHPLVDGGR | 1 | MMMFFMPLVLMVSSAYAHEIVQWPKGTYTLVKPTAGCPSRWKEGWRKQDNEDNDNQNYITPGHHFYGSFGRNFVLYYCTKDENVIPGTQNWPRGNYCILKSGISCTPGFQTGHIHWDDEDSNNENSYGGVLPSGSYDRNTLIYYCCRSDGSASTPIALPTNKPFYLLRYKGPCQQVKGMHVRDEIVKTDDEDSDNKNSARGSHPLVDGGRNYRLFYCYYY | apextrin-like protein [Mytilus galloprovincialis] | AEK10749.1/1e-96 | Signal peptide(1-17)/ApeC (PF16977) |
| **Unigene26528** | 3.8972 | LTVADALEPVQFEDGEEIVR | 1 | FGELALIYGTPRAATVKAKGDVKLWGIDRDSYRRILMGSTIRKRKMYEEFLSKVSILENLDKWERLTVADALEPVQFEDGEEIVRQGEPGEDFFIIVEGQASVLQRKADDEEPVEVGRLGLSDYFGEIALLLDRPRAATVVARGQLKCVKLDRGRFERVLGPCSDILKRNISQYNSFVSLSV | cAMP-dependent protein kinase regulatory subunit isoform X3 [Mizuhopecten yessoensis] | XP_021346603.1/3e-117 | cNMP_binding（PF00027）；cNMP（SM000100） |
| **Unigene2635** | 3.8972 | FSPDGQYIAIGGGDR | 1 | MTEVTPRCVFASLPRTTRGVPIVLGGDPKKKNFLYTNNNSVFIRDVANPSECDVYTQHQCQVVSAQYSPSGFYIASGDVSGKVRIWDTVNREHILKNEFQPLGGTIKDIAWSGDNQRMAVCGEGREKFAHVFAADTGTSVGDLGGSSKSCNSVSFRAERPFKIVIGSEDFKSAFYQGPPFKFVKTMNEHTNFVNSARYAPDGSVFITGGAEGKAYVYEGKTGDLLGELGSPAHKGGIYAISFSPNSSEVLTVSGDKTAKIWNVANRELVTTFEVGKTVDEMLVGCLWQGDFIMGVALSGYIYYFNRDDSSSPPRVIVGHNKPITAMALSEDRSTIYTADQIARTVCWNAASGENSVFSGKGHSNQVQDMAVTDGKLVSVGMDDTIRFTDLSNNQFGDNSMKLDSMPRGIAARSSVVAVACINHVVVFKNGSKLLSQGVKFEPLSVDVSPNGSLIAVGGDQDRKVHIFDISSGGLTEVNTITCSGSPTCVRFSPDGQYIAIGGGDRYLRLYSMSDLNTTIIEYMGHTAKVTCVAWSPDSSRLATGGLDSQVVVWDPKDPMKPKVFRRAHAASYVNKIAWLNDSVVVSAGQDSNIKQWDA | WD repeat-containing protein 1-B-like isoform X1 [Crassostrea virginica] | XP_022289109.1 /0.0 | WD40（SM000320） |
| **Unigene2567** | 2.0978 | TNESYSNNQNR | 1 | GNKTDQAHPPIHPTKYTDNLQGNEQRVYEFIVRHFLACVSKDAQGFETTVDIEVAEEKFSASGLMITAKNYLEVYTYERWNAKIIPVYNKGDTFQPNSIEMIASETSPPSLLTEADLIALMDKHGIGTDATHADHIETIKSRMYVGVKPDGKFVPGELGMGLVEGYDSMGYQMSKPNLRAELEADLKRICEGKKQKEAVLHQQVAKYKEVFMEACRQAIKLDGALSQYLGEAQNLPIEEVLNVMASVPVKKCPNCGQDMTLKSKREGKGYYIGCMGYPACKNAIWLPDYVLEATVEDSVCQTCLPDEVKHIKFKFKRGSVPPMVPIEYTGCIGGCDETLNEALNLSGQRRSSTSTQRTNTNTSLNNSLSDSGYGSSGQTRSYSTQSSTFHTQISQRPHPNQRTNESYSNNQNRLNNSRQSKGFGSPSTLNRADQNENMRYSRGTGSMESNSRVPLLPVQPFSRGTSGGVQSEDSVVCNCGEDGVLLTVRKEGPNTGRQFYKCSNSKCNFFLWADEVGGNSYGGERNSNYSRGNDSGFQSSQSKRGYPTNDGDVPSCQCGIPGKSLTVQKEGTNKGRQFYGCSKPRGEGCNFFQWADENSGGSGSWGGRSSGGVGGKKQA | DNA topoisomerase 3-alpha-like isoform X1 [Mizuhopecten yessoensis] | XP_021375710.1/0.0 | TOP1Ac(SM000437);zf-C4_Topoisom(PF01396); zf-GRF (PF06839) |
| **Unigene24778** | 2.2109 | IAQNQVAFAMGNHSPFIR | 1 | ESNVATYKEVWNGIVKFNQSDPGVLSESGLDHLHKVTLGKYALIMGKQYIDYEAADNCNLIVLDEKIAQNQVAFAMGNHSPFIRIVSDEILSLHEVGLLEKWKTERWPQKSCDSQEFETRMISVEDIQSAFYMIA | glutamate receptor ionotropic, kainate 3-like [Mizuhopecten yessoensis] | XP_021356287.1/7e-31 | PDB 2V3U\|A |
| **Unigene23964** | 3.8972 | YDFILSANQPIR | 1 | RLEGYHDHPCIRQCEEDEEPKECTYDFTVEYYYTLTQACYDCPYNITDCLRPHCVSADGISRGLITVNRMLPGPSIHVCKGDTVVVNVKNKLEGGEGVSLHWHGVLQEGTPHMDGVSMLTQCPIHPYTTFQYRFKANDPGTHFWHAHAGLQRTDGIFGSFVIRQPRAHDPHSILFDDDLPEHTIMLNDWMEHIGAVSFAAGHHAMKDHFPTSILINGKGTVKEFNEMMDHSAHKDSALPEDMIMDTLTTTRTMEMNHVDQMFEHMMHRRSSENQEMPPSHNMDNMPHSMTDLDARHTPHSIFNVKQGKRYRFRVISNGIANCPIQFSIDHHSLTMVTTDGSPFNPITVESFTVFAGERYDFILSANQPIRNYWIRARGLAECGPQYKSVSQTAFLTYEGSAIVLPQEPPDYHSGNRPGLMLNPLNIGPSRGFITVD | laccase-2-like [Crassostrea virginica] | XP_022312912.1/2e-128 | Cu-oxidase_3 (PF07732）;Cu-oxidase(PF00394) |
| **Unigene23851** | 2.8558 | VSGTTIPGYSVNR | 1 | KQCSGSQCYCVTRVSGTTIPGYSVNRWEAQDQNCQCARDKVENTVPGKIFLCDKMGNYKETPCQKEYRENTEATNNGAMGARIPKCGPDGHYDPKQCSGSQCYCVTRIQGKMIPGYSINVWEAQDQNCRCARDKYENTIPGKIFLCDEKGNYRFPCNGYPC | saxiphilin-like [Crassostrea virginica] | XP_022328567.1/3e-19 | Internal repeat；TY（SM000211） |
| **Unigene23701** | 2.4307 | ANVQLNGVDSAAIR | 1 | SSFRKPSAAVLFTLCWFYYTRPVTDCDDCWQYGVVIEHFITLSMMILPILLVMLFTEVSGRMKMGARAHCDDGHTKLDKDFTCAEFMCNGRQIKGAVKNIIVKKHMKEKDYGNDHACMDKTLSYNVTIPSSGTHRPAWPKYGEYPYVPPQRWIHSLEHGAIVFLYNPCLERSQVELLWKETQRMARKRKHLFIMTRYNLPADMPIALVAWRANVQLNGVDSAAIREFIKAKYNQGLEKVSKDGQYNYLQGKKEIPVEFIEFCRMMNVQSQFVQQYPYNLI | AGAP006915-PA-like protein [Anopheles sinensis] | KFB50798.1/9e-36 | DUF3105(PF11303) |
| **Unigene2277** | 2.7547 | ITPSYVAFTEDGER | 1 | MKLFLSVVALLAISLVTAKDEDSKKESVGTVIGIDLGTTYSCVGVFKNGRVEIIANDQGNRITPSYVAFTEDGERLIGDAAKNQLTTNPENTVFDVKRLIGRDWSDKSVQADIKHYPFKVVNKNSKPHIQVNVGDAAKSFAPEEISAMVLGKMRDIAEGFLGKKVTNAVVTVPAYFNDAQRQATKDAGVIAGLNVMRIINEPTAAAIAYGLDKKEGEKNILVFDLGGGTFDVSLLTIDNGVFEVVSTNGDTHLGGEDFDQRVMEHFIKLYKKKKGKDIRKDNRAVQKLRREVEKAKRALSSAHQARIEIESLFEGEDFSETLTRARFEELNMDLFRSTMKPVQKVLEDADMKKEEIDEIVLVGGSTRIPKIQQLVKDQFNGKEPNRGVNPDEAVAYGAAVQAGVLGGEEDTGDLLLLDVNPLTMGIETVGGVMTKLIPRNTVIPTKKSQVFSTAADNQPTVTIQVFEGERPMTKDNHLLGKFDLTGIPPAPRGVPQIEVTFEIDVNGILKVSAEDKGTGNKNNIVIQNDNNRLSPEDIERMINDAEKFADEDKAVKEKVEAKNELESFTYNLKNQIGDKEKLGGKLSDDDKSTIETAVDEKIKWLDSNPDATTEELKEQKKELEEKVNPIISKIYQGAGGAPPPGGEEEGSEKDEL | byssal HSP-like protein 1 [Mytilus coruscus] | ANN45953.1 /0.0 | Signal peptide(1-18)/MreB_Mbl（PF06723） |
| **Unigene22659** | 2.6077 | VNGFVQR | 1 | TLKQVLAKLPGLTDRVNGFVQRIFDDDILGFVEEEEFESIGCFLRELKQNVFSKDVASLEDLKSKFKDAANQDDNVFTCTDPQEVLDYYKNGLEILNTP | tetratricopeptide repeat protein [Cecembia rubra] | WP_106568208.1/3.9 | Leu(12.1%)；Asp(11.1%) |
| **Unigene22033** | 2.3052 | TINNDPIIGDR | 1 | AIHSNIIKNETFKDFAEMFPERFQNKTNGITPRRWLLLCNPSLSDVIADKIGEGWVTNLKELEKLKKFASDETFLRTVIKVKQENKMKLADYIKQQFNLTVNTSSIFDMQVKRIHEYKRQLLNCFHMIVLYNRLKKNPSMQFTPRTIMVGGKAAPGYHMAKLIIKLINNVGRTINNDPIIGDRLKIVYLENYR | glycogen phosphorylase, muscle form-like isoform X3 [Crassostrea virginica] | XP_022331556.1/2e-105 | Phosphorylase(PF00343) |
| **Unigene21654** | 3.8972 | LLTAENIIVMR | 1 | MIKIILIICIVGSSFGQAGGWQDYHGPIPIAVLNSVRMKLLTAENIIVMRMETRNAKSQVVTGVNYTFDVKVTSVGGQIVNCHFLVFQGLSGIARVKSHTC | 50S ribosomal protein L23 [Methylobacterium sp. 17SD2-17] | WP_109895574.1/0.076 | Signal peptide(1-20)/SCOP d1stfi_ |
| **Unigene20852** | 2.7547 | YGLEDYIEIK | 1 | SADIEKAVTGVMGSKFRNTGQACICANRIFVQDSIYDKFVSSLANRMTKELHIADGFDDKATQGPLINQRAVDKIESLVNDAKEQGGKVVVGGQRRKGNFFDPTLISDVTTKMRCYNEEQFGPLAAVIKYHTDEEVISMANNTSSGLAGYVFTENINQMWRIAEKLEYGIVGVNEGLPAMPEAIFGGWKESGLGREGGKYGLEDYIEIKYVCLGGLSD | succinate-semialdehyde dehydrogenase, mitochondrial-like isoform X2 [Crassostrea virginica] | XP_022301488.1/2e-93 | Aldedh（PF00171） |
| **Unigene20448** | 2.8558 | APGQAWGFR | 1 | MATVQVQRAPGQAWGFRLAGGRDFNVPLQIKKVEQGSPVAGVLSPGDNIIGIGHSDARNMTHMQANQMIRGAGNMLQLTIVKGHGDVNSRISSIKPKGPVKFSPWKAQST | PDZ and LIM domain protein 3-like [Mizuhopecten yessoensis] | XP_021353042.1 /8e-36 | PDZ(SM000228) |
| **Unigene20345** | 2.4307 | MGIPMHNFIR | 1 | ADTRQIIKTIRDSFACRKNNPGLSVAIVKDGQIVYANGFGVQSLESKKPVNKDTLFGIASLTKAFTSTLLAKLTDKNVNYSLNTQVAEFYGNSPVFDGYFRSKFASIKDLLSHRMGIPMHNFIRFDSSLTRRNLIRRLKVLRPRGRFRDSFYYSNIMYGQAARIAEKIGRKKFEDLITDELFVPIGMTKSGFFTTANE | Protein flp [Crassostrea gigas] | EKC28749.1 /7e-54 | Beta-lactamase(PF00144) |
| **Unigene20294** | 2.7547 | MGHAGAIIAGGK | 1 | PVLTMSTATKVLGKVSAIGRVGIRTCYTNSRPNLGINKKTKVICQGFTGKQGTFHSQQAIEYGTKMVGGVSPGKGGQKHLGLPVFNSVKEAREQTGADASAIYVPPPFAAAAIIEAIDAEVPLIVCITEGIPQQDMVKVKHKLIRQSKSRLVGPNCPGIIKPGECKIGIMPGHIHKRGKIGIVSRSGTLTYEAVHQTTQAGLGQSLCVGIGGDPFNGTNFIDCLEVFLQDPQTHGIVLIGEIGGQAEEKASEYLRNNNCGSDAKPVISFIAGVTAPPGRRMGHAGAIIAGGKGGADEKIEALREAGVDVTMSPAQLGTTMAKAMSAAGKL | succinate--CoA ligase [ADP/GDP-forming] subunit alpha, mitochondrial-like isoform X1 [Mizuhopecten yessoensis] | XP_021357524.1 /0.0 | CoA_binding(SM000881);Ligase_CoA (PF00549) |
| **Unigene20162** | 2.6077 | GMTAMGAVR | 1 | ISHVGTEAQRLNYNGPTIGAKPTEKRAVKFSYEQLKQSCGLIGLQSGTNKFASQRGMTAMGAVRHISDIRADKFSKEAEGEINLQSGTNKFASQRGMTAMGAVRHICDIRADQYDPESNKEINLQSGTNKFDSQAGMRGFGAIRHISDVKVNELDREGTSVLRLDMGYVGGDSQKGMTSFGAQRHITNVKVNDLAEEFALQHGKPAPTPQPQAVEEVAQEEEEE | calponin-like protein-2 [Mytilus coruscus] | AKS48163.1 /2e-125 | Calponin（PF00402） |
| **Unigene19648** | 2.8558 | VVGNTAYFNITAPCR | 1 | PAPPQEFQLQPVTAEQTSAPAPAPAPATKPKPTLDAIDRQVLHNVDDHAIQVSKSQHSSFRDMIWDLIYSKNITNEIEKVRVIFRWLATKNLKEMHFDNVEKDSPEEVLLGLKTGKTTYAMVFDTMCNYAGLHSKIISGYAKGADYRPGQSFTPGTNQHSWNAVYIYGTWCLVDCHWAARRIIGKQTTHEEFHYQLDEYFFLPDPHQLIYTHFPEDGKWQLLERPVSLEEFESMPHMKPQFFKYGLEFVSHRTAVIYGRGEVNIRLRYPAHRIVVAFNFTIQYENGDEEYRGTKLNKYGMQESVGGIASFRLRLPEKGSYIIYIYAKEDTQENKDNVYAQVCEFKIVQEEVMAPPPQPFPPCAYLNWGPGTAFYKYGLQSYQNTATILTREGKVELQVRIPKPMQFMAKLKHNDMSDSELEGYIMNRVVGNTAYFNITAPCRGEFGLEIYANDPSVEGQTLYHVAQYLIECHEDVKTVPLPKLPHGYLGPQPKFNEFGLNTLSHHDSVIHLETNSVEIQFATAAEMRVTANLIGVDDEKEHPDLVFTQTQGDIVSFVVHIPATGFYKLQLYAIPTRDPSQQLPGVYNYLINCQKIVRASYPFPKQYAQWKEGCYMYEPLSLHRDVTPPTVNFKIGIPRAEAVAVVADSDWHHLQQSQPGVWEGKVPLDKHYGRGVKVTVNANYGGDKTNYATLLEYNV | hillarin-like [Mizuhopecten yessoensis] | XP_021344941.1/0.0 | TGc（SM000460）；SCOP d1bf2_1 |
| **Unigene1897** | 2.8558 | EAESAVFHTQLFEELR | 1 | MSGTKLGGSGSSAKFDLDAMEQEGAYYCQSQQLQAAYASSHLEHMCCLDIDSEPHEVRMTGIICTIGPACVAVPTLQKMIIQGMNIARLNFSHGTYEYHGNTIKNIREAVSKFSVPKPVAIALDTKGPEIRTGLLKGGASAEIALKTGDKIKLTTDEKYYEECTADILYVDYKNITKVMNVGGKIFIDDGLISVIVKEKGDDWLMCEIENGGDLGSKKGCNLPGTPVDLPAVSEKDKKDLLFGVEQGVDMVFASFIRSGSHIQEIRKILGEKGKNIKIIAKIENHEGVKKFNEILNEVDGIMVARGDLGIEIPPEKVFLAQKMMIGRCNRYGKPIICATQMLESMVKKPRPTRAETSDVANAVLDGADCVMLSGETAKGDYPLEAVKIMHKICREAESAVFHTQLFEELRKHTPTPTDPTHTVAIAAVEASFKCMAAAIIVITTSGRSAHLISNYRPRCPILAITRKDQTARQGHLWRGVFPIHYIEPKVSSWTEDMDKRIYKGIQVGRRRNFIKTGDPLIIVTGWRTGAGFTNTMRIVNTPEKDGDPIIGTPVIADDE | pyruvate kinase PKM-like isoform X3 [Crassostrea virginica] | XP_022311726.1/0.0 | PK（PF00224） |
| **Unigene18932** | 3.8972 | FEELNADLFR;TTPSYVAFTDTER | 1 | LFGYKIAAASRGIHLKEKKRAYKNKKEQSPQNRNMAKAPAVGIDLGTTYSCVGVFQHGKVEIIANDQGNRTTPSYVAFTDTERLIGDAAKNQVAMNPVNTVFDAKRLIGRKFDDASVQSDMKHWPFTVINDSSKPKIRVEYKGEQKTFFPEEISSMVLVKMKETAESYLGKTITNSVVTVPAYFNDSQRQATKDAGTISGMNVLRIINEPTAAAIAYGLDKKATGERNVLIFDLGGGTFDVSILTIEDGIFEVKSTSGDTHLGGEDFDNRMVNHFIQEFKRKHKKDISENKRAVRRLRTACERAKRTLSSSTQASVEIDSLFEGIDFYTSITRARFEELNADLFRGTLEPVEKSLRDAKMDKASIHDIVLVGGSTRIPKIQKLLQDFFNGKDLNKSINPDEAVAYGAAVQAAILSGDKSEEVQDLLLLDVAPLSLGIETAGGVMTSLIKRNTTIPTKQTQTFTTYSDNQPGVLIQVYEGERAMTKDNNLLGKFELTGIPPAPRGVPQIEVTFDIDANGILNVSAVDKSTGKENKITITNDKGRLSKEEIERMVNDAEKYKDEDEKQKDRIGAKNSLESYAFNMKSTVEDEKLKDKISEDDKKVIMDKCDEIIKWLDANTLAEKEEFEDKQKELEKTCNPIITKLYQAAGGAPGGAGGMPGGMPNFGGAGGPTGGAGSGGSGGPTIEEVD | heat shock protein 71 [Perna viridis] | ABJ98722.1/0.0 | MreB_Mbl(PF06723) |
| **Unigene17750** | 2.8558 | FNTIAECMNACR | 1 | MMWLPLGFITLAIFHGVNGQAANAKQKAAKTVGKMDKKCFDNPRTGMCMGMFQAKWFFNEATGKCVMSQGCFYQGFISMQECRKECQCRQPLNEGSGSGPVGANCELEVQKYAMVGEVCTPFMFTGCGGNGNRFNTIAECMNACREREPLDMMGEMMPGMMGWAGMSGMGGGMMGNMNFRGA | proline-rich protein HaeIII subfamily 1-like [Crassostrea virginica] | XP_022333931.1 /2e-29 | Signal peptide(1-19)/KU（SM000131） |
| **Unigene17193** | 2.7547 | GLVVPVIR | 1 | LKSMALLLVQRCLPRITRRLSPTNTKFVLEEASKNIRVKSHICCSSQCRNYTDVQYIRFTKKYCCNSQLIIKRNFHVTNRYFDDVLTAATPPFADSISEGDVRFEKAVGDFVKEDEIVCEIETDKTSVPVQAPKSGIIQSFLVDDGATVQAGTPLFTLKLSDSPGESAPESVAASEKPPPPAVTSKAPETPVATPASGPIPTTPPPPQPIPKAPISTKPLDSIKPIPATDAPVMGARTEKRVKMTRIRQKTSQRLKAAQNECAMLTTFNEIDMSNVIEMRNQYKEAFQKKYGLKLGFMSAFVKAAAYALTDQPAVNAVIDESEILYRDYIDISVAVSTPKGLVVPVIRNVGSMNYADIERAIAELGEKARTGSLAIEDMDGGTFTISNGGVFGSLFGTPIINPPQSAILGMHAINDKPVAIKGKVEIRPIMVVALTYDHRLIDGREAVTFLKKIKSAVEDPRVLLLDL | 2-oxoglutarate dehydrogenase complex component E2 [Mizuhopecten yessoensis] | OWF50391.1/0.0 | Biotin_lipoyl_2 (PF13533)；2-oxoacid_dh（PF00198） |
| **Unigene17031** | 2.0656 | MGILDR | 1 | DESHVVNPADVIVPTLVPSRVPPTRAAVQEEVQPLDKVQKQQTTPPVTIFKMKIIQTVVNSNVDQDSEKKIQHTVRVETPATKNDISVNIEHTSKNIDNVGSGLQSSVVPNQDTMTRERMGILDRKPPTISNKEDSSDSIEDVVHELTPIPEMEIIHPNQEDEVKSILSVYDNVTSTKDSSSSEEVKIQGPKLVSETETEVESSESVSSVSEQSEESFPAIEQTSIEQGSESSSEQTISEIKTTAKSNTSTKPPFRISEKTTTKAPNLRSPESRHHKGSGSSGSRTECSNVLVSFFASLIYFLLIT | — | — | Ser(15.4%);Glu(10.5%);Val(10.1%) |
| **Unigene16540** | 2.4307 | QDIGVTHLLR | 1 | LSRDLYHSSYRNCSRLFNETPRMASRTPGLLLRSLKGRLYGTAAAAAESSEGASLVRQPARVTKLDNGLTVASIENCSPVSQVAVLVNAGSRFESRQDIGVTHLLRTGTGLTTKNMSDISLLKTVQQAGASLGCTGTREYLIYNVEALRNEVFKTPMDALNEIIVNPDFRNWELQFGSIRNHLRVQLADLKEQPQVRLMEMVHEAAFRDQLGRSLYTQPQFIEQFNQEQLLAYHKKFYTLDRMALVGVGVSHEDLVAEGKSFTLGKGQNLSTEKSVYHGGEIRHSSGEDMTHAAIVTEGPSLKSKDLFAVCILQQIMGTGPFIKYGDNRLSKLGKAVCEAIPEHAYATTCVSANYTDSGLFGFYLCAAPADMEKGLRAAMNTFASVTKSGVTDEEVTRGKNLLKSAIAMNLEQSENVLQDLAEQAYGSEKINSTEDLIKAVDAVQTSDVSSVAKKIINGKASMASLGNLQSVPYLDELTK | cytochrome b-c1 complex subunit 2, mitochondrial [Pelodiscus sinensis] | XP_006123876.1/1e-101 | Peptidase_M16 (PF00675);Peptidase_M16_C(PF05193) |
| **Unigene16201** | 2.7547 | NTMEIGVYTGYSLLATALAIPEDGK | 1 | SKAPIFLFDHIHKARKINTAMAENGEEQKSRHQEVGHKSLLQSDALYQYILETSVYPREPECMKELRELTAKHPWNIMTTSADEGQFLNMLLKLINAKNTMEIGVYTGYSLLATALAIPEDGKILAMDINKENYELGLPVIEKAGVAHKIDFREGPALPVLDQMIEDEKNHGSFDFIFVDADKDNYINYHKRLIDL | caffeoyl-CoA O-methyltransferase [Rosa chinensis] | XP_024168231.1/2e-122 | Methyltransf_24 (PF13578) |
| **Unigene15131** | 2.4307 | LPDAVLNVVR | 1 | NRELCAMFKTILILCIIGSSLGQPGGWQDYHGKLPDAVLNVVRKRLLVAENMIVAKMETRNVKSQVVAGVNYKFDVKVTGVSGRIVDCHFLVYVNLSGIAKVKSHKC | 2-oxoglutarate dehydrogenase [Halioglobus sp. HI00S01] | KZX60278.1/0.011 | Signal peptide(1-22)/SCOP d1stfi_ |
| **Unigene14496** | 3.8972 | SNTLQNLHVQQPAR | 1 | GACCGAPPSPEMGMRSNTLQNLHVQQPARQQYETQASILTNFNEMANVYVDKNAQKTPAKNESDVLSPDYRPYLPPSTYSPFGPYGAPSQPNNGNYDQNAQIKK | — | — | Pro(12.5%)；Asn(10.6%) |
| **Unigene14034** | 2.7547 | DTDIAGVTQDSR | 1 | ASHQQNQQQQQQIQHTEHREYKYNTQNFNYNQQPNQQFMTGSQQHINMTEGMPAQNIPIQHQQGRYQQQQNQHQQQLQYHTGSQQNINNMLDGMPVQNMPIQHNHGGSQFNTMSSGHGSVSSPQGFNTLGSNQSYRTEQHQKYHTMSSSGSHDGFGTMGSQNFGTMSSGHGYNTMGSQQNGSLHVDTTNRSMHSGPSSAGSPHSPDTLNALRQQLHVAHNMSSSSGGALSPGPHSMTGQSSPSVYFGMSRRGSLTSLADTADAVHATPKFVKNTSKYWYMPNITREEAISMLKDKAPGTFVVRDSNSFPGAFGLALKVATIPPNVQTKSSGDPAADLVRHFLIEPTPKGVRLRGCSNEPVFGSLASLVYQHSITPLALPCKLVLPEVDPSIESSMDVTHTSEQPSSAAALLAQGAACNVLYINSIDTESLTGPQAVARALKLTFDTAPSPTTTVVHFKVSNQGITLTDNQRKLFFRRHYPVSAVTYCGMDPESRKWKRDTDIAGVTQDSRVFGFVARKHSGASDNACHLFAELDPEQPASAIVNFVTKIMIGQGSKK | Tensin-1 [Mizuhopecten yessoensis] | OWF49222.1/3e-175 | Internal repeat;SH2(SM000252);PTB(SM000462) |
| **Unigene13412** | 2.6077 | ILEFFGLK | 1 | MLPILLSACVIALTFGADIKEEEGVLVLTTANIEDALKENTNILVEFYAPWCGHCKALAPEYAKAAKALADEGSDIKLAKVDATVESSLAEKYEVRGYPTIKFFRDGKAIDYSGGRQSADIVNWLKKKTGPPCLALESVDDAKKMVEKDEVVVIGFFKDLKSADAQQYEKAAQGIDDIPFGITSNTDLFKEYEMESDGIALFKKFDEGRNNFEGSVTAEAVNAFVMANRLPLVIEFTQESAQKIFGGEIKNHILMFLEKTADGSDKLIDGYKKAAETFKGKVLFITLDTSDEDNARILEFFGLKKEDCPSARLITLGEDMTKYKPESNDLSEDAVRSFVQNFLDGKLKPHLMSEEIPSDWDAKPVKVLVGKNFQDVAMNKDKAVLVEFYAPWCGHCKQLAPIWDELGEKYKDSADIVIAKMDSTANEVEDVKVQSFPTIKYFPKGSSEVVDYNGERTLDGFVKFLESGGKDGAGEAEDEDDDDEEEDEDEPAAKDEL | procollagen-proline dioxygenase beta subunit [Mytilus galloprovincialis] | AFM30917.1/0.0 | Signal peptide(1-16)/Thioredoxin （PF00085）；Thioredoxin_6 (PF13848) |
| **Unigene13309** | 3.8972 | GILGNSGGSGGGLLSR | 1 | QLLRAQPQGSTENLWEDELNNTMRILSIFVFLAALACADALLSDLLRLQVLKGILGNSGGSGGGLLSRLGQSSNGAQSASVVSSSSAGSYMENYYKLQYCRETPFRFIRKCTTSSQCSPYLECFENVCCATNPLSLRIVD | — | — | Leu(15.0%);Ser(13.6%) |
| **Unigene13266** | 2.6077 | NATTQGVSGTGSLR | 1 | PKFLTLKMALRQAKKLTNFTRLLPQCTSITTVKEGSTWANVEMGPPDAILGISEAFKKDSSPNKINLGVGAYRDDTGKPFILQCVKTAEKQMFDENVDHEYAPISGTPAFCKASAELAFGEDSPVLKEGRNATTQGVSGTGSLRLGAAFFSKYYTKSKVFYVPTPTWGNHGPIFKHAGLDVQTYRYYEPKTCGFDFNGALEDISKIPEGGVVVLHACAHNPTGVDPKPEQWKEISNVIKQKKLYPFFDMAYQGFASGDISKDAFAVRQFLADGHELALCQSYAKNMGLYGERAGAFTLVCPSKEEADRVMSQIKIIVRAMYSNPPIHGARIVERVLTKPELRNVWLGEVKGMADRIITMRQKLRDGLAREGSSHNWQHITDQIGMFCFTGLKPDQVERLTKDFSIYLTKDGRISVAGVSSGNVDYLAKAMHEVTK | aspartate aminotransferase, mitochondrial-like [Crassostrea virginica] | XP_022344823.1/0.0 | Aminotran_1_2 (PF00155) |
| **Unigene12198** | 2.7547 | HAFGDQYR | 1 | SLLSKCVEMASPVAALARVVCRASGTAQNLVAPAVITNNPKRNYAGPAKRIKVANPVVELDGDEMTRIIWEKIKDTLILPYLDVDLKYYDLGLPYRDQTDDQVTIDAAMAIKKYNVGVKCATITPDEERVEEFKLKKMWLSPNGTIRNILNGTVFREPILCEKIPRLVPGWTRPIVIGRHAFGDQYRATDFVAQGNGKFEMVWTPEGGAEQRMEVFNFTNGGGCVMGMYNTDESITGFAHACFQYAINKKWPLYMSTKNTILKRYDGRFKDIFQEIYDKQYKGEFEKLGIWYEHRLIDDMVAQALKSDGGFVWACKNYDGDVQSDVVAQGYGSLGLMTSVLVAPDGKTIESEAAHGTVTRHYREHQKGNPTSTNPVASIYAWTRGLEHRGKLDGNPELQRFANTLEKACVATVDSGKMTKDLAGCIYGLKNIKPEQYLYTMDFLAAISEELNRQLK | isocitrate dehydrogenase [Mytilus trossulus] | AFI56373.1 /0.0 | Iso_dh （SM001329） |
| **Unigene12120** | 2.4307 | LALIEYR | 1 | LDHSLKMVPSFQMFSVLIALTLLPNFGLFEIIEQLETPKGKPVNLDYPLDLAFIMDTTGSMGSYIRSAKENIEAIVNEIIKTSKSRVRLALIEYRDHPPQDRSFVTRVHDFTESVGEMKRWLESAKAQGGGDSPEAVADGLYNVTKLAWSAESTKIAVLISDAPPHALDPTAGNSFMNGCPEGHDPMQTVREIAKIGVTLYSVGVEPSINAYKEFFAAMAYITGGQYVPMRDPRQLINVIIGGAQEELSLKEFKTEVENEIEQVIDNGGVYDEDEIAQAVYQKLESRGAKSTHLVKNKKGLKGPNKLTLRLAKVSSMEQVRKKMEKKIKQRKSRLYSRRHMSSRAPDTRRYRKSRLERPEITSMHEHTEMDSFGLPGSLPMYSMALDEHEPRKSKSKMKKKKKESYSTEKSTVSLDQIHRLVKKLSFKRKGSP | LOW QUALITY PROTEIN: alpha-protein kinase vwkA-like [Crassostrea virginica] | XP_022331188.1/1e-97 | VWA(SM000327) |
| **Unigene10714** | 2.0978 | YQQQILDVHNDYR | 1 | HQEYEESYTMYGVTILAGLVIIASGQIVQDEGGKYQQQILDVHNDYRKKQGASNMNKLVWDKQLEKEAGDWAKGCRFEHQMKGRGENLAFQTFNKDPDYKTMLDKAMKGWYDEINMYSYSGKSCHASCHYTQMVWAETRLVGCAMNLCPASAFGFGRPGWKAWYFVCFYDPVGNDITQYPYLVGNKPCDKCMEGQSCEDSLCTGKGKMTCDDKEDKCPYWEFKGECQKDKKMMEKNCRRSCNFCEDDDIKVAEVCKDKNPECANYPKTECEKNKAYMVMHCRKTCGFCK | Cysteine-rich secretory protein LCCL domain-containing 2 [Crassostrea gigas] | EKC31006.1/9e-79 | SCP(SM000198);ShKT(SM000254) |
| **Unigene10230** | 2.7547 | VQYSNSQNNLYHHQTR | 1 | NFPTPSATSYRTSETNKMSKQKRHRNDHINNAQMSQNSQISKRRQYLATRQRTQQPTQRRQKQVSRQIVAQRRQQSVSRQRVKSTTQQRQQPIQRKGVQQMSNQMSYESMIDSKNEITALILKYMASLPMDTAMETAIRECDGYARPVCKALPMWTNNRPFDEWCSVLCPTGVCPAAVCSCTCPQQNSRYQQNRVQYSNSQNNLYHHQTRIRCKAISVWGDPSMDQWCSSTCNANPDNCPTDHCWCDGL | chitin binding protein [Pinctada martensii] | AIF72920.1 /6e-14 | Internal repeat |
| **CL955.Contig1** | 2.7547 | CNMISGEVEELR;ELEGELDSEQR;ELEGELDSEQRR;ESYNLAER;GQLEISNVR;LEEAESQALK;SSVSISR;VGLSVIQR;VRELEGELDSEQR;YQQQVSEVQR | 1 | TASVLHMGEMKFKQRGEQAEPDGTAEAEKVSFLLGVNSNDFVKCLVKPKIKVGTEVVAQGRNKAQVMNSISAMAKSLYDRLFAWLVKRVNHSLDTKAKRNYYIGVLDIAGFEIFDFNTFEQLCINYTNERLQQFFNHHMFVLEQEEYKKEGIQWEFINFGMDLQACIDLIEKPMGILSILEEQCMFPKADDKSFKEMLFTNHMGKSPNFTKPGKAAKGKNGDFELHHYAGSVPYNIAGWLDKNKDPINETVVELLQGSKEHLVVTLFAPPEGAEATGGTKKKKKSSAFQTISAVHRESLNKLMKNLYSTHPHFVRCIIPNEMKQPGVIDAELVLNQLQCNGVLEGIRICRKGYPSRIIYAEFKQRYSILAPNAVPQGFVDGKVVTEKVLLALQLDPAEYKLGNTKVFFKAGVLGNLENMRDERLGAIVSMFQAHIRGYLIRKAYKKLQDQRVGLSVIQRNIRKWLLLRNWQWWKLFAKVKPLLNYAREEEEMQKKMEMMKKMEEDLAKTEKIKKELEIKNVELLEQKNDLFLQLQTQEDTVIDLEERVQQLVNQKCEFEAQMKEMEERLLDEEDAAAELENVKKKMEGENSELKRDIEDLETTLAKAEQDKTTKDNQIKTLQGEMAQQDEQIGKLNKEKKNMEELQKKTLEDLQKEEDKVNHLNKVKQKLEQTLDEMEDSLEREKKIRGDVDKAKRKVEQDLKATQELVEDLESNKRELEEANRKKDSEMSMLNSRIEDCEGVNAQQNRKIKDLMATIEELEEELEAERAARAKAEKQRAELARELDEISSQLEEQGGATQAQVDLNKKREQELVRLRREMEEMTLQNESQVSQIRKKAQDQANELADQIDGLNKLKSKLEKEKKDLKRELDDVQSQVQYSMKNKGVSDKVAKQMEVQISEMNSRVEESQRTIVDINSLKTKLQSEVADLNRQLEDAEHNIGSLTKDKTSLNHQLEESKRSLEDETRTRQKLQNEIRNLNADVDSIREAFEEEQESKSDLQRQLSRAKNEAQQWRSRFETEGTAKADELEEAKRKLAARLAEAEQNAEAANAKASGLEKAKNRLQGELDDLLVEIERSNVSSSTLEKKQRQFDRTIQEWTTKVKELQTEVDTAQAEARGYSAELFRSKAQYEECNSTIESLRRENKNLADEIRELTDQLSDGGRNAHEVEKAKRRLEMEKEELQAALEEAESALEQEEAKVMRGQLEISNVRSEIERRLAEKEEEFENTRRNHQRALDSMQASLEAEAKGKAEAMRIKKKLEQDINELEIALDASNRAKAELEKNIKRYQQQVSEVQRQVEEEQRQKEEVRESYNLAERRCNMISGEVEELRTALEQAERARKGAENELFEANDRVNELSAEVQSISSQKRKLDGDIQAMQSDLDEMNNEVRNADDRARRAQEDSARLADEIRNEQEHSQQIEKFRKSLEGQVKDLQVRLEEAESQALKGGKKMIAKLEQRVRELEGELDSEQRRHAETQKNMRKADRRLKEIAFQADEDRKNQESLNSMIDTLNAKLKTYKRQVEEAEEIAAINLAKYRKVQQELEDAEERADSAEGSLQKLRAKNRSSVSISRSSVTHTPATSPSVLNSSNLLSPRSMSRGPDSSFLSPRSASRGPGLYRRSVTPSYEDDDY | pedal retractor muscle myosin heavy chain [Mytilus galloprovincialis] | CAB64663.1 /0.0 | MYSc（SM000242）；IQ（SM000015）； Myosin_tail_1 （PF01576） |
| **CL79.Contig3** | 2.1286 | NSGAQVYDSNTGR | 1 | MNPPCAKCSKTVYPTEKLNCLDKIWHKGCFKCQVCNMTLNMKNYKGYDKLPYCNAHYPTTKHTAVADTPENRRIADNTKIQSNIKYHEDFERQKGKKLTVVDDPETQRVRQNTANISQVVYSGHKDQLKDMEYNRPAEQVNDVRVRPNPGSIHSYDPMRDQQQNQGTPYSQRNSGAQVYDSNTGREDRNFNARIQPFYQHPAYGMDNPSSQRRVGSISDYDPMNDRWGSVAGQFNAQNQQQRNQPPPPQQYAPPPQEADRFAGKGDKNDDDLSYGYDTTGKGMVCRAAYDYVAADDDEVSFNEGDFIIFCQPIDAGWMEGTVEATGRRGMLPSNYVETVKK | LIM and SH3 domain protein F42H10.3 isoform X6 [Lingula anatina] | XP_023930739.1/3e-101 | LIM(SM000132);NEBU(SM000227);SH3(SM000326) |
| **CL563.Contig3** | 3.8972 | AGFAGDDAPR;AVFPSIVGRPR;DLTDYLMK;GYSFTTTAER;GYSFTTTAEREIVR;HQGVMVGMGQK;IIAPPER;IWHHTFYNELR;QEYDESGPSIVHR;SYELPDGQVITIGNER;TTGIVLDSGDGVTHTVPIYEGYALPHAILR;VAPEEHPVLLTEAPLNPK | 1 | MCDDEVAALVVDNGSGMCKAGFAGDDAPRAVFPSIVGRPRHQGVMVGMGQKDSYVGDEAQSKRGILTLKYPIEHGIVTNWDDMEKIWHHTFYNELRVAPEEHPVLLTEAPLNPKANREKMTQIMFETFNAPAMYVAIQAVLSLYASGRTTGIVLDSGDGVTHTVPIYEGYALPHAILRLDLAGRDLTDYLMKILTERGYSFTTTAEREIVRDIKEKLCYVALDFEQEMATAASSSSLEKSYELPDGQVITIGNERFRCPESLFQPSFLGMESAGIHETTYNSIMKCDVDIRKDLYANTVLSGGTTMFPGIADRMQKEITALAPSTMKIKIIAPPERKYSVWIGGSILASLSTFQQMWISKQEYDESGPSIVHRKCF | actin, adductor muscle [Crassostrea virginica] | XP_022325998.1/0.0 | ACTIN(SM000268); |
| **CL4656.Contig1** | 3.8972 | LNDQIGGYEGELANLR | 1 | MSQEKVEVRTRNTKTAQTMGPRSTIITRHSTSGTLPMAGTRSSTFRASYGGGGFGGAASFATGTVSGMSQKNVANVLDTRAKEKNEMNVLNERFASYIEKVRFVEAQNKALLAEIERLKKQKNFDVSEIKELYEQEIADSRKIIDDLSDEKAKFDSTLVSLQDQLEDEKRDRINAEKTVDDLRNKIDRLNDQIGGYEGELANLRLRIDSLEDENARLKKDKKTLQDDIARIRADLDEETCKRIQAEMKLQTAEEDFKFNQNIYEAEIAELRAMLDKDKSIEMKDIWKGEIQKAISELQAQYAAELDRMQGDMQKNFEMQLNEMKAGVNRDNMEALSAREESKKVKGKLSELQPLINQLQAENAMLKSRLDALQIQYDDECREHEEDRLKLESQIQKLSSELESILRELQILQDAKLSLELEISCYRKLLESEEQSLKRVVEESSGARSSGAQLLSDMIVTKGGSEASQKSSMSTSSRKVNLVKNSRGDLRFERCDPSGTKVTIKNNGTKSISMRGWRLIKNINGVDKCKFNFADDYTIGAQREVTICGKVMADELEYGELLGDFNTWGTHGKFILFDDKNVEKASMDVQVL | retrograde protein of 51 kDa-like isoform X4 [Crassostrea virginica] | XP_022320110.1/0.0 | Filament(SM001391);SCOP(d1ifra_) |
| **CL4349.Contig1** | 2.8558 | SQHQDLGWAGSCLQR | 1 | GMKGEKGGLGNSGPPGPRGQDGLPGMKGNPGLDGLPGTSGPKGESGFPGNNGFPGAKGETGLPGLNGQPGIDGVQGEPGTPGLPGRDGDPGLPGLKGDPGQPGFEGLPGAKGNPGLNGSPGLPGEKGNPGFPGGQGLSGLPGPKGERGFPGSSGPKGEAGRTSEGAKGEPGNPGRNGLNGLPGMKGDVGLPGRDGFNGVKGEAGEPGSGIRGQKGERGLDGLPGSAGLRGLPGNDGLPGANGGKGEPGERGLDGLPGQQGRPGFPGAKGDSGLPGGFGTKGSKGERGLDGFPGSPGPKGSSGLPGLQGPPGDSGLNGLKGDRGLPGLPAVLDITNLPPGPKGDKGSPGIPGNSGRDGLPGERGPSGPPGFPGAKGDNGLNGLPGMKGEVGPTGLNGIRGQDGLPGTRGQPGEPGQPGPAGFTPPSGFLMVRHSQDKVIPQCPDRMVKMWEGYSLLYIEGNERSQHQDLGWAGSCLQRFSTMPFLFCNTNNVCNYANRNDKSYWLATNAPLPMMPVTDSTIARYISRCVVCDAPSNVIAVHSQTLQIPECPIGWSGLWIGYSFAMHTGAGPSGGGQSLSSPGSCLEDFRATPFIECNGDGGNCHFYSNKYSFWLTTIDRNSQFSNPIPQTLKAGNLRDRVSRCQVCTKNV | collagen alpha-2(IV) chain-like [Mizuhopecten yessoensis] | XP_021346726.1 /0.0 | Gly(24.5%)；Pro(12.6%) |
| **CL4310.Contig5** | 2.7547 | AILVDLEPGTMDSVR;FPGQLNADLR;ISEQFTAMFR;LHFFMPGFAPLTSR | 1 | MREIVHIQAGQCGNQIGAKFWEVISDEHGIDPTGTYHGDSDLQLERINVYYNEATGGKYVPRAILVDLEPGTMDSVRSGPFGQIFRPDNFVFGQSGAGNNWAKGHYTEGAELVDSVLDVVRKEAESCDCLQGFQLTHSLGGGTGSGMGTLLISKIREEYPDRIMNTFSVVPSPKVSDTVVEPYNATLSVHQLVENTDETYCIDNEALYDICFRTLKLTTPTYGDLNHLVSATMSGVTTCLRFPGQLNADLRKLAVNMVPFPRLHFFMPGFAPLTSRGSQQYRALTVPELTQQMFDAKNMMAACDPRHGRYLTVAAMFRGRMSMKEVDEQMLNVQNKNSSYFVEWIPNNVKTAVCDIPPRGLKMSATFVGNSTAIQELFKRISEQFTAMFRRKAFLHWYTGEGMDEMEFTEAESNMNDLVSEYQQYQDATAEEEGEFEEEGEEEDA | tubulin beta-4B chain isoform X1 [Heterocephalus glaber] | XP_004848801.1 /0.0 | Tubulin (SM000864);Tubulin_C(SM000865) |
| **CL4206.Contig1** | 2.7547 | EVMGHESELFR | 1 | MAGLVKAKKYDWKDSNMALFGSDLERQVKKDSAMGEPAWNGAGEAVGLKIWRVVKFQITDWPEEDYGKFYDGDSYIILNTYKKNEDSEELCYDLHFWIGKHSTQDEYGTAAYKTVELDTFLDDKAVQHREVMGHESELFRSYFKSIMTMQGGADTGFKHVLPEEYRPRLLHFSGKRNIQIKEVPYLQSSLKSGDVFILDMGMQVYQWNGAESSGMERIKAAQYLQEMESERPSCSTTVLDESGISKAHRFYEAIPEEGEDDSGDSDDEDVDGPDEKKLLRLSDAGGSVQFAEVKSDGITMDDMGEDDVFVLDTRKELFVWVGAKASVEEKRNGMGYAHRYLMDKARHYKPVSVVSEKVGRERVEVAISA | gelsolin-like protein 2 [Crassostrea virginica] | XP_022299451.1/9e-151 | GEL（SM000262） |
| **CL4067.Contig1** | 2.8558 | VVGATIAQLLR | 1 | VEYSDKMWLVVFALLAVNSRTLVSGWLFWYSEKCYDHIGCFSNRPPFNRAGMRVPQSPDHIQIQFYLFTRHNQEIPILIDPYSPDSIMQSTFDGTKPSIFVIHGYSDSGAPSSSSWQRRMTQEFLTKDDLNVIVVDWEKGADSMIYQQSAANTRVVGATIAQLLRTLVSNGPTSYDMFTLVGHSLGSHIAGYAGAYVNGTIARIFGLDPAGLSFENSEPEARLNPTDAKYVEAIHTDQESLTALGFGLEKAIGHADFYPNGGQNQPGCERNSRTQLLQLIQGRIGDLSNTIACSHMRVLDLFTESINSNCVFTGTSCSSLTDYESGQCLECDNGCADMGYNGRVRGQGRYYFDTNTSPPYCYTEK | inactive pancreatic lipase-related protein 1-like [Crassostrea virginica] | XP_022340243.1 /8e-125 | Lipase(PF00151) |
| **CL3814.Contig2** | 3.8972 | AEDPVPEEDYEYTSVR | 1 | MPPPIAGHTPRKVTLNKFGGGTTSFGQSFQSNKKSSTTWQPVPAPAGGSMMNRVQDSLDSALSPTSPPQGYYQQQYQQPQQQQYRPPPQQQQYRPPPQQYQSPSQQQYQPPPQQQYQPPPQQYQPPQNQYQPQPQQEPYTPTYQTVGDLQPDYVRAEDPVPEEDYEYTSVRDRKKQFIETRQDAPLIKRGKKKFVPPVAAAYQSFGTDYSSPQPKQVEQPRFPPAPKPVPPPVNRAPEPVDQQDGPKPWAGSLRSESGGPKLWELEDKEYIMPSQLEARQQQQQQQRQSRGQTRQQRQPPAVSPKPISKGTNQIKVAVAPPQQSPARQISVRTSSTVSKQQPQQSQQQGDRDWNQSYVYKMVKEETKRETQMYPGQAPITTQTYSSKTYQSGQPAQEDTYGISDF | PDZ domain-containing protein-1 [Mytilus coruscus] | AKS48171.1 /4e-91 | Gln(19.5%)；Pro(15.6% ) |
| **CL3802.Contig3** | 2.7547 | IANFGALR | 1 | EDKYEMCLILEFLAGGELFDRIAAEDYKMTECEVINYMRQVCDGLKHMHENSIVHLDVKPENVMCTTKNSNEVKMIDFGLATKLNPDEVVKVTTATAEFAAPEIVDREPVGFYTDMWAVGVLAYVLLSGLSPFAGEDDLETLANVQRCDWEFADDAFSNISPEAKDFIRQLLIRQPQRRMTVHECLDHPWLKGDLSSRTTRIPSSRYDNIRKKLKAKYADWPAPNPAIGRIANFGALRKNRPKEFSIFDAWFDRKEAAPRFIRKPRTVITAEGQTAKFDCKIIGASPPIVTWSFDNSVLSQSVKYMQKYRGNEYELKISRIKMSDKGVYTVIAENSFGKKEEHGTLKVEANPDLPKIPSSRDTTPLRRSRRPSMSPAPEVKPIEEAPRISFGLRPRLIQAGTEFKLLTCVQSTPTPKVTWSKDGKDISKDPHYMCSYSGGVATIEVQGARMADTGVYTIHAVNELGEHETSSKVVVEDRAHDFDKADIFKSHKQSRSARRTKSGFNFEDSSSSYTETSTSSSSTKTSRSSRRFESSTEESYSSSSSRRSNRRKEIEEPSYEAPEFTTQLSPLILDEGDRLKLTCTVKGRPDPEVEWFYNGQLMQSDDAIKITAIGGVHTLVINSCILDDDGSYVCKAKNPGGQASTRTTVQVNEKKSLSSSKPDFIEHPAGVSLEDGDQATIAAKISGNPEVLWYRGKELIKDSADFQYKQDGNVFKLIIAEVFPDDTGVYKCIASNTAGSVTSSFYIKVEEPDIAPSGPVFVSHPKSQSLEEGTAFVASCTLDKADSVQWSKDGKDVESSERFKFSQDGNTFTFEIPAALATDSGEYTVTAKNSTGSSQWTFTLSVA | twitchin-like protein-1 [Mytilus coruscus] | AKS48140.1 /0.0 | S_TKc(SM000220);IGc2(SM000408);IG(SM000409) |
| **CL3650.Contig2** | 2.7547 | YGGITCPNTCEDYAR | 1 | MKCKLLALAVPIVFLFGCIIAEISVKCLQCICNVESGCNQKENCNSNGGYEACGAFHIYEAYWIDCGKPGSSFKNCTLDYECSKDCVMKYMKRYGGITCPNTCEDYARMHNGGPYGCQNNALSSIKKNFAVIPRVAFLI | lysozyme [Mytilus galloprovincialis] | OPL33781.1/1e-37 | Signal peptide(1-21)/Destabilase（PF05497） |
| **CL3476.Contig1** | 2.4307 | DGFIDVGDLR | 1 | MSGKEDDAQAQKARAATSSVLTKFNQKQIQEMKEAFTMIDQNRDGFIDVGDLREMYSSLGAVPQDSVMKEMLAEAPGPLNFTMFLSLFSEKLSGTDPENTIQNAFSMFDDAGKGYLPEAYIKDLLQNMGDNFTQDEMRQTFKEAPIEGGKFDYNKFTAIIKGSNVED | myosin regulatory light chain B, smooth adductor muscle-like isoform X3 [Crassostrea virginica] | XP_022340190.1/8e-89 | EFh（SM000054） |
| **CL330.Contig2** | 2.3052 | NTLINYCCR | 1 | MLPLSLIVLIIVSPAYSVQWPKGTYTLPKPNTGCPLHWKNGWRKQDNEDSHPENSHSSRLNLKGHFGRNTILHYCTKDEHVVEGTAYWPKGNYCILKQGLSCPRGFKTGSIRWDDEDSRNKNAQDGVLPSGSYGKNTLINYCCRADGSHNIEIQLPTKTPFYLHEFKAGCQKVKGMNVQLEWVQMDDENSRNRNWASGSHPRIGRKGSLTTLYYCYYFK | apextrin-like protein [Mytilus galloprovincialis] | AEK10749.1/5e-75 | Signal peptide(1-17)/ApeC (PF16977) |
| **CL3196.Contig1** | 3.8972 | QAVSTSVTQFLSDQISR | 1 | MDFVKYLMVPAFLGSIFHNTVNGELTSSLKQAVSTSVTQFLSDQISRVKISPISIAGIILTVTGTFTANAKKVYVYIEIVKATNQVGYCRAIIGQMTIGFKPSQPSGSTEGPVNLSSSLIGELKKKLAEEICKNMKNTLNSNPLFPPP | — | — | Ser(10.8%) |
| **CL3182.Contig2** | 3.8972 | AADTIANILSANR | 1 | MNLSFHVFFFSLLLAGDVLASGSDKFTSTRSGVYFYGRAADTIANILSANRKQWNGEKCQDARPSDCGELDKRVCPSGIYTIYPDKSSGFDVFCDMEKDGGGWTVFQRRQNGLVNFYRDWEAYKEGFGSLRGEFWLGNDHINKITSQEIYTLRVDLVDFKNNHKYAKYSKFSVGPLNSEYKLDVGGYSGNAGNDFGGHDGQKFSTKDKDNDVYSGNCAESYKGGWWYSSCHSSNLNGLYLRGNHTSFADGINWKTWTGYNYSLKATTMMIKRTKLF | fibrinogen C domain-containing protein 1-B-like [Lingula anatina] | XP_013401135.1 /2e-80 | Signal peptide(1-20)/FBG(SM000186) |
| **CL2840.Contig4** | 2.3637 | ASSYLDDIYYPEPIVR;GFYDTTR;GFYDTTREENEIR;LVVYPISER;VEVVTPR | 1 | MTVRRSRFQSVPPGYFSSTKGHSSLKRWYPTTTRASSYLDDIYYPEPIVRSRGFYDTTREENEIRRDVNHELLYTSNLVDDTYDIANKSRNRDQMLLREATRALPSTTTENKDVGKRTVSLTPARRFQAPLSSLYSRNPTPYPKLTPPMYRATTNIQPLGILTKLDWTSPLRKYEMGASLPELENGDDVSEYSTYAAPTETESVRSSPERDYEEGIRPRTRPKRKIRTVYRRMPRRTVRASKNVKYYPYLDTESVVSDDVGTINGEYMDDDDYQSTYCSEVGDGGDYYYVDKERSPSPLEYVPFAPRHKINLDDDEYKCQVPSVAEMAVSHFVPPKKDPDFDPDKSRIKNDLVFHDVVSQTASKARDALKNVNLDEYDSASLVISVEGEYKDPVKGENMGFQYIARPLALKGPMLAGPAVYASTASDSAFNRYMDDMRSFRAQIRHRLETGYKTLSNVSSNYRDHSYPILASSHRSYSRALSPPKSSSPAGRSVSPVRVPHTSYTAHSLPISNLRKRLEEIESKYKTMGYEKRLDDIPDRLVVYPISERASIKPMTATENLPGHTKTVELYEGSAKGNELSTLEKINIKAALVGNRVEVVTPRKRKPRSTYAANKMRELKRDEREMEVEAVPVQSTTSLKASTVSLVRY | RS-rich protein-2 [Mytilus coruscus] | AKS48164.1/1e-36 | Ser(10.6%) |
| **CL2825.Contig1** | 3.8972 | AGFAGDDAPR;AVFPSIVGRPR;DLTDYLMK;GYSFTTTAER;GYSFTTTAEREIVR;HQGVMVGMGQK;IWHHTFYNELR;QEYDESGPSIVHR;SYELPDGQVITIGNER;TTGIVLDSGDGVTHTVPIYEGYALPHAIMR;VAPEEHPVLLTEAPLNPK | 1 | MSDDDVAALVIDNGSGMCKAGFAGDDAPRAVFPSIVGRPRHQGVMVGMGQKDSYVGDEAQSKRGILTLKYPIEHGIVTNWDDMEKIWHHTFYNELRVAPEEHPVLLTEAPLNPKANREKMTQIMFETFNSPAMYVAIQAVLSLYASGRTTGIVLDSGDGVTHTVPIYEGYALPHAIMRLDLAGRDLTDYLMKILTERGYSFTTTAEREIVRDIKEKLCYVALDFEQEMSTAASSSSLEKSYELPDGQVITIGNERFRCPESLFQPSFLGMETAGIHETTYNSIMKCDVDIRKDLYANIVMSGGTTMFPGIADRMQKEVTALAPQTMKIKVIAPPERKYSVWIGGSILASLSTFQQMWISKQEYDESGPSIVHRKCF | actin A1 [Haliotis iris] | AAX19286.1/0.0 | ACTIN（SM000268） |
| **CL2758.Contig1** | 2.6077 | SAVVAAER | 1 | MATNILCFGFLISTTILKLVNGAAPISAKGGGSNSFVFSAKNKFSFSNPSPPTSSQGQGFSSSSSSSSSSSNAGSGGGFSPNNNQNQWKQTLPINYEPNKQGNNQQPNQQNGNQLSSQQQQQQQQQQQQSGQQHQQQTQMQHQNMQMHPNNQFGQPQTKPMNQNQKFNSPGQMGSGQQWQPMGFGQQQQQQPQNPQHQQQNTFSNPQQPQNGFSNPQQNSFSNPQQHQLSNMNTGQTMFQQPPNQQQKQQFGQPNQIQNSHMTPFGQQQQQQQQQQQRNQFNKFGQPQSGSNMNVLSQGGQPNNGMPPYQGMPLTANNQQGFQGQRPQQQQQSGHMTQYFPPHQSQQTNQMHGPGQNQGHQGQMPMNGNMPMQGSPQNMQMGSHQQPMNGQLPAHMQGGPQSMPHPSGSSAPAHQQMGRPHPPIGGAINPPMGGAINPPHGGPQMGGPGHSPHGGPYNPNQGGPGYPPQGHGPRPGHPNHNVAVIDHPRKTTTTPKPQTSTIDGEGMVCMTTADCEIGCCFNATGQLLDTTTYGAGGPKEGRASGKCFIRKPGLGDVCDDLCACTMGHDCYRRYVPVYPKPGQKTAPVIDPEAAPKPQRTCVRSAVVAAERIAFWSCYFDVSCSGPLP | extensin-like [Mizuhopecten yessoensis] | XP_021354369.1 /3e-34 | Signal peptide(1-22)/Gln(19.6%);Gly(12.1%);Pro(11.6%) |
| **CL2714.Contig1** | 3.8972 | QQYDMIELAER | 1 | MSDTEETTQPENEAKLAMEEAAARKAEKIAMEIAEFEEQRREEKAKEEEELAMLREKREQRKIERAEEEKRLAQLRIEEEARRKQEEKERQQKKAEDEQRRKEERERKRKEQEERLKLVKKPNFVITKRADGGDDERRKKAEQKAEEMQKSKEQLEQEKRAILAQRIQELNIDGLKSDGLIQKAKDLHEKLHNLMGEQYDLEQKFKRQQYDMIELAERARQMNKGKNRSTMGVKVDESFDRLADKFINAPPKIQLCSKYERHTDNRSYNDRMNLFEEFSKPKPPPEIIRKGAQTSGAEDGEEEEEE | troponin T, skeletal muscle-like isoform X1 [Mizuhopecten yessoensis] | PVD33201.1/3e-90 | Troponin(PF00992) |
| **CL2603.Contig1** | 3.8972 | EFEFQSGDLWEVR | 1 | MSDKTRANKSGLGYAVEKKMEDNYDREEAAGTPTHVVNWVNGILGSEHDPIPGTDWKSICNHLRDGVALCKMVNILLKKDGKSPITFQKKVMSPFVAMTNIENFNKGCLDYGLDREFEFQSGDLWEVRKGPFLNVINCIHSLGFVANKKNVVPGYTGEIRKYLDNE | transgelin-like protein-3 [Mytilus coruscus] | AKS48154.1/8e-109 | CH(SM000033) |
| **CL249.Contig2** | 2.2570 | EPFPNVNR | 1 | RNAHATSFLVGAMAAGTLYTYPDNFRAFKAQIAAQFSGTDIKLASGFKFGETNQSKDFLSKFPLGKVPAFVSSKGDCIFESNAIAYYLGNPQLRGTSDKDASLVLQWINFGDNEILPSACTWVYPCLGIVQYNKQETEKAREQIKKALSVLNNYLLTRTYLVGERITQADISLACNMLSLYKYVLDPKFREPFPNVNRWFTTVVNQPQFKKVVGDFKFCEKMAEFDSKKYQELHGKGDGGKKDKKKEEKKQDKPKQQPQAKKEKPKKEEEEEDDGFPKEKEQKDPFGQLPRGNFNMDEFKREYSNKDTIKEALPYFWKNFEKDNYSIWFCNYKYNDELTRIFMTCNLVGGFYQRIDKMRKNAFGSMCVFGEDNNNSISGIWFWKGQDLAFKLSPDWQIDYESYDWKKLDPTSAETKKMVQEYFAWEGDFGGKKFNQGKIFK | elongation factor 1-gamma-like [Crassostrea virginica] | XP_022332141.1/0.0 | GST_N（PF02798）；GST_C_2 (PF13410)；EF1G（SM001183） |
| **CL1827.Contig4** | 2.4307 | VTVPVLWDK | 1 | SIKCKHMFQFLTLIASYIYIYIFAWNCLASCSWKYKMSKAFDSVIGIKGEFKRAQSAFRSFITADGSSGFPAEANRYHLYVSLACPWAHRTLIVRKLKGLEDVISTTVVDWNMGDRGWRFTDKVERCSLDTLNGKDYLREVYQIIDPNYQGRVTVPVLWDKKQSTIVNNESSEIIRMLNKEFNAFCKTKEQAVLDFYPEKLRSKIDDINSWIYNDINNGVYKSGFARSQEAYDAAVTALFSALERVEEILSKNRYLTGDRLTEADIRLFVTLIRFDKVYHGHFKCNKKRIIDYPNIWGHTKEIYQLDGIKDTVDFHHITWHYMFSHDSINPYRIISIGPDLGLDEPHGRGNM | Glutathionyl-hydroquinone reductase YqjG [Stylophora pistillata] | PFX30627.1/ 4e-156 | GST_N_2 (PF13409);GST_C_2 (PF13410) |
| **CL1815.Contig1** | 2.7547 | DIVQFVPFR | 1 | LTSFKLQRTPSFLEYLYGGMQINFTVGIDFTASNGDPNSSNSLHYINPYQPNEYQQAIQAVGNVCQDYDTDKMFPALGFGARIPPNNEVSMEFALNFNATNPYCAGVQGILEAYTNCIRQIRLYGPTNVAPIIYHVARFADAAQKEEATKGAHSYFTLLLLTDGVITDMNDTRQAIVNASGLPMSLIIIGVGDADFADMEFLDGDGGVLKAPNGQPAQRDIVQFVPFRDFKRVSAAQLAKHVLAEVPQQVVKYYTMRQIMPNPPRQAQQ | copine-3-like [Crassostrea virginica] | XP_022342060.1 /9e-143 | VWA（SM000327） |
| **CL1396.Contig1** | 2.2109 | TLIEDAESR | 1 | KRSTITSVTSLVSTVRQYIALSHVFRTTIINHILSPSSLRVFHNHPCVKEQDREGLENYNPSQQKAITIASKIAIEPPVQNRIVLLQGPPGTGKSFTIVGIIKKIFERSNGRCKICLCAPSNSAVDELIKRLITERRKMIEEKKNPFGLVRIGKQENMHQDVLKYTFQEILKSNLDVELKRKMRANGSSEFKELNTLTSRIQRLQLDMEEKTVRLQNIQAGKLMSEITKLQKKKNDMEAEIQQLNKDVKLTRDEEENVKKNILIKSHIVCGTLSSFGHHQYMNILAPLQRENQIRRSYFNCIIVDEASQATELDTLIPLQYGTSKMILVGDPEQLPPTVLSQKCALKSFGQSLFERFFNHFKHEEVNPVLFLDTQYRMHPDIAYFPSKFVYLGALKTDESVKGRCNKLQLRPYVVFDMQEGQEVTTRRGAIINSIEAEFTIELCEFLIKRGRLQQKEIGIIAPYQQQKKIIKEGLKSSQLDHVEVNTVDGFQGREKEVIILSCTRAKNSSGGIGFLANSKRMNVALTRGKSALYVIGCLDSLKKGDSAWKTLIEDAESRHVITKVDTQGSHEMFNACLKTDARLGRQCVS | helicase senataxin [Mizuhopecten yessoensis] | OWF55838.1/0.0 | AAA (SM000382) |
| **CL1319.Contig2** | 3.8972 | TDIAGTTVVGVLESTPAER | 1 | ATNKGKVEATDKVKIAQKTESVKTKSQTSNKAKTTQSKVESKTVKKSDGVVVENGLDNEVYRETQEKEEVSFDQMSNIKNKLESNKAEDEDVIYRERKKSVHEEIADAEGGEYENEPVRNPDVVRESDKDIGAELPEVGTAKNILEKFKQIETTPKMFKREITPPEVRATGKVEYVSEPKPGIEIEAQKIEGGVFENQPVQEEDVVHSYDTVEEALPEKGYAKNILEKFKQIGTTSGESTPQRRELTPDRNTKYEYVSEPRAVLEKYEGKVESGVFESQPAGDLPDIIRSGMSNEEVLPEKGTARNLVSRYKEISDQAASPKASERKRELTPDKTGKVEYVSEPRSVLPKYEGKSEVGVFESRPEDKEEIVKSDMQLEDILPERGSAKNIAAKFREIESSKSPSPSSPKPRFKEVTPPRTDIAGTTVVGVLESTPAERGDIVKASEGIELKEEEMPVRGMAKDIANKFKQYQQSAPASSRGKKEFTPPPEGGVFENTPKPSLQVEVRQAESGVLESTPLKRDDVAREENYSAGDVEFPEEGYAKNMVSKWKQMESKSGNSTPERKQRDFTPPRDYPMSPKSPAGVDSSVKPSDLPGQYQEQKGYGGVYENKPQHLVGVVREGDGDIAEELPEKDAAKSMVQKFKAIQEQAKKAEEAPKPKSRKPKGSFVPVQLDKCAACQKTVYAMEKMEMNKNIYHRACFKCSHCKCVLTPKTFSMNEGVIFCTNHFKQLFARKGNYDEGFGRQQYKKKWGTADSANEEQ | LIM domain-containing protein 2 [Mizuhopecten yessoensis] | OWF54964.1/0.0 | Internal repeat；LIM（SM000132） |
| **CL105.Contig2** | 2.8558 | SQIAIPAQGMIEFR | 1 | RQNRKGCTMSDRESGDEQQEHQGQGSGEQELATKTLQIQSKRFYLDVKQNRRGRFIKIAEVGAGGKKSRLLLAMSTAAEFRDYLTDFSEHYASLDNLPEDGKLKSETMIKDNRRYYLDLKENQRGRFLRVAQTRPRGGPRSQIAIPAQGMIEFRDALTDLLDEFGTDDHGESDEGQGELPESKYLRVENKVFYFDVGSNRRGVYLRISEVRSNYRTAVTIPERSWGRFRDMLSEFVEGSSQSGTAAEAPKETK | transcriptional activator protein Pur-beta-like isoform X2 [Mizuhopecten yessoensis] | XP_021353033.1/5e-148 | PUR(SM000712) |
| **CL1023.Contig5** | 2.8558 | DMDDLVEPSR;FELPYSTIR | 1 | NSLVSVLAVDKSVILLRTGNDVTVKDVIDELNSYDRTFFPRFGDWDYWFPRPISGIDASSVFKDMGVHVLTDSLLYKHSEVVRRNQLSSGSMAKGMGGGASFAMDAPEMSRDMDDLVEPSRTRKNFPETWMWTNTMTGASGISIINAKAPDTITEWVTSAFAVNPTSGLGVSSDLANLTIFQKFFMRFELPYSTIRGEIVIVQIT | protease inhibitor-like protein-1 [Mytilus coruscus] | ALA16013.1 /5e-67 | A2M(SM001360) |
