# Supplementary material for: Microstructure and in-depth proteomic analysis of Perna viridis shell
Source: PLoS One. 2019 Jul 19;14(7):e0219699. doi: 10.1371/journal.pone.0219699 (PMC6641155; doi:10.1371/journal.pone.0219699)
Supplement: S4 Table — (DOCX) [file pone.0219699.s009.docx]

**S4 Table**

| **matched unigene** | **score** | **matched peptides** | **number of matched peptides** | **protein sequence** | **Homologous protein [species]** | **Homologous ID / E-value** | **Domain and signal peptide(SP)** |
| --- | --- | --- | --- | --- | --- | --- | --- |
| **Unigene10181** | 247.6202 | ADMAEKNLTAVR;ANLEQEVYR;AQLEAERDNLAAALR;AQLQILCDDLK;AQLQILCDDLKR;AQSMIDEAEQR;CDELGAENAQLR;DLDSDVSTSTR;DLELASAQFEATEANMR;DLENELEADQR;DLENELEADQRR;DNLAAALR;DTEEALR;DTEEALRDAEAK;EKDEEIDSIR;ELEDALDSER;ELEGALDNANR;ELELQLEETQR;ENGQLQAALR;EVVQQADDDR;EVVQQADDDRR;GSSPGTQNR;GSSPGTQNRLEGR;HQEALNDLTDQLEHMGK;HTYNVYR;IAIQQELEDAR;IDALEGSNGR;INVDDLTR;IQELEDNCEQLR;IRDLENELEADQR;IRELEDALDSER;IVRHTYNVYR;KAQSMIDEAEQR;KNAENELGEVSAR;KQLEIEIR;LAAAQAALNQLR;LADELRQEQENYK;LEEAEAFAQR;LEEAEAFAQREGK;LEEAIGSSTTFSEVSR;LQAEVNR;LTEVQLQVTALTNDKR;LTQENFDLQHQVQELDGANAGLAK;MEADIAAMQSDLDDALNAQR;MIEEAEDVANITMNK;NAENELGEVSAR;NQLSISER;NRELELQLEETQR;QEELEEIKR;QEQENYK;QINELTALK;QLDDAR;QLDDARNQLSISER;QLEIEIR;QLENDNAALQK;QNLQVQLAALQSDYDNLNAR;QSAELTFQLDQLSDR;QVAELTSLTDQLTMK;RCDELGAENAQLR;RMEADIAAMQSDLDDALNAQR;SLLEHAER;TLVEVETR;YEEESEAASNLR;YETDIR | 64 | MSLLRDLDSDVSTSTRIVRHTYNVYRGSSPGTQNRLEGRIRELEDALDSERELRLRYEKQSAELTFQLDQLSDRLEEAIGSSTTFSEVSRKREAEVSKVRKDLELASAQFEATEANMRRRHQEALNDLTDQLEHMGKAKARAEKEKNQLIIEIDSLQGINDGLQKAKMSADSKIDALEGSNGRLKINVDDLTRQLNDANSAKARLTQENFDLQHQVQELDGANAGLAKAKAQLQILCDDLKRNLDDESRQRQNLQVQLAALQSDYDNLNARYEEESEAASNLRAQLSKVNADYAALKTKYDKELIAKQEELEEIKRRLSVRIQELEDNCEQLRTRCNSLEKTKNKLTAEIREITIELENTQIIVQDLTKRNRQLENDNAALQKRCDELGAENAQLRNDKANLEQEVYRLKVANAELAEKNGNLERENGQLQAALREAQNELKSANRQINELTALKAQLEAERDNLAAALRDTEEALRDAEAKLAAAQAALNQLRAEMEQRLREKDEEIDSIRKSSARAIDELQRTLVEVETRYKTEITRIKKKYETDIRELEGALDNANRANAEYLKQIKSLQNRNRELELQLEETQRQLDDARNQLSISERKRIAIQQELEDARSLLEHAERARKNAENELGEVSARLTEVQLQVTALTNDKRRMEADIAAMQSDLDDALNAQRAAEERADRLQAEVNRLADELRQEQENYKNAESLRKQLEIEIREITVRLEEAEAFAQREGKRQIAKLQARIRDLENELEADQRRLREAAASARKFERQWKEVVQQADDDRRQVAELTSLTDQLTMKCKTYKRMIEEAEDVANITMNKYRKAQSMIDEAEQRADMAEKNLTAVRRSRSMSVSREVTRVVRV | paramyosin-like isoform X3 [Crassostrea virginica] | XP_022322570.1/0.0 | Myosin_tail_1(PF01576) |
| **CL1886.Contig2** | 89.0478 | ASVGGGSGGTVYTR;DIQNEYDNKVDQIR;ELAALAYR;EVNLDGWK;EVVYTFR;EYESMQSEHTMETVK;FANYIEK;FITLENTSSQR;FLEAQNR;GDMESYYNLK;GSAQEAGINDLVFR;IDLNNETLNHLDAENR;IDLNNETLNHLDAENRR;LADLEAR;LEIQNSSLQEEMNGLR;LKEEITNLR;LLESEESR;LNQQLSDYESEINMLR;NAQLEAQYNSLLR;NVIEQSMNTQSR;QTLEEEMEFLK;SMYEQELAEAR;SSIGPSMR;SSTVVNR;SSTVVNRSSIGPSMR;TISSLETER;TVEYGMGR | 27 | MSKSSERITEKRTVITSSSSNYDDGDDSIYYKSGIQPRSSTVVNRSSIGPSMRASVGGGSGGTVYTRTVEYGMGRSSGLGNLSPGSYEKVSNTGVMTVKSSREKEKKDMQDLNERFANYIEKVRFLEAQNRKLAGELEHLKTKWGKETSAIKSMYEQELAEARKLIDDLTRDKNKLEIQNSSLQEEMNGLRRQMDDLKKYHALDQEQINKLNQQLSDYESEINMLRRTISSLETERARDKDRINKLQGEVDRLRIDLNNETLNHLDAENRRQTLEEEMEFLKKVHEQELKELAALAYRDTTEENREFWKSELSQAIRDIQNEYDNKVDQIRGDMESYYNLKVQEFRTGATKQNMEVTHVKEENKKLVKSISDLKGRLADLEARNAQLEAQYNSLLREYESMQSEHTMETVKLKEEITNLRAEMEAILVELQSLMDAKLSLELEIAAYRKLLESEESRVGMRNVIEQSMNTQSRGAAQLSEMITEYETKGDSHSSMKMMRGEVSAKTTYQKTSTGPVSIAEVNPEGKFITLENTSSQRREVNLDGWKIRRELDGQREVVYTFRNFTLKPHKSVKIFARGSAQEAGINDLVFRDEETWGVGSQVSTCLVNEKGEEKATHRQRTAYN | filament-like protein-2 [Mytilus coruscus] | AKS48133.1 /0.0 | Filament(SM001391);LTD(PF00932) |
| **Unigene2367** | 95.1889 | ACPAGTIYSASQCQCK;AIGSASGLLASK;ASLLLQSAR;AVFGGNSR;CLIPGTLHYR;DISNGGLAFDHSHIYLR;DNPMTIGLCSGK;GGSTSASGSVSAGGSVGSSR;GLGASSGFSSAGAAASASGLGSASLGSTR;GLGSVSVTTGGLGQDLR;GLNGIGGSVTSSLTSK;HGSISGSGISSLDSR;LAVVGDR;LLTALTK;LSFDNDER;LTVGLGNAR;LVQCFYLGGK;MYLWGFQSK;QQIAEAVK;SGSSSGSSGSSGSSGSGGSSGSSSFGSSTSGSDSR;SRPEPIISNCGPNGDSSVEIVVYR;TGGNLEIR;TSDNPDTVYFNEK;YGYLAPEHGGLR;YKLPEQGGTLNILSQK | 25 | GAGAGAGSGVGLGFGVGAGAGAGSGTGLGVGAGAGAGAGSGIGQGIGIGAAGAGSGIGSGAGAGIGVGSGIGFGAGAGAGAGAGSGIGIGVGAGAGAGAGAGLGIGSGIGAGAGAGAGAGSGVEIGMGAGIGAGAGSGFGQGIGATSGLGSSSSGSWGSAGSGESSCSCASQGPKSETIQASAKLAVVGDRNVLIAKMPRAKVVDSDGLGDFLLEFPNLTIQPTLTEKIASPAELFGMNMNKGTHSTDDSDILVVALLPKGSKLGSKMFQPKSSGSGSSSGSSSSSGTGSSSGSSSSSASTNDNPSVSVDTSQDDGKLKIKIKAENVKDSTASIVKNQLDKIKSDEISVDTSKDDGKLKIKIKAKVDDLSKSDINALSSESSYSGSSSGSGSSGGSGSAGGSGSAGGLGSAGGSGSASGSGSAGGSSAAGGWGSNGGLGFSGDLGSAGSSTSAGGSGAASGSTGGSGSVGISGSISVSGSTGGSGINVGLGSAGALGTAGLSGSVGGSKLTVGLGNARGGSTSASGSVSAGGSVGSSRGSGSARAIGSASGLLASKIKASLLLQSARGLGASSGFSSAGAAASASGLGSASLGSTRGLGSVSVTTGGLGQDLRSGSSSGSSGSSGSSGSGGSSGSSSFGSSTSGSDSRLKIEKSKDDGKIKYKIKTVKGKGDSKTKSEYKLVVDKSDGSSSKVKVKFGKSDQDRSSSKIKHGSISGSGISSLDSRGKSYRREEDGDKWKLKSSGSRGLNGIGGSVTSSLTSKKQQIAEAVKVLKALKASQKASNALTAGVSLKAKLPDTRYKLPEQGGTLNILSQKLTKDIKFKQSRDNDERKKLSYKLSFDNDERKSRNRLLTALTKTSTNTVVPKTTGGAKVPNLEGLCLIYLGAFVDAIGYAPLPGQCHKLVQCFYLGGKLKAVARDCPAGMFWDQNTLSCRPPGDVLCFEDKCLIPGTLHYRRNGGCNCFYKCRDGISEPSCCPKGFRYDDDKGCVPAFGSLACDDECETPTTLTTQVTVSSCPSLPDPNNKYGYLAPEHGGLRIRACPAGTIYSASQCQCKSNMNGSGAMRGSLRKQYRQCSAEFNINFDDGFKDISNGGLAFDHSHIYLRKGKAVFGGNSRMYLWGFQSKYLGKTFAIKTRVKVNKGAGRSRPEPIISNCGPNGDSSVEIVVYRGKLIFKAKTSDNPDTVYFNEKYDDDKWMDITYYYDGNYFGGSCNGRSFKQRTGGNLEIRDNPMTIGLCSGKNPGFHGEIDELEIYTACIPKGF | matrix protein-1 [Mytilus coruscus] | AKS48137.1 /0.0 | ChtBD2(SM000494);Laminin_G_3 (PF13385) |
| **Unigene39912** | 62.9260 | GGPVIALETLADAPSEPR;ICDSTQTIASITDLSPHSR;INLVEPDYPYIVVVR;LADMEQATGELVR;LEWTSDFLDDDTTHVFR;QDMFSQAENK;QLQIMIIR;SLPIEGATQR;TPDLIPSR;VGADVFETVITK;VGLGDAAITTYR;VTWTPPPPQPGDGIIR;WELVEVFESNK;WELVEVFESNKK;YTDVSEHR | 15 | MLSIGLILVSLVSVSHGQWRQDMFSQAENKLRNIVQNGQILLDFIYQERQKHGGGNMTSGSMMSQNLAYSSFINDVEVRLADMEQATGELVRIMRTCPDAPLAPPSPTNVIVESTTTDNVSSIVVKWDPPFNPPENMQYKVYFVPVDINGMQTAGEVVFRICDSTQTIASITDLSPHSRYRIRVGAVAGSIAESTSVPLNVKTPDLIPSRVQNVMVKSSTPNTITLMWNPPSTMGDLVSYEIYYEENPINKMHVTVSPPENTFTIKDLSEGTTYKFEVSAKSDNGEGIRSLPIEGATQRFIPRAPQSFTGVALNKTAVKVTWTPPPPQPGDGIIRGYLINYTDVRYTDVSEHRVGADVFETVITKLTPAQVYYFRAFAFTKKSVGRGGPVIALETLADAPSEPRQLQIMIIREEPPKIGLTWLPPLHTYGNLLNYTLIWGVQNGANRTEYISPTRLEWTSDFLDDDTTHVFRLAAVNKVGLGDAAITTYRTPKKVPIIPPNVKVKRVTFENNGTTILNVTWDNPVVAVDGFRILYRKFQLVYSGRWELVEVFESNKKFVRINLVEPDYPYIVVVRGIPKGQIFNQYNMGGGQHMSRPNSHIAQSFGGASPPI | shell protein-6 [Mytilus coruscus] | AKI87977.1 /0.0 | Signal peptide(1-17)/FN3(SM000060) |
| **CL955.Contig7** | 50.2367 | ALDSMQASLEAEAK;AQYEESQDSVDALR;CNMISGEVEELR;DLEESTLQHEAQISSLR;DTTDLALVAPGLK;EEEFENTR;EIAFQADEDR;ELEDLGER;ELEGELDSEQR;EQIEEEQEGK;ERNSLQSIIDELR;ESYNLAER;GAENELFEANDR;GQLEISNVR;GTSPAVLEGIASQPVLYSK;IEELEEELEAER;KGAENELFEANDR;KVQQELEDAEER;LAEKEEEFENTR;LDEAGGATSAQVELNK;LEEAESQALK;LQGELEDLGIDVER;NLNADLDAAK;NSLQSIIDELR;QNVEEETR;QVEEAEEIAAINLAK;QVEEEQR;RCNMISGEVEELR;RDLEESTLQHEAQISSLR;SECDDLQAQLQHYSK;SSVSISR;SYSAELFR;TALEQAER;VQQELEDAEER;VRELEGELDSEQR;YQQQVSEVQR | 14 | MSDLKIQVSTSVTSSKTEKPAVVKEEKAPEERIKTPDIISSPPAPEEAKVTTVKEETRRASASTLTPKMSDTSRITKTTRTSSIRTADYESTVGQLTKDYRGTSPAVLEGIASQPVLYSKAFDKIGQQKLSARSRKILRDTTDLALVAPGLKNLLEARIEELEEELEAERAARTKVDKQRAELARELEDLGERLDEAGGATSAQVELNKKREQELLKLRRDLEESTLQHEAQISSLRKKQQDATNELADQVDQLQKAKAKVEKERQQFRSECDDLQAQLQHYSKNKGVSEKMAKQLENQIAELQQKCDEANRNVNDLNSQKAKMQAENSNVVAQLEDTEHQIGSLSKERNSLQSIIDELRQNVEEETRARMKLQSDIRNLNADLDAAKEQIEEEQEGKADLQRQLSKANNEAQQWRSKYENEGANKAEELEEAKRKLQAKLQEAEQNAEAANAKVSSLEKAKNRLQGELEDLGIDVERANANANALEKKQRAFDKTIQEWQAKVSDLQSELENAQKEARSYSAELFRCKAQYEESQDSVDALRRENKNLADEIHELTEQLSEGGRNVHEVEKARRRLEMEKEELQAALEEAESALEQEEAKVMRGQLEISNVRSEIERRLAEKEEEFENTRRNHQRALDSMQASLEAEAKGKAEAMRIKKKLEQDINELEIALDASNRAKAELEKNIKRYQQQVSEVQRQVEEEQRQKEEVRESYNLAERRCNMISGEVEELRTALEQAERARKGAENELFEANDRVNELSAEVQSISSQKRKLDGDIQAMQSDLDEMNNEVRNADDRARRAQEDSARLADEIRNEQEHSQQIEKFRKSLEGQVKDLQVRLEEAESQALKGGKKMIAKLEQRVRELEGELDSEQRRHAETQKNMRKADRRLKEIAFQADEDRKNQESLNSMIDTLNAKLKTYKRQVEEAEEIAAINLAKYRKVQQELEDAEERADSAEGSLQKLRAKNRSSVSISRSSVTHTPSRTLLSTERSDI | catchin protein [Mytilus galloprovincialis] | CAB64664.1 /0.0 | Myosin_tail_1(PF01576) |
| **Unigene6649** | 57.1628 | ATDLLENNFFR;EHNDIVDGLR;FFYENNFVPTGFTTAQLR;FIPPTYGDPSNIGTTPR;IISNAVHR;INSVIGVPLPSPR;IPSMPVNAFVSDR;LFQTAPGNGFDLSALNIQR;NAFGAAAYR;NPALFGADGIGR;SDFGPEPGCPPGPR;SGGALPDAGEFSR;SMNPSWDDER;STPVGHSNSYNPTANPATR | 14 | KDMIKNCLSITIVLSLFPVLTYTQGDIGAAIADAVTEVGTRRPGGPALASAATGAGPPADVSGFGGVPGAGLAGVAAPGPADAATDPISIHALFSTRREAPGTVAAFGTAGDITTTATANFAARSGGALPDAGEFSRQIAPYCPVRRALCDPGAPYRTADGSCNNLNNPLWGASITNQARFIPPTYGDPSNIGTTPRINSVIGVPLPSPRIISNAVHRGGVSPPRSTIFNVALTHFGQFIDHDIISTPILRDGNEEIRCCTGAGVPVLRPECFSFFTPPGDFQTTCMHFVRSDFGPEPGCPPGPRNQINQRTSFLDLSVTYGNTRDGQNDLREFNTGRLLEGPGRILPEGPSGSECVGPSPCFKAGDNRPAEIPMLTVMHIVFLREHNDIVDGLRSMNPSWDDERLYQVAKKILTGIYQHIIYTEFLPIIIGQQGMDIFGLRSTPVGHSNSYNPTANPATRNAFGAAAYRFGHSLVGSFVAAYNIDLTPRATDLLENNFFRTDAIRNPALFGADGIGRWMTSQLKSRADRFLTPSVRDRLFQTAPGNGFDLSALNIQRGRDHGIPSYNRWRQFCGLPPALHFGTGPLGLINHDPSAAAALGSVYSHPNDIDLYAGALSERPAPGALVGPIFRCIIGLQFRLYKIGDRFFYENNFVPTGFTTAQLREIKSQTMSALYCRTIRIPSMPVNAFVSDRSGAPRIPCQFLRPLDLRPWRK | byssal peroxidase-like protein 1 [Mytilus coruscus] | ANN45955.1 /0.0 | Signal peptide(1-23)/An_peroxidase( PF03098) |
| **Unigene20161** | 41.3441 | AAHAYGVPNTSLFQTVELYEAR;ADDLDKEGTTLLTLQAGTNR;ADDLDR;DGVILCNLMK;EAQAEIPLQYGTNK;GMTGFGAIR;GMTGFGAVR;GMTSFGSQR;HISDIR;LNLTPSPSELPFK;NLPMVLATISHVGTEAQR;VQFTYEQLK;YDPAVESEVR | 12 | MADRVKPMGMDRALISKMGAKYDPAVESEVRGWINQLIGEDIGEGPSNLEKGLRDGVILCNLMKKIIDGTPSESLPPACNKLNLTPSPSELPFKQMENIEKFLKAAHAYGVPNTSLFQTVELYEARNLPMVLATISHVGTEAQRHNYNGPTIGSKPTEKHRVQFTYEQLKNSHGTIGLQSGTNKFASQKGMRIGAIRHISDIRADDLDKEGTTLLTLQAGTNRFASQKGMTGFGAVRHIADIRADDADKAGDNIITLQAGTNKFASQRGMTGFGAIRHVSDIKADEFDPNTQSHIGLQAGSNQFASQKGMTGFGAVRHICDIRADDLDREAQAEIPLQYGTNKGASQRGMTSFGSQRHIADIKVSDLAEDMKRQDLDMTPKEYQQYRRELEEAAKQQGQEVEEPQYE | calponin-like protein-1 [Mytilus coruscus] | AKS48134.1 /0.0 | CH(SM000033);Calponin(PF00402) |
| **Unigene9058** | 40.2960 | CIIPNEMK;EEQAEADGTAEAEK;EHLVQTLFAPAK;ENQSILITGESGAGK;EQVVYSVSAMAK;IAGADIETYLLEK;IEDMANLTYLNEASVLHNLR;IHFGPTGK;NLYSTHPHFVR;SSAFQTISAVHR;VNQTLDTK;VTFQQAAER;WLPIYTDSIIQK | 10 | MTSGISPDDPDYQYLCVDRKALMKEQTVTFDGKKNCWVPDEKLGFVAAEIQSSKGDEITVKTVEKMEMRTVKKDDIQQMNPPKFEKIEDMANLTYLNEASVLHNLRSRYGSGFIYTYSGLFCVVINPYRWLPIYTDSIIQKFKGKRRSEMPPHLFSISDNAYQFMLQDRENQSILITGESGAGKTENTKKVIMYFAKVAASLGKKDKEEETAAKDKKGNLEDQIIQANPVLEAFGNAKTVRNNNSSRFGKFIRIHFGPTGKIAGADIETYLLEKSRVTFQQAAERDYHIFYMLLSNAFPKYHEMMLLTPDPGLFSFINQGALTVDGIDDVEEMKIADSSFDILGFTEEEKTSLYKCTAAIMHMGETKFKQRPREEQAEADGTAEAEKAAFLLGVNAGDLLKSLLKPKIKVGTEVVTQGRTKEQVVYSVSAMAKSLYDRMFKWLVMRVNQTLDTKNKRNYFIGVLDIAGFEIFNFNTFEQLCINYTNERLQQFFNHHMFILEQEEYKKEGIQWEFIDFGMDLQACIDLIEKPLGILSILEEECMFPKADDKSFKDKLFANHLGKSPNFGKPGAASKGKGQSDFELHHYAGIVPYSTVGWLDKNKDPINETVVELLSHSKEHLVQTLFAPAKEEPGTTHKKKKSSAFQTISAVHRESLNKLMKNLYSTHPHFVRCIIPNEMKQPGLIDAGLVLNQLQCNGVLEGIR | myosin heavy chain, striated muscle-like isoform X6 [Crassostrea virginica] | XP_022317649.1/0.0 | Myosin_N (PF02736);MYSc(SM000242) |
| **Unigene40545** | 40.4858 | EVGVHLVNVYR;FNDEHIPDSPFR;GDYMLIVK;GEINQPCDFNIYTR;IEGQAGPSDVIQHGMSQVVVETVTK;LANGHLGISFTPR;VNVSPSIGDAK;VVAPSGVEEEAIVQEIDDGQYAVR;VVEEDINNLMATIR;YAGVHIAGSPFK | 10 | ATYYPKDEGKAKVDVKYAGQNVPGSPFPVEVFPGVDASKVLVSGPGVGKNVYASMPATFTIDTRNAGNAPLDVVVQRPDGSFIKPLVQDNGDGTYTVQYVPDDLGTYVLRVKFAGKEVPNSPFKVTSHPTGDASKCVITEGLENKTVQVNKETVICVDASQAGDGKVTCRIRSPQGSDIDIDIVENADGTFSLLFTPQIEGAYTISIKFGGQTVPGGEYDIQTTGYTAVNEDLISADTVDSVKGAAPGSGLFQPVDFCIPVGPIFNFVSAYIVMPSGKKAYPKIEDNKDGTVTIRYQPTETGLHELHVNYNNEEIEGSPFKFHVDAVNSGHVTAYGPGLSHGIVNEPAYFTIVTKDAGAGGLSLSIEGPSKTEIKCNDNGDGTCTVSYIPTAPGEYNITVKFAGQHISGSPFTSKITSPPGEIKRKSQFGRSSEFELKVVEEDINNLMATIRTPSGVEEPCLLKRLANGHLGISFTPREVGVHLVNVYRNGHHIQNSPFQITVGESELGNASKVKVYGPGLEQGNANELNEFTVDTKDAGYGGLSLSIEGPSKADIECQDNEDGACRVTYKPTEPGNYIVNVKFADEHVPGSPFNVKVAGEPSPKLTERITRHREAADVMHIGSQCELSLKIPASFFMKMMTHEQIEAILVQQQEGTSPFDMTASVTNPSGVTELCDIVSLDDNHYSIKFVPKEMGVHTVSVKHKDMHIPGSPFEFTVGPIAGGGSHKVHAAGPGLERGEINQPCDFNIYTREAGAGGLSIAVEGPSKAELDFDDRKDGSCGVTYKVAEPGEYLVSIKFNDEHIPDSPFRVNVSPSIGDAKKLSVSALQSKGLQIGKPAAFVVNFNGAQKGKLKARVVAPSGVEEEAIVQEIDDGQYAVRFIPRENGGHNVHVFFNDCEIPESPFRIMVGKVDCDPGMVHASGDGLRTGQSGQPAKFFVNTVNAGPGALGVTVEGPSKVKLECTEKEEGYEFTYYPTAPGDYLITIRYAGVHIAGSPFKARIEGQAGPSDVIQHGMSQVVVETVTKTSVMSKFQAIPQFKSDASRVTCEGNGLKKAFRGKQATFNVDTSNAGNNMMFVGMMGPKGPCEELCVHHKGGYQYKINYVVKERGDYMLIVKWGEEQIPGSPFCVHVE | filamin-like protein-1 [Mytilus coruscus] | AKS48135.1 /0.0 | IG_FLMN(SM000557) |
| **Unigene51088** | 40.3825 | EGSSDAQEIMQDR;QSLNNSPMFNSR;QTPFSMMFR;QTSLQLSSAR;SPLLSQDLATIR;SSFPQSR;SSFSQSSLPMMQSR;SSLSLLDR;TVVNPTDNFAALMER;YIEIVHGNVHER | 10 | MYQEGIILIFFIFISHSNGQGCPHSATPVPGDPTRYIEIVHGNVHERNCAPGTEYNTFTCDCSVFSGASIQALMDGSAGSHPDLLNMPLETLQKEFLQLTSQRAPQSTRTDSRRTSKRRKSKTAEFQYNPKKKESQESILFGLLADSALLNLLPTNPPSKAVKHVEKPQFKSEFIDYSLLAPTKAPKIKTFSALNLPKVTPKPKLKSPLLSQDLATIRAFLKERREQRAREKALLKTKQSPITSSASTSSIGLSGTKNAFSFDSGLKILGSSTTKQSTASNLGLNDLRTSAAKSTSSLDIGFNDYHKAALASNTGGLNTNNVKSSVGIAVPTPDAGLNEFRSSLSLLDRNYPTPTPHSRDTFANVKTVVNPTDNFAALMERRKLEQALITKSEPSSSISGISNTASKSSNGLGGLDATKKAILKNLVARLNALEEKNKGTATSSQQQNSMMSQTSSNSDQMQGQSTSLSQRQSLNNSPMFNSRQTSLQLSSARSSFPQSRSSFSQSSLPMMQSRQTPFSMMFRGVAAKEGSSDAQEIMQDRITEMGGAAALAMGAPLSMINSEISSQLSSTMLENGPLPFLM | Matrilin-2 [Crassostrea gigas] | EKC40227.1 /1.2 | Signal peptide(1-19)/SCOP(d1dqca_) |
| **CL1011.Contig5** | 38.5526 | DGHPGVVGPMGPR;GAPGPAGPR;GPIGPEGPSGQGLPGPQGPPGR;GPMGPEGPQGPPGLAGPR;GQPGPQGSPGLR;GQVGIPGPR;GSPGLGGTSTR;MGAVGAPGR;TPHTGPPPGYGWSQQR;YPPYGNSYGPSAYQQR | 10 | GPPGKDGRPGMRGLTGKPGNAGIQGPIGPPGPQGPPGKDGRPGMRGLTGKPGNAGIQGPIGPPGPQGPPGKDGKVGLQGAPGVAGKPGTNGVPGMRGLTGRPGKQAIPIKAAAIPGPPGPIGPQGLIGPAGPKGANGRPGMRGLTGRPGAHGNDGKAGPPGKDGLPGKPGPPGAAGRPGMRGLTGRPGAGKDGPQGPPGKDGAPGRPGMRGLTGRPGAGKEGPPGKDGAPGKPGQRGIRGWRGWRGFKGAPGPAGPRGPMGPEGPQGPPGLAGPRGSPGLGGTSTRQTVVRGPPGPPGPQGTRGPIGPEGPSGQGLPGPQGPPGRGQPGPQGSPGLRGATGSPGLQGKPGIPGKPGAPGMQGLPGPIGNPGPPGAGVKGMGKEQVAVQGPPGPPGPPGAAGRPGKDGHPGVVGPMGPRGHPGEPGMMGPHGPPGKDGKPGPKGDSGAEGPPGQVGPMGPAGPQGKAMQATHHDIVQGLPGPPGPPGERGQVGIPGPRGYRGHPGAKGDYGARGPPGRMGAVGAPGRMGQPGPPGRQGLDGAVGPIGKPGPKGECSCGGPPMGAMRPPMGAGMGPRMGPGPYRPHMPYMAPKTPHTGPPPGYGWSQQRYPPYGNSYGPSAYQQRSNSLIGGTGKPSGSWRKKPVDTEAEMK | collagen alpha-1(II) chain isoform X2 [Otolemur garnettii] | XP_003793620.1 /2e-35 | Pfam(PF01391) |
| **Unigene51075** | 38.1946 | ETADTDTAEQVMQSFK;LASDLLEWIR;LENSFNTLQTR;LMEEYER;NINEVENQILTR;QGLEEAER;TTDNTLPGTQR;VEQIAAIAQELNVLHYHDVQSVNTR;VGWEQLLTAIAR;YTHYTMETLR | 10 | MEDYPSDGYMDEEEEWDREGLLDPAWEKQQKKTFTAWCNSHLRKAGTQIEDIEEDFRNGLKLMLLLEVISGEQLPRPDRGKMRFHKIANVNKALDYIASKGVRLVSIGAEEIVDGNCKMTLGMIWTIILRFAIQDITVEELTAKEGLLLWCQRKTAPYKNVNVQNFHLSWKDGLAFCALIHRHRPELIDYYKLSRENPLENLNTAFNVAEQHLDIPRMLDPEDMVNSAKPDERSVMAYVSSYYHAFSGAQQAETAANRICKVLKVNQENERLMEEYERLASDLLEWIRKTTPWLENRTTDNTLPGTQRKLEEFRDYRRKHKPPKLEDKARLENSFNTLQTRLRLSNRPAYLPTEGKMVSDIANAWKGLELAEKGFEEWLLSELQRLERLDHLAQKFRHRCEIHEEWAEGKEDMLQSQDYLKCRLNELKAMKKKHEAFESDLAAHQDRVEQIAAIAQELNVLHYHDVQSVNTRCQLICDQWDRLGTLTAQRRQGLEEAERILEKIDQLYLDFAKRAAPFNNWLDGAKEDLLDMFIVHSIEEIQDLIEAHEQFKGTLGEADKEYNSIMGLANEVQRLAQQYGLTLKENPYTTVSPQEDIANKWGEVKQLVPKRDRTLHDEKIKQENNERLRRQFAQKSNVVGPWIENQLDGVASIGVTARTSLEEQLNKLRQFEKATESYRVHMDELERYNEEVQESMIFENRYTHYTMETLRVGWEQLLTAIARNINEVENQILTRDSKGISEDQMNEFRVSFNHFDKNRTRRLEPKEFKACLVSLGYNIRDDRQGDADFQRIMSIVDPNNSGYVTFEAFLDFMTRETADTDTAEQVMQSFKILAGDKPFITAQILRQELPPDQAEYCIQRMAPYSGRDAVPGALDYMSFSTALYGESDL | Alpha-actinin, sarcomeric [Crassostrea gigas] | EKC43084.1 /0.0 | CH(SM000033);SPEC(SM000150);EFh(SM000054);efhand_Ca_insen (SM001184) |
| **CL1023.Contig1** | 28.5096 | FELPYSTIR;HQAVPIR;LMQGGYQR;LSEISEPSNEVIR;QLSFAR;QSYNIPLLLEAQSPNQGTVIR;STESADAVER;TPVTFPPNR;VLMSEIAPRFR | 8 | KNTKTFVVEDVVLPKFEVNVILPSIQLTTDSHFTATITAQYTFGKPVEGDVLLSIYSGSKRRGITKRFKINGKATIRVLMSEIAPRFRYFTVEAEVTEAVTGDKQGNAQRTHLYETQEKLLFSPTMPYTFKPGLDYNIILRATQENDKALTGYLGQVNVTVFYRVPKQKEEKTGALQCTEGMCPPSDETEEKVLWSKNIQIPESGLMKEVASFPINALSGQVQADYRMASDRKYLSKAQSPSSNYIQVTIVNDQKAKAGTNLPLMIKATEPVQYVNYKIFAKQYMMQQGTFDMQNTSSKQVQIAITTDMAPKVKLLVYYTRPSDGEIVAAAVKFPIEGIFDYEVTLRFNKERALPGDNVTLSVTADPNSLVSVLAVDKSVLLLRTGNDVTVKDVINELNNYDGNLYPNFGAWDYWFSRPISSIDASSVFKDMGVYVLTDSLLYKHSEVVRRSQSFNVPMNAEILAMDAPAMSFTSGGMDDLAKPSRTRKNFPETWLWTNTMTGASGISMINAKAPDTITEWVTSAFAVNPTSGLGVSSDIANLTIFQKFFMRFELPYSTIRGEIVIVQITLFNYLATAQNVQVKLNGGEGFSFVDANGDPLNTGSNGMTKTTLVKNDSVSSVYFPIKPTTVGKVTLSATARSTESADAVERELIVEAEGIKQSYNIPLLLEAQSPNQGTVIRTPVTFPPNRVPDSTFVKVQVIGDFLGLALANVENLLGSTSYGSGEQNMITFVPNVYISSYLKTTNRLTTEIKKKTERLMQGGYQRQLSFARIDGSFSAFGSNDPRGSTWLTAYVIKSFAQAAEFTYIDKKVITKAIKWLLTQQAHSGNFEENGIFIRKELQGGSTSSARSLTAFVLIALYEAKSNDQVEAEIKSNVDSAISKATQFVAEGAPASITNVYELAISFYALSLVKHASKNLLLVELEKKESIDGEEKFWRIPKTEADIIQPWKEWSPQNEEFRALDIETTAYVLQGYNLNDDTKNGTRILRWFGRRRRSRGGFKSAQDTAIALEGLSELAKKLYVPSTSLTINVKADNLAGRTFNIRDENSLVLQNEDITTLVDHIEVTTSGTGISLMDIDVYFNVMSELRVPAFNMTTALPKDSTKGFRLRICFSYLKDDESGMALLEISLPSGMEADLTSLDTSRTWGKFKKAEKAFRQINLYFESIQSRNMCVELDVNRVSLVARHQAVPIRLSEISEPSNEVIRLYKSKALSTATIIEVCGADNCQELRK | protease inhibitor-like protein-1 [Mytilus coruscus] | ALA16013.1 /0.0 | A2M_N_2(SM001359);A2M(SM001360);Thiol-ester_cl(PF10569);A2M_comp (PF07678);A2M_recep(SM001361) |
| **Unigene48535** | 31.6407 | FGNSGASGNGWNNNVGANGGR;GLNSGGVNSGTR;GQQGMLGNNNNNGK;MGGVNSGTR;QINLSNLNAEQLEVIMDQR;SCPINTCQGGAK;TEFFIPR;YDTVNSMMCR | 8 | SLICILLLVKFALIAGQRQLPRTEFFIPRTQQNAKRVQQKFTQLERLKQLNNMSGMKFGNSGASGNGWNNNVGANGGRGLNSGGVNSGTRMGGVNSGTRMGGNRRQINLSNLNAEQLEVIMDQRRMQMEMGMGMGMGMSGGGMGAGNGGYTFINVEALNNAAEMGIMPSMFMGNNGARGQQGMLGNNNNNGKGGMNVGRGNGSRRNGMNLPLCQPLYECAENFIPPQCRTWRYDTVNSMMCRSCPINTCQGGAKEMFNTMYEHHPKRLLFEALTGK | — | — | Signal peptide(1-16)/Gly(16.3%);Asn(12.7%) |
| **CL185.Contig3** | 32.2540 | AESPLPDDVLIER;APPAFQQPPPR;HPPPPHQPNQPPPPR;QPAPQQHR;QPQYQQPQQPPVAR;QQHPQPLPPQQPPR;QQQPQYPQQQPAYR;QQYQSSQQPYQGQR | 8 | PEVNLWNGSEMKLINVWTKVWTVFGLWNIAPTLGQGPMAMGGIGGASPSRGGKMTQKFINTILKLHNDYRRTEGASNMKKLRWSRALQRDAQLWANKCRYTHAYGKWGENLFKAESPLPDDVLIERAVNEWYYEKMSWKFTPDCNEACHYTQVVWAESEEIGCAYKRCTTLMLMEEFVMNGWMLVCYYNTQGNIIGKMPYSVGKACSACKPGYKCDRGLCDKQRTVHKYLPQHNQQKPKPVFRAPPAFQQPPPRHAPPPRHAPPPRHGPPSRHPPPPHQPNQPPPPRQQHPQPLPPQQPPRFRVSAPQPPPRPNSSLRPQYPPQHGQPAPYRPQQLANQPARPPSGSPPTNRPKPEPKPQPKPQEPQMQYISGKVLSGWIPAQTSKATPAPTNATFQQPQVQYQHQVPLKQQPYQHSNQAQPQRQQYQSSQQPYQGQRQQQPQYPQQQPAYRQPAPQQHRQPQYQQPQQPPVARQQPNQPQHQQHARPQSTRHSPYQQPYQQPHPQYNAQPAPAQQPQYPPAQPHNPYPDQPSHQPAPYVDPRPIYVPPAPAAPSSPTTTIPPPPPPTCLDGDKHCKHWGVHCKTNPYVHTNCRLTCNTCDIPTPPVHAKPIQTTQKSKAEPTIIPSRPPSPSPGRDHFHQPKPQHRPQPKPRRPPPQPQYNHYATTTAAYYPTTTGYNNQAPTTQTPYNAPGQGTSAAASSVASVGPHVGFCRDFDNRCKEWAKYCGMDEYVDDMCRLTCMRCKK | DNA N6-methyl adenine demethylase-like isoform X4 [Crassostrea virginica] | XP_022319021.1 /1e-44 | SCP(SM000198);ShKT(SM000254); |
| **CL299.Contig1** | 27.9989 | AVCMLSNTTAIAEAWAR;AVFVDLEPTVVDEVR;EDAANNYAR;EDLAALEK;EIVDLVLDR;LIGQIVSSITASLR;NLDIERPTYTNLNR;TIQFVDWCPTGFK | 8 | CISIHVGQAGVQMGNACWELYCLEHGIQPDGQMPSDKTIGGGDDSFNTFFSETGAGKHVPRAVFVDLEPTVVDEVRTGTYRQLFHPEQLITGKEDAANNYARGHYTIGKEIVDLVLDRIRKLADQCTGLQGFLIFHSFGGGTGAGFTSLLMERLSVDYGKKSKLEFAIYPAPQVSTAVVEPYNSILTTHTTLEHSDCAFMVDNEAIYDICRRNLDIERPTYTNLNRLIGQIVSSITASLRFDGALNVDLTEFQTNLVPYPRIHFPLATYAPVISAEKAYHEQLTVADITSACFEPANQMVKCDPRHGKYMACCLLFRGDVVPKDVNASIATIKTKRTIQFVDWCPTGFKVGINYQPPTVVPGGDLAKVQRAVCMLSNTTAIAEAWARLDHKFDLMYAKRAFVHWYVGEGMEEGEFSEAREDLAALEKDYEEVGVDSVEGEGEEEGEEY | tubulin alpha-1A [Enchytraeus cf. crypticus SL-2017] | AOR07106.1 /0.0 | Tubulin (SM000864);Tubulin_C (SM000865) |
| **Unigene43460** | 29.3655 | FLQAQVGPSGPR;GEDGESGGVGQVGPAGPPGEMGVPGDSGIR;GEPGPNGAAGQQGAR;GPQGDIGPSGER;GPQGEDGR;GTMGEPGPTGPQGER;SVYGGAMTSWFR | 7 | MKFGTVRWKNCIAPLFLFIVLVKSQEEDKANCLYEGTTYYHGDQWKPENCKWCVCDNGVADCKELLDCDGFGQITTGQESNTAIQGHQAIENEAEGSVGSPGRDGSQGLPGPIGDPGVNGKHGIPGPPGPPGVPPMSADQAYNRYFQQTYGQSFKAGGPAMGPRFLQAQVGPSGPRGSPGLPGQPGPQGADGVRGESGDTGPPGNPGLRGAPGAPGPPGLEGDSGRNGETGPRGLSGPKGPTGPAGMPGMPGMKGHRGLQGVQGPSGEQGRPGDKGSSGAPGAPGPNGPEGPRGSQGDRGSDGSAGPAGLPGVDGLAGAAGEPGPVGRTGPPGSPGLPGQKGEAGASGPKGSQGLQGSRGDPGISGPPGAEGMAGSDGLPGSNGEKGASGDPGPAGSPGFQGPRGPSGLNGSPGNAGAKGAPGQPGSPGFKGERGPKGIRGSGGDRGPPGAPGNEGKRGQRGTMGEPGPTGPQGERGSTGMRGYPGPIGDPGAAGEEGGIGPRGRRGEPGPNGVPGRMGPPGARGPRGGNGGPGIDGMAGRPGPPGVTGNDGRPGEMGAPGIPGPAGIQGVQGNPGTRGPPGKDGNPGAQGPRGPQGDIGPSGERGNTGPRGAVGEPGGRGPEGNGGAPGFVGAPGPPGGQGEPGKPGEPGPPGKAGKAGRPGSRGERGIPGVTGEPGAPGLSGVQGPEGGAGRDGERGAPGEPGGLGEPGPEGPAGRQGMRGPRGERGAKGEMGEAGLPGEDGREGRKGPSGPQGPPGEPGPPGEPNEKGSVGDLGLPGERGARGTPGDRGPQGTAGIQGEPGQPGMAGAPGPKGQRGQTGQKGEQGAAGIAGAQGAPGPTGRDGMNGRKGVRGDRGSQGLPGQPGTPGGVGPVGNAGPHGDDGPPGPPGEDGIKGSRGETGHVGRPGESGAPGLPGEPGLKGARGEDGESGGVGQVGPAGPPGEMGVPGDSGIRGERGNAGPPGRPGQPGDPGRAGLNGAPGNAGPPGPPGLAGPSGEVGHPGPPGPDGAPGLQGAQGEKGPDGDIGMPGAMGLMGFSGPPGPPGPAGPAGERGERGEPGPNGAAGQQGARGPPGPQGPQGPSGEKGSLGENGDKGDPGLMGMPGLSGPEGPVGDLGPTGPQGPPGQRGPDGRRGDPGSDGMVGPSGPPGPPGPRGPQGEDGRRGSMGEAGNPGPPGAPGRSVYGGAMTSWFRGSSGNKGWQGDEPVPAEEIDTDVFKALEEVTLQIEKIKNPTGEQDSPGRTCEDLRAHNPDIKDGYYWINPNLGPIYDVIKVRCDFRKKRVFTCVQPEVKTIENMNIAQKNDHTWISEVLGSKFDYDPSLFVKPQIKFLQYLHQKANQEIVYKCKNSVAIDDDKSIQLAGFDNSLLSSKGKRSIRYKIKKDNCKNKNGSWEKTVLEVNTKRTKALPIMDIGVYDIGGADQDFKIELGEVCFFN | fibril-forming collagen alpha chain-like [Crassostrea virginica] | XP_022341069.1 /0.0 | Signal peptide(1-24)/VWC(SM000214);Collagen (PF01391);Internal repeat 2();COLFI(SM000038) |
| **Unigene6274** | 26.0717 | DMEETILQHEAQVSTLR;ELTDQLSEGGR;EQVEEEQEGRSDLQR;IEELEEELEAER;IGLSVIQR;IVYSEFK;LSAIISMMQAHIR;NLTGDLDAAR;NLYSTHPHFVR;SSAFQTISAVHR;SYSAELFR | 7 | LQQFFNHHMFVLEQEEYKKEGIQWEFINFGMDLQACIDLIEKPMGILSILEEECMFPKASDKSFKEKLFTTHMGKSPNFNKPGKASKGKKSDFELTHYAGIVPYGTEGWLEKNKDPINETVVDLLSKSKEHLVQTLFAPPAPVEGGGSKKKKSSAFQTISAVHRESLNKLMKNLYSTHPHFVRCIIPNELKQPGLIDAFLVLNQLQCNGVLEGIRICRKGFPSRIVYSEFKQRYSILAPNAIPQGFVDGKVVTDKVLTALQLDPAEYRLGNTKVFFKAGVVGNLENMRDERLSAIISMMQAHIRAYLIRKSYKKLCDQRIGLSVIQRNIRKWLVLKNWQWWKLYSKVKPLLNIARQEEEMQKKLEQLKKLEEDLAKCEKIKKELEVQNVTLLEQKNDLFLQLQTEQDNVIDLEQRVEQLVKQKADFESQIKELEERLLDEEDAASELENIKKKMEGENDELKKDIEDLESSLAKAEQEKTTKDNQIKTLQDEMAQQDEMIAKLNKDKKGMDEAHKKTLEDLQKEEDKVNHLNKVKQKLEQTLDELEDGLEREKKVRSDVEKAKRKVEQDLKATQETVEDLERVKRDLEEANRKKDAEINSLNSRLEDESSLVAQLQRKIKELNARIEELEEELEAERAARTKVEKQRAEISRELDDLSDRLDEAGGATQAQLDLNKKREQELVKMRRDMEETILQHEAQVSTLRKKQADAANEMADQIDQLQKVRNKLEKEKKDMKREMDDMQATFQHQLKNRGASDKVVKQFESQIADLNAELEKSQRNLSDMVNNKTKFEREAAELSQQLEEAEHNVGSFSKEKSRLAQQLEEARSALEDETRVRQKLQSEIRNLTGDLDAAREQVEEEQEGRSDLQRQLNKANTEAQTWRSKYETEGAARAEELEDSKRKLQAKLAEAEQNADAANAKVSQLEKAKNRLQGELEDLAIETERATANANAMEKKQRGFDKTVAEWKSKVNDLQLELEAAQKEARSYSAELFRVKAQVEESQDSVEALRRENKNLAEEIRELTDQLSEGGRSVHEVEKAKRRLEMEKEELQAALEEAESTLEQEEAKVVRAQLEISTIRNDIDRRLHEKDEEFENTRRNHQRALDSMNASLEAEAKGKAEAMRIKKKLEQDINELEVALDASNRAKAELE | myosin heavy chain [Mytilus galloprovincialis] | CAB64662.1 /0.0 | MYSc(SM000242);IQ(SM000015);Internal repeat;Myosin_tail_1 (PF01576) |
| **Unigene57834** | 25.1704 | FAVIITDGSSR;MLFYLNDFK;QGFSYINGGR;TYLGLEYVHNTIFAPGNGER;VAAVSFSNR;VGMMTFGTNPR | 6 | QSSLVVDCFTMGYFALLVLVAVSVVECRNYHSTHVRCEKPVDLAFVIDISSSIWYKHFRREISFIHDIVNLLDVGDRPTQSRVAAVSFSNRLKPEFGLGQYSTKEGVLNAINNIAYEGGDATRTYLGLEYVHNTIFAPGNGERSNVANVVVVLTDGVTNPGSYDNFTRTEAKQKTQIHAQNIRDIVRAQIYAIGIGNEVDKNEIKGIANKPSEQFTLFVDTFTELDTDAVKKAVLTKVCDSLPQREQECSTSKADIFFVVDESSSLMWDANFRKELKFVGSVIDQFELGKDLVRVGMMTFGTNPRMLFYLNDFKTKTEIESLLKITPWHGGNTYLDKAIESLMTYGLNPGFGSRSDVPQIAVIITDGKSTHPTETEKQIAIMKRMNYVVFAIGVGPNKDPIELHKIASHPSNVFEVDNLDGLVAIRQQLLSQLCPGDQPKPPPVNNCQNSMADLIFVADSSTSIGLTAYNEFKTFAKSVVEKFTVGPKNIQIGLITFSNDAHYEFSLNEYRTKEEVTKAIERVPYSTGNTNTHKALEILIKQGFSYINGGRGTSVPRFAVIITDGSSRQPEMTKQLARKAKDQGIILFSIGVGPYITQTELDGMASSPTSMYSFKVDNYAALTRIEQSLVKRTCEEATRQRQFS | Collagen alpha-5(VI) chain [Mizuhopecten yessoensis] | OWF49639.1 /1e-152 | Signal peptide(1-27)/VWA(SM000327); |
| **Unigene6555** | 21.9945 | DHLEYTATAISIIK;DTPAVGDFCR;ISTTFVFQAPK;KPTVDGPR;NSYVQQCDCEIIIGR;SDFALVGR | 6 | DNKVYIVAVLILWTCCPADAVCTCKYLDLQTKLCKSDFALVGRIVSRKPTVDGPRDHLEYTATAISIIKGRISTTFVFQAPKTSSDCGVEFPVRSIQFLMGKRSGGKFVVTSCEVNGQNRPFTTAQWTYLFSRSKRNSYVQQCDCEIIIGRDTPAVGDFCRLVGDGKNQTCYMRNALCRKVGSQGSRQCSWINGEACG | byssal metalloproteinase inhibitor-like protein 1 [Mytilus coruscus] | ANN45954.1 /4e-09 | Signal peptide(1-20)/NTR(SM000206) |
| **Unigene3051** | 23.0788 | AIQNTQNGMWAAGR;CQMIIGIPALPDLR;FTPLHTLLTR;QMPTWPR;QTGNFFVVDTVPGR;STTLAPTGYPCLHK | 6 | SIIPPDTKYQDIVVDNLSYSGRPGSGSVLIEPVPVSSSFGTLPDGVVIADSGSPAAGALPDGVIIEPVPEGSNLGGIQIPTGGNVPVLIKDDNLLIKPSPEKTVWTSEPILINDSNLIATTPSKEEYGQNLSGSGKGSTSAIKTTTSTSSVTIQSQEPSAVVTVNNGTKSNGDNEGTTWLVTPTLPPTTKAPVINPNSGTPKPLSCPDFDSRYENGKFIVTLDEGKCEMVVSERAIQNTQNGMWAAGRFTPLHTLLTRKYKPEINFRSTTLAPTGYPCLHKTRQTGNFFVVDTVPGRCQMIIGIPALPDLRNLLTQRQQKVPTWNKPRQMPTWPRRRPSKEREDNESSD | mucin-3A-like [Mizuhopecten yessoensis] | XP_021361490.1 /0.002 | Pro(10.6%);Thr(10.3%) |
| **CL4671.Contig1** | 23.0788 | GPGGPVAVPQR;GPVLLNIDTVDAASGQR;IPDGAVVAR;SGPASVAGVSAPR;VFAGTQEDAAVTAEASR;VPFETPNLIVR | 6 | MLPVVLLISLFAAGTLGTGYVPPPKLPKKVVSLPIPKPVLKGKPLRQDTAFDHHQIVPIKKTEVRRVVEKVPLVHEVPIFITKNKPFYQPQVVPKPIVVHEVVGLPIVRDKHVVHPVYIDRPYIVEKKQFVEQPFPVEKPIPIDFIKRITKVYDRKVEVPHIVNIPLVKTVDRPRAVPYTREVVQHYDVHVNVPRPRPVKVLRHKTHVEKVPFETPNLIVRKNPVHHAVNEYIPKVPQGFNGRVFAGTQEDAAVTAEASRFGPGSGGRTISVSRGPGGPVAVPQRGPVLLNIDTVDAASGQRIPDGAVVARGARSGPASVAGVSAPRQGGAPVLLGQDLSGPGVGSLGPAIPGGPGPVGVEAVAVGGPGPVGGPGPLVGGGIGGAAVVVGGGGGGIGGGAGVVAVGGGGGGVDLGAGPVVVDGGRGGGAGPIVVSGGGGGGGVDGGFEIVDVGANGEGIINIGPGGLGQDIEIVAVGPDGKDIGSFVVGAGDIAGGTGKKGKK | valine-rich protein-like isoform X4 [Crassostrea virginica] | XP_022307204.1/3e-09 | Signal peptide(1-17)/Gly(18.3%);Val(16.7%);Pro(11.3%) |
| **CL2840.Contig7** | 21.5286 | ALVDTETFASPR;ALVDTETFASPRSAVTSR;ASSYLDDIYYPEPIVR;ASTVSLVR;EATRALVDTETFASPR;EMEVEAVPVQSTTSLK;ERSLPPPVISLER;FQSVPPGYFSSTK;GFYDTTR;GFYDTTREENEIR;SAAMAVR;SLPPPVISLER;SVHPQLLPSDILEK;TPTPVTTSR;VAVLASPLR;VEVVTPR;WYPTTTR | 6 | MTVRRSRFQSVPPGYFSSTKGHSSLKRWYPTTTRASSYLDDIYYPEPIVRSRGFYDTTREENEIRRDVNHELLYTSNLVDDTYDIANKSRNRDQMLLREATRALVDTETFASPRSAVTSRRVRQTSVVRTPTPVTTSRAVSCPPVSRGSSQVVVVLTSPAFKGGSRVFSAKSAAMAVRERSLPPPVISLERSKSVHPQLLPSDILEKRINARRIINTVAGPYMLPLYHPYQSIYQPYISMYQPLYARRKYLATLKDVPSRGRVAVLASPLRRKKYRKAALVGNRVEVVTPRKRKPRSTYAANKMRELKRDEREMEVEAVPVQSTTSLKASTVSLVRY | RS-rich protein-2 [Mytilus coruscus] | AKS48164.1/4e-146 | Arg(12.8%);Ser(11.3%);Val(10.1%) |
| **CL93.Contig1** | 19.4072 | FTQAGSEVSALLGR;GISELGIYPAVDPLDSNSR;IINVVGDPIDER;ILDPFVVGEEHYDVAR;VALTGLTVAEYFR | 5 | VIAQSTVAMMHAARRACVGLLKATKHSLTSPAVPSTATKALPSYFNTRHYAAEQTQPNTAKGRVVSVIGAVVDVQFDEELPPILNALSVENRTPKLILEVAQHLGENTVRTIAMDGTEGLVRGTSCIDTGYPIRIPVGPATLGRIINVVGDPIDERGPVKTDKFLSIHAEAPDFVEMSVTQEVLETGIKVVDLLAPYAKGGKIGLFGGAGVGKTVLIMELINNVAKAHGGYSVFAGVGERTREGNDLYHEMITSKVISLTDDTSKVSLVYGQMNEPPGARARVALTGLTVAEYFRDQEGQDVLLFIDNIFRFTQAGSEVSALLGRIPSAVGYQPTLATDMGTMQERITTTKKGSITSVQAIYVPADDLTDPAPATTFAHLDATTVLSRGISELGIYPAVDPLDSNSRILDPFVVGEEHYDVARNVQKILQNYKSLQDIIAILGMDELSEEDKLTVARARKIQRFLSQPFQVAEVFTGSEGKYVPLKESIAGFQRILSGELDHLPEVAFYMVGPIEEAVAKAERLAEDQS | ATP synthase subunit beta, mitochondrial [Mizuhopecten yessoensis] | XP_021356377.1/0.0 | ATP-synt_ab_N (PF02874);AAA(SM000382) |
| **Unigene39610** | 19.0895 | DGNAGGMSFSSGTGSGNGFAFGGTFNR;GGNGGGFVTSR;SRDGNAGGMSFSSGTGSGNGFAFGGTFNR;TVLINDMKPGTR;VNNGQGIAFR | 5 | MMKALAVLFFIIQVSHGSFGGPYGSSYYNPFQMDKFMYDFFTTFNNIMSMKAPAPRPKPQTFPGAQLFPPTFPDFSGKNSGFKTVLINDMKPGTRKTFKVNNGQGIAFRSRDGNAGGMSFSSGTGSGNGFAFGGTFNRGGNGGGFVTSRSGPKGTKVSYSKGIPKFAKNLFSSFSFF | shell mytilin-3 [Mytilus coruscus] | AKI87980.1/7e-40 | Signal peptide(1-17)/Gly(15.3%);Phe(14.1%);Ser(10.7%) |
| **Unigene58102** | 20.9753 | EPENPPMENALEPAR;EPLTVDPYGDALTSGRPEVAHPEFISSR;SVFINTDNMR;VNSILEQPQYIPETILTK;VRPSTAEFIEQGPLFEHDGTQR | 5 | MKETVLCIVFLQLALVFGAPKSTQTNKVVPKDRVLQGAVEQLFIPDGNSLFEIDIVQGYNPEVIQHVRRKRQAEEKSLERKSSSKKRRRKKKKRRRSEKKPGAVTTLREITPTEKIMLPPVEREPENPPMENALEPARKSRRTFRPPPPTDPAPTLPALVLSEMENNNDDNSGLLKVKSLKSVFINTDNMRTNKLMADRIKADAIHAKDVVVTSKKKKSGSRSGGRKRRRRVRPSTAEFIEQGPLFEHDGTQRVNSILEQPQYIPETILTKNPYGLGDTIYQPEKPRRRFRVNERHLYREPLTVDPYGDALTSGRPEVAHPEFISSRVRSKVPLLSEFIEGTSGNSKPSTTFVNRRRPFAKQRPIEEFHIFDNPINTERQNPVAKRVDKTSKRFTKNVDPWFL | KS-rich protein [Mytilus coruscus] | AKS48160.1/5e-14 | Signal peptide(1-18)/Arg(10.7%) |
| **CL4276.Contig2** | 19.2462 | EDSYEETIR;KLAITEVDLER;LAITEVDLER;QIQEHEQEIQSLTR;TLQVQNDQASQR | 5 | MDAIKKKMVAMKMEKENALDRAEQLEQKLRETEEAKAKIEDDYNSLVKKNIQTENDYDNCNTQLQDVQAKYEKAEKQIQEHEQEIQSLTRKISLLEEDIMKAEERFTTASGKLEEASKAADESERNRKVLENLNSGNDERIDQLEKQLTEAKWIAEEADKKYEEAARKLAITEVDLERAEARLEAAEAKVIDLEEQLTVVGANIKTLQVQNDQASQREDSYEETIRDLTNRLKDAENRATEAERTVSKLQKEVDRLEDELLTEKEKYKAISDELDATFAELAGY | Tropomyosin [Saccostrea glomerata] | AVD53650.1/1e-139 | Tropomyosin(PF00261) |
| **Unigene34337** | 17.5772 | TDPADVAR;VDEHGEFLWPGFGDNIR;VSPVVLDK;VVGDDIAWLK;WEAPEGVPISALIFGGR | 5 | ETRSKGRRSVLLSTLHLQFFLQVNPDQKMSVFGEEDTQFYEVHNIVVQHIGPVPIAKGDFHMLPKKVQKFIAKWVDICAPRALYICDGSQHEADEITHKLLERGVLTTLPKYENCYLCRTDPADVARVEAKTWISTEDRYETVPHVREGVKGCLGQWIAPKDLEKEMGERYPGCMKGRTMFVIPFSMGPVGGPISKIGIQLTDSNYVLLCMRIMTRVSPVVLDKLGDGDFVRCVHSVGCPRPVQRKVVNHWPCNPEKIMIAHRPKEREILSYGSGYGGNSLLGKKCFALRIASVIARDEGWLAEHMLIMGLTNEKTGEEKFICAAFPSACGKTNLAMLKPTIPGYKVRVVGDDIAWLKFDKDGVLRAINPEAGFFGVAPGTNMKTNPNAMLTFQKNSIFTNVAETADGGFYWEGMEDEYDKNMLITTWLNQKWHIGAPGKAAHPNSRFTCPASQCPIIHPKWEAPEGVPISALIFGGRRPTGVPLVFETFSWEHGVSVGACVKSEATAAAEFKGKVIMHDPMAMRPFMGYNFGNYLQHWLDLNAPPHKMPKIFHVNWFRVDEHGEFLWPGFGDNIRVLDWVLRRCAGEQNIAEETAIGYVPKKSSFNLSGIEDQVKWEELFSLPKHYWLDDMRESRRFLEDQVGSDVPKTIWKEIEDQEKRIEKML | Phosphoenolpyruvate carboxykinase [GTP] [Crassostrea gigas] | EKC27095.1/0.0 | PEPCK(PF00821) |
| **Unigene37646** | 19.9339 | EFIAQGLVWLASPSNNNK;LTWACESPCR;MDGEENEYEGPTSMSNR;RMDGEENEYEGPTSMSNR;VSFWYQCK | 5 | VSFKFLSFFLLLMTVTGQRRRPISKTDDRVSFWYQCKQECIRLTWACESPCRNYAETRMAYKMCALECKLDRLVCYDQCREFIAQGLVWLASPSNNNKRNRRRMDGEENEYEGPTSMSNRKDFMAFLNYILES | P,N-U7 [Pinctada fucata] | AKV63173.1/0.71 | Signal peptide(1-17)/Arg(10.5%) |
| **CL3397.Contig2** | 18.3658 | FADEHVR;GSPFLLNVGGQPSGR;SEMDYEETGNR;TPSGGHGDCFVQEMDDGIYAIR;YGDEDIPGSPFVLHAA | 5 | MPSVEGLKPDIKDCDDGSIIVQYKPSKSGTHEVQMAYEGSATEGSPFSCVVDEIGGGYVTAFGAGLVGGMSGQNQTFTITAKKGTLSDIDINIDGPTKTEYKRSDHGDRCDVTFMPMTPGAYNINIKYKGKTIKGSPFVSKVSGEGRKRSQISLGNASEYALNVMEPDIVDLVGSVRGPKGGFEPCILKKSKDGHLCVSSFSPKVAGDYKVQVYRDEKNIKGSPFNVSVSDKDIANAGKVKVTGATEKAVANESNVLNIDASEAGYGGITMVVEGPHRSEMDYEETGNRTFKCTYSPHEPGIYILNVRFADEHVRGSPFLLNVGGQPSGRIRETVEIDMEQAEPVKKGTKCEFLLKIPGTNPFDMEASVTDPAGTTELCEVMDEDDFHYRINLTPHKDGLHTLSIKHKALHISGSPFQYSVGQLSSGGYHKVQVGGPGVEKGEVGKENHFNVYTREAGVGKLSIGIEGPSPAKIVQERRPNGFLGVSYKVEKPGMYGIHVKFNDEHIPNSPFMVNIAPDSGIARTVTVHALKDRGLAVDKATTFTVSYNGAKGRLNAHLRTPSGGHGDCFVQEMDDGIYAIRYIPKENGVHYIDIKLDDHHIPDSPFAVMVGSAAADPAMVHAYGEGLESGKSGMKNKFVVRTAGAGSGFLALTIDGPSKAALSCKEVDEGYEFSYTPFCPGKYLIAIKYGNINIAGSPYVADITGSGRKPSPVKEQSTMVVETVEKQPGAKSLARFKGDAGRVNVRGPGLKKAVPGRLQSFTVDVKDAGHAMLMVGMVAPSGIAEPELAVKKNTKTEFTVSFKVQELGDHTLSVKYGDEDIPGSPFVLHAA | filamin-A-like isoform X5 [Crassostrea virginica] | XP_022315171.1/0.0 | IG_FLMN(SM000557) |
| **Unigene483** | 13.7472 | AAAAAAASASASAGSGIGVASR;AALVQLVIK;LYAYDYYK;RAALVQLVIK | 4 | GFGGGFGGGAGAGAGAGAGAGAGAGAGAGASAAAAAAAAASARRAALVQLVIKARAAAQARAAAAAAASASASAGSGIGVASRFGGGFGGGAGAGAGAGAGAGAGAGAGGAGGASAAAAAAAAAAAAARNANLRGWQSANANSLAAAIAAASAGGGGGAGAGAGAGAGAGAGGGAGGGAGGGAGGGSGGSGGSGGSGGSGGSGGSGGSGGSGSAVRLYAYDYYKNSDDKKGPGYERS | — | — | SCOP d1gkub1 |
| **Unigene34861** | 15.4737 | EFDDLSIEEQR;IGVFGENESTEGLSFTGR;ISTIHSTTLR;REDMDLEDSR | 4 | MAGKNAESAIEALKEYEPEIAKVVRKNHRGIQRIRASNLVPGDIVEVSVGDKVPADIRISTIHSTTLRIDQSILTGESVSVIKHTDPIPDPRAVNQDKKNVLFSGTNIAAGKCRGIVIGTGLNTEIGKIRDEMMDTETEKTPLQQKLDEFGQQLSKVITIICIAVWAINIGHFNDPAHGGSWVKGAIYYFKIAVALAVAAIPEGLPAVITTCLALGTRRMAKKNAIVRSLPSVETLGCTSVICSDKTGTLTTNQMSVCRMFTFAKIEGNDVKTDQFEITGSTYAPEGDIYKDGKKVLSGEFPGLEELATVCVMCNDSSVDYNDTKNIYEKVGEATETALTVLVEKMNYYNTDKTGLNKREKGTACNHVISQMWKKEFTLEFSRDRKSMSVYCSPNKPTRTAQGCKMFAKGAPEGILDRCTHVRVGANKVPMSPAIKNEIMKHVASYGTGRDTLRCLALATIDNPPRREDMDLEDSRKFIEYETNMTFVGVVGMLDPPRTEVMDSIKRCRDAGIRVIVITGDNKATAEAICRRIGVFGENESTEGLSFTGREFDDLSIEEQRRACMKARLFARVEPTHKSKIVEYLQGEGEVSAMTGDGVNDAPALKKAEIGIAMGSGTAVAKTASEMVLADDNFASIVAAVEEGRAIYNNMKQFIRYLISSNIGEVVCIFLTAALGIPEALIPVQLLWVNLVTDGLPATALGFNPPDLDIMKKQPRSTKDSLISGWLFFRYMAIGIYVGCATVGAAAWWFMVYDHGPKLNYYQLTHHSQCLAQDQRFQGINCNIFDSPEPMTMALSVLVVVEMLNALNSLSENQSLLAMPPWSNPWLLGAIALSMSLHFLILYTEVMSTIFQITPLNFAEWSAVLKISIPVIILDETLKFVARKFTDDYPNFF | sarco/endoplasmic reticulum calcium ATPase isoform A [Pinctada fucata] | ABS19815.1/0.0 | E1-E2_ATPase(PF00122);HAD (PF12710);Cation_ATPase_C(PF00689) |
| **CL2603.Contig1** | 12.9166 | EFEFQSGDLWEVR;GCLDYGLDR;MVNILLK;NVVPGYTGEIR | 4 | MSDKTRANKSGLGYAVEKKMEDNYDREEAAGTPTHVVNWVNGILGSEHDPIPGTDWKSICNHLRDGVALCKMVNILLKKDGKSPITFQKKVMSPFVAMTNIENFNKGCLDYGLDREFEFQSGDLWEVRKGPFLNVINCIHSLGFVANKKNVVPGYTGEIRKYLDNE | transgelin-like protein-3 [Mytilus coruscus] | AKS48154.1/8e-109 | CH(SM000033) |
| **Unigene34815** | 12.8063 | AVDSIHYTR;EMNVEECGVR;YSETLVR;YVYGSTNTQDALR | 4 | GVVSFSDSARQEFYLNTYNNADDINKAIRGIRYVYGSTNTQDALRMVREYQFTQSNGDRPGVPNVVVMVTDGESNIEHTRTLPEAMMLKDTGATLITIAVGFTSDSAELRGLTSEPVQSNLIKVDDYDSLDVLKDKLVTPLCTDANLCSPNPCKNNAECVDGLRSYRCICKEGNYGANCEKECTGEADVVFMLDSSSNIGEQTFDRMKRYSETLVREMNVEECGVRVGFMKYSSRPMVQFNMNRYHDTNTITRAVDSIHYTRGQANMADALKEVRTRMFNSADDRRDVRNVIFLMSDGSADIKKDETMMEAEMTISSGINIIPIGIQLRRREELDNIALVQGVNVEEIKDEKDVMAMSDQVLKPVKQVSDFCSGNPCQHGGSCHSDALGYRCDCVPGFTGDNCSKRCKASGDIVFAVDTSRYVTRKDLRQVKKFLKSLVKRMSFRNRMMRAGIVQFGKTADVKLTFRESVKKRNVINAVSSIRRSDGNPDPAEAFRKARISILEAGDRVDVPNYVILITHSMRQESDVIHEANKLKQKGTKVFGVGIGLSASDKEFMMAAVSNPENTYMYNTESVSGLADISDQIMAYLCNDQDYCAAAPCQNGGYCVNKQDGYYCECKDGYAGQNCEKACDAKADVAFLLDSSGSIGQQNFRLVKDFVHRVVQEMAIGKGHTRVGVASYSTNARMGFHLDDYLTKESVQDAISSIGYEYGNTNTAAGIKMVRRSIFNPARGDRSDAQNYLVIITDGVSNVNAENTIPEAKRAKEDGAHVYTIGVGSFDPTELKAMASEPVDKNSFMIDDFKALSSLTTDLIKATCRDPSACADNPCLNGGVCSIGVASFVCACPNGYSGERCEKACVDRKDIAFVLDSSSSVGKGNFDYMLDFVRALVEEIGSTSNEHKFALITYSTEVHLIFSFGRYRNNGEVGKAIATTRYTAGSTNTAGGLRTACEVFNGGEYGGRRAAEDVVILLTDGQSNVNSHDTIPAAEALKQKGIKVITVGINIQDTAEIKAIASSDNDVFLAESFKSLQDIKQDISDNSCKAKGS | Collagen alpha-3(VI) chain [Crassostrea gigas] | EKC21865.1/0.0 | VWA(SM000327);EGF(SM000181) |
| **CL3748.Contig1** | 14.7293 | APDFVFYAPR;IGFPWSEIR;LAAQYAEAQEEIQR;QQEELEEQR | 4 | MPKAVNVRVTTMDAELEFAIQPSTTGKQLFDQVVKTIGLREIWFFGLQYVDSKGYTTWLKLNKKVLSQDVKRETPLQFKFRAKFFPEDVTEELIQDITQRMFFLQVKDCILSDEIYCPPETSVLLASYACQAKFGDYNKETHPNGFLANERLLPQRVMEQHKMTREQWEERITNWWSEHHGSLREDAMMEYLKIAQDLEMYGVNYFDIKNKKGTELLLGVDALGLNVYEKEDKLSPKIGFPWSEIRNISFNDKKFVIKPIDKKAPDFVFYAPRLRINKRILALCMGNHELYMRRRKPDTIEVQQMKAQAKEDKMSKQQEKQRLESERIAREEAEKKQKEMEEKLRRFEEESERRAKEMAEQERRLRDMTEEMEAYKRQQEELEEQRRAAEELRRQYEESAHLAQEEKDRLAAQYAEAQEEIQRSMAVLEEKESEMNSMQQNLEQAQKEREEKEQALIEAMNTIHVRETEHEENTVEEVNHEYSQVETSEDVHMTFENEYEENTVEEMNHEYSADLQDYEQVESLPRPEEERLTEAEKNQRMKEQLKSLQEELQNTKIDEKATTTDMLHAENVKQGRDKYKTLKQIRQGNTKKRIDEFESM | radixin-like isoform X4 [Crassostrea virginica] | XP_022292008.1/0.0 | B41(SM000295);FERM_C (SM001196);ERM(PF00769) |
| **Unigene8686** | 14.7931 | RLGLDDFGDDNGDDN;RPNDGDSDGNSPPGVK;SDGEYGSNGAGYR;WDSDQDFR | 4 | SGTLYGSCHSKTLDMRKLAVVSILLFLGLPSNIICRNDVKEKDGNRGNRWDDMSDSWGFSEDSSDGGWGSNSVKWDSDSEGWDSDNFGSDRRSPRGRGGNGNNRLDSDSGWYTSEYFSDESDNRVGIRGDNGNKLRNIVGLESEGIDWDSDNSDSSNGAVKKAGNGNSGFGSNGIDRNSETSDSSDGVSKNAGNGNESNGRGRKGNINIGGHSGGRFDSDDSDWGSDRWDSDQDFRRGRSNGNKENNGGGDSNGGGRRWGDSDRRDSDSWDSDDNNIWDGSDGWERERSDGEYGSNGAGYRGSDDSDWFESDNRFDSAESFERLIKRLRRPNDGDSDGNSPPGVKFLGNNGVKRADFWDDSDDLWNDYAIGRRRHRPRRRRISAKRLGLDDFGDDNGDDN | — | — | Internal repeat |
| **CL4656.Contig1** | 15.7389 | DNMEALSAR;IDSLEDENAR;LNDQIGGYEGELANLR;LSSELESILR | 4 | MSQEKVEVRTRNTKTAQTMGPRSTIITRHSTSGTLPMAGTRSSTFRASYGGGGFGGAASFATGTVSGMSQKNVANVLDTRAKEKNEMNVLNERFASYIEKVRFVEAQNKALLAEIERLKKQKNFDVSEIKELYEQEIADSRKIIDDLSDEKAKFDSTLVSLQDQLEDEKRDRINAEKTVDDLRNKIDRLNDQIGGYEGELANLRLRIDSLEDENARLKKDKKTLQDDIARIRADLDEETCKRIQAEMKLQTAEEDFKFNQNIYEAEIAELRAMLDKDKSIEMKDIWKGEIQKAISELQAQYAAELDRMQGDMQKNFEMQLNEMKAGVNRDNMEALSAREESKKVKGKLSELQPLINQLQAENAMLKSRLDALQIQYDDECREHEEDRLKLESQIQKLSSELESILRELQILQDAKLSLELEISCYRKLLESEEQSLKRVVEESSGARSSGAQLLSDMIVTKGGSEASQKSSMSTSSRKVNLVKNSRGDLRFERCDPSGTKVTIKNNGTKSISMRGWRLIKNINGVDKCKFNFADDYTIGAQREVTICGKVMADELEYGELLGDFNTWGTHGKFILFDDKNVEKASMDVQVL | retrograde protein of 51 kDa-like isoform X4 [Crassostrea virginica] | XP_022320110.1/0.0 | Filament(SM001391); SCOP(d1ifra_) |
| **Unigene31163** | 14.8605 | FDFDLSR;TQLILSSVLTEGR;VQSGAWVAYTDR;YDVPYIAVLK | 4 | LWFPVLLYINPCISSIIYPRSTIDMDPKITLYKDKDFKGRTVTFTESIPNFVNVGFNDTLSSVDVQGGVWIFFSDINYQGNIYVVKEGDRVNVSGYNDKASSVKLINYDFSDPPSCTLYADSNYWGRSLNLTADALNLKWYDFNDRVSSIRVQSGAWVAYTDRDFGGKQSFYLQGGHSLSASEGSFPNDSISSMRAIQTKPSGPITILKFDFDLSRAIITNKPSVVFQWTQINDSSVEQNLSITTEKSITKDNTYEFHWKQGTSITASMEMSVGIPVLSEGKLTMSATASYEVGSKTGTRTSKTEKWVVKFPSRIPARTQLILSSVLTEGRYDVPYIAVLKQGNKTWTEQGVFRGVNYYGFVTDFKEEPL | gamma-crystallin N-like [Acanthaster planci] | XP_022092298.1/3e-74 | XTALbg(SM000247); ETX_MTX2(PF03318) |
| **Unigene57065** | 14.8267 | ALFQFISGK;NFEAADR;TAQAIYNSLNK;YHSQLLTDINEYAADHPK | 4 | KILAMSQRYKYLYGVPKSGVNSASKNFIENYPSTFENSGTKLYQAIIHKQGVFQENLATSVMRYTKNDRRKTQEFLMGVMKLYLQAVKMELSYYALQQQDTLMKYMKGVLENHVKEVQRNFEAADRTCRDRYHSQLLTDINEYAADHPKSSQSNKDFVKALFQFISGKYYWRHWFVAAYNPISGSDNHWVQVCSGHIRFRVHGRNFIVASKDRYSGRMDLARAERDMKSVATTERKGNWFTGYYTARRTAQAIYNSLNKSGACSVGVIRCGNDLWYYYHSYRFKYVNRCPNFNLHMWG | H5p [Mytilus edulis] | AFR31803.1/ 1e-79 | — |
| **Unigene49503** | 11.5256 | FLFAEVNGDR;NTVIPVTNVR;TSLYEIPNLILR;YTVYIDTIHR | 4 | MMHKDKYEVTPEPRTTISDRLSSFYGNIRSHLLGGVLTGRRSVYEVSDADVAEGGDDLQAFKQETEGKEEVQLKPKSVELQRTLNRQAHRTSLYEIPNLILRRGQSFDIVITFDRSFSERDDDIALRFVTGRQPMQSKNTVIPVTNVRDLQQGQWGYQITSVEDRKVCLKICTGSNCVIGRYTVYIDTIHRNSEKKEEKFRYTHPDDVFIIFNPWCPADIVYIEDENEREEYVLNETGRIWMGTVGKFSVRPWNFAQFDDVCLMASLAMIEKSELADAARGDPLLVVRAISGVINNNEHDGGMLVGNYSGKYDDGVAPYAWNGSAAILEEYLKKRKGVKFGQCWTFAAVATTALRALGIPTRCITNFISAHDSDFSHQIDNFWSTEQKPKKLLNDTIWDFHVWNESWFRRPDLPDGYDGWQAFDPTPQECNEGVFTCGPCSVKAVREGKLYLGFDARFLFAEVNGDRIHWTIDPDGNMEPVSVERGIVGTFISTKAVNTISREDVTNCYKHPEGTSDYEAVVKLAKKLCTRSDVKIVSSPTNDIEFSYSGINQKSGDIDVEVSMKNNGKESRVVDIYIAAIATKYTAVPSNDLKDSLAANVLEPGAENSTKLSLKATDYINTIDPDSHVNIFVIANVKDTNQKFIKRDILRCERPNLELKTDGNATVGKPFDVIVKLVNTLSVPLSGGYVNVEGPGMQKVNTFKIKKPIAPGEEMRETIQIKPRRAGRREIIANFYCKQLCDVTGVTEVEITADNSKS | protein-glutamine gamma-glutamyltransferase K-like isoform X1 [Mizuhopecten yessoensis] | XP_021343535.1/0.0 | Transglut_N(PF00868);TGc(SM000460);Transglut_C(PF00927) |
| **CL563.Contig3** | 16.7803 | AGFAGDDAPR;AVFPSIVGRPR;DLTDYLMK;DLYANTVLSGGTTMFPGIADR;DSYVGDEAQSK;EITALAPSTMK;GYSFTTTAER;HQGVMVGMGQK;IWHHTFYNELR;KDLYANTVLSGGTTMFPGIADR;QEYDESGPSIVHR;SYELPDGQVITIGNER;TTGIVLDSGDGVTHTVPIYEGYALPHAILR;VAPEEHPVLLTEAPLNPK | 4 | MCDDEVAALVVDNGSGMCKAGFAGDDAPRAVFPSIVGRPRHQGVMVGMGQKDSYVGDEAQSKRGILTLKYPIEHGIVTNWDDMEKIWHHTFYNELRVAPEEHPVLLTEAPLNPKANREKMTQIMFETFNAPAMYVAIQAVLSLYASGRTTGIVLDSGDGVTHTVPIYEGYALPHAILRLDLAGRDLTDYLMKILTERGYSFTTTAEREIVRDIKEKLCYVALDFEQEMATAASSSSLEKSYELPDGQVITIGNERFRCPESLFQPSFLGMESAGIHETTYNSIMKCDVDIRKDLYANTVLSGGTTMFPGIADRMQKEITALAPSTMKIKIIAPPERKYSVWIGGSILASLSTFQQMWISKQEYDESGPSIVHRKCF | actin, adductor muscle [Crassostrea virginica] | XP_022325998.1/0.0 | ACTIN(SM000268); |
| **CL2714.Contig1** | 14.6887 | IQELNIDGLK;LAMEEAAAR;QQYDMIELAER;VDESFDR | 4 | MSDTEETTQPENEAKLAMEEAAARKAEKIAMEIAEFEEQRREEKAKEEEELAMLREKREQRKIERAEEEKRLAQLRIEEEARRKQEEKERQQKKAEDEQRRKEERERKRKEQEERLKLVKKPNFVITKRADGGDDERRKKAEQKAEEMQKSKEQLEQEKRAILAQRIQELNIDGLKSDGLIQKAKDLHEKLHNLMGEQYDLEQKFKRQQYDMIELAERARQMNKGKNRSTMGVKVDESFDRLADKFINAPPKIQLCSKYERHTDNRSYNDRMNLFEEFSKPKPPPEIIRKGAQTSGAEDGEEEEEE | troponin T, skeletal muscle-like isoform X1 [Mizuhopecten yessoensis] | PVD33201.1/3e-90 | Troponin(PF00992) |
| **Unigene41989** | 12.9692 | GNLANCIR;QFNGMVDVYR;TEGVLPFWR;YFPTQALNFAFK | 4 | MSKQLSFVENFALSGAAAVISKTAAAPIERIKLLVQNQDEMLKTGRLSEPYKGVIDCTMRTYKTEGVLPFWRGNLANCIRYFPTQALNFAFKDKVKAMFKSSKSDSYGLKFGKNIASGGAAGAMSLCFVYSLDYCRTRLANDAKSGKKGGERQFNGMVDVYRKTIASDGIAGLYRGFVISCVGIVVYRGFYFGLFDTLRPILLGESANVLLSFALGYVVTISAGLLSYPIDTIRRRMMMTSGEAVKYKGSIDCTLQIVKNEGFMSLMKGAGANILRGVAGAGVLAGFDKFQEYYIKWRVGANK | ADP,ATP carrier protein 3, mitochondrial-like [Crassostrea virginica] | XP_022315856.1/0.0 | Mito_carr(PF00153) |
| **Unigene19938** | 16.7803 | EAYPGDVFYLHSR;ITDFEEAFLQHIR;QMSLLLR;VLSIGDGIAR | 4 | LQSAILVGHRREQELVKMLSARFAATLVRQLPRAAPKVCRHALGAGYVASRNISTSTPLCAGAEVSSILEERILGQTSQTNLEETGRVLSIGDGIARVYGLKNIQAEEMVEFSSGLKGMALNLERDNVGVVVFGNDKLIKEGDIVKRTGAIVDVPVGKEMLGRVVDALGIPIDGKGPLGTSTRARVGVKAPGIIPRISVKEPMQTGIKAVDSLVPIGRGQRELIIGDRQTGKTAIAIDTIINQKRFNDGTDEKAKLYCIYVAIGQKRSTVAQIVKRLTDADAMKYTVIVSATASDAAPLQYLAPYSGCAMGEYFRDNGMHAVIIYDDLSKQAVAYRQMSLLLRRPPGREAYPGDVFYLHSRLLERAAKMNDDNGGGSLTALPVIETQAGDVSAYIPTNVISITDGQIFLETELFFKGIRPAINVGLSVSRVGSAAQTKAMKQVAGSMKLELAQYREVAAFAQFGSDLDQATQNLLNRGVRLTELLKQGQYIPMPIEEQVAIIYAGVRGHLDKLDPTKITDFEEAFLQHIRGSQKDLLATIAKDGMITEDSDAKLKQVVKNFLAGFEG | mitochondrial H+ ATPase a subunit [Pinctada fucata] | ABJ51956.1 /0.0 | HAS-barrel(PF09378);ATP-synt_ab (PF00006);ATP-synt_ab_C(PF00306) |
| **CL3951.Contig2** | 15.7389 | FIGSLTQSFNNGR;GNDLEAAR;GTFTDGTR;SGPFLVFR | 4 | MKDIVYIAVLFFTLFHVANSLCSHPCSRVNRGTFTDGTRNFVFGCTNTSVLQVYEGNRFIEDRECYARSGPFLVFRVGSRYQCFKDTVVDPNTNVVMIYFAPTQTFSTNPSICDVCAGEYTFALFVPRGNDLEAARRLPRPPLGCNRPPNCPILPDPYYIPCTGCEPKEDDGLCCSSCQDINNVYNRYGRGNQDRRNRDRRGRSNRFIGSLTQSFNNGRYSRPTKRSAKTC | — | — | Signal peptide(1-20)/Arg(10.8%); |
| **Unigene43** | 12.5852 | CPILAITR;EAESAVFHTQLFEELR;VIIAIDTR | 3 | MSGTKLGGSGSSAKFDLDAMEQEGAYYCQSQQLQAAYASSHLEHMCCLDIDSEPHEVRMTGIICTIGPACVAVPTLQKMIIQGMNIARLNFSHGTYEYHGNTIKNIREAVSKFSVPKPVAIALDTKGPEIRTGLLKGGASAEIALKTGDKIKLTTDEKYYEECTADILYVDYKNITKVMNVGGKIFIDDGLISVIVKEKGDDWLMCEIENGGDLGSKKGCNLPGTPVDLPAVSEKDKKDLLFGVEQGVDMVFASFIRSGSHIQEIRKILGEKGKNIKIIAKIENHEGVKKFNEILNEVDGIMVARGDLGIEIPPEKVFLAQKMMIGRCNRYGKPIICATQMLESMVKKPRPTRAETSDVANAVLDGADCVMLSGETAKGDYPLEAVKIMHKICREAESAVFHTQLFEELRKHTPTPTDPTHTVAIAAVEASFKCMAAAIIVITTSGRSAHLISNYRPRCPILAITRKDQTARQGHLWRGVFPIHYIDSHMSEWTVDVDRRIYMGINVGINRGFIKPGDPVVLVTGWKPGSGSTNTMRVIIAIDTRKKDMLEPITGVSSVPSFNKIGPGEQSAESLKNLSISSESSKSIEGDVKFF | pyruvate kinase PKM-like isoform X7 [Crassostrea virginica] | XP_022311730.1/0.0 | PK(PF00224);PK_C (PF02887) |
| **CL2227.Contig1** | 12.5852 | EMLGGNAAAEILR;FPDPYGYDDFPAFGR;RFPDPYGYDDFPAFGR | 3 | GNGPAEPTPTGTGNGPSDPTPANGPWDPTPADDSNGPSDPTPANGPSDPTPVADSNGPSDPILAADISGPTDATTADSNGSSEDGNGDDEGPPKEDPTPKADSTEPSDPTPAAGANGPSDPTPAAGAGGPSDPTPNGDNGPTDPTPADDEDPSGEDPTPKADSTEPSDPTPAADAVGPTDPTPKGDSNGPTDPTPADLDQMELMKLQSQTQLQMMQIMLEMVKQTKMSAESDTSETKKSKAPKSRKVPDATEPTKTSDATGPTKSTETTAQTESTETSAPTEASETQATEEPSTEVSGPISEPSPTEPSAEPTPTEAESADKSTSASTAASTDKTETSTDPSSSDGPDPTEKTTTTESESSDPTPTTDEISKLAGGIKTEMMKMLMQVMGEVNKIMKIEDAMPTASSSATQDEKPNGDLTITPWTTNPTSESDAESPSKAPGEEEVTEKPTTTPEPAESTTSLEEDSTTAEEPKSSPDTEEPITTPTPTEPSTTAGTGAATDDKLKPTMPSHGIVLKMVALQSETQQMLMKMMEKILSAEDTKSPEPSPSADVSEGATEASEAPTEPTAPGTTSSSDVETTSASEETEPASGPEEPELTTTSKETEPTTSEKPETTTAFTEQETTTASEQEPKPTTTAAPLADAKRDDNSIMMMLQQQTQLMLLQMMKDLRKPDEPVEPESAPEAQTEASTEAPTETPTEAPTEAPIEAPTKAPTEAQTEAPTEAQTEAPTEAQTEAPTEAPTEAPTEAPTEAPTPAPTSPIVEAQADKEESVPPPAETSPSADDLRNIVADTLKEMLGGNAAAEILRVPTMSPGQMERQQQFLRNARQRFVPPTRRFPDPYGYDDFPAFGRGKPYGQKQKLIYFDI | type I secretion C-terminal target domain-containing protein [Comamonas terrigena] | WP_098066268.1/6e-19 | Internal repeat 1 |
| **CL1231.Contig1** | 10.7490 | MFGTDPMLQISK;TFVVEEVVLPK;VLMSEIAPK | 3 | KRPASTYISTVPQTIRPGDVAEITIASTKTDAWTSDADVTLYDSMNKSISNIKANGIQGTTQPAIVLLPIPNDVKSDETYKIRVQSTNGLTFDETSESIFVASKSASIFIQTDKPQYKPGDLVQFRMFGTDPMLQISKDSLTIYIEDPKGNRLQQYVNANPEMGVFAGSFQLSTLTSMGTWKLIVEQGREKNTKTFVVEEVVLPKFEVNVILPSYQLQTDSHFTATITARYTFGKPVEGDVLLWIYSWRKSEGITKRFKINGKATIRVLMSEIAPKFRYFTVEAEVTEAVTGDKQTDSQKTQIYDTHEKLAFSPTMPSTFKPGLDYNIILRATQKDNKPLTGYLGQVNVTVLYRVPK | protease inhibitor-like protein-1 [Mytilus coruscus] | ALA16013.1 /3e-109 | A2M_N ( PF01835) |
| **CL4310.Contig5** | 10.5980 | AILVDLEPGTMDSVR;ALTVPELTQQMFDAK;FPGQLNADLR;INVYYNEATGGK;ISEQFTAMFR;LAVNMVPFPR;LHFFMPGFAPLTSR;YLTVAAMFR | 3 | MREIVHIQAGQCGNQIGAKFWEVISDEHGIDPTGTYHGDSDLQLERINVYYNEATGGKYVPRAILVDLEPGTMDSVRSGPFGQIFRPDNFVFGQSGAGNNWAKGHYTEGAELVDSVLDVVRKEAESCDCLQGFQLTHSLGGGTGSGMGTLLISKIREEYPDRIMNTFSVVPSPKVSDTVVEPYNATLSVHQLVENTDETYCIDNEALYDICFRTLKLTTPTYGDLNHLVSATMSGVTTCLRFPGQLNADLRKLAVNMVPFPRLHFFMPGFAPLTSRGSQQYRALTVPELTQQMFDAKNMMAACDPRHGRYLTVAAMFRGRMSMKEVDEQMLNVQNKNSSYFVEWIPNNVKTAVCDIPPRGLKMSATFVGNSTAIQELFKRISEQFTAMFRRKAFLHWYTGEGMDEMEFTEAESNMNDLVSEYQQYQDATAEEEGEFEEEGEEEDA | tubulin beta-4B chain isoform X1 [Heterocephalus glaber] | XP_004848801.1 /0.0 | Tubulin (SM000864);Tubulin_C(SM000865) |
| **Unigene20345** | 9.3624 | DSFYYSNIMYGQAAR;FDSSLTR;MGIPMHNFIR | 3 | ADTRQIIKTIRDSFACRKNNPGLSVAIVKDGQIVYANGFGVQSLESKKPVNKDTLFGIASLTKAFTSTLLAKLTDKNVNYSLNTQVAEFYGNSPVFDGYFRSKFASIKDLLSHRMGIPMHNFIRFDSSLTRRNLIRRLKVLRPRGRFRDSFYYSNIMYGQAARIAEKIGRKKFEDLITDELFVPIGMTKSGFFTTANE | Protein flp [Crassostrea gigas] | EKC28749.1 /7e-54 | Beta-lactamase(PF00144) |
| **Unigene3720** | 11.2787 | DNIQGITKPAIR;ISGLIYEETR;VFLENVIR | 3 | YNQYNMSGRGKGGKGLGKGGAKRHRKVLRDNIQGITKPAIRRLARRGGVKRISGLIYEETRGVLKVFLENVIRDAVTYTEHAKRKTVTAMDVVYALKRQGRTLYGFGG | Histone H2B type 1-M [Tupaia chinensis] | ELV13502.1 /1e-66 | H4(SM000417) |
| **CL1023.Contig4** | 8.6714 | DSINGFR;LYVPSTSLTINVR;TCFSYLK | 3 | GRRRRSSGGFKSSQDTAVALEGLSELATKLYVPSTSLTINVRADNWAGRTFTIRDENALVLQNEDVTTLIDHIEVTASGTGICLMGVDVYFNVMKELRVPAFNMTTALPKDSINGFRLRTCFSYLKDDESGMALLEISIPSGMEADLTSLDTSSTWGKF | protease inhibitor-like protein-1 [Mytilus coruscus] | ALA16013.1 /4e-46 | A2M_comp(PF07678);SCOP d1ayoa_ |
| **Unigene42847** | 10.7490 | FDYQISEGR;FTENIETNLAR;YGVDSGR | 3 | MLVISVCVLLCVFSPAFPYDLKFDYQISEGRARFTENIETNLARNTVKYYTPAHNDVLESYKMLDFNKKMQITCLPALKQCRLRDIDVEELTGDAGTVAESFVHSWNKGENSIDSANSRTINEVYYIDNEEVKNTIGLGEDLREFYEKYGKNDKGEGFPLYKEKKLPENAVLLNITRSGVKRTKRAFNPLNNDCNGQAPVTRYGVDSGRSCNYLKICKQAAVVNGQRVFADCGNVHITSPLVYVCVCCPGVTEINLNSNKCACTKMNGP | integral membrane protein 2B-like [Stylophora pistillata] | XP_022807324.1 /0.014 | Signal peptide(1-18)/~~ |
| **Unigene30013** | 12.5852 | GDIASGIGGGAIGGR;GTTGFAGAVSGR;TGAVSNFPVFGPGINR | 3 | GRTGAVSNFPVFGPGINRGFGSSFDGNFGAGFGIGPIGGGFPSFGGPASLASLNAALSGSINAATDGFPGFVGGPLGTLSGSLSGGLNAATGGFPGSIGGPGPLGTLTGALSGGLSAATTGLNAGLNAGLSAALRGDIASGIGGGAIGGRGTTGFAGAVSGRFAGTVGAGAGGAIGKGKVY | glycine-rich cell wall structural protein-like [Crassostrea virginica] | XP_022339053.1 /4.9 | Gly(30.9%);Ala(13.8%) |
| **CL678.Contig2** | 10.4936 | AAAAAGAAAAAGAGAGAGGSSGLSAALR;GILGWLLR;VVIQLLTR | 3 | GGLGGGLGGGLGGGLGGGLGGGLGGGADAELELFEDLLGTYGLDIFEGEEGLAALSLLGGLGAGAGAGAGAGAGLGLGGAGAAAAAAAAAAAAAGAGAGAGAGAGAGAGAGAGAGAGAGAGFGGAGGSAAAAAAAAAAAASARSRAAAAAGAAAAAGAGAGAGGSSGLSAALRSRLLARIAARRAAASAAAAASAAAAGGAGGAGGAGAGAGAGAGAGAGGGAGSGAGAGAGAGAGAGAGAGAGAGAGAGAGSGGARGILGWLLRRRAMARAAAAAAAGAGSGGSGGSGGNGGGSDGDCGDSDSDSGSDSNGDNDTDSSDSEGSDGSDSDSGSDPDGDGDSDSSGSSNSSDDNGDSGDYDSGDDGDGDDGGDFKAVAKVVIQLLTRVLSSGVLTAGASAGASASAGAGAGAGAGLGGGFGAGGGAGAGAGAGAGAGFGSGIGLGFGGGFGGGFGGGAGAGAGAGAGAGAGAGAGAGAGAGAGAGAGAGAGAGA | — | — | scop(d1gkub1) |
| **Unigene12260** | 12.5852 | MTEHGLAR;QNGLGGGNPLICSR;RQNGLGGGNPLICSR | 3 | MDPYLFLCVLAIAFSVHNVYAQMGMGHQNQGNMRRKFPGRQAGMGGGMGGGMGTGNNNQQGMNLGGSPADMGMPGNGMGGQGQGQGHGNGGQGQGNGMNNMGGGGGSGGLMGGMLGGGMPMDAMMGGMQANMMAMGAMNSEIPPHMIMSGTFNPKNFAQYRSCEKTPSNLNTICDPSSPNPCPQGAMISKSTPFAMGMGMAMGGMGGMGMGGMNRRMNRRQNGLGGGNPLICSRMTEHGLARCCAKNMMTARMLDKWFK | — | — | Signal peptide(1-21)/Gly(23.6%);Met(15.4%) |
| **Unigene12178** | 10.7490 | EIIVGALPNPTYYR;ELYGGQMTR;YNVAVTR | 3 | NKPEFNLTSTHRATVFSNYVFMVEAYIPNKQDVLLHLDDRRRKPAREARVVIHVYQRLSGIKEVREIIVGALPNPTYYRIVPGRQTPVPYIYLPQSMVQERVIGPKLRAYVDRVAGRMLRELYGGQMTRCGNRCIVFGFRTNLPTRISGKNTRLSWYWMEQAIEYYILHPIDMAILVEKIDENFIIHRVYLEEGLFDSLPAAVNFYYQNRQNFTRVQFPVFSNNLFSTLNQRGMPSFQTPLRNPTSVYPDGKRYNVVDRHVQYMFWNFEISFGTVNGPQLFDIRYKNERIAYELSLQEVAALYSGMKPFEQLDLLDSYMLLGTLTNYLVPGVDCPMDATFIGSYVIKESSDTPLHNRHAMCLFEHNTELPIRRHYTFSTREGAFYEGAVNTVLVARSILTVSNYDYFVDVWFYPNGNIEVKAIATGYISASFRRPRDYHYGFQLHDNVHGPLHHHLFHFKVDLDIKGRRNRYRTSEIVPDIVENKSYAKDANRKTDKFIYQNKVVHKTHLTERVASLQANTKTPKYLTIINEFSKDKYGNAAGFRILNRRPSELLRPLNSRKESALGWARYNVAVTRHKESERRSSSMYGYLDEAQAVVNFQKFVNDNERIVDQDLVVWLTLGLHHIPHTEDLPVTHTPGMDSSFILSPYNYFSEDPAMSMTNAVRIQKTGVNNARVQRYGVRKGTDCSTSQDFLDVVLRRDLCNIFSCN | byssal amine oxidase-like protein 1 [Mytilus coruscus] | ANN45949.1 /0.0 | Cu_amine_oxidN2(PF02727);Cu_amine_oxid(PF01179) |
| **CL3802.Contig3** | 9.0108 | IANFGALR;TVITAEGQTAK;YADWPAPNPAIGR | 3 | EDKYEMCLILEFLAGGELFDRIAAEDYKMTECEVINYMRQVCDGLKHMHENSIVHLDVKPENVMCTTKNSNEVKMIDFGLATKLNPDEVVKVTTATAEFAAPEIVDREPVGFYTDMWAVGVLAYVLLSGLSPFAGEDDLETLANVQRCDWEFADDAFSNISPEAKDFIRQLLIRQPQRRMTVHECLDHPWLKGDLSSRTTRIPSSRYDNIRKKLKAKYADWPAPNPAIGRIANFGALRKNRPKEFSIFDAWFDRKEAAPRFIRKPRTVITAEGQTAKFDCKIIGASPPIVTWSFDNSVLSQSVKYMQKYRGNEYELKISRIKMSDKGVYTVIAENSFGKKEEHGTLKVEANPDLPKIPSSRDTTPLRRSRRPSMSPAPEVKPIEEAPRISFGLRPRLIQAGTEFKLLTCVQSTPTPKVTWSKDGKDISKDPHYMCSYSGGVATIEVQGARMADTGVYTIHAVNELGEHETSSKVVVEDRAHDFDKADIFKSHKQSRSARRTKSGFNFEDSSSSYTETSTSSSSTKTSRSSRRFESSTEESYSSSSSRRSNRRKEIEEPSYEAPEFTTQLSPLILDEGDRLKLTCTVKGRPDPEVEWFYNGQLMQSDDAIKITAIGGVHTLVINSCILDDDGSYVCKAKNPGGQASTRTTVQVNEKKSLSSSKPDFIEHPAGVSLEDGDQATIAAKISGNPEVLWYRGKELIKDSADFQYKQDGNVFKLIIAEVFPDDTGVYKCIASNTAGSVTSSFYIKVEEPDIAPSGPVFVSHPKSQSLEEGTAFVASCTLDKADSVQWSKDGKDVESSERFKFSQDGNTFTFEIPAALATDSGEYTVTAKNSTGSSQWTFTLSVA | twitchin-like protein-1 [Mytilus coruscus] | AKS48140.1 /0.0 | S_TKc(SM000220);IGc2(SM000408);IG(SM000409) |
| **Unigene25503** | 12.5852 | GSPFMMIDNR;TNPWNENSLYR;YSTQYQQTPNQQHTTMSGFR | 3 | GESDNNEENEESSTQEKQKMQYYGNNDENRLLDDSGIESDDSAQSDGKNDKFDYSDRESFEKEYNSLSSEEDHSEMANNDLNKDYDSDEDSYQHNEIKLNVKTSSSSLNWGYNQPTPTPSSVRTNPWNENSLYRSNVDYDDKDVNVEGDSVNAFKKIVPGFTEKTPSYNYMEIQNNKLQVNTFGSDTNDYSESENEKENEGDDVGDGDENKKWNKFSEEKEDKQQNEHWSAGAVPDIYAGTVIVTSRKRGSRIDNTGKEKENENEIENEDKISSKNSKLLGVKKGKKVSSWGGQEGNSAEKAENEVVEKSPDAENEKPEKGSLDKQKEDENSNNLIGLTTIYQEEGDGKSANEENELKQSVKENSKKSLKEENEMKTLKEENEIKSHKEENEMKSPEEEIKIKTPKEENSFGEMKVDLAGHIGDSNIVKPEEDIGESKSNSVFMNNQQFRNVDDPSEPVKSASNSMGVKPFIGDSPKFMGNMKQAQMFYPTAFPAMNSFTTRGSPFMMIDNRYSTQYQQTPNQQHTTMSGFRKRVQNRISKSMGHNIDDPVMTQPLTLKVKIKSTRKSKKGLSPNLSKLVLKMFMNSKSGAPPCDGNLKSVCRPVKMWSKYQQVADWCTSLCPYGQCPSAVCKCQCTGSPMGSVGQKKCRATNTYKQNSGELDQWCQKTCSKGDCPALLCVCS | — | — | Internal repeat |
| **Unigene13632** | 7.0836 | QYEMVFILPK;SFSPQMANDIESK | 2 | AKWTKNKITDFVSSDMFSSATVMFLVNAVYFKGVWEKEFNPKKTRKTDFFLTESNTVEVDMMTMKHSVLYFNGNDYSAISLPYTGKQYEMVFILPKRIDGLNDLKRSFSPQMANDIESK | Neuroserpin [Orchesella cincta] | ODM93513.1 /5e-22 | Serpin(PF00079) |
| **Unigene49709** | 6.4366 | TNTITAGNFR;YIAQYTSPR | 2 | KHPQKIFEFAMTHQKQLTMKLFPNYFTFLRKIAVDLIPRVSCYWGISLQGIRTNTITAGNFRNAAVCKSSELFLTDSIIKIKVSQGIIPCSIFSRIKGPVFTSKTSVLRYIAQYTSPRATCYWGYDLKDLRSQPNGIITNYENAEISTAMYSTLSFIPEVNVAYGMMCSQYKICDRKWQNFILCSNPSSRKLLQCSSEETGTGTGCLPPKRKTQSC | — | — | Internal repeat |
| **Unigene32384** | 8.3901 | ALLFVPR;EVVQNSAFVER | 2 | MPEPETTMDEGEVETFAFQAEIAQLMSLIINTFYSNKEIFLRELISNSSDALDKIRYESLTDPSKLDSGKDLEIRIIPDKNNNTLTIIDTGIGMTKADLVNNLGTIAKSGTKAFMEALQAGADISMIGQFGVGFYSAYLIADKVIVQTKHNDDEEYIWESSAGGSFTVKPATGEPIGRGTKIVLCIKEDQAEYLEEKRIKEVVKKHSQFIGYPIKLLVEKERDKEVSDDEEEEKKEEEDEEKKDDKPKVEDLEEDDDEDKNKDKKKKKKIKEKYTEDEELNKTKPIWTRNPDDITQEEYGEFYKSLTNDWEDHLAVKHFSVEGQLEFRALLFVPRRAPFDMFENKKKKNNIKLYVRRVFIMDNCEELIPEYLNFVKGVVDSEDLPLNISREMLQQSKILKVIRKNLVKKCLELFDDISEDKDNYKKFYEHFAKNLKLGIHEDSTNRKKIAGYLRYHTSSSGEEMASLKEYVSRMKDNQKYIYYITGESREVVQNSAFVERLRKRGLEVIYMVDPIDEYAVQQLKEYDGKNLVSVTKEGLELPEDEEEKKKFEEEKAKYEGLCKVMKDILDKKVEKVVVSNRLVTSPCCIVTSQYGWSANMERIMKAQALRDTSTMGYMAAKKHLEINPDHSIVKSLKEKADADKNDKAVKDLVLLLFETSLLASGFSLEEPGLHANRIHRMIKLGLGIDEEEAPVEEATTEEMPPLEGDEDDASRMEEVD | HSP90 [Mytilus coruscus] | ALL27016.1 /0.0 | HATPase_c(SM000387);HSP90(PF00183) |
| **CL4067.Contig1** | 6.9252 | TQLLQLIQGR;VVGATIAQLLR | 2 | VEYSDKMWLVVFALLAVNSRTLVSGWLFWYSEKCYDHIGCFSNRPPFNRAGMRVPQSPDHIQIQFYLFTRHNQEIPILIDPYSPDSIMQSTFDGTKPSIFVIHGYSDSGAPSSSSWQRRMTQEFLTKDDLNVIVVDWEKGADSMIYQQSAANTRVVGATIAQLLRTLVSNGPTSYDMFTLVGHSLGSHIAGYAGAYVNGTIARIFGLDPAGLSFENSEPEARLNPTDAKYVEAIHTDQESLTALGFGLEKAIGHADFYPNGGQNQPGCERNSRTQLLQLIQGRIGDLSNTIACSHMRVLDLFTESINSNCVFTGTSCSSLTDYESGQCLECDNGCADMGYNGRVRGQGRYYFDTNTSPPYCYTEK | inactive pancreatic lipase-related protein 1-like [Crassostrea virginica] | XP_022340243.1 /8e-125 | Lipase(PF00151) |
| **Unigene2638** | 7.0836 | ADTLSNGPAELR;NYFTQYIHGYGTVR | 2 | MYRIVFPVLLIIVSCLHLSLSQRLRSCVLRAVEGDKRKYKVYLTASRASGEFKCADGTQFYQDKCLCGWPPKNETDNTRPITRPVDLRPDTSRPVVQPVDPRPITRPIDLRPVNPVQPIFPDRPNPNIRPVIPRPPEPCLTEADPDRNYFTQYIHGYGTVRRRCPIGTLYNEVECGCVDLAPVTSIYCKPEIFLDFDSKPINDNGGTHIPIGNNGHVDSVAKAGRFNGIGRLTIWMYSNIDFGEKLTISFRFYDFPGGPEEQVLVSNCMDNELGAVEIALAPRNKEVVFRADTLSNGPAELRLPYKDKSWKNVTFVYDGTALRGKVDEEEQAVAMKGHLDTRAGGFEIGMCNRRGYVGYIDDLKIHRCVDVDYVIGNGNGPPKPYDARRSNLDIAGP | shell matrix protein-like [Mizuhopecten yessoensis] | XP_021364733.1 /7e-123 | Signal peptide(1-21)/scop d1c4ra_ |
| **Unigene40702** | 8.3901 | LPSYPGYNPR;NPFFTINMYYFNAQTGR | 2 | WKLFCESNTELRMIRFLIILLVPVTAAFLFSSLNFNNVNQNLDLRKLLTYRQLPHRCRLPSYPGYNPRNPFFTINMYYFNAQTGRCETFGYSGRGGNRNRYRSPVECLSRCACHMPVDPGTCHNSTTGITRYYYNKVFKMCASFQFNGCEGNDNNFADFMSCQLACGRSGGGGEIEL | nacre protease inhibitor-like protein 1 [Mytilus galloprovincialis] | AKQ70858.1 /2e-47 | KU(SM000131) |
| **CL1757.Contig1** | 5.7771 | ALTVPELTQQMFDAK;FPGQLNADLR;ISEQFTAMFR;LAVNMVPFPR;LHFFMPGFVPLTSR;YLTVACMFR | 2 | MREIVHIQAGQCGNQIGSKFWEVISDEHGIDPRGLYHGDSDSQLERISVYFSEATGNKYVPRAVLLDLEPGTMDSVRSGPFGQLFRPDNYIFGQSGAGNNWAKGHYTEGAELVDSVMDIIRRETEGCECLQGFQMAHSLGGGTGSGMGTLLNSKIREEYPDRIMTTFSVMPSPKVSDTVVEPYNATLSVHQLVENTDETFCIDNEALYDICFRTLKLTTPTYGDLNHLVSATMSGVTTCLRFPGQLNADLRKLAVNMVPFPRLHFFMPGFVPLTSRSSQQYRALTVPELTQQMFDAKNMMTACDPRHGRYLTVACMFRGKMSMKEVDEQMLNVQNKNSSYFVEWIPNNVKTAVCDVAPRGLKMSATFIGNSTAIQEIFKRISEQFTAMFRRKAFLHWYTGEGMDEMEFTEAESNMNDLVSEYQQYQDATVEDDMDFEEEEGEADEF | tubulin beta chain isoform X1 [Parasteatoda tepidariorum] | XP_015920593.1/0.0 | Tubulin(SM000864);Tubulin_C(SM000865) |
| **Unigene35808** | 7.0836 | FTMLVQQLR;INQELEGSR | 2 | MDPHLCTHIIYSFAKLNGNRLAPFEWNDESTEWMKGMYEKFNSIKQQNPRIKTLLAIGGWNMGSEPFTHMVKTTQSRQEFVKSAVDFLRQRNFDGLDLDWEYPANRGSPPKDKHRFTMLVQQLREAFDRDALTTGRSRLLITAAVAAGKKNIDSGYDVPALGRLLDFISIMTYDLHGSWESNTGHNSPLFARSGETGEQRYLNLDWAANYWNRMGVPKSKLNIGLGLYGRSFTLADRNVNNVGAVASGKGKAGKFTREGGFLSYYEVCEMMKSGGKKYYINEQKVPYLVKDDQWVGYDDVDSLSIKVQYVKQQRFAGIMVWALDLDDFKGSCGQGRYPLLKRINQELEGSRYQPDYSIMNAPSIPDILNQPLAPVAPPPRRRKQKPVRKPPVQTPILPALPPAHHTPLLPPVPRVDQHRTSSKDFTCRKGYDGYFASPDSCSKYYMCTDGTAFKFNCAPGLKFNKEHNFCDWPEKVKCTESTKKSSKKNKNRVQQALLPPNPPIRQPEPPRYEPPPPPQPPPTSANGWNFNANAHAATLPPPVPSSPTNSQQSSAWDWVSMIDNPMPFFMSLFGSNDLFADMCANKANGIYPQRDNCRGFIECSEGVSFKGACGPGLAFNPSQQTCDYTHNVPGCK | chitinase-3 [Hyriopsis cumingii] | AFO53261.1 /0.0 | Glyco_18(SM000636);ChtBD2(SM000494) |
| **Unigene53667** | 6.8220 | CELFYFR;YFFNQQTGR | 2 | DFVTAVPTMKILVLLVVCLTVASVSGFWKWGRRHHRPRRPDCTSPKQKGWGLLKFRRYFFNQQTGRCELFYFRGLKGNRNRYKTLQKCEEACGVATTRTLPITTTAATTTSTLPPITTLNEGGNGGGGGNGGNGGSGGNGGNGGSGGDGGGG | Papilin [Echinococcus granulosus] | XP_024351037.1 /4e-09 | KU(SM000131) |
| **CL955.Contig5** | 6.5539 | ALDSMQASLEAEAK;EEEFENTR;EIAFQADEDR;GQLEISNVR;ITELEAELDNEQR;LAEKEEEFENTR;LQEMIDALNNK;QVEEAEEIAAINLAK;TALEQAER | 2 | QAALEEAESALEQEEAKVMRGQLEISNVRSEIERRLAEKEEEFENTRRNHQRALDSMQASLEAEAKGKAEAMRIKKKLEQDINELEVALDASNRAKAELEKNIKRYQQQVTELQVQVEDEQRQREEARDSYNLVERRCNMLQGETDELRTALEQAERARKSAENDLYESNDRVNELSAELSSMSSQKRKLESDINAMQTDLDDMSNEIKAADDRARKSTADAERLANELRSEQEHSMQIEKHRKSIESTVKDLQIRLDEAESQALKGGKKIIAKLEQRITELEAELDNEQRRHAETQKNMRKADRRLKEIAFQADEDRKNQDRLQEMIDALNNKIKTYKRQVEEAEEIAAINLAKYRKVQQEFEDAEERAESADSALQKLRMKNRSSVSMARTTISSTTTTS | catchin protein [Mytilus galloprovincialis] | CAB64664.1 /0.0 | Myosin_tail_1 (PF01576) |
| **CL1023.Contig5** | 8.3901 | DMDDLVEPSR;DVIDELNSYDR;FELPYSTIR | 2 | NSLVSVLAVDKSVILLRTGNDVTVKDVIDELNSYDRTFFPRFGDWDYWFPRPISGIDASSVFKDMGVHVLTDSLLYKHSEVVRRNQLSSGSMAKGMGGGASFAMDAPEMSRDMDDLVEPSRTRKNFPETWMWTNTMTGASGISIINAKAPDTITEWVTSAFAVNPTSGLGVSSDLANLTIFQKFFMRFELPYSTIRGEIVIVQIT | protease inhibitor-like protein-1 [Mytilus coruscus] | ALA16013.1 /5e-67 | A2M(SM001360) |
| **Unigene3946** | 8.3901 | IGGIGTVPVGR;YYVTIIDAPGHR | 2 | MGKEKIHINIVVIGHVDSGKSTSTGHLIYKCGGIDKRTIEKFEKEAAEMGKGSFKYAWVLDKLKAERERGITIDIALWKFETNKYYVTIIDAPGHRDFIKNMITGTSQADCAVLIVAAGTGEFEAGISTNGQTREHALLAFTLGVKQMIVGVNKMDSTEPPYSEARFSEIQKEVTTYLKKIGYNPKAVAFVPISGWHGDNMIEASEKMSWYKGWAVERKEGNASGKTLFDALDAILPPKRPTDKPLRLPLQDVYKIGGIGTVPVGRVETGIIKPGMVVTFAPSNLTTEVKSVEMHHESMPEALPGDNVGFNVKNVSVKEIKRGNVCGDSKNDPPKGAKSFIAQVIILNHPGEIKNGYAPVLDCHTAHIACKFVEIKEKIDRRSGKSLEEFPKFIKSGDAGIVNMTPSKPMCVETFSTYAPLGRFAVRDMRQTVAVGVIKEVTKAEASGGKVTKAAQKAGKK | elongation factor 1 alpha [Mytilus galloprovincialis] | BAD35019.1/0.0 | GTP_EFTU (PF00009);GTP_EFTU_D2 (PF03144);GTP_EFTU_D3(PF03143) |
| **Unigene39231** | 6.2986 | HYGALQGLNK;YTIVFVR | 2 | MAKYTIVFVRHGESEWNQKNLFCGWHDADLSDTGLAEAKNAGKMLKEKGYTFDIAFTSLLKRAIKTLFFVQDELDLHWIPVVRHWRLNERHYGALQGLNKSETAAKYGEAQVKIWRRSYDTPPPALEKSDERWSAKEAKYGNIDESIVPPCECLKDTVARALPFWHDAIIPAMKAGKRVIVSAHGNSLRAIVKYLDNIPDADISELNIPTGIPLVYELDADMKPVKHYYLADEAQVKAAMEKVANQGKAK | phosphoglycerate mutase [Hymenolepis microstoma] | CDS33978.1/8e-130 | PGAM(SM000855) |
| **Unigene52025** | 8.3901 | FAESTSALFR;TINSFMEPFQR | 2 | DLQSYLDDLPMGTGLTGRNMHNNNFNGQDLQSYLDDLPPGQRMNGGGLRQFPDHVYENKFKFNRMGGHQSQFGTQNHKVVGDTMKATTRTINSFMEPFQRIRSMNNMNSNQFLGLSKATDLGSNINRITNHRESPEPTSFHHQIRPHATQMSKQRFAESTSALFREPKSANSRFPKHSTLNNVISQTVITEPSLSPMGLGAFLTKQNPIQKLLSPSKPSTTSTLQDNSATKTSVDNQSKLPIQKSLSAVSNTFRMGNNNQVKEFSPKTAKFQNKHI | cyclin-dependent kinase 1 isoform X2 [Trichechus manatus latirostris] | XP_023595093.1/1.1 | Ser(10.9%);Asn(10.1%) |
| **Unigene34402** | 6.4366 | DQFAQLVR;VILQTLEGHLR | 2 | AKGLHLFTKAEKDTSMGNIQTVGPNQAMVISGGCCGGTNRKLIVGSWGWAWCLVTDVQVISLEVMTLNPVCESVETSEGVPVTVTGVAQVKIMRDQSFLEKACEQFLGKSVREVERVILQTLEGHLRAILGTLSVEAIYQDRDQFAQLVREVASPDVCKMGIEIMSFTIKDIFDNVEYLASLGRAQTAAVKRDADIGVAEANRDAGIREAECDKTKMDTKFSADAKIADSSRNYQMQKADFDQEVNARKAEAELAYQLQAAKEKQKIRAEEIEIEVVERRKMIDVEEKEILRKEKELIATVKRPAEANAFKMELIAEGQRTQTVEQARAEAEKIRLVGAAEAEAIEAVGKAEAERMRLKASAYKQYGEAAMLSLVLETLPKVAAEVSAPLSKTDEIVLVGDDRTTSEVSRLVSQLPPAVQALTGVDLSKVLSKVPGAST | flotillin-2a-like isoform X2 [Crassostrea virginica] | XP_022341488.1/0.0 | PHB(SM000244);Flot (PF15975) |
| **Unigene17159** | 8.3901 | EGQGYISGAEMR;LTDEQVDEIIR | 2 | MSKLSKGEIEDAREVFDLFDFWDGRDGDVDAAVVGDVCRCLGINPTNAVIKKNGGTDKMGEKGYKFEDFLSIYETVNQQTEQGTYADYMEAFKTFDREGQGYISGAEMRQVLSSLGEKLTDEQVDEIIRLTDLQEDLEGNVKYEDFIKKVMAGPYPD | myosin essential light chain [Crassostrea gigas] | CAD91423.1/3e-88 | EF-hand_7(PF13499) |
| **Unigene7565** | 6.2556 | QLMIQAR;TVLRPCPGGTAFDIR | 2 | KDSSPATTTSFAALAPKPNPTVPVFLQQKKKVAANLKNARRNVLNLRDQSFQKEISKPIPNKTSVQNTNKITRKDRTKQTSKPSRKLPDSALSRKGRPINTGSLSLTKDLKQEVAMLLEILMLEHDRMIKGKKKKKQKKTKKKLKTDTKIPSTTPTTLWKSVKRENKIKLPSKDKLLSPQRIRNSLKPRNGEEQNSWNFRRPASFRPGSEIKRQYLSQKDKQTTDEESKSDNDNCQLKPYDKDSSFYIEKTENRTVLRPCPGGTAFDIRSCACTIMTVTRSISDCEPEILLDFNNNMKDKSGKNIHVGADHIEIHNGSALFLENGDITIWRFTGSHIGRQLMIQARFKATKDPETRQYVISNCNYGTDISYGIEIDRVDEVVIFTLDTEPRDQKEIKIPFELDKWTSVSLIYDGLRFSCEVNGKKKSMPLAGNVETRSSPLRIGGCGVKQNRFSGYIDEVSMSFCIPKKRKGLLSG | sushi-like protein [Mytilus coruscus] | AKS48157.1/5e-27 | Laminin_G_3 (PF13385) |
| **Unigene240** | 6.2986 | AAAEAAAAAVAAEQAIADAEK;VLPNFVTNR | 2 | MRTTVFLACFVVCVAVASAHFWHPVIKIPGQKWCTCRPKCQKNEFTAVDGSCGLKILFQTSYDTCCKVLPNFVTNRYPSGIRSIVVRRLGGNGGAGGNGGNGGAGGNGGNGGAGGNGGNGSNGGNGGNGGEGNGAAEKAAETAAAEAAAAAAEMEAAQAKAKAARAAAEAAAAAVAAEQAIADAEKAAADAAAAKAEQAAAEAEEAASKAQAIADAAEKAAAAAQVADEEAAAAAEEAEKAAGEAKEAKEDADKAAAEAGPEVALVGDDAGKDDGGDEGGRGPTNIKIVLAGRRGGFKPYKPFPKYA | — | — | Signal peptide(1-19)/scop d1gkub1 |
| **CL105.Contig2** | 6.9252 | LLLAMSTAAEFR;SQIAIPAQGMIEFR | 2 | RQNRKGCTMSDRESGDEQQEHQGQGSGEQELATKTLQIQSKRFYLDVKQNRRGRFIKIAEVGAGGKKSRLLLAMSTAAEFRDYLTDFSEHYASLDNLPEDGKLKSETMIKDNRRYYLDLKENQRGRFLRVAQTRPRGGPRSQIAIPAQGMIEFRDALTDLLDEFGTDDHGESDEGQGELPESKYLRVENKVFYFDVGSNRRGVYLRISEVRSNYRTAVTIPERSWGRFRDMLSEFVEGSSQSGTAAEAPKETK | transcriptional activator protein Pur-beta-like isoform X2 [Mizuhopecten yessoensis] | XP_021353033.1/5e-148 | PUR(SM000712) |
| **Unigene1886** | 6.4703 | FVGLMSMIDPPR;TADFTNENPLETR | 2 | FPLYWTSDTMASTKVSKSQDKRADSYRYAVTPGGDDGKKKKSKKQKKKENLDELKQELEMDEHKVPIDELYERLGSNPNTGLSVEEARRILERDGPNALTPPPTTPEWVKFCKQMFTGFSLLLWIGAILCFIAYSIQASQDENPPGDNLYLGIVLTAVVVVTGCFSYYQEAKSSKIMDSFKNMVPQYATVVRGGSIFEVKAEEIALGDIVNIKFGDRVPADVRVITAHGFKVDNSSLTGESEPQTRTADFTNENPLETRNLAFFSTNAVEGTCKGIVVKTGDATVMGRIANLASGLDVGTTPIAKEIAHFIHIITGVAVFLGVSFFIIAFILGYFWLDAVIFLIGIIVANVPEGLLATVTVCLTLTAKRMASKNCLVKNLEAVETLGSTSTICSDKTGTLTQNRMTVAHMWFDGRIYEADTSDDQTNATYGKNDESWMALSRISMLCNRAEFKAEQDNVPVLKRECTGDASESALLKCVELSIGKVTQFRQKNKKICEIPFNSTNKYQVSIHETDNPNDPRYLLVMKGAPERILDRCSTVLFQGKEIPLDDNFREHFNSAYMELGGLGERVLGFCDYFLPSDQYPIGYAFDSDEQNFPLTGLRFVGLMSMIDPPRAAVPDAVGKCRSAGIKVIMVTGDHPITAKAIAKGVGIISEGSKTVEDIAAERGCPIEEVEPSEANAAVVHGSDLRDMTPAQIDEILKNHPEIVFARTSPQQKLIIVEGCQRQGQIVAVTGDGVNDSPALKKADIGVAMGIAGSDVSKQAADMILLDDNFASIVTGVEEGRLIFDNLKKSIAYTLTSNIPEISPFLMFILLDIPLPLGTITILCIDLGTDMVPAISLAYEGPESDIMKREPRDPVKDKLVNERLISMAYGQIGMIQASAGFFVYFVIMVENGFWVSRLLGLREEWDSMAVNDLQDSYGQEWTYGQRKILEYTCHTAFFVSIVVVQWADLLICKTRRLSIVQQGMKNHHMTFGLFFETALAAFLTYCPGLDKGLRMQNLRATWWFPAMPFSLAIFVYDESRKFILRQNPGGFVERETYY | sodium/potassium-transporting ATPase subunit alpha-like [Crassostrea virginica] | XP_022323941.1/0.0 | Cation_ATPase_N(SM000831);E1-E2_ATPase(PF00122);HAD (PF12710);Cation_ATPase_C(PF00689) |
| **Unigene17542** | 6.5539 | SIAIHQR;SLVIMQGGHEVESER | 2 | MKLLMLSLVIFAALALQVRADGQCTPNTSSKNHDDPHDDNHKDDQHGDDHHDDDHHDDDETMHYAQCEMEPNPHMASNLHHHVHGSIELSQKGHGAVYLEVHLVGFNTSEDHADHHHGLHLHMLGDMSAGCDSIGDLYNAHPEKHANPGDLGDLVDDDRGVVNEVHHYDWLDIDGTAPNTEALIGHSMTILQGSHKDPDTPASRIACCVIGHGKARPKTAAALHHELEEDKTEHYAHCDVRSNTHQPKALHHHVHGTIDMKQVGYGDLEVTYHLEGFNVSDDYKDHLHDVQIYTNGDLTSGCDNLGAKYDPHEDYHSDLGDLGDIHDDDYGVVNESHRYSWINIFGDDSVLGRSIAIHQRDHLHTSAKIACCVIGRGQSHPEIVHKAKCVVRPNTESTGLHHHVTGNITFEQTPGGATHMTADLTGFNVSEDLSHHRHGVQLHEWGDMSNGCHSLGRMYHGHDDPHDPKRPGDLGDVIDDSNGDVHATRALDHINVEDLNARSLVIMQGGHEVESERVACCVIGRA | pernin precursor [Perna canaliculus] | AAK20952.1/0.0 | Signal peptide(1-20)/Sod_Cu(PF00080) |
| **Unigene37654** | 6.2986 | NIGHFHPAGSVR;TELLESIIDGSSVR | 2 | LWKRAFVYNVDPQIKSSKTELLESIIDGSSVRIVIKENENNIMSVRADNLGLNTNKSEVAAQSIRNIGHFHPAGSVREFHTVPYWEFTTVVTTGDLIKTRWSTGTYEF | phage head morphogenesis protein [Clostridium amazonitimonense] | WP_032122034.1/3.8 | — |
| **Unigene38699** | 8.3901 | IQNAGTEVVEAK;LFGVTTLDIVR | 2 | MFSRLAKPSCIVHVARRSFSLTSQAQQGPKVTVCGASGGIGQPLSLLLKNSPKVASLSLYDIAHTPGVAADLSHIETRAKVSGHLGPESLEACLTGSDVVLIPAGVPRKPGMTRDDLFNTNAGIVRDLVEACGNFCPKAMICIITNPVNSTVPIAAEVLKKKGVYDPRRLFGVTTLDIVRANTFIAEAKGLDVSKVNVPVIGGHSGVTIVPIISQATPSVSFPSEERKKISVRIQNAGTEVVEAKAGAGSATLSMAFAAARFTSSLLEALDGGEGQVECAYVQSEETDAPFFSTPILLGKNGVEKNLGRGKLIDYEMQLLEEAMPELKANIQKGVDFVSK | — | — | Ldh_1_N (PF00056);Ldh_1_C(PF02866) |
| **Unigene18932** | 8.3901 | FEELNADLFR;IINEPTAAAIAYGLDK;TTPSYVAFTDTER;VEIIANDQGNR | 2 | LFGYKIAAASRGIHLKEKKRAYKNKKEQSPQNRNMAKAPAVGIDLGTTYSCVGVFQHGKVEIIANDQGNRTTPSYVAFTDTERLIGDAAKNQVAMNPVNTVFDAKRLIGRKFDDASVQSDMKHWPFTVINDSSKPKIRVEYKGEQKTFFPEEISSMVLVKMKETAESYLGKTITNSVVTVPAYFNDSQRQATKDAGTISGMNVLRIINEPTAAAIAYGLDKKATGERNVLIFDLGGGTFDVSILTIEDGIFEVKSTSGDTHLGGEDFDNRMVNHFIQEFKRKHKKDISENKRAVRRLRTACERAKRTLSSSTQASVEIDSLFEGIDFYTSITRARFEELNADLFRGTLEPVEKSLRDAKMDKASIHDIVLVGGSTRIPKIQKLLQDFFNGKDLNKSINPDEAVAYGAAVQAAILSGDKSEEVQDLLLLDVAPLSLGIETAGGVMTSLIKRNTTIPTKQTQTFTTYSDNQPGVLIQVYEGERAMTKDNNLLGKFELTGIPPAPRGVPQIEVTFDIDANGILNVSAVDKSTGKENKITITNDKGRLSKEEIERMVNDAEKYKDEDEKQKDRIGAKNSLESYAFNMKSTVEDEKLKDKISEDDKKVIMDKCDEIIKWLDANTLAEKEEFEDKQKELEKTCNPIITKLYQAAGGAPGGAGGMPGGMPNFGGAGGPTGGAGSGGSGGPTIEEVD | heat shock protein 71 [Perna viridis] | ABJ98722.1/0.0 | MreB_Mbl(PF06723) |
| **Unigene7251** | 8.3901 | GPQGPAGEPGPAGPPGPFGER;QGPSGDTGPR | 2 | MMLSRVRWGLFGLLVVIWNVNAQKACKYQGNDIGVEEEFVPDENVPCRTCKFDQDCRLHCDFKSCDPLECLEGQTEVLHPGQCCPVCEELILVDKSLKNPKVDGKLVNDEKAGVWGGAVGPGSANGYVVGAPGPRGFTGPPGPPGPVGYQGPRGEPGEPGQPGPSGERGFPGPSGPPGSPGEEGLPGEQGPTGPIGSLGNAGQPGMPGMPGPKGHRGFPGQTGKTGDEGRPGEKGPAGPSGAPGSPGPMGPRGPPGERGRDGSPGPQGIRGQDGKRGDSGPPGPIGSSGSPGFPGSSGPKGDSGQPGQRGEQGLQGPPGVSGLPGPPGESGNPGRPGQDGDPGIKGDLGQSGAPGAAGFPGPQGPPGQPGEPGTPGPAGESGLAGQDGRQGDPGERGYPGAPGEPGLTGLAGAEGKRGAPGIPGPPGPSGISGERGPPGPAGAPGQVGPAGAKGRDGERGADGERGVAGEPGTPGIPGPPGPVGQRGPIGNTGNEGKPGVQGPAGNSGADGRPGEQGQQGPPGAPGLAGPQGQPGESGPSGRDGESGPPGAQGPRGERGPAGEPGPIGGPGLPGPQGERGSPGPQGETGVQGLAGAAGAPGEPGRTGESGSAGPQGEPGVQGERGEQGFPGDLGPEGKRGPSGERGPQGPAGEPGPAGPPGPFGERGAPGPQGLVGLQGDRGPNGPPGTRGNRGPAGERGKDGAVGIPGEVGPAGLPGPAGPVSLIMPEKGDAGPPGTPGENGLKGDTGPQGYPGNPGPMGPQGAAGPPGVPGEVGSEGRQGPSGDTGPRGYPGEPGPGGETGKNGVDGAQGPPGEIGQQGPAGAPGSPGHPGPPGPQGPQGNTGFVGAPGKAGQRGDRGETGPPGPAGKDGEQGPPGLNGQVGERGNPGQQGPQGPPGPSGPQGERGSPGYPGGQGESGPAGEPGVQGSVGQPGDDGADGIPGPPGPPGPSGQPGFTGPNGEPGAPGLNGLPGAIGQSGDKGSRGPSGPPGLQGPPGPPGQSGPTGNAGPQGERGERGVAGEAGIPGAPGQQGATGATGPAGSPGETGRTGAKGDKGWPGMPGGQGLPGPQGPGGEKGPSGPPGPPGQPGSNGARGNPGRDGEPGPPGMPGRAGSRGPQGDDGLTGPSGPPGPPGPPGSPGYAPVWPGGNWQQQQNKGPDPLYYGDEPDKTPVINDDLSRIQEALHRTKRPSGKKHNPGVTCKDLLLQNPDFEDGWYYIDPNGGSFYDAVEVYCRMNIDGETCIPAGRKLYAEDQWTKVTKSQWFAKEILGGAEFDYKIDDIQLKMLQMHSTKARQRVTYKCMNSDPTGAVLMSNEFESLETLANKQEPYSAVKVEAFGNCDRTSNANRWGEMIFDVKSERSESLPLLDIRLKDVGQSNQEFALSLGEVCFNT | collagen pro alpha-chain [Haliotis discus] | BAA75668.1/0.0 | Signal peptide(1-22)/VWC(SM000214);Collagen (PF01391);Internal repeat 3();Internal repeat 5();Internal repeat 6();COLFI(SM000038) |
| **Unigene37757** | 6.8220 | EGGIPSYTTEYR;YGTDITVPNR | 2 | MEYERKVYRKETTREGGIPSYTTEYRIGTDRPRYGTDITVPNRYTTTTYKTTGPIVYSSYSSRPATEYTTYTEPSVEDTRVRKEWDETFKRVAPRADDWSLSDVISKRMVLVKDDEWDPYETSFPEERKRFGKPSPITSDVSGRKTFLVEYQIGDFRPEEVEIKTIGNTLKIHAKNSDSGSMKREYSREISIPQEVNPDLISAKLNRTGRLSIEAPIFNTSHKTKIDRRIPVLRN | heat shock protein 30C-like [Crassostrea virginica] | XP_022323008.1/5e-16 | HSP20 (PF00011) |
| **Unigene2736** | 8.3901 | NGPFTVLR;TQGSYTEIQECFAR | 2 | RKVMEQKLFLIGVVLMCSLLSVNSLCNFPCSVQTGGDYDGELGSFRWSCDNSSRLIRTQGSYTEIQECFARNGPFTVLRRNGNQYQCVKEAAVTGKVTWVYETEFLTMWNPPTVCSICTPVLMRPMMYVDPSVICIPGTKKKGQYTLKSLKKMKPPPIGCNRPKNCPLSSTLDVPCTGCEPFDDGSCCPGCKRKLQQSYAYAYAYADSMFGPQFMGHYNTML | — | — | Signal peptide(1-24)/~ |
| **Unigene32111** | 8.3901 | IEDAQVPSR;IEDTEVQSR | 2 | SLLGKLKEMKKRIEDAQVPSRDTKEMERKTEDLEKSVFGKLKEMKERFEEVQVPSHDVKEMERKIEEIEQYLFDNLNDLKKRIEDTEVQSRGDVKEMKRRTDDLEISLLGKLKEMKKRIEDAQVPSRDTKEMERKTEDLEKSVFGKLKEMKERFEEVQVPSHDVKEMERKIEEIEQYLFDNLNDLKKRIEDTEVQSRGDVKEMKRRTDDLE | apolipoprotein A1/A4/E [Desulfobacca acetoxidans] | WP_013707457.1/0.007 | Internal repeat |
| **Unigene6047** | 8.3901 | FLEAAYGYR;TFLVWANEEDHLR | 2 | MYRNYITQLDQKKGGQQQGKMAEETQAQLWDKLNKGESKSLLKKHLTPELYEQLKDKKTSLGGTLGDCIRSGANNLDSGVGLYACDPEAYTTFKPLFDAVIKDYHKVDGVNHPKPDFGDVSKLEDLDQYGGDMIVSTRVRVGRSHDGYSFPPCLTKESRKEMMDKTAEACDKLTGDLKGKMYRLESMSKEENQQLIDDHFLFKNDDRFLEAAYGYRDWPNNRGIFHNPSKTFLVWANEEDHLRFISMQKGGNLKEVYGRLVEAIKQLEAKLTFAKKDGYGYLTFCPTNLGTTCRASVHIKIPKLSKLPEFKQICEKHNLQPRGIHGEHTESVGGVFDISNKRRLGLTEFEAIMEMQNGVKEIIKMEKSL | arginine kinase-like protein-1 [Mytilus coruscus] | AKS48144.1/0.0 | ATP-gua_PtransN(PF02807);ATP-gua_Ptrans (PF00217) |
| **CL579.Contig12** | 7.0836 | ILTDDLPR;VAVSPIVTVEPR | 2 | EDEVTGKRITRILTDDLPRYFALVTKIKEEKRQVGDEGGVISSTVVPQVQAVFPEGALTKKIRVGLQAQKINPDLVAKMFGNRVAVSPIVTVEPRRRKFHKDIILTIPVPKAAQKGMINQYGGDAPTLRVLYSIAEGTDAAVWDDYTNNTKLEFSEDCVSFTTRVSARFWLMDCQNIADAPRMATELYHEAITVPYMSRFVVFAKRNAEEEGKLRMFCMTDDKIDKTLEKQEHFVEVARSRDVEVLDGRPQFVEMAGNLIPITKSGDQLYINFKAFRENRLPCTVRIRDMDQDPAARVAFMKEPKVARGEVPQTPICNLNVSLPDMSTSSSMEMDGEAVRELRNRSSLIKEHDIVVQDRVQQASIRLTDVADTVKGDWVMLAQQLDVSSSEINDINNQYKTVNDQGLAMLQLWVDKDTQPNKGQVLEKALVKIGREDVVKKCIYNVENVEDEMEAAAARVAMDQSGFDTFTEEVGISKEDSMKRNMSLDVQFDEQDMVKESESIAESPSSDGKHAPQALEEETEPRDDEVVVQVDERRPLAKQEVKGEKQADDYMDLIEQLDRYCDEKEQKESEGQDTDKPLVFEDEITHTKHERMTPEEIISIVESIGQQSAPPPRQDDDDEGVHTPPPSPAERDQYEDEEEECEETTEIVETYTERIDDNTVRTIRKTTTINSHGTTIRTEILKEEQHRDASLDQLAARLSQTDEPAGGANETGV | titin-like [Mizuhopecten yessoensis] | XP_021347443.1/0.0 | ZU5(PF00791);DEATH(SM000005) |
| **Unigene44023** | 6.2336 | QFYIQVER;VLITTDLLAR | 2 | RYFSENMSYNPEDGDRSRNTNEGPEQKSNEEYSAEAGGVIQSNWDEVVDNFDDMRLKEELLRGIYAYGFEKPSAIQQRAIIPCIKGRDVIAQAQSGTGKTATFSIAILQQLDISQKDCQALVLAPTRELAMQIQKVVIALGDYMGAQCHACIGGTNVREDMHKLSTGVHIVVGTPGRVHDMINRRALNPRAIKLFVLDEADEMLSRGFKDQIYDVFRYMPEDIQVILLSATMPNEVLDVTQKFMRDPIRILVKKEELTLEGIRQFYIQVEREEWKLDTLCDLYETLTITQAVIFCNTRRKVDWLTDKMLSRDFTVSAMHGDMDQKERDVIMREFRTGSSRVLITTDLLARGIDVQQVSLVINYDLPANRENYIHRIGRGGRFGRKGVAINFVTQEDIRTLKDIEQFYNTQIEEMPMDVADLI | eukaryotic initiation factor 4A-I-like [Crassostrea virginica] | XP_022288944.1/0.0 | DEXDc(SM000487);HELICc(SM000490) |
| **Unigene49324** | 8.3901 | FAPINVENTEENR;NSPAENALATVTALSR | 2 | MPVFPQYLSKEQEDELRQIANAIVAPGKGILAADESTGSIGKRFAPINVENTEENRRRYRELLFTCDKSLAENISGVIMFHETFYQKAKDGTPFPKLLQSVGIIPGIKVDKGVVPLAGTDNECTTQGLDGLSERCAQYKKDGAQFAKWRCVLKIQQYTPSYQAMLENANVLARYASICQQNGLVPIVEPEVLPDGEHDLATAQKVTEEVLAFTYKALADHHVFLEGTLLKPNMVTAGMSCAKRNSPAENALATVTALSRAVPPAVPGVTFLSGGQSEDDATVNLNAINTCPGKKPWALTFSFGRALQASVLKAWQGKDENVKGAQDELMKRAKANGLAAVGKFSGGLAGTAGSDSLFVAQHAY | Fructose-bisphosphate aldolase [Crassostrea gigas] | EKC30386.1/0.0 | Glycolytic(PF00274) |
| **Unigene6218** | 6.4703 | FIVNYVR;VAEQQPSPVR | 2 | EVKTTEKTGKVYYQIISKGSIVQTGEIIMGDATVMKAPIMITTEMAPKARFIVNYVRADGEIVTDGVTFSVDGTFKNNVAIRFSKDEALPGSRMLVDLEADAGSQVNVLAVDKSVLLLKSGNDISEDKITEELQSYDNAGGGGFYPVFARMWYWPSTGRDASDVFDNNGMVVLTDAMLYNYEDPFRPMWRRGGAIAFAGAPMPMMAVQNSPPEMAEMARPDTLKAVTKIRKIFPETWLWVNASVMDTGKTTLNVKVPDTITSWVATAFAVNQKSGLGLTPQPANLNVFMPFFVQLVLPYSVVRGELVVLQVNIHNYLPRNEWVLVILEQNMGMHNVITYSNGMRKLAHAKVGRWIKLQGGSIGSAFFPIKPQEVGQLKISVSAKTGSASDAVEKHLLVEPSGTPQEYNVPVLIDLKSVGTFQKKVNITFPKQTSPGSRRVAASVIGDLMGPSINGLDKLIKMPYGCGEQNMLNFAPNIFVRRYLSITNNLKSDMDAKSKEYMVKGYQRELTYQHKDGSFSAFGESDKSGTTWLTSFVVKSFAQAKQYIFIDDELVLKALEWLVKQQNETIGTFDEPGKVLHKAMQGGSAAGERSLTAFVLIAMKEADVIQGASDVTSRSVQKATNFLEGEVDKLIDTYEMAIVSYALKLVNSIKGDIILERLNALATVEDGLKYWEHDVQESKEQKFVSWNPPHSQSNAIDIELTSYILMNYALNKNINNGLPILRWLTTQRNPDGGFSSTQDTIIALQALAEFAGEIYSSDFNMRLTLQSSKGEPFMDSYTITPENALVLKTFDIPVGVEELTVIANGKGVALAEVAVYFHTDSDIKTSSFDINTTLSEETTNGFKLEVCGRYLRKGATGMSLMEIGIPSGMTPDYETLDFKKAPEYKRKEELFRKLVLYFDSFDKEPQCVSLYIIRTDRVAEQQPSPVRIYDYYEPSNQKTNFYTSSKLSDSSLCDVCGKECFCTN | CD109 antigen-like isoform X1 [Crassostrea virginica] | XP_022323515.1/0.0 | A2M_N_2(SM001359);A2M(SM001360);Thiol-ester_cl(PF10569); A2M_comp(PF07678);A2M_recep(SM001361) |
| **CL1804.Contig1** | 8.3901 | GIPGPESSLLNPELLR;QPGSATGLIQDR | 2 | DALALLHQDDAIPSAHDGPNFLSWHRYFLVLFEEALRTKNPKMALPYWDSTLDFRMVDPSKSIYWTEIFVGNPTGVVYTGPFADWITPANNTLLRRGIPGPESSLLNPELLREVFKQKYHYQILFPTNVSLHSNFETHHDGVHRWIGGQDGHMGGLSTSPQEPAFWNFHCHIDYLWEQFREQQRRRGIDSSRDYPLMDIPVHQPFRKMDNLWPPLRNIDGYSNVFTQYIYRYEAAPTCRNKCGGAKTEYLYCNRRINQCVSRSRSTFERLTGIGRSRQPGSATGLIQDRVASLQPERNVSLSSLADRPNTEPKFRKAIVDPRTT | tyrosinase-like protein-1 [Mytilus coruscus] | AKS48166.1/3e-98 | Tyrosinase(PF00264) |
| **Unigene33532** | 8.3901 | FYTDAVWMK;SVPSAVSTIPEWLTEAK | 2 | MALQSLAVLALTLLGVESAFNATSCGEYKCPEFTVDFTGNGFERRFYTDAVWMKASAIGNYKDATKNTFSRLRKYMNGDNNKNTAIPISIPIVTMMWPVNDTAVLNNFTQFVFIEPASTPAPEPNDKTITKTEVPPKHMYIRSVPSAVSTIPEWLTEAKKLKDAINDEKKYWGSFFFIAEYNRDSPNQNDHYNEVWFYAF | heme-binding protein 2-like [Crassostrea virginica] | XP_022345420.1/5e-28 | Signal peptide(1-18)/SOUL(PF04832) |
| **Unigene51584** | 8.3901 | ACPSGTVFNAR;KPLGGLLETR | 2 | TTHLLENVLTGKPINDKQIVSTIQQPKQVADTAGNDVTTGDLKAEFQDLGITPSKKETGNNLLSREADHIGNLDQDPIQDVTETIQQTETSGLALESLCEHSSFIDSIGYAEYPGRCDKLVQCYNYRGRDIAVLRDCPYGYFWHQEQVLCRPPAEVPCYDDPCLDIDIVQYNRSGGCRSYYACEYGISIPQCCDKGFRFNGRRCIPDATCYDPCSTPFDIEQQMRYQPCKFLPDEYNPYGYITMEHNGLRIRACPSGTVFNARDCGCRSIGKGSPKYRQSRRRMRQCKPQFAMDFDQGFKEDSGSNMAFDVNNVVITNRAAQFDGKGKITLWGFMNRELGTRFAIRLRFKPDPTSTDIGSLISNCGMTGSPTVSIDLQKHMLVSSAKSKSFTEPTKIKDHFDPYSWNDITYVYDGRNFMVLTNGYKQRKPLGGLLETRSNAIVIGGCPRPGKGYKGLIDSVEIYSGCIPRDILKKRNRR | mucin-2-like [Crassostrea virginica] | XP_022335717.1/ 6e-134 | ChtBD2(SM000494); LamG(SM000282) |
| **Unigene15131** | 7.0836 | LLVAENMIVAK;LPDAVLNVVR | 2 | NRELCAMFKTILILCIIGSSLGQPGGWQDYHGKLPDAVLNVVRKRLLVAENMIVAKMETRNVKSQVVAGVNYKFDVKVTGVSGRIVDCHFLVYVNLSGIAKVKSHKC | 2-oxoglutarate dehydrogenase [Halioglobus sp. HI00S01] | KZX60278.1/0.011 | Signal peptide(1-22)/scop d1stfi_ |
| **Unigene41665** | 7.0836 | SIGNFIYNSDEGTVLGR;VYDWQPEPYSER | 2 | MADRGQKETIGDKARSIGNFIYNSDEGTVLGRGAKSWGEIGLFYMIYYACLAGFFAAALAIFYQLVDDKVPNLYGADSLLKANPALGFRPMPKLTTTVIRFDTSEKSYQSFVDHLDSFLEPYANQAATVCPGDNAPRPESMWDQACQFPLSLVGTECAHNSKYGYDKGKPCVLIKMNRVYDWQPEPYSERLNDTQAVPAEIRDLYKDYSVTVKCQGENPADRDNLGTVKYFPEGGFPYKYFPYMNQANYVSPVVMVQFTNPKVQTLLMVECRVYAKNIKYDKMALDGAVHFELMVDYPPHNETNVAGK | Na+/K+ ATPase beta subunit [Doryteuthis opalescens] | ABO61331.1/9e-80 | Na_K-ATPase(PF00287) |
| **Unigene40680** | 7.0836 | FPTCENIR;GYNSEVTER | 2 | ISSPPKINVPKRFQDVAIFEKGENVVLKIPFTGSPKPTITWLHDGEEVKGRGYNSEVTERHAILTIKDAGKDQDGPYRITAENSLGSDSAVIKIQINDRPDPPRFPTCENIRDDSVLLTWKPPLNDGGSFITQYVIEKCEPPNKNWIRVATSRMCFFNITSLTPEHEYQFRVIAENFYGSSDPCEPTPVIKTDIPDAIKKKRAMEDEFGRKVRGTGPKVDNYDKYYHDLWKKYVPQPVSVKTGNVSDYYDILEELGSGAFGVVHRCVEKSTGRVFVAKFINTPYPLDKYAVKNEINVMNQCHHPKLLQLKDAFEDKY | twitchin [Mytilus galloprovincialis] | BAC00784.1/0.0 | IGc2(SM000408);FN3(SM000060);Pkinase_Tyr(PF07714) |
| **Unigene31729** | 6.3126 | GESEALR;SVGIHLFCVGVGR | 2 | SGRQKTAQIGVIVTDGRSKYPDASRGESEALRSVGIHLFCVGVGRLVDEKQLRSMAFKPNEYFYIEPIDLAVDKLVQSVRKLTSASTAKIPASADHVGMPASEISPKFNALPTKTTIATTITTTKSTTMKTTTTTRK | sushi-like protein [Mytilus coruscus] | AKS48157.1/2e-37 | VWA(PF00092) |
| **Unigene2452** | 8.3901 | IQTQPGWSSTLR;LGLLGMWK | 2 | LLISPFRRNTPRRRFKMEAISKAVKNNIFSSPFQQAKCEGLKVNETRTIAAASANAEEISCEFGSNKYYALCGFGGILSCGITHTAVVPLDLVKCRIQVDPGKYGGIVNGFKISVKEGGAKELTKGWAPTLIGYSMQGLCKFGFYEVFKIVYGNMLGEENTFLWRTSLYLAASASAEFFADIALCPMEACKVRIQTQPGWSSTLREGFPRILKEEGVSGFYKGLVPLWARQIPYTMMKFACFERTLEALYKYVVPKPRSECNKTEQLMVTFSAGYIAGVFCAIVSHPADTIVSKLNQDKGSNFVDIAKRLGLLGMWKGLFPRIIMIGTLTALQWFIYDSVKVYFRLPRPPPPEMPESLKRKMGIA | mCG10343, isoform CRA_c [Mus musculus] | EDL21528.1/5e-177 | Mito_carr(PF00153) |
| **Unigene34954** | 5.2978 | NAPEMPPPR;QPFPMQQQIENTRPSR | 2 | QKQSALENFQVIAESILKSIPPSHPTHMQPPFSQRNAPEMPPPRQPFPMQQQIENTRPSREWSRQILPPPDRNVQRVSPKPTELPHQPPANLQTPQTKQPNPIPEKNILSDKNSGNTIVERKPDKLKNTKLFTPDLKQTQTQAKPQIQPHWLVWKDPRTAGQNSAIQGEKNLEPKPTPKPQAPAKSKILQTAKNQIKQQIQTEKIKQTIQNSIENQQQKEIPVEKKEPTLEDLENGNTEMSATKLLKLIKKKLKQAKKTGNIPFSLLKILLEKRKTLIAKLKEERALKKKKEAESNKEAENKKNNEQNDIQIKQVTKDKKKEERPVKQEEVKTTTITTLKPTTTTVTTTTIQTTTTTPTTTTTPSTTPTTTTQTTTTTTTKPPPTTTKTTHPPPTTPPTMATNIVQDQFDENSKQTNLAPPIIGIVQDKQIQNNDNVEETYKFTEQLTQANAQSSANTITAFSSAQKSASYDVVAQ | — | — | Thr(15.1%);Lys(12.4%);Pro(10.9%);Gln(10.7%) |
| **Unigene5067** | 5.5154 | QMSLFPSFK;YDWDFFDR | 2 | MSGRLVPVHRYDWDFFDRQMSLFPSFKDDFDKDFFSDFKSTKIEDEIARMKREMFQLTSPEQSLKVDQPFVEDFTGNKKMALRFDCSKFKPEEIEVKTVDRSLTVHAKHEEKSPGRSVYREFTKSYTLPRDIDPLSLKSSLTNDGFLQVEAPAPKTCIARKEIFIPIEKMLK | small heat shock protein 22 [Mytilus galloprovincialis] | AEP02967.1 /2e-92 | HSP20(PF00011) |
| **CL2840.Contig1** | 8.3901 | ALVDTETFASPR;ALVDTETFASPRSAVTSR;ASSYLDDIYYPEPIVR;EATRALVDTETFASPR;EMEVEAVPVQSTTSLK;FQSVPPGYFSSTK;GFYDTTR;GFYDTTREENEIR;GSSQAALVGNR;GSSQAALVGNRVEVVTPR;TPTPVTTSR;VEVVTPR;VTQPTNLMSWQYR;WYPTTTR;YLPDEDSK | 2 | MTVRRSRFQSVPPGYFSSTKGHSSLKRWYPTTTRASSYLDDIYYPEPIVRSRGFYDTTREENEIRRDVNHELLYTSNLVDDTYDIANKSRNRDQMLLREATRALVDTETFASPRSAVTSRRVRQTSVVRTPTPVTTSRAVSCPPVSRGSSQAALVGNRVEVVTPRKRKPRSTYAANKMRELKRDEREMEVEAVPVQSTTSLKASTVSPNYKGGKLHWDEDGKVTQPTNLMSWQYRIESRVPPGDHLFPVKTIGHVRDKLLHVKEQMDRHRQLMDRYLPDEDSKTDVKTKIMNMYVDMEQHNPAS | RS-rich protein-1 [Mytilus coruscus] | AKS48138.1 /4e-86 | Arg(11.2%) |
| **Unigene31135** | 6.8220 | DPCFDFTVR;GSQYNPGLCTCDR | 2 | GPTADRVNDELTPSRQWPTREATDMKSPSSSLLQAENGKKEPSAGWQTQDTIDNNQDSSRQWQTDRTIDSNNGPQRQNPTRVSVDNRLYRHREEDRDTNKLLKISVEANLLNGKDIVGTKIEKFVGGDPWWTQNTNPNYEVQDPTYQQPSFNEQKWYKQSDPIKAPWVNIPVQKSTPAAWKTYAPSTPSYQVYTKPQTETQTTTSAWASEYRNPLISPVTLFSINGPVDNRRTQPVYPRRPIPRFLTTTSTTTTTTTTTTPRAPVVSEPQPFAVGPAQGKDRIEEEHLCYGSQYVDGVGYNADPKSCNEFIQCFYEGGQIKVERARCPFRMFWDQRLLLCRPSGSMSCWRDPCFDFTVRSYDHKGNCRAHWSCRNGLSQAKCCPDGFAFNEEKQECVPDPGCHDWCEDSGELTFTSPFASCRMTPFETNERFYFEEIPGLGMMTRSCPRGSQYNPGLCTCDRSEEVRKNSCRPELILDFNDGFSDKSGNNLAVTPENVHIENGAARFDGSGKIRLWRFSGVSYGSQFAVTMKYKENPHVQSAEPMHLLSNCFNPEVMRPSIDIAISPNKGAMFLATTDKGGEQMSVMKYNSTSWKTLSFVYDGRHLIGNIDGKKTTVPLTGTIEERPSALILGACDYFGGYIGMMDDVKLYLCVPHGIT | sushi-like protein [Mytilus coruscus] | AKS48157.1 /0.0 | ChtBD2(SM000494) |
| **Unigene7385** | 4.1951 | IPAINVNDSVTK | 1 | MSTKPPFKVADINLADWGRKCIEIAENEMPGLMQMRKMYGETKPLKGARVAGCLHMTTQTAVLIETLTALGAQVQWSSCNIFSTQDFAAAAIAKTGVPVYAWKGETDEEYIWCIEQTLVFPDGQPLNMILDDGGDLTNLVHERFPQYLPGIVGLSEETTTGVHNLHKMMKDGKLKIPAINVNDSVTKSKFDNLYGCRESLVDGIKRATDVMLAGKVAMVAGYGDVGKGCAHALRAFGARVMVVEIDPIIALQAAMEGFEVTTVEECLPKCRLFVTATGCSSIIHDKMFEQMLEDSIVCNIGHFDCELDVKWLNENCAKKEQIKPQVDRYTLKNGRHVILLAEGRLVNLGCAHGHPSFVMSNSFTNQVLAQIELWTKKEEYKNKISVTVLPKKLDEAVAAAHLDHLGVKLTKLTEEQSSYLGIPRDGPFKPEIYRY | S-adenosylhomocysteine hydrolase [Crassostrea ariakensis] | ACT35639.1 /0.0 | AdoHcyase_NAD(SM000997) |
| **Unigene2230** | 4.1951 | QTATQVFR | 1 | MKKGKKKKVAAAPSVAKKAIETKKAVNPLIEKRPRNFGIGQDIQPKRDLSRFVRWPKYIRLQRQKAVLLRRLKVPPPINQFRQTLDRQTATQVFRLLEKYKPETKQQKKQRLKERAEKRADGKDDKPTKRPPVVRSGINTVTTLVEQKKAQLVVIAHDVEPIEIVIFLPALCRKMGVPYCIVKGKNRLGRVVHRKTCSSLCLTTVNSEDKTALNKVTEAVKTNFNDRGDEIRKHWGGGIMGNKSQARITKLEKLRAKELAQKMG | 60S ribosomal protein L7a [Crassostrea gigas] | EKC37537.1 /8e-155 | Ribosomal_L7Ae(PF01248) |
| **CL4205.Contig2** | 4.1951 | EMEAELEDER | 1 | EAMTIMGICPEDQAALLRVISAVLMFGNMQFKQERSSDQATMPDDTVAQKSCHLLGLSVTNMTQAFLRPRIKVGRDFVTKAQTKEQVEFAVEAIAKACYERMFRWLVTRINRSLDRTKRQGASFIGILDIAGFEIFKMNSFEQLCINYTNEKLQQLFNHTMFILEQEEYQREGIEWKFIDFGLDLQPTIDLLEKPMGVLALLDEECWFPKATDKSFIEKVKSQHSTHPKFKKPDFRADADFSLIHYAGMVDYCASNWLTKNMDPLNENVVSLLQNSSDPFVAAIWKDAEIVGMGATAGGDTMFGSRTRKGMFRTVGQLYKEQLAKLMATLRNTNPNFVRCIIPNHEKKAGKIDSPLVLEQLRCNGVLEGIRICRQGFPNRILFQEFRQRYEILCPNTIPKGFMDGKKAVEKMIKALELDPNLYRIGQSKIFFRAGVLAHLEEERDLKLTDVIIQFQSLCRGLLARRNYQKRLQQLSAIRVIQRNCAAYLKLRNWAWWRLFTKVKPLLPVTGQEEKLTVKEEELRKVSEAFDKSKNEVQELERKYAQIIEEKSILAEQLQAETEMCAEAEESRARMAAKKQELEDILHDLELRIEEEEDKCNQLIDERKKFQQTVNDLEEQLEEEEQSRQKLQLEKVSADSKIKKLEEDLAIQDDSNQKLTKEKRFLDERVSELQTQLVEEEEKSKQMTKLKNKYEQIIKDLEEKLRKEQQARQELEKIKRRLETELNDLREQLNEKRQQLEDLQYQLSKREEEVQHALQNAEEESVGKAATMKQVREIQNQIQELQEDLDAERESRNKAEKQKRDISEELEALKTEFEESLDATAAVQELRNKREDELRDLKKSLDDAQKKYEGNLYELRSKYNQQAEGLNEELENVKKSKSSLEKMKQTLEHENSDLANDLKAVQMAKQESERKRRQLEQNVQELNVKLTEVERMKGDSSDRATKLQNELDQINSQLEQSDAKLLQTNAKNSSLEAQLTEINEQLQEETKGKLSVQSRLRQAEDDNRALKDQLEEEEDQKMALQKQISDLQFKVTEYKKKAEDETKVQEALDEYKKKTDREMEAMNAKLDEMKAVNDRLEKSKKKLQQEVEDANVELESQRSSFTQMERKQRKFDQMLAEEKAVSEKLALDRDQAEKDSRDKETRILNMARELDDLREQYDKQRSLTVSQQRELDDLMSSKDDVGKNVHDLEKAKRTLDAMLEEQKQKIEELEDDLQTTEDAKLRLEVNMQAMKAQHDRDLLAKEETVEEQKKSLLRQLREMEAELEDERKQKASAVNSRKKLENDIKDLQQQAELAVRVKEDAVKQLKRCQAQMKDNMRELEEARQSRDEMQAAMKDMERKLKNYEADLLKLQEDLAASERQRRNAEAERDELQDEMGSNAAGRSAFLDEKRKLEARIAALEDELEDEQTNSESLLDKARRSQLQLDQLTADLNSEKSVTQKLENQRTTLERQNKELKEKLNELENTLRTRTKATIASLEGKISSLEEQLDIESKERSGMQKVNRRLERKLKEMSVQAEEERRHADQNKEQADKMMNRVKALKRQVDEAEEEITRLNAQKRKIQRELDEQMEQNESAAREISQLKKYRPSASRAGARSTVLSSRIADLDDDDDQDETKENAEA | myosin heavy chain, non-muscle-like isoform X5 [Mizuhopecten yessoensis] | XP_021350593.1 /0.0 | MYSc(SM000242);IQ(SM000015);Myosin_tail_1 (PF01576) |
| **Unigene44069** | 4.1951 | ESLPLIVFLR | 1 | MGYRGRRKHLKRLTAPKSWMLDKLGGVFAPRPSSGPHKLRESLPLIVFLRNRLKYALTHDEVKKIVMQRLIKVDGKVRTDMGFPAGFMDVVTIDKTSENFRLLYDVKGRFAVHRIKPEEAKYKLCRVKKLGVALKGVPYVITHDGRTIRYPDPLVKVNDSIMVDIATGKIKDFIKFDSGNLCMITGGHNLGRVGVIQHRERHPGSFDIVHIKDSLGHTFATRLSYVFVIGKGNKPYVSLPRGKGVKLSIAEERDRRIAARS | 40S ribosomal protein S4-like [Crassostrea virginica] | XP_022290789.1 /4e-172 | RS4NT(PF08071);S4(SM000363);KOW(PF00467);40S_S4_C (PF16121) |
| **CL3460.Contig1** | 2.1035 | TLPTLIR | 1 | MLQTAMEVDTGWDSISILMADEAGKWMDSIINLKNSVIGNNKYKSLAVQQGIIPRLLQWIIDDGVPIELRTEAAIVLGSLAKGTEQDLVMLVSAGSVSVLLIGITNNNVKFVEACLRCLKTIYLMNDPPIQLIFENKTVIPHMINIISKSICTQECITTIFSRCCTMKEHQEKLCMNGALATLAPLLTSSIYKVQMPTLKSIAVLCYQNEDVAKATATATYNGESIPSLLLKLLARDKTSEMQMAAARCLTYLCRGGALQPSSNVIMYKTLPTLIRMCKKDRTLEENVEGAETLAYLIEEDPDLQAMASISDHIIKTLAEYLRYTDVQQINSRVTQKKEINWSNELRQAAFRAFASLGANDEDIRKKIIETENLMDHIISGMNSDDVKVKGAAVRCLHSLSRSVQQLRTTFQDHVIWKPLMNMIQNGPEELLMITSSTLCNLLLEFSPGREVSNPRKAILDAGAISILVTLTARQEPELRLNGIWGLMNMAFQSELKVKSQIIEAVGTEQLFKLLSDPDPNILIRTLGLIRNLLSGKNHIDSIMSVYGTQIMQAVVFILEGDHASDIKEQTLCILTNVADGDDAKGYIIENEDVLKKLMTYMMHTNVKLQISATTCISNLVWNEDEGAYKRQGKLREMGVQKILQQLLSSNDAILFDRVKDALKQFG | armadillo repeat-containing protein 8-like [Mizuhopecten yessoensis] | XP_021358110.1 /0.0 | ARM(SM000185) |
| **Unigene27989** | 4.1951 | SGFGAAGFGVGGGR | 1 | MGSGKGMGNGGRGMHMEHGMGSGGDGMGMGNGGVGGSLNVWDHLSSNTGSSGSGSGRNPANNGRSGFGAAGFGVGGGRTLSNMGGSMHKNGGMGSGLASHGTNVWNRVNGQSNNKPMSNSGSVLSGPGGARNNGVANMNNGGTPLVDQGTKSKPVEHPPTPPASMSSVGGGFGRASDISFHTMGSMLQPNQGGSVSQPQSKNQGQTQPKQPGNATPQVSNQNPANSGSSGGGVGVNPQTNQPATNSQNSGPTGNNIPGINNGGSQGGNVGGSHGGNMGGSQGGNMGGSPQIETTGGATSAPNHNFGQGNSFFGHGTNNQGNLGTNNVVGTTAYPGFTSMATWMFLK | — | — | Signal peptide(1-36)/Gly(25.1%);Asn(13.0%);Ser(12.1%) |
| **CL2407.Contig1** | 2.8885 | LLELFPLNK | 1 | PRKKRHKACPALLFHLKFRIFMSQKAEKPSLSGTRLKTRKRDEKEKYDPSLFRDAIITGLNETDGDLEQVSKYLDTAGAKLDYRRYAEVLFDILVAGGQLAPGGSIVEDPDPNKLSKTHVCVFGLEESTEKIRSFYEVYYKLLRRYKYLEKSFEESLKKLILFLKGYHESDQRKLATITGIFLATGFCSAKVLSSLFEHHLVKDGLSLDFSTVMFRVWLSERDIQAINGTLKKAQIDGRLLELFPLNKRTLENFVDHFEKAKLSQIADMQKAISTSGVRKGLQKQLGEMMKDEESVEDMIKFVQEQIQTNSLSDSDITIMVWNTIMNAVEWNKKEELVADQAIKHIKIYCPLLAAVAKSGKAELTLMVKIQEYCYGNVNFMKSFQKIIVMLYKNDVLSEDVILKWYKEGHSPKGKSTFLEQMKKFIEWLENAEEESDEED | basic leucine zipper and W2 domain-containing protein 1-like [Crassostrea virginica] | XP_022295017.1 /0.0 | eIF5C(SM000515) |
| **Unigene20448** | 4.1951 | APGQAWGFR | 1 | MATVQVQRAPGQAWGFRLAGGRDFNVPLQIKKVEQGSPVAGVLSPGDNIIGIGHSDARNMTHMQANQMIRGAGNMLQLTIVKGHGDVNSRISSIKPKGPVKFSPWKAQST | PDZ and LIM domain protein 3-like [Mizuhopecten yessoensis] | XP_021353042.1 /8e-36 | PDZ(SM000228) |
| **Unigene635** | 2.1035 | VNLFVR | 1 | ICTASSAAGIDRDYVDLKVQGVLPDGNGPIKIDTQTVNIGERVEMECVVTGEPRPTVSWSRVGEPIPDTATVNDVFLVIPQVRIEDAGTYVCTAQNLGGIVQQRVNLFVRARPIISGSQA | heparan sulfate proteoglycan-like protein-1 [Mytilus coruscus] | AKS48136.1 /3e-57 | IGc2(SM000408) |
| **Unigene38686** | 4.1951 | LTHIFPDGR | 1 | RSNMIRKAALFLVILTVSCNLSKSSKHYLGYDSSDTNRINLYPTKLTVYKNNPRLLGITYKLTGMEKYHLLASDKISGIWFVDDQPIFQSSSSTGFQFQTSTRFWGHTWCESYLDSNIPRVELANKTLKHVAHLVRVDDPEVKTMVDYRVVKYEVTIDGNDVVFKIEDKKDKVDIAFEKLRYKVYYFEEHNEFLVNVEYPPQSLSSINTVEVALEGLITPEGGDPELVKSISSCSNGKCRNYNGHVLLYDISKSLLSFQENIFVLIPDDVVNKTQGLVTIAVESTNAIGSKFKVSFDVTSQLFSEIYSIKRFLFFPTILDLQVTKGNGGYTVACRALGTYNVIHHGIRLTHIFPDGRLVHLTDFYSTFDVYSTTKYYTLPTDTDIKGTYICSIMEHMANTYLDFDTKYFYVM | — | — | Signal peptide(1-24)/~ |
| **Unigene10315** | 2.2416 | TGIDISK | 1 | SGNPSNSSSSQNDNNSADPMQADSKSDMSSASCSSDVYESARDTSGVPDCKISSKKSDQSPNINQESNNNGTVQVSKKITIPDYVKKEWKGQTDRASTIRKAYEEIPKQTGCNHLCRVRGDNYCGIRGTLFQCLRTGIDISKHWSDYNTITDLSKKYADENSGLKLWTFAKRLSCTEGQRLTLMRQCLSMLFSKFEEAKAIES | ubiquitin thioesterase otulin-like [Crassostrea virginica] | XP_022308633.1 /2e-32 | Signal peptide(1-16)/Peptidase_C101 (PF16218) |
| **Unigene10904** | 2.4029 | YVTFNLR | 1 | MAGQVIQMSICLLLVCICGIHATIYINTTLPDEAFNSENQQKPQCELKKLTVEHVNGINNLLVTFKISLKNRLIDTQWDGNWFVNGSKVTAIPYFFNHSKFAHSTKHYWENDYVLVVFILSSTLPAEQLRYVTFNLRVQFSESDDLAKRSTGQILWFYRILSYNIKLYGDHIEFPSAGKVDNKFDKLQYSIRKNKDARNYQTLFSFSKFSKSYVSMMSAKFEGLNANSVQMRYTSGKSSCGAVCYNDSELVKMSKKSTGYRKTRSQANYVELSTDTSGTYEGVYTVSAKTRNRLNSDITLTFDLTSHLYDGIGPIFLGNRFLYITNFSMSMLSDKKAMLTCRVLGNTYNDVTLYKVNDQGQRDEIQSVLFNNPLSVYVKTLVYEFAWSMKNRGTYICRVTRQHYPYNTEKTLSF | — | — | Signal peptide(1-22)/IG_like(SM000410) |
| **CL3182.Contig2** | 4.1951 | AADTIANILSANR | 1 | MNLSFHVFFFSLLLAGDVLASGSDKFTSTRSGVYFYGRAADTIANILSANRKQWNGEKCQDARPSDCGELDKRVCPSGIYTIYPDKSSGFDVFCDMEKDGGGWTVFQRRQNGLVNFYRDWEAYKEGFGSLRGEFWLGNDHINKITSQEIYTLRVDLVDFKNNHKYAKYSKFSVGPLNSEYKLDVGGYSGNAGNDFGGHDGQKFSTKDKDNDVYSGNCAESYKGGWWYSSCHSSNLNGLYLRGNHTSFADGINWKTWTGYNYSLKATTMMIKRTKLF | fibrinogen C domain-containing protein 1-B-like [Lingula anatina] | XP_013401135.1 /2e-80 | Signal peptide(1-20)/FBG(SM000186) |
| **Unigene17031** | 2.6269 | AAVQEEVQPLDK | 1 | DESHVVNPADVIVPTLVPSRVPPTRAAVQEEVQPLDKVQKQQTTPPVTIFKMKIIQTVVNSNVDQDSEKKIQHTVRVETPATKNDISVNIEHTSKNIDNVGSGLQSSVVPNQDTMTRERMGILDRKPPTISNKEDSSDSIEDVVHELTPIPEMEIIHPNQEDEVKSILSVYDNVTSTKDSSSSEEVKIQGPKLVSETETEVESSESVSSVSEQSEESFPAIEQTSIEQGSESSSEQTISEIKTTAKSNTSTKPPFRISEKTTTKAPNLRSPESRHHKGSGSSGSRTECSNVLVSFFASLIYFLLIT | — | — | Ser(15.4%);Glu(10.5%);Val(10.1%) |
| **CL79.Contig3** | 2.0605 | VGSISDYDPMNDR | 1 | MNPPCAKCSKTVYPTEKLNCLDKIWHKGCFKCQVCNMTLNMKNYKGYDKLPYCNAHYPTTKHTAVADTPENRRIADNTKIQSNIKYHEDFERQKGKKLTVVDDPETQRVRQNTANISQVVYSGHKDQLKDMEYNRPAEQVNDVRVRPNPGSIHSYDPMRDQQQNQGTPYSQRNSGAQVYDSNTGREDRNFNARIQPFYQHPAYGMDNPSSQRRVGSISDYDPMNDRWGSVAGQFNAQNQQQRNQPPPPQQYAPPPQEADRFAGKGDKNDDDLSYGYDTTGKGMVCRAAYDYVAADDDEVSFNEGDFIIFCQPIDAGWMEGTVEATGRRGMLPSNYVETVKK | LIM and SH3 domain protein F42H10.3 isoform X6 [Lingula anatina] | XP_023930739.1/3e-101 | LIM(SM000132);NEBU(SM000227);SH3(SM000326) |
| **Unigene10230** | 4.1951 | VQYSNSQNNLYHHQTR | 1 | NFPTPSATSYRTSETNKMSKQKRHRNDHINNAQMSQNSQISKRRQYLATRQRTQQPTQRRQKQVSRQIVAQRRQQSVSRQRVKSTTQQRQQPIQRKGVQQMSNQMSYESMIDSKNEITALILKYMASLPMDTAMETAIRECDGYARPVCKALPMWTNNRPFDEWCSVLCPTGVCPAAVCSCTCPQQNSRYQQNRVQYSNSQNNLYHHQTRIRCKAISVWGDPSMDQWCSSTCNANPDNCPTDHCWCDGL | chitin binding protein [Pinctada martensii] | AIF72920.1 /6e-14 | Internal repeat |
| **Unigene20294** | 4.1951 | MGHAGAIIAGGK | 1 | PVLTMSTATKVLGKVSAIGRVGIRTCYTNSRPNLGINKKTKVICQGFTGKQGTFHSQQAIEYGTKMVGGVSPGKGGQKHLGLPVFNSVKEAREQTGADASAIYVPPPFAAAAIIEAIDAEVPLIVCITEGIPQQDMVKVKHKLIRQSKSRLVGPNCPGIIKPGECKIGIMPGHIHKRGKIGIVSRSGTLTYEAVHQTTQAGLGQSLCVGIGGDPFNGTNFIDCLEVFLQDPQTHGIVLIGEIGGQAEEKASEYLRNNNCGSDAKPVISFIAGVTAPPGRRMGHAGAIIAGGKGGADEKIEALREAGVDVTMSPAQLGTTMAKAMSAAGKL | succinate--CoA ligase [ADP/GDP-forming] subunit alpha, mitochondrial-like isoform X1 [Mizuhopecten yessoensis] | XP_021357524.1 /0.0 | CoA_binding(SM000881);Ligase_CoA (PF00549) |
| **CL1802.Contig1** | 2.4029 | MLQEDELR | 1 | MGNVFQKLLGNLFGKKEMRILMVGLDAAGKTTILYKLKLGEIVTTIPTIGFNVETVEYKNISFTVWDVGGQDKIRPLWRHYFQNTQGLIFVVDSNDRERIGEAREELNRMLQEDELRDAVLLVFSNKQDLPNAMNAAEVTDKMGLHQLRNRQWYIQATCATSGDGLYEGLDWLSNTLKRAS | ADP-ribosylation factor 1-like 2 isoform X1 [Exaiptasia pallida] | XP_020917326.1 /2e-120 | ARF(SM000177) |
| **Unigene14034** | 4.1951 | DTDIAGVTQDSR | 1 | ASHQQNQQQQQQIQHTEHREYKYNTQNFNYNQQPNQQFMTGSQQHINMTEGMPAQNIPIQHQQGRYQQQQNQHQQQLQYHTGSQQNINNMLDGMPVQNMPIQHNHGGSQFNTMSSGHGSVSSPQGFNTLGSNQSYRTEQHQKYHTMSSSGSHDGFGTMGSQNFGTMSSGHGYNTMGSQQNGSLHVDTTNRSMHSGPSSAGSPHSPDTLNALRQQLHVAHNMSSSSGGALSPGPHSMTGQSSPSVYFGMSRRGSLTSLADTADAVHATPKFVKNTSKYWYMPNITREEAISMLKDKAPGTFVVRDSNSFPGAFGLALKVATIPPNVQTKSSGDPAADLVRHFLIEPTPKGVRLRGCSNEPVFGSLASLVYQHSITPLALPCKLVLPEVDPSIESSMDVTHTSEQPSSAAALLAQGAACNVLYINSIDTESLTGPQAVARALKLTFDTAPSPTTTVVHFKVSNQGITLTDNQRKLFFRRHYPVSAVTYCGMDPESRKWKRDTDIAGVTQDSRVFGFVARKHSGASDNACHLFAELDPEQPASAIVNFVTKIMIGQGSKK | Tensin-1 [Mizuhopecten yessoensis] | OWF49222.1/3e-175 | Internal repeat;SH2(SM000252);PTB(SM000462) |
| **Unigene19402** | 4.1951 | AAIESAFQDYR | 1 | MADYGEPISDTEKVKIASDFILHAPPGEFNEVFNDVRILLNDDNLLKEGASGAFAQYNKDQFTPCKVEGSQENVLITEHGDLGGSRFLDPRTKKSFRYDHLRKEASDYQPGDIDRNAEKWRAAIESAFQDYRSKHYKQGVITVYGSSSGGNITIIACLESHQFQPKNFWNGRWRSQWSVTFPESGGKCEMTGILKVQVHYYEDGNVQLVSSKEVKESLTVTSDHQTAKEFVNIIADAEKGYQSAIMENYLMMSDTTFKALRRQLPVTRTKIDWNKIISYSVGSQLGQGKK | F-actin-capping protein subunit alpha-1-like isoform X2 [Crassostrea virginica] | XP_022298240.1 /1e-169 | F-actin_cap_A( PF01267) |
| **CL2758.Contig1** | 2.1175 | SAVVAAER | 1 | MATNILCFGFLISTTILKLVNGAAPISAKGGGSNSFVFSAKNKFSFSNPSPPTSSQGQGFSSSSSSSSSSSNAGSGGGFSPNNNQNQWKQTLPINYEPNKQGNNQQPNQQNGNQLSSQQQQQQQQQQQQSGQQHQQQTQMQHQNMQMHPNNQFGQPQTKPMNQNQKFNSPGQMGSGQQWQPMGFGQQQQQQPQNPQHQQQNTFSNPQQPQNGFSNPQQNSFSNPQQHQLSNMNTGQTMFQQPPNQQQKQQFGQPNQIQNSHMTPFGQQQQQQQQQQQRNQFNKFGQPQSGSNMNVLSQGGQPNNGMPPYQGMPLTANNQQGFQGQRPQQQQQSGHMTQYFPPHQSQQTNQMHGPGQNQGHQGQMPMNGNMPMQGSPQNMQMGSHQQPMNGQLPAHMQGGPQSMPHPSGSSAPAHQQMGRPHPPIGGAINPPMGGAINPPHGGPQMGGPGHSPHGGPYNPNQGGPGYPPQGHGPRPGHPNHNVAVIDHPRKTTTTPKPQTSTIDGEGMVCMTTADCEIGCCFNATGQLLDTTTYGAGGPKEGRASGKCFIRKPGLGDVCDDLCACTMGHDCYRRYVPVYPKPGQKTAPVIDPEAAPKPQRTCVRSAVVAAERIAFWSCYFDVSCSGPLP | extensin-like [Mizuhopecten yessoensis] | XP_021354369.1 /3e-34 | Signal peptide(1-20)/Gln(19.6%);Gly(12.1%);Pro(11.6%) |
| **CL3080.Contig2** | 2.2753 | LLPAGFGIYR | 1 | TCSLALVLFFSISCAQDTRNMQRFQSCRDVNLCCPGRNNTCYAIGPRMDGNFNESKCFCDDNCKAMKDCCTDHESYCQYQRGQDCVLSEWTQWTECNSIHRCGKGIQKRKRHVIESAAHGGKPCGHQRQKRVCYIDDCTEIQSVEYSGRELKEVGRLLPAGFGIYRISEEYNPHKDIRKNLPFFQDLKNEVPTVTAYCGTYKITESSTRCTNNTQEHQHWATYLKAGHEVCVECQPFAMHKHLGMRCKGHGVPGQSTRWRAIDVPHCYGRWELIKREKCTCNIHQNTSFILI | somatomedin-B and thrombospondin type-1 domain-containing protein-like [Crassostrea virginica] | XP_022341228.1 /9e-81 | SO(SM000201);TSP1(SM000209) |
| **Unigene7419** | 4.1951 | FYGPEGAYGVFAGR | 1 | KEWLTVEMAEKAVESESGGFFSNLLSELFGSPLNLLLLGICLFLLYKILGGRRTVAPSSPKPQIPKMKKKDFTLEQLREFDGRGPDERILIAVNFKVFDVTRGKRFYGPEGAYGVFAGRDASRGLGTFSLNEDALKDEYDDLSDLSLEQMERVREWEMQFTEKYDYVGRLLKPGEQPRDYSDTEDEQSEEKSSEGKKKD | membrane-associated progesterone receptor component 1-like [Mizuhopecten yessoensis] | XP_021375937.1 /2e-84 | Cyt-b5(SM001117) |
| **Unigene13664** | 4.1951 | NVLNILGYGK | 1 | VTVELTSNHKGYFEFRLCPNDNPNKPVTQACLNRNVLNILGYGKRYIIDTKDYNMFMEFQLSLPPGMTCSQCVLQWKWRAAQNKGPDGKGGECFGCGPQEHFINCADVSIASGTRPLRLNRPGHTGVYISKTGPKTVVSHTPSVTTTAQTKPQPQVYDPSVMEPPSGVYSNFYPTPSSTANDEQFLTRSSTSNND | chitin binding protein [Pinctada martensii] | AIF72920.1 /2e-45 | Chitin_bind_3 (PF03067) |
| **Unigene2555** | 4.1951 | FPGQLNADLR;LAVNMVPFPR;LHFFMPGFAPLTSR;YLTVATIFR | 1 | SESCECLQGFQLCHSLGGGTGSGMGTLIISKIREEYPDRIMNTFSVVPSPKVSDTVVEPYNSTLSVHQLVENTDETFCIDNEALYDICFNTLKLKNPTYGDLNHLVSLTMSGVTTCLRFPGQLNADLRKLAVNMVPFPRLHFFMPGFAPLTSRACKDYRAVSVQELTQQMFDAKNMMAACDPRHGRYLTVATIFRGHISMKEVDEQMLNVQNKNSSYFVEWIP | beta-tubulin [Papilio machaon] | BAN92310.1 /6e-141 | Tubulin(SM000864);Tubulin_C(SM000865) |
| **Unigene57134** | 4.1951 | IPDWFLNR | 1 | KMSLVIPEKFQHILRVLNTNIDGRRKIAFAITAIKGVGRRYAHVVLRKADIDLNKRAGELTDEEVERVVTIMQNPRQYKIPDWFLNRQKDVKDGKYSQVLANGLDNKLREDLERLKKIRAHRGLRHFW | 40S ribosomal protein S18 [Oryzias latipes] | XP_004073779.1/7e-87 | Ribosomal_S13(PF00416) |
| **Unigene34064** | 4.1951 | AVLVDLEPGTMDSVR;FPGQLNADLR;LAVNMVPFPR;LHFFMPGFAPLTSR | 1 | RKKIKMREIVHLQAGQCGNQIGAKFWEVISDEHGIDPTGTYHGDSDLQLDRINVYYNEASGGKYVPRAVLVDLEPGTMDSVRSGPFGQIFRPDNFVFGQSGAGNNWAKGHYTEGAELVDSVLDVVRKEAESCDCLQGFQLTHSLGGGTGSGMGTLLISKIREEYPDRIMNTFSVVPSPKVSDTVVEPYNATLSVHQLVENTDETYCIDNEALYDICFRTLKLTTPSYGDLNHLVSATMSGVTTCLRFPGQLNADLRKLAVNMVPFPRLHFFMPGFAPLTSRGSQQYRSLTVPELTQQMFDA | tubulin, beta, 2 [Danio rerio] | NP_001032487.2/0.0 | Tubulin(SM000864);Tubulin_C (PF03953) |
| **Unigene51780** | 2.1035 | FFNYIAALR | 1 | MSHILHSVRVIGRFPSANCLKLVPSTLASSGDQTAQLHSVKIEGHPVRDYAPWPYKSRPYRWYHQIYEHTTKRMNENSKIVVVEGNIGTDKTSVAKKISEEFDMLYLPDVREEAYFINENGFDQRELNSRLGGDRFYDLKDLYSAKNPEDALGYGRTQLKMFELRFFNYIAALRHLLNTGQGVVIERSPWSDRCFADAMRKCGYFTKEGKEYYNMLYSKPTQVVWMPHLTIYIDASVSQCKELIAKRNNPLEKNSPLLTDKYLQAMEESYKERVLPHLSKFGELLQYDLDDIEKFDEIVAHDMQKVNLEPPVLMDEFKFDDWYEDKEWKWNFVRQKVADDAFFHRKYFLDNSLPFRAPEICVQDFNNYDLVVNEHPSNRIPKGYDPEKDNVLFKTRGPGPYVTRVQPTSTS | NADH dehydrogenase [ubiquinone] 1 alpha subcomplex subunit 10, mitochondrial-like [Limulus polyphemus] | XP_013783567.1 /9e-63 | dNK(PF01712) |
| **CL3196.Contig1** | 4.1951 | QAVSTSVTQFLSDQISR | 1 | MDFVKYLMVPAFLGSIFHNTVNGELTSSLKQAVSTSVTQFLSDQISRVKISPISIAGIILTVTGTFTANAKKVYVYIEIVKATNQVGYCRAIIGQMTIGFKPSQPSGSTEGPVNLSSSLIGELKKKLAEEICKNMKNTLNSNPLFPPP | — | — | Ser(10.8%) |
| **Unigene1514** | 4.1951 | VVEDITTVNDDIGAR | 1 | RINEEHLKLVENVSPLIMATPTTPTVGTPKKKDESFLDKIGTIGRKKKAKEASILEEEGKNAIESPTSPTIPDMGPEGYLLDEMEERSMIDPTSKDDPKVRELTKMLLDWINDVLAEDRIIVKDILEDMFDGQILQKLIEKLADIRIQVPEVTQSEQGQKQKLQIILSVINRLLQLQWNQVRWNVDKVHSKNLVAILHLLVTVARHYRAPIRMPDNVSVNLIVVTKRDGMLQTKRVVEDITTVNDDIGARFERDAFDTLFDHAPDKLNLVKKTLVTFVNKQLLKINFEVTDLEQQFHDGVYLILLMGLLEGYFVPLYDYHQTATVFDQKVHNVQFAFELMQDAGLPRPKARPEDVVNQDLKSTLRVLYNIFTKYKAQGS | beta-parvin-like isoform X1 [Crassostrea virginica] | XP_022294623.1 /0.0 | CH（PF00307）；CH（SM000033） |
| **CL955.Contig1** | 4.1951 | ALDSMQASLEAEAK;CIIPNEMK;CNMISGEVEELR;EEEFENTR;EIAFQADEDR;ELEGELDSEQR;ESYNLAER;GAENELFEANDR;GQLEISNVR;KGAENELFEANDR;KVQQELEDAEER;LAEKEEEFENTR;LEEAESQALK;NLYSTHPHFVR;QVEEAEEIAAINLAK;QVEEEQR;RCNMISGEVEELR;SSAFQTISAVHR;SSVSISR;TALEQAER;VGLSVIQR;VQQELEDAEER;VRELEGELDSEQR;YQQQVSEVQR | 1 | TASVLHMGEMKFKQRGEQAEPDGTAEAEKVSFLLGVNSNDFVKCLVKPKIKVGTEVVAQGRNKAQVMNSISAMAKSLYDRLFAWLVKRVNHSLDTKAKRNYYIGVLDIAGFEIFDFNTFEQLCINYTNERLQQFFNHHMFVLEQEEYKKEGIQWEFINFGMDLQACIDLIEKPMGILSILEEQCMFPKADDKSFKEMLFTNHMGKSPNFTKPGKAAKGKNGDFELHHYAGSVPYNIAGWLDKNKDPINETVVELLQGSKEHLVVTLFAPPEGAEATGGTKKKKKSSAFQTISAVHRESLNKLMKNLYSTHPHFVRCIIPNEMKQPGVIDAELVLNQLQCNGVLEGIRICRKGYPSRIIYAEFKQRYSILAPNAVPQGFVDGKVVTEKVLLALQLDPAEYKLGNTKVFFKAGVLGNLENMRDERLGAIVSMFQAHIRGYLIRKAYKKLQDQRVGLSVIQRNIRKWLLLRNWQWWKLFAKVKPLLNYAREEEEMQKKMEMMKKMEEDLAKTEKIKKELEIKNVELLEQKNDLFLQLQTQEDTVIDLEERVQQLVNQKCEFEAQMKEMEERLLDEEDAAAELENVKKKMEGENSELKRDIEDLETTLAKAEQDKTTKDNQIKTLQGEMAQQDEQIGKLNKEKKNMEELQKKTLEDLQKEEDKVNHLNKVKQKLEQTLDEMEDSLEREKKIRGDVDKAKRKVEQDLKATQELVEDLESNKRELEEANRKKDSEMSMLNSRIEDCEGVNAQQNRKIKDLMATIEELEEELEAERAARAKAEKQRAELARELDEISSQLEEQGGATQAQVDLNKKREQELVRLRREMEEMTLQNESQVSQIRKKAQDQANELADQIDGLNKLKSKLEKEKKDLKRELDDVQSQVQYSMKNKGVSDKVAKQMEVQISEMNSRVEESQRTIVDINSLKTKLQSEVADLNRQLEDAEHNIGSLTKDKTSLNHQLEESKRSLEDETRTRQKLQNEIRNLNADVDSIREAFEEEQESKSDLQRQLSRAKNEAQQWRSRFETEGTAKADELEEAKRKLAARLAEAEQNAEAANAKASGLEKAKNRLQGELDDLLVEIERSNVSSSTLEKKQRQFDRTIQEWTTKVKELQTEVDTAQAEARGYSAELFRSKAQYEECNSTIESLRRENKNLADEIRELTDQLSDGGRNAHEVEKAKRRLEMEKEELQAALEEAESALEQEEAKVMRGQLEISNVRSEIERRLAEKEEEFENTRRNHQRALDSMQASLEAEAKGKAEAMRIKKKLEQDINELEIALDASNRAKAELEKNIKRYQQQVSEVQRQVEEEQRQKEEVRESYNLAERRCNMISGEVEELRTALEQAERARKGAENELFEANDRVNELSAEVQSISSQKRKLDGDIQAMQSDLDEMNNEVRNADDRARRAQEDSARLADEIRNEQEHSQQIEKFRKSLEGQVKDLQVRLEEAESQALKGGKKMIAKLEQRVRELEGELDSEQRRHAETQKNMRKADRRLKEIAFQADEDRKNQESLNSMIDTLNAKLKTYKRQVEEAEEIAAINLAKYRKVQQELEDAEERADSAEGSLQKLRAKNRSSVSISRSSVTHTPATSPSVLNSSNLLSPRSMSRGPDSSFLSPRSASRGPGLYRRSVTPSYEDDDY | pedal retractor muscle myosin heavy chain [Mytilus galloprovincialis] | CAB64663.1 /0.0 | MYSc（SM000242）；IQ（SM000015）； Myosin_tail_1 （PF01576） |
| **Unigene44594** | 2.7302 | DPQIFVGR | 1 | MTEWVKTSANNIPNGSIRGGYDKNGHTLFIARALTDDGFYSAGKASLHYEDGAHIPYRGQEIIVYEYEILVLPSQADGFYDWKPTASANVPSNAVPSDINRDPQIFVGRFVHEGCLIPGKVDKKKMKCYIAHNGKEYPNDHYEVLVKVK | Chain A, Crystal structure of a DM9 domain containing protein from Crassostrea gigas | 5MH0_A /4e-32 | DM9/SM000696 |
| **Unigene3849** | 4.1951 | TFLGSVSDYVVQHAR | 1 | LHSLSKFVSHIILFFVKVHIKMAEGSSRIVMIAVDASKHSDEAFDWYLNNMARDSDDVRVVHCAEYNIDLGFGLNYDEKQIQQITQQVKEQQEKVDQLKSSFVDKLRGKGPGDESFRIKGEAFVITGKKPGEAIIAEAEKQSANLIIMGTRGLGKIRRTFLGSVSDYVVQHARCPVVVVRH | universal stress protein PHOS32-like [Crassostrea virginica] | XP_022298339.1 /2e-42 | Usp（PF00582） |
| **Unigene52081** | 2.1746 | VMVDGTSR | 1 | VREFTQGGKHYYRVMVDGTSRDVEIPGDQKLELTGYLYIGGLPTAMFRDTAVLSNIKSRYGFMGCMASVDLNGQSPDLFSTANNQASVMSECTASTTTGPVRWNITNAPER | Neurexin-1-alpha [Crassostrea gigas] | EKC36962.1 /1e-12 | Laminin_G_1 （PF00054） |
| **Unigene13412** | 4.1951 | LPLVIEFTQESAQK | 1 | MLPILLSACVIALTFGADIKEEEGVLVLTTANIEDALKENTNILVEFYAPWCGHCKALAPEYAKAAKALADEGSDIKLAKVDATVESSLAEKYEVRGYPTIKFFRDGKAIDYSGGRQSADIVNWLKKKTGPPCLALESVDDAKKMVEKDEVVVIGFFKDLKSADAQQYEKAAQGIDDIPFGITSNTDLFKEYEMESDGIALFKKFDEGRNNFEGSVTAEAVNAFVMANRLPLVIEFTQESAQKIFGGEIKNHILMFLEKTADGSDKLIDGYKKAAETFKGKVLFITLDTSDEDNARILEFFGLKKEDCPSARLITLGEDMTKYKPESNDLSEDAVRSFVQNFLDGKLKPHLMSEEIPSDWDAKPVKVLVGKNFQDVAMNKDKAVLVEFYAPWCGHCKQLAPIWDELGEKYKDSADIVIAKMDSTANEVEDVKVQSFPTIKYFPKGSSEVVDYNGERTLDGFVKFLESGGKDGAGEAEDEDDDDEEEDEDEPAAKDEL | procollagen-proline dioxygenase beta subunit [Mytilus galloprovincialis] | AFM30917.1/0.0 | Signal peptide(1-16)/Thioredoxin （PF00085）；Thioredoxin_6 (PF13848) |
| **Unigene53649** | 4.1951 | IILLAEGR | 1 | SEDMNGGGRNDSAGILAMKPNPGLKFEKKLSETERSTRDMKLVAKSGKKKKDVKFVLTPTKKRLLSRSISASSSTDSFDSTSYTGSSSDEDDVNPREKVQKNSKGSGDFCVRNIDHAAFGRREIEIAEQEMPGVIALRKRAEADKPLSGAKIIGCTHITAQTAVLIETLAALGASVRWAACNIYSTQNEVAAALAEAGYPIFAWKGETEEDFWWCIDKCINCEGWQPNMILDDGGDATHLMLKRYPAMFNMIKGIVEESVTGVHRLYQLSKGGKLTVPAMNVNDSVTKTKFDNLYSCRESVLDALKRTTDVMFGGKQILICGYGEVGKGCAAALKGLGCSVMVTEIDPICALQACMDGFRVVRLEEVIRSIDILITCTGNKNVVTRSHMDRLKTGCIICNMGHSNTEIDVGSLRTPDLTWEQVRSQVHHIIWPDGKRIILLAEGRLVNLSCSSVPSFVVSITAATQALALIELYNAPPGRYKQDVYLLPKKMDEYVASLHLPNFDAHLTELSDEQAKYLGLNKTGPFKPNYYRY | S-adenosylhomocysteine hydrolase-like protein 1 isoform X2 [Mizuhopecten yessoensis] | 0XP_021362456.1 /0.0 | AdoHcyase_NAD（SM000997） |
| **Unigene20162** | 4.1951 | GMTAMGAVR;HISDIR | 1 | ISHVGTEAQRLNYNGPTIGAKPTEKRAVKFSYEQLKQSCGLIGLQSGTNKFASQRGMTAMGAVRHISDIRADKFSKEAEGEINLQSGTNKFASQRGMTAMGAVRHICDIRADQYDPESNKEINLQSGTNKFDSQAGMRGFGAIRHISDVKVNELDREGTSVLRLDMGYVGGDSQKGMTSFGAQRHITNVKVNDLAEEFALQHGKPAPTPQPQAVEEVAQEEEEE | calponin-like protein-2 [Mytilus coruscus] | AKS48163.1 /2e-125 | Calponin（PF00402） |
| **Unigene23034** | 4.1951 | IIDVVYNASNNELVR | 1 | PNSNFLFSIIMGISRDKWHKRRKTGGRMTQMRKKRKFELGRPAANTKLGPQRIHTVRTMGGNKKYRALRLDQGNFAWGSEGIAKKTRIIDVVYNASNNELVRTKTLVKSAIVSVDATPFRQWYEAHYAKPLGRKKNVKLSEAEETVLNKLEKGSKRLKRKYEERQKTAKVDQALDDEFSAGRVLAKLASRPGKCGRADGYILEGKELEFYLRKIKTKKGK | ribosomal protein S8e [Mytilus trossulus] | ALX27203.1/5e-130 | Ribosomal_S8e（PF01201） |
| **CL1102.Contig1** | 4.1951 | ILIVGEFGSERLVQQVVNTILR | 1 | RILIVGEFGSERLVQQVVNTILRRTAAQKKDKESSVVNPYSDDQDWSAETASKIILTGHAHKSLHKEELYKSEKEKEVKKRITSEASKQQQKKTTEMSKTSVQKSSPKKVRELHVVCLILGILSIC | deubiquitinating protein VCIP135-like [Crassostrea virginica] | XP_022335712.1 /3e-26 | Lys(14.3%)；Ser(11.9%) |
| **Unigene23041** | 2.2753 | WIVNLIR | 1 | HIGSMAEWDLTSRMGQYLDRHLVFPLLEFLSVKELYDEKEMLKGKLDLLSNTNMVDFAMDVHKNLYPDTDVPQNLKDKRSNVVSQLKYLQSETELITKIFEDPEVIRQVQSSRDGRQLFEFLSKEFGFQSKMIDTLYNFAKFQYECGNYSGAAEYLYFVRVLLLSTDKNFLDALWGKLASEILMQNWETALDDLTKLKEIIDGNTFGSALQNLQQRTWLIHWSLFVFFNHPKGRDLIIDMFLYQPLYLNAIQTTCPHILRYLTTAVITNKSRRRTVLKDLVKVIQQESYTYKDPITEFVECLYVNFDFDGAQQKLRECEEVLVNDFFLVACLEDFIENARLLIFETFCRIHECISIRMLAEKLNMSEEEAERWIVNLIRNARLDAKIDSKLGHVVMGTQAISPYQQVIEKTKTLAFKSQMLAMNIEKKLGLKTDSTNQWGPE | eukaryotic translation initiation factor 3 subunit E-like [Crassostrea virginica] | XP_022313018.1 /0.0 | eIF3_N（SM001186）；PINT （SM000088） |
| **Unigene8424** | 4.1951 | YMYTANSWYDISGR | 1 | MVLVLLLCLFPTTIASAVNNDPCGVSGNWTNQFGSLMVVSCQTGPVVPSEGSVTGRYMYTANSWYDISGRYTMINGDCILGFSIAIGNNYGNSNITSWTGIHYASEDNIHTQWFLSK | Chain A, Streptavidin Mutant With Osteopontin Hexapeptide Insertion Including Rgd | 1MOY_A/5e-05 | Signal peptide(1-17)/Avidin（PF01382） |
| **Unigene4671** | 4.1951 | GYSPAHNLR | 1 | WAVSSLDRRLTQNKMKVALVVIAVCLGLAFAEKGQRGYSPAHNLRTYGTVEGYGLWLECGWYGDCSGRRGGGGGGHGGYGGYGGYGGYGGYGGYGGYGGYGGYGGYGGYGGYGGYGGYGGYGGYGGYGG | — | — | Gly(39.5%)；Tyr(16.3% ) |
| **Unigene12198** | 2.6269 | HAFGDQYR | 1 | SLLSKCVEMASPVAALARVVCRASGTAQNLVAPAVITNNPKRNYAGPAKRIKVANPVVELDGDEMTRIIWEKIKDTLILPYLDVDLKYYDLGLPYRDQTDDQVTIDAAMAIKKYNVGVKCATITPDEERVEEFKLKKMWLSPNGTIRNILNGTVFREPILCEKIPRLVPGWTRPIVIGRHAFGDQYRATDFVAQGNGKFEMVWTPEGGAEQRMEVFNFTNGGGCVMGMYNTDESITGFAHACFQYAINKKWPLYMSTKNTILKRYDGRFKDIFQEIYDKQYKGEFEKLGIWYEHRLIDDMVAQALKSDGGFVWACKNYDGDVQSDVVAQGYGSLGLMTSVLVAPDGKTIESEAAHGTVTRHYREHQKGNPTSTNPVASIYAWTRGLEHRGKLDGNPELQRFANTLEKACVATVDSGKMTKDLAGCIYGLKNIKPEQYLYTMDFLAAISEELNRQLK | isocitrate dehydrogenase [Mytilus trossulus] | AFI56373.1 /0.0 | Iso_dh （SM001329） |
| **CL919.Contig2** | 2.1035 | QNLDLR | 1 | MDAFENVKEFSPPRQDEDELGKDDEDEEETDDEEDDDEDGDESGDEEEGDDDELGEDEEWVTDDDDAEDSQDVTLVTSDDDGEGGESCPICLNKFRDQDIGTPESCDHCFCLECIQEWANNVNTCPVDRQVFHLILAKHAYGDRVFKKIPVEDRKIEDEEDEDPTYCEICGRCDREDRLLLCDGCDQGYHCECLTPQLQDIPVEEWFCPDCAQTETEAVTVDDDEEIAEIISENAESLPSLRPQRRLIARTRASERVREQIIELRLRRADREARVTRRRTILLSDDDEDNSQPQAEISTQPSTSGALRKTPTKRKTPKAKRKTRKRKKTTKGAKKTPGKGKGKKKRRRKRKKVTKTSKRKAQIKKLVGKMVSREVRREVVPPASVKSRIAKNLGLSKPPTGTTIPLQKVPTDKGVDTARSNIGITQLSVMGDKNELIGFCDEPGDLIPSASKPSKKSKYSTLALFSHRPVGKPVIKIDQSPAASTGAAGFDLLGSIMDNQEMLGKDSSDVTIHRDGSLSKKAECKPVRKPSGQLLTPQKSQAELYDDLPPLEVSDLNSDLPSPEKETQTAGQNQISDESMKEIKYLDRQVDTTVSPDKSLDLSAVRNDFVETSKDTSFQIKKSNENSIIDNAGESDSNDRSDKNSVNCDIQNSESDLVTEEGSSKKKSLSTNKESGDQNSNSEKSADKSTDSLLESNNKTAKKADKNISNSSDKIISKSNSTTKKIEMASQLDDISDESNGFHDYLEEKELEEKLQQAQEKLRKLENKGKKDKKKELLKEKKKEVLKEKRPEKTNTGKEKTNEIESRKEKSDKVKKKLKESDKSEKKVVKKTKEAGNEKSKEKKCRGSKDEDDIMITTPPPQEIEVIEIPDDDDESEYVQNLDKKIIKSYSGDGSRKTKSRQNLDLRSVVKKEKTDKDEKENRKRRESERDHESDRNSKKKKRYRSRSKSRDRRHRSRSYERSHRSTSRSRDRYKHRRSRSRSRDRSRRSRSRERWSRDRSRDWSRDRSRGRDRSRDRERSRERNRSRDRDRSRSKDRSRKRRHRDKSKDRSTSREKNRMRSRDTSRERRRDSSPQVSLNISSLDSGRLDTGRLIDLFDDKWKKREDLKKSKKKKKDKGHESPIKSVQSPLKRPLEEDNDAEGNITKKSKTPVKEKTPEPVDSVQPIPKIIHVFPEKEKKEIDMFADDNSDMDTDIPEPAQDTVILINTVDNISNSQRPNLVPLPIVPGDDKKDDKVPEMISQVESNEVIPVINSANTDNITVASPEYDPAFPTDDMEESPLQDEKSPPGTPVEYTNPPPQTNLPLIAGEPPRLITVTQPLDEIFPDRVSPLNQLPPQLGKGLLGEGGPLILQRGEAPIIQPQDVQVGIRFLQGIRPGMSPQRAMLMNPVLMNRPPQMQPRFTRIPPGHLPQFGLQVTTQEGVALPVQGQFPPGMVAPGRLPPPQPGLINGSFEGIQPDTSQPPPPFQLLAGAGNHRLPGDPRLQLPPHVLAHGPPAAGAPLHLQPGPGMEQHVSIPSPLVSVSGPPMEGPHLTLQGPPQISLSGPLPRLALPNHPLSVPSSQIRIPSPDAPLGNPLTKLPARMPRLQIPASAALVSVEPAGILERPYTPPTPTNDDTPFMNQAVEASALAKLSRSQSPQTMPNISLLGGKPLMSINLKASKTLQNVNPLLSGKSGHLDEYPSPDENEGDDIIKDSGKTSGNVPQSQSTQLTQLTKLLNAQAQLAQLVSKGKTAKPSSKSGHHKSNDHKFKVPLPPKGKTSNGKVKVGESVDVIDMDVASPLDESSIEIPDSPDDFDKAVFGSKEKENKKRHDKHDRKSHKHKDRKAKASSSNVKLGGSNKKPVMSVNDENLKDVIRELEMDEVPSSAVELTNKEKFLKKLHLQERVVDEVKHALKPFYSQRRINKDQYKEIMRKAVPKVCHSKSGDINPQKIATLVEAYVSKYTKKQKKGEES | protein SCAF11-like [Mizuhopecten yessoensis] | XP_021339730.1 /6e-108 | RING（SM000184）； |
| **Unigene36836** | 4.1951 | TLTPSFAILLNK | 1 | MLLEIQRTVILFVLLLSYICAQEVAKVGGKIKVESLDPFAKPGVDPFASIRSAGGSGAQKQGATILTKKETVRTVTTNNKPVVDGSDVRSSQAGTSKNTQTTNEKPYVETTTIERKTRVIGSGSGTLRKSSNTNAQNIASKTETTKTQRTVNVVNRGTGNTGTQSRSGSVQTTGSRSQSSTTRTVQTTNTGSRTGSGERNTDLPRQTGGNRGSGQNIISVSSQTDRQLGQIGSRGSQNTIETVRRNDNVGPFGSSGGSNTGIYTNDGTMKIDTNRGNALPEMGGEMGATDKPILYFRKLYHDQSICLNCIGSGSDCYTEHPDDCRKYIRCTTSSLGGRMEALEMDCAFGTFWSNEANTCDTASEVFCNKDPCMDTSLKTYHSGLNCRSYWRCVNGRSNAECCDNGQEFVDGHCIPSDRCTIDDGECPFKSQFLDTIPEKKPTCNLEPVPEKGEKAFRMNNHWALMYCADGTKFDMKTCGCIVSIFNKVRCVPTVDIGFDEDRVIDRTRGYIKSENVTVTGGKGSAYALFQGESEINVPYFKNNENIKSHLVVSVRFFKNDDKGPGSQVLASNCKTSNKRTLTPSFAILLNKKEKQIVFFASTKKNDKYGATQHITLPFTPGKWTTATLKFLDETLQGTVETLEADGTPKIQKEEKPLKGTLNTGIMPLMIGTCNMNDGFFGYIHKAKAYMCKPDQP | Follistatin-related protein 4 [Crassostrea gigas] | EKC18454.1 /9e-43 | Signal peptide(1-21)/ChtBD2 （SM000494） |
| **CL867.Contig1** | 2.0605 | SVESIEELTQR | 1 | LAFFGCLLLLVCEGFQREIGGKRVINELQDNALYPKPNHETEDFLNKDEEFKGIVPFVRRKRGIGSVLVMGISLLLRTTNLGLTIAGAASEGCAFFRSICTNRDRIEVLDSEIKNMKKEFDVLYRDNGRSYVDLKQSYVNNGYISYYTNETIRSVESIEELTQRLVEKISLKDFPTFAEIAKSIEHNVSSEDYLKEVVGQLEYSLEYLTARIQQTKTNLLVGIAVQYLAEALLNRFMKAYSTAYGQIAEDIFFQSQIQNMFNPDGVGDQGKITFLKKVKLIPKVFKQMGKNVGKYFKNGWHSLKKWKRINSWKGFKGKLRHSLRKPLAKLKAVKNFMTNSAARRHSIRNFKLGWSQRIGVTLGIVADAINIALGIHEWSKVRDEMARAKDNYEEYKANLTREIADTKVEADRLMKGWENSSNTFSDVTKSIRSLFTDLANNSDFNGVLGLGGLPVNFVSDYLPENFENVHKGNLLSKQAIVIDFLKKINHNFTKIREDLNARLIMYDHVEQGVKKDQSINEMLQTLKNIYKYKTGEEKFFGSSLSDRDIICAIAVSFPLKTNYDFYNLNSLRPTCHVSDAQFASIKAKAMEKRKKVLMGNVVKQEVSNDPTASLEHLLNLVKNAYSFSNDNSLKQYGKTITEKDVVCTVASVFPALQSFDFLSLVPLRPDCAAVSDSDFANMKQAIANKKLIVEQIKGAVNACISWNMCPCIPVIRTKLQGDGITASESEVKEAIKSARPELTQYCGTTGCSCVNL | Isopenicillin N synthase [Macleaya cordata] | OVA15883.1 /1.0 | Signal peptide(1-21)/Internal repeat |
| **CL1791.Contig2** | 4.1951 | LMIGMFDR | 1 | MAYYGQQQPAGYYGQQPGPPPPQDQNQQFLWGVFQRVDKDKSGQISGNELGSALSNGTWTPFNPETVRLMIGMFDRDHSGTINFQEFQQLWKFVTDWQNCFRGYDRDNSGSIDKNELKTALTSFGYRLSDRFYEILVVKFDRQGRGTIAFDDFVQCCVVMQTLTSAFRAYDTDQDGWIQIGYEQFLTLVFNLKS | programmed cell death protein 6-like isoform X1 [Mizuhopecten yessoensis] | XP_021343811.1/2e-107 | EFh（SM000054） |
| **Unigene24855** | 2.6269 | GYSFITFVDR | 1 | MADVNGLDHKDQNMAESDVKPEEYVKLVEYGINARVAEELCNVYKTEKLTHAELDERALDALKEFNADDAIAVLKQFLDSPLEHVTNKSAYLCGMMKTYRQNKKQGSTATVAKGPDEAKLKEILDRTGYSLDVTTGQRKYGGPPPGMDESSQPGAGHEVFCGKIPKDVFEDEIIPLFEKCGQIWDLRLMMDPMTGFNRGYSFITFVDREGANEAVKQLDNYEIKSGKRLKVNISVANQRLFVGNIPKSKTKEEIMEEFSKKTEGLQDVIIYRSADKENQKNRGFAFLEYDSHKSASTAKRKLSSGRSKVWNCDVIVDWADPIEEPDSGTMSKVKVLYIRNLSSDVTEDVLKEKFSEYGKIERVKKIKDYGFVHYEERDDATKALEAMNGQKLGKLEIEVSLAKPPTENKKKEQRKREQERRQMMGYDDFGYGPPMPPGRMMPPGRGMRRGMPPPPRFDYYDDFFGYEDYNDYYGGGYAPQMPRVRGGRGAPPFASANARTRRGPTPWWT | RNA-binding protein [Pinctada fucata] | ABP04054.1 /0.0 | RRM（SM000360） |
| **Unigene2635** | 4.1951 | FSPDGQYIAIGGGDR | 1 | MTEVTPRCVFASLPRTTRGVPIVLGGDPKKKNFLYTNNNSVFIRDVANPSECDVYTQHQCQVVSAQYSPSGFYIASGDVSGKVRIWDTVNREHILKNEFQPLGGTIKDIAWSGDNQRMAVCGEGREKFAHVFAADTGTSVGDLGGSSKSCNSVSFRAERPFKIVIGSEDFKSAFYQGPPFKFVKTMNEHTNFVNSARYAPDGSVFITGGAEGKAYVYEGKTGDLLGELGSPAHKGGIYAISFSPNSSEVLTVSGDKTAKIWNVANRELVTTFEVGKTVDEMLVGCLWQGDFIMGVALSGYIYYFNRDDSSSPPRVIVGHNKPITAMALSEDRSTIYTADQIARTVCWNAASGENSVFSGKGHSNQVQDMAVTDGKLVSVGMDDTIRFTDLSNNQFGDNSMKLDSMPRGIAARSSVVAVACINHVVVFKNGSKLLSQGVKFEPLSVDVSPNGSLIAVGGDQDRKVHIFDISSGGLTEVNTITCSGSPTCVRFSPDGQYIAIGGGDRYLRLYSMSDLNTTIIEYMGHTAKVTCVAWSPDSSRLATGGLDSQVVVWDPKDPMKPKVFRRAHAASYVNKIAWLNDSVVVSAGQDSNIKQWDA | WD repeat-containing protein 1-B-like isoform X1 [Crassostrea virginica] | XP_022289109.1 /0.0 | WD40（SM000320） |
| **CL1815.Contig1** | 4.1951 | DIVQFVPFR | 1 | LTSFKLQRTPSFLEYLYGGMQINFTVGIDFTASNGDPNSSNSLHYINPYQPNEYQQAIQAVGNVCQDYDTDKMFPALGFGARIPPNNEVSMEFALNFNATNPYCAGVQGILEAYTNCIRQIRLYGPTNVAPIIYHVARFADAAQKEEATKGAHSYFTLLLLTDGVITDMNDTRQAIVNASGLPMSLIIIGVGDADFADMEFLDGDGGVLKAPNGQPAQRDIVQFVPFRDFKRVSAAQLAKHVLAEVPQQVVKYYTMRQIMPNPPRQAQQ | copine-3-like [Crassostrea virginica] | XP_022342060.1 /9e-143 | VWA（SM000327） |
| **Unigene49599** | 2.7302 | SPIAEAVFLR | 1 | MSSKKHSVLFICLGNICRSPIAEAVFLRLLKEKNQLQDWMVDSAAMGDWHVGKGPNERTVTTLHKFGIKDYVHKARMIQPADFEKFDYIFGMDHDNMSDLDSIKPKNCHAKLHMLGEFDPQKELIIVDPYYGHDMKDFDKVYEQCCRCCEGFFNSVTGS | acid phosphatase isoenzyme As [human, erythrocytes, Peptide, 157 aa] | AAB22516.1 /8e-53 | LMWPc（SM000226） |
| **CL1924.Contig1** | 2.7302 | AIIDVLAFR | 1 | KILLTYEWTFRNFNLAFTFSDEKKKMSYPYGGGNPGYGPPGGGYPQQGGYPQGPPQPGYPQQQPGYPGQVAMPTVESASAAAYAPGPGGYPGGPGQGLGFDGVGGPPQGSGYGPPQPAPYGAPPAPAPAMPSSYGPPQPQYQSSTPAYGSAPGYNPAPQGAPPPAQPGYGAPPFQPGHNSMPNQSYGASAPPPAGGSYGAPPQPQGGYGAPTSQPGGYGQPQQQQQGYGQPTSQNYGQPPSSQQYGSQQKSQPAANAYVQRTEGTLRPASNFNSENDANILRKAMKGFGTDEKAIIDVLAFRSGEQRQQIRTMFKTMFGKDLIKELKSELGGKFEDVVIALMMPWDEYDAYELKRAMKGVGTDEDAMIEILCSRSNKQIQEINATYKRLYHKKLEDDIISDTSGHFKRLMVSMANGGRMESQDVDSNKAQQDAQRLLQAGEKRLGTDESTFNAILASQSYIQLRAVFDAYQKIAGRDIEQSIKSEMSGNLEIGMLAIARIVKNRPAYFAQKLYHSMKGLGTDDKTLIRVIVTRAEVDMVQIKQEFQKLYGKSLDQFIREDTSGDYRKVLVALVSQGGY | annexin A7-like isoform X1 [Crassostrea virginica] | XP_022317069.1/0.0 | ANX（SM000335） |
| **Unigene7351** | 2.1035 | AAEISMGTEISEEDIMNVTYLCDQVIEITDYR | 1 | EAVYLVACKTVYKKKMLVLFETAAGYAVFKLQNEKKLRETENLYKEFESPESASKLLKLKHFKKFNDTTEALAAATAAVEGKMSKSLKKLMKKLVVQDAQEELAVADAKLGNVIKEKFDLNCVFNSNIHELMRGIRSQMNSLITGLPEKEMSAMALGLAHSLSRYKLKFSPDKVDTMIVQAISLLDDLDKELNNYVMRCREWYGWHFPELGKIVTDNLAFAKIVKMMGSRTNAATTDFSDVLPEELEAELKKAAEISMGTEISEEDIMNVTYLCDQVIEITDYRAQLYDYIKNRMLAVAPNLTILVGELVGARLIAHAGSLLNLAKHPSSTVQILGAEKALFRALKTKHDTPKYGLIYHASMVGQSTPKIKGKVSRMLAAKCALAIRVDALGEETNTELGLEHRAKLEYRMKRLEDKARGISGSGKAKAKWEKYENKSEVKTYDIGADSTLPGVPKKRKFDDADDEDVKPKVAKIEEIDETPEKKKKKKKKIKEEVNGDIDDMETSVNTTQEDVGSEKKKKKKKKVPFDLDAAMGETEVAADGAPPAAPEQSDAPKEVEESKPDKETVDDDMSFDFTKKKKKKKKAFDLEELGDALPEPTTTDSPQPPEDQDGGATEEAPADENVDLGLPTKKKKKKKKVDFDQIDVDLPVEDADEKDDKKEDRLSSSNTSGKSWLDSDRDYTYDELLTRVFDIIREKNPDMVTGEKKKFVMRPPQVLKVGTRKSSFANFADICRLLHRQPKHVLAFLLAELGTSGSVDGNNQLIIKGKYNQKQIENVLRRYIKEYVTCHTCRSPDTLLQKDTRLFFLQCETCGSRCSVASIKSGFQAVTGKRAAIRAKTT | nucleolar protein 58 [Lingula anatina] | XP_013401006.1 /0.0 | NOP5NT （PF08156）；NOSIC （SM000931）；eIF2B_5 （SM000653） |
| **Unigene9089** | 2.3093 | QDQIAIAR | 1 | SLLHAVVASEDCSLIVTLPAQFDIILILRDFITTGTMAAENGHNNAPDSWDQDDTDDSTQSGQSLSKLNANAPAFIPGKNPYAAEFVPTFGNTNNQAQSSSVQEDTSSPNNEVSADWDGDVDEEKKEEVKEQENDEGGGNEIVEDDDADEDEDEDEEEEASSKPQIKTQTSIPGEKRDHVNVVFIGHVDAGKSTIGGHLMYLTGMVDKRTLEKYEREAKEKNRETWYLSWALDTNLEEREKGKTVEVGRAYFETDKKHFTILDAPGHKCFVPNMIGGASQADLAVLVISARRGEFETGFEKGGQTREHAMLVKTAGVKHLIVVINKMDDPTVLWSEERYTECKDKLIPYLKKVGFNPKSDIYFMPVSGMTGAFLKEIPDESVCPWYRGPALIEYLDKLNLSSSSRYADKPVRMPIVDRYKDMGTILLGKLETGTVTKNQTLTIMPNKQNVKVMQMWSDEIEMDSAGPGENLKIKVSGVEEEDVSGGFVLCSPDNLCHTGKLFDAQIVILEHKSIICAGYSAVIHIHTCAEEVTIKALLCIIDKKTGEKTQLKPRFVRQDQIAIARIEVNGGLICMESFKDFPQMGRFTLRDEGKTIGIGKVLKVLD | eukaryotic peptide chain release factor GTP-binding subunit ERF3A-like [Crassostrea virginica] | XP_022317219.1/0.0 | GTP_EFTU (PF00009)； GTP_EFTU_D3 （PF03143） |
| **Unigene885** | 2.1035 | ILIPTPFSPLQ | 1 | VKTSIAMANAALKQLFSRTALIAGCHNRLIVAKPIRCLSSSQKLFAPDEEESSEKRGFLKNLLSVRTLKPSKESHSSQLSSKETVYEIQFHAVKPEFMEDYLNQFSKFQQLIQEKQTGADLKASFTVEVGDQDEAIHIWEYKGGYPVLNNATEIYRTDRDFIDFRISRNKMLRSRKNQILLGFSFWPKLEPREGRNIYEMRSYTLKAGTMIEWANCWARGIQHRGQGEDPVFGLFSQIGDLYTVHHIWGYKDLQTRKETREAAWSRPGWDECVAYTVPLIRHMQSRILIPTPFSPLQ | protein NipSnap-like isoform X2 [Mizuhopecten yessoensis] | XP_021367300.1 /3e-142 | NIPSNAP（PF07978） |
| **Unigene27228** | 2.5405 | VLGLVLLR | 1 | SKQPEREEKRVLGLVLLRGENLVSMTVEGPPPKDTGIARVPLAGVAGGPGVGRAAGRGVPAGAPMPQAPAGLAGPVRGVGGPSQQVMTPQGRGTVAAAAAGASIAGAPTQYPPGRGAPPPMGRGAPPPGMM | small nuclear ribonucleoprotein-associated proteins B and B' [Maylandia zebra] | XP_004558997.1 /2e-76 | Gly(19.8%)；Pro(16.8%)；Ala(15.3%) |
| **Unigene4279** | 4.1951 | TQTTGTNTAQTTGGTR | 1 | MKLFILLCTTCCLATISAQQQRTQTTGTNTAQTTGGTRGQQFGGGAGGGNGGFGGGNPFGGPFGPPGGMMGPGPTGFGFPPRFGGGPPSAFGGFGGGPGGPPTMQFGGPPAFAGVQGFGTPAGAGNLGNFGGAGTGGGNSLATSQFGTNAGTQGGAGGTPATQFGGTQQGGFPGAGGFGSQQGGFPGAGGFGGAQQGGFGGAQGGFGGAQQRGFGGAQQGGFGGPPPFMGFGGSPQFGGFPGAFPGVNGMFPPTGGFGGAGGGFGQFGGAGQNGGAGQPAANNQFGGAGAGGMAGFGFGSGMNLGTLPPTGANTGAAAGMTSTLFTGSNSFTPSSTGGVTGATQTQTANGAAGGTAAGGFGGSGAFTGMPGMTGMPGLGGMQQQGASSQFDMSSMMVPPMGGAGGMQLGGMQFAGMPQLGGAAGGAGMFSGMSGTQPSTGSTTGGQFQGAGANNGMSSQQGQPITPFTNTFDNFGSGTSAGTTGSGNTQNTGSLPVMPNPSMFQTGFGGQQG | — | — | Signal peptide(1-18)/Gly（33.0%) |
| **Unigene33174** | 4.1951 | YLFLGDYVDR | 1 | MASTDPKLSTKERIVKGVPFPPSYRLTDSEVFDSKDKPRPDVLKQHFILEGRLQEEVALKIINRGASLLRQEKTMIDIEAPVTVCGDIHGQFYDLMKLFEVGGPPATTRYLFLGDYVDRGYFSIECVLY | calcineurin A [Haliotis discus discus] | ABO26624.1 /3e-74 | PP2Ac（SM000156） |
| **CL2275.Contig1** | 4.1951 | ILVATNLFGR | 1 | STAGRSSYFLVAGKDHELKMADADAELLDYEEEDQETAGDGAGDAATKKDVKGTYVSIHSSGFRDFLLKPELLRAIVDCGFEHPSEVQHECIPQAILSMDVLCQAKSGMGKTAVFVLATLQQLEPVDGQVSVLVLAHTRELAFQISKEYERFSKYMNTTKIAVFFGGMNIKKDEEVLKKNCPHIVVGTPGRILALLNSKVLNLKNVKHFILDECDKMLAELDMRTDVQSIFRATPHEKQVMMFSATLSKDIRAVCKRFMQDPMEVYVDDDSKLTLHGLQQHYVKLKDNEKNRKLFELLDVLEFNQVIIFVKSVQRCMALAQLLVEQNFPAIAIHRAMTQEERLSRYQQFKDFQKRILVATNLFGRGMDIERVNIVFNYDMPEDSDTYLHRVARAGRFGTKGLAITFVSDETDVKVLNDVQERFEVNITELPDEIDISSYIEGR | spliceosome RNA helicase DDX39B [Crassostrea virginica] | XP_022325126.1/0.0 | DEXDc（SM000487）；HELICc（SM000490） |
| **Unigene27075** | 4.1951 | IDSTVVEGIR | 1 | MKRMMKYEFLPICIIYITFLICVHCWHHRRCGQYINEKYRTINGHCNNLNHPEWGQTGTPQTRFKDKRGRPMVEFDDDCNVPRGYHQLPNPRIISNQLSNAHGHDIFDYKRTAQHMAWGQFLSHDIVLTPMVSADIDCCNRKDKNSSNIDVCFPMSAPAHDSLPHGCLNFVRSAKAPDNRHRGYRENINKITAFIDASHVYGSDSETESKVRVNKHYLLKTTACNLLPTKEKGCFKTNKCPFGGDPRAQEVPNLTLNHHCYLREHNYISTELHKINPHWSNEKVFQETRRIIIAQMQHITYNHFLPLTLDWHTMRRYGLYSKKRGYENVYDDTVNPSVRNVFGAAAFRYGHSQIVPEQSELESDFKTKKVHLLEDQFFDPSLYQDNCGKKNEGLSRFLATSKACRIDSTVVEGIRSKLFFKNKPPGADLYATNINRGRDQGIPGFNKWRRFCGLKPFRRFRQFGYPAERKLRKLYRSIEDVDLFVGGLLECGNRGSVGPTFSCLIGKQFASLKQGDRYWYETNDYHIAFTEDQLNSIKSNTLLSKIWCRNFGLKKIQKDIFTTPSNRNRIVNCDDLPDIDLSLWKETESKYHYYMNKFWTK | peroxidase-like [Mizuhopecten yessoensis] | XP_021358062.1 /2e-130 | An_peroxidase （PF03098） |
| **CL1994.Contig2** | 4.1951 | IIVRPLQLNDLAESLK | 1 | MHVLLACTIYALLQGVNGAIDWAVAISTASAAAGAVTSGASMLQSLAGAPGYNVVCTVEIENWTKYPLVYPEASINKGIIKSPPVPVQPGKRESFIAHKTGHTATGTFGTASWLISSSNRRAVVMWSCPYNFDFHTNWLAVGLTSNGTTSHNKDWTNQMYYERNSPVLKYKLGQYYHHTKTISIKDEQFEITGIMGTSHKAKARIIVRPLQLNDLAESLKKNVIRKRRSADDQFENAQ | conotoxin [Conus andremenezi] | ATF27386.1 /4e-51 | Signal peptide(1-18)/Anemone_cytotox （PF06369） |
| **CL818.Contig4** | 4.1951 | QLDNVGYR | 1 | MSAPQVNGPSSPTGKYIDNIDPDDPEYQRQMRRPAEIKEDVKQMQDRSRVSLVLNSEAFRKELEEIIKEQFSEGNCPTNLLALQQITELLNPNNKTQSSGGVWRGVPSVIPINDIKGTETAKYAKHEKQLRCKVASLYRIIDMNGWTCGIYNHISARINQEQEHFLLNPFGVLYSEVTASSLVKIDMQGEVIDPGSTTLGINKAGFTLHSAIHQARPDIKCIIHLHTPEAIAVSTMKCGFLPLSQEALIVGEVSYHDYNGILVDEAERDQLQRSLGPNNKVMFLRNHGVVACGSSIEEAYHYAVNVMSACTTQTKAVPAGIDNLILVSEEIKKLTFQVGSQGGGGVDTGGRKWKTGELEFEAVMRQLDNVGYRTGYLYRNPHIKQEVKKEKNNSDVEIPPTSSSFTYVFDGDIEHSKYISPLKLAKDRQKQNYKAGWLVNSPNTYTRQEIEETGTPNPKKYSKWIAEADGSPNRKCTPIRLENPNQFAPQGENPKEYREKQKAIRKDYYEERISAGPQSKILEGITWEEAKQLQDGNLSMVGDNVVIYGAASKGIIQRDQQHNVQVYKTQYAANPFDSINEEEIEKYKIEVEQRGKGDAEPTEDLSPGPDGKLISTEERMQIIQQQQVETTPEHKPEVEKPKPEEKAPAVRRTSSNREPPRSPELIQELKEKNFERSKSERFGRDRVLNGDEKPSSPAKSDTLKSTDSASGGDTLDDRSSKEGSPVKELPSPTKDKKKKKKFRMPSFSKTKKNKDSKESTL | Protein hu-li tai shao [Crassostrea gigas] | EKC20098.1 /0.0 | Aldolase_II（SM001007） |
| **Unigene44900** | 4.1951 | AVGQDLGTSGSCLR | 1 | GVPGLNGRPGQVGPQGDTGLPGFPGLAGSRGEDGLDGLPGINGEPGQPGDSGYPGPAGPPGDDSPYIQGARGDPGIDGIDGRPGIKGHRGDDGLPGLDGLPGMKGNPGFEGFGLKGEPGDNGITGLPGSAGPKGYPGRPGIFGFNGAKGEPGLPGVNGEPGPDGQPGLNGLPGDAGLDGFPGSKGESGDYGYNGQPGLPGTPGENGFRGSKGEPGLNGLPGMEGDVGEQGKTMNPNGYTSGPKGVPGDFGEKGLNGIDGNRGFSGLEGIRGEPGLPGLPGEIGIPGPKGVRGDAGRDGYPGLPGLDGLPGLAGLPGENGIAGRSGIPSGVYFARHSQTTLVPECPTGTSLMWEGYSLSFIMGNGRAVGQDLGTSGSCLRRFSTMPFMFCNINNVCNVANRNDYSYWLSTLEPMTPMMNPISGPPLQDYISRCSVCEATGEVIAVHSQTVRLPDCPVGFKSLWIGYSFLMNTGAGGRGSGQNMQSPGSCLEDFRAAPFIECHSRGTCNYYATSLSFWMATIESFNQFRRPIGETLKAGNLRTRVGRCQVCMRY | Collagen alpha-1(IV) chain [Crassostrea gigas] | EKC43052.1 /4e-172 | Collagen（PF01391）；C4（SM000111） |
| **CL4349.Contig1** | 4.1951 | SQHQDLGWAGSCLQR | 1 | GMKGEKGGLGNSGPPGPRGQDGLPGMKGNPGLDGLPGTSGPKGESGFPGNNGFPGAKGETGLPGLNGQPGIDGVQGEPGTPGLPGRDGDPGLPGLKGDPGQPGFEGLPGAKGNPGLNGSPGLPGEKGNPGFPGGQGLSGLPGPKGERGFPGSSGPKGEAGRTSEGAKGEPGNPGRNGLNGLPGMKGDVGLPGRDGFNGVKGEAGEPGSGIRGQKGERGLDGLPGSAGLRGLPGNDGLPGANGGKGEPGERGLDGLPGQQGRPGFPGAKGDSGLPGGFGTKGSKGERGLDGFPGSPGPKGSSGLPGLQGPPGDSGLNGLKGDRGLPGLPAVLDITNLPPGPKGDKGSPGIPGNSGRDGLPGERGPSGPPGFPGAKGDNGLNGLPGMKGEVGPTGLNGIRGQDGLPGTRGQPGEPGQPGPAGFTPPSGFLMVRHSQDKVIPQCPDRMVKMWEGYSLLYIEGNERSQHQDLGWAGSCLQRFSTMPFLFCNTNNVCNYANRNDKSYWLATNAPLPMMPVTDSTIARYISRCVVCDAPSNVIAVHSQTLQIPECPIGWSGLWIGYSFAMHTGAGPSGGGQSLSSPGSCLEDFRATPFIECNGDGGNCHFYSNKYSFWLTTIDRNSQFSNPIPQTLKAGNLRDRVSRCQVCTKNV | collagen alpha-2(IV) chain-like [Mizuhopecten yessoensis] | XP_021346726.1 /0.0 | Gly(24.5%)；Pro(12.6%) |
| **Unigene6711** | 4.1951 | FEFSYFGTR | 1 | TDGFLAHNGRNNGMTYNIINYKLLTNKRLCIRFEFSYFGTRTPHHSCHQALLTTTATSKITKPSTVLTTTLRPRTTSSASDTSSHSTTKTSTTADTTTEKLITPTKSTTQRLTTEHQTWPTVSATTATTSVRSYTATSAIFTTTMQTTQNTQRTTAQQRRTTTTLPPTTTTLPTRPPMPDPSR | — | — | Thr(30.6%) |
| **Unigene1328** | 2.2079 | WVLISPYGTR | 1 | VCFFLMFCALVKSENCIIDIQDEFWPMQWYLKKGVDDKYDMNVLPSWESCINGTGVTVGVVDKGVQDHFDLNIDRSISFKTAGEDSLENLQHGTRAAGVIGALKNGQLTVGIAFGSVIADIDLSSTNVWETLDTRAMTHKMNDIDIYSCSFANFHTGTKTYPLLDDQEAAFRNGTTLGRNGRGNVYVFATGNTGGTEDDLFRDSCAYDRLVTNRFVISVAGVQHDLSRVPNGEACSAMLVAAFTARAGATDHKVYTTDIRNNYTEHFNQNSAAAPMVSGAVALALSVRPDLTYRDVMHLLVLTSRSNLSELQQSDNFITNAANLSVSSYFGFGLLDIGSLVARSRSWQNVPSLKSCTANNKYTYPVASGYLIVHVDSCQVTYTEHVEVTLRVNHPQAGQIRWVLISPYGTRSIILPGRGLDPTKSMDLTVLTVQMWGENPNGYWRLEAIPVFGAKLDDGMVPLFKLTAYGFSSQDSLDPAVQDSSQFVTPKPNVTDDTEVQDEFLIVKVIVPVIVLVLVVSGIIVFVVWYKKRDSKRQW | PC3-like endoprotease variant B [Lingula anatina] | XP_013403777.1 /4e-78 | Peptidase_S8（PF00082）； P_proprotein （PF01483） |
| **Unigene8830** | 2.2753 | TTDGYLLR | 1 | MAVGKNKRLTKGGKKGGKKKIIDPFTKKDWYDVKAPCMFVVRQIGKTLVTRTQGTKIASDGLKGRVFEISLADLQNDEVSFRKFKLMAEEVQGRNVLTNFHGMDLTRDKLCSMVKKWQTLIEANVDVRTTDGYLLRMFCIGFTKKKNMQVKKTCYAQHTQVKAIRKKMVDIITKEVSSNDMKEIVNKLIPDSIGKDIEKACQGIYPLHDVYIRKVKVLKKPKFDLGRLMELHGEGGASKTVVTESGETVERPEGYEPPVLESV | ribosomal protein S3a [Mytilus trossulus] | ALX27208.1 /5e-178 | Ribosomal_S3Ae（PF01015） |
| **Unigene50450** | 2.0188 | EPVVLEER | 1 | RVFKMAAEKSDSEGSRRHQKSESQGQDGDYEDFGDEPDFSDSEDFIDDITEEELLGDLLKSKPKESDGIDNIIVVDNVPEVGPDRQEKLSKVIRKLFEKFGKIVNEHYPTENDKTKGYIFIEYSSPSHAMEAVKMANGYKLDKAHTFSVNLFSDFDKYGSIPDEWEPPKPKPFKDVGNLRYYLQEADCFDEFSVIYDGGERTAIYKNSPREPVVLEERTRWTETYVRWSPQGRYLATFHGKGIALWGGPKFEQIMRFSHPGVQLIDFSPCERYLVTFSPLQASGEEPNAIIIWDIRTGQKKRGFHCETQSTWPIFKWNPDGSYFARISPDTLSVYETPSFGLLDKKSLKIQGIRDFSWSPTDNYIAYWVPEEMNVPARVTLIQMPSRRELCVKNLFNVADCKMHWQKNGDYLCVKVDRYSKAKKVEEKDQYKYSGMYYNFELFRIREKQIPVDKVEVKENVMAFAWEPTGSKFAFIHGESPRISVSFYSIKHGKVELIKTLERRQANHLFWSPAGQFIVLAGLRNMNGVLEFVDASDVTVMAQNEHFMATDVEWDPTGRYVGSAVSWWGHKVDNAYWLWSFQGRILQKQPLERFCQLQWRPRPPSLLAPEQIKGIKKNMKKYTEQFESMDRLRQSNVSSEQLEKRKMLMSDFQQYRQDKDEEYATFKNRRMQLRDGIDTDLLYEEEIDEEIVEFLLKVEEVVLEE | eukaryotic translation initiation factor 3 subunit B-like [Crassostrea virginica] | XP_022314527.1 /0.0 | RRM（SM000360）；WD40（SM000320）；eIF2A（PF08662） |
| **Unigene27988** | 4.1951 | TLDLPIYITR | 1 | EDLIVSASLDQTVRVWDISGLRKKNVSPGPSSIEDRLRNTGQTDLFGMSDAVVKHVLEGHDRGVNWAAFHPTLPLIVSGADDRQVKLWRMNDAKAWEVDTCRGHYNNVSCCTFHPRQELILSNSEDKSIRVWDMSKRTGVQTFRREHDRFWIMAAHPTLNLFAAGHDSGMIVFKLERERPAHAVHNNFLFYVKDKYLRKLDFNTSKDGAVMQLRGGSRSPVYSMSYNPAENAVLLCTRTNVVENSTYDLYSIPKDSDSQNPDSPEGKRSAGLTAVWVARNRFAVLDKTHSIVIKNLKNEITKKVPAPNCEDIFYAGTGCLLLKDADGVTLFDVQQKRSLASVKISKVKYVVWSSDMSYVALLSKHVISVCNRKLENMCTIHENIRVKSGAWDESGVFVYTTSNHIKYALTNGDHGIIRTLDLPIYITRIKGNSVYCLDREARPRVLSIDPTEFKFKLALVNRKYEEVLHMVRSAKLVGQSIISYLQKKGYPEVALHFVKDEKTRFGLALECGNIEIALEAARALDDPACWEKLGEAALLQGNHQVVEMAYQRTKNFDKLSFLYLITGNLEKLRKMMKIAEIRKDTSSHFQNAMFLGDASERVKILKNVGQKSLAYVTAATHGLNEEAEQLKEQFGENERVPDLFPNAMLLQPPVPIMQQESNWPLLTRTKGFFEGAVSATGAKAAGGLAAVDMDDMAGAGGGWGDDADLVLDDEGGFGGDGLDEEGIAGGDEEGGGWDVDDDLELPADLDVGPTTDGGEGYFVPPTKGTSQAQVWCNNSQLPVDHVLAGSFETAMRLLHDQVGVVSFDTYKQLFLQTYSRSRTCFQGIPSTLPLFGYPHRNWKEAGARNGVPAVGLKLQSLVQQLQVAYQMTTSGKFPEAIEKFRTILLSVPLLVVDNKQEIAEAQQLIEICREYIVGLTMEQTRKELPKSTLPEQKRLCEMAAYFTHCNLQPIHMILTLRTALNLFFKLKNYKTAASFARRLLELGPKPDIATQTRKILAACEQKNTDEHELKYDQHNPFDICAATYVPIYRGKPVAKCPLSGACYLPEFKGQTCRVTQVTEIGKDCIGLRISPIQFR | coatomer subunit alpha-like isoform X2 [Crassostrea virginica] | XP_022308214.1/0.0 | WD40 （SM000320）；Coatomer_WDAD （PF04053）；COPI_C （PF06957） |
| **Unigene27815** | 4.1951 | APLIADLDISHLAGQIR | 1 | MLNLSCGGVVWLIVCLILECKTGNFVSSADAPLLTNLRAPLIADLDISHLAGQIRNLIKEEVQNAVSGLNISSISGLKDNPAFFVTLQQHPFTLHGIDDVVKFDGIKVNIGGGYNRQTGYFVCPKAGVYHFSAMILGNHGHVVHYQLNKNNSPYVIGYSKMGTADSSTVSVVAKLEVGDRIFIKHRYRNEVEQIFGLDHSSFSGFFLHE | putative C1q domain containing protein MgC1q23 [Mytilus galloprovincialis] | CBX41672.1 /5e-43 | Signal peptide(1-22)/ C1Q（SM000110） |
| **Unigene17750** | 4.1951 | FNTIAECMNACR | 1 | KNFGMMWLPLGFITLAIFHGVNGQAANAKQKAAKTVGKMDKKCFDNPRTGMCMGMFQAKWFFNEATGKCVMSQGCFYQGFISMQECRKECQCRQPLNEGSGSGPVGANCELEVQKYAMVGEVCTPFMFTGCGGNGNRFNTIAECMNACREREPLDMMGEMMPGMMGWAGMSGMGGGMMGNMNFRGA | proline-rich protein HaeIII subfamily 1-like [Crassostrea virginica] | XP_022333931.1 /2e-29 | Signal peptide(1-23)/KU（SM000131） |
| **Unigene1265** | 4.1951 | QGYTPGYILR | 1 | LHHPSLQNRMKTTLVFLTALLSFAAAEYGRQGYTPGYILRNYFERQQFSKGLENQAFGGYGYGLGGGLGGGYGLVGGLGYGYGGAVGGLGGYGGYGYGASGYGGYGYKKPFY | — | — | Signal peptide(1-26)/Gly（29.5%)；Tyr(14.3% )；Leu(11.6%) |
| **CL474.Contig2** | 4.1951 | LTIVTDCIR | 1 | VGEDKICCPAGKCHVKDCVYPPRYGRPNPGVCPVPRALTPLQCSRLTIVTDCIRDTDCRDGKKCCETGCDVLKCLTPMINRPECPSVKCTQYTDLCKSDKDCKYGEVCCYGENSDAYCKKCVKRDTTNGHSRRKHSSAPVPMCLTCCGPPPCCGPCISCGAGQNSSHMFCNL | fibrillin-2-like [Stylophora pistillata] | XP_022806910.1 /0.003 | WAP（SM000217） |
| **Unigene2277** | 4.1951 | IINEPTAAAIAYGLDK;ITPSYVAFTEDGER;VEIIANDQGNR | 1 | MKLFLSVVALLAISLVTAKDEDSKKESVGTVIGIDLGTTYSCVGVFKNGRVEIIANDQGNRITPSYVAFTEDGERLIGDAAKNQLTTNPENTVFDVKRLIGRDWSDKSVQADIKHYPFKVVNKNSKPHIQVNVGDAAKSFAPEEISAMVLGKMRDIAEGFLGKKVTNAVVTVPAYFNDAQRQATKDAGVIAGLNVMRIINEPTAAAIAYGLDKKEGEKNILVFDLGGGTFDVSLLTIDNGVFEVVSTNGDTHLGGEDFDQRVMEHFIKLYKKKKGKDIRKDNRAVQKLRREVEKAKRALSSAHQARIEIESLFEGEDFSETLTRARFEELNMDLFRSTMKPVQKVLEDADMKKEEIDEIVLVGGSTRIPKIQQLVKDQFNGKEPNRGVNPDEAVAYGAAVQAGVLGGEEDTGDLLLLDVNPLTMGIETVGGVMTKLIPRNTVIPTKKSQVFSTAADNQPTVTIQVFEGERPMTKDNHLLGKFDLTGIPPAPRGVPQIEVTFEIDVNGILKVSAEDKGTGNKNNIVIQNDNNRLSPEDIERMINDAEKFADEDKAVKEKVEAKNELESFTYNLKNQIGDKEKLGGKLSDDDKSTIETAVDEKIKWLDSNPDATTEELKEQKKELEEKVNPIISKIYQGAGGAPPPGGEEEGSEKDEL | byssal HSP-like protein 1 [Mytilus coruscus] | ANN45953.1 /0.0 | Signal peptide(1-18)/MreB_Mbl（PF06723） |
| **Unigene33388** | 4.1951 | FDVSEFRPEEIQVR | 1 | MAVFRSELHVPVKRDEMNFQDRQVKSWENIESRMDRRQSEWDKEFDKIRNEFFTLKPVDRIKNSPDRLDDLKTIYETDPYGNQKFKVRFDVSEFRPEEIQVRTQDGKISVTAKHQEKSTTASVSKEYSRKLDIPNNVDEDKLQCVLSKDGILTVEGPLKSGHLVTTNTYLPIQQGASSMSPQPTPHNQVAIAKPLDIATPVKNPIITEPDGTRKLRLSVDVGEFKPDEIVVKTAEKKLIVHAEHEEKLSGRTLHKEFNKEYELPESVDQSSITAYIGEEGKLYIEAPLKPAQQKRYSITQTHDVRKVVVTKESQVTITDSQNRPMITINVHRN | major egg antigen-like [Mizuhopecten yessoensis] | XP_021350380.1 /2e-180 | HSP20 （PF00011） |
| **CL416.Contig2** | 4.1951 | ESTLHLVLR | 1 | IQDKEGIPPDQQRLIFAGKQLEDGRTLADYNIQKESTLHLVLRLRGGMQIFVKTLTGKTITLEVESSDTIDNVKAKIQDKEGIPPDQQRLIFAGKQLEDGRTLADYNIQKESTLHLVLRLRGGF | ubiquitin 11 [Arabidopsis thaliana] | NP_001190681.1/9e-84 | UBQ（SM000213） |
| **Unigene34546** | 2.6269 | QLTENTDVINNYLSK | 1 | VNTQQKQLTENTDVINNYLSKIEDLKTGLSEKCFSLEAELDKTRALEDSLLSISNETTALKGQISELEDTSEKMKSEHQIELTNANEELDTLTKNFNELEEELKNSQKTVFDLERQIDLLKEEKDSMNIKIKENQDSFEAEVNDLKQKLVDAEKRLISKESESEEVYGKLIEETN | ribosome-binding protein 1 isoform X4 [Kryptolebias marmoratus] | XP_017268539.1/1.2 | scop(d1bgxt2) |
| **Unigene51624** | 4.1951 | SSQSSLAASLFDLTTTIAK | 1 | MNKLFLSLLVFSVLLAFAHSKPAKKRSNALKSSQSSLAASLFDLTTTIAKIGSSRSKKRSSKKSRRGKKKSSSRKSKSKKSKKSKKSKKSSKKSKKRRS | — | — | Signal peptide(1-20)/Lys(27.3%)；Ser（27.3%)；Leu(10.1%) |
| **Unigene21654** | 4.1951 | LLTAENIIVMR | 1 | MIKIILIICIVGSSFGQAGGWQDYHGPIPIAVLNSVRMKLLTAENIIVMRMETRNAKSQVVTGVNYTFDVKVTSVGGQIVNCHFLVFQGLSGIARVKSHTC | 50S ribosomal protein L23 [Methylobacterium sp. 17SD2-17] | WP_109895574.1/0.076 | Signal peptide(1-20)/scop(d1stfi_) |
| **Unigene27369** | 4.1951 | SGNEIDFWVQR | 1 | MSEDQPVLVQLRRKDPSSPWGFRMNGGRDQGTVLYIQKLARNGIADRSGMRPGDAILKINNVPATYLDHEQAKMEIIRSGNEIDFWVQRNVIDVGQHQPKPQQKSRAEVDEEPSEYKGYTNPNVQSRSFKILQESLNYSEAPQDEEDGGGQMKLMDMSEFMFRSGLLN | LOW QUALITY PROTEIN: PDZ and LIM domain protein 7-like [Crassostrea virginica] | XP_022313065.1/4e-54 | PDZ （SM000228） |
| **Unigene16648** | 2.1035 | QAFVIR | 1 | MSAPEVPLKPKAAPLRTLTLNGHVGFDSLPDQLVNKSISAGFCFNILCLGETGIGKSTMMDTLFNTHFDSTPSTHDLPGVKLKASTYELQESNVRLKLTVCDSVGFGDQINKEESWKPLVDYIDSQFEVYLQEELKIKRSLHNFHDTRIHACLYFIAPTGHSLKALDLVTMKKLDSKVNIIPVIAKADTITKAELQKFKAKIMSELMSNGVQIYQFPTDDETVADTNKTMNSHLPFAVVGSSDEIKIGNKMVKARQYPWGTVQVENENHCDFVKLREMLIRTNMEDLRETTHKTHYELYRRNKLQDMGFSESESQSLSETYELKRQQHLSELSRKEEEMRQAFVIRVKEKEAHLKETEKELHDRFDALKKKHAEEKKKLEDSKSRLEAEMNQFSERKINYQQQSATLGKKGRR | septin-11-like isoform X2 [Mizuhopecten yessoensis] | XP_021348373.1/0.0 | Septin（PF00735） |
| **CL189.Contig1** | 2.4660 | QMLLTR | 1 | ALLLAVVSAMMLQHIYYTLYIVLLTVSDGLSRSPTMPPAPKAICKIDLAFVVDTSVSIGTDDYKKQINFVRDIVDFLEVGPDSTSVGAVSYSAFVKSEFGFDPFKSKMDVLSDISNIEHSQGDATRTYLALEEVHKNLYAPGNGERPDVIDVVVVLTDGGTNPGSYDRFTEDMGKRQTQIEAAKIKSRPAFVFAIGVGSQVDIAELNGISSDPDSKFTILVDSFSKLDSQEIKEMLYKRACKVPAPKPVPTTTPVITTTMQKEEECKETVADIFFVLDQSSSIKSYTNFKKELQFVTNVIDYLEIGEQKTRVGALKFSTDAHMEFYLSGSQTRLELAKRVNKITWEGGDTYMDKALRMVRLQGLTRGHGSRALEGVPQIVVIITDGEATDTKALDAELQRFHDKKYIVFAIGVGPDINIDELKQIAGNANNVYTVQDVYGLLAIRQMLLTRLCTQSNIAYDVIKKP | collagen-like protein-2 [Mytilus coruscus] | AKS48142.1/2e-154 | VWA（SM000327） |
| **Unigene49314** | 4.1951 | ADGDWSECDTALNIR | 1 | MKFQLALVLVALVVVTSAGRKHIDDAGCKFVKGEWSECDSSNQMKRTLTLREQNDGCPATKEQTRPCKDKKSKGCKYRADGDWSECDTALNIRTRKVVLHKGDSHCALEKTNEISCDKYQEIVRRDEERKKARERATAMKAEKKVLKHMQKKLKQLKKIGCQYVEEKSQCNNETQKVTLTFTFESGSDPSCVMEPVEVSCELHEKYEKIKQAKMEKKERKKKEKMERKKNKKLKDKKNKKERRKQRKSEG | pleiotrophin [Parasteatoda tepidariorum] | XP_015920374.1/6e-09 | Signal peptide(1-18)/PTN_MK_C（PF01091） |
| **Unigene30522** | 2.8885 | VIPVTIIR | 1 | MQASMKTDIKLCLCAVILVIFVIGVNSAGSYAGGYKGGYSGGGKYGGGSVYGYGGGSYGKGYGGGYGGGYGYGGKGYGKKVSPQRRGGGTLVIPYRYPVPVPVRRRPRVIPVTIIRRNGGLLGGGGGLGGALGGIFPLIITLALLPLIATLIANIAGAGGGGGK | — | — | Signal peptide(1-27)/Gly(29.9%) |
| **Unigene25455** | 4.1951 | AMVGIVAGGGR | 1 | QQLPFPVNCHPLLVMGRVIRSQRKGPGSVFKAHTRNRKGRAALRPVDFAERHGYIKGCIKDIIHDSGRGAPLAKVVFRDPYRFKQRTETFVAAEGMYTGQFVYCGKKAALQIGNILPVGVMPEGTIICSLEEKTGDRGRIAKASGGYATVISHNPDTRKSRVKLPSGSKKVIPSANRAMVGIVAGGGRIDKPMLKAGRAYFKYKAKRNCWPKVRGVAMNPVEHPHGGGNHQHIGKASTVRRDTSAGRKVGLIAARRTGRIRGTKKVVAKGGDKE | 60S ribosomal protein L8-like [Mizuhopecten yessoensis] | XP_021341018.1/1e-168 | Ribosomal_L2（SM001383）；Ribosomal_L2_C （SM001382） |
| **Unigene12513** | 4.1951 | TEIIILATR | 1 | KMAVQISKKRKFVSDGIFKAELNEFLTRELAEDGYSGVEVRVTPTRTEIIILATRTQNVLGEKGRRIRELTAVVQKRFGFPEGSVELYAEKVATRGLCAIAQAESLRYKLLGGLAVRRACYGVLRFI | 30S ribosomal protein S3 [Enterococcus faecium] | PWS23245.1/3e-84 | KH（SM000322） |
| **CL308.Contig1** | 4.1951 | LFTLTALR | 1 | MASDLTATFVGIGLSEQKAKETLKNDQLSSNLKQAIDQAKKCGASTIDKDMGKLLYSIASKLKAQIKSHQSMIIEYVAQKKISSEIQLNAAMDYLLHHPLPPVDRIKFEEESGVGVTITPDMIEAAVEEVIQKYKSELMEKRYRFNMGTIMGDARTKLKFADGKIIKNEVDMQVLDLLGPKTDADLAKPVSKKGDKGGKSAESSKATKDDAPKAAESFVSEDGEVKSFMDIMGAALKFHKTGENYKTEGYVIYPRTMELLKKHMDEIEGKVHTRFPPEPNGILHIGHAKAINFNFGYARANGGNTYLRYDDTNPEKEEEKFFKAILEMVNWLGYKPFKVTHASDNFDKLYELAVDMIKKGHAYVCHMKSEDIKGFNPPDSPWRNRPIEESLQLFEDMKNGKIAEGEATLRMKTVLEEGKKDPVAYRIKFTPHHRTGDKWCIYPTYDYTHCLCDSLENITHSLCTKEFQSRRSSYYWLCNVLDQYCPVQWEYGRLNLNYAVVSKRKIAKLITSGHVRDWDDPRLFTLTALRRRGFPPEAINLFCAKVGVTMAQTALDPSMLEACVRDVLNVTAPRVMAVLEPLKVTISNFPGDHSGNITVPNFPADESRGSHTIPFTSTLYIERSDFKETADKNYKRWSKEQPVGLRHAGYVLTVDKINKDSEGNITGIVAKCTKTSDTAKPKGFIHWVSNPIISEVRLYECLFYHKNPEDPSEVPGGFLTDINPNSMSVITNAYVDVSVKNAKHFDKFQFERVGFFSVDPDTSHSKMVFNRTVTLKEDPGKK | glutamine--tRNA ligase-like [Crassostrea virginica] | XP_022302781.1/0.0 | tRNA_synt_1c_R1（PF04558）；tRNA_synt_1c_R2 （PF04557）；tRNA-synt_1c（PF00749） |
| **Unigene23851** | 4.1951 | VSGTTIPGYSVNR | 1 | KQCSGSQCYCVTRVSGTTIPGYSVNRWEAQDQNCQCARDKVENTVPGKIFLCDKMGNYKETPCQKEYRENTEATNNGAMGARIPKCGPDGHYDPKQCSGSQCYCVTRIQGKMIPGYSINVWEAQDQNCRCARDKYENTIPGKIFLCDEKGNYRFPCNGYPC | saxiphilin-like [Crassostrea virginica] | XP_022328567.1/3e-19 | Internal repeat；TY（SM000211） |
| **Unigene14356** | 4.1951 | AIQAVYPFPK | 1 | MADKKPDLPEGYLGPQDKFKEMKLQTMSHPDPEITARDNHLLIKIEAKSHIKFTHQLINCRTDQDCTKYVFAQMKEGVLHMHVHMPESDWYKLQIYALPMADQSKSLPNVYNYLINCVRAIQAVYPFPKQFAQWKDGCFLSEPLVLHSNSKLTNINWQVYVPKANAVAVVADGEWFHFENRGGPVWTAKFSLDKYRNKNTKVTLSANFQGAEESKYSTLLEYLLE | LKD-rich protein-1 [Mytilus galloprovincialis] | AKS48185.1/2e-129 | — |
| **Unigene16633** | 4.1951 | GDLLFLTNYR | 1 | MFGLMEADFLDDVRRMNKRQLYYQVLNFGMIVSSALMIWKGLMVVTGSESPIVVVLSGSMEPAFHRGDLLFLTNYREEPIRVGEIVVFKVEGRDIPIVHRVLKVHEKEDGTVKFLTKGDNNSVDDRGLYAPGQLWLEKKDVVGRARGFVPYVGIVTILMNDYPKFKYAILACLGLFVLLHRE | Signal peptidase complex catalytic subunit SEC11A [Crassostrea gigas] | EKC32404.1/6e-122 | Peptidase_S24 （PF00717） |
| **CL2205.Contig2** | 4.1951 | VVTEAEER | 1 | MIRIFTLMSVFVCVQCLRTTTSSPEGSTDIPEEIERGEDVTTNNSSIPTTPFANDPMEQNDFYNPTRKPPAGKIQNTARQSKAIKSTTKKTTEKVVTEAEERVQSTTIEAEEMEPEEATVRQVYSYVTSAPEVYPTYSLFTTVPDYSWIWWLSKK | Kinesin-like protein [Actinidia chinensis var. chinensis] | PSR99819.1/0.24 | Signal peptide(1-16)/Thr(14.8%)；Glu(11.0%) |
| **Unigene542** | 4.1951 | NFQQMQNPMMNQMQMMGGPR | 1 | GGGMGMGGMGMGGGMGGGMMGYPNGMMGNGMMPGGGQGMMGNNAGGGPGAGGAAGKGGKAKGKKQPAVEYEPPEAPAPTKKPAKGKGGAAAAGEAGAPNVNIQKHAMQHQQMQNRNFQQMQNPMMNQMQMMGGPRQQRYSQQQRMGIMAQFSQSMNQMMGTFNDIRASLAKLITTLSKFVKLQK | — | — | Gly(22.3%)；Met(14.1%)；Gln(11.4%)；Ala(10.3% ) |
| **Unigene5795** | 2.1035 | IVIGLFGK | 1 | LRTGNLNFHECTRKLLRRKKIVFTMILGCIVTILIVPAFMTNAAKGPKVTDKVYFDIEIGGEKAGRIVIGLFGKTVPKTVKNFKTIAEGTEERDGKKLTYKGSKFHRVIKDFMIQGGDFTRGDGTGGRSIYGEKFADENFKLKHYGAGWLSMANAGKDTNGSQFFITTKKTEWLDGRHVVFGKIIEGMKVVRKIESNPTAAGDKPKKEVVIADSGSIAVDEPFAVEKSATPDSV | peptidyl-prolyl cis-trans isomerase B-like [Crassostrea virginica] | XP_022330620.1/1e-120 | Pro_isomerase （PF00160） |
| **Unigene15218** | 2.7302 | QAVDVSPLR | 1 | MTETWDEPATTVAELPEIKLFGKWSSDDVQVSDISLTDYIAVKEKYAKYLPHSAGRYQMKRFRKAQCPIVERLVCSLMMHGRNNGKKLLAMRIVKHAFDIIHLLTGENPLQVLVNAIINSGPREDSTRIGRAGTVRRQAVDVSPLRRVNQAIWLLCTGARDTSFRNIKTIAECLADELINAAKGSSNSHAIKKKDELERVAKSNR | 40S ribosomal protein S5 [Mizuhopecten yessoensis] | XP_021375269.1/9e-143 | Ribosomal_S7（PF00177） |
| **Unigene53044** | 4.1951 | GYQDVIFGGFDQR | 1 | DKSSVGRNISTKVPDGMPVKPYLTDVASIRQDITDDYKYKDESDRERLAVINANRSALKTRPVYNRGYQDVIFGGFDQRDFIMIGKEFKVTVTFRNVGDHVRTIKGRLICESVSYIGIKQKTIKEHSFEIPLDPRSEDVASMKVGLTEYLPHISEQMGMKVSAILQVVETGHIQAFQDDFRLRKPDIQIEILDNHIRVGESFKCRLSFTNPLSAALSKCCLTIEGPGLDNEEQFDLSNISGHQEWAAHLQLTPRKPGRRQITASLDTHQIQNIVGVAEVKVLP | Protein-glutamine gamma-glutamyltransferase K [Mizuhopecten yessoensis] | OWF48045.1/3e-98 | Transglut_C（PF00927） |
| **CL3476.Contig1** | 4.1951 | DGFIDVGDLR | 1 | MSGKEDDAQAQKARAATSSVLTKFNQKQIQEMKEAFTMIDQNRDGFIDVGDLREMYSSLGAVPQDSVMKEMLAEAPGPLNFTMFLSLFSEKLSGTDPENTIQNAFSMFDDAGKGYLPEAYIKDLLQNMGDNFTQDEMRQTFKEAPIEGGKFDYNKFTAIIKGSNVED | myosin regulatory light chain B, smooth adductor muscle-like isoform X3 [Crassostrea virginica] | XP_022340190.1/8e-89 | EFh（SM000054） |
| **CL2825.Contig1** | 4.1951 | AGFAGDDAPR;AVFPSIVGRPR;DLTDYLMK;DSYVGDEAQSK;GYSFTTTAER;HQGVMVGMGQK;IWHHTFYNELR;QEYDESGPSIVHR;SYELPDGQVITIGNER;TTGIVLDSGDGVTHTVPIYEGYALPHAIMR;VAPEEHPVLLTEAPLNPK | 1 | MSDDDVAALVIDNGSGMCKAGFAGDDAPRAVFPSIVGRPRHQGVMVGMGQKDSYVGDEAQSKRGILTLKYPIEHGIVTNWDDMEKIWHHTFYNELRVAPEEHPVLLTEAPLNPKANREKMTQIMFETFNSPAMYVAIQAVLSLYASGRTTGIVLDSGDGVTHTVPIYEGYALPHAIMRLDLAGRDLTDYLMKILTERGYSFTTTAEREIVRDIKEKLCYVALDFEQEMSTAASSSSLEKSYELPDGQVITIGNERFRCPESLFQPSFLGMETAGIHETTYNSIMKCDVDIRKDLYANIVMSGGTTMFPGIADRMQKEVTALAPQTMKIKVIAPPERKYSVWIGGSILASLSTFQQMWISKQEYDESGPSIVHRKCF | actin A1 [Haliotis iris] | AAX19286.1/0.0 | ACTIN（SM000268） |
| **CL1159.Contig1** | 2.0188 | GSSYQAIR | 1 | MPGSQRSKSRSRSRSRSRSPAKKSPVKKTKRKSPKKGGKAKGGAKRPTTLAMIVTAIQAMKNKKGSSYQAIRRYILANNKNVSQAHLKSAMKLALARGLKTGVLVRPKASANVVGAIGTFRVGKVPTAPKKKKPAKAKKAKRSTAKKNKSNNAKRRKSTGGKKKTVKKSTKSKAKKPKKSPKKKKPAKKAVKKSPKKKASKAKPKTKKAAKSK | H1/protamine-like protein precursor [Ostrea edulis] | AAO16249.1/3e-34 | H15（SM000526） |
| **Unigene38481** | 4.1951 | MLVGSEFVGAVIGR | 1 | NRPLNNRRMGSRPNGQNQQNRQPEFPLRMLVGSEFVGAVIGRQGQTIHGITSQTRARVDIHRRENISSETLVTIKGSPESCTEACKEIYKVVQQEAQSLNKGEYPLKVLCPNSVCGRIIGKQGNVIKNFMEQTGAHIIVSSATDMSNFYVDRVITITGSVEACSKAEALLSEKMRKCLEQDAQSYNQQMNMFGGMPPMGMMGPGPGMGYQNMRNFPPYGMVRRNEESNYSGYPGIYGAVPNVPPQQELEVTYLYIPENTVGAVIGSKGSNIKEIMRLSSARIKILPQKNGEMNGDRKPGPPNQMEERKVIITGSAESQWKAQFYIFDKIKSEGGFARIEEVHLRSEVMVPRSMVGRIIGKGGQNVREMQRVSGAIVKLPEVNSQNSQMSDDTEVAVSIIGHFYAMQPAIRRIRSLVNQQGGQQGGGRMGGPPLQRRGPRREQNGN | insulin-like growth factor 2 mRNA-binding protein 2 isoform X1 [Crassostrea virginica] | XP_022305782.1/0.0 | KH （SM000322） |
| **Unigene5808** | 4.1951 | EGHFVWESSSESLR | 1 | MVGTGKEVFVFGFQPQLTHGLVLIANVNVWVGSWQNQKNSDIDGYISRMGTSKDVMYWIGLSDLKREGKFVWKSSGQAVRYTNWRPGDPNNYKGIEHCGLTNWNKAGEGWVDAPCNWKMNYVCQAKLECAPGWHKKGKSCFWFSDITDTWYGAKEFCRKVGAFLAEPKDAETEKFITNDAIWKRVNSWIGLSDLKREGHFVWESSSESLRFSNWVPGDPNNYNGNEHCVLTNWHRKWADAPCTWRKHFVCQGNL | macrophage mannose receptor 1-like [Crassostrea virginica] | XP_022290497.1/6e-34 | CLECT（SM000034） |
| **CL249.Contig2** | 2.8885 | EPFPNVNR | 1 | RNAHATSFLVGAMAAGTLYTYPDNFRAFKAQIAAQFSGTDIKLASGFKFGETNQSKDFLSKFPLGKVPAFVSSKGDCIFESNAIAYYLGNPQLRGTSDKDASLVLQWINFGDNEILPSACTWVYPCLGIVQYNKQETEKAREQIKKALSVLNNYLLTRTYLVGERITQADISLACNMLSLYKYVLDPKFREPFPNVNRWFTTVVNQPQFKKVVGDFKFCEKMAEFDSKKYQELHGKGDGGKKDKKKEEKKQDKPKQQPQAKKEKPKKEEEEEDDGFPKEKEQKDPFGQLPRGNFNMDEFKREYSNKDTIKEALPYFWKNFEKDNYSIWFCNYKYNDELTRIFMTCNLVGGFYQRIDKMRKNAFGSMCVFGEDNNNSISGIWFWKGQDLAFKLSPDWQIDYESYDWKKLDPTSAETKKMVQEYFAWEGDFGGKKFNQGKIFK | elongation factor 1-gamma-like [Crassostrea virginica] | XP_022332141.1/0.0 | GST_N（PF02798）；GST_C_2 (PF13410)；EF1G（SM001183） |
| **CL4206.Contig1** | 4.1951 | EVMGHESELFR | 1 | MAGLVKAKKYDWKDSNMALFGSDLERQVKKDSAMGEPAWNGAGEAVGLKIWRVVKFQITDWPEEDYGKFYDGDSYIILNTYKKNEDSEELCYDLHFWIGKHSTQDEYGTAAYKTVELDTFLDDKAVQHREVMGHESELFRSYFKSIMTMQGGADTGFKHVLPEEYRPRLLHFSGKRNIQIKEVPYLQSSLKSGDVFILDMGMQVYQWNGAESSGMERIKAAQYLQEMESERPSCSTTVLDESGISKAHRFYEAIPEEGEDDSGDSDDEDVDGPDEKKLLRLSDAGGSVQFAEVKSDGITMDDMGEDDVFVLDTRKELFVWVGAKASVEEKRNGMGYAHRYLMDKARHYKPVSVVSEKVGRERVEVAISA | gelsolin-like protein 2 [Crassostrea virginica] | XP_022299451.1/9e-151 | GEL（SM000262） |
| **Unigene50547** | 4.1951 | SAYLTLGAMADR | 1 | KEEATSMTFTFADELRNADSDLIRDIWGQYKEGKDKSNDEKYYKWKLMLDTLPALGTPSAVEVLVDYIKKSEIPHEGAVHALIGLTVTASPDINIAKYLLELVRDQNVRSHEAVLRSAYLTLGAMADRIRSENDRRRRELERKERKMKKLKDRE | vitellogenin-like protein [Perna canaliculus] | ACV04918.1/6e-23 | Vitellogenin_N （PF01347） |
| **Unigene32892** | 4.1951 | LGITDIFAPSR | 1 | QSLSGQIFRTTGNDVNIATKLFVKNGLTVTADVRNVATTYFDADIGNQDFTNAISSAKDMNNYVASKTNNKITDLVKSNWLNINTVMVLVNAVYFRGIWETQFDPKNTQKEDFFLTNGNKIKVDMMSMKHYIRYYNGGKYSAIALPYKGKQYEVVIVKPSSFNGLRDLKRLFSPALAKVIDSRLSNTTLLVKIPKFTFEAETDLKVILPKLGITDIFAPSRADFSNLVQEVQTSMYVSEAHHKVVIEVNELGTEAAAGSAIVVTRKSQLQEFVANHPVLFYIRHVPTGSILFIGHFNP | Leukocyte elastase inhibitor [Mizuhopecten yessoensis] | OWF49040.1/2e-54 | SERPIN （SM000093） |
| **Unigene17193** | 4.1951 | GLVVPVIR | 1 | LKSMALLLVQRCLPRITRRLSPTNTKFVLEEASKNIRVKSHICCSSQCRNYTDVQYIRFTKKYCCNSQLIIKRNFHVTNRYFDDVLTAATPPFADSISEGDVRFEKAVGDFVKEDEIVCEIETDKTSVPVQAPKSGIIQSFLVDDGATVQAGTPLFTLKLSDSPGESAPESVAASEKPPPPAVTSKAPETPVATPASGPIPTTPPPPQPIPKAPISTKPLDSIKPIPATDAPVMGARTEKRVKMTRIRQKTSQRLKAAQNECAMLTTFNEIDMSNVIEMRNQYKEAFQKKYGLKLGFMSAFVKAAAYALTDQPAVNAVIDESEILYRDYIDISVAVSTPKGLVVPVIRNVGSMNYADIERAIAELGEKARTGSLAIEDMDGGTFTISNGGVFGSLFGTPIINPPQSAILGMHAINDKPVAIKGKVEIRPIMVVALTYDHRLIDGREAVTFLKKIKSAVEDPRVLLLDL | 2-oxoglutarate dehydrogenase complex component E2 [Mizuhopecten yessoensis] | OWF50391.1/0.0 | Biotin_lipoyl_2 (PF13533)；2-oxoacid_dh（PF00198） |
| **Unigene35334** | 4.1951 | FLGGGVSVVTGGPSFGGR | 1 | MRPLVILALLVAAVSASGKYGGGETVIVDESVYNGEDDSTGGSDGNGGGGDGGGGDAGDASGDASSSGAYGAGRYWCTCRPKKCFYKELTLDEKKCRLAPLFPKPWGTCCRWFPWWATDNGGGRGIGFGAGGGGFGYGGVGLGFPGVGGGYGGGSFKAGGIGGGFGGFGGSYRGGLAGGYGGGIIKGGRIGGIGGLGGGLGGGYGGGYKGVGGGGGFGYGYSSGGYGPRFLGGGVSVVTGGPSFGGRGGYGYGPSGKLYG | — | — | Signal peptide(1-16)/Gly(20.4%)；Thr(11.8%) |
| **Unigene89** | 2.7302 | FGAATGHELQR | 1 | MSFSHIFGTAVFFINVIYVFAQRPSQIGQNPFAATQGFGMRQGMQNMFNGQRRFGGGNGGFNIPPPAGFQQVNRQFQNNMPFQLQNALPRNPTADGNNVNGVLSVLNGGGAGFTGRFGAATGHELQRQLTLTGEPGAVFTGTNNPQARPGFTQFNQRQNQLPSAQLNRIQQPNMFLNRPGQNPNMPFQRPPMMQFGQQFGRQGMIQPSVVGNVPPTQSRGPRNQPQAVMPMNVQGIDMSSILPGQRLPNEPSLVNRGMPSNQQQGQTTRFGRNTMMQPQQPQTFGVPSQTIRAPQATIRQQPSQTFGPQQIGQPQTFIPPQPVAPQPSRPTQIASQGQSSRTSIAFPDQTLTQSLGVGRPASQVNVPDGAPPMLPGEVEIAPPISPAEAAQMQGSSANPPQAVSEIITISDINGNVLYRGVDLSDTEIEKITQQLVSNMTDAQAAEAAARGRELEAQGKLDSAGAPQSTGQTSSASLSSQPSTQQASGGFSSWGQQSPGIRFPGQNVVGESRTVVDFLNPSNTNEG | — | — | Signal peptide(1-21)/Glu(15.9%)；Ala(11.7%)；Arg(10.0%) |
| **Unigene54678** | 2.0793 | LDLYGK | 1 | MATYKVTYFNGRGRGELTRLLLVAAGQQFEDERLTREEWTKIKHTMPQAQVPVLTVNGKVLPQSGAINRYLARRLDLYGKTNEEMTINDIVMETVNDVRNALVKAHFEPDDAKKVEMFKNVKEQTIPNFLKVMETLLNENNGGNGFFVGSELTVGDLALMDLMDIILQRISPDALENQDKLKAHRERVCNSPRIKKWLETRPQTEM | glutathione S-transferase sigma 3 [Mytilus galloprovincialis] | AFQ35985.1/4e-61 | GST_N_3 (PF13417)；GST_C （PF00043） |
| **Unigene3834** | 2.1035 | GVLMVGPPGTGK | 1 | MSSATLLGEIDENTKLGRENALLGNYDTSLVYYQGVLQQIQKLISTISEADRKRKWMQARDLISQEHDQIKEISETLSSFKSNNPKPYPDNDFGSPFGDYARHEEPTRDPDVWPPPTPVEYRPSPNIRGGRPAPKKVEPVRRPGGPSKAAPSRQPDRGRPGAPGYGRDNRGRDNKDNKKKNDDGEKKFDPTGYDKGLVEGLERDIIQKNPNVNWDDIADLTEAKKLLQEAVVLPLVIPDFFKGIRRPWRGVLMVGPPGTGKTMLAKAVATECGTTFFNVSSSTLTSKYRGESEQLVRLLFEMARFYAPSTIFIDEIDSICSKRGSDSEHEASRRVKSELLIQMDGMYEGVGGSADDSEEQKIVMVLAATNFPWDLDEALRRRLEKRIYIPLPTAVGREELLKINLKGLEIAKDVKLSQLAETLEGYSGADITNVCRDAAMMSFRRRISGLTPEQVRNIPKEELEIPPNMEDFEMAIKKVNKSVSAQDLEKYQNWMKEFGSV | Katanin p60 ATPase-containing subunit A-like 1 [Mizuhopecten yessoensis] | OWF43912.1/0.0 | PDB 2RPA\|A；AAA（SM000382）；Vps4_C（PF09336） |
| **Unigene48684** | 4.1951 | YAVMPIILAEQHR | 1 | NYLSPLRPSLVKMAKMGYAQDMPPPGGYGPIEFERKLPKKGNVFLKVALFAGITTYGVVTFAINKKRWRQRKIEMNDARYAVMPIILAEQHRQMMKHWRRNRDEENELMKDVPGWETGTLYGEPIYYNPRGRWIDVNEVEYYAHNSEQVMLEDTKLKRTYGIAHRIGTGDAKV | NADH dehydrogenase [ubiquinone] 1 alpha subcomplex subunit 13-like [Limulus polyphemus] | XP_013789118.1/6e-33 | GRIM-19（PF06212） |
| **CL3608.Contig2** | 4.1951 | IPIASLSLGR | 1 | TTHSLPSTMNDPAREYFWGITLDKENSSHTWSFDEEDEDSDYLIHTLFLKMAVLGASAVKDERNIIQVETQNFDQKELKIPIASLSLGRLDQTTVDVSFGHEVPVVFRLVEGSGPVHLIAQQLVEYPEDKYPSQNESEFECTEEEDEEEEEEEESPVKSKKRKASSQKEGKSKKKGKMEVVSQSESDEDEDDEDEEEDEDESMEEDTEEDIESSPEKGKKSKKAKDTKNEKPKKGGKLAKASPKAVKKGKKAGKAK | nucleoplasmin-like protein ANO39 isoform X1 [Mizuhopecten yessoensis] | XP_021358909.1/5e-54 | Na_trans_assoc（PF06512） |
| **Unigene1420** | 2.4029 | DHLIDDALVALEMTAMDNPQDLKK | 1 | KLAKVWSKFGQDWLLEKVRSLHQLITVKIVNNEGRWGRSYHINDDDGIAGATKMMKILYYASIYGGDRDSEQTISEEKAVNEVDENLQDMLQLQGAVGHSEPKESRHQKDDPFAQQMNVYCVDCRNPLVPYDEFINELLNEYLDVETDYKYKDDTEDNKISFMNHSYILTTSSKFTLMYFDNRVRMFNERRTSILQTFVHGLPPIPFLRLQVRRDHLIDDALVALEMTAMDNPQDLKKQLFVEFDGEQGLDEGGVSKEFFQLVIEEIFNVDFGMFVYNEEQNIFWFNSTSFENDGQFTLIGIMLGLAIYNSTIVDVHFPPVVYRKLMGKKGMFEDLNEVDPTLASSLKDLLNYTGDDIEDVFMQNFQIGYKDVFGSSLTHDLKDNGSQIPVTQENKQEFVDLYSDFILNKSIQKQFRAFKRGFQMVVNESPLKNLFRPEEIELLVCGSKVFDFNALEQATEYDGGFTADHRTIKNFWEIVHEFSEDQKRKLLQFTTGTDRVPVGGLSKLKLIIARNGPDSERLPTSHTCFNVLLLPDYTTKDKLKERLIKAISYSKGFGML | ubiquitin-protein ligase E3A-like isoform X1 [Mizuhopecten yessoensis] | XP_021348287.1/0.0 | HECTc（SM000119） |
| **CL2378.Contig1** | 2.8885 | GYTNAPTTQYIR | 1 | MGGIRIKFSVIFLFCVWTVTDISKAHEGEHHGSQTNHMIVTKQSVVQIPESKDTNKCIALLRRTVVRKELLTYPCFHPVPGVVPVVAHQTPMGLVCRGYTNAPTTQYIRLPTCCPGSQPDYRGRCVKDVKATTEPEKKPEPYTHQQQSAPTRRLEELPKYPASYENKRKEVFRKVMSQPRHWMPHPMIMRRIDAIMKQRKHLIEQLKKDMEESKQRQVPPRTSISDLFPSIPHPLLGKHNQQQKPSAPEIPRPSQQARAKEAPHHHHHHMSHHMPHHIPHHQMAAELHAALHKNNIPHSHDDQGNPQMHELKPSAAFLQQQEQLKQQMEARKHMSRQHQLHMLQQQKQKALAMRQQMLHHRRMQQAQLQRQQMIEQMRNAAQQSYRQMPLVPQQQRPSVQPYVVQRTFTSNNRQFPNVPNMRASVMVAVSPNSIQHFVQKTIQQSFPSIPQHPVTRYRAQLPRIPVESPPVSISPGPNSPFKLFWKCRHHHHQMLNDVAPAIHAQPSPTCFTQYMMVLKKCFNAAGVDIPIDHNMFNVNFNYNGVCEKREMIMTCIFDNLANCNTPQEQHLVKTTIGNTVDEMNRFCQIGQINGIEERLGGRGDNMVPPAMEQVQQQEVVPMEPVQEMPIIPQDHHAMSLGQHVLSHEVQEETLPKLNEVPEQQHEVQKVPEEVKPVKDEKKPAVTISEHEDSENIPGHAHAHVNGADHDHDSHISIEELREESFYLPILIGTAIGVAGIFILLLAILCICCRRRLHNKMYIEKETEKPKLFDVYTIGVPPPVYEVNGIPPISYEEAKGEKFTGSPASVRRNNAENESQQQGVSTISGSVSDI | uncharacterized protein LOC110452343 isoform X2 [Mizuhopecten yessoensis] | XP_021356476.1/9e-67 | Signal peptide(1-25)/~ |
| **Unigene4478** | 2.1035 | LCPYCPIR | 1 | NNQETVKYGIQHRERKHISLEMQFNKDIFLLLVLCLHVRYCETVIIGEWTQWTSWTSCSVICGQGTQDRIRQCYFGSSCFGPAFQRRTCISKPCSRLPQWSAWSAWTECPTSCEHRDRIKLRSRTCSEPGRCRGDGLQTRLCPYCPIRSTRPSIPQWSAWSEWTDCPASCEHEDRIKFRSRTCSQQGRCRGNGIQIRPCPRCSIPPNRPINSQWSIWSQWSPCSESCGYGSKERTRQCSGRYCPGESNERRTCFERICPTDCTDTRRDCPGLVSLYGCTYGLWNYNNCRKSCNRC | coadhesin-like isoform X2 [Orbicella faveolata] | XP_020632436.1/1e-30 | TSP1（SM000209） |
| **Unigene44068** | 4.1951 | MNVLADALR | 1 | MVRMNVLADALRSIAAAERRGKRQVLIRPSSKVIIKFLTVMMKHGYIGEFEIVDDHRNGKIVVNLTGRLNKCGVISPRFDIALRDMEKWTTNLLPSRQFGFLVLTTSGGIMDHEEARRKHLGGKILGFFF | 40S ribosomal protein S15Aa [Mizuhopecten yessoensis] | [XP_021374413.1/ 1e-85](https://www.ncbi.nlm.nih.gov/protein/XP_021374413.1?report=genbank&log$=prottop&blast_rank=1&RID=JCENSRBY014) | Ribosomal_S8 （PF00410） |
| **Unigene13256** | 4.1951 | LAEDVVNELLR | 1 | AILLSQCTAQFGPTVLPQTAIVSRPPDSSGGGFPQPCLQVLAKGGDSPYPPTSVARIYSMFCPVINEFATCLELHLNESSVEYFVLLSKHFDKNLMVSRMQHLCSLIPSDATSLDTLKTNNSDVQACVANFNREMARPSQPLTLNILCRLLDEHVFCMRQYATPDVIPENVLEVYFAYTLIFTPGQCNPPIPSTFMPTINTPPAVSNDPGQTWLAQSVVARCAYETYGEVIKNIMAIYKTHTNQLKTVGQVLGQAMGSLCQNATRFSDCISQNVKQPYSSIDAGLSVIINLQNLGSSLIQICSTSMTQSELMCLFQSGSSMPVCGSLISASTKLLQRSRSSTTPTNSINQLLSYCPLLREGDTCARIQLQRCSPALAETFSAVEKQLLTTNCYYADASVINASPYSNRMAYCAAPVMAEAYQRFANRPWAFDDEHVTSFACKESSNLATCLSREVGISKNLVDKMTSKLIDFSSGPRLQYIFDTVCNNTEEILRQSRCMVNATNSFRVCKMEQSYKDLQARYIFNFVFNISGIDVNGVTAFCKPYKELALCNARQISTCSESLSRLAEDVVNELLRMDCGGSRPLTQYMNFNAASDHKTVKVTLLLCIMLLLNVIM | — | — | Internal repeat |
| **Unigene22659** | 2.2753 | VNGFVQR | 1 | TLKQVLAKLPGLTDRVNGFVQRIFDDDILGFVEEEEFESIGCFLRELKQNVFSKDVASLEDLKSKFKDAANQDDNVFTCTDPQEVLDYYKNGLEILNTP | tetratricopeptide repeat protein [Cecembia rubra] | WP_106568208.1/3.9 | Leu(12.1%)；Asp(11.1%) |
| **Unigene29280** | 2.1035 | TYRPKQEYHMPDVITVR | 1 | ENNLQWRLSVHDCLDLKKKMADQEGSAPTEQKTEVPTEESEKQEAVAEVSEKAEESAPEVQEAEEKKEDQSQEQSEQKEEQTEQVEEKKDDPQDVQKEQGDGQTEEKVEGQTEVSPEDQGEKGEDQAELTEVDGQSGEKVDGQTETKAEEPSETKPDEPERDKTKESTDIPAPPGTPQPTQHSMEPEKVEPTKPNLDATATVKIVLMPSGQVVTLACTLGQTLKELKDHFATELKMQFNMMYLMFDGKQADDNLALADLGVGPNGTVQLELQSTDPVNTPIKTYRPKQEYHMPDVITVRIQGGEEGEEARDVVVEIERATRKKPFIGGYKHKQSGVEYHHASAQTMKKQPPPSLVPKYCRDTQTVEQKHQVQQTTNDMSTQMTKIGVFISNNPDKLMTPGKYTTADDHHYMILQRIIIIQKYYRRWLAKRYVNKLKEDKKRREEFEKQEEIRKKKEKEERIRKEFQRRMNPKTKEDFDLLYHALEKWRLEEMERIDQTLTGAERKAALCMLLDQETQLISAIGRHKIEADGENKDKRIQQFLSKAAAPKKWKGFDGKTTEMDTPYTIRAKELQDIYNSINMKYLTQDERLDVLLTLKHTVKEHDCKLTQEIIELIDREADLLMRGVRENNLDGLRKRISTLFLQYIKTPTFNPEAAKLLKVPQDPSVLRKNIYFCPSCNQYLPSTDFALSSNSRNVGKCRRCAKIDNDARTRQDFSTYQHMLKCLRKSEEGQRDGSRIAFLLQEQDLRYLVEKIWSGQSILSAWEDLYDLTFVRWDKHQEWSPWNCILLTKDEAASHVKLVDLEEGYGQVFMHRVKQKHTLARNYFSRLPGMAEHVRERANEKTMSLGPSVAGQPAPVQQRA | IQ and ubiquitin-like domain-containing protein [Mizuhopecten yessoensis] | XP_021341824.1/0.0 | UBQ（SM000213）；IQ（SM000015） |
| **Unigene26528** | 4.1951 | LTVADALEPVQFEDGEEIVR | 1 | FGELALIYGTPRAATVKAKGDVKLWGIDRDSYRRILMGSTIRKRKMYEEFLSKVSILENLDKWERLTVADALEPVQFEDGEEIVRQGEPGEDFFIIVEGQASVLQRKADDEEPVEVGRLGLSDYFGEIALLLDRPRAATVVARGQLKCVKLDRGRFERVLGPCSDILKRNISQYNSFVSLSV | cAMP-dependent protein kinase regulatory subunit isoform X3 [Mizuhopecten yessoensis] | XP_021346603.1/3e-117 | cNMP_binding（PF00027）；cNMP（SM000100） |
| **Unigene3465** | 2.0605 | GLDTLGNMGFHDYR | 1 | IDRISGVHHDGIREITDKINWEKLYEKLLNSDNKSFEEVLKDLSPDDLLVYEDTINPDDIEKVRTFLLSKLEKNKKYESQSKRGLDTLGSMGFHDFRKRGLDTLGGMGFHSFKKRLNGQNGYTDFGKRGLDTLGGMGFHNYKKKGLDTLGGMGFHNFKKSVSDENQAHIIKKRSLDTLGGMSYHDYKKRGLDTLGNMGFHDFKKRGLDSLGGMGFHDFKKRGLDTHGGMGFHNYKRGLDTLGGMGFHDYKRGLDTLGSMGFHDFKRGLDTLGGMGFHNYKRGLDTLGNMGFHDFKKRGIDTLGGMGFHDAKRGLDTLGGMGFLDYKRGLDTLGNMGFHDYRKRGIDTLGGMGFFDYKKRGLDTLGGMGFHDYKRGLDTLGSMGFHDFKRGLDTLGGMGFHDSKKRGFDTLGSMAFPIYKRQLDINMADEYPSDEEIADILDDLNDQLSLADDNTILNSQSNTGRLKRNVNMAEEPPKQKSRR | feeding circuit activating peptides-like [Crassostrea virginica] | XP_022335398.1/2e-59 | Internal repeat |
| **Unigene51161** | 4.1951 | GLVVEAVDEQGR | 1 | MEQISFFCFRCLTLSVLFKLYFCNPARVDLRDHDLSHISSEQLRRVVQQNENQDITTDDDNLLEKLYNQTQYHPTNDESRCISCTIREDQKKHRIESIKNRISHALRIDVLGKPNMTNTKLPKIPQFQKLKERYEIREALDMQNDQYRGEREDYEDEFGQSHRTFTFAQNPPEELGIQQPNAIYFDMPDQTDRSLQKATLWVYVTPSDQQHVTEIYLYTLVKRSKSSDTLIKQFLYRKKRTSRGWQQFNLLNEVEKWTEDPSYNRGLVVEAVDEQGRNVVVMPSSDDNGYQPVLETRTSPHHQHSRNKRSIYLDCSEQRATEACCRYPLTVDFVEFGWDFIIAPLTYSAYYCAGECRNQHMDSGAHSYLQQQVGNVPTEHGPCCSPTRMGHLSMLYFDHSMQIQFTTLPRMKVERCGCA | myostatin [Mytilus chilensis] | AGU13048.1/0.0 | Signal peptide(1-23)/TGFb_propeptide（PF00688）；TGFB（SM000204） |
| **Unigene26699** | 2.3588 | GTIHVFPVR | 1 | MRVISWIIAITLGLYQLSQVSGCTCNPSTFHEQQYFCDADFVIRAVVLRRRYIGNNPPNREIRYTVQIEAIYRGTIHVFPVRRAIIYAYEGSSLCGVILEVGDDMLLTGHRSNDGQYRLSSCNSWITHWDSLTQLEKLGLRYGLYTRACLDGCNIVSDEQDGDQLCDMGFPVCCHVEESPCLGSVCLRTSQGRCDWSRCLRE | tissue inhibitor of metalloproteinase [Tegillarca granosa] | AFB81539.1/2e-17 | Signal peptide(1-22)/C345C（SM000643） |
| **Unigene58311** | 4.1951 | NVQNFLGLVVGR | 1 | ITLEDDADVIVQDLNFTSKIVALLDQFSPRIVANYVILRFLRYRLHYLPKRFEDLSLDLEKLTDGKTTSVPRWRKCTRNVQNFLGLVVGRMFVKDNFVP | neprilysin-like [Mizuhopecten yessoensis] | XP_021369615.1/2e-17 | Peptidase_M13_N（PF05649） |
| **Unigene46263** | 4.1951 | TTITTVDSGSSR | 1 | NCPASRSERICNTQPDQCRECEVKGRRYRGNSRFSYDEDCYRYNCDCNCNGSWNCPASRTQNICGSGQDQQIGESRSTVTTSNSGSITRTTTTGSGCRECEVKGRRYSGNSRFSYDDGCYRFNCDCNCDGSWNCPASRTQDICRSGQGQNTGGSQTIVTTLESGGSTRTTSTGTGCRHCEVKGRRYRGNSRFSYDEGCSRFNCDCRCDGSWNCPASRTQNICESGQRRQTGGSRTTITTVDSGSSRTTGTGCNECEVKGRRYRGNSRFSYDEGCYRFNCDCNCDGSYNCPSSRTQNLCGSGQGVRTGTRTTLTTSTKTTGSGESAIGNGVDSRRTGSSQISGSRRSTGSGVSLTESRRTGGGLSRAEKTFTETRTSTSSRSTSSKTVVGGSQTKTELRSRSGSTTGSAVDSYGCHYCIVNKIQHRGNSNFQFEDNCFRFSCECRCDGSWLCPADRTINICPDRCQNCNVNGKSYPSNTIFEYDEGCNRFNCDCACDGSFNCPAERTKDICSGRQDKCKECNVNGRRYQPNTQFSYDERCYRYICDCNCDGSYRCPAERTKNICGSLRADTCQECRVNGKNYEPKKTFSYIDGCALRKCTCDCSGRHRCTATTDICKSSSQVCRDCVVKEKRYLPNEPFKYTEGCDRFNCDCNCDGSWTCPAERTENLCAIGQCQKCSVRGEVKEGNSGFVVTENCRQYKCWCHCDGNWSCSKDFTRSCNLG | Kielin/chordin-like protein [Crassostrea gigas] | EKC25979.1/0.003 | Internal repeat |
| **Unigene9334** | 4.1951 | CPNPPMLNLISVGGQHQGVYGFPR | 1 | DKMADNMKTSIVCFLIVLAISGIHSTTPVVLWHGMGDSCCNPLSMGSIKKLIEKKVERVYVNSLEIGSNIEEDTLNGFFLNVNTQISMVCDMLAKDSKLKGGYNAIGFSQGGQFLRAVAQRCPNPPMLNLISVGGQHQGVYGFPRCPGNNETICNMVRELLNYGAYVDQIQDRLVQAEYWHDPLNEDEYKKKSVFLAEINNENVKNATYKTNLLKLKNFVMVKFLDDGMVQPRDSEWFGFYNAGQAKTVYNLTQSKLYTEDWLGLQQLDKSGRLHFLSSPGDHLQFTEQWFIDNIINKFLK | palmitoyl-protein thioesterase 1-like [Mizuhopecten yessoensis] | XP_021340150.1/5e-156 | Palm_thioest /PF02089 |
| **Unigene58006** | 2.0793 | KVVISAPSK;LTGMAFR | 1 | IPWGSVGADIVVESTGVFTDKDKAAAHLKGGAKKVVISAPSKDAPMFVVGVNEHEYKSDLCIVSNASCTTNCLAPLAKVINDRFGIVEGLMTTVHSITATQKTVDGPSAKDWRGGRAASFNIIPSSTGAAKAVGKVLPALNGKLTGMAFRVPTVDVSVVDLTVRLEKKATYDQIKAAIKEESEGKMKGILGYTEDDVVSTDFIGDNRSSIFDAKAGIALNENFVKLVSWYDNEWGYSSRVIDLIVHIAKA | glyeraldehyde 3-phosphate dehydrogenase [Rosa multiflora] | AEQ75490.1/4e-177 | Gp_dh_N（SM000846）；Gp_dh_C（PF02800） |
| **CL758.Contig1** | 4.1951 | LTGMAFR;VPVPDVSVVDLTVR | 1 | MKVGINGFGRIGRLVMRAAIDKGVSVVAVNDPFIDLDYMVYMFKYDSTHGCFNGTVEAKDGKLIINGNAVAVFGERDPANIPWGANGAEYVVESTGVFTTKDKASAHFKGGAKKVVISAPSADSPMFVMGVNEEKYTKDLTVVSNASCTTNCLAPLAKIINDKFGIIEGLMTTVHAITATQKTVDGPSMKDWRGGRGAAQNIIPSSTGAAKAVGKVIPELNGKLTGMAFRVPVPDVSVVDLTVRLQNGASYDNIKKAIKEASEGPMKGIMGYTEDDVVSQDFRGDNRSSIFDAKAGIALSETFVKLVSWYDNEYGYSCRVIDLLKHMSKVDSA | glyceraldehyde-3-phosphate dehydrogenase [Littorina littorea] | AJA37895.1/0.0 | Gp_dh_N（SM000846）；Gp_dh_C(PF02800) |
| **Unigene4173** | 2.0605 | FEFDDIHR | 1 | MMIVILFLLLQAFLGSTAPSDDSCGISGRWRNDLGASMIFTCEEGNVVPTEGYLTGRFEFDDIHRYDLSGRYTMVGGDVVLGFSVAIGNDDGDSSVASLTGIHYTTEDSIHSKWLLSRSTEYNDMWKNTHFGSSVFIRVK | tamavidin1 [Pleurotus cornucopiae] | BAH20870.1/8e-10 | Signal peptide(1-17)/Avidin(PF01382) |
| **Unigene18442** | 4.1951 | FLTGDSAQHEER | 1 | KFLTGDSAQHEERNHMHVGKKFRFPTVPTLLRPPENKSIQPRIGPSRHGVTKKAHPKHEPIFVDWSDFEDTDEEYYYDDESASFEESSANRDTEFTAREIVAGSMYADSVKAKEVHVRNVIANDVVVSKTLG | SKD-rich protein-1 [Mytilus coruscus] | AKS48175.1 /0.083 | — |
| **Unigene22100** | 4.1951 | NIVEAAAVR | 1 | NMTKKRRNNGRAKKGRGHVQPIRCTNCARCVPKDKAIKKFVIRNIVEAAAVRDITEASVFDPYVLPKLYVKLHYCVSCAIHSKVVRNRSCEARKDRTPPPRFRPAAGAPRAPPKPM | 40S ribosomal protein S26 [Fundulus heteroclitus] | XP_012719203.2 /2e-75 | Ribosomal_S26e(PF01283) |
| **Unigene38646** | 4.1951 | IDPWAVVVR | 1 | GSCPIPHNSKVKLVVKTKTGELVDRIDPWAVVVRRPKDVPVFEQIFWNPPHDQVYQFKHRRPQKPASLRIYESHVGIASHEGRVATYSEFTRDILPRVHSLGYNSIQLMAIMEHAYYASFGYQITSFFAASSRYGTPEELKEMIDVAHSLGIVVLLDVVHSHASKNVVDGLNQFDGTNSCYFHDGGRGNHDLWDSRLFNYTE | 1,4-alpha-glucan-branching enzyme-like isoform X2 [Crassostrea virginica] | XP_022302818.1 /9e-108 | Alpha-amylase(PF00128) |
| **Unigene34096** | 4.1951 | TDVLLDINR | 1 | TTNWIDGLHQQSGNFNLYLAGNEFSCVCDYKSFIDWLSRTDVLLDINRNYSCTFPNGTRIRIPEVIQNYHRIFSHCNAIAWLRTGVICIVSSFFVIGLTAIIYQFRWRFTYFMYRQLKSRYIKEDPFVFDFVYDVFVAYANDCSEWLVESLIPTLEQEWNLNVCIKDRDFPIGADRGDTVVQS | toll-like receptor d [Mytilus galloprovincialis] | AFU48616.1 /1e-64 | LRRCT(SM000082);SCOP(d1fyva_) |
| **Unigene42863** | 4.1951 | ASANAAAFLQWIPAVGWR | 1 | MRQLVLVALLGFVTNTYAGCSFKASANAAAFLQWIPAVGWRSFSCAPGLFFNPGLCCCAPFGVGASAAASASAAAGAGGSAAAAAAAAAAAAAAAGLGVGAGAGALAGAGALAGAGALAGAGALA | — | — | Signal peptide(1-18)/SCOP d1gkub1 |
| **Unigene27658** | 4.1951 | VPIPTIFVGSVQR | 1 | MEYAMMRSMGAVDENTIIITTVHDCQVMDFPDNLIEDHDVVVDYIVTPSEIIKCNRTRSKPTGIIWSKITPDKLRRVPILRKLMKMEEANGKDVTLKDGAAPPEIKTEDEGEESDRQNRRRPFNRRFRRKPFNRRNRRERNSENETEGRGEGKDESEAKSGDEENQRQRRPQRRRRFRRRRNTSENKENEEPRSGDDRRNRRSFGESENDEGKSGDEDKSRRRRRPPPRRFRRNNRRSEGEEGGEDQGQGQSPRRRRGPPRVPIPTIFVGSVQRSVRISELKSKIREKNVNPLRVIWNGAKCYALLQFPKMNEMEEAFDTLNGFEMNGRKLKIEISNRVKQFMDEQHGQGEGHAQVAQDDQPEN | methenyltetrahydrofolate synthase domain-containing protein-like [Mizuhopecten yessoensis] | XP_021347230.1/6e-66 | RRM(SM000360) |
| **Unigene38586** | 4.1951 | SSSEVPWGFR | 1 | METIWLQRSSSEVPWGFRLQGGREFAQPLSIQKVTPGSVAGNVLVPGDILLKIGNNDVTNVSHNQGQDLIRYSGDLLQLTIKRCPRVAASAPVSPTPISQYNYQHSESPSSFRSLSPISDNFPMYQQGYPYQPRMYQEDRESPRQFASLPR | PDZ and LIM domain protein Zasp [Mizuhopecten yessoensis] | OWF52989.1 /1e-46 | PDZ(SM000228) |
| **CL2072.Contig1** | 4.1951 | LDYVLGLR | 1 | MPRVPLVQSKTFTTPRRPFEKERLDQELKLIGEYGLRNKREVWRVKFTLAKIRTAARELLTLDEKDPKRLFEGNALLRRLVRIGVLEESKMKLDYVLGLRLEDFLERRLQTQVFKLGLAKSIHHARVLIRQRHIRVRKQVVNIPSYIVRLDSQKHIDFSLRSPYGGGGPGRAKRKNMKKAQGGGGDEDED | ribosomal protein S9 [Haliotis diversicolor] | ALS46559.1 /2e-120 | Ribosomal_S4(SM001390);S4(SM000363) |
| **CL2084.Contig1** | 4.1951 | ELSELQDQLTGSTSSR | 1 | MDTKCVFLLLCAVTMVIGSSIRKKRSPANPLTKSEVSYYLKELSELQDQLTGSTSSRKRDRSSKRRKNKHEEQWRAGKSWWEQKHGKNKKSGRSRSWSSMSFKCDQPLSYGNTDCTEAVVRYYYERESKMCREFHYTGCGGNRNNFRSLSECKQKCKR | protease inhibitor-like protein-C [Mytilus coruscus] | AKS48156.1 /4e-40 | Signal peptide(1-18)/KU(SM000131) |
| **Unigene34486** | 4.1951 | LTNLTASVLK | 1 | LTNLTASVLKLKGTICFKCSKVGGDMMLNVAFIGRPDVKVQGKPVNPYQDPSDLVDIEVVEEVVRNAICLASTDINLTKWLTGSTQNLPRPDSTAKPSSPRHVQEPVFETMEAKETQSAFHAPPQQRVQRTSSTDKRLLVKIIKASGLGSKE | C2 domain-containing protein 2-like isoform X11 [Crassostrea virginica] | XP_022321168.1 /7e-48 | — |
| **Unigene19314** | 4.1951 | AQAQIAAR | 1 | MAEPLKVLITGAAGQIAYSLLYSVAKGDVFGPDQPIILVLLDIPPMMGVLEGVVMELMDCALPLLKEVIPSVDGMTAFKDVDVAMLVGAMPRKEGMERKDLLAANVKIFKAMGAAIDSQAKKTIKVVVVGNPANTNALICRKYAPSIPAENFTALTRLDQNRAQAQIAARLGVANNSVKNCIIWGNHSSTQYPDVNHAVVTKDGNTQAVRAAVQNDDWLNNEFIKTVQQRGAAVIKARKLSSAMSAAKAIVDHVRDWWNGVPEGSIVSMAVPSDGSYGIEEGLIYSFPVRTKPNHSYEIVQGLSVDDFSREKMELTMKELKEERDMAVSACDS | cytosolic malate dehydrogenase [Mytilus californianus] | AAZ79368.1 /0.0 | Ldh_1_N(PF00056);Ldh_1_C(PF02866) |
| **Unigene30568** | 2.0188 | AFNMIFSR | 1 | CLSVSVKGTPSKDNMGVINDVRNLDQNLTAVTPKRSVQVPGETSRENIPKRFGNGTGFTRLLPKVFEYFGAGTYPKNYNPRVHGPYYPFRYYGKADPVMECKVGDLKSWFLRREKGVRAFNMIFSRAWWNLSARYFHVAKPALYVVYFYVAFGMLNNLFNQYGKYSNNRHAKYR | putative ATP synthase subunit f, mitochondrial [Athalia rosae] | XP_012266791.1 /1e-17 | WRW(PF10206) |
| **Unigene44042** | 4.1951 | EECGWSGISQLTCEAR | 1 | QNLFVRQGVSNNQSVGPRNIFEDKMKLFAVLLVASVSYGMTLGNDETCEVQPPYRKECGWVGISQETCEARGCCFNSSTSGVKWCFQKAKLFSDNPECAVQPSNREECGWSGISQLTCEARGCCFDSSMSGTKWCFQKEKEFSNDTNCDVAPIFREECGWVGISLQTCEASGCCFDSSISSAKWCFQTAKPECWSLPNERRECGWPGISKRTCEARGCCFNSNTENTKWCFHKKQ | putative gastrointestinal growth factor xP4 isoform X1 [Stylophora pistillata] | XP_022785025.1/3e-45 | PD(SM000018) |
| **Unigene2679** | 4.1951 | EVDIGIPDATGR | 1 | MADGANEDLQTAILKRKDRPNRLLVEEAINEDNSVVSLSQGKMDELQLFRGDTVLLKGKRRRETVCIVLSDDTCSDDKIRINRCVRNNLRVRLGDVVSIQACPDVKYGKRIHVLPIDDTVEGLAGNIFDVFLKPYFLEAYRPIKKGDIFIVRGGMRAVEFKVIETDPSPYCIVAPDTVIHCEGEPVKREEEEEALNEVGYDDIGGCRKQLAQIKEMVELPLRHPQLFKAIGVKPPRGILLYGPPGTGKTLIARAVANETGAFFFLINGPEIMSKLAGESESNLRKAFEEAEKNSPAIIFIDELDAIAPKREKTHGEVERRIVSQLLTLMDGLKQRSHVIVMAATNRPNSIDAALRRFGRFDREVDIGIPDATGRLEILRIHTKNMKLSDDVDLEQVASETHGHVGADLAALCSEAALQQIREKMDLIDLEDETIDAEVLDALAVTMEDFRWALSKSNPSALRETSVEVPNVTWDDVGGLENVKKELQELVQYPVEHPEKFLKFGMTPSKGVLFYGPPGCGKTLLAKAIANECQANFISIKGPELLTMWFGESEANVREIFDKARSAAPCVLFFDELDSIAKARGGNSGDGGGAADRVINQLLTEMDGMSAKKNVFIIGATNRPDIIDPAILRPGRLDQLIYIPLPDLKSRVQILKANLRKSPVSRDIDLEYLAKVTEGFSGADLTEICQRACKLAIRQAIEAEIRNERERQNNPDLDMETEDYDPVPEITRAHFEEAMKFARRSVSDNDIRKYEMFAQTLQQSRGFGGNFSFPGSGGSNQPNQGGSGGGNFQDDGDDDLYS | Transitional endoplasmic reticulum ATPase [Mizuhopecten yessoensis] | OWF38174.1 /0.0 | CDC48_N(SM001073);CDC48_2(SM001072);AAA(SM000382);Vps4_C(PF09336) |
| **Unigene13309** | 4.1951 | GILGNSGGSGGGLLSR | 1 | QLLRAQPQGSTENLWEDELNNTMRILSIFVFLAALACADALLSDLLRLQVLKGILGNSGGSGGGLLSRLGQSSNGAQSASVVSSSSAGSYMENYYKLQYCRETPFRFIRKCTTSSQCSPYLECFENVCCATNPLSLRIVD | — | — | Leu(15.0%);Ser(13.6%) |
| **CL1361.Contig2** | 4.1951 | VPINTGAAVMDTYR | 1 | MHFTEVLVLCLIALCASKPTREKRQEEFQVDSFLEEFGYMDELGDTTGGHDASDRERAIREFQTMTGLPVTGELNKDTVAKMKAPRCGVKDVLRPSERPGELNKDPKSVQLKPLGNVYKWQNNKVSWKIMGYTRQLGSTSQRRSYINAFKKWTDVTPLDIREVSSGDADILISYARGNHYDGSPFDGRGRTLAHAFFPGTHDISGDTHFDDDEQWTLGTKTGTNLEIVAAHEFGHALGLGHSSDSKALMAPYYQGYDPNYGLNNDDIRRIQTLYGSRRPPSRTTRPPSGTTRRPSTPKPEICNLKFDSIFYNVEDRRVYPLRYRKIYKLDNDNNGIEKSFTRVSRKVLTPRVPINTGAAVMDTYRRLYIFKGNKLFRYTRWRLDSGFPKRISDPFFQKIDAAVVYMSNIYVFSDDKFSIWHESYTRPPSGYPKAISSFWNGIPNNIEAAVSINDGYTYFFKGLKYFKFNNSYRRTEYRKDKAPAWLGCGRPVPK | matrix metalloproteinase-14-like isoform X2 [Crassostrea virginica] | XP_022345031.1 /3e-163 | Signal peptide(1-17)/PG_binding_1（PF01471）；ZnMc（SM000235）；HX (SM000120) |
| **CL3814.Contig2** | 4.1951 | AEDPVPEEDYEYTSVR | 1 | MPPPIAGHTPRKVTLNKFGGGTTSFGQSFQSNKKSSTTWQPVPAPAGGSMMNRVQDSLDSALSPTSPPQGYYQQQYQQPQQQQYRPPPQQQQYRPPPQQYQSPSQQQYQPPPQQQYQPPPQQYQPPQNQYQPQPQQEPYTPTYQTVGDLQPDYVRAEDPVPEEDYEYTSVRDRKKQFIETRQDAPLIKRGKKKFVPPVAAAYQSFGTDYSSPQPKQVEQPRFPPAPKPVPPPVNRAPEPVDQQDGPKPWAGSLRSESGGPKLWELEDKEYIMPSQLEARQQQQQQQRQSRGQTRQQRQPPAVSPKPISKGTNQIKVAVAPPQQSPARQISVRTSSTVSKQQPQQSQQQGDRDWNQSYVYKMVKEETKRETQMYPGQAPITTQTYSSKTYQSGQPAQEDTYGISDF | PDZ domain-containing protein-1 [Mytilus coruscus] | AKS48171.1 /4e-91 | Gln(19.5%)；Pro(15.6% ) |
| **Unigene55910** | 2.7302 | QQVSLLIR | 1 | MDPKKMGEALGRMKGGGGRIGGLGLGLLAAGGAIYGIANSFYTVDGGHRSIIFSRIGGVQKNIYPEGLHFRVPWFQYPIIFDIRAKPKKLTSPTGSKDLQMVNISLRVLYRPDQKMLPEIYRQLGLDYDERVLPSICNEVLKSVVAKFNASQLITQRQQVSLLIRRELMERAQDFYMILDDVSITELSFGKEYTAAVESKQVAQQEAQRAQFLVEKAKQERQQKVVQASGEAEAARLIGEAISLNPGYLKLRKIRASQSIARTIAQSQNRIYLTANNLMLNLSDKDFDSIVDQYSKKK | prohibitin-2-like [Mizuhopecten yessoensis] | XP_021372589.1 /3e-169 | PHB（SM000244） |
| **Unigene38688** | 2.8885 | YTIEEPR | 1 | VKVSSPKREDLLCSDFSDFYKMSDYHRPDAKTLQTLKDIANKLRVHSIESTDASNSGHPTSCCSMAEVMSVLFFKTMRYTIEEPRGPSNDRFILSKGHAAPILYAAWAEAGLFNAADLLNLRKLESDLEGHPTPRLNFVDVATGSLGQGLSCAVGMAYMGKYIDKASYRVYCLMGDGESAEGSVWEAMAFASYYKLDNLVAVFDVNRLGQSEPTSLQHDMATYKARAEAFGFNTYVVDGHDIEALCKAFHDASTVAGKPTCLIAKTFKGKGIPGIEDQMNWHGKALGKESANALKAIKENIANQGPHGLKPGTVVNDAPVVNIRNVQLSEPPSYQKGQMVATRAAYGTALVKIGKNNNRVVAMDGDVKNSTFSIKFKEAFPDRFIECFIAEQNLVGAAIGCACRDRSVVFVSTFAAFFSRAFDQIRMGAISQTNVNFVGSHAGVSIGEDGPSQMALEDLSMFRSIPGSTVFYPSDAVSTERAVELAANTPGVTFIRTSRPNTTVRYDNDEPFAIGKAKVVRQSNNDAVTVVGCCVTLEEALKAADKLAADGVNIRVIDPFTVKPIDTETIINSARATGGKIITVEDHYPFGGVGEAVCSAVRECKDITVKMLAVQEVPRSGKSAELIEKYGISANCIVKAVNQVLSQ | Transketolase-like protein 2 [Crassostrea gigas] | EKC40969.1 /0.0 | E1_dh(PF00676);Transket_pyr(SM000861);Transketolase_C(PF02780） |
| **Unigene45227** | 4.1951 | FTPGTFTNQIQAAFR | 1 | MSGGLDVLSLKEEDVTKFLACSTHLGANNVDCQMEQYVFKRKPDGVYIINLRRTWEKLLLAARAIAAIENPADVCVISARPYGQRAVLKFASATGATPIAGRFTPGTFTNQIQAAFREPRLLVVTDPRTDHQPVTEASYVNIPVIALCNTDSPLRYVDIAVPCNNKGTHSIGLMWWLLAREVLRLRGTISRDHPWEVMVDLYFYRDPEEAEKEAEAAVEKAPQKDEAAIPDQWNTQAEIAPPLESSDWANEAMPVVPAPAAGGAAPFDIGAAPAPTEDWSASADDWSATPAPPSTTADWGGSNAENWN | 40S ribosomal protein SA-like [Crassostrea virginica] | XP_022313762.1 /3e-162 | Ribosomal_S2（PF00318）；40S_SA_C (PF16122) |
| **Unigene26518** | 4.1951 | SVLVDLEPGTLDYAR | 1 | GNQIGSIFWEMISGEHGIDDTGKYVGDSDLQLERIDVYYMEGSHKNYVPRSVLVDLEPGTLDYARSSPYGQLFRPDNFVGGYAGAGNNWAKGHYTEGAEMVDTVLDVIRK | Tubulin FtsZ domain containing protein [Haemonchus contortus] | CDJ88214.1 /1e-57 | Tubulin(PF00091) |
| **Unigene8735** | 2.7302 | TVFVEVNR | 1 | MGVMTGVARRFSLNCLLLILACLPTFAQVPPGAPSITGPQVIIMGNQYTLICTVNGGIPTPTVKWLRDDSVIDDSYTTVGQVTTNEYTFTASSAEHLEVFECQSENGILQNPLSRTVFVEVNRAPSPPVLSGPTDILSGTPTTWTCRSEGAYPQQSMQMRVGNNVFANEFSSNVVFDTVAESYTMTGTLNWAPTMVNNGNTLFCDVFHPATLGANAPQTVSLPLTVRSPLAVNAPNTLYTPKVGNPVTLQCVVTSGTATQIRWYKNNQLLNLIGNARYSGATPSNPSLTINQVILTDSGNYICEGSNANTFRNTSVITVSPRSTPSTPILNGPTQLTQGETGTWTCTSRGGYPEQRMGMRIGNSNFNNELSVQSVFDNTARTYTLTGTLVWAPNMGHNGMTVYCDVTHPETLAAPQTVSLPLTVLSPLSVNAPTTFYNPTEETSVTLTCVVTSGTATGIRWYKNNQQMSINGRFSGGNTANPSLTITNVQLSDGGNYICEATDGFSSPRTNTITVQVQEKPATEYYIITTVLDGYNKLVRPIDPVNNITHSLIPMEIMDFDKKKLVMQAMQCMNWNDPRLSWTRGQPRISLPASVIWIPDIVLYGQKYMPDFDTKVIVMKDGSVTYCPQGPLMATDCTEKAGEKYECTFKFLSWSYDKVMLDKYFPGFLQRPSIDMTVYREHHEYEIVSKCAIRNEMTYPCRIGTYPQLTYTFVLSRDRNFCINRSMNPTCN | egg surface protein-1 [Mytilus edulis] | ATL23494.1 /0.0 | Signal peptide(1-27)/IG_like(SM000410);IGc2(SM000408); C2-set_2 (PF08205);Neur_chan_LBD (PF02931） |
| **Unigene40520** | 2.2753 | GPFVVALGK | 1 | PSPPAPGGPRGGAPPKPTGVGAVRAKKGEASMFKSGDAAPGGNRIPTCNSCGMNIRGPFVVALGKTWCPDHFVCANPRCGQKLLDIGFVEEGGFLYCEKDYELYFAPHCAVCNNAIVGECVNALQKTYHPKCFTCAHCRQPIGGNQFHLEEGKPYCENDWRQLYQTMCHSCDFPVEPGDNWVEAMGKNYHSGCFNCSTCQINLEGQPFFAKGGKPYCKQHAR | PDZ and LIM domain protein 5 [Crassostrea gigas] | EKC26352.1 /4e-125 | LIM(SM000132) |
| **Unigene43422** | 4.1951 | AGVLAGHDNR | 1 | MSSELEQLRQEAEQLKNQIREARKAAADTTLAHATANVDPVGRIQMRTRRTLRGHLAKIYAMHWASDSSNGLNLVSASQDGKLIVWDGYTTNKVHAIPLRSSWVMTCAYAPSGSYVACGGLDNICSIYSLKTREGNVRVSRELPGHTGYLSCCRFIDDNQIVTSSGDMSCALWDIETGQQTTAFTGHTGDVMSLSLSPDFRTFVSGACDASAKLWDIRDGMCKQTFSGHESDINAITYFPNGYAFATGSDDATCRLFDIRADQEIGMYSHDNIICGITSVAFSKSGRLLLGGYDDFNCNVWDVLKQERAGVLAGHDNRVSCLGVTEDGMAVATGSWDSFLKIWN | guanine nucleotide-binding protein subunit beta [Crassostrea virginica] | XP_022343829.1 /0.0 | WD40(SM000320) |
| **Unigene9746** | 4.1951 | AYGPGLSEGVCNQPAR | 1 | PTKTSKIRAYGPGLSEGVCNQPARFTVETNGEVGALGFSIEGPSEAKIDCQDNGDGSADVTYYPTSPGEYAVHILCNDEDIPESPYMAQIAPATNAFDASKVIAEGPGLQKTGVTTNKYAEFTVDTRKAGKAPLKITCEDDQHKPVNVEIVDKKNGTFACKYMPKKQCKHTVTITYGGVQIPKSPFKVNVGEVSNPGNVKVYGPGVEKGVKTFKTTYFIVDCKSAGPGDIAIALVDAQGKDVPVNTIDQKDGTFKIEYTPNSPGTYIVSVYFANQEIPKSPIKVNVESSIDLSKVKVVGLDTPIKIGEKRDISVITKGCGKADGPVKVTMITPSKKKVNIPVKESLETWKG | filamin-like protein-3 [Mytilus coruscus] | AKS48150.1 /0.0 | IG_FLMN(SM000557) |
| **Unigene27944** | 4.1951 | TELEEIAAENPGR | 1 | HIFVTVAANRSMGRALTVGLPVAIGLGVVAVTAILAKIFLFGNKKKKSPVTLEDPEKKFPLKLIDKEEVSPDTRRFRFALPSPEHILGLPIGQHIYLTARIDGQLVIRPYTPVSSDDNKGFMDLVVKVYFKNVHPKFPDGGKMSQYLENMDIGDFIDVRGPSGLCVYDGQGVFKIKPDKKSEPETVMAKKLGMIAGGTGITPMLQLIRACFKDRNDTTQIFLLFANQTENDILLRTELEEIAAENPGRFKLWFTLDRPEDDWKYSKGFISADMIKDHLPPPGDDTLILMCGPPAMINFACLPNLDKLGYTPKMRFAY | NADH-cytochrome b5 reductase 3-like isoform X1 [Crassostrea virginica] | XP_022332541.1 /1e-178 | FAD_binding_6(PF00970); NAD_binding_(PF00175) |
| **CL2920.Contig2** | 4.1951 | VADEISTIAAR | 1 | SMLGITNIINCTLDVPDFHIPGVHCTSIRVDDHPQARLDLFFDRVADEISTIAARGGKVIVHCVAGVSRSASLCIAYLMKYHRMSLVKAHDYVKRRRSMIRPNMGFWRQLIDYERKLYRRTTVQMVPSNIGLIPDVYTEQARPTPRSPAWTPSSSYSYSPATSFSRSPSTTYSRSYGRHTWSRPRLGF | dual specificity protein phosphatase 14-like [Mizuhopecten yessoensis] | XP_021362799.1 /9e-60 | DSPc(SM000195) |
| **Unigene23964** | 4.1951 | YDFILSANQPIR | 1 | RLEGYHDHPCIRQCEEDEEPKECTYDFTVEYYYTLTQACYDCPYNITDCLRPHCVSADGISRGLITVNRMLPGPSIHVCKGDTVVVNVKNKLEGGEGVSLHWHGVLQEGTPHMDGVSMLTQCPIHPYTTFQYRFKANDPGTHFWHAHAGLQRTDGIFGSFVIRQPRAHDPHSILFDDDLPEHTIMLNDWMEHIGAVSFAAGHHAMKDHFPTSILINGKGTVKEFNEMMDHSAHKDSALPEDMIMDTLTTTRTMEMNHVDQMFEHMMHRRSSENQEMPPSHNMDNMPHSMTDLDARHTPHSIFNVKQGKRYRFRVISNGIANCPIQFSIDHHSLTMVTTDGSPFNPITVESFTVFAGERYDFILSANQPIRNYWIRARGLAECGPQYKSVSQTAFLTYEGSAIVLPQEPPDYHSGNRPGLMLNPLNIGPSRGFITVD | laccase-2-like [Crassostrea virginica] | XP_022312912.1/2e-128 | Cu-oxidase_3 (PF07732）;Cu-oxidase(PF00394) |
| **Unigene55607** | 4.1951 | FVNDFDSIVVSPIDR | 1 | GLHRKLKNLCFILILTSVVANAANLGDIANSLKAGYDVQRNGIPLQVGPTTMTTYLANITLENIGKTPIPVDGWSLNLCHEKLIHPMFFNISTLQYAGGGLILPGNFTVTHGKGCMFSFKPYTGPNANFKARPIQPGEKVVIPIVASDFSVSKYTTYPNWYLSNDTHTNLIPNTMDYKNHDFVGYFGQDIQYTRFVNDFDSIVVSPIDRYRGFNFSTKKQTQSEIL | putative beta-hexosaminidase [Mizuhopecten yessoensis] | XP_021345502.1 /5e-41 | Signal peptide(1-22)/CHB_HEX(SM001081) |
| **CL3668.Contig2** | 4.1951 | IDNGQNQPAVR | 1 | GRCTKFGYGGCDGNANNFATREACGNKCGDAYTPDGTTSISPSIPKEQCQQPKDFGNGQGREVMYYYNPAVGDCQPFWYSGAGGNNNRFRNSTRCRTVCRRDYTRVPVVTSRPPVTTTPPTIPSPDPGTDVIITGNECDENSERGPCTNYTVKWFYNRSQGRCTRFWYGGCEGNGNKFDSEAACKARCVEKVYTGSETADLVDGNATVIIEQPDRSLLIECRGPQGTVAWYKNARILTSDQRFTVFENGSLFIAQLNKDDSGVYACRIDNGQNQPAVRRFRLQIEVPITIFPTPSTIVVRPGENAFLHCQAYGSPQPTVTWTKDGQSIASGGRYYMYQNGTLIISNTQQTDISDYTCTARNGRSSPAQRLVRLSLQESVSASIKPIDGRLREGENLYLACDSRGFPPPTVRWEKMGVELITTGRMTVNGPNLRITDLTLDDTGSYTCVVNNAEDSAKDSKSIQVIPKDVDIPECVDSASVTMCRLIVRAGLCGYTDYSKQCCYSCDQSKLRG | Papilin [Mizuhopecten yessoensis] | OWF36203.1 /9e-150 | Kunitz_BPTI(PF00014);KU(SM000131); IGc2(SM000408);PLAC(PF08686) |
| **Unigene49135** | 2.6269 | DLTSVQNLR | 1 | WANLQTVTAERAQSLGSAQEVQRFHRDIDETKDWIDEKDFALANENYGHDLASVQALQRKHDALERDLSALGEKVRDLDDSAKRLMQTHPEQAEQIYEHQKQINEQWNTLTAKADARKAKLLDSYDLQRFLSDYRDLTSWINSMMTLVSSDELAKDVTGAEALLERHQEHRTEIDARSGTFQAFEVFGQQLLQNEHYASDDVKNKLDELANAREELEKAWIARRMKLDQCLELQLFYRDCEQAESWMESREAFLSGDQVDGDNVESLIKKHEDFDRAISSQQEKIQALQLFADQLVGGEHYDSGAITDKRDQVLDRWSQLKDALIDNRSKLGEAQTLQQFSRDADEMENWLQEKLQIASDESYKDPSNIQSKHQKHQAFEAELAANADRLQSLLGVGQALIDQKQCAGSEDAVQARLESLASQWETLVSKSAEKSDKLKEASRQQTYNAGVKDMEFWLGEVEQMLASEEYGKDLASVQNLLKKHQLLEADVAAHEDRIKDLNSQADQFIDGGVWDAESLEVRKRTINERYDKIKECAIVRRNRLNEANTMHQFLRDIDDEEAWIKEKKLLVGSDDYGRDLTSVQNLRKKHKRLEAELATHEPAIQAVQETGAKLIEESDINTADIKARLDQLDRSWEELKQMSNTRGQKLDESYTFQQFCANVEEEEAWITEKQHLLTGGDYGETMAAVQGLLKKHEVFETDFQIHRDRCTEIKNEGEKLISEGNHNADGIAQRIAGLQDKLNSLSDAANRRKAGLIDNSAFLQFIWKTDVVESWIADKETQVRSEDYGRDLSSVQTLLTKQETFDAGLQAFEKEGIQTITALKDQLVAANHAQTPAIEKRYNDVMDRWQKLLADSDARKQRLLRLQDQYRQIEDLYLTFAKKASGFNSWFENAEEDLTDSVRCNSVEEIKALIEAHDAFKASLSAAQADFNQLAALDQQIKSFNVGANPYTWFTMDTLEDTWKNLQKIIKERDYELEEEQKRQMENDQLRKQFAQAANAFHSWLTATRSAMMEGTGTLEDQLEATKQKSSEVRSQKGQLKQIEDIGARMEERLILDNRYTEHSTVGLAQQWDQLDQLGMRMQHNLEQQIQARNRSGVSEDALREFSMMFKHFDKDKSGKLDHQEFKSCLRALGYDLPVVDEGQVDPEFQAILDMVDPNRDGQVSLQEYMAFMISRETENVQSSSEVVEAFRALTSGEKPYITAQELYANLTKEQADYCLQRMKPFVDKTGRTVPDAYDYTEFTESMFIN | Spectrin alpha chain [Crassostrea gigas] | EKC38224.1 /0.0 | SPEC（SM000150）；EFh（SM000054);efhand_Ca_insen(SM001184) |
| **Unigene14496** | 4.1951 | SNTLQNLHVQQPAR | 1 | GACCGAPPSPEMGMRSNTLQNLHVQQPARQQYETQASILTNFNEMANVYVDKNAQKTPAKNESDVLSPDYRPYLPPSTYSPFGPYGAPSQPNNGNYDQNAQIKK | — | — | Pro(12.5%)；Asn(10.6%) |
| **CL2840.Contig5** | 2.2079 | ASSYLDDIYYPEPIVR;EMEVEAVPVQSTTSLK;FQSVPPGYFSSTK;GFYDTTR;GFYDTTREENEIR;LVVYPISER;VEVVTPR;VTQPTNLMSWQYR;WYPTTTR;YLPDEDSK | 1 | MTVRRSRFQSVPPGYFSSTKGHSSLKRWYPTTTRASSYLDDIYYPEPIVRSRGFYDTTREENEIRRDVNHELLYTSNLVDDTYDIANKSRNRDQMLLREATRALPSTTTENKDVGKRTVSLTPARRFQAPLSSLYSRNPTPYPKLTPPMYRATTNIQPLGILTKLDWTSPLRKYEMGASLPELENGDDVSEYSTYAAPTETESVRSSPERDYEEGIRPRTRPKRKIRTVYRRMPRRTVRASKNVKYYPYLDTESVVSDDVGTINGEYMDDDDYQSTYCSEVGDGGDYYYVDKERSPSPLEYVPFAPRHKINLDDDEYKCQVPSVAEMAVSHFVPPKKDPDFDPDKSRIKNDLVFHDVVSQTASKARDALKNVNLDEYDSASLVISVEGEYKDPVKGENMGFQYIARPLALKGPMLAGPAVYASTASDSAFNRYMDDMRSFRAQIRHRLETGYKTLSNVSSNYRDHSYPILASSHRSYSRALSPPKSSSPAGRSVSPVRVPHTSYTAHSLPISNLRKRLEEIESKYKTMGYEKRLDDIPDRLVVYPISERASIKPMTATENLPGHTKTVELYEGSAKGNELSTLEKINIKAALVGNRVEVVTPRKRKPRSTYAANKMRELKRDEREMEVEAVPVQSTTSLKASTVSPNYKGGKLHWDEDGKVTQPTNLMSWQYRIESRVPPGDHLFPVKTIGHVRDKLLHVKEQMDRHRQLMDRYLPDEDSKTDVKTKIMNMYVDMEQHNPAS | RS-rich protein-1 [Mytilus coruscus] | AKS48138.1 /1e-80 | — |
| **CL873.Contig1** | 2.4029 | QLGWLPR | 1 | MAVISKLPREYGYVVLTGVASTFLLQWMTYKVIVARKKYEVPYPTLYSPTNEKFNCVQRVHQNTLEGYPTYLMLLFAGGLQYPKISAAAGCVWILGKIAYAFGYYSDDIQKRRYGGFGYFGLFTMLGCTIGFGVRQLGWLPRSCH | microsomal glutathione S-transferase 3-like [Crassostrea virginica] | XP_022337663.1 /6e-56 | MAPEG（PF01124） |
| **Unigene15445** | 2.7302 | GNVDVVVLDPHGR | 1 | KGNVDVVVLDPHGRKDTIRPSINLAPGKEKDGVYLVEYVPLEQGLHSVNVYFAGQQIPKSPFGVGVGAASNAKMCYATGRGIQPRGIRVNDKAPFRVHTKGAGNGEVKVQIFGPGGQEIKCVPQKSKTEEGVWECLYIPPKVGQYIINVTFGDQHITKSPFKVDVGPTKT | filamin-like protein-3 [Mytilus coruscus] | AKS48150.1 /4e-99 | IG_FLMN(SM000557） |
| **Unigene35748** | 2.0188 | TTTTGGTAVR | 1 | MFIVSVYFKVAFALICVGLVFELIGFASPYWYSTRVNNVGLWQSCDDRIYHGDQCLSYRATTAVKAARAFSAIALILFVVLVILLLLYWCQCPRSDLILASIIICFITAACVFIGIICWVTRFSSNLSWAFYLCVIAGILAVVAGVLLIPERRIIIIGRTTTTGGTAVRTVTTTRTVTVR | lens fiber membrane intrinsic protein-like [Mizuhopecten yessoensis] | XP_021368751.1 /9e-33 | Ile(12.2%);Val(11.7%);Leu(10.6%);Ala(10.0%) |
| **Unigene14389** | 2.6269 | VDILDPALLR | 1 | EKNKMATLEDKAIWDEGEESLGEDILKLNTDEIVTRTKLLDNEIKIMRSEILRITHDLAATKDKIKENTEKIKVNKTLPYLVSNVIELLDMDPVDSAEEDGANIDLDAQRKGKCAVIKTSTRQTYFLPVIGLVDPEKLNPGDLVGVNKDSYLILETLPQEFDSRVKAMEVDERPTEQYSDIGGLDKQIQELIEAVVLPMTHKERFEALGIQPPKGVLLYGAPGTGKTLLARACAAQTKSTFLKLAGPQLVQMFIGDGAKLVRDAFALAKEKQPAIIFIDELDAIGTKRFDSEKAGDREVQRTMLELLNQMDGFQPNAAIKVIAATNRVDILDPALLRSGRLDRKIEFPHPNEEARARILQIHSRKMNVSKEVNFEELARCTDDFNGAQLKAVCVEAGMIALRRGATELQHEDYMDAIIEVQAKKKANLQYYA | 26S protease regulatory subunit 6A-B [Mizuhopecten yessoensis] | XP_021341301.1 /0.0 | AAA（SM000382); |
| **CL3860.Contig1** | 2.0605 | REIDDNLGK | 1 | LRPEFMDVFGLFTIPGWLVVIPLMLFLTFCLYTLYSQTYFWRNGIPGPTPTPFLGHFTQYMKKGVIGTDIDIVRKYGKVIGIYFGNLQTVLVSDVELIKQLCIKDFSKCPNRTLPMGEDKYLKGAMSITRDENWKFIRSSLSPAFSSGKIRQMVPIMQHCIDSMVKILKEDTKNGEVIDICPYCGAYSMDVIASTAFGMDIDAQNNKDSEFIKNSKAAFARGFGNPFFLIIVFLPFLRFILPKLNFSFFPKKVADFFNNAVGSVIADKKADVNARQDLLWLMMNAHKDIGKAEDEVEYTSESRFEFKKRPLTNQEILANSLIFFLAGHETTANVISFVMYSLALYPDYADELRREIDDNLGKDKPTYDNVYKLPFLEQFMSEVLRLYGSAARFNRILEEDIVLNGYKIPKGTDVHFPVTAIHRDPELWPDPEVFDPSRFSPDNKERIHPYAWVPFGVGPRNCVGMRFALTELKMATAALVQNFTIVKCDETEVPITFHRGGFVKAQNGIKVKLQSR | cytochrome P450 3A11-like isoform X1 [Crassostrea virginica] | XP_022304770.1 /2e-147 | p450（PF00067） |
| **Unigene56883** | 2.0605 | AGLQFPVGR | 1 | GKDSGKTKTKAISRSQRAGLQFPVGRIHRHLKSRTTSHGRVGATAAVYSAAILEYLTAEVLELAGNASKDLKVKRITPRHLQLAIRGDEELDSLIKATIAGGGVIPHIHKSLIGKKGQQKTV | histone [Limosa lapponica baueri] | PKU30505.1 /2e-80 | H2A(SM000414） |
| **Unigene48689** | 4.1951 | NLPDIPIHLTGR | 1 | MTGKKQNNTSPAINNSQPQHTASYKNFPISIGDHGKKFLPKPAVNIHALRNLPDIPIHLTGRAKTTLQELLQFAKPLDIVNPIKTRSKTLKNFRHKTVLAKKLSNIFRKQNRRRLHR | Transcriptional regulator, LuxR family [Granulibacter bethesdensis CGDNIH4] | AHJ65725.1 /8.6 | Lys(12.8%);Leu(10.3%) |
| **Unigene26867** | 4.1951 | IVVYLNK | 1 | FFPRTFFAIWGTSKYQIRQLLPRNRLTILTIWRNYCKVANSTSKPRCNVGTIGHVDHGKTTLTAAITKVLAKHGQSKLVTFDQIDKAPDEKKRGITINTAHVGYETSKRHYAHTDCPGHIDYIKNMITGTSQMDGAILVVAASEGSMPQTREHLLLAKQIGVDKIVVYLNKMDLVDDELGDLVELEMRELLEEYGYDSTKTPVIRGSA | elongation factor Tu, mitochondrial [Culex quinquefasciatus] | XP_001843948.1 /1e-84 | MMR_HSR1(PF01926) |
| **Unigene40538** | 4.1951 | QEQPVGLFGLGLGGLGGGR | 1 | MKTFVVVALVLCVMVAHVRSQGHSLDLFGMGMGNNMGNPGGGIGGSVSASTGAGAANSGSDAMFMKMMKLILLKLNKLEKQQRPPKQEQPVGLFGLGLGGLGGGRRGNSLHHHLLLEHLSK | — | — | Signal peptide(1-20)/Gly(18.2%);Leu(14.9%) |
| **Unigene3757** | 2.8885 | WAYISDTLGR | 1 | VTFLNFVFCDSHRSTQPSVVYEWETVDMDWPNETVRQNYYQNNKYIPKNNGINGIKLHKGDVYLTIPKLKDGIPFSLVKVVKDGTIRPLLQPYPNWDIHMEGDCSNMKLIQSMEIDPYSEFMWIIDTSFVPRGSVNIADQCPKKLIIWNLEKNTEVHRYSFPDSVIGPGMSYLNDIVIDFDKNKTARWAYISDTLGRKLIVYDRLLDDSWAFTHESMNPVPQYSNITIGNTTSDYSVLGINGISMSSDFRYVYYCPVAGIKVYRVSAKILRNKQSSDTDFANEVKIFGNKQYQSDGIYYGQKHNLYYSAQGPRSVYKWSVEFENGEVIQADRRSTVAFHNRIEWIDSFAFDDNGFLWFVTNNLNTFFSEDGKNGTSNFFVWKVYVDDASYLKFGDDTSSAIPHVVSVPTLLLSCFIFMYTK | Protein yellow [Mizuhopecten yessoensis] | OWF40019.1 /6e-138 | MRJP(PF03022) |
| **Unigene19623** | 4.1951 | TTGEMIANQIWR | 1 | ECNGSIQIVAPNSGGVSVSSSVSDRGLPVTTVCTVIWGQSEACTKVMNPDSSFATSINSMGNTLYVHLQSVAHDLGGNVGMFVNQMQTVVGDMNSTATNEEIERMLNELNFGLSGMQLELQNTLNGVENNVQQSMANVHQTVSQIQPNIDRIMSNINPGMRMTQMGSGLHQSLSNLGPSIRQQVNNNVGSMLYNLHHGLNRWRTNFGHSMNHFFSNLFGRKKRQAQVISHPSHEIQCEYMLKNSSVCAEYKTKCLSCPATPNVDDRDVIRQVCGDVIVDQVMQIDTKLAELANIEQMVVSVRQVIDQVEIDKALWNPNTFAFDSMFVTARIKGQPVRFRSKSELKIMDLKTTGEMIANQIWRYWNDQASYKPL | Sodium-coupled monocarboxylate transporter 1 [Crassostrea gigas] | EKC41285.1 /5e-17 | — |
| **Unigene44374** | 4.1951 | EGASGAPVEDQGGAETTR | 1 | LQPRVARWEKKEGASGAPVEDQGGAETTRWLNSGLNWFYLHYDYVPEFIDLWVKSLNEQVLKLGGPVQLKFERVKPGSLPPKFNEITFEAGTDNKYVVHCKVDSKDLSFAIFASQQTSGGVRLT | phospholipid transfer protein C2CD2L-like isoform X9 [Crassostrea virginica] | XP_022321166.1 /4e-51 | — |
